# Insight on Gaussian Basis Set Truncation Errors in Weak to Intermediate Magnetic Fields with an Approximate Hamiltonian

**DOI:** 10.1021/acs.jpca.3c04531

**Published:** 2023-12-16

**Authors:** Hugo Åström, Susi Lehtola

**Affiliations:** Department of Chemistry, Faculty of Science, University of Helsinki, P.O. Box 55 (A.I. Virtanens plats 1), Helsinki FI-00014, Finland

## Abstract

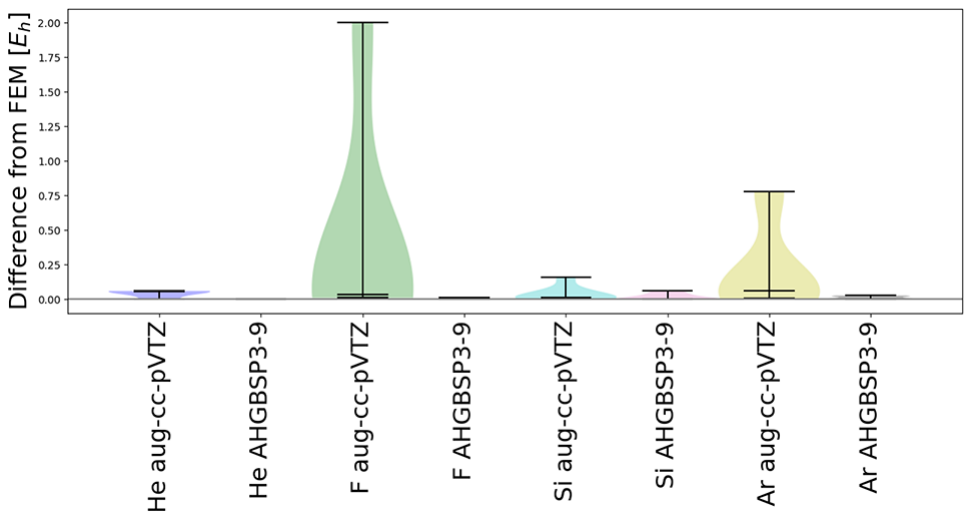

Strong magnetic fields such as those found on white dwarfs have significant effects on the electronic structures of atoms and molecules. However, the vast majority of molecular studies in the literature in such fields are carried out with Gaussian basis sets designed for zero field, leading to large basis set truncation errors [Lehtola et al., *Mol. Phys*. **2020**, *118*, e1597989]. In this work, we aim to identify the failures of the Gaussian basis sets in atomic calculations to guide the design of new basis sets for strong magnetic fields. We achieve this by performing fully numerical electronic structure calculations at the complete basis set (CBS) limit for the ground state and low lying excited states of the atoms 1 ≤ *Z* ≤ 18 in weak to intermediate magnetic fields. We also carry out finite-field calculations for a variety of Gaussian basis sets, introducing a real-orbital approximation for the magnetic-field Hamiltonian. Our primary focus is on the aug-cc-pVTZ basis set, which has been used in many works in the literature. A study of the differences in total energies of the fully numerical CBS limit calculations and the approximate Gaussian basis calculations is carried out to provide insight into basis set truncation errors. Examining a variety of states over the range of magnetic field strengths from *B* = 0 to *B* = 0.6*B*_0_, we observe significant differences for the aug-cc-pVTZ basis set, while much smaller errors are afforded by the benchmark-quality AHGBSP3-9 basis set [Lehtola, *J. Chem. Phys*. **2020**, *152*, 134108]. This suggests that there is considerable room to improve Gaussian basis sets for calculations at finite magnetic fields.

## 1 Introduction

The behavior of atoms and molecules in strong magnetic fields is of interest in astrochemistry and astrophysics, as magnetic neutron stars and white dwarfs exhibit magnetic fields with strengths of the order of 1 *B*_0_ ≈ 2.35 × 10^5^ T. Such fields are well-known to cause significant changes in the electronic structure.^1−5^ Moreover, as these field strengths are several orders of magnitude larger than what can be achieved in experiments on Earth, a computational approach is required to study their effects.

Many methods for performing quantum chemical electronic structure calculations at finite magnetic fields have been developed in recent years.^6−28^ These studies have revealed the spectra of white dwarfs,^29−31^ as well as a vast richness of new chemistry, such as the paramagnetic bonding mechanism of H_2_ and He_2_^32^ and the lattice structure of He atoms in strong magnetic fields.^7^

A central aspect of all electronic structure calculations is the choice of the one-electron basis set. In the weak field region, isotropic Gaussian-type orbitals (GTOs) are a good choice due to their long history in quantum chemistry: a richness of GTO basis sets has been developed for a variety of purposes.^33−35^ GTOs enjoy an overwhelming popularity in the literature also in finite-field calculations at various levels of theory, including Hartree–Fock (HF),^6,7,14,15,36^ density-functional theory (DFT),^8,11^ coupled cluster (CC) theory,^12,18,37,38^ and configuration interaction (CI) theory.^32,39−46^

However, the choice of the one-electron basis set requires special attention when studying atoms and molecules in magnetic fields: as was already mentioned above, the field affects the electronic structure. One of these effects is that when the magnetic field is turned on, the atomic orbitals that are spherical at zero field become cylindrical. Isotropic GTOs are therefore not well-suited for describing the electronic structure in strong magnetic fields, as we have demonstrated by the existence of large basis set truncation errors in diatomic molecules.^47^

An alternative is to use anisotropic GTOs;^48,49^ however, they introduce new types of challenges. As the magnetic field interaction confines movement in the direction orthogonal to the field (see section 2), the anisotropic GTO basis set splits the exponents in the directions parallel and orthogonal to the field, which complicates the optimization of the exponents.^50−52^ Moreover, because the basis set is formed by the product of these two sets of exponents, the number of basis functions explodes if the basis is required to be accurate for a range of magnetic field strengths. The use of anisotropic GTOs also requires dedicated approaches,^48,49^ and such basis functions are supported in few programs.

The wide support for isotropic GTOs in quantum chemical packages motivates their continued use at intermediate field strengths: as long as the field is not too strong, the cylindrical distortion to the atomic orbitals at the finite field can be recovered by including additional polarization functions, analogously to the manner in which the linear combination of atomic orbitals (LCAO) works at zero fields to model polyatomic systems in which the atomic symmetry is similarly lifted.

This begs the following question: can standard isotropic GTO basis sets be modified to better suit calculations in external magnetic fields? The first step toward the answer is to identify the failures in standard Gaussian basis sets, which is the focus of this work. We will introduce an approximation for performing finite-field calculations with Gaussian basis sets that is compatible with established Gaussian-basis methodology, that is, the use of real-valued basis functions and orbital coefficients. Employing this approximation and a computational approach the senior author has recently developed,^47,53^ we will study shortcomings of existing Gaussian basis sets by examining differences in total energies observed for the various low-lying electronic states of atoms.

We will carry out fully numerical calculations^54^ for atoms in finite fields with the Hartree–Fock (HF) method with complex atomic orbitals, enabling us to determine the complete basis set (CBS) limit for a number of single-determinant HF states over a range of magnetic field strengths. Similar calculations have been previously reported on the series of neutral atoms from H to Ne and their singly positive ions in a larger range of magnetic fields;^55−60^ in this work, we will examine the whole series of atoms from H to Ar and focus on weak to intermediate fields.

Equipped with the CBS limit data for the complex wave functions for a number of configurations, yielding numerically exact HF energies, we will determine the field-dependent differences in total energies in a wide variety of isotropic GTO basis sets for these configurations, enabling us to assess both the accuracy of the GTO basis and the employed real-orbital approximation. Especially, we rely on the large benchmark-quality GTO basis sets of ref (61) for insight onto the limitations of isotropic GTO basis sets. These large basis sets enable us to identify states that are poorly described by commonly used basis sets optimized for field-free calculations and thereby allow the identification of the kinds of exponents that should be included in future isotropic basis sets for finite-field calculations.

The outline of this work is as follows. We outline the theory behind the methods used in this study—the Hamiltonian in a finite magnetic field and the numerical methods employed in this work, in section 2; especially, the real-orbital approximation is discussed in section 2.1. We discuss the computational details in section 3, followed by the results of the calculations in section 4. We end the article with a summary and discussion in section 5. Atomic units are used throughout the work: the magnitude of the magnetic field is given in units of *B*_0_ and energy in units of *E*_h_.

## 2 Theory

We have previously discussed electronic structure calculations in the presence of an external magnetic field in ref (47). Following the same outline, we employ a Hamiltonian of the form
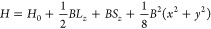
1where the terms linear in *B* are the orbital and spin Zeeman terms, respectively, which are responsible for the paramagnetic response that can either increase or decrease the energy of the system relative to zero magnetic field. The quadratic term in eq 1 leads to a diamagnetic response that always increases the energy of the system relative to the zero field case. It also acts as a confining potential for the orbitals in the (*x*, *y*) plane, which leads to the orbitals ballooning in the direction of the magnetic field, which is chosen to coincide with the *z* axis in eq 1.

The fully numerical calculations are pursued in atomic orbital basis sets of the form
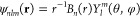
2where *B*_*n*_(*r*) are the piecewise polynomial basis functions of the finite element method (FEM), and *Y*_l_^m^(θ, φ) are complex spherical harmonics; we refer to the earlier literature for discussion on the FEM approach.^47,53,54^ The evaluation of the magnetic field terms in the Hamiltonian of eq 1 with respect to basis functions of the type of eq 2 has been described in ref (47).

The GTOs calculations, on the other hand, are pursued with basis functions of the type
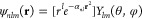
3where *Y*_*lm*_ are spherical harmonics of the real form. In accordance with standard practices of finite field calculations, we use uncontracted GTO basis sets to allow better flexibility to the wave function to adapt to the finite magnetic field.

We note that the use of London atomic orbitals (LAOs),^62,63^ also known as gauge-including atomic orbitals (GIAOs),^64^ is generally important at finite magnetic field. In the LAO/GIAO approach, one includes a magnetic gauge factor in the definition of the atomic-orbital basis functions
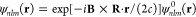
4where ψ_*nlm*_^0^ is the zero-field basis function centered at **R**, **B** is the magnetic field, and *c* is the speed of light. However, the gauge factor in eq 4 yields unity in the case of linear molecules in a parallel field—such as the case of the diatomic molecules previously studied in ref (47)—as well as in the present case of atoms where the basis functions are located at the origin. The calculations of this work and ref (47) are therefore of LAO/GIAO quality.

### 2.1 Real-Orbital Approximation

Even though the calculations in the GTO basis sets are carried out with real-orbital basis functions, while the magnetic field interaction matrix elements are defined in terms of complex GTOs, for the purposes of this study we chose to disregard this difference and carry out approximate calculations instead.

The real-orbital approximation employed in this work consists of reusing the magnetic field interaction matrix elements derived for complex-valued spherical harmonic *Y*_*l*_^*m*^ basis functions in the basis of the real-orbital *Y*_*lm*_ basis functions that are actually used in the GTO calculations.

While this approximation may seem coarse, it avoids the need to deal with complex basis functions or complex expansion coefficients altogether, allowing the reuse of field-free machinery. The approximation is also exact in a number of cases. The energy is exact for all states with only σ orbitals, since *Y*_*l*0_ = *Y*_*l*_^0^. The energy is also exact for states occupying π, δ, φ, ... orbitals, if both the |*m*| and −|*m*| magnetic subchannels are equally occupied, because the resulting density is cylindrically symmetric. An analogous approximation was used in ref (65) to implement linear molecule symmetry in Erkale, as it is exact for the Σ states that were considered in that work.

We will find below that this real-orbital approximation does afford an excellent level of accuracy for many states and that it captures the most important effects of the magnetic field on the basis functions.

## 3 Computational Details

The fully numerical calculations are performed with the HelFEM program.^47,53,65,66^ We employed five radial elements with shape functions determined by 15-node Legendre interpolating polynomials (LIPs) defined by Gauss–Lobatto quadrature nodes and a practical infinity of *r*_∞_ = 40*a*_0_, which was found to afford the CBS limit for the studied systems. The sole exception was the field-free (*B* = 0) calculations, for which the high-lying excited states are extremely diffuse, and the calculations used seven radial elements and *r*_∞_ = 100*a*_0_ instead.

All calculations in this work are performed at the unrestricted HF (UHF) level of theory, where all spatial and spin restrictions on the sought-for atomic orbitals are let go. The resulting atomic UHF configurations can be identified by their symmetry: the configuration is fully specified by the number of alpha and beta electrons for each value of *m*, which is the quantum number resulting from Noether’s theorem that describes the orbital’s symmetry around the magnetic field axis, as the corresponding angle does not appear in the Hamiltonian.^54^ The value *m* = 0 corresponds to σ orbitals, *m* = ±1 to π orbitals, *m* = ±2 to δ orbitals, *m* = ±3 to ϕ orbitals, etc.

To keep the notation more compact, we will denote the configurations with the following notation
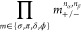
5where +/– indicates the sign of the *m* value, and *n*_α_ and *n*_β_ are the number of α and β electrons occupying orbitals with this value of *m*. As an example, the zero-field UHF ground state of F with orbital occupations of 1σ^2^2σ^2^3σ^1^1π_+_^2^1π_–_^2^ would be written out in our compact notation as σ^3,2^π_+_^1,1^π_–_^1,1^. Note, however, that the α and β spatial orbitals may differ in the UHF calculations!

The work began by identifying the atomic configurations of interest in the range of studied fields, *B* ∈ [0, 0.60*B*_0_]. An automated Python program was employed to find the 3 lowest states at each of the field strengths *B* = [0, 0.10*B*_0_, ..., 0.60*B*_0_]. The logic employed was to generate trial configurations from the current-lowest UHF configuration by moving one electron at a time, allowing changes in *m* as well as flips of the electron’s spin.

Because at this stage the states’ total energies are not of importance (only the relative ranking of the configurations is), while many candidate configurations need to be considered, this part of the study employed a smaller numerical basis set than the CBS limit calculations discussed above and described in detail below. Approximate total energies were computed for each group of candidate configurations by beginning from the smallest possible value of *l* able to describe all the configurations in the group, *l*_min_ = max_*iσ*_|*m*_*iσ*_|, where *m*_*iσ*_ is the *m* quantum number of occupied orbital *i* with spin σ. The set of three lowest configurations was then converged by increasing the truncation with the *l* quantum number by 2, that is, until the same set of three lowest-lying configurations is obtained with *l*_max_ = *l*_max_^0^ and *l*_max_ = *l*_max_^0^ + 2. If the lowest-lying configuration changes at this stage, then the procedure automatically restarts from the generation of a new set of candidate configurations.

In the second stage, calculations were performed at the CBS limit for all of the low-lying configurations determined in the initial step. The procedure to converge the numerical basis was the same as in the first part, but now the procedure is carried out for each state separately until its total energy converges to the threshold of 10^–6^*E*_h_; the most difficult states required *l*_max_ = 21 to reach this criterion for the CBS limit. Because HF energies are well-known to exhibit exponential convergence in the basis set, we are confident that our energies are accurate to μ*E*_h_ precision; this exponential convergence is also demonstrated graphically in the Supporting Information (SI).

We used a superposition of atomic potentials (SAP)^67,68^ as the initial guess in the FEM calculations. The only exception is the σ^2,0^ state of He, for which the SAP guess was found to lead to saddle point convergence at stronger fields, *B* ≳ 0.3*B*_0_, and for which the core guess was employed instead.

The Gaussian-basis calculations were performed with the Erkale program.^69^ Calculations were carried out with the double-ζ (D) to quintuple-ζ (5) correlation-consistent cc-pVXZ and aug-cc-pVXZ basis sets,^70−73^ which have been commonly used in the literature at finite magnetic fields.

Insisted by a reviewer, we also performed calculations with the def2-TZVP^74^ and the 6-311++G(3df,3pd)^75^ basis set for completeness, because these families of basis sets have also been used in some studies of the literature.^20−23,76,77^ The results for these basis sets are not discussed in the main text, but they are available in the Supporting Information. We note that the 6-311G Pople family of basis sets are obsolete, carry many problems,^78,79^ and should not be used.

Importantly, both HelFEM and Erkale are free and open-source software,^80^ and are publicly available on GitHub. As was already mentioned above in section 2, all GTO basis sets are employed in a fully uncontracted form.

As mentioned above in section 1, an approximate Gaussian basis set limit was determined with the benchmark quality hydrogenic Gaussian basis sets (HGBS) of ref (61). The HGBS basis sets are modular and determined with one-electron calculations, only: the basis set for angular momentum *l* of the element *Z* is determined with calculations on the one-electron ions *Z*^(*Z*–1)+^, ..., He^+^, and H.

Requiring that the energy of the lowest-energy orbital of each angular momentum converge to the relative accuracy 10^–*n*^ with respect to the number of even-tempered exponents on the shell yields the HGBS-*n* basis set.^61^ Augmented versions of the HGBS-*n* basis sets—the AHGBS-*n* basis sets—are obtained by extending the consideration to also the fictitious single-electron ion with nuclear charge *Z* = 1/2.^61^ Finally, polarized versions of the basis sets are obtained by adding higher angular momentum shells, the basis with *m* added polarization shells being denoted as (A)HGBSP*m*-*n* basis sets.^61^

In this work, we will examine the (A)HGBSP*m*-*n* basis sets with *m* ∈{1, 2, 3} and *n* ∈ {5, 7, 9}. Comparing data at fixed *m* and growing *n* demonstrates convergence with respect to the radial expansion, whereas comparison of data for fixed *n* and growing *m* demonstrates convergence with respect to the angular momentum expansion. The utility of the HGBS basis sets is exactly their modular nature: the basis sets can be straightforwardly determined for an arbitrary element, an arbitrary precision, and an arbitrary number of polarization shells.

We found many of the Gaussian-based calculations to be sensitive to saddle point convergence, even with the explicit handling of the orbital symmetry with respect to the magnetic field axis. This issue was diagnosed from discontinuities in plots of the total energy as a function of the strength of the magnetic field. We were able to circumvent most of this issue by calculating each state of each atom on a hysteresis curve. Starting from the converged field-free calculation, we ran calculations with increasing field strength by reading in the orbitals from the previous calculation as the initial guess. This gave us one set of solutions. In the next step, we repeated the calculations in the opposite direction: starting from the strongest field, we ran a new set of calculations in decreasing field strength by reading in the orbitals from the previous field strength as the initial guess. The reported energies at each field strength were then obtained by choosing the minimal energy of these two calculations.

Finally, given the CBS limit energy from the HelFEM calculation and the GTO energy from Erkale for a given state and field strength *B*, *E*^CBS^(*B*) and *E*^GTO^(*B*), respectively, we calculate differences in energy as
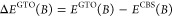
6In the case where the approximation discussed in section 2.1 is exact, the difference measured by eq 6 is a metric of basis set truncation error (BSTE) and Δ*E*^GTO^ is positive, Δ*E*^GTO^ > 0.

Note, however, that a positive Δ*E*^GTO^ value may also be obtained when the approximation is not exact. Indeed, we observe that this difference can take either sign for the configurations examined in this work. Such differences are usually observed already at zero field, indicating that the differences between the FEM and GTO calculations arise already from the differences in handling the orbital symmetries in these two programs, as discussed in section 2.1.

To simplify the analysis, we will furthermore average the energy difference over the magnetic field:
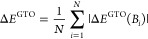
7where the average is performed with respect to the *N* = 7 considered values for the magnetic field strength *B*_*i*_ ∈ {0, 0.10*B*_0_, ···, 0.60*B*_0_}. A comparison of the mean absolute energy differences (MAEDs) defined by eq 7 allows a straightforward identification of states that are ill-described by the studied GTO basis.

## 4 Results

The results for all basis sets are available in the Supporting Information (SI). Due to the large amount of data, we will limit the discussion to results obtained with the aug-cc-pVTZ basis set, which generally provides a good balance between cost and accuracy at zero field and is therefore considered an attractive choice in most GTO calculations. This basis set was also used in our previous work on diatomic molecules in ref (47), and has been employed in studies by other authors as well in the literature.^7,11,32,38,81^

The AHGBSP3-9 basis set^61^ is the largest GTO basis considered in this work, and we use it to represent a feasible limit for GTO basis sets in the discussion. The comparison of the aug-cc-pVTZ and AHGBSP3-9 results then affords insights into the limitations of GTO basis sets in finite field calculations when GIAOs/LAOs are employed. As we believe AHGBSP3-9 to be close to the CBS limit for GTOs, an optimized GTO basis set for finite fields should be able to get close to the AHGBSP3-9 values with considerably fewer basis functions, showing a marked improvement on the aug-cc-pVTZ values, which are limited by the basis set designed for field-free calculations.

To aid the discussion on the real-orbital approximation of section 2.1, we will show differences in total energies in blue, if the energy difference between the FEM and AHGBSP3-9 calculations are positive at all studied magnetic field strengths, and in red if the difference is negative for at least one field strength; we note that the latter usually happens already at zero field. We will now discuss the results atom by atom.

### H

**Figure 1:**
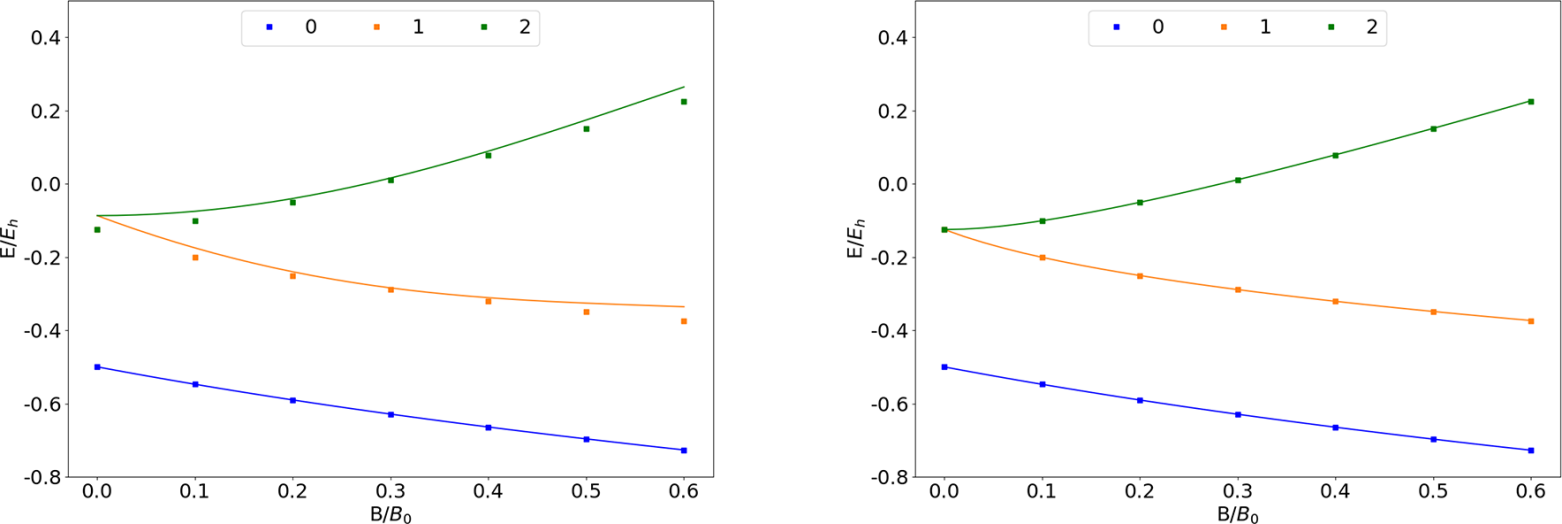
Total energy for various states of the H atom as a function of the magnetic field strength *B* in the aug-cc-pVTZ (left) and AHGBSP3-9 (right) basis sets compared to complex-orbital FEM calculations at the CBS limit (squares).

The energies of the low-lying states of the H atom are shown as a function of the field strength in Figure 1. The mean differences between the FEM and GTO energies are shown in Table 1. The 1σ state of H, which is also the ground state throughout the range of field strengths considered in this work, is qualitatively well described by both aug-cc-pVTZ and AHGBSP3-9, with the latter affording much lower MAEDs.

**Table 1:**
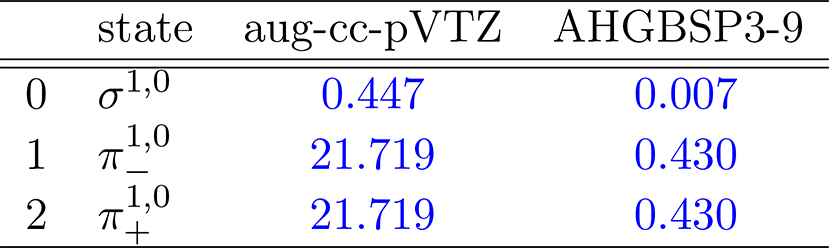
MAEDs between GTO and FEM Energies in m*E*_h_ for H in the Fully Uncontracted aug-cc-pVTZ and AHGBSP3-9 Basis Sets^a^

a For all tables in this work: differences in total energies are in blue if the energy difference between the FEM and AHGBSP3-9 calculations are positive at all studied magnetic field strengths, and in red if the difference is negative for at least one field strength.

The BSTEs for the Π states of H are significant in the aug-cc-pVTZ basis set. We also see from Figure 1 that the BSTE for the Π states in the aug-cc-pVTZ basis have a minimum around *B* = 0.3*B*_0_, which likely arises from fortuitous error cancellation. The energy differences for the Π states are negligible in the AHGBSP3-9 basis set.

We also note that even the aug-cc-pV5Z basis set exhibits significant differences for the Π states, which are visually discernible in the plots included in the SI, while the AHGBSP3-9 data appear spot-on. These results suggest that the description of the Π states for H could be significantly improved for finite field calculations in standard basis sets by adding more *p* and higher functions to improve the description of the π orbital.

### He

**Figure 2:**
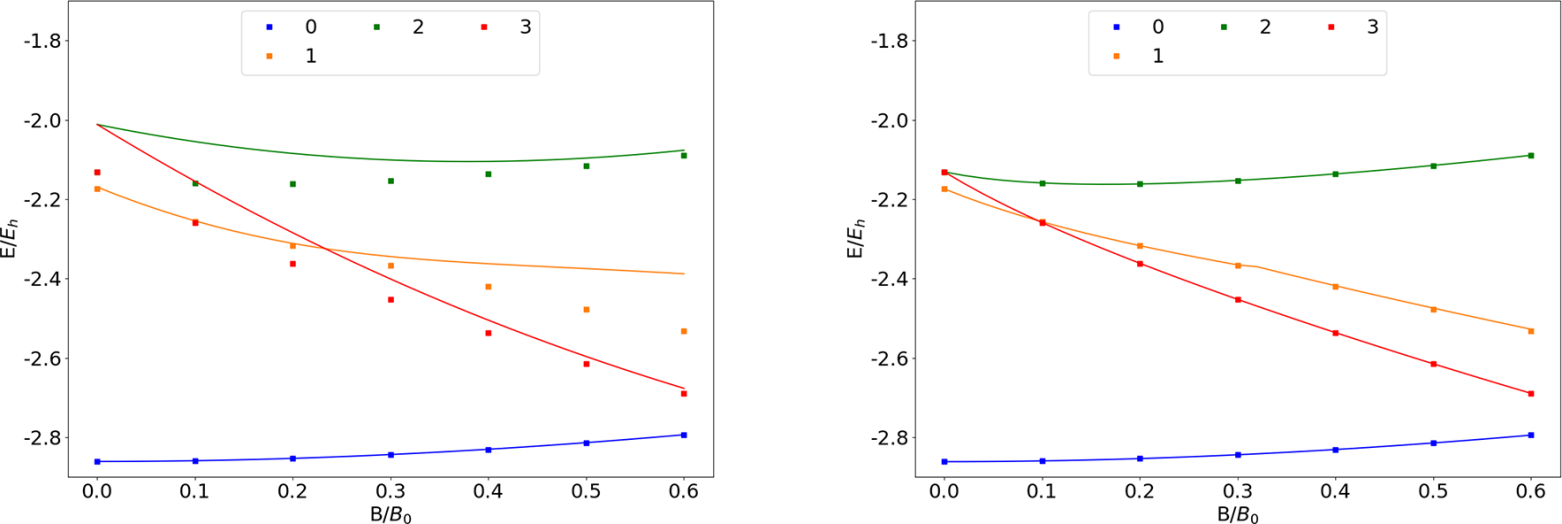
Total energy of the He atom as a function of the magnetic field strength *B* in the aug-cc-pVTZ (left) and AHGBSP3-9 (right) basis sets compared to complex-orbital FEM calculations at the CBS limit (squares).

The energies of the low-lying states of the He atom are shown as a function of the field strength in Figure 2. The mean differences between the FEM and GTO energies are shown in Table 2. Similarly to H, He does not exhibit ground state crossings in the observed range of field strengths. The 1σ^2^ ground state configuration is again qualitatively well described by both GTO basis sets, with AHGBSP3-9 affording much smaller errors.

**Table 2:**
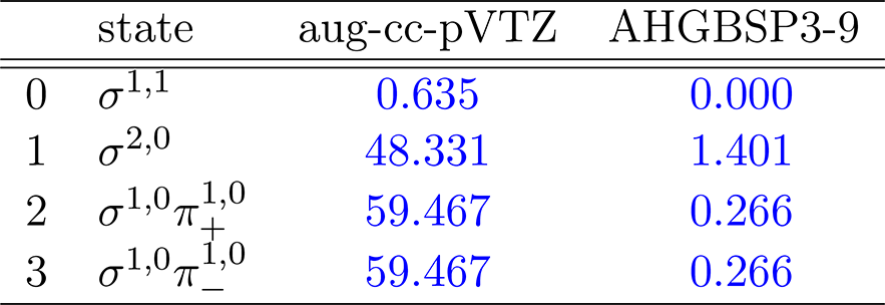
MAEDs between GTO and FEM Energies in m*E*_h_ for He in the Fully Uncontracted aug-cc-pVTZ and AHGBSP3-9 Basis Sets

The Π states exhibit large energy differences in the aug-cc-pVTZ basis set in the weak field regime, but the differences decrease at stronger fields, indicating that the spatial shape of the orbitals can be better described in the aug-cc-pVTZ basis set at the relatively stronger fields.

The AHGBSP3-9 basis set, in contrast, again affords much lower MAEDs for the Π states as well. This again indicates room to improve on the standard basis sets for finite-field calculations.

The lowering of the σ^2,0^ triplet state in increasing field strength is described extremely poorly by the aug-cc-pVTZ basis set but is better recovered by AHGBSP3-9. Also the aug-cc-pV5Z basis set gives poor results for the σ^2,0^ state, as shown by the data in the SI.

### Li

**Figure 3:**
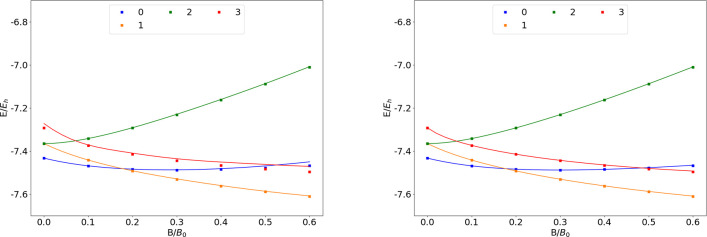
Total energy of the Li atom as a function of the magnetic field strength *B* in the aug-cc-pVTZ (left) and AHGBSP3-9 (right) basis sets compared to complex-orbital FEM calculations at the CBS limit (squares).

The energies of the low-lying states of the Li atom are shown as a function of the field strength in Figure 3. The mean differences between the FEM and GTO energies are listed in Table 3. We observe that Li is the first element to exhibit a ground state crossing at the studied field strengths: the UHF ground state configuration changes from σ^2,1^ to σ^1,1^π_–_^1,0^ around *B* ≈ 0.2*B*_0_.

**Table 3:**
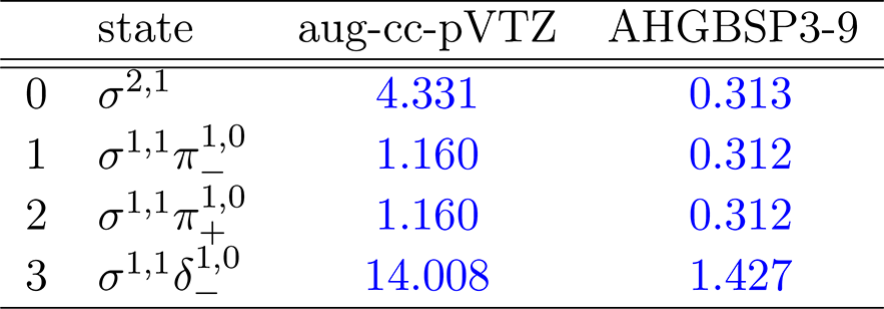
MAEDs between GTO and FEM Energies in m*E*_h_ for Li in the Fully Uncontracted aug-cc-pVTZ and AHGBSP3-9 Basis Sets

Interestingly, a large BSTE is observed for the σ^2,1^ state at larger fields for the aug-cc-pVTZ basis set, while the state is again much better described by AHGBSP3-9. However, the remaining mean energy difference for AHGBSP3-9 is still surprisingly large, even though the FEM and AHGBSP3-9 energies are in perfect agreement at zero field, as can be seen from the data in the SI. The growing difference in the total energy as a function of the magnetic field from 0 μ*E*_h_ to 1.38 m*E*_h_ at *B* = 0.6*B*_0_ for the σ^2,1^ state is explained by the weak binding of the outermost electron, which is thereby strongly affected by the magnetic field and undergoes a large deformation.

We also see that the Δ state with an occupied δ orbital is poorly described by the aug-cc-pVTZ basis set, while it is well recovered by the AHGBSP3-9 basis set.

### Be

**Figure 4:**
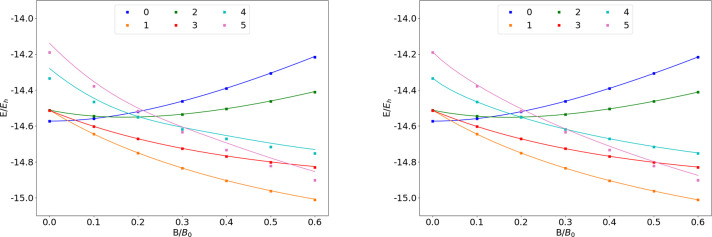
Total energy of the Be atom as a function of the magnetic field strength *B* in the aug-cc-pVTZ (left) and AHGBSP3-9 (right) basis sets compared to complex-orbital FEM calculations at the CBS limit (squares).

The energies of the low-lying states of the Be atom are shown as a function of the field strength in Figure 4. The mean differences between the FEM and GTO energies are shown in Table 4.

**Table 4:**
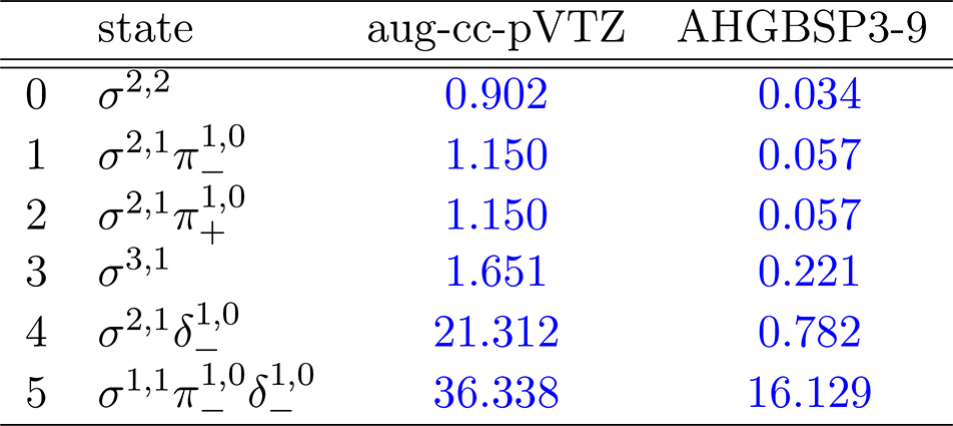
MAEDs between GTO and FEM Energies in m*E*_h_ for Be in the Fully Uncontracted aug-cc-pVTZ and AHGBSP3-9 Basis Sets

We see a ground state crossing between σ^2,2^ and σ^2,1^π_–_^1,0^ around *B* ≈ 0.05*B*_0_. All of the Σ and Π states are well described in both GTO basis sets. The σ^2,1^δ_–_^1,0^ state is ill-described in aug-cc-pVTZ, but it is well recovered by AHGBSP3-9, indicating that higher polarization functions can recover the state well.

However, the σ^1,1^π_–_^1,0^δ_–_^1,0^ state is ill-described even by AHGBSP3-9 with a MAED of over 10 m*E*_h_. This large difference likely arises mostly from the difference in the real-orbital approximation (section 2.1) used in the GTO calculations and the complex-orbital FEM calculations, instead of the incompleteness of the GTO basis set.

### B

**Figure 5:**
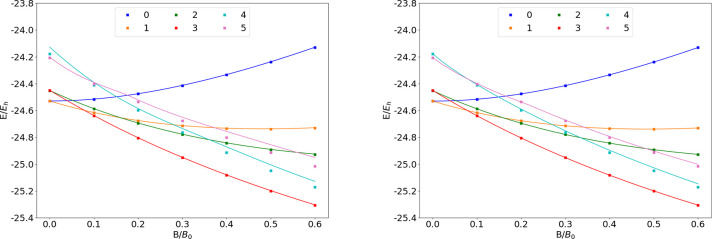
Total energy of the B atom as a function of magnetic field strength *B* in the aug-cc-pVTZ (left) and AHGBSP3-9 (right) basis sets compared to complex-orbital FEM calculations at the CBS limit (squares).

The energies of the low-lying states of the B atom are shown as a function of the field strength in Figure 5. The mean differences between the FEM and GTO energies are shown in Table 5.

**Table 5:**
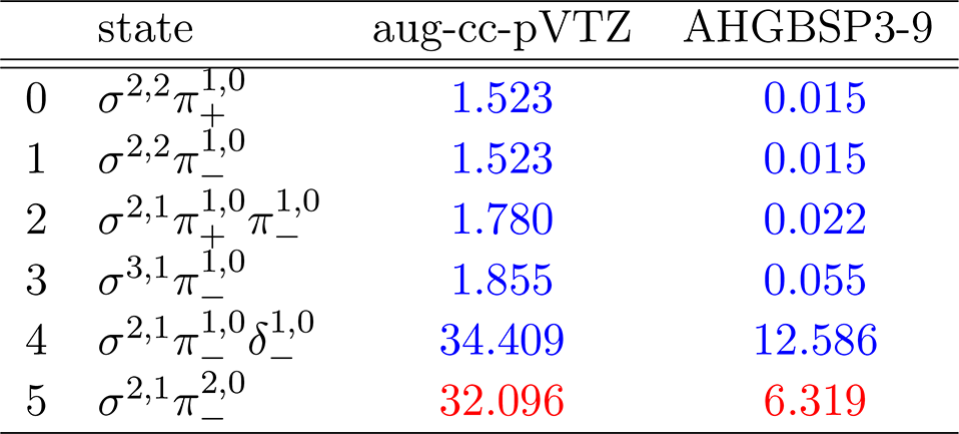
MAEDs between GTO and FEM Energies in m*E*_h_ for B in the Fully Uncontracted aug-cc-pVTZ and AHGBSP3-9 Basis Sets

There is a ground state crossing between σ^2,2^π_–_^1,0^ and σ^3,1^π_–_^1,0^ around *B* ≈ 0.075*B*_0_. All of the Π states are well described in both GTO basis sets, except the σ^2,1^π_–_^2,0^ state that has a large error in aug-cc-pVTZ of over 30 m*E*_h_, which is reduced considerably to 6 m*E*_h_ in the AHGBSP3-9 basis set. We note that this state is one of the states prone to saddle point convergence (see section 3).

The state with an occupied δ orbital also exhibits large MAEDs, which we again tentatively attribute to the use of the real-orbital approximation (section 2.1).

### C

**Figure 6:**
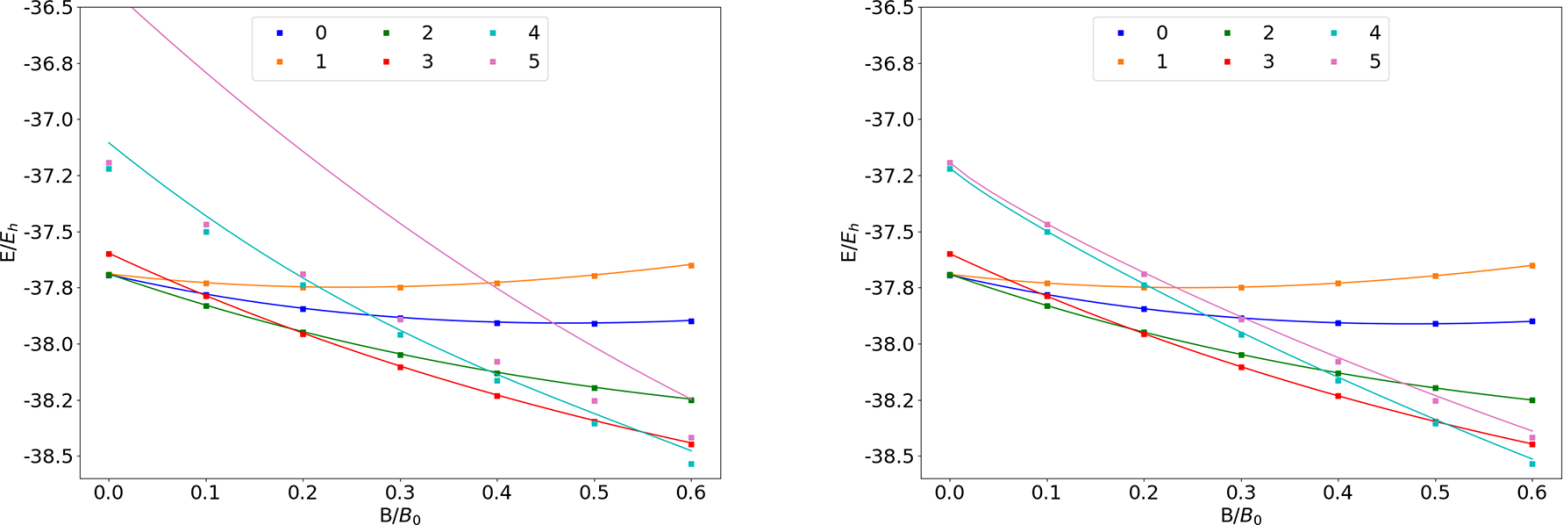
Total energy of the C atom as a function of magnetic field strength *B* in the aug-cc-pVTZ (left) and AHGBSP3-9 (right) basis sets compared to complex-orbital FEM calculations at the CBS limit (squares).

The energies of the low-lying states of the C atom are shown as a function of the field strength in Figure 6. The mean differences between the FEM and GTO energies are shown in Table 6.

**Table 6:**
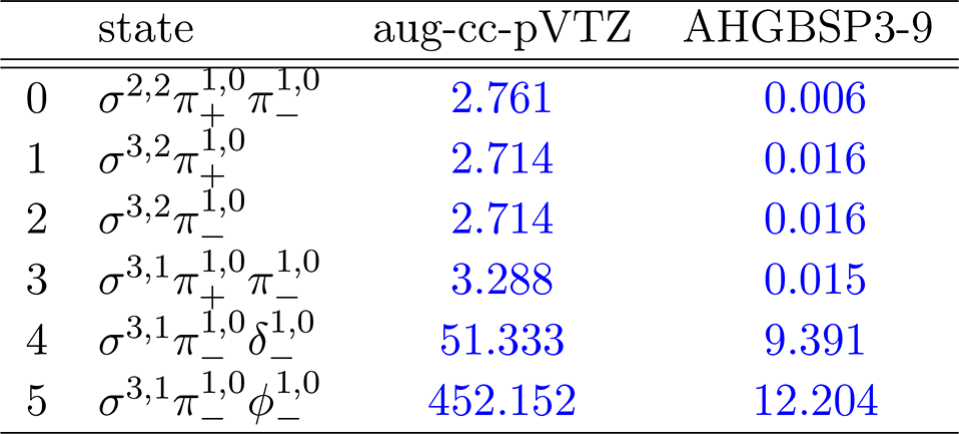
MAEDs between GTO and FEM Energies in m*E*_h_ for C in the Fully Uncontracted aug-cc-pVTZ and AHGBSP3-9 Basis Sets

C is the first element with more than one observed ground state crossing: we see a change from σ^3,2^π_–_^1,0^ to σ^3,1^π_+_^1,0^π_–_^1,0^ around *B* ≈ 0.2*B*_0_, and further to σ^3,1^π_–_^1,0^δ_–_^1,0^ around *B* ≈ 0.5*B*_0_.

All of the low lying Π states are reasonably well described by the aug-cc-pVTZ basis. However, the energy differences are 2 orders of magnitude smaller in the AHGBSP3-9 basis set.

We see that the state with an occupied δ orbital is ill-described by the aug-cc-pVTZ basis set, that it is better described by the AHGBSP3-9 basis set, and that the remaining MAED would likely be much smaller without the use of the real-orbital approximation in the GTO calculations.

The state with the occupied φ orbital exhibits very large energy differences in aug-cc-pVTZ. The state is drastically better described on the AHGBSP3-9 basis, reducing the mean difference by hundreds of millihartrees from the aug-cc-pVTZ value. However, a negative energy difference is observed for this state at zero field with the AHGBSP3-9 basis set, which can arise only from the use of the real-orbital approximation of section 2.1.

### N

**Figure 7:**
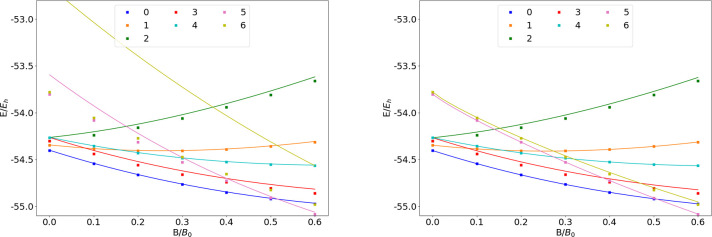
Total energy of the N atom as a function of the magnetic field strength *B* in the aug-cc-pVTZ (left) and AHGBSP3-9 (right) basis sets compared to complex-orbital FEM calculations at the CBS limit (squares).

The energies of the low-lying states of the N atom are shown as a function of the field strength in Figure 7. The mean differences between the FEM and GTO energies are listed in Table 7.

**Table 7:**
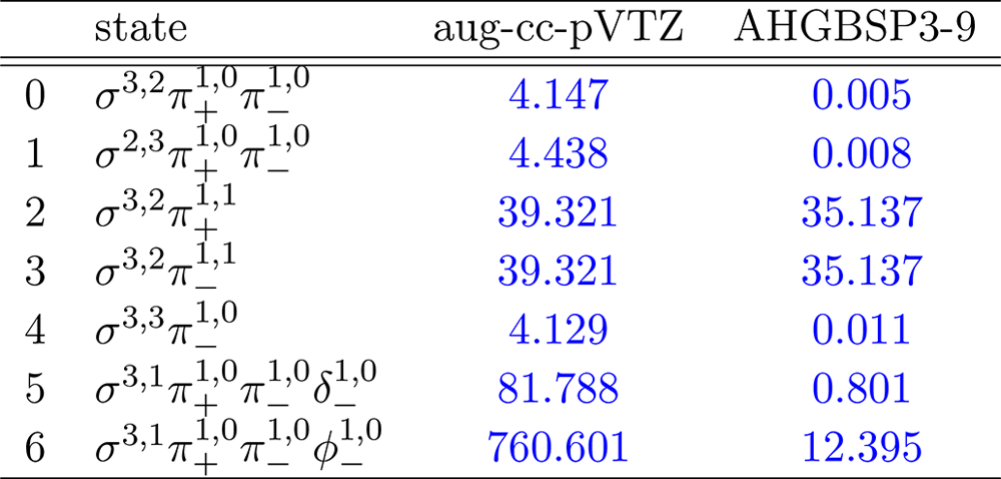
MAEDs between GTO and FEM Energies in m*E*_h_ for N in the Fully Uncontracted aug-cc-pVTZ and AHGBSP3-9 Basis Sets

N only has one observed ground state crossing: around *B* ≈ 0.5*B*_0_ the ground state changes from σ^3,2^π_+_^1,0^π_–_^1,0^ to σ^3,1^π_+_^1,0^π_–_^1,0^δ_–_^1,0^.

The Π states σ^3,2^π_±_^1,1^ are poorly described by both GTO basis sets, with mean differences of 39.32 m*E*_h_ and 35.14 m*E*_h_, respectively; this is likely again an artifact of the real-orbital approximation used for the GTO calculations. The other Π states are well described by both of the GTO basis sets.

The large MAED for the state with the occupied δ orbital arises mainly at the weak field regime in the aug-cc-pVTZ basis set; the state is much better recovered at stronger fields. The small MAED of AHGBSP3-9 indicates that this state can be recovered by GTO expansion.

Similarly to the case of carbon discussed above, also here the description of the low-lying state with an occupied φ orbital is drastically improved by the AHGBSP3-9 basis, even though a negative energy difference arising from the real-orbital approximation is again observed.

### O

**Figure 8:**
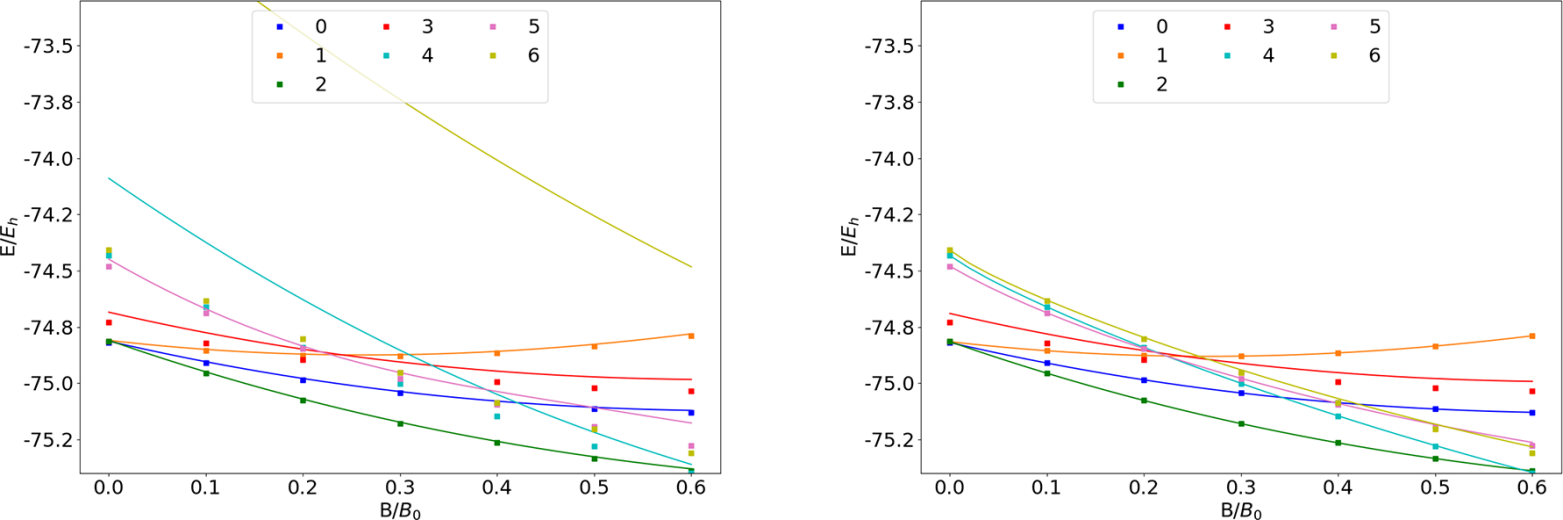
Total energy of the O atom as a function of the magnetic field strength *B* in the aug-cc-pVTZ (left) and AHGBSP3-9 (right) basis sets compared to complex-orbital FEM calculations at the CBS limit (squares).

The energies of the low-lying states of the O atom are shown as a function of the field strength in Figure 8. The mean differences between the FEM and GTO energies are shown in Table 8.

**Table 8:**
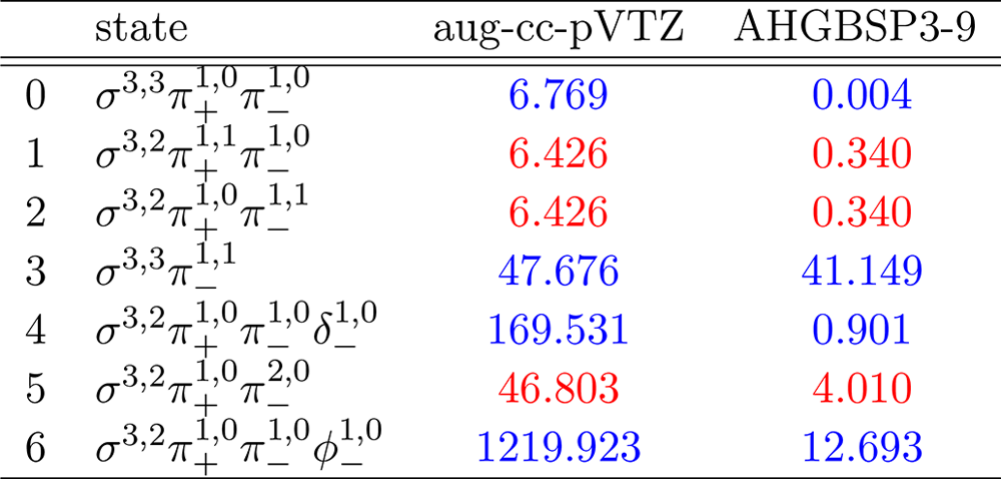
MAEDs between GTO and FEM Energies in m*E*_h_ for O in the Fully Uncontracted aug-cc-pVTZ and AHGBSP3-9 Basis Sets

We observe a ground state crossing around *B* ≈ 0.6*B*_0_ from σ^3,2^π_+_^1,0^π_–_^1,1^ to σ^3,2^π_+_^1,0^π_–_^1,0^δ_–_^1,0^. The Π states σ^3,3^π_+_^1,0^π_–_^1,0^, σ^3,2^π_+_^1,1^π_–_^1,0^, and σ^3,2^π_+_^1,0^π_–_^1,1^ are reasonably well described in aug-cc-pVTZ. However, we still see a significant improvement going to the AHGBSP3-9 basis, while aug-cc-pV5Z exhibits similarly large errors to aug-cc-pVTZ.

The σ^3,3^π_–_^1,1^ state exhibits large errors of similar magnitude in both GTO basis sets, again suggesting that this state is not captured by the real-orbital approximation of section 2.1.

The state with the occupied δ orbital has a very large MAED in aug-cc-pVTZ, and even though the difference becomes smaller in increasing field strength, it remains significant at *B* = 0.6*B*_0_. The description of the state is drastically better in AHGBSP3-9.

The σ^3,2^π_+_^1,0^π_–_^2,0^ state and the state with an occupied φ orbital again show drastic improvement going from aug-cc-pVTZ to the AHGBSP3-9 basis set, indicating room to improve upon standard GTO basis sets at finite magnetic fields.

### F

**Figure 9:**
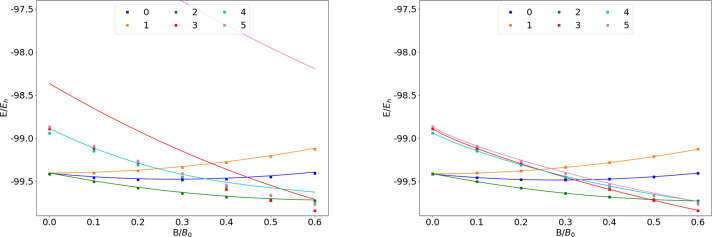
Total energy of the F atom as a function of the magnetic field strength *B* in the aug-cc-pVTZ (left) and AHGBSP3-9 (right) basis sets compared to complex-orbital FEM calculations at the CBS limit (squares).

The energies of the low-lying states of the F atom are shown as a function of the field strength in Figure 9. The mean differences between the FEM and GTO energies are listed in Table 9.

**Table 9:**
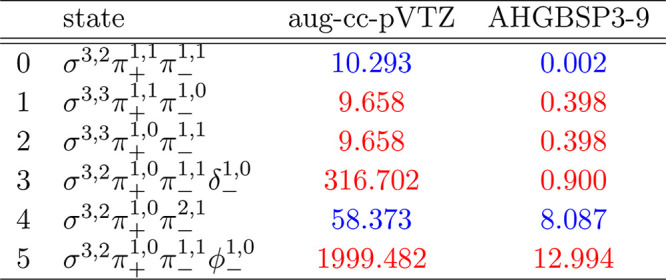
MAEDs between GTO and FEM Energies in m*E*_h_ for F in the Fully Uncontracted aug-cc-pVTZ and AHGBSP3-9 Basis Sets

We observe a ground state change from σ^3,3^π_+_^1,0^π_–_^1,1^ to σ^3,2^π_+_^1,0^π_–_^1,1^δ_–_^1,0^ around *B* ≈ 0.5*B*_0_. Similarly to the case of oxygen discussed above, the states σ^3,2^π_+_^1,1^π_–_^1,1^, σ^3,3^π_+_^1,1^π_–_^1,0^, and σ^3,3^π_+_^1,0^π_–_^1,1^ are relatively well described by aug-cc-pVTZ, but their errors are orders of magnitude smaller in the AHGBSP3-9 basis set.

The MAED of the state with the occupied δ orbital decreases in increasing field strength in aug-cc-pVTZ, but the difference remains significant at *B* = 0.6*B*_0_; AHGBSP3-9 affords a MAED for this state that is over 2 orders of magnitude smaller.

The σ^3,2^π_+_^1,0^π_–_^2,1^ state is likewise ill-described in the aug-cc-pVTZ basis but is better described by AHGBSP3-9.

We again notice that AHGBSP3-9 offers a drastic improvement in accuracy for the state with an occupied φ orbital that is very poorly described even in the aug-cc-pV5Z basis.

### Ne

**Figure 10:**
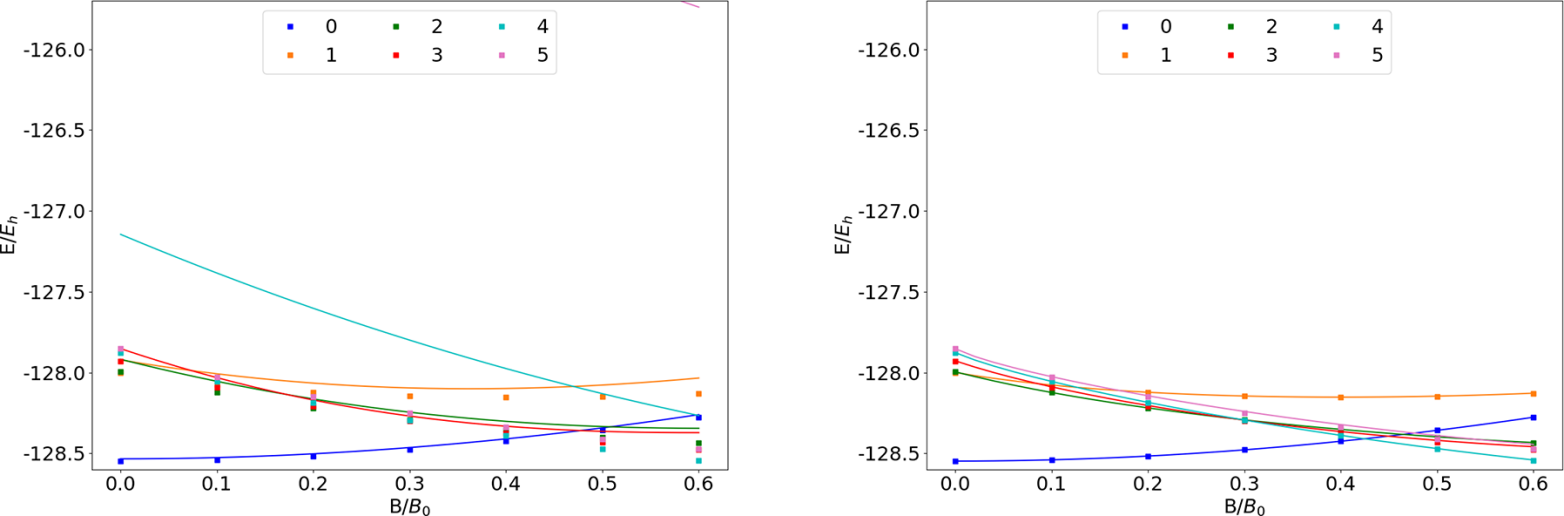
Total energy of the Ne atom as a function of the magnetic field strength *B* in the aug-cc-pVTZ (left) and AHGBSP3-9 (right) basis sets compared to complex-orbital FEM calculations at the CBS limit (squares).

The energies of the low-lying states of the Ne atom are shown as a function of the field strength in Figure 10. The mean differences between the FEM and GTO energies are shown in Table 10.

**Table 10:**
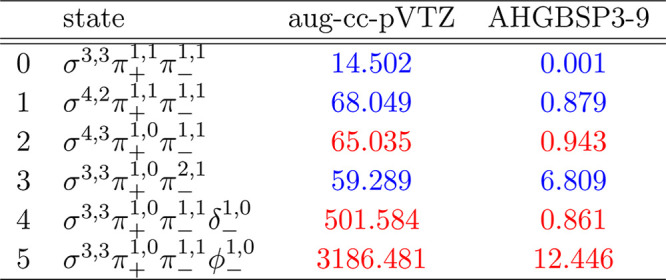
MAEDs between GTO and FEM Energies in m*E*_h_ for Ne in the Fully Uncontracted aug-cc-pVTZ and AHGBSP3-9 Basis Sets

Ne has one ground state crossing: around *B* ≈ 0.42*B*_0_ we observe a change from σ^3,3^π_+_^1,1^π_–_^1,1^ to σ^3,3^π_+_^1,0^π_–_^1,1^δ_–_^1,0^. We observe that only σ^3,3^π_+_^1,1^π_–_^1,1^ is reasonably well described by the aug-cc-pVTZ basis and that the MAED is 5 orders of magnitude smaller in the AHGBSP3-9 basis set.

The Π states σ^4,2^π_+_^1,1^π_–_^1,1^ and σ^4,3^π_+_^1,0^π_–_^1,1^ are poorly described by aug-cc-pVTZ, but they are well recovered by AHGBSP3-9. The σ^3,3^π_+_^1,0^π_–_^2,1^ state is poorly described by aug-cc-pVTZ and recovered well by AHGBSP3-9 as well.

Similarly to several cases discussed above, the MAED for the state with an occupied δ orbital is large in the aug-cc-pVTZ basis, and AHGBSP3-9 again offers drastic improvement. Likewise, the description of the state with an occupied φ orbital is drastically improved by AHGBSP3-9, suggesting room to improve standard basis sets.

### Na

**Figure 11:**
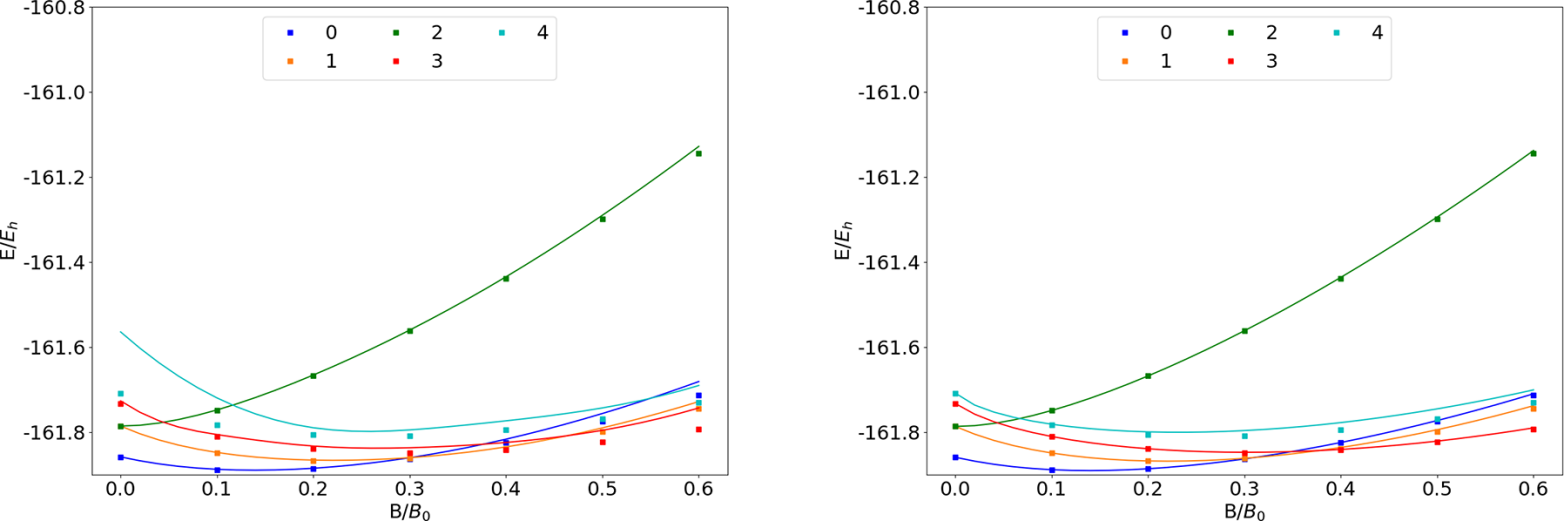
Total energy of the Na atom as a function of magnetic field strength *B* in the aug-cc-pVTZ (left) and AHGBSP3-9 (right) basis sets compared to complex-orbital FEM calculations at the CBS limit (squares).

The energies of the low-lying states of the Na atom are shown as a function of the field strength in Figure 11. The mean differences between the FEM and GTO energies are listed in Table 11.

**Table 11:**
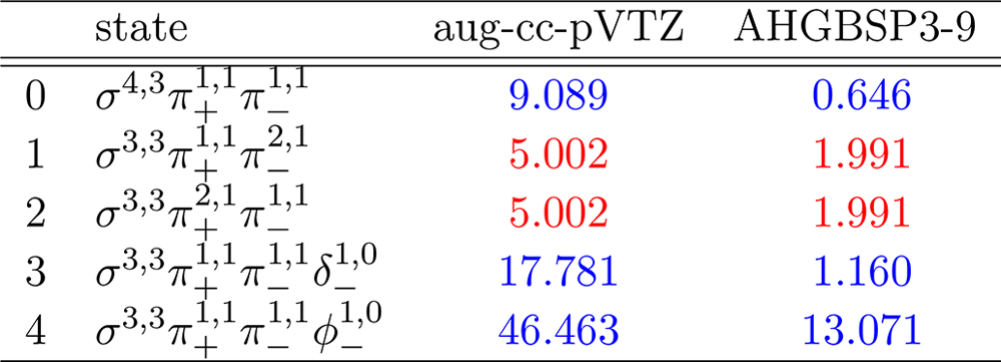
MAEDs between GTO and FEM Energies in m*E*_h_ for Na in the Fully Uncontracted aug-cc-pVTZ and AHGBSP3-9 Basis Sets

We observe two ground state crossings: the ground state changes briefly from σ^4,3^π_+_^1,1^π_–_^1,1^ to σ^3,3^π_+_^1,1^π_–_^2,1^ around *B* ≈ 0.3*B*_0_, before changing again to σ^3,3^π_+_^1,1^π_–_^1,1^δ_–_^1,0^ around *B* ≈ 0.4*B*_0_. All of these Π states are quite well described by aug-cc-pVTZ, with the AHGBSP3-9 basis set exhibiting strongly reduced MAEDs.

The states with occupied δ or φ orbitals again have significant MAEDs in the aug-cc-pVTZ basis, while the corresponding MAEDs are orders of magnitude smaller in the AHGBSP3-9 basis set.

### Mg

**Figure 12:**
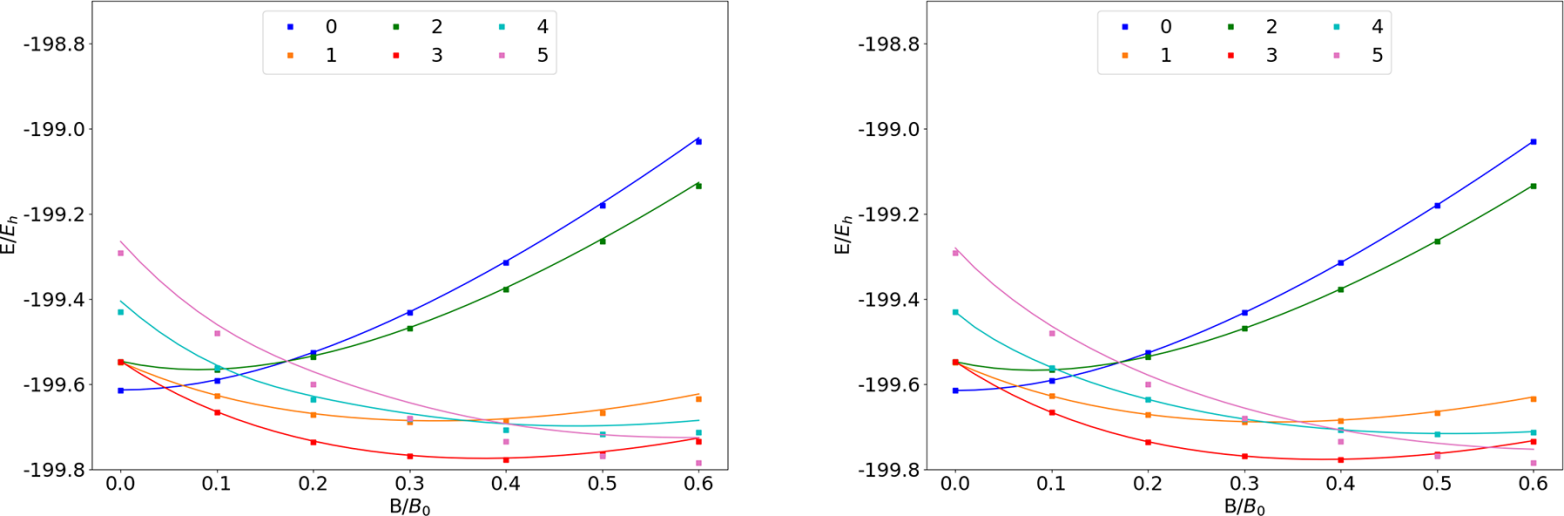
Total energy of the Mg atom as a function of the magnetic field strength *B* in the aug-cc-pVTZ (left) and AHGBSP3-9 (right) basis sets compared to complex-orbital FEM calculations at the CBS limit (squares).

The energies of the low-lying states of the Mg atom are shown as a function of the field strength in Figure 12. The mean differences between the FEM and GTO energies are listed in Table 12.

**Table 12:**
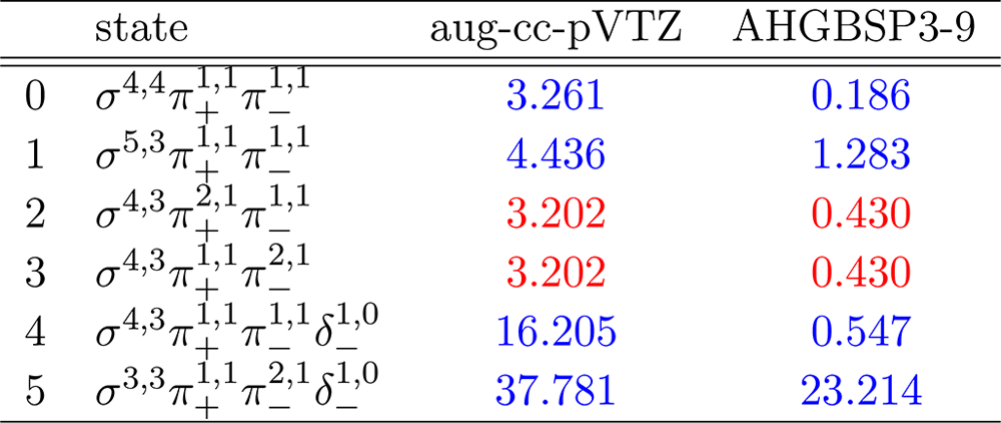
MAEDs between GTO and FEM Energies in m*E*_h_ for Mg in the Fully Uncontracted aug-cc-pVTZ and AHGBSP3-9 Basis Sets

We see the ground state changing from σ^4,4^π_+_^1,1^π_–_^1,1^ to σ^4,3^π_+_^1,1^π_–_^2,1^ around *B* ≈ 0.05*B*_0_. The ground state changes again to σ^3,3^π_+_^1,1^π_–_^2,1^δ_–_^1,0^ around *B* ≈ 0.5*B*_0_.

The states with occupied σ and π orbitals are well described by the aug-cc-pVTZ basis. The σ^4,3^π_+_^1,1^π_–_^1,1^δ_–_^1,0^ state has an error of over 10 m*E*_h_ in aug-cc-pVTZ, which is reduced by almost a factor of 30 in the AHGBSP3-9 basis set. The σ^3,3^π_+_^1,1^π_–_^2,1^δ_–_^1,0^ state still shows a large MAED, which likely arises from the use of the real-orbital approximation in section 2.1.

### Al

**Figure 13:**
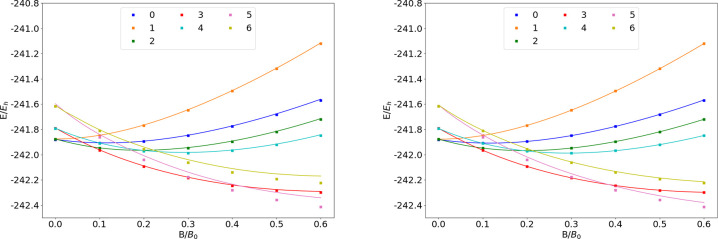
Total energy of the Al atom as a function of the magnetic field strength *B* in the aug-cc-pVTZ (left) and AHGBSP3-9 (right) basis sets compared to complex-orbital FEM calculations at the CBS limit (squares).

The energies of the low-lying states of the Al atom are shown as a function of the field strength in Figure 13. The mean differences between the FEM and GTO energies are shown in Table 13.

**Table 13:**
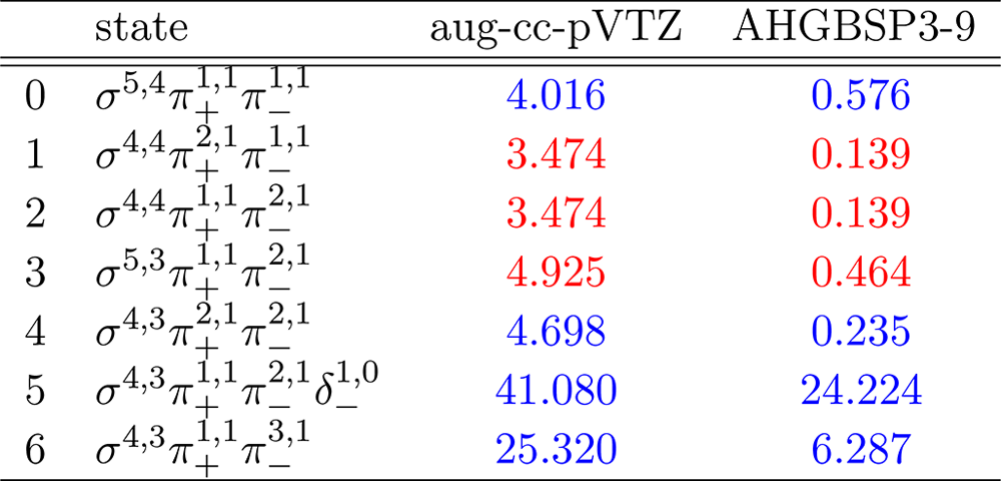
MAEDs between GTO and FEM Energies in m*E*_h_ for Al in the Fully Uncontracted aug-cc-pVTZ and AHGBSP3-9 Basis Sets

Al changes ground state twice: from the zero-field configuration σ^4,4^π_+_^1,1^π_–_^2,1^ to σ^5,3^π_+_^1,1^π_–_^2,1^ around *B* ≈ 0.08*B*_0_, and then again to σ^4,3^π_+_^1,1^π_–_^2,1^δ_–_^1,0^ around *B* ≈ 0.35*B*_0_.

The states with occupied σ and π orbitals are adequately described by the aug-cc-pVTZ basis set, although the AHGBSP3-9 basis set affords considerably smaller MAEDs. The only exception is the σ^4,3^π_+_^1,1^π_–_^3,1^ state, which exhibits large MAEDs, which can likely be attributed to the use of the real-orbital approximation of section 2.1.

The MAED for the state with the occupied δ orbital decreases by almost 60% in going from aug-cc-pVTZ to the AHGBSP3-9 basis set. The large remaining MAED is again likely attributable to the real-orbital approximation.

### Si

**Figure 14:**
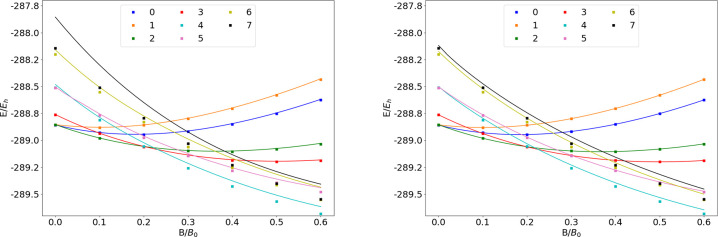
Total energy of the Si atom as a function of magnetic field strength *B* in the aug-cc-pVTZ (left) and AHGBSP3-9 (right) basis sets compared to complex-orbital FEM calculations at the CBS limit (squares).

The energies of the low-lying states of the Si atom are shown as a function of the field strength in Figure 14. The mean differences between the FEM and GTO energies are shown in Table 14.

**Table 14:**
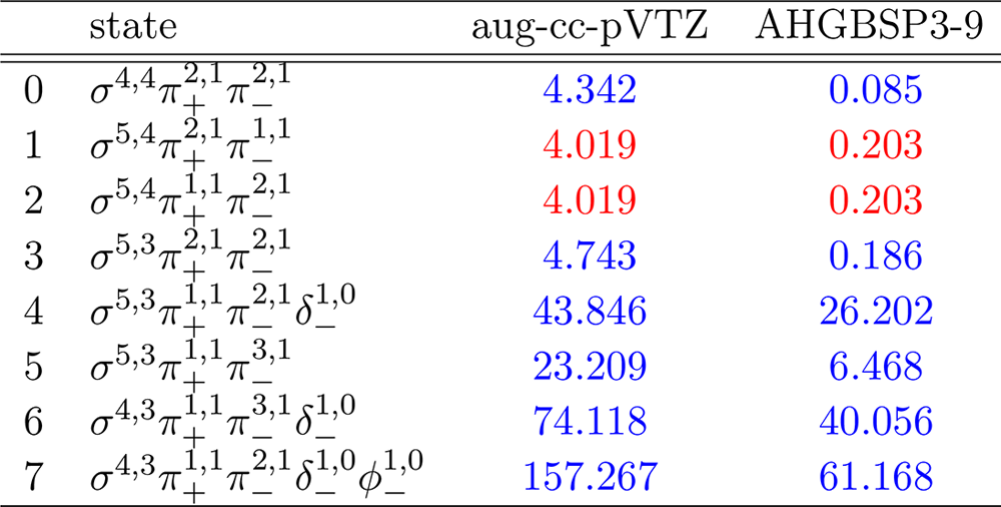
MAEDs between GTO and FEM Energies in m*E*_h_ for Si in the Fully Uncontracted aug-cc-pVTZ and AHGBSP3-9 Basis Sets

Si has a ground state crossing from σ^5,4^π_+_^1,1^π_–_^2,1^ to σ^5,3^π_+_^1,1^π_–_^2,1^δ_–_^1,0^ around *B* ≈ 0.2*B*_0_.

The states with occupied σ and π orbitals appear to be adequately described in the aug-cc-pVTZ basis set with MAEDs around 4 m*E*_h_. The exception is the σ^4,3^π_+_^1,1^π_–_^3,1^ state that has a MAED of over 20 m*E*_h_, while the AHGBSP3-9 basis affords a smaller MAED that is likely dominated by artifacts from the real-orbital approximation. The state is decently described also by the aug-cc-pV5Z basis set.

The states with occupied δ and φ orbitals are all badly described in the aug-cc-pVTZ basis set. The energy differences are still significant in the AHGBSP3-9 basis set, indicating artifacts from the real-orbital approximation, even though the improvement in the MAEDs over aug-cc-pVTZ is clear.

### P

**Figure 15:**
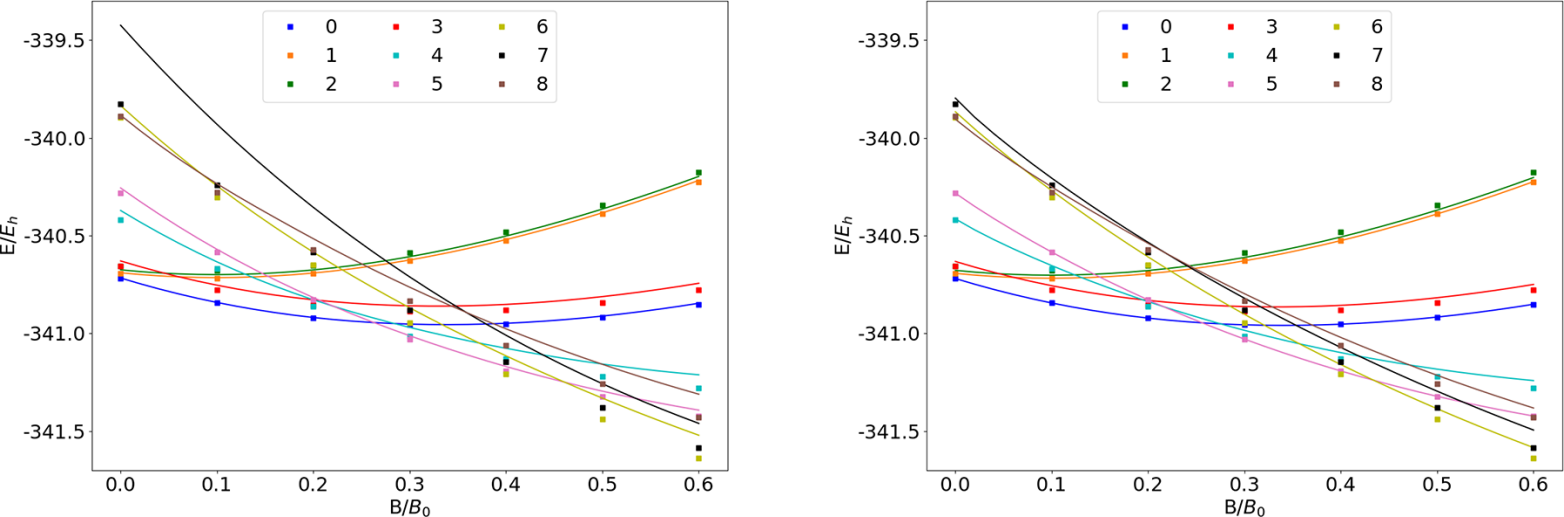
Total energy of the P atom as a function of the magnetic field strength *B* in the aug-cc-pVTZ (left) and AHGBSP3-9 (right) basis sets compared to complex-orbital FEM calculations at the CBS limit (squares).

The energies of the low-lying states of the P atom are shown as a function of the field strength in Figure 15. The mean differences between the FEM and GTO energies are shown in Table 15.

**Table 15:**
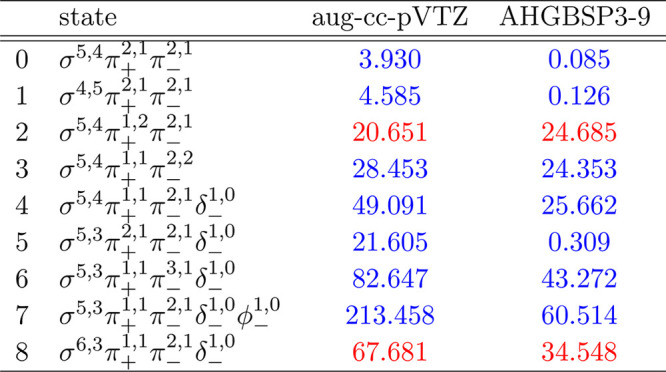
MAEDs between GTO and FEM Energies in m*E*_h_ for P in the Fully Uncontracted aug-cc-pVTZ and AHGBSP3-9 Basis Sets

P exhibits two ground state crossings: from σ^5,4^π_+_^2,1^π_–_^2,1^ to σ^5,3^π_+_^2,1^π_–_^2,1^δ_–_^1,0^ around *B* ≈ 0.25*B*_0_ and then again to σ^5,3^π_+_^1,1^π_–_^3,1^δ_–_^1,0^ around *B* ≈ 0.4*B*_0_.

The only states well described by aug-cc-pVTZ are σ^5,4^π_+_^2,1^π_–_^2,1^ and σ^4,5^π_+_^2,1^π_–_^2,1^. The significant differences observed for the σ^5,4^π_+_^1,2^π_–_^2,1^ and σ^5,4^π_+_^1,1^π_–_^2,2^ states are of similar magnitude for both GTO basis sets and likely arise from the real-orbital approximation of section 2.1.

The improvement for all states with occupied δ and φ orbitals when going from aug-cc-pVTZ to AHGBSP3-9 is clear. However, only the σ^5,3^π_+_^2,1^π_–_^2,1^δ_–_^1,0^ state has a small MAED in AHGBSP3-9, the MAEDs for the other states likely arising from the real-orbital approximation used in the GTO calculations.

### S

**Figure 16:**
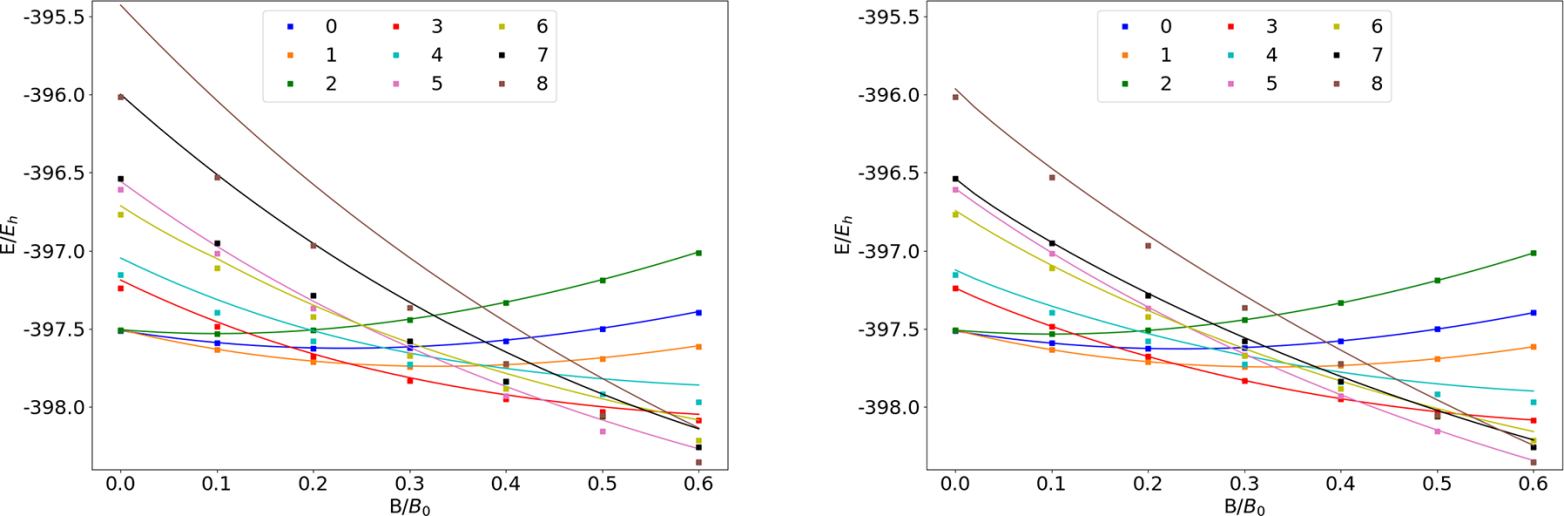
Total energy of the S atom as a function of magnetic field strength *B* in the aug-cc-pVTZ (left) and AHGBSP3-9 (right) basis sets compared to complex-orbital FEM calculations at the CBS limit (squares).

The energies of the low-lying states of the S atom are shown as a function of the field strength in Figure 16. The mean differences between the FEM and GTO energies are shown in Table 16.

**Table 16:**
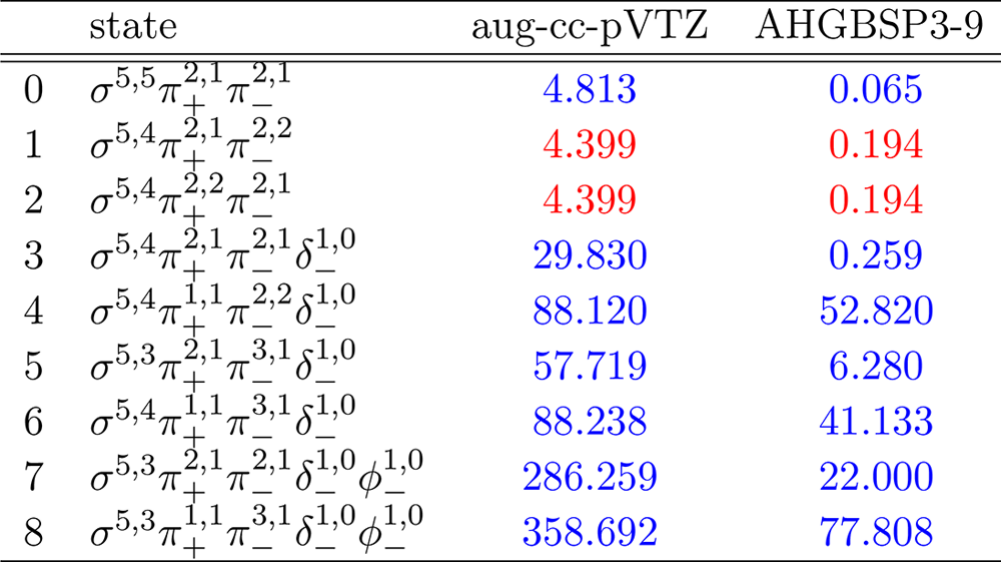
MAEDs between GTO and FEM Energies in m*E*_h_ for S in the Fully Uncontracted aug-cc-pVTZ and AHGBSP3-9 Basis Sets

S is the only studied element to have three ground state crossings in the considered range of magnetic field strengths: the ground state first changes from σ^5,4^π_+_^2,1^π_–_^2,2^ to σ^5,4^π_+_^2,1^π_–_^2,1^δ_–_^1,0^ around *B* ≈ 0.225*B*_0_, then to σ^5,3^π_+_^2,1^π_–_^3,1^δ_–_^1,0^ around *B* ≈ 0.4*B*_0_, and finally to σ^5,3^π_+_^1,1^π_–_^3,1^δ_–_^1,0^ϕ_–_^1,0^ around *B* ≈ 0.6*B*_0_.

All of the states with occupied σ and π orbitals are again well described by both GTO basis sets. The σ^5,4^π_+_^2,1^π_–_^2,1^δ_–_^1,0^ and σ^5,3^π_+_^2,1^π_–_^3,1^δ_–_^1,0^ states are ill-described by aug-cc-pVTZ but well recovered by AHGBSP3-9, suggesting room to improve on standard basis sets.

The σ^5,4^π_+_^1,1^π_–_^2,2^δ_–_^1,0^ and σ^5,4^π_+_^1,1^π_–_^3,1^δ_–_^1,0^ states show considerable improvement going from aug-cc-pVTZ to AHGBSP3-9, even though these remaining MAEDs are still significant and likely caused by the real-orbital approximation.

The improvement for the states with occupied φ orbitals is drastic when going from aug-cc-pVTZ to AHGBSP3-9, again indicating room to improve on standard basis sets, even though the remaining MAEDs are large, which is likely an artifact of the real-orbital approximation.

### Cl

**Figure 17:**
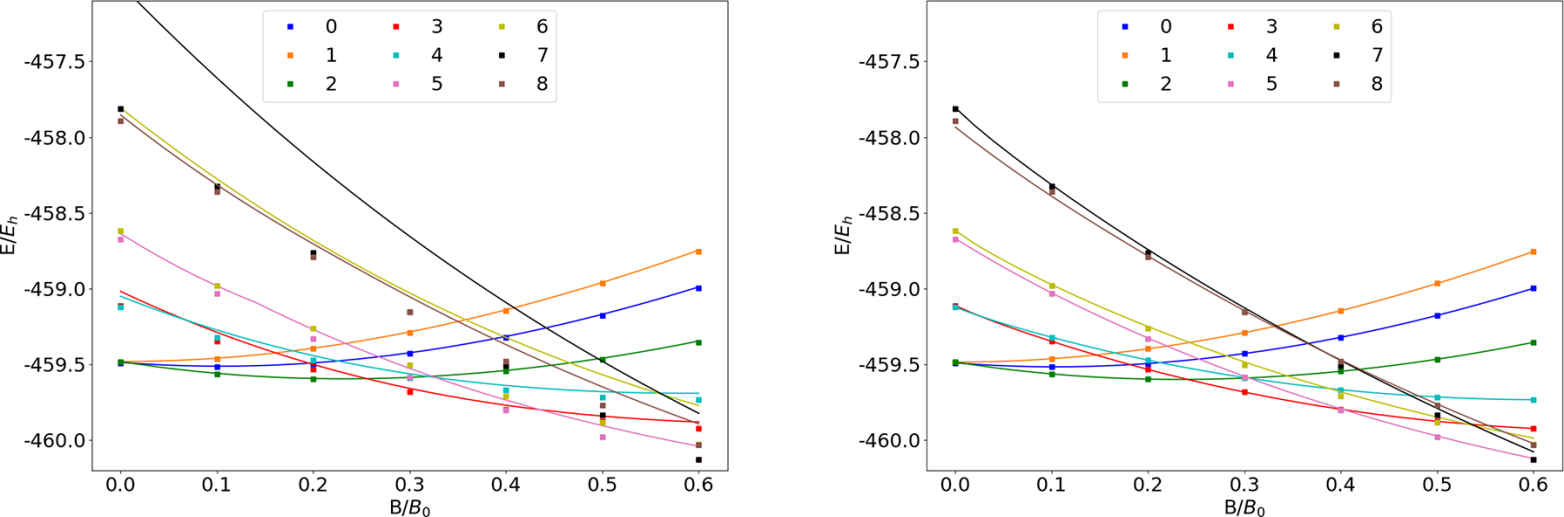
Total energy of the Cl atom as a function of the magnetic field strength *B* in the aug-cc-pVTZ (left) and AHGBSP3-9 (right) basis sets compared to complex-orbital FEM calculations at the CBS limit (squares).

The energies of the low-lying states of the Cl atom are shown as a function of the field strength in Figure 17. The mean differences between the FEM and GTO energies are listed in Table 17.

**Table 17:**
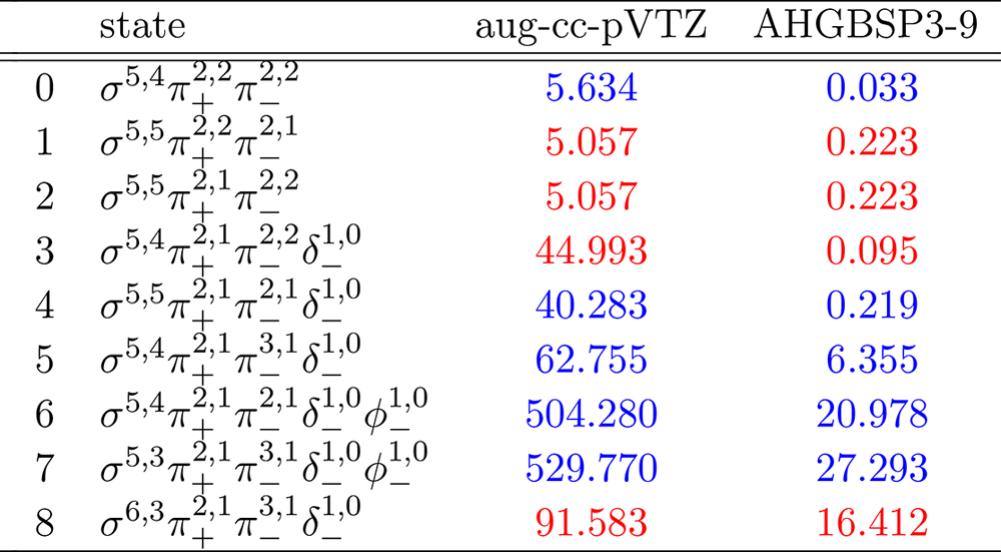
MAEDs between GTO and FEM Energies in m*E*_h_ for Cl in the Fully Uncontracted aug-cc-pVTZ and AHGBSP3-9 Basis Sets

Cl exhibits two ground state crossings: from the field-free ground state configuration σ^5,5^π_+_^2,1^π_–_^2,2^ to σ^5,4^π_+_^2,1^π_–_^2,2^δ_–_^1,0^ around *B* ≈ 0.25*B*_0_, and then to σ^5,4^π_+_^2,1^π_–_^3,1^δ_–_^1,0^ around *B* ≈ 0.4*B*_0_. We again see that all of the states with occupied σ and π orbitals are well described by both GTO basis sets.

We likewise again observe that the description of the states with occupied δ and φ orbitals can be significantly improved by going from aug-cc-pVTZ to AHGBSP3-9, showing room to improve standard basis sets for finite field calculations.

### Ar

**Figure 18:**
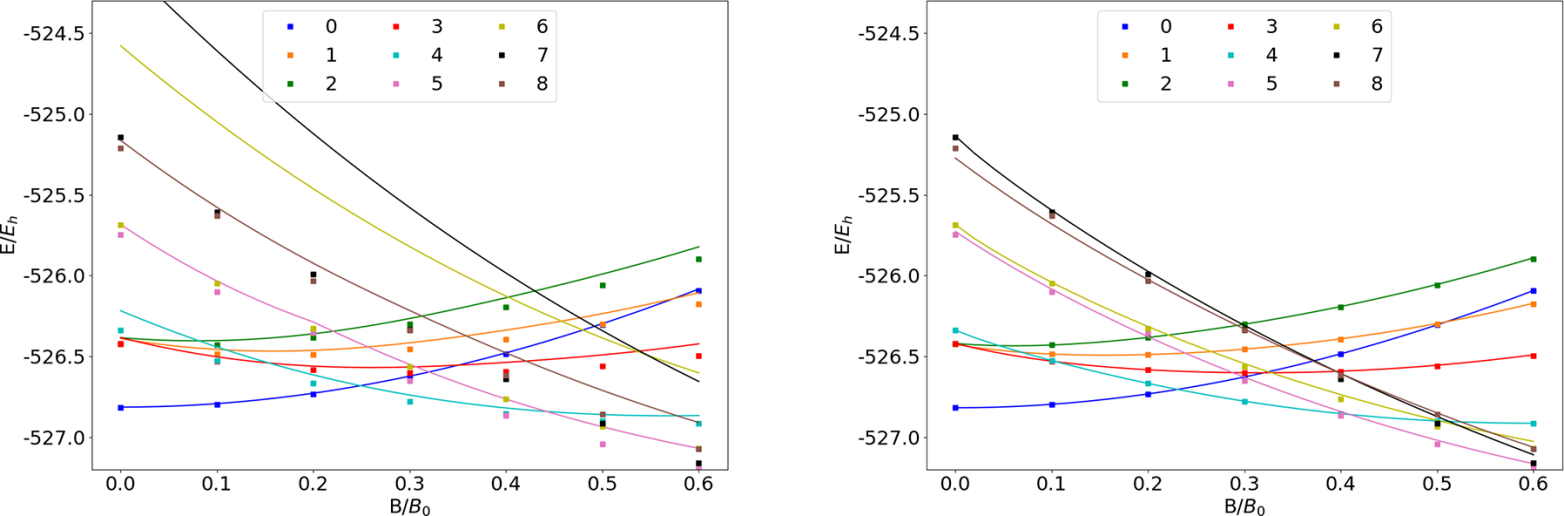
Total energy of the Ar atom as a function of magnetic field strength *B* in the aug-cc-pVTZ (left) and AHGBSP3-9 (right) basis sets compared to complex-orbital FEM calculations at the CBS limit (squares).

The energies of the low-lying states of the Ar atom are shown as a function of the field strength in Figure 18. The mean differences between the FEM and GTO energies are shown in Table 18.

**Table 18:**
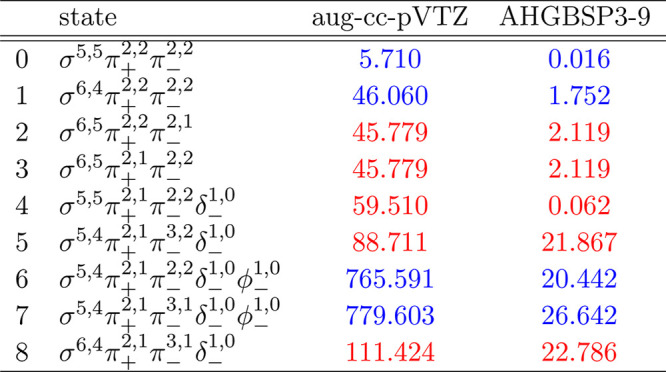
MAEDs between GTO and FEM Energies in m*E*_h_ for Ar in the Fully Uncontracted aug-cc-pVTZ and AHGBSP3-9 Basis Sets

Ar has two ground state crossings: from σ^5,5^π_+_^2,2^π_–_^2,2^ to σ^5,5^π_+_^2,1^π_–_^2,2^δ_–_^1,0^ around *B* ≈ 0.225*B*_0_, and then again to σ^5,4^π_+_^2,1^π_–_^3,2^δ_–_^1,0^ around *B* ≈ 0.4*B*_0_.

Only the σ^5,5^π_+_^2,2^π_–_^2,2^ state is well described by aug-cc-pVTZ. σ^6,4^π_+_^2,2^π_–_^2,2^ exhibits a large MAED in aug-cc-pVTZ, which is significantly reduced in AHGBSP3-9; the other states with occupied σ and π orbitals exhibit similar behavior.

The MAED of the σ^5,5^π_+_^2,1^π_–_^2,2^δ_–_^1,0^ state is large in aug-cc-pVTZ, and small in AHGBSP3-9. The other states with occupied δ orbitals also see drastic improvements in going to AHGBSP3-9, proving that these states can be described significantly better by improved basis sets.

The states with occupied φ orbitals are extremely badly described in aug-cc-pVTZ, and they are much better described in AHGBSP3-9 whose MAEDs are likely dominated by the real-orbital approximation.

## 5 Summary and Conclusions

We have determined complete basis set (CBS) limit energies of the low lying states of H–Ar in magnetic fields of *B* ∈ [0, 0.6*B*_0_] at the unrestricted Hartree–Fock (UHF) level of theory with fully numerical calculations with the finite element method (FEM), employing complex wave functions.

We have also suggested a real-orbital approximation for calculations with Gaussian-type orbital (GTO) basis sets, which we employed to carry out calculations in a large variety of GTO basis sets.

We have computed energy differences between the GTO basis set and FEM calculations to identify atomic states that are poorly described by the standard GTO basis sets optimized for zero field and indicated several states that could be described significantly more accurately with GTO basis sets optimized for finite field calculations with London atomic orbitals (LAOs), also known as gauge-including atomic orbitals (GIAOs).

In general, we observe that states with high ⟨−*L̂*_*z*_⟩ that become important at stronger magnetic fields due to the orbital Zeeman term are poorly described in the aug-cc-pVTZ basis set. We notice that the benchmark quality AHGBSP3-9 basis can often recover these states to high accuracy.

Larger errors are also encountered by higher spin states, which similarly couple to the magnetic field by the spin Zeeman term and which become the ground state at stronger fields. These larger errors are likely caused by the differences in the spatial form of the orbitals: the higher spin state has more electrons of the same spin, which have to obey Pauli’s exclusion principle. This results in a difference in the spatial form that is not taken into account in standard basis sets optimized at zero field. Also these states are well described by the benchmark quality AHGBSP3-9 basis set.

Some states appear to be ill-described even by the very large AHGBSP3-9 basis set. We believe these discrepancies to stem from the real-orbital approximation employed in this work, which was described in section 2.1. Even when large mean absolute energy differences (MAED) are observed for the AHGBSP3-9 basis set, we do observe significant reductions of MAED from the aug-cc-pVTZ basis set.

The use of complex GTOs could be visited in later work as they will enable apples-to-apples studies of the MAED, affording direct access into the basis set truncation error (BSTE). The physical Hamiltonian could also be recovered with the use of real spherical harmonics by employing a complex Hamiltonian matrix. Although such complex wave functions may be supported in Erkale in the future, the results of this work are already sufficient to serve as a basis for developing improved Gaussian basis sets for calculations at finite magnetic fields: our results indicate that basis sets tailored for calculations at finite magnetic fields can be constructed with the approximate method employed in this work.

## Supporting Information Available

Plots demonstrating the convergence of the numerical basis sets to the CBS limit, and tables of the numerical CBS limit energies; mean absolute energy differences for each state of each atom in each of the studied basis sets; plots of the total energies of each state of each atom as a function of the magnetic field strength in each of the studied basis sets; tables of total energies for all states of all atoms over the studied range of magnetic field strengths (PDF)
