# Supplementary material for: Insight on Gaussian Basis Set Truncation Errors in Weak to Intermediate Magnetic Fields with an Approximate Hamiltonian
Source: J Phys Chem A. 2023 Dec 16;127(51):10872–88. doi: 10.1021/acs.jpca.3c04531 (PMC10758122; doi:10.1021/acs.jpca.3c04531)
Supplement: Supplementary file 1 — jp3c04531_si_001.pdf [file jp3c04531_si_001.pdf]

Supporting Information: Insight on Gaussian Basis Set Truncation Errors in Weak to Intermediate Magnetic Fields with an Approximate Hamiltonian

Hugo Åström and Susi Lehtola*

University of Helsinki, Department of Chemistry, Faculty of Science, P.O. Box 55 (A.I. Virtanens plats 1), FI-00014 University of Helsinki, Finland

E-mail: susi.lehtola@alumni.helsinki.fi

1 Convergence of Total Energies in FEM to the CBS Limit

We begin by showing that the complete basis set (CBS) limit is achieved with the employed finite element method (FEM) by plotting the convergence of the total energy at the field strength $B = 0.60$ as a function of the angular truncation parameter $l_{\max}$. The energy difference $\Delta E = E(l_{\max}) - E(l_{\max} - 2)$ is shown in fig. S1 for H, in fig. S2 for He, in fig. S3 for Li, in fig. S4 for Be, in fig. S5 for B, in fig. S6 for C, in fig. S7 for N, in fig. S8 for O, in fig. S9 for F, in fig. S10 for Ne, in fig. S11 for Na, in fig. S12 for Mg, in fig. S13 for Al, in fig. S14 for Si, in fig. S15 for P, in fig. S16 for S, in fig. S17 for Cl, and in fig. S18 for Ar.

The resulting CBS total energies of all atoms, determined with FEM, are given in in table S1 for H, in table S2 for He, in table S3 for Li, in table S4 for Be, in table S5 for B, in table S6 for C, in table S7 for N, in table S8 for O, in table S9 for F, in table S10 for Ne, in table S11 for Na, in table S12 for Mg, in table S13 for Al, in table S14 for Si, in table S15 for P, in table S16 for S, in table S17 for Cl, and in table S18 for Ar.

2 Difference of GTO Energies to FEM Values

2.1 Mean Absolute Differences

Given these total energies, the differences $\Delta E^{\mathrm{GTO}} = E^{\mathrm{GTO}} - E^{\mathrm{CBS}}$ at each field strength for each state are obtained. First, the average absolute energy differences for the studied Gaussian basis sets in the fully uncontracted form are given in in table S19 for H, in table S20 for He, in table S21 for Li, in table S22 for Be, in table S23 for B, in table S24 for C, in table S25 for N, in table S26 for O, in table S27 for F, in table S28 for Ne, in table S29 for Na, in table S30 for Mg, in table S31 for Al, in table S32 for Si, in table S33 for P, in table S34 for S, in table S35 for Cl, and in table S36 for Ar.

Missing entries in the tables indicate either that the basis set for the given element does not exist on the Basis Set Exchange, e.g. aug-cc-pV5Z for Li in table S21, or that the basis set is too small to describe the state in question, e.g. the state of the C atom with the occupied φ orbital in table S24 which requires at least f functions in the atomic basis.

2.2 Plots of FEM and GTO Total Energies

Next, we include plots of the differences for all the studied states of all the studied atoms in all the studied basis sets as a function of the field strength B.

The results for the H atom are given in in fig. S19 for cc-pVDZ, in fig. S20 for cc-pVTZ, in fig. S21 for cc-pVQZ, in fig. S22 for cc-pV5Z, in fig. S23 for aug-cc-pVDZ, in fig. S24 for aug-cc-pVTZ, in fig. S25 for aug-cc-pVQZ, in fig. S26 for aug-cc-pV5Z, in fig. S27 for HGBSP1-5, in fig. S28 for HGBSP1-7, in fig. S29 for HGBSP1-9, in fig. S30 for HGBSP2-5, in fig. S31 for HGBSP2-7, in fig. S32 for HGBSP2-9, in fig. S33 for HGBSP3-5, in fig. S34 for HGBSP3-7, in fig. S35 for HGBSP3-9, in fig. S36 for AHGBSP1-5, in fig. S37 for AHGBSP1-7, in fig. S38 for AHGBSP1-9, in fig. S39 for AHGBSP2-5, in fig. S40 for AHGBSP2-7, in fig. S41 for AHGBSP2-9, in fig. S42 for AHGBSP3-5, in fig. S43 for AHGBSP3-7, in fig. S44 for AHGBSP3-9, in fig. S45 for 6-311++G(3df3pd), and in fig. S46 for def2-TZVP, all basis sets being employed in the fully uncontracted form.

The results for the He atom are given in in fig. S47 for cc-pVDZ, in fig. S48 for cc-pVTZ, in fig. S49 for cc-pVQZ, in fig. S50 for cc-pV5Z, in fig. S51 for aug-cc-pVDZ, in fig. S52 for aug-cc-pVTZ, in fig. S53 for aug-cc-pVQZ, in fig. S54 for aug-cc-pV5Z, in fig. S55 for HGBSP1-5, in fig. S56 for HGBSP1-7, in fig. S57 for HGBSP1-9, in fig. S58 for HGBSP2-5, in fig. S59 for HGBSP2-7, in fig. S60 for HGBSP2-9, in fig. S61 for HGBSP3-5, in fig. S62 for HGBSP3-7, in fig. S63 for HGBSP3-9, in fig. S64 for AHGBSP1-5, in fig. S65 for AHGBSP1-7, in fig. S66 for AHGBSP1-9, in fig. S67 for AHGBSP2-5, in fig. S68 for AHGBSP2-7, in fig. S69 for AHGBSP2-9, in fig. S70 for AHGBSP3-5, in fig. S71 for AHGBSP3-7, in fig. S72 for AHGBSP3-9, in fig. S73 for 6-311++G(3df3pd), and in fig. S74 for def2-TZVP, all basis sets being employed in the fully uncontracted form.

The results for the Li atom are given in in fig. S75 for cc-pVDZ, in fig. S76 for cc-pVTZ, in fig. S77 for cc-pVQZ, in fig. S78 for cc-pV5Z, in fig. S79 for aug-cc-pVDZ, in fig. S80 for aug-cc-pVTZ, in fig. S81 for aug-cc-pVQZ, in fig. S82 for aug-cc-pV5Z, in fig. S83 for HGBSP1-5, in fig. S84 for HGBSP1-7, in fig. S85 for HGBSP1-9, in fig. S86 for HGBSP2-5, in fig. S87 for HGBSP2-7, in fig. S88 for HGBSP2-9, in fig. S89 for HGBSP3-5, in fig. S90 for HGBSP3-7, in fig. S91 for HGBSP3-9, in fig. S92 for AHGBSP1-5, in fig. S93 for AHGBSP1-7, in fig. S94 for AHGBSP1-9, in fig. S95 for AHGBSP2-5, in fig. S96 for AHGBSP2-7, in fig. S97 for AHGBSP2-9, in fig. S98 for AHGBSP3-5, in fig. S99 for AHGBSP3-7, in fig. S100 for AHGBSP3-9, in fig. S101 for 6-311++G(3df3pd), and in fig. S102 for def2-TZVP, all basis sets being employed in the fully uncontracted form.

The results for the Be atom are given in in fig. S103 for cc-pVDZ, in fig. S104 for cc-pVTZ, in fig. S105 for cc-pVQZ, in fig. S106 for cc-pV5Z, in fig. S107 for aug-cc-pVDZ, in fig. S108 for aug-cc-pVTZ, in fig. S109 for aug-cc-pVQZ, in fig. S110 for aug-cc-pV5Z, in fig. S111 for HGBSP1-5, in fig. S112 for HGBSP1-7, in fig. S113 for HGBSP1-9, in fig. S114 for HGBSP2-5, in fig. S115 for HGBSP2-7, in fig. S116 for HGBSP2-9, in fig. S117 for HGBSP3-5, in fig. S118 for HGBSP3-7, in fig. S119 for HGBSP3-9, in fig. S120 for AHGBSP1-5, in fig. S121 for AHGBSP1-7, in fig. S122 for AHGBSP1-9, in fig. S123 for AHGBSP2-5, in fig. S124 for AHGBSP2-7, in fig. S125 for AHGBSP2-9, in fig. S126 for AHGBSP3-5, in fig. S127 for AHGBSP3-7, in fig. S128 for AHGBSP3-9, in fig. S129 for 6-311++G(3df3pd), and in fig. S130 for def2-TZVP, all basis sets being employed in the fully uncontracted form.

The results for the B atom are given in in fig. S131 for cc-pVDZ, in fig. S132 for cc-pVTZ, in fig. S133 for cc-pVQZ, in fig. S134 for cc-pV5Z, in fig. S135 for aug-cc-pVDZ, in fig. S136 for aug-cc-pVTZ, in fig. S137 for aug-cc-pVQZ, in fig. S138 for aug-cc-pV5Z, in fig. S139 for HGBSP1-5, in fig. S140 for HGBSP1-7, in fig. S141 for HGBSP1-9, in fig. S142 for HGBSP2-5, in fig. S143 for HGBSP2-7, in fig. S144 for HGBSP2-9, in fig. S145

for HGBSP3-5, in fig. S146 for HGBSP3-7, in fig. S147 for HGBSP3-9, in fig. S148 for AHGBSP1-5, in fig. S149 for AHGBSP1-7, in fig. S150 for AHGBSP1-9, in fig. S151 for AHGBSP2-5, in fig. S152 for AHGBSP2-7, in fig. S153 for AHGBSP2-9, in fig. S154 for AHGBSP3-5, in fig. S155 for AHGBSP3-7, in fig. S156 for AHGBSP3-9, in fig. S157 for 6-311++G(3df3pd), and in fig. S158 for def2-TZVP, all basis sets being employed in the fully uncontracted form.

The results for the C atom are given in in fig. S159 for cc-pVDZ, in fig. S160 for cc-pVTZ, in fig. S161 for cc-pVQZ, in fig. S162 for cc-pV5Z, in fig. S163 for aug-cc-pVDZ, in fig. S164 for aug-cc-pVTZ, in fig. S165 for aug-cc-pVQZ, in fig. S166 for aug-cc-pV5Z, in fig. S167 for HGBSP1-5, in fig. S168 for HGBSP1-7, in fig. S169 for HGBSP1-9, in fig. S170 for HGBSP2-5, in fig. S171 for HGBSP2-7, in fig. S172 for HGBSP2-9, in fig. S173 for HGBSP3-5, in fig. S174 for HGBSP3-7, in fig. S175 for HGBSP3-9, in fig. S176 for AHGBSP1-5, in fig. S177 for AHGBSP1-7, in fig. S178 for AHGBSP1-9, in fig. S179 for AHGBSP2-5, in fig. S180 for AHGBSP2-7, in fig. S181 for AHGBSP2-9, in fig. S182 for AHGBSP3-5, in fig. S183 for AHGBSP3-7, in fig. S184 for AHGBSP3-9, in fig. S185 for 6-311++G(3df3pd), and in fig. S186 for def2-TZVP, all basis sets being employed in the fully uncontracted form.

The results for the N atom are given in in fig. S187 for cc-pVDZ, in fig. S188 for cc-pVTZ, in fig. S189 for cc-pVQZ, in fig. S190 for cc-pV5Z, in fig. S191 for aug-cc-pVDZ, in fig. S192 for aug-cc-pVTZ, in fig. S193 for aug-cc-pVQZ, in fig. S194 for aug-cc-pV5Z, in fig. S195 for HGBSP1-5, in fig. S196 for HGBSP1-7, in fig. S197 for HGBSP1-9, in fig. S198 for HGBSP2-5, in fig. S199 for HGBSP2-7, in fig. S200 for HGBSP2-9, in fig. S201 for HGBSP3-5, in fig. S202 for HGBSP3-7, in fig. S203 for HGBSP3-9, in fig. S204 for AHGBSP1-5, in fig. S205 for AHGBSP1-7, in fig. S206 for AHGBSP1-9, in fig. S207 for AHGBSP2-5, in fig. S208 for AHGBSP2-7, in fig. S209 for AHGBSP2-9, in fig. S210 for AHGBSP3-5, in fig. S211 for AHGBSP3-7, in fig. S212 for AHGBSP3-9, in fig. S213 for 6-311++G(3df3pd), and in fig. S214 for def2-TZVP, all basis sets being employed in the fully uncontracted form.

The results for the O atom are given in in fig. S215 for cc-pVDZ, in fig. S216 for cc-pVTZ, in fig. S217 for cc-pVQZ, in fig. S218 for cc-pV5Z, in fig. S219 for aug-cc-pVDZ, in fig. S220 for aug-cc-pVTZ, in fig. S221 for aug-cc-pVQZ, in fig. S222 for aug-cc-pV5Z, in fig. S223 for HGBSP1-5, in fig. S224 for HGBSP1-7, in fig. S225 for HGBSP1-9, in fig. S226 for HGBSP2-5, in fig. S227 for HGBSP2-7, in fig. S228 for HGBSP2-9, in fig. S229 for HGBSP3-5, in fig. S230 for HGBSP3-7, in fig. S231 for HGBSP3-9, in fig. S232 for AHGBSP1-5, in fig. S233 for AHGBSP1-7, in fig. S234 for AHGBSP1-9, in fig. S235 for AHGBSP2-5, in fig. S236 for AHGBSP2-7, in fig. S237 for AHGBSP2-9, in fig. S238 for AHGBSP3-5, in fig. S239 for AHGBSP3-7, in fig. S240 for AHGBSP3-9, in fig. S241 for 6-311++G(3df3pd), and in fig. S242 for def2-TZVP, all basis sets being employed in the fully uncontracted form.

The results for the F atom are given in in fig. S243 for cc-pVDZ, in fig. S244 for cc-pVTZ, in fig. S245 for cc-pVQZ, in fig. S246 for cc-pV5Z, in fig. S247 for aug-cc-pVDZ, in fig. S248 for aug-cc-pVTZ, in fig. S249 for aug-cc-pVQZ, in fig. S250 for aug-cc-pV5Z, in fig. S251 for HGBSP1-5, in fig. S252 for HGBSP1-7, in fig. S253 for HGBSP1-9, in fig. S254 for HGBSP2-5, in fig. S255 for HGBSP2-7, in fig. S256 for HGBSP2-9, in fig. S257 for HGBSP3-5, in fig. S258 for HGBSP3-7, in fig. S259 for HGBSP3-9, in fig. S260 for AHGBSP1-5, in fig. S261 for AHGBSP1-7, in fig. S262 for AHGBSP1-9, in fig. S263 for AHGBSP2-5, in fig. S264 for AHGBSP2-7, in fig. S265 for AHGBSP2-9, in fig. S266 for AHGBSP3-5, in fig. S267 for AHGBSP3-7, in fig. S268 for AHGBSP3-9, in fig. S269 for 6-311++G(3df3pd), and in fig. S270 for def2-TZVP, all basis sets being employed in the fully uncontracted form.

The results for the Ne atom are given in in fig. S271 for cc-pVDZ, in fig. S272 for cc-pVTZ, in fig. S273 for cc-pVQZ, in fig. S274 for cc-

pV5Z, in fig. S275 for aug-cc-pVDZ, in fig. S276 for aug-cc-pVTZ, in fig. S277 for aug-cc-pVQZ, in fig. S278 for aug-cc-pV5Z, in fig. S279 for HGBSP1-5, in fig. S280 for HGBSP1-7, in fig. S281 for HGBSP1-9, in fig. S282 for HGBSP2-5, in fig. S283 for HGBSP2-7, in fig. S284 for HGBSP2-9, in fig. S285 for HGBSP3-5, in fig. S286 for HGBSP3-7, in fig. S287 for HGBSP3-9, in fig. S288 for AHGBSP1-5, in fig. S289 for AHGBSP1-7, in fig. S290 for AHGBSP1-9, in fig. S291 for AHGBSP2-5, in fig. S292 for AHGBSP2-7, in fig. S293 for AHGBSP2-9, in fig. S294 for AHGBSP3-5, in fig. S295 for AHGBSP3-7, in fig. S296 for AHGBSP3-9, in fig. S297 for 6-311++G(3df3pd), and in fig. S298 for def2-TZVP, all basis sets being employed in the fully uncontracted form.

The results for the Na atom are given in in fig. S299 for cc-pVDZ, in fig. S300 for cc-pVTZ, in fig. S301 for cc-pVQZ, in fig. S302 for cc-pV5Z, in fig. S303 for aug-cc-pVDZ, in fig. S304 for aug-cc-pVTZ, in fig. S305 for aug-cc-pVQZ, in fig. S306 for aug-cc-pV5Z, in fig. S307 for HGBSP1-5, in fig. S308 for HGBSP1-7, in fig. S309 for HGBSP1-9, in fig. S310 for HGBSP2-5, in fig. S311 for HGBSP2-7, in fig. S312 for HGBSP2-9, in fig. S313 for HGBSP3-5, in fig. S314 for HGBSP3-7, in fig. S315 for HGBSP3-9, in fig. S316 for AHGBSP1-5, in fig. S317 for AHGBSP1-7, in fig. S318 for AHGBSP1-9, in fig. S319 for AHGBSP2-5, in fig. S320 for AHGBSP2-7, in fig. S321 for AHGBSP2-9, in fig. S322 for AHGBSP3-5, in fig. S323 for AHGBSP3-7, in fig. S324 for AHGBSP3-9, in fig. S325 for 6-311++G(3df3pd), and in fig. S326 for def2-TZVP, all basis sets being employed in the fully uncontracted form.

The results for the Mg atom are given in in fig. S327 for cc-pVDZ, in fig. S328 for cc-pVTZ, in fig. S329 for cc-pVQZ, in fig. S330 for cc-pV5Z, in fig. S331 for aug-cc-pVDZ, in fig. S332 for aug-cc-pVTZ, in fig. S333 for aug-cc-pVQZ, in fig. S334 for aug-cc-pV5Z, in fig. S335 for HGBSP1-5, in fig. S336 for HGBSP1-7, in fig. S337 for HGBSP1-9, in fig. S338 for HGBSP2-5, in fig. S339 for HGBSP2-7, in fig. S340 for HGBSP2-9, in fig. S341 for HGBSP3-5, in fig. S342 for HGBSP3-7, in fig. S343 for HGBSP3-9, in fig. S344 for AHGBSP1-5, in fig. S345 for AHGBSP1-7, in fig. S346 for AHGBSP1-9, in fig. S347 for AHGBSP2-5, in fig. S348 for AHGBSP2-7, in fig. S349 for AHGBSP2-9, in fig. S350 for AHGBSP3-5, in fig. S351 for AHGBSP3-7, in fig. S352 for AHGBSP3-9, in fig. S353 for 6-311++G(3df3pd), and in fig. S354 for def2-TZVP, all basis sets being employed in the fully uncontracted form.

The results for the Al atom are given in in fig. S355 for cc-pVDZ, in fig. S356 for cc-pVTZ, in fig. S357 for cc-pVQZ, in fig. S358 for cc-pV5Z, in fig. S359 for aug-cc-pVDZ, in fig. S360 for aug-cc-pVTZ, in fig. S361 for aug-cc-pVQZ, in fig. S362 for aug-cc-pV5Z, in fig. S363 for HGBSP1-5, in fig. S364 for HGBSP1-7, in fig. S365 for HGBSP1-9, in fig. S366 for HGBSP2-5, in fig. S367 for HGBSP2-7, in fig. S368 for HGBSP2-9, in fig. S369 for HGBSP3-5, in fig. S370 for HGBSP3-7, in fig. S371 for HGBSP3-9, in fig. S372 for AHGBSP1-5, in fig. S373 for AHGBSP1-7, in fig. S374 for AHGBSP1-9, in fig. S375 for AHGBSP2-5, in fig. S376 for AHGBSP2-7, in fig. S377 for AHGBSP2-9, in fig. S378 for AHGBSP3-5, in fig. S379 for AHGBSP3-7, in fig. S380 for AHGBSP3-9, in fig. S381 for 6-311++G(3df3pd), and in fig. S382 for def2-TZVP, all basis sets being employed in the fully uncontracted form.

The results for the Si atom are given in in fig. S383 for cc-pVDZ, in fig. S384 for cc-pVTZ, in fig. S385 for cc-pVQZ, in fig. S386 for cc-pV5Z, in fig. S387 for aug-cc-pVDZ, in fig. S388 for aug-cc-pVTZ, in fig. S389 for aug-cc-pVQZ, in fig. S390 for aug-cc-pV5Z, in fig. S391 for HGBSP1-5, in fig. S392 for HGBSP1-7, in fig. S393 for HGBSP1-9, in fig. S394 for HGBSP2-5, in fig. S395 for HGBSP2-7, in fig. S396 for HGBSP2-9, in fig. S397 for HGBSP3-5, in fig. S398 for HGBSP3-7, in fig. S399 for HGBSP3-9, in fig. S400 for AHGBSP1-5, in fig. S401 for AHGBSP1-7, in fig. S402 for AHGBSP1-9, in fig. S403 for AHGBSP2-5, in fig. S404 for AHGBSP2-7, in fig. S405 for AHGBSP2-9, in fig. S406 for AHGBSP3-5, in fig. S407 for AHGBSP3-7, in

fig. S408 for AHGBSP3-9, in fig. S409 for 6-311++G(3df3pd), and in fig. S410 for def2-TZVP, all basis sets being employed in the fully uncontracted form.

The results for the P atom are given in in fig. S411 for cc-pVDZ, in fig. S412 for cc-pVTZ, in fig. S413 for cc-pVQZ, in fig. S414 for cc-pV5Z, in fig. S415 for aug-cc-pVDZ, in fig. S416 for aug-cc-pVTZ, in fig. S417 for aug-cc-pVQZ, in fig. S418 for aug-cc-pV5Z, in fig. S419 for HGBSP1-5, in fig. S420 for HGBSP1-7, in fig. S421 for HGBSP1-9, in fig. S422 for HGBSP2-5, in fig. S423 for HGBSP2-7, in fig. S424 for HGBSP2-9, in fig. S425 for HGBSP3-5, in fig. S426 for HGBSP3-7, in fig. S427 for HGBSP3-9, in fig. S428 for AHGBSP1-5, in fig. S429 for AHGBSP1-7, in fig. S430 for AHGBSP1-9, in fig. S431 for AHGBSP2-5, in fig. S432 for AHGBSP2-7, in fig. S433 for AHGBSP2-9, in fig. S434 for AHGBSP3-5, in fig. S435 for AHGBSP3-7, in fig. S436 for AHGBSP3-9, in fig. S437 for 6-311++G(3df3pd), and in fig. S438 for def2-TZVP, all basis sets being employed in the fully uncontracted form.

The results for the S atom are given in in fig. S439 for cc-pVDZ, in fig. S440 for cc-pVTZ, in fig. S441 for cc-pVQZ, in fig. S442 for cc-pV5Z, in fig. S443 for aug-cc-pVDZ, in fig. S444 for aug-cc-pVTZ, in fig. S445 for aug-cc-pVQZ, in fig. S446 for aug-cc-pV5Z, in fig. S447 for HGBSP1-5, in fig. S448 for HGBSP1-7, in fig. S449 for HGBSP1-9, in fig. S450 for HGBSP2-5, in fig. S451 for HGBSP2-7, in fig. S452 for HGBSP2-9, in fig. S453 for HGBSP3-5, in fig. S454 for HGBSP3-7, in fig. S455 for HGBSP3-9, in fig. S456 for AHGBSP1-5, in fig. S457 for AHGBSP1-7, in fig. S458 for AHGBSP1-9, in fig. S459 for AHGBSP2-5, in fig. S460 for AHGBSP2-7, in fig. S461 for AHGBSP2-9, in fig. S462 for AHGBSP3-5, in fig. S463 for AHGBSP3-7, in fig. S464 for AHGBSP3-9, in fig. S465 for 6-311++G(3df3pd), and in fig. S466 for def2-TZVP, all basis sets being employed in the fully uncontracted form.

The results for the Cl atom are given in in fig. S467 for cc-pVDZ, in fig. S468 for cc-pVTZ, in fig. S469 for cc-pVQZ, in fig. S470 for cc-pV5Z, in fig. S471 for aug-cc-pVDZ, in fig. S472 for aug-cc-pVTZ, in fig. S473 for aug-cc-pVQZ, in fig. S474 for aug-cc-pV5Z, in fig. S475 for HGBSP1-5, in fig. S476 for HGBSP1-7, in fig. S477 for HGBSP1-9, in fig. S478 for HGBSP2-5, in fig. S479 for HGBSP2-7, in fig. S480 for HGBSP2-9, in fig. S481 for HGBSP3-5, in fig. S482 for HGBSP3-7, in fig. S483 for HGBSP3-9, in fig. S484 for AHGBSP1-5, in fig. S485 for AHGBSP1-7, in fig. S486 for AHGBSP1-9, in fig. S487 for AHGBSP2-5, in fig. S488 for AHGBSP2-7, in fig. S489 for AHGBSP2-9, in fig. S490 for AHGBSP3-5, in fig. S491 for AHGBSP3-7, in fig. S492 for AHGBSP3-9, in fig. S493 for 6-311++G(3df3pd), and in fig. S494 for def2-TZVP, all basis sets being employed in the fully uncontracted form.

The results for the Ar atom are given in in fig. S495 for cc-pVDZ, in fig. S496 for cc-pVTZ, in fig. S497 for cc-pVQZ, in fig. S498 for cc-pV5Z, in fig. S499 for aug-cc-pVDZ, in fig. S500 for aug-cc-pVTZ, in fig. S501 for aug-cc-pVQZ, in fig. S502 for aug-cc-pV5Z, in fig. S503 for HGBSP1-5, in fig. S504 for HGBSP1-7, in fig. S505 for HGBSP1-9, in fig. S506 for HGBSP2-5, in fig. S507 for HGBSP2-7, in fig. S508 for HGBSP2-9, in fig. S509 for HGBSP3-5, in fig. S510 for HGBSP3-7, in fig. S511 for HGBSP3-9, in fig. S512 for AHGBSP1-5, in fig. S513 for AHGBSP1-7, in fig. S514 for AHGBSP1-9, in fig. S515 for AHGBSP2-5, in fig. S516 for AHGBSP2-7, in fig. S517 for AHGBSP2-9, in fig. S518 for AHGBSP3-5, in fig. S519 for AHGBSP3-7, in fig. S520 for AHGBSP3-9, in fig. S521 for 6-311++G(3df3pd), and in fig. S522 for def2-TZVP, all basis sets being employed in the fully uncontracted form.

2.3 Tables of GTO Total Energies

Finally, we report the state specific total energies for all the studied states of all the studied atoms in all the studied Gaussian basis sets as a function of the field strength B, employing the real-orbital approximation.

For H, the results are given in in table S37 for cc-pVDZ, in table S38 for cc-pVTZ, in ta-

ble S39 for cc-pVQZ, in table S40 for cc-pV5Z, in table S41 for aug-cc-pVDZ, in table S42 for aug-cc-pVTZ, in table S43 for aug-cc-pVQZ, in table S44 for aug-cc-pV5Z, in table S45 for HGBSP1-5, in table S46 for HGBSP1-7, in table S47 for HGBSP1-9, in table S48 for HGBSP2-5, in table S49 for HGBSP2-7, in table S50 for HGBSP2-9, in table S51 for HGBSP3-5, in table S52 for HGBSP3-7, in table S53 for HGBSP3-9, in table S54 for AHGBSP1-5, in table S55 for AHGBSP1-7, in table S56 for AHGBSP1-9, in table S57 for AHGBSP2-5, in table S58 for AHGBSP2-7, in table S59 for AHGBSP2-9, in table S60 for AHGBSP3-5, in table S61 for AHGBSP3-7, in table S62 for AHGBSP3-9, in table S63 for 6-311++G(3df3pd), and in table S64 for def2-TZVP, all basis sets being employed in the fully uncontracted form.

For He, the results are given in in table S65 for cc-pVDZ, in table S66 for cc-pVTZ, in table S67 for cc-pVQZ, in table S68 for cc-pV5Z, in table S69 for aug-cc-pVDZ, in table S70 for aug-cc-pVTZ, in table S71 for aug-cc-pVQZ, in table S72 for aug-cc-pV5Z, in table S73 for HGBSP1-5, in table S74 for HGBSP1-7, in table S75 for HGBSP1-9, in table S76 for HGBSP2-5, in table S77 for HGBSP2-7, in table S78 for HGBSP2-9, in table S79 for HGBSP3-5, in table S80 for HGBSP3-7, in table S81 for HGBSP3-9, in table S82 for AHGBSP1-5, in table S83 for AHGBSP1-7, in table S84 for AHGBSP1-9, in table S85 for AHGBSP2-5, in table S86 for AHGBSP2-7, in table S87 for AHGBSP2-9, in table S88 for AHGBSP3-5, in table S89 for AHGBSP3-7, in table S90 for AHGBSP3-9, in table S91 for 6-311++G(3df3pd), and in table S92 for def2-TZVP, all basis sets being employed in the fully uncontracted form.

For Li, the results are given in in table S93 for cc-pVDZ, in table S94 for cc-pVTZ, in table S95 for cc-pVQZ, in table S96 for cc-pV5Z, in table S97 for aug-cc-pVDZ, in table S98 for aug-cc-pVTZ, in table S99 for aug-cc-pVQZ, in table S100 for aug-cc-pV5Z, in table S101 for HGBSP1-5, in table S102 for HGBSP1-7, in table S103 for HGBSP1-9, in table S104 for HGBSP2-5, in table S105 for HGBSP2-7, in table S106 for HGBSP2-9, in table S107 for HGBSP3-5, in table S108 for HGBSP3-7, in table S109 for HGBSP3-9, in table S110 for AHGBSP1-5, in table S111 for AHGBSP1-7, in table S112 for AHGBSP1-9, in table S113 for AHGBSP2-5, in table S114 for AHGBSP2-7, in table S115 for AHGBSP2-9, in table S116 for AHGBSP3-5, in table S117 for AHGBSP3-7, in table S118 for AHGBSP3-9, in table S119 for 6-311++G(3df3pd), and in table S120 for def2-TZVP, all basis sets being employed in the fully uncontracted form.

For Be, the results are given in in table S121 for cc-pVDZ, in table S122 for cc-pVTZ, in table S123 for cc-pVQZ, in table S124 for cc-pV5Z, in table S125 for aug-cc-pVDZ, in table S126 for aug-cc-pVTZ, in table S127 for aug-cc-pVQZ, in table S128 for aug-cc-pV5Z, in table S129 for HGBSP1-5, in table S130 for HGBSP1-7, in table S131 for HGBSP1-9, in table S132 for HGBSP2-5, in table S133 for HGBSP2-7, in table S134 for HGBSP2-9, in table S135 for HGBSP3-5, in table S136 for HGBSP3-7, in table S137 for HGBSP3-9, in table S138 for AHGBSP1-5, in table S139 for AHGBSP1-7, in table S140 for AHGBSP1-9, in table S141 for AHGBSP2-5, in table S142 for AHGBSP2-7, in table S143 for AHGBSP2-9, in table S144 for AHGBSP3-5, in table S145 for AHGBSP3-7, in table S146 for AHGBSP3-9, in table S147 for 6-311++G(3df3pd), and in table S148 for def2-TZVP, all basis sets being employed in the fully uncontracted form.

For B, the results are given in in table S149 for cc-pVDZ, in table S150 for cc-pVTZ, in table S151 for cc-pVQZ, in table S152 for cc-pV5Z, in table S153 for aug-cc-pVDZ, in table S154 for aug-cc-pVTZ, in table S155 for aug-cc-pVQZ, in table S156 for aug-cc-pV5Z, in table S157 for HGBSP1-5, in table S158 for HGBSP1-7, in table S159 for HGBSP1-9, in table S160 for HGBSP2-5, in table S161 for HGBSP2-7, in table S162 for HGBSP2-9, in table S163 for HGBSP3-5, in table S164 for HGBSP3-7, in table S165 for HGBSP3-9, in table S166 for AHGBSP1-5, in table S167 for AHGBSP1-7, in table S168 for AHGBSP1-9, in table S169 for AHGBSP2-5, in table S170 for AHGBSP2-7, in table S171 for AHGBSP2-9,

in table S172 for AHGBSP3-5, in table S173 for AHGBSP3-7, in table S174 for AHGBSP3-9, in table S175 for 6-311++G(3df3pd), and in table S176 for def2-TZVP, all basis sets being employed in the fully uncontracted form.

For C, the results are given in in table S177 for cc-pVDZ, in table S178 for cc-pVTZ, in table S179 for cc-pVQZ, in table S180 for cc-pV5Z, in table S181 for aug-cc-pVDZ, in table S182 for aug-cc-pVTZ, in table S183 for aug-cc-pVQZ, in table S184 for aug-cc-pV5Z, in table S185 for HGBSP1-5, in table S186 for HGBSP1-7, in table S187 for HGBSP1-9, in table S188 for HGBSP2-5, in table S189 for HGBSP2-7, in table S190 for HGBSP2-9, in table S191 for HGBSP3-5, in table S192 for HGBSP3-7, in table S193 for HGBSP3-9, in table S194 for AHGBSP1-5, in table S195 for AHGBSP1-7, in table S196 for AHGBSP1-9, in table S197 for AHGBSP2-5, in table S198 for AHGBSP2-7, in table S199 for AHGBSP2-9, in table S200 for AHGBSP3-5, in table S201 for AHGBSP3-7, in table S202 for AHGBSP3-9, in table S203 for 6-311++G(3df3pd), and in table S204 for def2-TZVP, all basis sets being employed in the fully uncontracted form.

For N, the results are given in in table S205 for cc-pVDZ, in table S206 for cc-pVTZ, in table S207 for cc-pVQZ, in table S208 for cc-pV5Z, in table S209 for aug-cc-pVDZ, in table S210 for aug-cc-pVTZ, in table S211 for aug-cc-pVQZ, in table S212 for aug-cc-pV5Z, in table S213 for HGBSP1-5, in table S214 for HGBSP1-7, in table S215 for HGBSP1-9, in table S216 for HGBSP2-5, in table S217 for HGBSP2-7, in table S218 for HGBSP2-9, in table S219 for HGBSP3-5, in table S220 for HGBSP3-7, in table S221 for HGBSP3-9, in table S222 for AHGBSP1-5, in table S223 for AHGBSP1-7, in table S224 for AHGBSP1-9, in table S225 for AHGBSP2-5, in table S226 for AHGBSP2-7, in table S227 for AHGBSP2-9, in table S228 for AHGBSP3-5, in table S229 for AHGBSP3-7, in table S230 for AHGBSP3-9, in table S231 for 6-311++G(3df3pd), and in table S232 for def2-TZVP, all basis sets being employed in the fully uncontracted form.

For O, the results are given in in table S233 for cc-pVDZ, in table S234 for cc-pVTZ, in table S235 for cc-pVQZ, in table S236 for cc-pV5Z, in table S237 for aug-cc-pVDZ, in table S238 for aug-cc-pVTZ, in table S239 for aug-cc-pVQZ, in table S240 for aug-cc-pV5Z, in table S241 for HGBSP1-5, in table S242 for HGBSP1-7, in table S243 for HGBSP1-9, in table S244 for HGBSP2-5, in table S245 for HGBSP2-7, in table S246 for HGBSP2-9, in table S247 for HGBSP3-5, in table S248 for HGBSP3-7, in table S249 for HGBSP3-9, in table S250 for AHGBSP1-5, in table S251 for AHGBSP1-7, in table S252 for AHGBSP1-9, in table S253 for AHGBSP2-5, in table S254 for AHGBSP2-7, in table S255 for AHGBSP2-9, in table S256 for AHGBSP3-5, in table S257 for AHGBSP3-7, in table S258 for AHGBSP3-9, in table S259 for 6-311++G(3df3pd), and in table S260 for def2-TZVP, all basis sets being employed in the fully uncontracted form.

For F, the results are given in in table S261 for cc-pVDZ, in table S262 for cc-pVTZ, in table S263 for cc-pVQZ, in table S264 for cc-pV5Z, in table S265 for aug-cc-pVDZ, in table S266 for aug-cc-pVTZ, in table S267 for aug-cc-pVQZ, in table S268 for aug-cc-pV5Z, in table S269 for HGBSP1-5, in table S270 for HGBSP1-7, in table S271 for HGBSP1-9, in table S272 for HGBSP2-5, in table S273 for HGBSP2-7, in table S274 for HGBSP2-9, in table S275 for HGBSP3-5, in table S276 for HGBSP3-7, in table S277 for HGBSP3-9, in table S278 for AHGBSP1-5, in table S279 for AHGBSP1-7, in table S280 for AHGBSP1-9, in table S281 for AHGBSP2-5, in table S282 for AHGBSP2-7, in table S283 for AHGBSP2-9, in table S284 for AHGBSP3-5, in table S285 for AHGBSP3-7, in table S286 for AHGBSP3-9, in table S287 for 6-311++G(3df3pd), and in table S288 for def2-TZVP, all basis sets being employed in the fully uncontracted form.

For Ne, the results are given in in table S289 for cc-pVDZ, in table S290 for cc-pVTZ, in table S291 for cc-pVQZ, in table S292 for cc-pV5Z, in table S293 for aug-cc-pVDZ, in table S294 for aug-cc-pVTZ, in table S295 for aug-cc-pVQZ, in table S296 for aug-cc-pV5Z, in table S297 for HGBSP1-5, in table S298 for HGBSP1-7, in table S299 for HGBSP1-9, in table S300 for HGBSP2-5, in table S301 for

HGBSP2-7, in table S302 for HGBSP2-9, in table S303 for HGBSP3-5, in table S304 for HGBSP3-7, in table S305 for HGBSP3-9, in table S306 for AHGBSP1-5, in table S307 for AHGBSP1-7, in table S308 for AHGBSP1-9, in table S309 for AHGBSP2-5, in table S310 for AHGBSP2-7, in table S311 for AHGBSP2-9, in table S312 for AHGBSP3-5, in table S313 for AHGBSP3-7, in table S314 for AHGBSP3-9, in table S315 for 6-311++G(3df3pd), and in table S316 for def2-TZVP, all basis sets being employed in the fully uncontracted form.

For Na, the results are given in in table S317 for cc-pVDZ, in table S318 for cc-pVTZ, in table S319 for cc-pVQZ, in table S320 for cc-pV5Z, in table S321 for aug-cc-pVDZ, in table S322 for aug-cc-pVTZ, in table S323 for aug-cc-pVQZ, in table S324 for aug-cc-pV5Z, in table S325 for HGBSP1-5, in table S326 for HGBSP1-7, in table S327 for HGBSP1-9, in table S328 for HGBSP2-5, in table S329 for HGBSP2-7, in table S330 for HGBSP2-9, in table S331 for HGBSP3-5, in table S332 for HGBSP3-7, in table S333 for HGBSP3-9, in table S334 for AHGBSP1-5, in table S335 for AHGBSP1-7, in table S336 for AHGBSP1-9, in table S337 for AHGBSP2-5, in table S338 for AHGBSP2-7, in table S339 for AHGBSP2-9, in table S340 for AHGBSP3-5, in table S341 for AHGBSP3-7, in table S342 for AHGBSP3-9, in table S343 for 6-311++G(3df3pd), and in table S344 for def2-TZVP, all basis sets being employed in the fully uncontracted form.

For Mg, the results are given in in table S345 for cc-pVDZ, in table S346 for cc-pVTZ, in table S347 for cc-pVQZ, in table S348 for cc-pV5Z, in table S349 for aug-cc-pVDZ, in table S350 for aug-cc-pVTZ, in table S351 for aug-cc-pVQZ, in table S352 for aug-cc-pV5Z, in table S353 for HGBSP1-5, in table S354 for HGBSP1-7, in table S355 for HGBSP1-9, in table S356 for HGBSP2-5, in table S357 for HGBSP2-7, in table S358 for HGBSP2-9, in table S359 for HGBSP3-5, in table S360 for HGBSP3-7, in table S361 for HGBSP3-9, in table S362 for AHGBSP1-5, in table S363 for AHGBSP1-7, in table S364 for AHGBSP1-9, in table S365 for AHGBSP2-5, in table S366 for AHGBSP2-7, in table S367 for AHGBSP2-9, in table S368 for AHGBSP3-5, in table S369 for AHGBSP3-7, in table S370 for AHGBSP3-9, in table S371 for 6-311++G(3df3pd), and in table S372 for def2-TZVP, all basis sets being employed in the fully uncontracted form.

For Al, the results are given in in table S373 for cc-pVDZ, in table S374 for cc-pVTZ, in table S375 for cc-pVQZ, in table S376 for cc-pV5Z, in table S377 for aug-cc-pVDZ, in table S378 for aug-cc-pVTZ, in table S379 for aug-cc-pVQZ, in table S380 for aug-cc-pV5Z, in table S381 for HGBSP1-5, in table S382 for HGBSP1-7, in table S383 for HGBSP1-9, in table S384 for HGBSP2-5, in table S385 for HGBSP2-7, in table S386 for HGBSP2-9, in table S387 for HGBSP3-5, in table S388 for HGBSP3-7, in table S389 for HGBSP3-9, in table S390 for AHGBSP1-5, in table S391 for AHGBSP1-7, in table S392 for AHGBSP1-9, in table S393 for AHGBSP2-5, in table S394 for AHGBSP2-7, in table S395 for AHGBSP2-9, in table S396 for AHGBSP3-5, in table S397 for AHGBSP3-7, in table S398 for AHGBSP3-9, in table S399 for 6-311++G(3df3pd), and in table S400 for def2-TZVP, all basis sets being employed in the fully uncontracted form.

For Si, the results are given in in table S401 for cc-pVDZ, in table S402 for cc-pVTZ, in table S403 for cc-pVQZ, in table S404 for cc-pV5Z, in table S405 for aug-cc-pVDZ, in table S406 for aug-cc-pVTZ, in table S407 for aug-cc-pVQZ, in table S408 for aug-cc-pV5Z, in table S409 for HGBSP1-5, in table S410 for HGBSP1-7, in table S411 for HGBSP1-9, in table S412 for HGBSP2-5, in table S413 for HGBSP2-7, in table S414 for HGBSP2-9, in table S415 for HGBSP3-5, in table S416 for HGBSP3-7, in table S417 for HGBSP3-9, in table S418 for AHGBSP1-5, in table S419 for AHGBSP1-7, in table S420 for AHGBSP1-9, in table S421 for AHGBSP2-5, in table S422 for AHGBSP2-7, in table S423 for AHGBSP2-9, in table S424 for AHGBSP3-5, in table S425 for AHGBSP3-7, in table S426 for AHGBSP3-9, in table S427 for 6-311++G(3df3pd), and in table S428 for def2-TZVP, all basis sets being employed in the fully uncontracted form.

For P, the results are given in in table S429 for cc-pVDZ, in table S430 for cc-pVTZ, in ta-

ble S431 for cc-pVQZ, in table S432 for cc-pV5Z, in table S433 for aug-cc-pVDZ, in table S434 for aug-cc-pVTZ, in table S435 for aug-cc-pVQZ, in table S436 for aug-cc-pV5Z, in table S437 for HGBSP1-5, in table S438 for HGBSP1-7, in table S439 for HGBSP1-9, in table S440 for HGBSP2-5, in table S441 for HGBSP2-7, in table S442 for HGBSP2-9, in table S443 for HGBSP3-5, in table S444 for HGBSP3-7, in table S445 for HGBSP3-9, in table S446 for AHGBSP1-5, in table S447 for AHGBSP1-7, in table S448 for AHGBSP1-9, in table S449 for AHGBSP2-5, in table S450 for AHGBSP2-7, in table S451 for AHGBSP2-9, in table S452 for AHGBSP3-5, in table S453 for AHGBSP3-7, in table S454 for AHGBSP3-9, in table S455 for 6-311++G(3df3pd), and in table S456 for def2-TZVP, all basis sets being employed in the fully uncontracted form.

For S, the results are given in in table S457 for cc-pVDZ, in table S458 for cc-pVTZ, in table S459 for cc-pVQZ, in table S460 for cc-pV5Z, in table S461 for aug-cc-pVDZ, in table S462 for aug-cc-pVTZ, in table S463 for aug-cc-pVQZ, in table S464 for aug-cc-pV5Z, in table S465 for HGBSP1-5, in table S466 for HGBSP1-7, in table S467 for HGBSP1-9, in table S468 for HGBSP2-5, in table S469 for HGBSP2-7, in table S470 for HGBSP2-9, in table S471 for HGBSP3-5, in table S472 for HGBSP3-7, in table S473 for HGBSP3-9, in table S474 for AHGBSP1-5, in table S475 for AHGBSP1-7, in table S476 for AHGBSP1-9, in table S477 for AHGBSP2-5, in table S478 for AHGBSP2-7, in table S479 for AHGBSP2-9, in table S480 for AHGBSP3-5, in table S481 for AHGBSP3-7, in table S482 for AHGBSP3-9, in table S483 for 6-311++G(3df3pd), and in table S484 for def2-TZVP, all basis sets being employed in the fully uncontracted form.

For Cl, the results are given in in table S485 for cc-pVDZ, in table S486 for cc-pVTZ, in table S487 for cc-pVQZ, in table S488 for cc-pV5Z, in table S489 for aug-cc-pVDZ, in table S490 for aug-cc-pVTZ, in table S491 for aug-cc-pVQZ, in table S492 for aug-cc-pV5Z, in table S493 for HGBSP1-5, in table S494 for HGBSP1-7, in table S495 for HGBSP1-9, in table S496 for HGBSP2-5, in table S497 for HGBSP2-7, in table S498 for HGBSP2-9, in table S499 for HGBSP3-5, in table S500 for HGBSP3-7, in table S501 for HGBSP3-9, in table S502 for AHGBSP1-5, in table S503 for AHGBSP1-7, in table S504 for AHGBSP1-9, in table S505 for AHGBSP2-5, in table S506 for AHGBSP2-7, in table S507 for AHGBSP2-9, in table S508 for AHGBSP3-5, in table S509 for AHGBSP3-7, in table S510 for AHGBSP3-9, in table S511 for 6-311++G(3df3pd), and in table S512 for def2-TZVP, all basis sets being employed in the fully uncontracted form.

For Ar, the results are given in in table S513 for cc-pVDZ, in table S514 for cc-pVTZ, in table S515 for cc-pVQZ, in table S516 for cc-pV5Z, in table S517 for aug-cc-pVDZ, in table S518 for aug-cc-pVTZ, in table S519 for aug-cc-pVQZ, in table S520 for aug-cc-pV5Z, in table S521 for HGBSP1-5, in table S522 for HGBSP1-7, in table S523 for HGBSP1-9, in table S524 for HGBSP2-5, in table S525 for HGBSP2-7, in table S526 for HGBSP2-9, in table S527 for HGBSP3-5, in table S528 for HGBSP3-7, in table S529 for HGBSP3-9, in table S530 for AHGBSP1-5, in table S531 for AHGBSP1-7, in table S532 for AHGBSP1-9, in table S533 for AHGBSP2-5, in table S534 for AHGBSP2-7, in table S535 for AHGBSP2-9, in table S536 for AHGBSP3-5, in table S537 for AHGBSP3-7, in table S538 for AHGBSP3-9, in table S539 for 6-311++G(3df3pd), and in table S540 for def2-TZVP, all basis sets being employed in the fully uncontracted form.

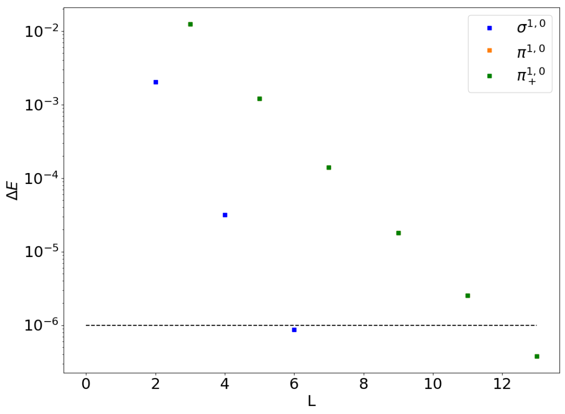

Figure S1: Convergence of the total energy of the considered states of the H atom as a function of the maximum angular momentum included in the fully numerical basis set.

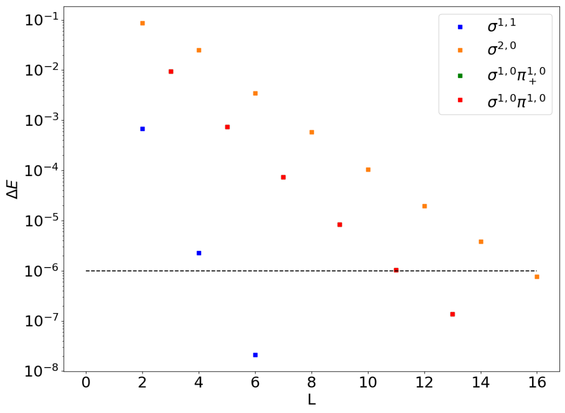

Figure S2: Convergence of the total energy of the considered states of the He atom as a function of the maximum angular momentum included in the fully numerical basis set.

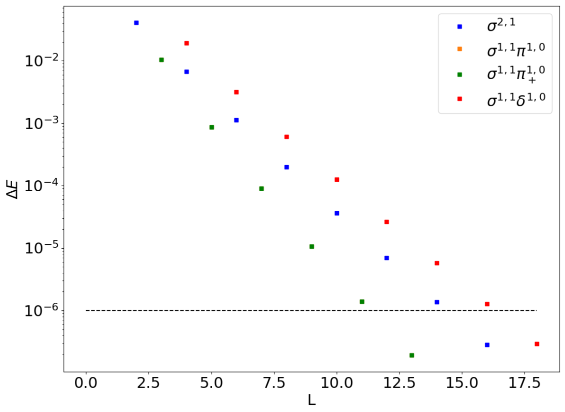

Figure S3: Convergence of the total energy of the considered states of the Li atom as a function of the maximum angular momentum included in the fully numerical basis set.

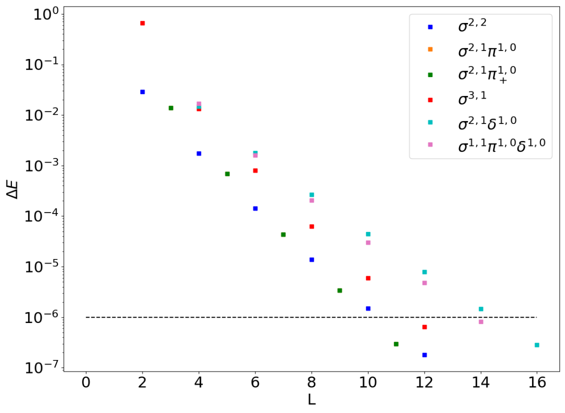

Figure S4: Convergence of the total energy of the considered states of the Be atom as a function of the maximum angular momentum included in the fully numerical basis set.

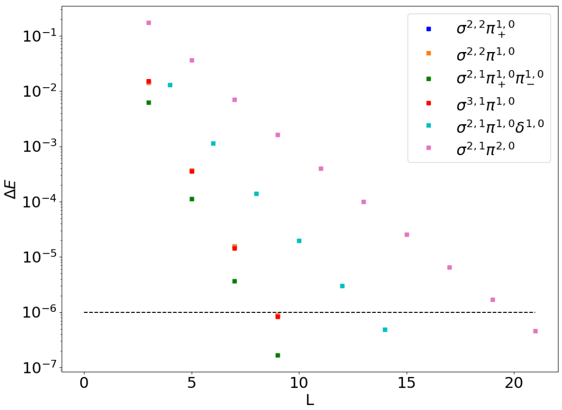

Figure S5: Convergence of the total energy of the considered states of the B atom as a function of the maximum angular momentum included in the fully numerical basis set.

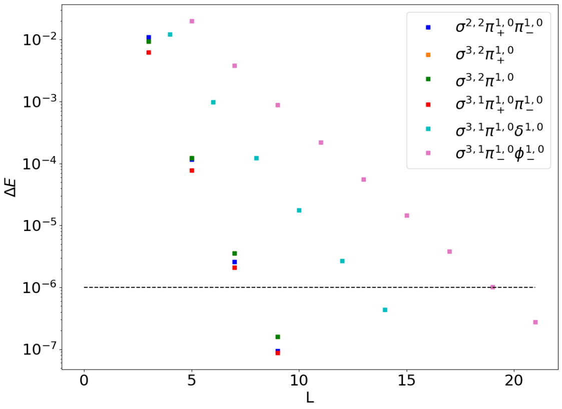

Figure S6: Convergence of the total energy of the considered states of the C atom as a function of the maximum angular momentum included in the fully numerical basis set.

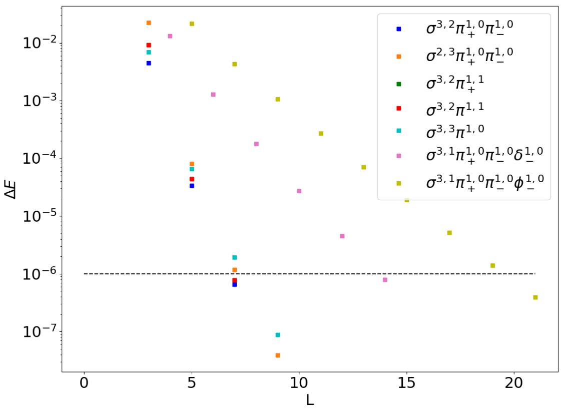

Figure S7: Convergence of the total energy of the considered states of the N atom as a function of the maximum angular momentum included in the fully numerical basis set.

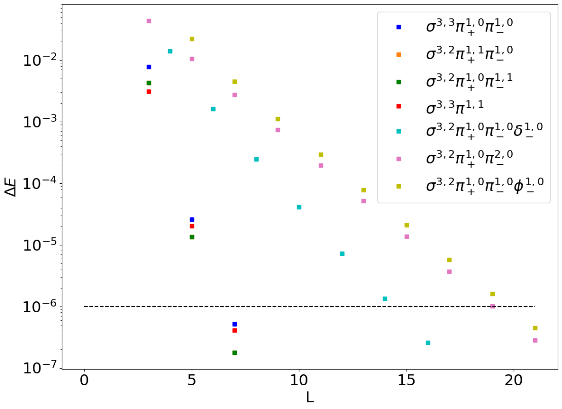

Figure S8: Convergence of the total energy of the considered states of the O atom as a function of the maximum angular momentum included in the fully numerical basis set.

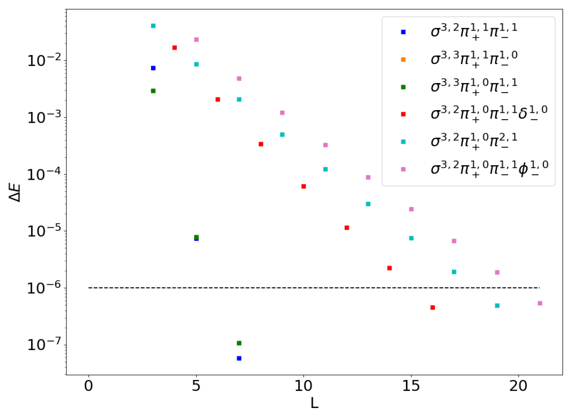

Figure S9: Convergence of the total energy of the considered states of the F atom as a function of the maximum angular momentum included in the fully numerical basis set.

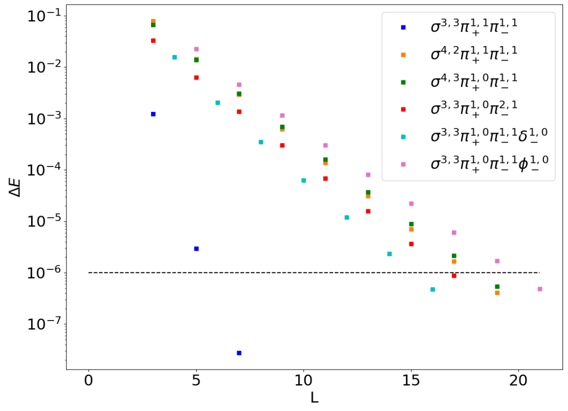

Figure S10: Convergence of the total energy of the considered states of the Ne atom as a function of the maximum angular momentum included in the fully numerical basis set.

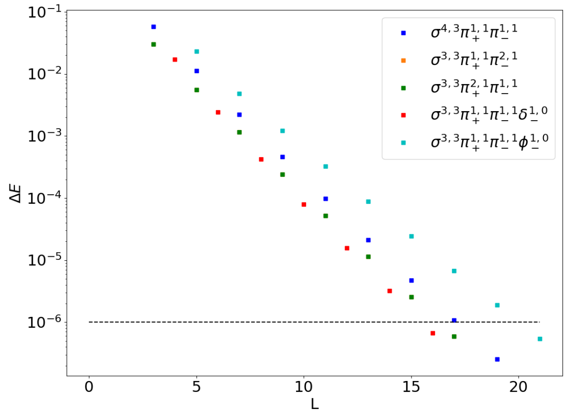

Figure S11: Convergence of the total energy of the considered states of the Na atom as a function of the maximum angular momentum included in the fully numerical basis set.

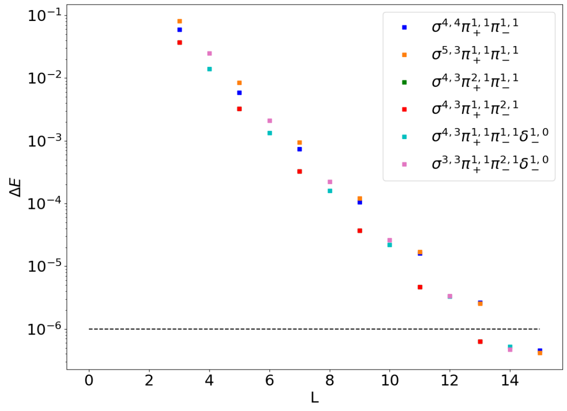

Figure S12: Convergence of the total energy of the considered states of the Mg atom as a function of the maximum angular momentum included in the fully numerical basis set.

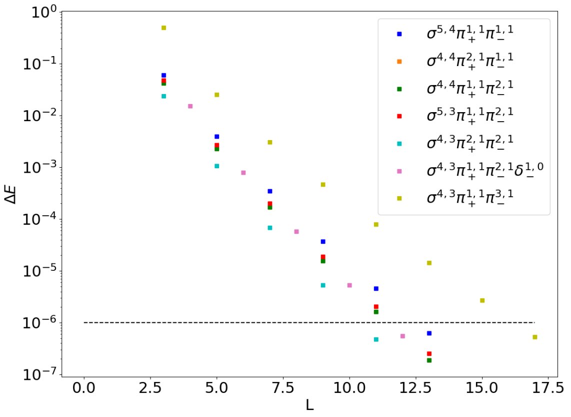

Figure S13: Convergence of the total energy of the considered states of the Al atom as a function of the maximum angular momentum included in the fully numerical basis set.

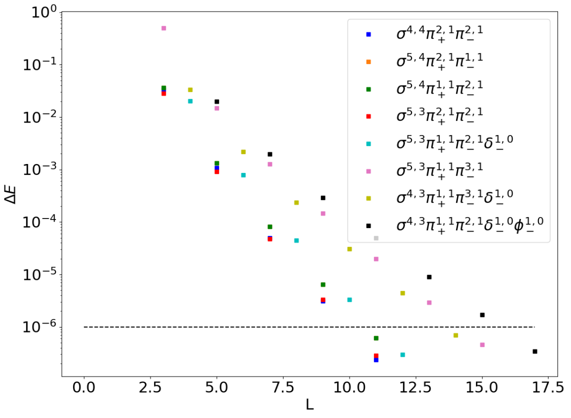

Figure S14: Convergence of the total energy of the considered states of the Si atom as a function of the maximum angular momentum included in the fully numerical basis set.

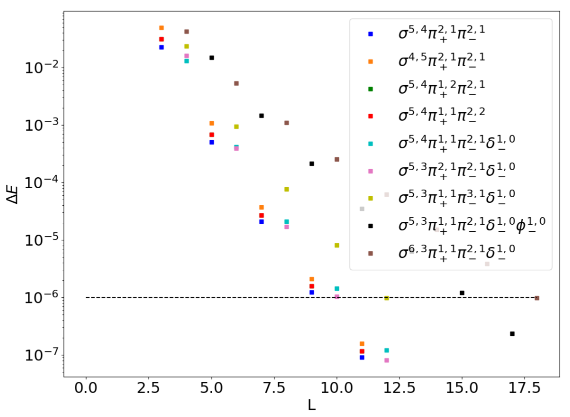

Figure S15: Convergence of the total energy of the considered states of the P atom as a function of the maximum angular momentum included in the fully numerical basis set.

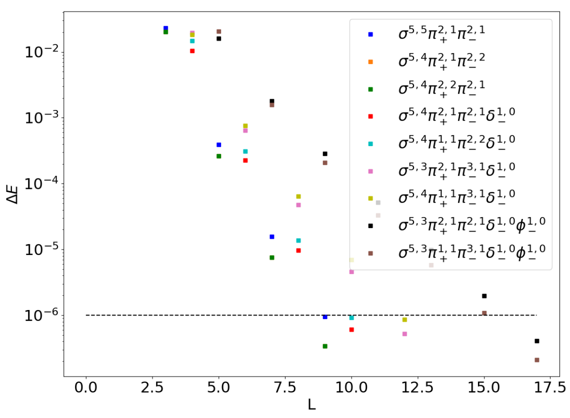

Figure S16: Convergence of the total energy of the considered states of the S atom as a function of the maximum angular momentum included in the fully numerical basis set.

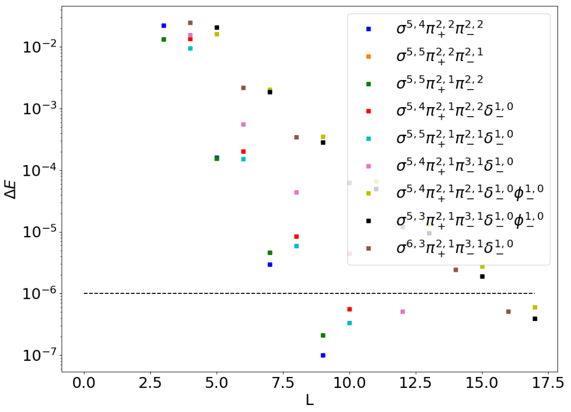

Figure S17: Convergence of the total energy of the considered states of the Cl atom as a function of the maximum angular momentum included in the fully numerical basis set.

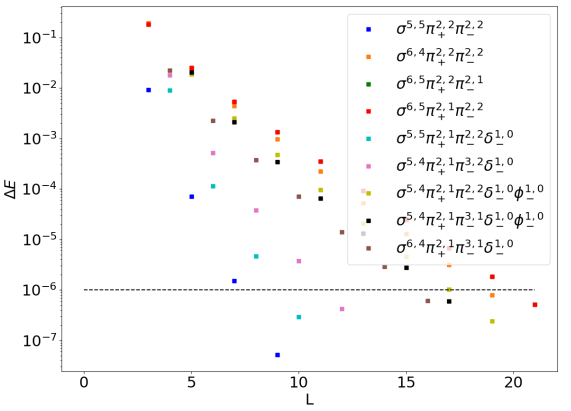

Figure S18: Convergence of the total energy of the considered states of the Ar atom as a function of the maximum angular momentum included in the fully numerical basis set.

Table S1: Complete basis set limit total energies in E_h for the H atom at all field strengths.

	$0.00B_0$	$0.10B_0$	$0.20B_0$	$0.30B_0$	$0.40B_0$	$0.50B_0$	$0.60B_0$
$\sigma^{1,0}$	−0.500000	−0.547526	−0.590382	−0.629187	−0.664605	−0.697211	−0.727462
$\pi_-^{1,0}$	−0.125000	−0.200846	−0.250539	−0.289092	−0.321355	−0.349477	−0.374624
$\pi_+^{1,0}$	−0.125000	−0.100846	−0.050539	0.010908	0.078645	0.150523	0.225376

Table S2: Complete basis set limit total energies in E_h for the He atom at all field strengths.

	$0.00B_0$	$0.10B_0$	$0.20B_0$	$0.30B_0$	$0.40B_0$	$0.50B_0$	$0.60B_0$
$\sigma^{1,1}$	−2.861680	−2.859709	−2.853845	−2.844217	−2.831013	−2.814451	−2.794755
$\sigma^{2,0}$	−2.174251	−2.257141	−2.317514	−2.367055	−2.419762	−2.477333	−2.532100
$\sigma^{1,0}\pi_+^{1,0}$	−2.131442	−2.159241	−2.161826	−2.152827	−2.136741	−2.115550	−2.090324
$\sigma^{1,0}\pi_-^{1,0}$	−2.131442	−2.259241	−2.361826	−2.452827	−2.536741	−2.615550	−2.690324

Table S3: Complete basis set limit total energies in E_h for the Li atom at all field strengths.

	$0.00B_0$	$0.10B_0$	$0.20B_0$	$0.30B_0$	$0.40B_0$	$0.50B_0$	$0.60B_0$
$\sigma^{2,1}$	−7.432751	−7.468560	−7.483999	−7.487940	−7.484914	−7.477407	−7.466912
$\sigma^{1,1}\pi_-^{1,0}$	−7.365081	−7.441744	−7.492192	−7.530686	−7.561898	−7.587886	−7.609754
$\sigma^{1,1}\pi_+^{1,0}$	−7.365081	−7.341744	−7.292192	−7.230686	−7.161898	−7.087886	−7.009754
$\sigma^{1,1}\delta_-^{1,0}$	−7.291977	−7.373551	−7.414902	−7.444023	−7.466109	−7.483227	−7.496452

Table S4: Complete basis set limit total energies in E_h for the Be atom at all field strengths.

	$0.00B_0$	$0.10B_0$	$0.20B_0$	$0.30B_0$	$0.40B_0$	$0.50B_0$	$0.60B_0$
$\sigma^{2,2}$	−14.573023	−14.558970	−14.520126	−14.462312	−14.390231	−14.307233	−14.215718
$\sigma^{2,1}\pi_-^{1,0}$	−14.512056	−14.645468	−14.750637	−14.835192	−14.904634	−14.962631	−15.011708
$\sigma^{2,1}\pi_+^{1,0}$	−14.512056	−14.545468	−14.550637	−14.535192	−14.504634	−14.462631	−14.411708
$\sigma^{3,1}$	−14.513724	−14.602670	−14.672033	−14.726282	−14.769071	−14.803012	−14.829966
$\sigma^{2,1}\delta_-^{1,0}$	−14.334213	−14.465624	−14.551429	−14.618315	−14.672140	−14.716078	−14.752260
$\sigma^{1,1}\pi_-^{1,0}\delta_-^{1,0}$	−14.190219	−14.377951	−14.517579	−14.633671	−14.733946	−14.822708	−14.902611

Table S5: Complete basis set limit total energies in E_h for the B atom at all field strengths.

	$0.00B_0$	$0.10B_0$	$0.20B_0$	$0.30B_0$	$0.40B_0$	$0.50B_0$	$0.60B_0$
$\sigma^{2,2}\pi_+^{1,0}$	−24.530274	−24.516277	−24.476314	−24.414360	−24.334168	−24.238806	−24.130714
$\sigma^{2,2}\pi_-^{1,0}$	−24.530274	−24.616277	−24.676314	−24.714360	−24.734168	−24.738806	−24.730714
$\sigma^{2,1}\pi_+^{1,0}\pi_-^{1,0}$	−24.452732	−24.587826	−24.695212	−24.779145	−24.843716	−24.892254	−24.927369
$\sigma^{3,1}\pi_-^{1,0}$	−24.451203	−24.639283	−24.805077	−24.951778	−25.082525	−25.199933	−25.306094
$\sigma^{2,1}\pi_-^{1,0}\delta_-^{1,0}$	−24.177886	−24.410798	−24.598528	−24.764740	−24.913789	−25.048432	−25.170850
$\sigma^{2,1}\pi_-^{2,0}$	−24.207652	−24.401767	−24.536033	−24.677216	−24.801939	−24.913888	−25.014977

Table S6: Complete basis set limit total energies in E_h for the C atom at all field strengths.

	$0.00B_0$	$0.10B_0$	$0.20B_0$	$0.30B_0$	$0.40B_0$	$0.50B_0$	$0.60B_0$
$\sigma^{2,2}\pi_+^{1,0}\pi_-^{1,0}$	−37.693740	−37.781132	−37.844271	−37.885369	−37.906921	−37.911255	−37.900408
$\sigma^{3,2}\pi_+^{1,0}$	−37.690933	−37.730147	−37.748536	−37.747851	−37.730109	−37.697226	−37.650912
$\sigma^{3,2}\pi_-^{1,0}$	−37.690933	−37.830147	−37.948536	−38.047851	−38.130109	−38.197226	−38.250912
$\sigma^{3,1}\pi_+^{1,0}\pi_-^{1,0}$	−37.599255	−37.788047	−37.955202	−38.102565	−38.232272	−38.346353	−38.446606
$\sigma^{3,1}\pi_-^{1,0}\delta_-^{1,0}$	−37.218715	−37.500849	−37.738631	−37.957786	−38.162296	−38.353891	−38.533847
$\sigma^{3,1}\pi_-^{1,0}\phi_-^{1,0}$	−37.192803	−37.468079	−37.689810	−37.891389	−38.078145	−38.252537	−38.416145

Table S7: Complete basis set limit total energies in E_h for the N atom at all field strengths.

	$0.00B_0$	$0.10B_0$	$0.20B_0$	$0.30B_0$	$0.40B_0$	$0.50B_0$	$0.60B_0$
$\sigma^{3,2}\pi_+^{1,0}\pi_-^{1,0}$	−54.404548	−54.544521	−54.664848	−54.766565	−54.851002	−54.919545	−54.973520
$\sigma^{2,3}\pi_+^{1,0}\pi_-^{1,0}$	−54.347429	−54.387277	−54.407262	−54.408490	−54.392352	−54.360278	−54.313623
$\sigma^{3,2}\pi_+^{1,1}$	−54.301096	−54.240642	−54.159809	−54.059866	−53.942345	−53.808764	−53.660532
$\sigma^{3,2}\pi_-^{1,1}$	−54.301096	−54.440642	−54.559809	−54.659866	−54.742345	−54.808764	−54.860532
$\sigma^{3,3}\pi_-^{1,0}$	−54.265587	−54.356508	−54.429651	−54.485966	−54.526659	−54.552974	−54.566102
$\sigma^{3,1}\pi_+^{1,0}\pi_-^{1,0}\delta_-^{1,0}$	−53.803609	−54.082735	−54.315114	−54.528193	−54.726576	−54.911967	−55.085368
$\sigma^{3,1}\pi_+^{1,0}\pi_-^{1,0}\phi_-^{1,0}$	−53.778555	−54.053067	−54.273063	−54.471877	−54.654760	−54.824088	−54.981353

Table S8: Complete basis set limit total energies in E_h for the O atom at all field strengths.

	$0.00B_0$	$0.10B_0$	$0.20B_0$	$0.30B_0$	$0.40B_0$	$0.50B_0$	$0.60B_0$
$\sigma^{3,3}\pi_+^{1,0}\pi_-^{1,0}$	−74.818980	−74.910450	−74.985079	−75.043452	−75.086368	−75.114715	−75.129394
$\sigma^{3,2}\pi_+^{1,1}\pi_-^{1,0}$	−74.814960	−74.855242	−74.876414	−74.879296	−74.864944	−74.834470	−74.788960
$\sigma^{3,2}\pi_+^{1,0}\pi_-^{1,1}$	−74.814960	−74.955242	−75.076414	−75.179296	−75.264944	−75.334470	−75.388960
$\sigma^{3,3}\pi_-^{1,1}$	−74.729990	−74.821240	−74.895244	−74.952669	−74.994401	−75.021400	−75.034619
$\sigma^{3,2}\pi_+^{1,0}\pi_-^{1,0}\delta_-^{1,0}$	−74.433062	−74.661096	−74.841129	−75.001203	−75.146421	−75.278751	−75.399247
$\sigma^{3,2}\pi_+^{1,0}\pi_-^{2,0}$	−74.479831	−74.687216	−74.846664	−74.979761	−75.093623	−75.192011	−75.277368
$\sigma^{3,2}\pi_+^{1,0}\pi_-^{1,0}\phi_-^{1,0}$	−74.408291	−74.632951	−74.803257	−74.952368	−75.085393	−75.204577	−75.311289

Table S9: Complete basis set limit total energies in E_h for the F atom at all field strengths.

	$0.00B_0$	$0.10B_0$	$0.20B_0$	$0.30B_0$	$0.40B_0$	$0.50B_0$	$0.60B_0$
$\sigma^{3,2}\pi_+^{1,1}\pi_-^{1,1}$	−99.416306	−99.457225	−99.480182	−99.485711	−99.474552	−99.447527	−99.405478
$\sigma^{3,3}\pi_+^{1,1}\pi_-^{1,0}$	−99.412213	−99.403969	−99.379401	−99.338952	−99.283238	−99.212963	−99.128856
$\sigma^{3,3}\pi_+^{1,0}\pi_-^{1,1}$	−99.412213	−99.503969	−99.579401	−99.638952	−99.683238	−99.712963	−99.728856
$\sigma^{3,2}\pi_+^{1,0}\pi_-^{1,1}\delta_-^{1,0}$	−98.892513	−99.119508	−99.297380	−99.454423	−99.595966	−99.724163	−99.840185
$\sigma^{3,2}\pi_+^{1,0}\pi_-^{2,1}$	−98.941723	−99.150686	−99.312808	−99.448678	−99.564957	−99.665084	−99.751286
$\sigma^{3,2}\pi_+^{1,0}\pi_-^{1,1}\phi_-^{1,0}$	−98.868033	−99.092454	−99.262310	−99.410710	−99.542705	−99.660480	−99.765347

Table S10: Complete basis set limit total energies in E_h for the Ne atom at all field strengths.

	$0.00B_0$	$0.10B_0$	$0.20B_0$	$0.30B_0$	$0.40B_0$	$0.50B_0$	$0.60B_0$
$\sigma^{3,3}\pi_+^{1,1}\pi_-^{1,1}$	−128.547098	−128.539298	−128.516006	−128.477529	−128.424308	−128.356868	−128.275772
$\sigma^{4,2}\pi_+^{1,1}\pi_-^{1,1}$	−127.999287	−128.076949	−128.121512	−128.144692	−128.152192	−128.147036	−128.130830
$\sigma^{4,3}\pi_+^{1,0}\pi_-^{1,1}$	−127.994937	−128.122716	−128.217746	−128.291913	−128.351124	−128.398619	−128.436147
$\sigma^{3,3}\pi_+^{1,0}\pi_-^{2,1}$	−127.928807	−128.090313	−128.207315	−128.299278	−128.372461	−128.430011	−128.473929
$\sigma^{3,3}\pi_+^{1,0}\pi_-^{1,1}\delta_-^{1,0}$	−127.876354	−128.054292	−128.184078	−128.293843	−128.388912	−128.471441	−128.542585
$\sigma^{3,3}\pi_+^{1,0}\pi_-^{1,1}\phi_-^{1,0}$	−127.851673	−128.027094	−128.149313	−128.251274	−128.337902	−128.411261	−128.472540

Table S11: Complete basis set limit total energies in E_h for the Na atom at all field strengths.

	$0.00B_0$	$0.10B_0$	$0.20B_0$	$0.30B_0$	$0.40B_0$	$0.50B_0$	$0.60B_0$
$\sigma^{4,3}\pi_+^{1,1}\pi_-^{1,1}$	−161.858954	−161.888141	−161.885898	−161.862906	−161.824537	−161.773800	−161.712464
$\sigma^{3,3}\pi_+^{1,1}\pi_-^{2,1}$	−161.786418	−161.848875	−161.867767	−161.862088	−161.837879	−161.798115	−161.744648
$\sigma^{3,3}\pi_+^{2,1}\pi_-^{1,1}$	−161.786418	−161.748875	−161.667767	−161.562088	−161.437879	−161.298115	−161.144648
$\sigma^{3,3}\pi_+^{1,1}\pi_-^{1,1}\delta_-^{1,0}$	−161.732630	−161.810151	−161.839235	−161.848064	−161.841978	−161.823159	−161.792788
$\sigma^{3,3}\pi_+^{1,1}\pi_-^{1,1}\phi_-^{1,0}$	−161.708120	−161.783495	−161.805807	−161.807981	−161.794896	−161.768571	−161.730145

Table S12: Complete basis set limit total energies in E_h for the Mg atom at all field strengths.

	$0.00B_0$	$0.10B_0$	$0.20B_0$	$0.30B_0$	$0.40B_0$	$0.50B_0$	$0.60B_0$
$\sigma^{4,4}\pi_+^{1,1}\pi_-^{1,1}$	−199.614636	−199.590846	−199.526386	−199.431762	−199.314406	−199.179300	−199.029952
$\sigma^{5,3}\pi_+^{1,1}\pi_-^{1,1}$	−199.548906	−199.627818	−199.671117	−199.688169	−199.685276	−199.666510	−199.634620
$\sigma^{4,3}\pi_+^{2,1}\pi_-^{1,1}$	−199.547308	−199.566180	−199.535009	−199.468777	−199.376449	−199.263609	−199.134056
$\sigma^{4,3}\pi_+^{1,1}\pi_-^{2,1}$	−199.547308	−199.666180	−199.735009	−199.768777	−199.776449	−199.763609	−199.734056
$\sigma^{4,3}\pi_+^{1,1}\pi_-^{1,1}\delta_-^{1,0}$	−199.430422	−199.560775	−199.635785	−199.682092	−199.707512	−199.716708	−199.712832
$\sigma^{3,3}\pi_+^{1,1}\pi_-^{2,1}\delta_-^{1,0}$	−199.290786	−199.480497	−199.599864	−199.680583	−199.734678	−199.768200	−199.784723

Table S13: Complete basis set limit total energies in E_h for the Al atom at all field strengths.

	$0.00B_0$	$0.10B_0$	$0.20B_0$	$0.30B_0$	$0.40B_0$	$0.50B_0$	$0.60B_0$
$\sigma^{5,4}\pi_+^{1,1}\pi_-^{1,1}$	−241.880838	−241.908479	−241.895591	−241.849618	−241.776914	−241.682316	−241.569488
$\sigma^{4,4}\pi_+^{2,1}\pi_-^{1,1}$	−241.877796	−241.848586	−241.768378	−241.648271	−241.496555	−241.319199	−241.120628
$\sigma^{4,4}\pi_+^{1,1}\pi_-^{2,1}$	−241.877796	−241.948586	−241.968378	−241.948271	−241.896555	−241.819199	−241.720628
$\sigma^{5,3}\pi_+^{1,1}\pi_-^{2,1}$	−241.791593	−241.965649	−242.093388	−242.184183	−242.245524	−242.282848	−242.300146
$\sigma^{4,3}\pi_+^{2,1}\pi_-^{2,1}$	−241.793154	−241.911009	−241.971972	−241.988183	−241.969022	−241.921133	−241.849331
$\sigma^{4,3}\pi_+^{1,1}\pi_-^{2,1}\delta_-^{1,0}$	−241.616057	−241.861572	−242.041120	−242.177392	−242.280751	−242.357861	−242.413395
$\sigma^{4,3}\pi_+^{1,1}\pi_-^{3,1}$	−241.616024	−241.811981	−241.956813	−242.063345	−242.139911	−242.192119	−242.224005

Table S14: Complete basis set limit total energies in E_h for the Si atom at all field strengths.

	$0.00B_0$	$0.10B_0$	$0.20B_0$	$0.30B_0$	$0.40B_0$	$0.50B_0$	$0.60B_0$
$\sigma^{4,4}\pi_+^{2,1}\pi_-^{2,1}$	−288.858938	−288.929643	−288.946298	−288.917337	−288.850307	−288.751199	−288.624726
$\sigma^{5,4}\pi_+^{2,1}\pi_-^{1,1}$	−288.855678	−288.880728	−288.859389	−288.798370	−288.703897	−288.581058	−288.433932
$\sigma^{5,4}\pi_+^{1,1}\pi_-^{2,1}$	−288.855678	−288.980728	−289.059389	−289.098370	−289.103897	−289.081058	−289.033932
$\sigma^{5,3}\pi_+^{2,1}\pi_-^{2,1}$	−288.763297	−288.936673	−289.060280	−289.141103	−289.185835	−289.199957	−289.187828
$\sigma^{5,3}\pi_+^{1,1}\pi_-^{2,1}\delta_-^{1,0}$	−288.510061	−288.812484	−289.056160	−289.258237	−289.426457	−289.566377	−289.682248
$\sigma^{5,3}\pi_+^{1,1}\pi_-^{3,1}$	−288.514046	−288.767174	−288.975479	−289.145088	−289.282638	−289.393137	−289.480496
$\sigma^{4,3}\pi_+^{1,1}\pi_-^{3,1}\delta_-^{1,0}$	−288.202067	−288.552450	−288.830888	−289.060634	−289.252921	−289.414764	−289.551115
$\sigma^{4,3}\pi_+^{1,1}\pi_-^{2,1}\delta_-^{1,0}\phi_-^{1,0}$	−288.145063	−288.509690	−288.793568	−289.030500	−289.231116	−289.401966	−289.547707

Table S15: Complete basis set limit total energies in E_h for the P atom at all field strengths.

	$0.00B_0$	$0.10B_0$	$0.20B_0$	$0.30B_0$	$0.40B_0$	$0.50B_0$	$0.60B_0$
$\sigma^{5,4}\pi_+^{2,1}\pi_-^{2,1}$	−340.719275	−340.844282	−340.921546	−340.955957	−340.952709	−340.916446	−340.851130
$\sigma^{4,5}\pi_+^{2,1}\pi_-^{2,1}$	−340.692692	−340.717561	−340.694543	−340.628721	−340.525395	−340.389240	−340.224216
$\sigma^{5,4}\pi_+^{1,2}\pi_-^{2,1}$	−340.654385	−340.678684	−340.654281	−340.586693	−340.481438	−340.343291	−340.176255
$\sigma^{5,4}\pi_+^{1,1}\pi_-^{2,2}$	−340.654385	−340.778684	−340.854281	−340.886693	−340.881438	−340.843291	−340.776255
$\sigma^{5,4}\pi_+^{1,1}\pi_-^{2,1}\delta_-^{1,0}$	−340.419742	−340.667795	−340.860411	−341.012938	−341.130878	−341.218590	−341.279733
$\sigma^{5,3}\pi_+^{2,1}\pi_-^{2,1}\delta_-^{1,0}$	−340.283176	−340.584393	−340.828535	−341.029198	−341.192101	−341.321994	−341.422880
$\sigma^{5,3}\pi_+^{1,1}\pi_-^{3,1}\delta_-^{1,0}$	−339.895832	−340.304305	−340.648835	−340.946960	−341.207612	−341.436644	−341.638422
$\sigma^{5,3}\pi_+^{1,1}\pi_-^{2,1}\delta_-^{1,0}\phi_-^{1,0}$	−339.826767	−340.242618	−340.583296	−340.880988	−341.144108	−341.377878	−341.586096
$\sigma^{6,3}\pi_+^{1,1}\pi_-^{2,1}\delta_-^{1,0}$	−339.930470	−340.279057	−340.571317	−340.832052	−341.059571	−341.257467	−341.429347

Table S16: Complete basis set limit total energies in E_h for the S atom at all field strengths.

	$0.00B_0$	$0.10B_0$	$0.20B_0$	$0.30B_0$	$0.40B_0$	$0.50B_0$	$0.60B_0$
$\sigma^{5,5}\pi_+^{2,1}\pi_-^{2,1}$	−397.513390	−397.591019	−397.625246	−397.619246	−397.576740	−397.501326	−397.396258
$\sigma^{5,4}\pi_+^{2,1}\pi_-^{2,2}$	−397.508055	−397.632986	−397.709550	−397.741718	−397.733894	−397.690184	−397.614227
$\sigma^{5,4}\pi_+^{2,2}\pi_-^{2,1}$	−397.508055	−397.532986	−397.509550	−397.441718	−397.333894	−397.190184	−397.014227
$\sigma^{5,4}\pi_+^{2,1}\pi_-^{2,1}\delta_-^{1,0}$	−397.238533	−397.483909	−397.676928	−397.830086	−397.946675	−398.030025	−398.083295
$\sigma^{5,4}\pi_+^{1,1}\pi_-^{2,2}\delta_-^{1,0}$	−397.153877	−397.393204	−397.579052	−397.726257	−397.837793	−397.916741	−397.966107
$\sigma^{5,3}\pi_+^{2,1}\pi_-^{3,1}\delta_-^{1,0}$	−396.607487	−397.017842	−397.365969	−397.666898	−397.928052	−398.154412	−398.349876
$\sigma^{5,4}\pi_+^{1,1}\pi_-^{3,1}\delta_-^{1,0}$	−396.769414	−397.108839	−397.422089	−397.671543	−397.883517	−398.062963	−398.213618
$\sigma^{5,3}\pi_+^{2,1}\pi_-^{2,1}\delta_-^{1,0}\phi_-^{1,0}$	−396.538645	−396.951286	−397.286874	−397.578737	−397.834474	−398.058656	−398.254609
$\sigma^{5,3}\pi_+^{1,1}\pi_-^{3,1}\delta_-^{1,0}\phi_-^{1,0}$	−396.014578	−396.528499	−396.967324	−397.363553	−397.723905	−398.052976	−398.354331

Table S17: Complete basis set limit total energies in E_h for the Cl atom at all field strengths.

	$0.00B_0$	$0.10B_0$	$0.20B_0$	$0.30B_0$	$0.40B_0$	$0.50B_0$	$0.60B_0$
$\sigma^{5,4}\pi_+^{2,2}\pi_-^{2,2}$	−459.489953	−459.515693	−459.494107	−459.428083	−459.321089	−459.176563	−458.997690
$\sigma^{5,5}\pi_+^{2,2}\pi_-^{2,1}$	−459.484491	−459.462230	−459.396468	−459.289701	−459.144990	−458.965421	−458.753889
$\sigma^{5,5}\pi_+^{2,1}\pi_-^{2,2}$	−459.484491	−459.562230	−459.596468	−459.589701	−459.544990	−459.465421	−459.353889
$\sigma^{5,4}\pi_+^{2,1}\pi_-^{2,2}\delta_-^{1,0}$	−459.111775	−459.347755	−459.533367	−459.682597	−459.795951	−459.875231	−459.922754
$\sigma^{5,5}\pi_+^{2,1}\pi_-^{2,1}\delta_-^{1,0}$	−459.123006	−459.320999	−459.471929	−459.586664	−459.666933	−459.714998	−459.733253
$\sigma^{5,4}\pi_+^{2,1}\pi_-^{3,1}\delta_-^{1,0}$	−458.673868	−459.033409	−459.331943	−459.585220	−459.799895	−459.980106	−460.129073
$\sigma^{5,4}\pi_+^{2,1}\pi_-^{2,1}\delta_-^{1,0}\phi_-^{1,0}$	−458.616127	−458.978599	−459.263544	−459.504760	−459.709931	−459.883490	−460.028531
$\sigma^{5,3}\pi_+^{2,1}\pi_-^{3,1}\delta_-^{1,0}\phi_-^{1,0}$	−457.813677	−458.325847	−458.761117	−459.153378	−459.509174	−459.832558	−460.126602
$\sigma^{6,3}\pi_+^{2,1}\pi_-^{3,1}\delta_-^{1,0}$	−457.893868	−458.359064	−458.792065	−459.152775	−459.478865	−459.771166	−460.032560

Table S18: Complete basis set limit total energies in E_h for the Ar atom at all field strengths.

	$0.00B_0$	$0.10B_0$	$0.20B_0$	$0.30B_0$	$0.40B_0$	$0.50B_0$	$0.60B_0$
$\sigma^{5,5}\pi_+^{2,2}\pi_-^{2,2}$	−526.817513	−526.795882	−526.731704	−526.626814	−526.483594	−526.304574	−526.092222
$\sigma^{6,4}\pi_+^{2,2}\pi_-^{2,2}$	−526.425353	−526.485202	−526.488225	−526.455622	−526.392829	−526.299807	−526.176880
$\sigma^{6,5}\pi_+^{2,2}\pi_-^{2,1}$	−526.419181	−526.429034	−526.382162	−526.301122	−526.193241	−526.058560	−525.896919
$\sigma^{6,5}\pi_+^{2,1}\pi_-^{2,2}$	−526.419181	−526.529034	−526.582162	−526.601122	−526.593241	−526.558560	−526.496919
$\sigma^{5,5}\pi_+^{2,1}\pi_-^{2,2}\delta_-^{1,0}$	−526.337543	−526.525031	−526.666661	−526.776546	−526.853656	−526.898636	−526.912976
$\sigma^{5,4}\pi_+^{2,1}\pi_-^{3,2}\delta_-^{1,0}$	−525.744429	−526.100081	−526.351675	−526.649228	−526.863409	−527.042607	−527.189466
$\sigma^{5,4}\pi_+^{2,1}\pi_-^{2,2}\delta_-^{1,0}\phi_-^{1,0}$	−525.684563	−526.045932	−526.327850	−526.564644	−526.764205	−526.930935	−527.067823
$\sigma^{5,4}\pi_+^{2,1}\pi_-^{3,1}\delta_-^{1,0}\phi_-^{1,0}$	−525.144630	−525.606848	−525.991931	−526.333963	−526.639617	−526.912901	−527.156757
$\sigma^{6,4}\pi_+^{2,1}\pi_-^{3,1}\delta_-^{1,0}$	−525.211942	−525.627965	−526.032442	−526.338491	−526.614367	−526.858938	−527.073246

Table S19: Mean absolute energy differences ΔE^{GTO} in mE_h for a variety of GTO basis sets for the H atom. States with all positive differences ΔE are shown in blue, and states that exhibit negative ΔE at one or more field strength(s) in red.

	$\sigma^{1,0}$	$\pi_-^{1,0}$	$\pi_+^{1,0}$
cc-pVDZ	1.97	905.76	905.76
cc-pVTZ	1.00	315.11	315.11
cc-pVQZ	0.61	169.30	169.30
cc-pV5Z	0.39	113.26	113.26
aug-cc-pVDZ	1.75	34.92	34.92
aug-cc-pVTZ	0.45	21.72	21.72
aug-cc-pVQZ	0.13	15.38	15.38
aug-cc-pV5Z	0.02	11.33	11.33
HGBSP1-5	0.64	5.67	5.67
HGBSP1-7	0.61	5.30	5.30
HGBSP1-9	0.61	5.27	5.27
HGBSP2-5	0.04	5.67	5.67
HGBSP2-7	0.01	5.30	5.30
HGBSP2-9	0.01	5.27	5.27
HGBSP3-5	0.04	4.52	4.52
HGBSP3-7	0.01	0.51	0.51
HGBSP3-9	0.01	0.43	0.43
AHGBSP1-5	0.62	5.63	5.63
AHGBSP1-7	0.61	5.30	5.30
AHGBSP1-9	0.61	5.26	5.26
AHGBSP2-5	0.03	5.63	5.63
AHGBSP2-7	0.01	5.30	5.30
AHGBSP2-9	0.01	5.26	5.26
AHGBSP3-5	0.03	4.48	4.48
AHGBSP3-7	0.01	0.50	0.50
AHGBSP3-9	0.01	0.43	0.43
6-311++G(3df,3pd)	0.86	65.39	65.39
def2-TZVP	1.03	1041.77	1041.77

Table S20: Mean absolute energy differences ΔE^{GTO} in mE_h for a variety of GTO basis sets for the He atom. States with all positive differences ΔE are shown in blue, and states that exhibit negative ΔE at one or more field strength(s) in red.

	$\sigma^{1,1}$	$\sigma^{2,0}$	$\sigma^{1,0}\pi_+^{1,0}$	$\sigma^{1,0}\pi_-^{1,0}$
cc-pVDZ	6.71	551.67	1623.56	1623.56
cc-pVTZ	0.80	210.00	766.74	766.74
cc-pVQZ	0.38	147.14	461.06	461.06
cc-pV5Z	0.22	109.75	324.07	324.07
aug-cc-pVDZ	6.45	55.88	103.37	103.37
aug-cc-pVTZ	0.63	48.33	59.47	59.47
aug-cc-pVQZ	0.22	42.09	35.84	35.84
aug-cc-pV5Z	0.08	36.56	25.18	25.18
HGBSP1-5	0.23	18.86	4.20	4.20
HGBSP1-7	0.18	12.75	4.03	4.03
HGBSP1-9	0.18	12.57	4.00	4.00
HGBSP2-5	0.05	10.02	4.20	4.20
HGBSP2-7	0.00	9.72	4.02	4.02
HGBSP2-9	0.00	9.54	3.99	3.99
HGBSP3-5	0.05	2.94	0.45	0.45
HGBSP3-7	0.00	1.75	0.29	0.29
HGBSP3-9	0.00	1.57	0.27	0.27
AHGBSP1-5	0.23	12.66	4.19	4.19
AHGBSP1-7	0.18	12.42	4.03	4.03
AHGBSP1-9	0.18	12.41	4.00	4.00
AHGBSP2-5	0.05	9.67	4.19	4.19
AHGBSP2-7	0.00	9.38	4.02	4.02
AHGBSP2-9	0.00	9.37	3.99	3.99
AHGBSP3-5	0.05	1.61	0.44	0.44
AHGBSP3-7	0.00	1.41	0.29	0.29
AHGBSP3-9	0.00	1.40	0.27	0.27
6-311++G(3df,3pd)	1.99	47.73	52.10	52.10
def2-TZVP	2.09	323.47	1156.18	1156.18

Table S21: Mean absolute energy differences ΔE^{GTO} in mE_h for a variety of GTO basis sets for the Li atom. States with all positive differences ΔE are shown in blue, and states that exhibit negative ΔE at one or more field strength(s) in red.

	$\sigma^{2,1}$	$\sigma^{1,1}\pi_-^{1,0}$	$\sigma^{1,1}\pi_+^{1,0}$	$\sigma^{1,1}\delta_-^{1,0}$
cc-pVDZ	7.59	10.14	10.14	78.13
cc-pVTZ	4.91	2.52	2.52	32.64
cc-pVQZ	2.13	1.37	1.37	21.56
cc-pV5Z	1.29	0.80	0.80	14.61
aug-cc-pVDZ	5.37	9.97	9.97	30.26
aug-cc-pVTZ	4.33	1.16	1.16	14.01
aug-cc-pVQZ	0.76	1.00	1.00	4.42
aug-cc-pV5Z				
HGBSP1-5	2.46	4.64	4.64	10.32
HGBSP1-7	2.27	4.40	4.40	9.85
HGBSP1-9	2.10	4.37	4.37	9.76
HGBSP2-5	2.46	0.57	0.57	13.79
HGBSP2-7	2.27	0.34	0.34	9.85
HGBSP2-9	2.10	0.31	0.31	9.76
HGBSP3-5	0.64	0.57	0.57	1.94
HGBSP3-7	0.47	0.34	0.34	1.51
HGBSP3-9	0.31	0.31	0.31	1.43
AHGBSP1-5	2.39	4.64	4.64	10.31
AHGBSP1-7	2.11	4.40	4.40	9.85
AHGBSP1-9	2.10	4.37	4.37	9.76
AHGBSP2-5	2.39	0.56	0.56	10.31
AHGBSP2-7	2.11	0.33	0.33	9.85
AHGBSP2-9	2.10	0.31	0.31	9.76
AHGBSP3-5	0.58	0.56	0.56	1.93
AHGBSP3-7	0.32	0.33	0.33	1.51
AHGBSP3-9	0.31	0.31	0.31	1.43
6-311++G(3df,3pd)	5.28	6.48	6.48	35.37
def2-TZVP	21.66	48.59	48.59	

Table S22: Mean absolute energy differences ΔE^{GTO} in mE_h for a variety of GTO basis sets for the Be atom. States with all positive differences ΔE are shown in blue, and states that exhibit negative ΔE at one or more field strength(s) in red.

	$\sigma^{2,2}$	$\sigma^{2,1}\pi_-^{1,0}$	$\sigma^{2,1}\pi_+^{1,0}$	$\sigma^{3,1}$	$\sigma^{2,1}\delta_-^{1,0}$	$\sigma^{1,1}\pi_-^{1,0}\delta_-^{1,0}$
cc-pVDZ	6.54	5.68	5.68	9.22	204.93	212.16
cc-pVTZ	2.20	2.45	2.45	4.68	108.03	118.72
cc-pVQZ	2.22	1.69	1.69	3.29	107.92	117.11
cc-pV5Z	1.19	0.79	0.79	2.10	71.35	81.80
aug-cc-pVDZ	2.00	4.15	4.15	6.77	31.45	47.52
aug-cc-pVTZ	0.90	1.15	1.15	1.65	21.31	36.34
aug-cc-pVQZ	0.80	0.76	0.76	0.79	16.12	30.51
aug-cc-pV5Z						
HGBSP1-5	1.11	2.38	2.38	4.95	8.09	24.86
HGBSP1-7	0.48	1.83	1.83	4.50	7.16	23.95
HGBSP1-9	0.45	1.81	1.81	4.48	7.10	23.90
HGBSP2-5	1.11	0.71	0.71	0.97	8.09	23.67
HGBSP2-7	0.48	0.19	0.19	0.54	7.16	22.78
HGBSP2-9	0.45	0.17	0.17	0.53	7.10	22.73
HGBSP3-5	0.64	0.59	0.59	0.64	1.63	16.94
HGBSP3-7	0.06	0.08	0.08	0.23	0.83	16.17
HGBSP3-9	0.03	0.06	0.06	0.22	0.78	16.13
AHGBSP1-5	0.86	2.27	2.27	4.85	8.01	24.85
AHGBSP1-7	0.47	1.82	1.82	4.49	7.15	23.94
AHGBSP1-9	0.45	1.81	1.81	4.48	7.10	23.90
AHGBSP2-5	0.86	0.60	0.60	0.87	8.01	23.66
AHGBSP2-7	0.47	0.18	0.18	0.54	7.15	22.77
AHGBSP2-9	0.45	0.17	0.17	0.53	7.10	22.73
AHGBSP3-5	0.43	0.49	0.49	0.56	1.56	16.93
AHGBSP3-7	0.05	0.07	0.07	0.23	0.83	16.17
AHGBSP3-9	0.03	0.06	0.06	0.22	0.78	16.13
6-311++G(3df,3pd)	2.51	2.56	2.56	4.38	38.35	52.84
def2-TZVP	2.76	4.10	4.10	6.98	116.04	126.84

Table S23: Mean absolute energy differences ΔE^{GTO} in mE_h for a variety of GTO basis sets for the B atom. States with all positive differences ΔE are shown in blue, and states that exhibit negative ΔE at one or more field strength(s) in red.

	$\sigma^{2,2}\pi_+^{1,0}$	$\sigma^{2,2}\pi_-^{1,0}$	$\sigma^{2,1}\pi_+^{1,0}\pi_-^{1,0}$	$\sigma^{3,1}\pi_-^{1,0}$	$\sigma^{2,1}\pi_-^{1,0}\delta_-^{1,0}$	$\sigma^{2,1}\pi_-^{2,0}$
cc-pVDZ	8.29	8.29	9.40	10.08	363.62	147.10
cc-pVTZ	2.62	2.62	2.98	3.77	137.33	79.79
cc-pVQZ	0.76	0.76	1.04	1.51	77.85	47.72
cc-pV5Z	0.31	0.31	0.34	0.61	61.08	33.71
aug-cc-pVDZ	5.34	5.34	7.98	8.39	58.62	40.59
aug-cc-pVTZ	1.52	1.52	1.78	1.85	34.41	32.10
aug-cc-pVQZ	0.37	0.37	0.53	0.49	24.29	23.16
aug-cc-pV5Z	0.13	0.13	0.16	0.17	20.83	16.62
HGBSP1-5	1.36	1.36	1.99	2.94	19.64	33.33
HGBSP1-7	0.75	0.75	1.42	2.41	18.92	24.73
HGBSP1-9	0.73	0.73	1.40	2.39	18.85	24.66
HGBSP2-5	0.71	0.71	0.61	0.63	22.81	22.18
HGBSP2-7	0.11	0.11	0.05	0.10	18.50	21.57
HGBSP2-9	0.09	0.09	0.03	0.09	18.43	21.55
HGBSP3-5	0.63	0.63	0.60	0.60	13.37	6.86
HGBSP3-7	0.03	0.03	0.04	0.07	12.64	6.33
HGBSP3-9	0.01	0.01	0.02	0.05	12.59	6.32
AHGBSP1-5	1.33	1.33	1.97	2.93	19.62	25.64
AHGBSP1-7	0.75	0.75	1.41	2.40	18.91	24.72
AHGBSP1-9	0.73	0.73	1.40	2.39	18.85	24.66
AHGBSP2-5	0.68	0.68	0.59	0.62	19.20	22.15
AHGBSP2-7	0.10	0.10	0.04	0.10	18.49	21.57
AHGBSP2-9	0.09	0.09	0.03	0.09	18.43	21.55
AHGBSP3-5	0.60	0.60	0.58	0.58	13.35	6.83
AHGBSP3-7	0.03	0.03	0.04	0.07	12.63	6.33
AHGBSP3-9	0.01	0.01	0.02	0.05	12.59	6.32
6-311++G(3df,3pd)	3.83	3.83	4.71	5.37	61.16	35.72
def2-TZVP	1.57	1.57	1.90	2.60	136.60	68.14

Table S24: Mean absolute energy differences ΔE^{GTO} in mE_h for a variety of GTO basis sets for the C atom. States with all positive differences ΔE are shown in blue, and states that exhibit negative ΔE at one or more field strength(s) in red.

	$\sigma^{2,2}\pi_+^{1,0}\pi_-^{1,0}$	$\sigma^{3,2}\pi_+^{1,0}$	$\sigma^{3,2}\pi_-^{1,0}$	$\sigma^{3,1}\pi_+^{1,0}\pi_-^{1,0}$	$\sigma^{3,1}\pi_-^{1,0}\delta_-^{1,0}$	$\sigma^{3,1}\pi_-^{1,0}\phi_-^{1,0}$
cc-pVDZ	11.18	10.47	10.47	12.98	729.62	
cc-pVTZ	4.03	4.20	4.20	4.67	310.83	2071.17
cc-pVQZ	1.22	1.51	1.51	1.56	169.29	1117.93
cc-pV5Z	0.40	0.59	0.59	0.55	119.98	659.65
aug-cc-pVDZ	8.82	8.53	8.53	11.62	93.54	
aug-cc-pVTZ	2.76	2.71	2.71	3.29	51.33	452.14
aug-cc-pVQZ	0.59	0.61	0.61	0.69	34.78	230.22
aug-cc-pV5Z	0.11	0.11	0.11	0.10	28.31	129.15
HGBSP1-5	1.36	1.89	1.89	2.08	16.43	
HGBSP1-7	0.59	1.12	1.12	1.33	15.23	
HGBSP1-9	0.58	1.11	1.11	1.32	15.17	
HGBSP2-5	0.81	0.80	0.80	0.77	15.71	14.08
HGBSP2-7	0.04	0.04	0.04	0.03	14.52	12.36
HGBSP2-9	0.03	0.03	0.03	0.02	14.46	12.22
HGBSP3-5	0.78	0.79	0.79	0.77	10.56	15.69
HGBSP3-7	0.02	0.03	0.03	0.03	9.44	13.95
HGBSP3-9	0.01	0.02	0.02	0.01	9.39	13.81
AHGBSP1-5	1.34	1.87	1.87	2.06	16.41	
AHGBSP1-7	0.59	1.12	1.12	1.33	15.23	
AHGBSP1-9	0.58	1.11	1.11	1.32	15.17	
AHGBSP2-5	0.79	0.78	0.78	0.76	15.70	14.06
AHGBSP2-7	0.04	0.04	0.04	0.03	14.51	12.36
AHGBSP2-9	0.03	0.03	0.03	0.02	14.46	12.22
AHGBSP3-5	0.77	0.77	0.77	0.75	10.55	14.06
AHGBSP3-7	0.02	0.03	0.03	0.02	9.44	12.35
AHGBSP3-9	0.01	0.02	0.02	0.01	9.39	12.22
6-311++G(3df,3pd)	5.45	5.54	5.54	6.56	92.72	2205.28
def2-TZVP	1.97	2.33	2.33	2.24	309.01	2069.46

	$\sigma^{3,2}\pi_+^{1,0}\pi_-^{1,0}$	$\sigma^{2,3}\pi_+^{1,0}\pi_-^{1,0}$	$\sigma^{3,2}\pi_+^{1,1}$	$\sigma^{3,2}\pi_-^{1,1}$	$\sigma^{3,3}\pi_-^{1,0}$	$\sigma^{3,1}\pi_+^{1,0}\pi_-^{1,0}\delta_-^{1,0}$	$\sigma^{3,1}\pi_+^{1,0}\pi_-^{1,0}\phi_-^{1,0}$
cc-pVDZ	14.92	19.06	51.13	51.13	15.98	1200.48	
cc-pVTZ	5.20	6.01	40.80	40.80	5.45	552.23	3217.83
cc-pVQZ	1.70	1.98	37.16	37.16	1.92	320.17	1792.72
cc-pV5Z	0.52	0.65	35.80	35.80	0.76	225.16	1116.81
aug-cc-pVDZ	13.34	16.01	48.83	48.83	13.07	187.04	
aug-cc-pVTZ	4.15	4.44	39.32	39.32	4.13	81.79	760.59
aug-cc-pVQZ	0.98	1.06	36.19	36.19	1.05	47.57	391.01
aug-cc-pV5Z	0.12	0.15	35.27	35.27	0.18	36.84	243.46
HGBSP1-5	1.69	3.75	37.81	37.81	2.43	8.06	
HGBSP1-7	0.65	2.70	36.76	36.76	1.39	6.78	
HGBSP1-9	0.64	2.69	36.75	36.75	1.38	6.71	
HGBSP2-5	1.06	1.09	36.20	36.20	1.07	7.65	14.68
HGBSP2-7	0.02	0.04	35.15	35.15	0.03	6.36	12.57
HGBSP2-9	0.01	0.02	35.14	35.14	0.02	6.29	12.41
HGBSP3-5	1.05	1.07	36.20	36.20	1.06	2.12	16.29
HGBSP3-7	0.02	0.02	35.15	35.15	0.02	0.85	12.57
HGBSP3-9	0.00	0.01	35.14	35.14	0.01	0.80	12.41
AHGBSP1-5	1.67	3.73	37.80	37.80	2.42	8.04	
AHGBSP1-7	0.65	2.70	36.76	36.76	1.39	6.77	
AHGBSP1-9	0.64	2.69	36.75	36.75	1.38	6.71	
AHGBSP2-5	1.04	1.07	36.19	36.19	1.06	7.63	14.66
AHGBSP2-7	0.02	0.04	35.15	35.15	0.03	6.36	12.57
AHGBSP2-9	0.01	0.02	35.14	35.14	0.02	6.29	12.41
AHGBSP3-5	1.04	1.06	36.19	36.19	1.05	2.11	14.66
AHGBSP3-7	0.02	0.02	35.15	35.15	0.02	0.85	12.57
AHGBSP3-9	0.00	0.01	35.14	35.14	0.01	0.80	12.41
6-311++G(3df,3pd)	7.11	7.65	42.35	42.35	6.79	172.47	2902.47
def2-TZVP	2.39	3.27	38.07	38.07	2.78	549.03	3215.00

Table S25: Mean absolute energy differences ΔE^{GTO} in mE_h for a variety of GTO basis sets for the N atom. States with all positive differences ΔE are shown in blue, and states that exhibit negative ΔE at one or more field strength(s) in red.

	$\sigma^{3,3}\pi_+^{1,0}\pi_-^{1,0}$	$\sigma^{3,2}\pi_+^{1,1}\pi_-^{1,0}$	$\sigma^{3,2}\pi_+^{1,0}\pi_-^{1,1}$	$\sigma^{3,3}\pi_-^{1,1}$	$\sigma^{3,2}\pi_+^{1,0}\pi_-^{1,0}\delta_-^{1,0}$	$\sigma^{3,2}\pi_+^{1,0}\pi_-^{2,0}$	$\sigma^{3,2}\pi_+^{1,0}\pi_-^{1,0}\phi_-^{1,0}$
cc-pVDZ	26.76	24.78	24.78	65.25	1962.35	488.34	
cc-pVTZ	7.91	7.45	7.45	48.55	916.56	257.27	4416.87
cc-pVQZ	2.31	2.04	2.04	43.42	537.24	150.08	2427.01
cc-pV5Z	0.60	0.36	0.36	41.75	372.98	68.42	1509.48
aug-cc-pVDZ	23.54	22.71	22.71	62.64	376.78	75.72	
aug-cc-pVTZ	6.77	6.43	6.43	47.68	169.53	46.80	1219.91
aug-cc-pVQZ	1.69	1.36	1.36	42.83	87.92	30.55	630.89
aug-cc-pV5Z	0.22	0.15	0.15	41.36	63.31	17.99	364.05
HGBSP1-5	2.99	2.25	2.25	43.20	9.16	22.21	
HGBSP1-7	1.60	0.84	0.84	41.80	7.35	19.65	
HGBSP1-9	1.58	0.82	0.82	41.78	7.28	19.47	
HGBSP2-5	1.43	1.08	1.08	42.57	8.95	6.35	15.97
HGBSP2-7	0.02	0.32	0.32	41.17	7.14	4.11	12.84
HGBSP2-9	0.01	0.34	0.34	41.15	7.07	4.01	12.71
HGBSP3-5	1.43	1.08	1.08	42.57	2.74	6.35	15.97
HGBSP3-7	0.02	0.32	0.32	41.17	0.96	4.11	14.43
HGBSP3-9	0.00	0.34	0.34	41.15	0.90	4.01	12.71
AHGBSP1-5	2.99	2.24	2.24	43.20	9.15	22.15	
AHGBSP1-7	1.60	0.84	0.84	41.80	7.35	19.64	
AHGBSP1-9	1.58	0.82	0.82	41.78	7.28	19.47	
AHGBSP2-5	1.43	1.08	1.08	42.56	8.93	6.31	15.95
AHGBSP2-7	0.02	0.32	0.32	41.17	7.13	4.11	12.84
AHGBSP2-9	0.01	0.34	0.34	41.15	7.07	4.01	12.71
AHGBSP3-5	1.43	1.08	1.08	42.56	2.73	6.31	15.95
AHGBSP3-7	0.02	0.32	0.32	41.17	0.96	4.11	12.84
AHGBSP3-9	0.00	0.34	0.34	41.15	0.90	4.01	12.71
6-311++G(3df,3pd)	10.55	10.37	10.37	51.25	345.12	53.61	4323.64
def2-TZVP	3.33	2.91	2.91	44.20	912.38	150.37	4413.13

Table S26: Mean absolute energy differences ΔE^{GTO} in mE_h for a variety of GTO basis sets for the O atom. States with all positive differences ΔE are shown in blue, and states that exhibit negative ΔE at one or more field strength(s) in red.

Table S27: Mean absolute energy differences ΔE^{GTO} in mE_h for a variety of GTO basis sets for the F atom. States with all positive differences ΔE are shown in blue, and states that exhibit negative ΔE at one or more field strength(s) in red.

	$\sigma^{3,2}\pi_+^{1,1}\pi_-^{1,1}$	$\sigma^{3,3}\pi_+^{1,1}\pi_-^{1,0}$	$\sigma^{3,3}\pi_+^{1,0}\pi_-^{1,1}$	$\sigma^{3,2}\pi_+^{1,0}\pi_-^{1,1}\delta_-^{1,0}$	$\sigma^{3,2}\pi_+^{1,0}\pi_-^{2,1}$	$\sigma^{3,2}\pi_+^{1,0}\pi_-^{1,1}\phi_-^{1,0}$
cc-pVDZ	40.23	38.21	38.21	2947.52	728.10	
cc-pVTZ	11.34	10.45	10.45	1386.97	397.78	6185.50
cc-pVQZ	3.21	2.59	2.59	847.40	237.54	3475.14
cc-pV5Z	0.62	0.34	0.34	598.47	105.84	2214.17
aug-cc-pVDZ	36.93	34.74	34.74	669.06	92.22	
aug-cc-pVTZ	10.29	9.66	9.66	316.70	58.37	1999.47
aug-cc-pVQZ	2.64	2.17	2.17	165.53	42.22	1076.10
aug-cc-pV5Z	0.30	0.14	0.14	112.08	27.56	643.86
HGBSP1-5	4.05	2.11	2.11	11.24	27.17	
HGBSP1-7	2.19	0.26	0.26	8.76	24.09	
HGBSP1-9	2.17	0.26	0.26	8.66	23.95	
HGBSP2-5	1.90	1.49	1.49	10.39	10.74	15.99
HGBSP2-7	0.02	0.38	0.38	8.00	8.16	13.21
HGBSP2-9	0.00	0.40	0.40	7.90	8.09	13.01
HGBSP3-5	1.90	1.49	1.49	3.20	10.74	15.99
HGBSP3-7	0.02	0.38	0.38	0.97	8.16	13.21
HGBSP3-9	0.00	0.40	0.40	0.90	8.09	13.01
AHGBSP1-5	4.05	2.11	2.11	11.23	27.07	
AHGBSP1-7	2.19	0.26	0.26	8.76	24.08	
AHGBSP1-9	2.17	0.26	0.26	8.66	23.95	
AHGBSP2-5	1.90	1.48	1.48	10.38	10.69	15.98
AHGBSP2-7	0.02	0.38	0.38	8.00	8.15	13.21
AHGBSP2-9	0.00	0.40	0.40	7.90	8.09	13.01
AHGBSP3-5	1.90	1.48	1.48	3.19	10.69	15.98
AHGBSP3-7	0.02	0.38	0.38	0.97	8.15	13.21
AHGBSP3-9	0.00	0.40	0.40	0.90	8.09	13.01
6-311++G(3df,3pd)	15.45	14.49	14.49	592.84	72.74	5958.67
def2-TZVP	4.42	3.48	3.48	1380.59	238.26	6179.45

Table S28: Mean absolute energy differences ΔE^{GTO} in mE_h for a variety of GTO basis sets for the Ne atom. States with all positive differences ΔE are shown in blue, and states that exhibit negative ΔE at one or more field strength(s) in red.

	$\sigma^{3,3}\pi_+^{1,1}\pi_-^{1,1}$	$\sigma^{4,2}\pi_+^{1,1}\pi_-^{1,1}$	$\sigma^{4,3}\pi_+^{1,0}\pi_-^{1,1}$	$\sigma^{3,3}\pi_+^{1,0}\pi_-^{2,1}$	$\sigma^{3,3}\pi_+^{1,0}\pi_-^{1,1}\delta_-^{1,0}$	$\sigma^{3,3}\pi_+^{1,0}\pi_-^{1,1}\phi_-^{1,0}$
cc-pVDZ	56.89	1048.25	1104.94	971.60	4106.68	
cc-pVTZ	15.31	622.10	663.00	538.45	1891.74	8369.68
cc-pVQZ	4.00	412.17	441.90	326.02	1180.85	4774.94
cc-pV5Z	0.60	225.80	243.21	143.64	846.75	3087.51
aug-cc-pVDZ	51.79	126.54	121.11	107.98	1029.04	
aug-cc-pVTZ	14.50	68.05	65.03	59.29	501.58	3186.47
aug-cc-pVQZ	3.71	45.46	43.68	42.18	276.81	1799.51
aug-cc-pV5Z	0.40	31.30	30.26	28.23	171.79	946.78
HGBSP1-5	2.71	10.92	9.17	22.41	10.88	
HGBSP1-7	0.29	6.99	5.23	19.29	8.01	
HGBSP1-9	0.27	6.66	4.90	19.20	7.91	
HGBSP2-5	2.45	9.26	8.74	9.79	10.41	15.80
HGBSP2-7	0.03	5.31	4.83	6.87	7.63	12.62
HGBSP2-9	0.00	4.98	4.59	6.81	7.54	12.46
HGBSP3-5	2.45	4.86	4.50	9.79	3.55	15.80
HGBSP3-7	0.03	1.28	1.09	6.87	0.92	12.62
HGBSP3-9	0.00	0.99	0.91	6.81	0.86	12.46
AHGBSP1-5	2.71	9.52	7.77	22.32	10.87	
AHGBSP1-7	0.29	6.60	4.84	19.28	8.01	
AHGBSP1-9	0.27	6.55	4.79	19.20	7.91	
AHGBSP2-5	2.44	7.86	7.34	9.74	10.40	15.79
AHGBSP2-7	0.03	4.92	4.64	6.87	7.63	12.61
AHGBSP2-9	0.00	4.87	4.62	6.81	7.54	12.46
AHGBSP3-5	2.44	3.68	3.33	9.74	3.54	15.79
AHGBSP3-7	0.03	0.92	0.94	6.87	0.91	12.61
AHGBSP3-9	0.00	0.88	0.94	6.81	0.86	12.46
6-311++G(3df,3pd)	20.81	91.34	87.24	88.52	883.93	8223.09
def2-TZVP	4.96	408.37	403.73	326.84	1882.35	8360.58

Table S29: Mean absolute energy differences ΔE^{GTO} in mE_h for a variety of GTO basis sets for the Na atom. States with all positive differences ΔE are shown in blue, and states that exhibit negative ΔE at one or more field strength(s) in red.

	$\sigma^{4,3}\pi_+^{1,1}\pi_-^{1,1}$	$\sigma^{3,3}\pi_+^{1,1}\pi_-^{2,1}$	$\sigma^{3,3}\pi_+^{2,1}\pi_-^{1,1}$	$\sigma^{3,3}\pi_+^{1,1}\pi_-^{1,1}\delta_-^{1,0}$	$\sigma^{3,3}\pi_+^{1,1}\pi_-^{1,1}\phi_-^{1,0}$
cc-pVDZ	17.52	56.79	56.79	82.64	
cc-pVTZ	9.90	7.18	7.18	28.81	151.41
cc-pVQZ	3.52	3.45	3.45	13.49	77.28
cc-pV5Z	3.33	2.78	2.78	11.08	69.37
aug-cc-pVDZ	15.89	55.39	55.39	47.32	
aug-cc-pVTZ	9.09	5.00	5.00	17.78	46.45
aug-cc-pVQZ	1.82	2.90	2.90	6.50	26.60
aug-cc-pV5Z					
HGBSP1-5	9.76	16.47	16.47	12.24	
HGBSP1-7	4.60	13.04	13.04	8.80	
HGBSP1-9	3.72	12.93	12.93	8.71	
HGBSP2-5	9.71	5.52	5.52	15.58	17.83
HGBSP2-7	4.55	2.08	2.08	8.65	13.24
HGBSP2-9	3.67	1.99	1.99	8.56	13.08
HGBSP3-5	6.39	5.52	5.52	4.66	19.44
HGBSP3-7	1.48	2.08	2.08	1.25	13.24
HGBSP3-9	0.65	1.99	1.99	1.16	13.08
AHGBSP1-5	7.24	16.44	16.44	12.22	
AHGBSP1-7	3.78	13.03	13.03	8.80	
AHGBSP1-9	3.72	12.93	12.93	8.71	
AHGBSP2-5	7.19	5.50	5.50	12.07	17.82
AHGBSP2-7	3.73	2.08	2.08	8.65	13.24
AHGBSP2-9	3.67	1.99	1.99	8.56	13.08
AHGBSP3-5	4.04	5.50	5.50	4.65	17.82
AHGBSP3-7	0.70	2.08	2.08	1.25	13.24
AHGBSP3-9	0.65	1.99	1.99	1.16	13.08
6-311++G(3df,3pd)	20.10	19.30	19.30	31.07	175.36
def2-TZVP	22.82	28.75	28.75	64.63	

Table S30: Mean absolute energy differences ΔE^{GTO} in mE_h for a variety of GTO basis sets for the Mg atom. States with all positive differences ΔE are shown in blue, and states that exhibit negative ΔE at one or more field strength(s) in red.

	$\sigma^{4,4}\pi_+^{1,1}\pi_-^{1,1}$	$\sigma^{5,3}\pi_+^{1,1}\pi_-^{1,1}$	$\sigma^{4,3}\pi_+^{2,1}\pi_-^{1,1}$	$\sigma^{4,3}\pi_+^{1,1}\pi_-^{2,1}$	$\sigma^{4,3}\pi_+^{1,1}\pi_-^{1,1}\delta_-^{1,0}$	$\sigma^{3,3}\pi_+^{1,1}\pi_-^{2,1}\delta_-^{1,0}$
cc-pVDZ	15.53	25.77	18.78	18.78	119.82	136.44
cc-pVTZ	4.31	12.35	6.62	6.62	53.05	71.55
cc-pVQZ	2.26	6.53	2.50	2.50	30.89	48.81
cc-pV5Z	1.81	4.02	1.32	1.32	31.92	48.30
aug-cc-pVDZ	10.04	22.30	16.95	16.95	30.94	57.61
aug-cc-pVTZ	3.26	4.44	3.20	3.20	16.20	37.78
aug-cc-pVQZ	1.11	2.20	1.18	1.18	7.76	29.29
aug-cc-pV5Z						
HGBSP1-5	8.47	19.07	11.66	11.66	11.04	37.88
HGBSP1-7	1.74	13.86	6.43	6.43	6.22	33.58
HGBSP1-9	1.65	13.78	6.32	6.32	6.11	33.45
HGBSP2-5	8.46	7.60	6.14	6.14	14.87	32.83
HGBSP2-7	1.72	2.56	0.92	0.92	6.17	28.51
HGBSP2-9	1.64	2.49	0.83	0.83	6.06	28.40
HGBSP3-5	6.77	6.28	5.62	5.62	5.32	27.62
HGBSP3-7	0.26	1.35	0.51	0.51	0.65	23.33
HGBSP3-9	0.19	1.28	0.43	0.43	0.55	23.21
AHGBSP1-5	6.10	18.26	10.84	10.84	10.68	37.85
AHGBSP1-7	1.73	13.85	6.42	6.42	6.21	33.58
AHGBSP1-9	1.65	13.78	6.32	6.32	6.11	33.45
AHGBSP2-5	6.09	6.85	5.33	5.33	10.63	32.80
AHGBSP2-7	1.71	2.56	0.91	0.91	6.17	28.51
AHGBSP2-9	1.64	2.49	0.83	0.83	6.06	28.40
AHGBSP3-5	4.58	5.61	4.90	4.90	5.06	27.60
AHGBSP3-7	0.25	1.34	0.50	0.50	0.64	23.32
AHGBSP3-9	0.19	1.28	0.43	0.43	0.55	23.21
6-311++G(3df,3pd)	9.94	14.79	10.26	10.26	25.15	46.89
def2-TZVP	13.09	29.14	20.48	20.48	47.30	72.89

	$\sigma^{5,4}\pi_+^{1,1}\pi_-^{1,1}$	$\sigma^{4,4}\pi_+^{2,1}\pi_-^{1,1}$	$\sigma^{4,4}\pi_+^{1,1}\pi_-^{2,1}$	$\sigma^{5,3}\pi_+^{1,1}\pi_-^{2,1}$	$\sigma^{4,3}\pi_+^{2,1}\pi_-^{2,1}$	$\sigma^{4,3}\pi_+^{1,1}\pi_-^{2,1}\delta_-^{1,0}$	$\sigma^{4,3}\pi_+^{1,1}\pi_-^{3,1}$
cc-pVDZ	20.83	15.14	15.14	22.05	18.35	121.22	115.97
cc-pVTZ	8.77	5.07	5.07	9.63	7.16	59.16	48.44
cc-pVQZ	2.90	1.96	1.96	2.83	2.52	41.74	29.64
cc-pV5Z	1.67	1.20	1.20	1.60	1.53	37.57	23.25
aug-cc-pVDZ	17.99	12.57	12.57	20.44	16.98	61.10	46.74
aug-cc-pVTZ	4.02	3.47	3.47	4.93	4.70	41.08	25.32
aug-cc-pVQZ	1.42	1.36	1.36	1.80	2.10	31.49	12.71
aug-cc-pV5Z	0.73	0.70	0.70	0.97	1.19	27.22	7.77
HGBSP1-5	13.69	8.71	8.71	14.93	11.56	36.06	29.56
HGBSP1-7	8.29	3.33	3.33	9.55	6.16	30.58	24.10
HGBSP1-9	8.21	3.22	3.22	9.46	6.04	30.46	23.99
HGBSP2-5	6.50	6.02	6.02	6.07	5.77	33.52	21.65
HGBSP2-7	1.18	0.65	0.65	0.76	0.38	28.05	16.18
HGBSP2-9	1.09	0.56	0.56	0.69	0.28	27.93	16.05
HGBSP3-5	5.97	5.59	5.59	5.83	5.73	29.78	10.86
HGBSP3-7	0.66	0.22	0.22	0.53	0.34	24.34	5.48
HGBSP3-9	0.58	0.14	0.14	0.46	0.24	24.22	5.35
AHGBSP1-5	13.63	8.63	8.63	14.87	11.49	35.99	29.50
AHGBSP1-7	8.29	3.32	3.32	9.55	6.15	30.58	23.13
AHGBSP1-9	8.21	3.22	3.22	9.46	6.04	30.46	23.98
AHGBSP2-5	6.44	5.95	5.95	6.02	5.71	33.45	21.55
AHGBSP2-7	1.17	0.64	0.64	0.75	0.37	28.04	16.15
AHGBSP2-9	1.09	0.56	0.56	0.69	0.28	27.93	16.05
AHGBSP3-5	5.92	5.52	5.52	5.78	5.66	29.72	11.70
AHGBSP3-7	0.65	0.21	0.21	0.52	0.33	24.33	5.44
AHGBSP3-9	0.58	0.14	0.14	0.46	0.24	24.22	6.29
6-311++G(3df,3pd)	11.82	8.79	8.79	11.91	9.83	59.54	43.60
def2-TZVP	13.56	9.80	9.80	14.70	12.06	57.73	48.59

Table S31: Mean absolute energy differences ΔE^{GTO} in mE_h for a variety of GTO basis sets for the Al atom. States with all positive differences ΔE are shown in blue, and states that exhibit negative ΔE at one or more field strength(s) in red.

	$\sigma^{4,4}\pi_+^{2,1}\pi_-^{2,1}$	$\sigma^{5,4}\pi_+^{2,1}\pi_-^{1,1}$	$\sigma^{5,4}\pi_+^{1,1}\pi_-^{2,1}$	$\sigma^{5,3}\pi_+^{2,1}\pi_-^{2,1}$	$\sigma^{5,3}\pi_+^{1,1}\pi_-^{2,1}\delta_-^{1,0}$	$\sigma^{5,3}\pi_+^{1,1}\pi_-^{3,1}$	$\sigma^{4,3}\pi_+^{1,1}\pi_-^{3,1}\delta_-^{1,0}$	$\sigma^{4,3}\pi_+^{1,1}\pi_-^{2,1}\delta_-^{1,0}\phi_-^{1,0}$
cc-pVDZ	19.72	20.74	20.74	22.78	182.64	173.86	328.09	
cc-pVTZ	7.24	8.68	8.68	9.51	77.49	59.32	128.09	604.11
cc-pVQZ	1.70	2.37	2.37	2.28	51.45	31.96	83.01	293.78
cc-pV5Z	0.84	1.09	1.09	1.06	47.59	28.15	74.59	197.90
aug-cc-pVDZ	15.70	17.08	17.08	20.14	73.61	49.53	114.03	
aug-cc-pVTZ	4.34	4.02	4.02	4.74	43.85	23.21	74.12	157.25
aug-cc-pVQZ	0.99	0.88	0.88	1.07	34.85	12.98	56.38	108.64
aug-cc-pV5Z	0.51	0.43	0.43	0.60	30.02	8.03	45.31	88.99
HGBSP1-5	9.65	12.23	12.23	13.02	39.77	25.10	61.46	
HGBSP1-7	3.19	5.78	5.78	6.60	33.27	18.65	54.96	
HGBSP1-9	3.07	5.67	5.67	6.49	33.15	18.55	54.84	
HGBSP2-5	6.83	6.81	6.81	6.71	34.79	18.81	57.25	70.40
HGBSP2-7	0.38	0.39	0.39	0.32	28.31	12.39	50.74	63.40
HGBSP2-9	0.27	0.32	0.32	0.22	28.20	12.29	50.63	63.21
HGBSP3-5	6.64	6.69	6.69	6.67	32.76	12.93	46.60	68.34
HGBSP3-7	0.20	0.28	0.28	0.28	26.31	6.56	40.16	61.34
HGBSP3-9	0.09	0.20	0.20	0.19	26.20	6.47	40.06	61.15
AHGBSP1-5	9.58	12.17	12.17	12.96	39.72	23.76	61.42	
AHGBSP1-7	3.18	5.77	5.77	6.59	33.26	18.65	54.95	
AHGBSP1-9	3.07	5.67	5.67	6.49	33.15	18.55	54.84	
AHGBSP2-5	6.77	6.75	6.75	6.66	34.74	18.77	57.21	70.36
AHGBSP2-7	0.37	0.39	0.39	0.32	28.31	12.38	50.74	63.39
AHGBSP2-9	0.27	0.32	0.32	0.22	28.20	12.29	50.63	63.21
AHGBSP3-5	6.58	6.64	6.64	6.62	32.72	12.89	46.56	68.30
AHGBSP3-7	0.19	0.27	0.27	0.28	26.30	6.56	40.16	61.34
AHGBSP3-9	0.09	0.20	0.20	0.19	26.20	6.47	40.06	61.15
6-311++G(3df,3pd)	8.66	10.10	10.10	10.14	61.63	35.01	90.47	552.52
def2-TZVP	10.67	12.40	12.40	12.07	71.56	58.95	116.34	598.36

Table S32: Mean absolute energy differences ΔE^{GTO} in mE_h for a variety of GTO basis sets for the Si atom. States with all positive differences ΔE are shown in blue, and states that exhibit negative ΔE at one or more field strength(s) in red.

	$\sigma^{5,4}\pi_+^{2,1}\pi_-^{2,1}$	$\sigma^{4,5}\pi_+^{2,1}\pi_-^{2,1}$	$\sigma^{5,4}\pi_+^{1,2}\pi_-^{2,1}$	$\sigma^{5,4}\pi_+^{1,1}\pi_-^{2,2}$	$\sigma^{5,4}\pi_+^{1,1}\pi_-^{2,1}\delta_-^{1,0}$	$\sigma^{5,3}\pi_+^{2,1}\pi_-^{2,1}\delta_-^{1,0}$	$\sigma^{5,3}\pi_+^{1,1}\pi_-^{3,1}\delta_-^{1,0}$	$\sigma^{5,3}\pi_+^{1,1}\pi_-^{2,1}\delta_-^{1,0}\phi_-^{1,0}$	$\sigma^{6,3}\pi_+^{1,1}\pi_-^{2,1}\delta_-^{1,0}$
cc-pVDZ	20.10	26.15	10.78	47.03	271.86	216.18	458.68		463.85
cc-pVTZ	7.62	8.88	16.48	33.13	112.44	67.56	167.96	891.67	203.35
cc-pVQZ	2.18	2.76	22.32	26.95	69.32	31.65	99.03	437.25	120.15
cc-pV5Z	0.93	1.22	23.74	25.39	64.24	25.58	87.17	284.17	97.24
aug-cc-pVDZ	16.82	21.67	9.65	43.43	82.04	56.79	132.85		119.97
aug-cc-pVTZ	3.93	4.58	20.65	28.45	49.09	21.61	82.65	213.45	73.72
aug-cc-pVQZ	0.71	0.95	24.02	25.03	37.09	10.27	62.25	132.22	56.70
aug-cc-pV5Z	0.28	0.36	24.50	24.53	32.45	4.68	49.78	107.28	45.25
HGBSP1-5	11.68	15.45	11.36	38.64	38.34	13.12	60.75		61.33
HGBSP1-7	3.96	7.73	19.09	30.89	30.51	5.40	53.03		52.58
HGBSP1-9	3.85	7.62	19.21	30.77	30.39	5.28	52.92		50.49
HGBSP2-5	7.93	8.09	16.82	32.24	35.36	9.65	56.81	69.90	57.05
HGBSP2-7	0.23	0.40	24.52	24.51	27.55	1.94	49.10	61.72	48.33
HGBSP2-9	0.12	0.29	24.63	24.40	27.43	1.82	48.99	61.54	46.26
HGBSP3-5	7.89	7.93	16.87	32.19	33.58	8.13	51.06	68.85	46.21
HGBSP3-7	0.20	0.24	24.57	24.47	25.78	0.42	43.38	62.28	37.90
HGBSP3-9	0.08	0.13	24.69	24.35	25.66	0.31	43.27	60.50	35.87
AHGBSP1-5	11.63	15.40	11.41	38.59	38.30	13.09	60.72		58.81
AHGBSP1-7	3.96	7.73	19.10	30.88	30.51	5.39	53.03		50.62
AHGBSP1-9	3.85	7.62	19.21	30.77	30.39	5.28	52.92		50.46
AHGBSP2-5	7.88	8.05	16.86	32.19	35.32	9.62	56.79	69.88	54.55
AHGBSP2-7	0.22	0.40	24.52	24.51	27.55	1.93	49.10	61.72	46.39
AHGBSP2-9	0.12	0.29	24.63	24.40	27.43	1.82	48.99	61.54	46.23
AHGBSP3-5	7.85	7.89	16.91	32.15	33.53	8.09	51.03	68.83	43.85
AHGBSP3-7	0.19	0.23	24.58	24.46	25.78	0.42	43.38	60.68	35.97
AHGBSP3-9	0.08	0.13	24.69	24.35	25.66	0.31	43.27	60.50	35.84
6-311++G(3df,3pd)	13.96	15.51	10.00	39.62	69.38	35.57	90.91	855.17	88.28
def2-TZVP	12.42	13.38	11.78	37.83	104.12	57.80	142.74	881.30	171.85

Table S33: Mean absolute energy differences ΔE^{GTO} in mE_h for a variety of GTO basis sets for the P atom. States with all positive differences ΔE are shown in blue, and states that exhibit negative ΔE at one or more field strength(s) in red.

	$\sigma^{5,5}\pi_+^{2,1}\pi_-^{2,1}$	$\sigma^{5,4}\pi_+^{2,1}\pi_-^{2,2}$	$\sigma^{5,4}\pi_+^{2,2}\pi_-^{2,1}$	$\sigma^{5,4}\pi_+^{2,1}\pi_-^{2,1}\delta_-^{1,0}$	$\sigma^{5,4}\pi_+^{1,1}\pi_-^{2,2}\delta_-^{1,0}$	$\sigma^{5,3}\pi_+^{2,1}\pi_-^{3,1}\delta_-^{1,0}$	$\sigma^{5,4}\pi_+^{1,1}\pi_-^{3,1}\delta_-^{1,0}$	$\sigma^{5,3}\pi_+^{2,1}\pi_-^{2,1}\delta_-^{1,0}\phi_-^{1,0}$	$\sigma^{5,3}\pi_+^{1,1}\pi_-^{3,1}\delta_-^{1,0}\phi_-^{1,0}$
cc-pVDZ	22.60	22.74	22.74	310.84	403.52	556.68	627.29		
cc-pVTZ	7.76	7.80	7.80	103.31	185.22	181.80	234.38	1125.89	1233.69
cc-pVQZ	2.70	2.08	2.08	49.39	120.77	76.95	127.82	538.57	601.05
cc-pV5Z	1.21	0.67	0.67	42.38	114.65	93.63	111.15	327.19	446.82
aug-cc-pVDZ	19.27	20.09	20.09	73.01	136.84	120.17	146.56		
aug-cc-pVTZ	4.81	4.40	4.40	29.83	88.12	57.72	88.24	286.25	358.68
aug-cc-pVQZ	0.91	0.56	0.56	14.98	70.79	35.55	65.07	137.18	226.01
aug-cc-pV5Z	0.30	0.10	0.10	8.44	65.54	18.49	49.91	95.97	178.01
HGBSP1-5	13.50	14.03	14.03	12.98	68.92	23.22	58.48		
HGBSP1-7	4.29	4.78	4.78	3.70	59.57	14.04	49.17		
HGBSP1-9	4.16	4.65	4.65	3.57	59.43	13.91	49.04		
HGBSP2-5	9.41	9.18	9.18	11.00	64.35	20.24	55.75	32.90	91.11
HGBSP2-7	0.22	0.12	0.12	1.73	55.02	11.06	46.44	22.97	81.28
HGBSP2-9	0.09	0.20	0.20	1.60	54.88	10.93	46.31	22.76	81.08
HGBSP3-5	9.38	9.17	9.17	9.65	62.27	15.57	50.54	32.12	87.81
HGBSP3-7	0.19	0.11	0.11	0.39	52.95	6.40	41.26	22.20	77.99
HGBSP3-9	0.07	0.19	0.19	0.26	52.82	6.28	41.13	21.99	77.80
AHGBSP1-5	13.47	14.00	14.00	12.95	68.88	23.21	58.43		
AHGBSP1-7	4.29	4.78	4.78	3.70	59.57	14.03	49.17		
AHGBSP1-9	4.16	4.65	4.65	3.57	59.43	13.91	49.04		
AHGBSP2-5	9.38	9.14	9.14	10.97	64.32	20.23	55.71	32.88	91.09
AHGBSP2-7	0.22	0.12	0.12	1.73	55.01	11.06	46.44	22.97	81.28
AHGBSP2-9	0.09	0.20	0.20	1.60	54.88	10.93	46.31	22.76	81.08
AHGBSP3-5	9.36	9.13	9.13	9.62	62.23	15.55	50.50	32.10	87.79
AHGBSP3-7	0.19	0.11	0.11	0.39	52.95	6.40	41.26	22.19	77.99
AHGBSP3-9	0.06	0.19	0.19	0.26	52.82	6.28	41.13	21.99	77.80
6-311++G(3df,3pd)	12.71	12.60	12.60	40.56	107.12	54.85	94.77	1059.60	1088.91
def2-TZVP	13.29	13.39	13.39	88.89	170.90	130.38	215.31	1108.95	1179.07

Table S34: Mean absolute energy differences ΔE^{GTO} in mE_h for a variety of GTO basis sets for the S atom. States with all positive differences ΔE are shown in blue, and states that exhibit negative ΔE at one or more field strength(s) in red.

	$\sigma^{5,4}\pi_+^{2,2}\pi_-^{2,2}$	$\sigma^{5,5}\pi_+^{2,2}\pi_-^{2,1}$	$\sigma^{5,5}\pi_+^{2,1}\pi_-^{2,2}$	$\sigma^{5,4}\pi_+^{2,1}\pi_-^{2,2}\delta_-^{1,0}$	$\sigma^{5,5}\pi_+^{2,1}\pi_-^{2,1}\delta_-^{1,0}$	$\sigma^{5,4}\pi_+^{2,1}\pi_-^{3,1}\delta_-^{1,0}$	$\sigma^{5,4}\pi_+^{2,1}\pi_-^{2,1}\delta_-^{1,0}\phi_-^{1,0}$	$\sigma^{5,3}\pi_+^{2,1}\pi_-^{3,1}\delta_-^{1,0}\phi_-^{1,0}$	$\sigma^{6,3}\pi_+^{2,1}\pi_-^{3,1}\delta_-^{1,0}$
cc-pVDZ	25.95	21.12	21.12	421.51	395.56	723.91			1012.37
cc-pVTZ	8.60	7.20	7.20	162.16	142.05	263.74	1568.47	1655.59	505.32
cc-pVQZ	2.54	2.18	2.18	79.81	66.57	118.75	785.32	822.70	231.90
cc-pV5Z	0.96	0.85	0.85	67.95	54.22	89.21	486.72	523.69	139.39
aug-cc-pVDZ	23.87	19.04	19.04	107.99	100.30	146.08			219.44
aug-cc-pVTZ	5.63	5.06	5.06	44.99	40.28	62.76	504.27	529.76	91.58
aug-cc-pVQZ	1.11	0.79	0.79	23.31	20.55	40.54	261.78	285.25	49.98
aug-cc-pV5Z	0.28	0.10	0.10	15.75	11.79	20.32	153.38	162.83	27.06
HGBSP1-5	17.61	13.51	13.51	16.47	15.87	23.90			28.32
HGBSP1-7	6.60	2.52	2.52	5.43	4.87	12.90			17.08
HGBSP1-9	6.45	2.38	2.38	5.28	4.72	12.75			16.60
HGBSP2-5	11.19	10.91	10.91	12.82	12.16	21.79	33.92	42.09	26.39
HGBSP2-7	0.19	0.10	0.10	1.81	1.16	10.80	21.85	30.15	15.15
HGBSP2-9	0.04	0.22	0.22	1.70	1.01	10.65	21.59	29.90	14.67
HGBSP3-5	11.18	10.90	10.90	11.18	11.36	17.49	33.29	41.06	19.32
HGBSP3-7	0.18	0.09	0.09	0.18	0.37	6.50	21.22	27.53	8.26
HGBSP3-9	0.03	0.22	0.22	0.09	0.22	6.36	20.96	28.88	7.75
AHGBSP1-5	17.59	13.49	13.49	16.45	15.85	23.88			27.89
AHGBSP1-7	6.60	2.52	2.52	5.43	4.86	12.90			16.77
AHGBSP1-9	6.45	2.38	2.38	5.28	4.72	12.75			16.60
AHGBSP2-5	11.16	10.88	10.88	12.79	12.14	21.78	33.90	42.08	25.95
AHGBSP2-7	0.19	0.10	0.10	1.81	1.16	10.80	21.84	30.15	14.83
AHGBSP2-9	0.04	0.22	0.22	1.70	1.01	10.65	21.59	29.90	14.66
AHGBSP3-5	11.15	10.88	10.88	11.16	11.34	17.47	33.27	39.45	18.92
AHGBSP3-7	0.18	0.10	0.10	0.18	0.37	6.50	21.22	27.52	7.91
AHGBSP3-9	0.03	0.22	0.22	0.09	0.22	6.36	20.96	27.28	16.41
6-311++G(3df,3pd)	16.23	14.77	14.77	55.50	46.37	58.37	1465.37	1413.26	60.62
def2-TZVP	14.78	13.46	13.46	132.22	112.03	257.65	1538.47	1583.36	399.39

Table S35: Mean absolute energy differences ΔE^{GTO} in mE_h for a variety of GTO basis sets for the Cl atom. States with all positive differences ΔE are shown in blue, and states that exhibit negative ΔE at one or more field strength(s) in red.

	$\sigma^{5,5}\pi_+^{2,2}\pi_-^{2,2}$	$\sigma^{6,4}\pi_+^{2,2}\pi_-^{2,2}$	$\sigma^{6,5}\pi_+^{2,2}\pi_-^{2,1}$	$\sigma^{6,5}\pi_+^{2,1}\pi_-^{2,2}$	$\sigma^{5,5}\pi_+^{2,1}\pi_-^{2,2}\delta_-^{1,0}$	$\sigma^{5,4}\pi_+^{2,1}\pi_-^{3,2}\delta_-^{1,0}$	$\sigma^{5,4}\pi_+^{2,1}\pi_-^{2,2}\delta_-^{1,0}\phi_-^{1,0}$	$\sigma^{5,4}\pi_+^{2,1}\pi_-^{3,1}\delta_-^{1,0}\phi_-^{1,0}$	$\sigma^{6,4}\pi_+^{2,1}\pi_-^{3,1}\delta_-^{1,0}$
cc-pVDZ	21.61	442.49	466.52	466.52	518.38	870.70			1143.18
cc-pVTZ	7.33	295.05	313.75	313.75	198.98	367.55	2117.67	2221.47	621.01
cc-pVQZ	2.27	174.11	190.46	190.46	103.89	185.94	1141.80	1174.37	311.79
cc-pV5Z	1.00	144.27	145.76	145.76	88.68	140.33	729.55	763.58	199.22
aug-cc-pVDZ	20.25	91.19	85.99	85.99	137.71	194.38			263.78
aug-cc-pVTZ	5.71	46.06	45.78	45.78	59.51	88.71	765.58	779.59	111.42
aug-cc-pVQZ	1.17	24.39	24.94	24.94	30.62	49.44	430.46	446.42	57.65
aug-cc-pV5Z	0.28	19.90	21.08	21.08	20.38	38.43	236.97	240.93	35.41
HGBSP1-5	15.12	36.86	34.03	34.03	16.62	39.04			30.27
HGBSP1-7	2.13	15.20	12.21	12.21	3.61	29.74			22.73
HGBSP1-9	1.96	15.07	12.07	12.07	3.43	29.61			22.57
HGBSP2-5	13.17	32.58	32.59	32.59	14.10	35.99	35.81	43.34	28.73
HGBSP2-7	0.19	10.91	10.78	10.78	1.11	26.68	21.50	29.13	14.85
HGBSP2-9	0.02	10.78	10.64	10.64	0.98	26.56	21.21	28.84	14.64
HGBSP3-5	13.17	24.11	24.59	24.59	13.13	31.29	35.04	42.72	22.15
HGBSP3-7	0.19	2.07	2.26	2.26	0.15	21.99	20.73	28.52	8.27
HGBSP3-9	0.02	1.97	2.20	2.20	0.06	21.87	20.45	28.23	8.08
AHGBSP1-5	15.10	28.26	25.27	25.27	16.60	39.03			32.11
AHGBSP1-7	2.13	15.06	12.06	12.06	3.61	29.73			22.72
AHGBSP1-9	1.96	14.85	11.85	11.85	3.43	29.61			22.57
AHGBSP2-5	13.15	23.96	23.82	23.82	14.08	35.98	35.79	43.32	30.88
AHGBSP2-7	0.19	10.77	10.67	10.67	1.11	26.68	21.50	29.13	21.49
AHGBSP2-9	0.02	10.56	10.55	10.55	0.98	26.56	21.21	28.84	21.35
AHGBSP3-5	13.15	15.05	15.22	15.22	13.12	31.28	35.02	41.10	24.31
AHGBSP3-7	0.19	1.94	2.16	2.16	0.15	21.99	20.73	26.92	14.99
AHGBSP3-9	0.02	1.75	2.12	2.12	0.06	21.87	20.45	26.63	22.79
6-311++G(3df,3pd)	11.11	59.29	59.07	59.07	58.77	71.42	1888.39	1819.55	65.19
def2-TZVP	14.31	282.55	307.06	307.06	160.12	374.99	2077.95	2147.44	542.07

Table S36: Mean absolute energy differences ΔE^{GTO} in mE_h for a variety of GTO basis sets for the Ar atom. States with all positive differences ΔE are shown in blue, and states that exhibit negative ΔE at one or more field strength(s) in red.

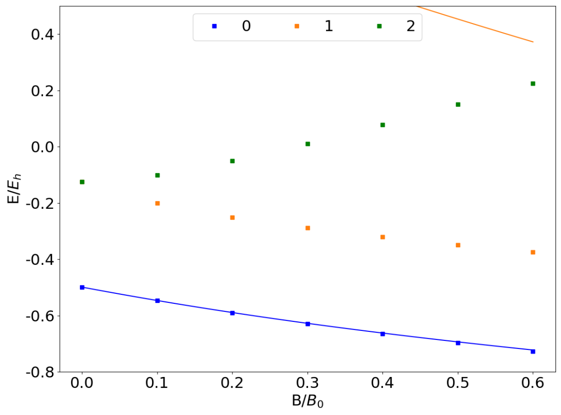

Figure S19: Total energies of all considered states of the H atom in the cc-pVDZ basis set in fully uncontracted form (solid lines). The FEM values are shown by the squares of the same color.

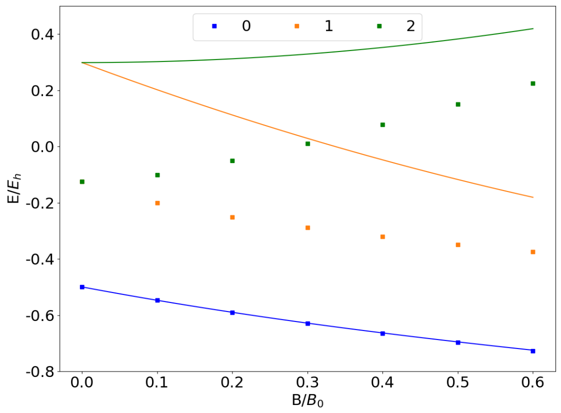

Figure S20: Total energies of all considered states of the H atom in the cc-pVTZ basis set in fully uncontracted form (solid lines). The FEM values are shown by the squares of the same color.

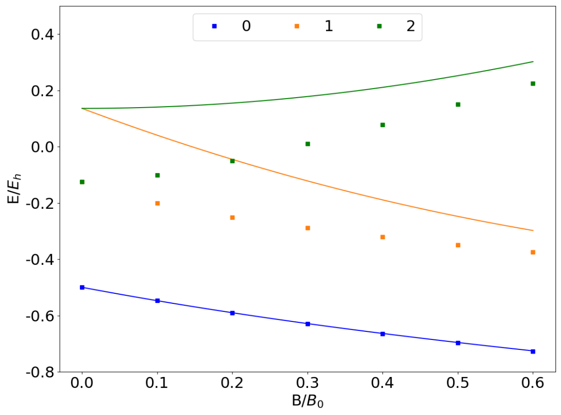

Figure S21: Total energies of all considered states of the H atom in the cc-pVQZ basis set in fully uncontracted form (solid lines). The FEM values are shown by the squares of the same color.

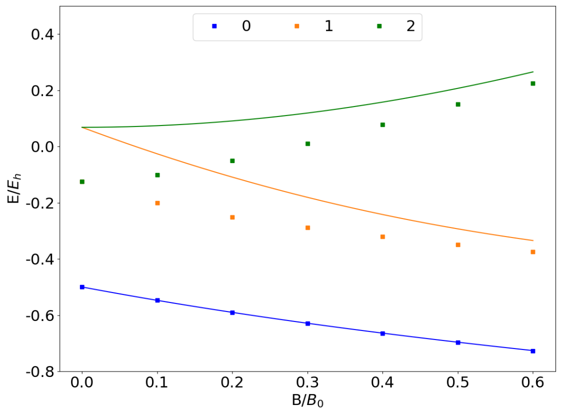

Figure S22: Total energies of all considered states of the H atom in the cc-pV5Z basis set in fully uncontracted form (solid lines). The FEM values are shown by the squares of the same color.

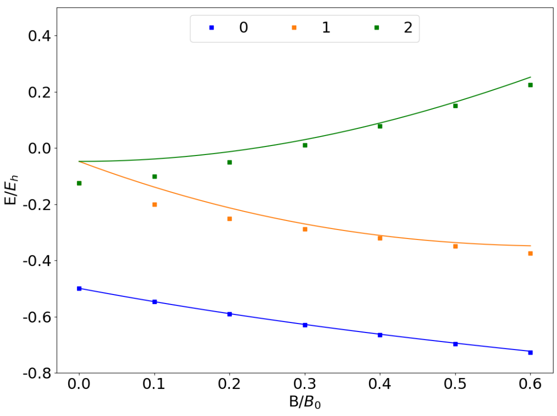

Figure S23: Total energies of all considered states of the H atom in the aug-cc-pVDZ basis set in fully uncontracted form (solid lines). The FEM values are shown by the squares of the same color.

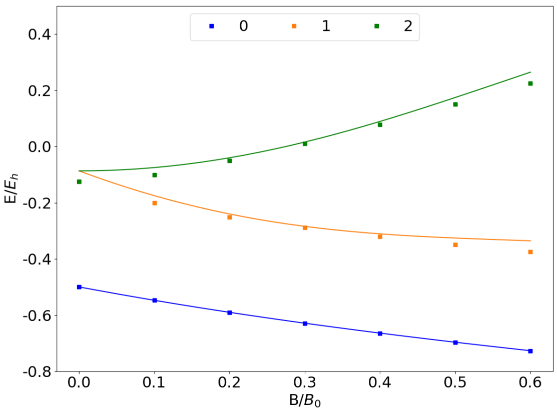

Figure S24: Total energies of all considered states of the H atom in the aug-cc-pVTZ basis set in fully uncontracted form (solid lines). The FEM values are shown by the squares of the same color.

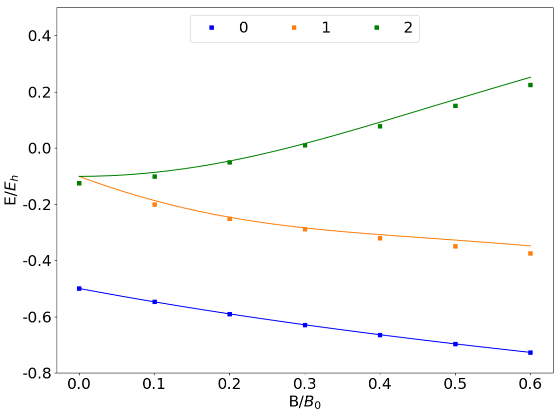

Figure S25: Total energies of all considered states of the H atom in the aug-cc-pVQZ basis set in fully uncontracted form (solid lines). The FEM values are shown by the squares of the same color.

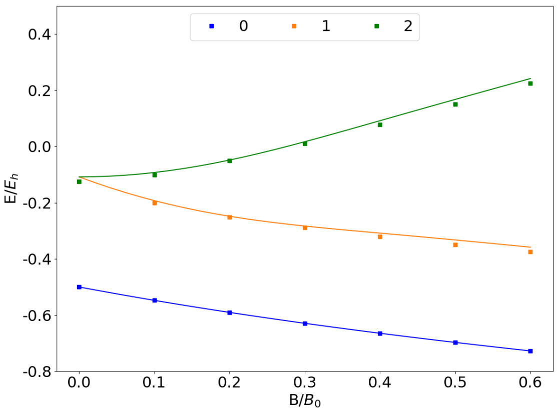

Figure S26: Total energies of all considered states of the H atom in the aug-cc-pV5Z basis set in fully uncontracted form (solid lines). The FEM values are shown by the squares of the same color.

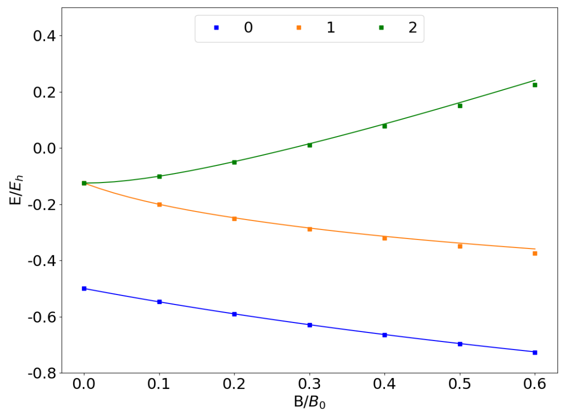

Figure S27: Total energies of all considered states of the H atom in the HGBSP1-5 basis set in fully uncontracted form (solid lines). The FEM values are shown by the squares of the same color.

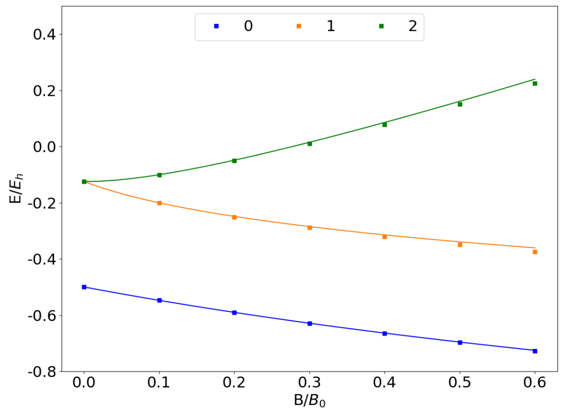

Figure S28: Total energies of all considered states of the H atom in the HGBSP1-7 basis set in fully uncontracted form (solid lines). The FEM values are shown by the squares of the same color.

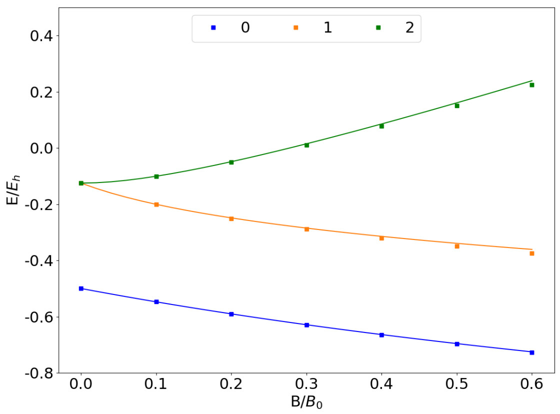

Figure S29: Total energies of all considered states of the H atom in the HGBSP1-9 basis set in fully uncontracted form (solid lines). The FEM values are shown by the squares of the same color.

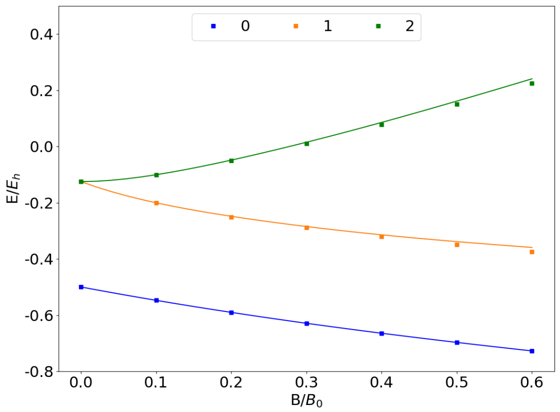

Figure S30: Total energies of all considered states of the H atom in the HGBSP2-5 basis set in fully uncontracted form (solid lines). The FEM values are shown by the squares of the same color.

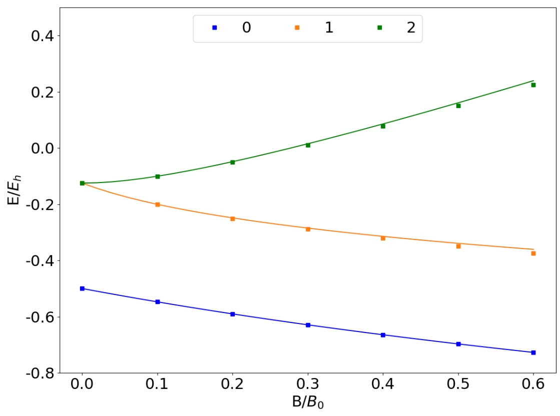

Figure S31: Total energies of all considered states of the H atom in the HGBSP2-7 basis set in fully uncontracted form (solid lines). The FEM values are shown by the squares of the same color.

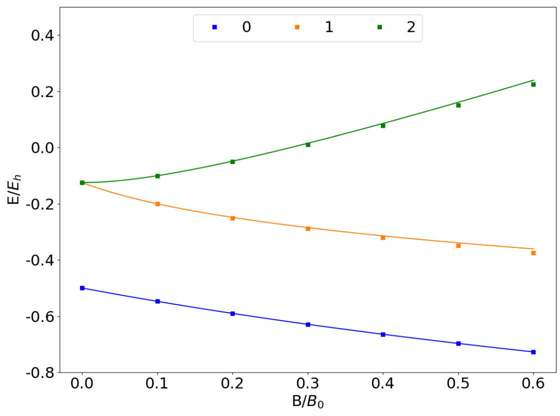

Figure S32: Total energies of all considered states of the H atom in the HGBSP2-9 basis set in fully uncontracted form (solid lines). The FEM values are shown by the squares of the same color.

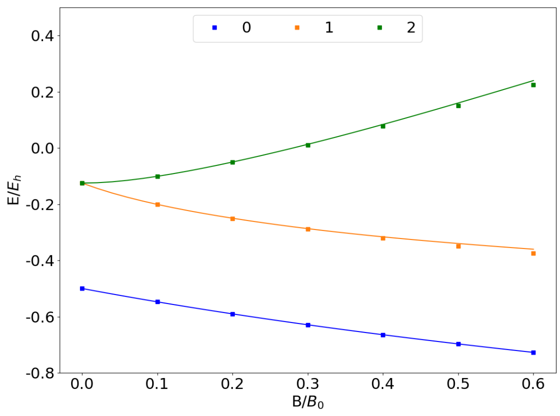

Figure S33: Total energies of all considered states of the H atom in the HGBSP3-5 basis set in fully uncontracted form (solid lines). The FEM values are shown by the squares of the same color.

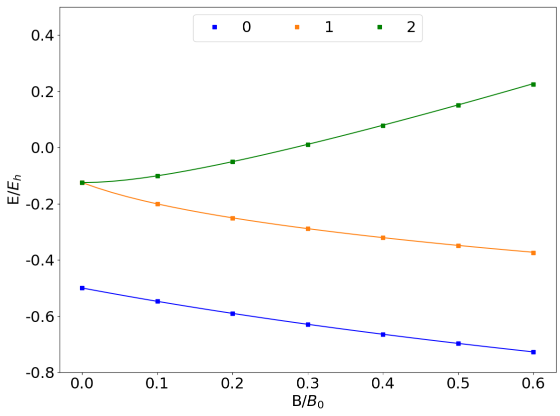

Figure S34: Total energies of all considered states of the H atom in the HGBSP3-7 basis set in fully uncontracted form (solid lines). The FEM values are shown by the squares of the same color.

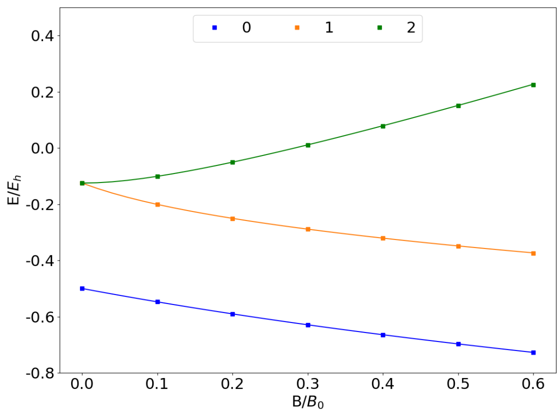

Figure S35: Total energies of all considered states of the H atom in the HGBSP3-9 basis set in fully uncontracted form (solid lines). The FEM values are shown by the squares of the same color.

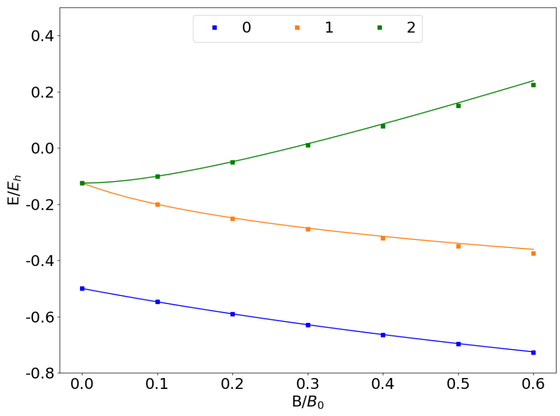

Figure S37: Total energies of all considered states of the H atom in the AHGBSP1-7 basis set in fully uncontracted form (solid lines). The FEM values are shown by the squares of the same color.

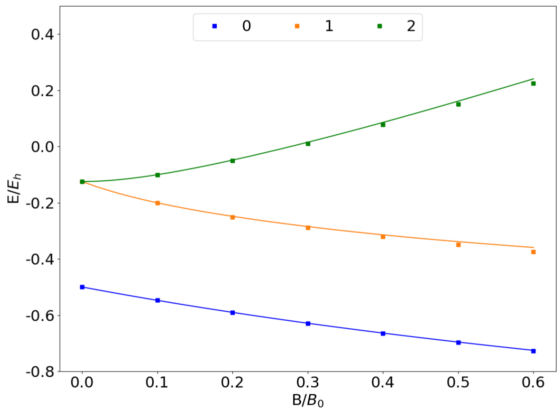

Figure S36: Total energies of all considered states of the H atom in the AHGBSP1-5 basis set in fully uncontracted form (solid lines). The FEM values are shown by the squares of the same color.

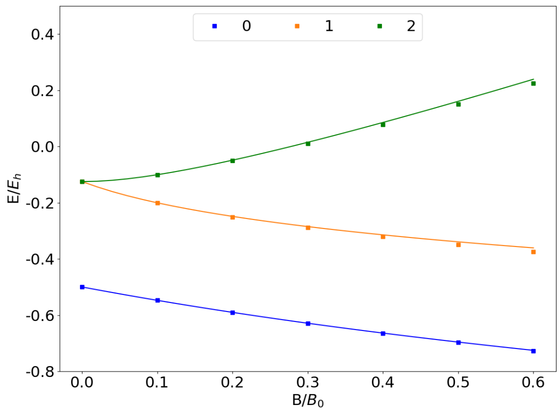

Figure S38: Total energies of all considered states of the H atom in the AHGBSP1-9 basis set in fully uncontracted form (solid lines). The FEM values are shown by the squares of the same color.

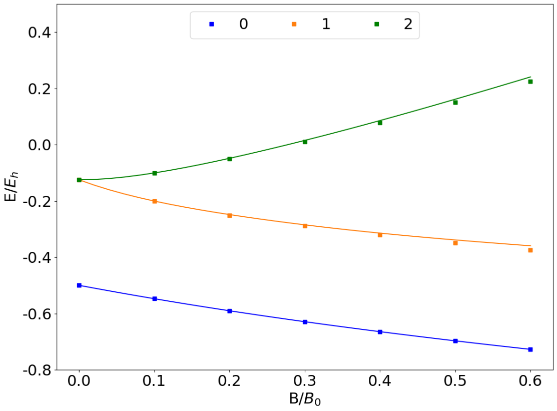

Figure S39: Total energies of all considered states of the H atom in the AHGBSP2-5 basis set in fully uncontracted form (solid lines). The FEM values are shown by the squares of the same color.

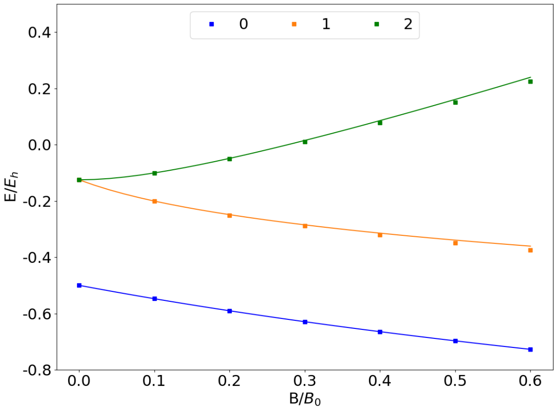

Figure S40: Total energies of all considered states of the H atom in the AHGBSP2-7 basis set in fully uncontracted form (solid lines). The FEM values are shown by the squares of the same color.

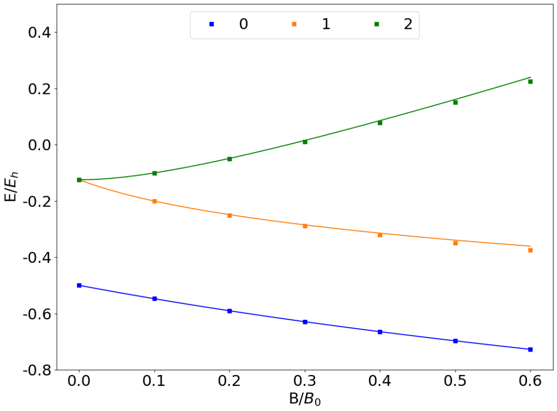

Figure S41: Total energies of all considered states of the H atom in the AHGBSP2-9 basis set in fully uncontracted form (solid lines). The FEM values are shown by the squares of the same color.

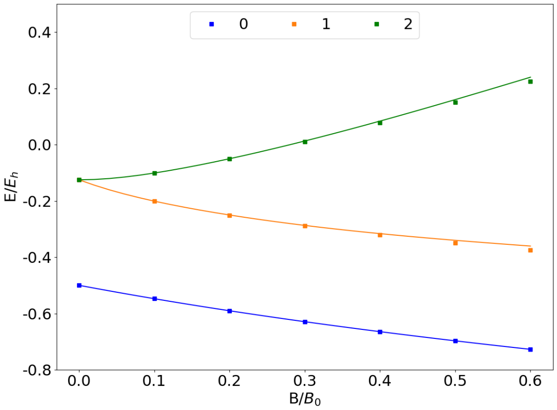

Figure S42: Total energies of all considered states of the H atom in the AHGBSP3-5 basis set in fully uncontracted form (solid lines). The FEM values are shown by the squares of the same color.

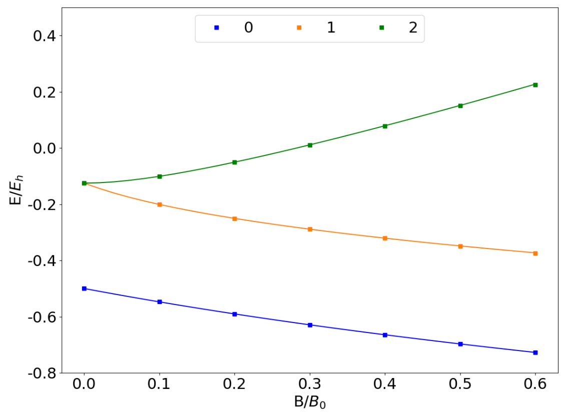

Figure S43: Total energies of all considered states of the H atom in the AHGBSP3-7 basis set in fully uncontracted form (solid lines). The FEM values are shown by the squares of the same color.

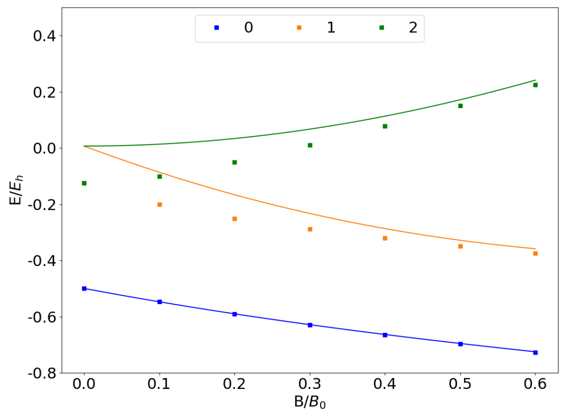

Figure S45: Total energies of all considered states of the H atom in the 6-311++G(3df,3pd) basis set in fully uncontracted form (solid lines). The FEM values are shown by the squares of the same color.

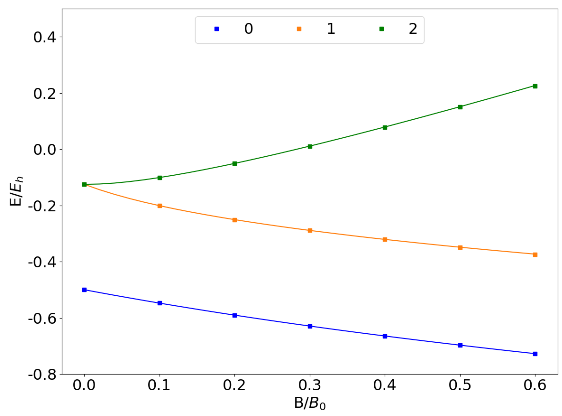

Figure S44: Total energies of all considered states of the H atom in the AHGBSP3-9 basis set in fully uncontracted form (solid lines). The FEM values are shown by the squares of the same color.

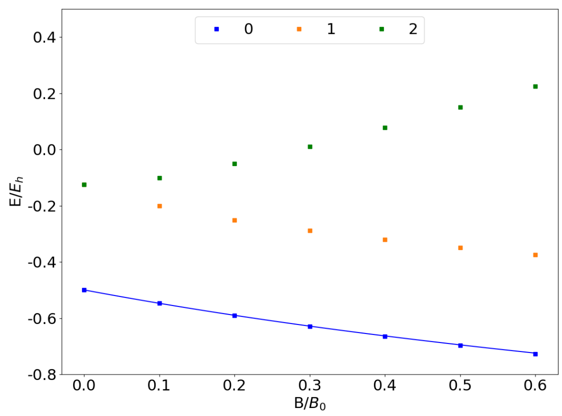

Figure S46: Total energies of all considered states of the H atom in the def2-TZVP basis set in fully uncontracted form (solid lines). The FEM values are shown by the squares of the same color.

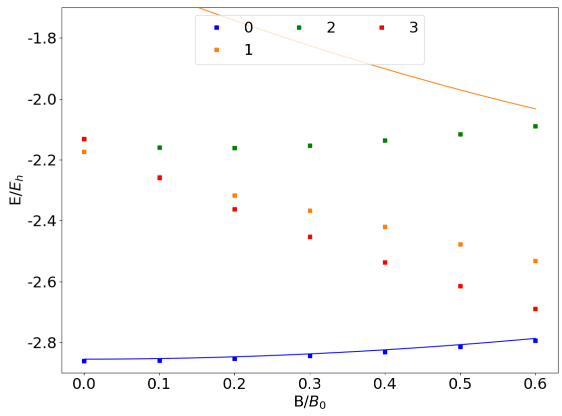

Figure S47: Total energies of all considered states of the He atom in the cc-pVDZ basis set in fully uncontracted form (solid lines). The FEM values are shown by the squares of the same color.

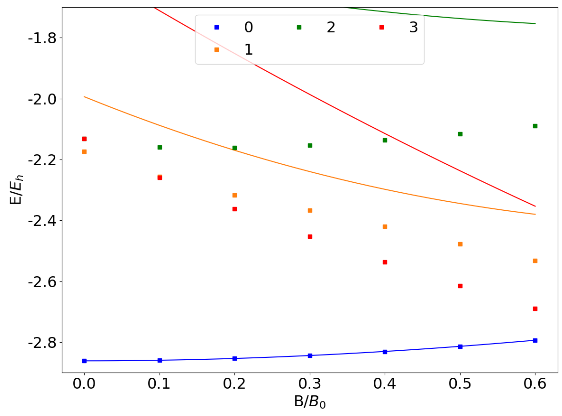

Figure S49: Total energies of all considered states of the He atom in the cc-pVQZ basis set in fully uncontracted form (solid lines). The FEM values are shown by the squares of the same color.

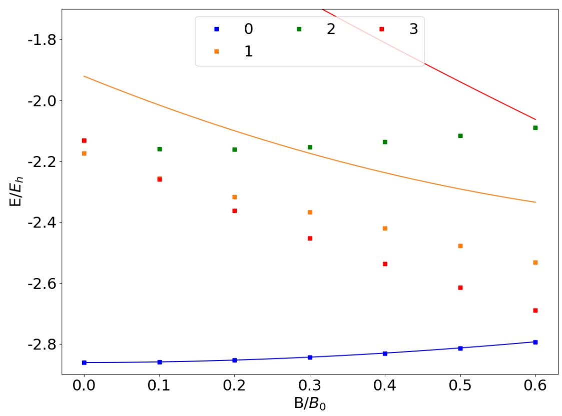

Figure S48: Total energies of all considered states of the He atom in the cc-pVTZ basis set in fully uncontracted form (solid lines). The FEM values are shown by the squares of the same color.

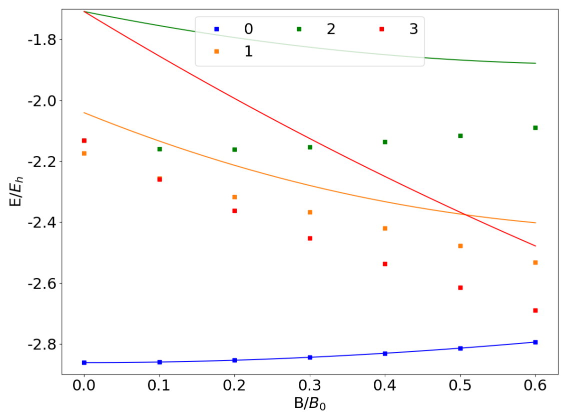

Figure S50: Total energies of all considered states of the He atom in the cc-pV5Z basis set in fully uncontracted form (solid lines). The FEM values are shown by the squares of the same color.

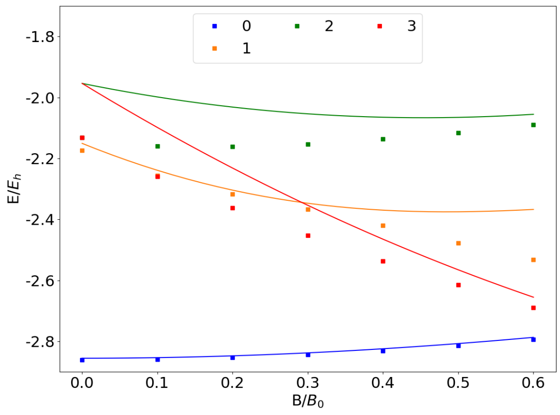

Figure S51: Total energies of all considered states of the He atom in the aug-cc-pVDZ basis set in fully uncontracted form (solid lines). The FEM values are shown by the squares of the same color.

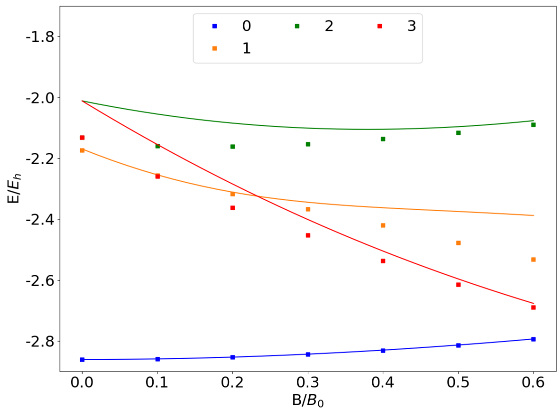

Figure S52: Total energies of all considered states of the He atom in the aug-cc-pVTZ basis set in fully uncontracted form (solid lines). The FEM values are shown by the squares of the same color.

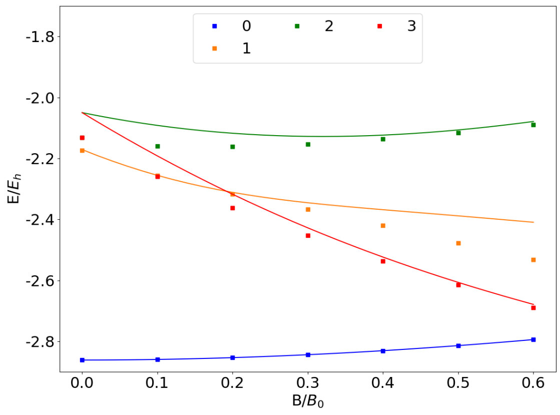

Figure S53: Total energies of all considered states of the He atom in the aug-cc-pVQZ basis set in fully uncontracted form (solid lines). The FEM values are shown by the squares of the same color.

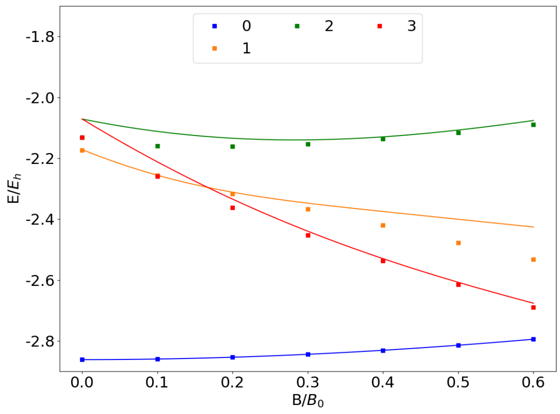

Figure S54: Total energies of all considered states of the He atom in the aug-cc-pV5Z basis set in fully uncontracted form (solid lines). The FEM values are shown by the squares of the same color.

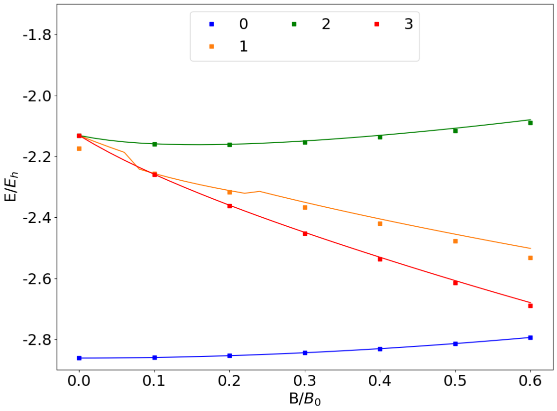

Figure S55: Total energies of all considered states of the He atom in the HGBSP1-5 basis set in fully uncontracted form (solid lines). The FEM values are shown by the squares of the same color.

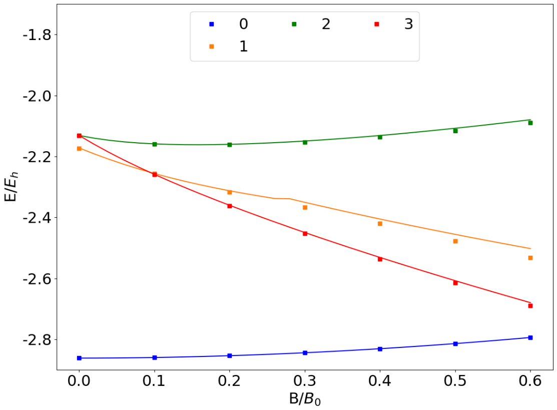

Figure S56: Total energies of all considered states of the He atom in the HGBSP1-7 basis set in fully uncontracted form (solid lines). The FEM values are shown by the squares of the same color.

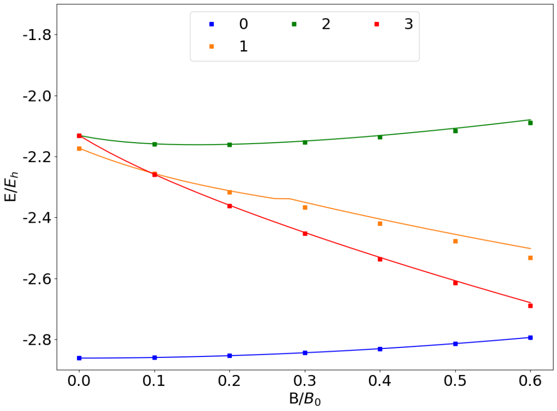

Figure S57: Total energies of all considered states of the He atom in the HGBSP1-9 basis set in fully uncontracted form (solid lines). The FEM values are shown by the squares of the same color.

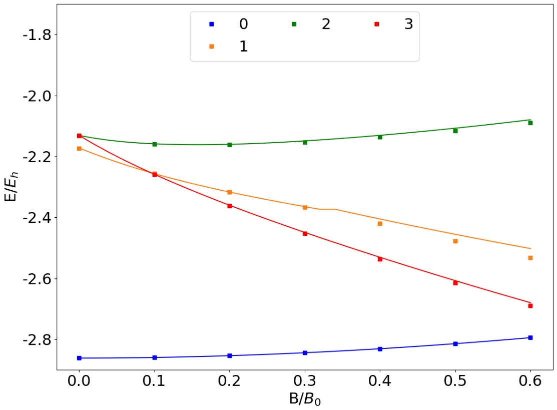

Figure S58: Total energies of all considered states of the He atom in the HGBSP2-5 basis set in fully uncontracted form (solid lines). The FEM values are shown by the squares of the same color.

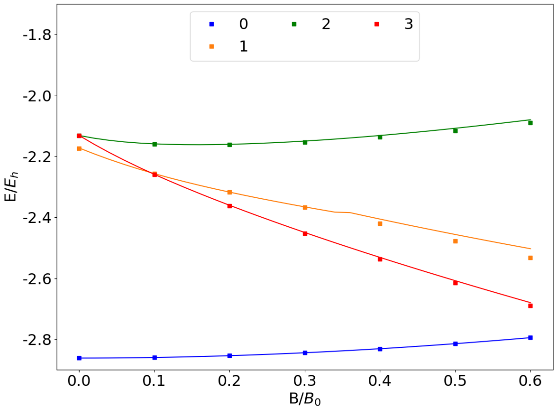

Figure S59: Total energies of all considered states of the He atom in the HGBSP2-7 basis set in fully uncontracted form (solid lines). The FEM values are shown by the squares of the same color.

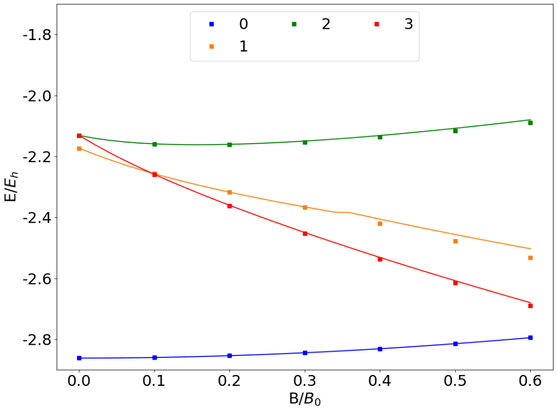

Figure S60: Total energies of all considered states of the He atom in the HGBSP2-9 basis set in fully uncontracted form (solid lines). The FEM values are shown by the squares of the same color.

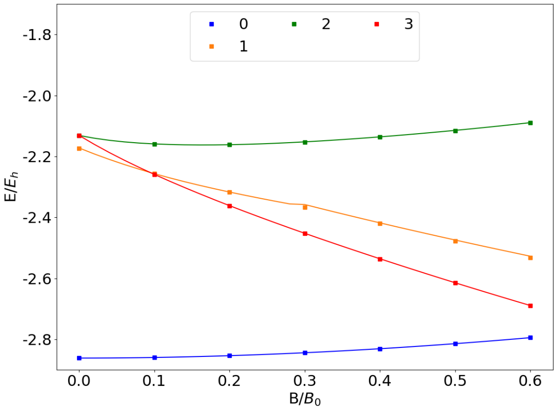

Figure S61: Total energies of all considered states of the He atom in the HGBSP3-5 basis set in fully uncontracted form (solid lines). The FEM values are shown by the squares of the same color.

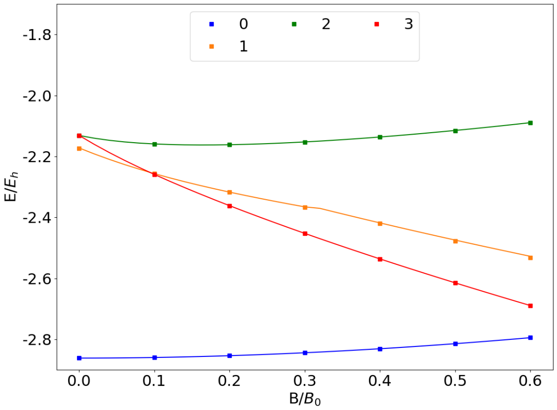

Figure S62: Total energies of all considered states of the He atom in the HGBSP3-7 basis set in fully uncontracted form (solid lines). The FEM values are shown by the squares of the same color.

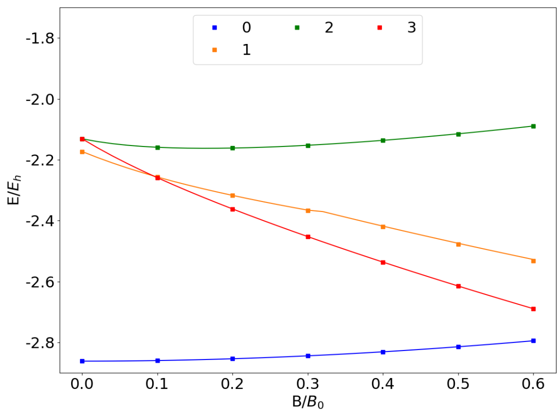

Figure S63: Total energies of all considered states of the He atom in the HGBSP3-9 basis set in fully uncontracted form (solid lines). The FEM values are shown by the squares of the same color.

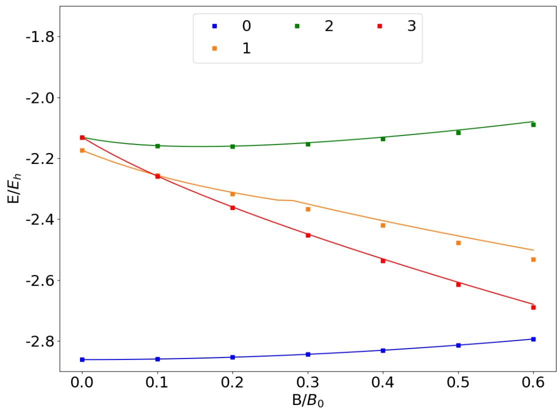

Figure S64: Total energies of all considered states of the He atom in the AHGBSP1-5 basis set in fully uncontracted form (solid lines). The FEM values are shown by the squares of the same color.

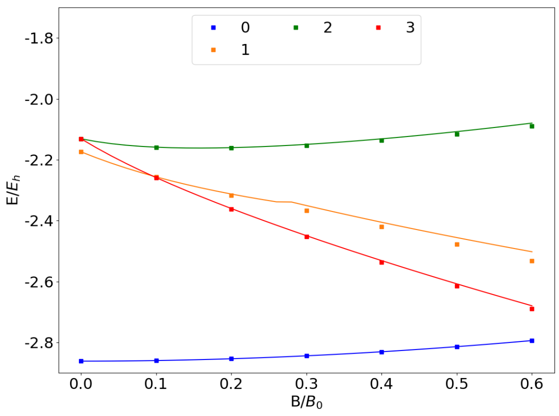

Figure S65: Total energies of all considered states of the He atom in the AHGBSP1-7 basis set in fully uncontracted form (solid lines). The FEM values are shown by the squares of the same color.

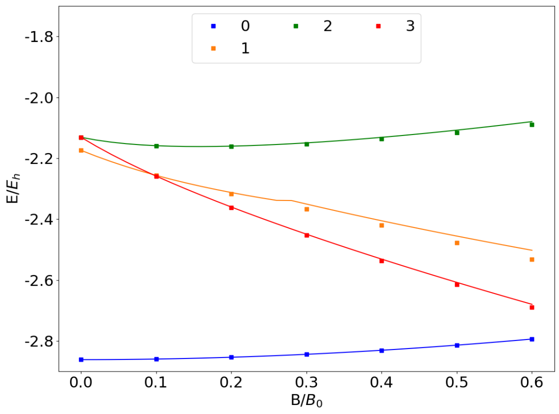

Figure S66: Total energies of all considered states of the He atom in the AHGBSP1-9 basis set in fully uncontracted form (solid lines). The FEM values are shown by the squares of the same color.

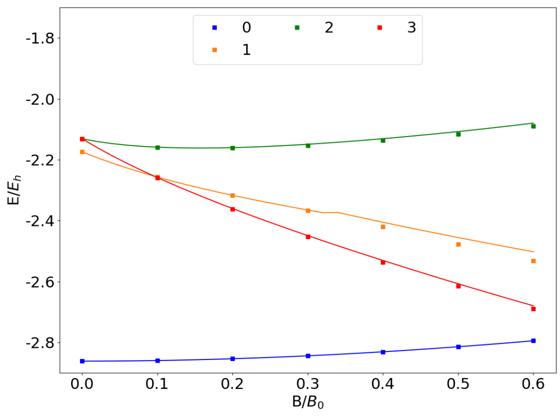

Figure S67: Total energies of all considered states of the He atom in the AHGBSP2-5 basis set in fully uncontracted form (solid lines). The FEM values are shown by the squares of the same color.

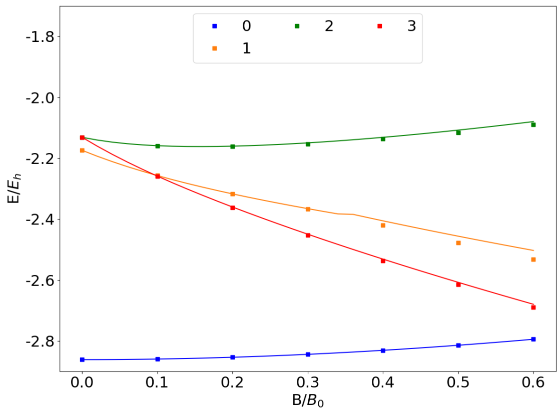

Figure S68: Total energies of all considered states of the He atom in the AHGBSP2-7 basis set in fully uncontracted form (solid lines). The FEM values are shown by the squares of the same color.

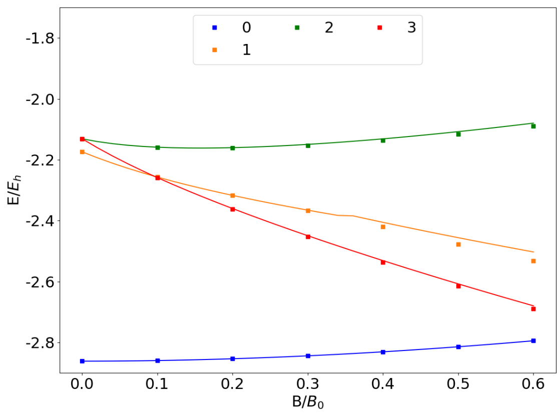

Figure S69: Total energies of all considered states of the He atom in the AHGBSP2-9 basis set in fully uncontracted form (solid lines). The FEM values are shown by the squares of the same color.

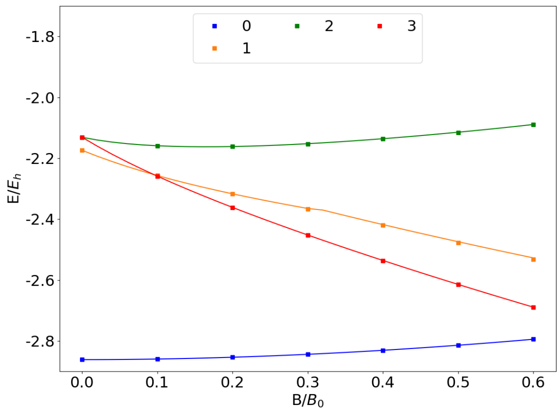

Figure S70: Total energies of all considered states of the He atom in the AHGBSP3-5 basis set in fully uncontracted form (solid lines). The FEM values are shown by the squares of the same color.

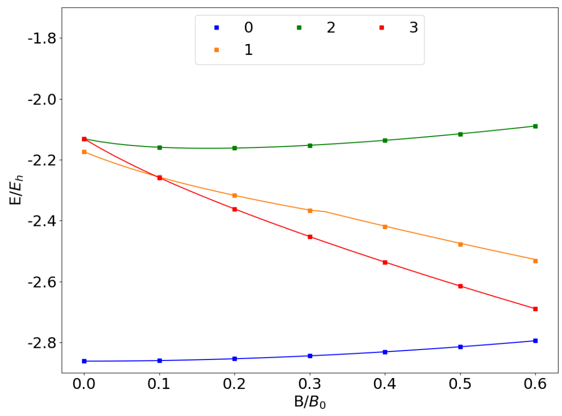

Figure S71: Total energies of all considered states of the He atom in the AHGBSP3-7 basis set in fully uncontracted form (solid lines). The FEM values are shown by the squares of the same color.

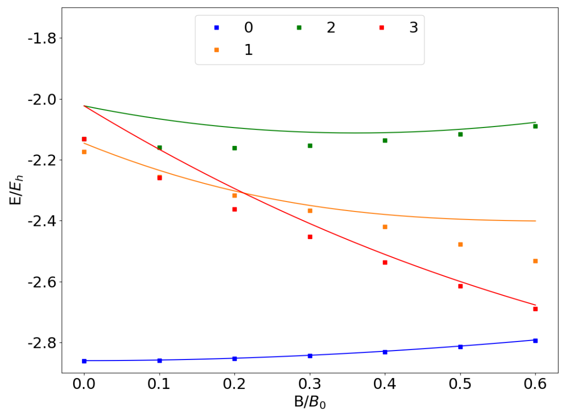

Figure S73: Total energies of all considered states of the He atom in the 6-311++G(3df,3pd) basis set in fully uncontracted form (solid lines). The FEM values are shown by the squares of the same color.

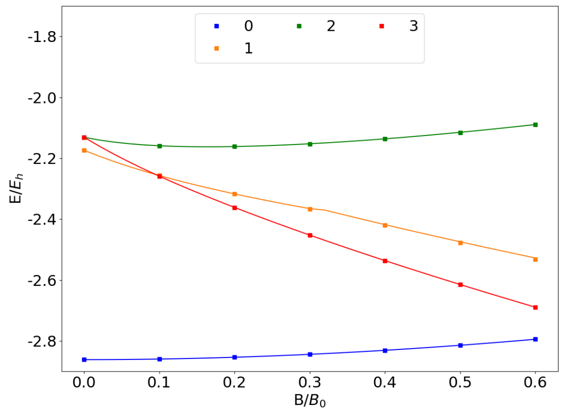

Figure S72: Total energies of all considered states of the He atom in the AHGBSP3-9 basis set in fully uncontracted form (solid lines). The FEM values are shown by the squares of the same color.

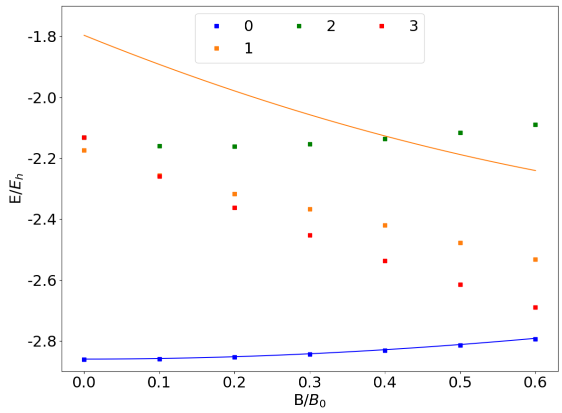

Figure S74: Total energies of all considered states of the He atom in the def2-TZVP basis set in fully uncontracted form (solid lines). The FEM values are shown by the squares of the same color.

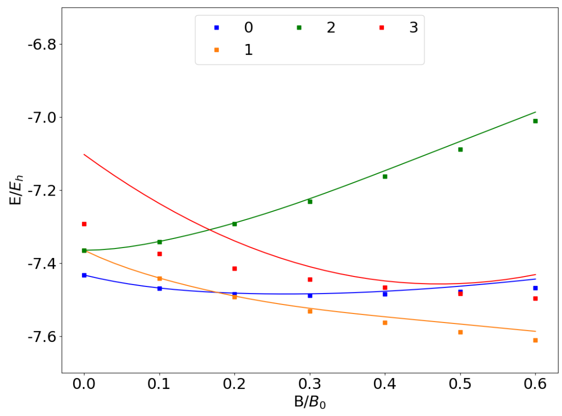

Figure S75: Total energies of all considered states of the Li atom in the cc-pVDZ basis set in fully uncontracted form (solid lines). The FEM values are shown by the squares of the same color.

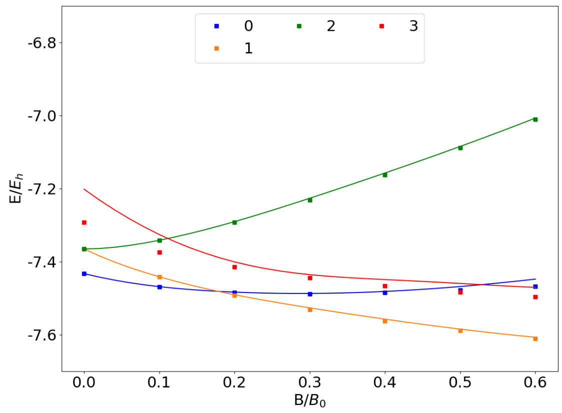

Figure S76: Total energies of all considered states of the Li atom in the cc-pVTZ basis set in fully uncontracted form (solid lines). The FEM values are shown by the squares of the same color.

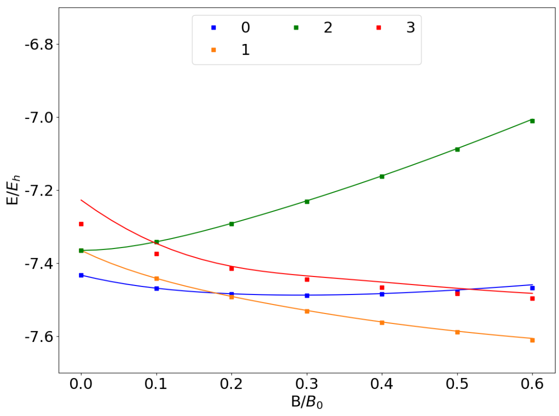

Figure S77: Total energies of all considered states of the Li atom in the cc-pVQZ basis set in fully uncontracted form (solid lines). The FEM values are shown by the squares of the same color.

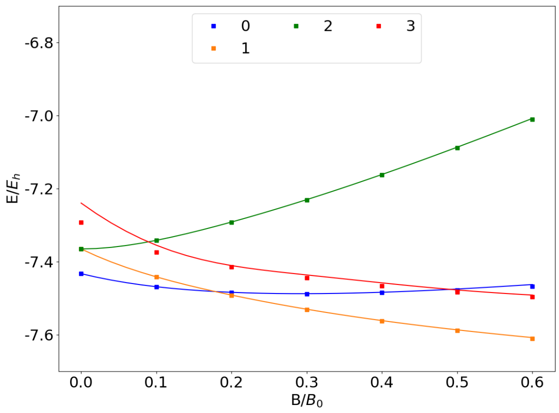

Figure S78: Total energies of all considered states of the Li atom in the cc-pV5Z basis set in fully uncontracted form (solid lines). The FEM values are shown by the squares of the same color.

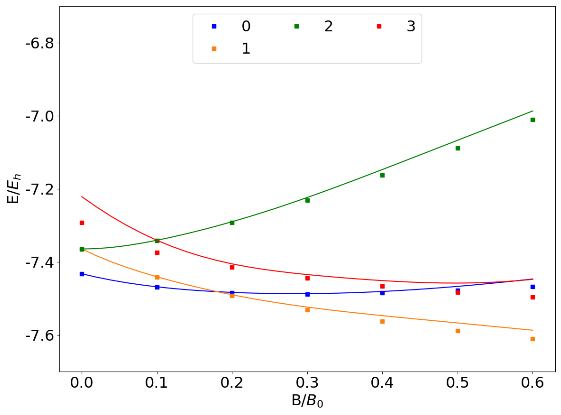

Figure S79: Total energies of all considered states of the Li atom in the aug-cc-pVDZ basis set in fully uncontracted form (solid lines). The FEM values are shown by the squares of the same color.

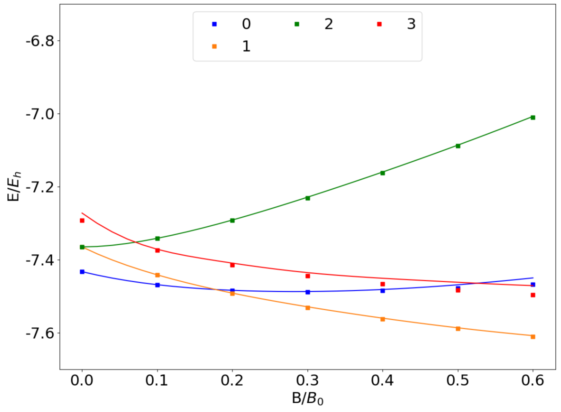

Figure S80: Total energies of all considered states of the Li atom in the aug-cc-pVTZ basis set in fully uncontracted form (solid lines). The FEM values are shown by the squares of the same color.

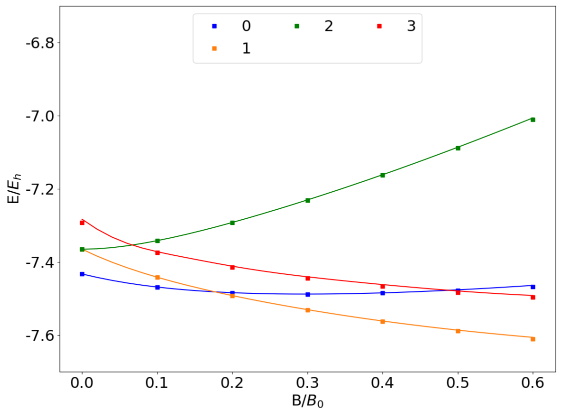

Figure S81: Total energies of all considered states of the Li atom in the aug-cc-pVQZ basis set in fully uncontracted form (solid lines). The FEM values are shown by the squares of the same color.

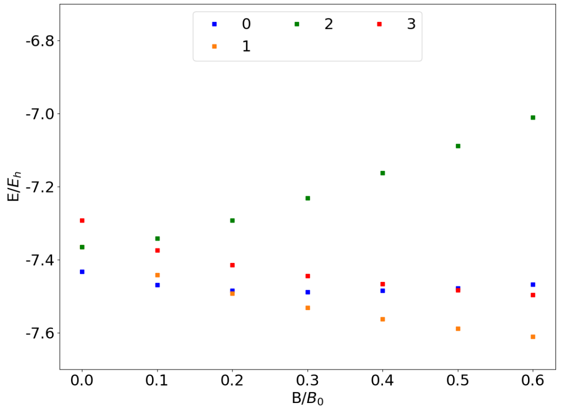

Figure S82: Total energies of all considered states of the Li atom in the aug-cc-pV5Z basis set in fully uncontracted form (solid lines). The FEM values are shown by the squares of the same color.

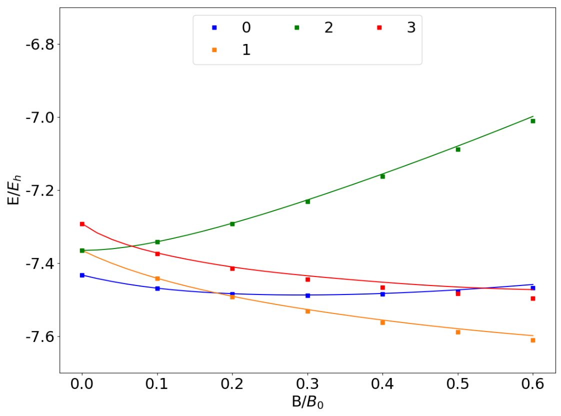

Figure S83: Total energies of all considered states of the Li atom in the HGBSP1-5 basis set in fully uncontracted form (solid lines). The FEM values are shown by the squares of the same color.

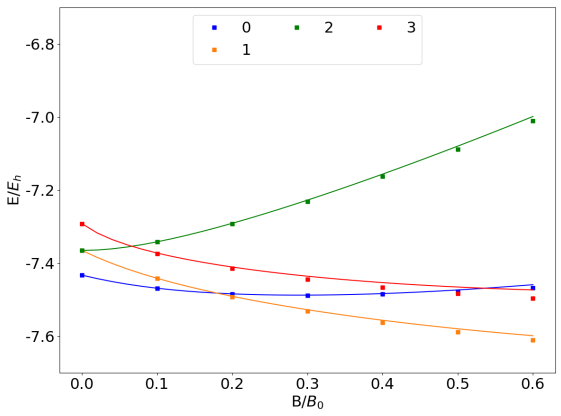

Figure S85: Total energies of all considered states of the Li atom in the HGBSP1-9 basis set in fully uncontracted form (solid lines). The FEM values are shown by the squares of the same color.

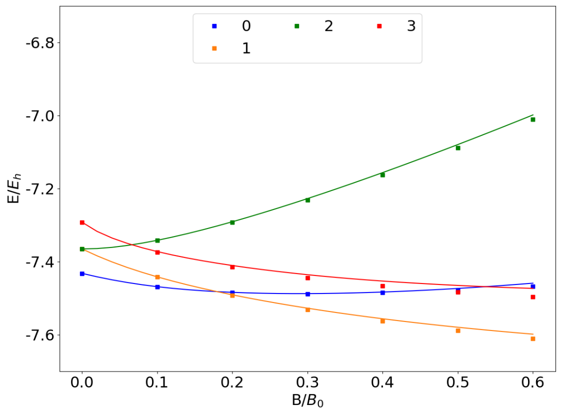

Figure S84: Total energies of all considered states of the Li atom in the HGBSP1-7 basis set in fully uncontracted form (solid lines). The FEM values are shown by the squares of the same color.

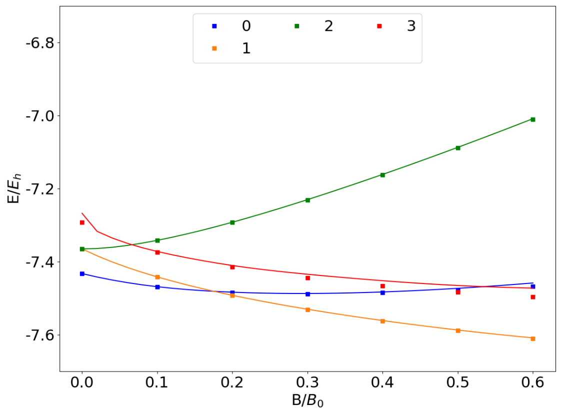

Figure S86: Total energies of all considered states of the Li atom in the HGBSP2-5 basis set in fully uncontracted form (solid lines). The FEM values are shown by the squares of the same color.

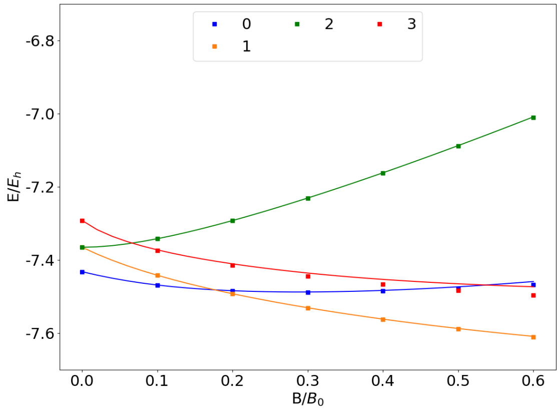

Figure S87: Total energies of all considered states of the Li atom in the HGBSP2-7 basis set in fully uncontracted form (solid lines). The FEM values are shown by the squares of the same color.

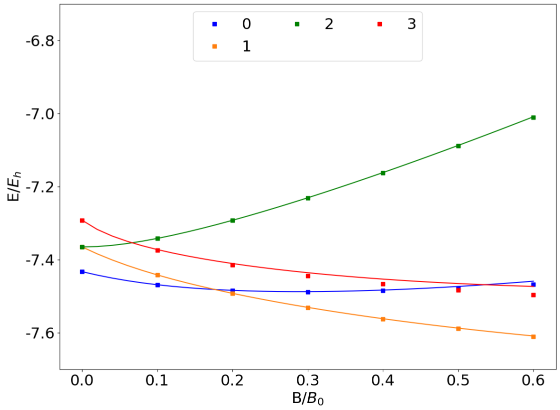

Figure S88: Total energies of all considered states of the Li atom in the HGBSP2-9 basis set in fully uncontracted form (solid lines). The FEM values are shown by the squares of the same color.

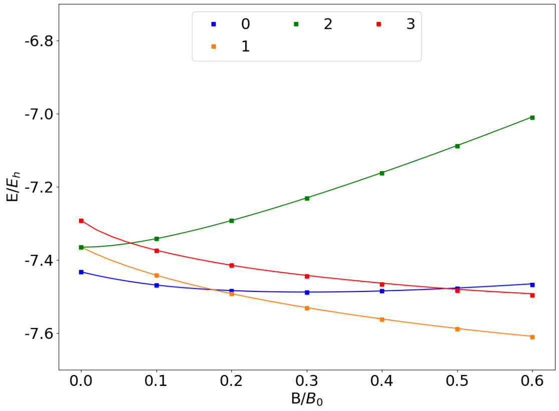

Figure S89: Total energies of all considered states of the Li atom in the HGBSP3-5 basis set in fully uncontracted form (solid lines). The FEM values are shown by the squares of the same color.

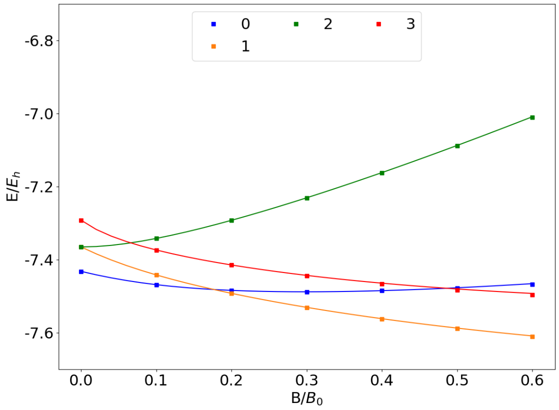

Figure S90: Total energies of all considered states of the Li atom in the HGBSP3-7 basis set in fully uncontracted form (solid lines). The FEM values are shown by the squares of the same color.

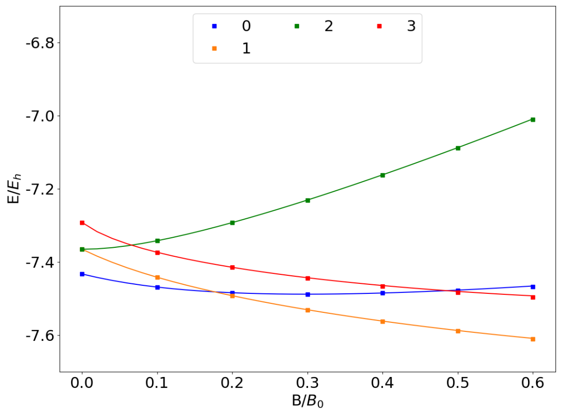

Figure S91: Total energies of all considered states of the Li atom in the HGBSP3-9 basis set in fully uncontracted form (solid lines). The FEM values are shown by the squares of the same color.

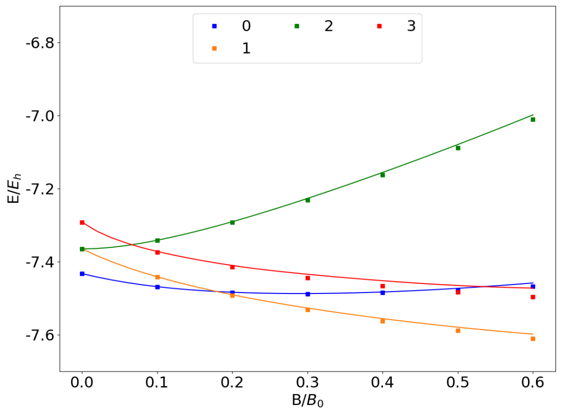

Figure S92: Total energies of all considered states of the Li atom in the AHGBSP1-5 basis set in fully uncontracted form (solid lines). The FEM values are shown by the squares of the same color.

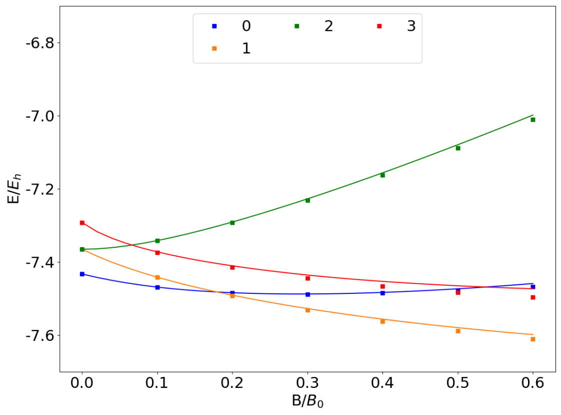

Figure S93: Total energies of all considered states of the Li atom in the AHGBSP1-7 basis set in fully uncontracted form (solid lines). The FEM values are shown by the squares of the same color.

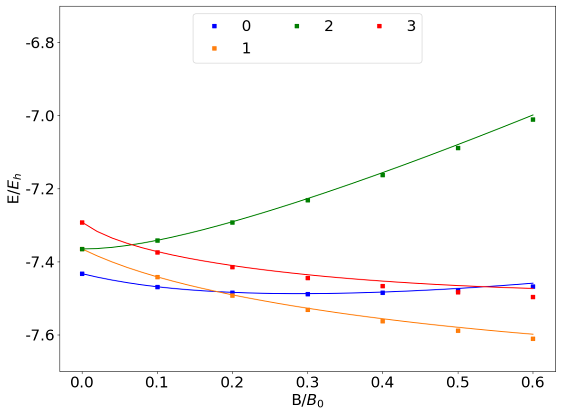

Figure S94: Total energies of all considered states of the Li atom in the AHGBSP1-9 basis set in fully uncontracted form (solid lines). The FEM values are shown by the squares of the same color.

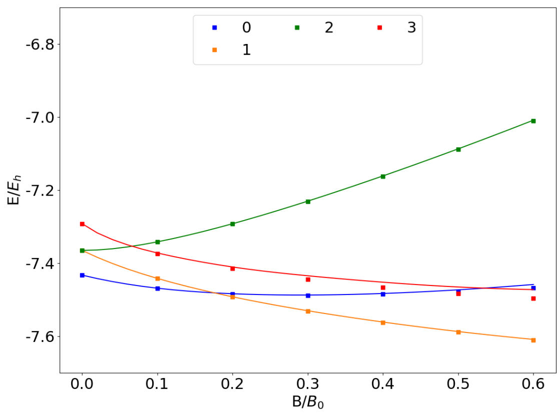

Figure S95: Total energies of all considered states of the Li atom in the AHGBSP2-5 basis set in fully uncontracted form (solid lines). The FEM values are shown by the squares of the same color.

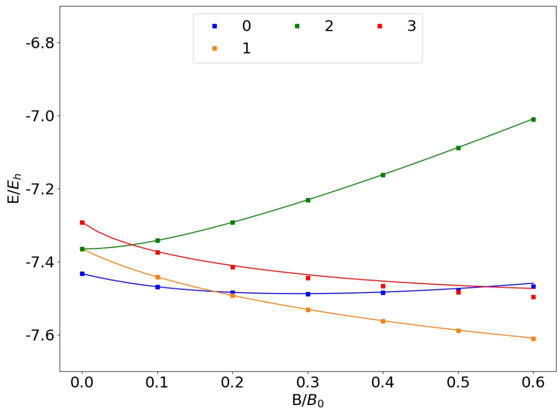

Figure S96: Total energies of all considered states of the Li atom in the AHGBSP2-7 basis set in fully uncontracted form (solid lines). The FEM values are shown by the squares of the same color.

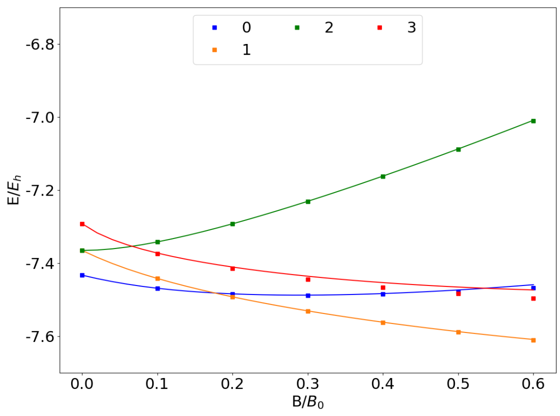

Figure S97: Total energies of all considered states of the Li atom in the AHGBSP2-9 basis set in fully uncontracted form (solid lines). The FEM values are shown by the squares of the same color.

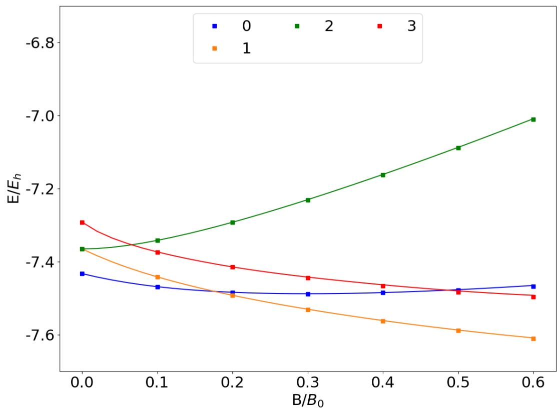

Figure S98: Total energies of all considered states of the Li atom in the AHGBSP3-5 basis set in fully uncontracted form (solid lines). The FEM values are shown by the squares of the same color.

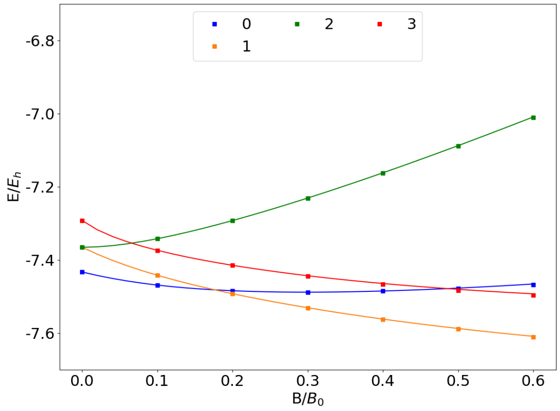

Figure S99: Total energies of all considered states of the Li atom in the AHGBSP3-7 basis set in fully uncontracted form (solid lines). The FEM values are shown by the squares of the same color.

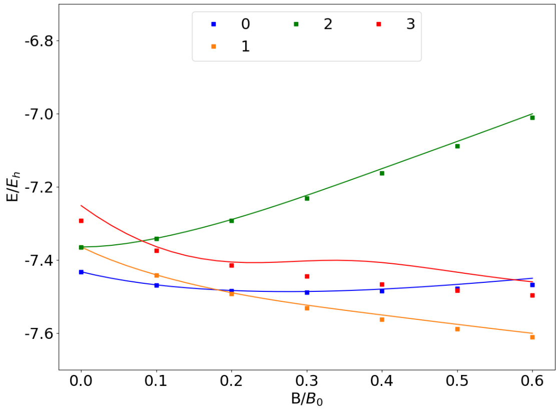

Figure S101: Total energies of all considered states of the Li atom in the 6-311++G(3df,3pd) basis set in fully uncontracted form (solid lines). The FEM values are shown by the squares of the same color.

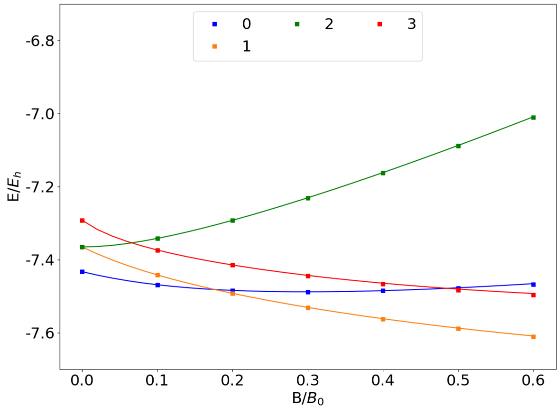

Figure S100: Total energies of all considered states of the Li atom in the AHGBSP3-9 basis set in fully uncontracted form (solid lines). The FEM values are shown by the squares of the same color.

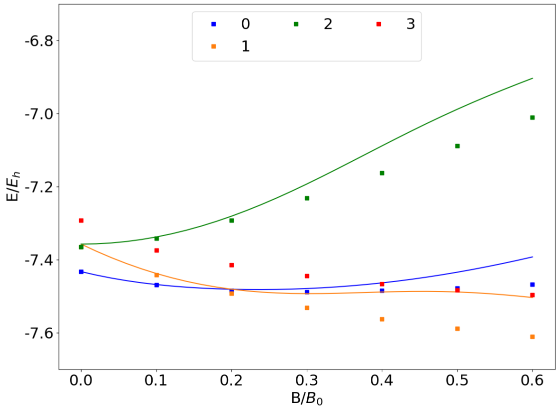

Figure S102: Total energies of all considered states of the Li atom in the def2-TZVP basis set in fully uncontracted form (solid lines). The FEM values are shown by the squares of the same color.

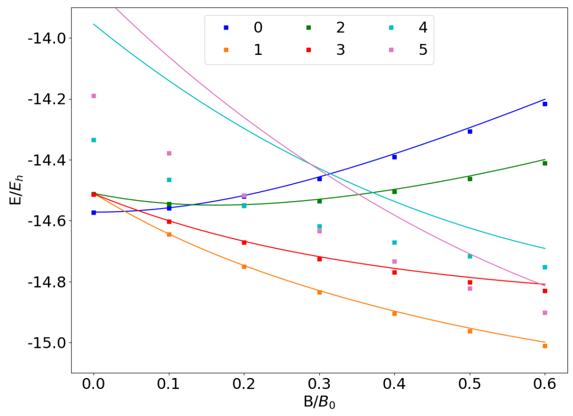

Figure S103: Total energies of all considered states of the Be atom in the cc-pVDZ basis set in fully uncontracted form (solid lines). The FEM values are shown by the squares of the same color.

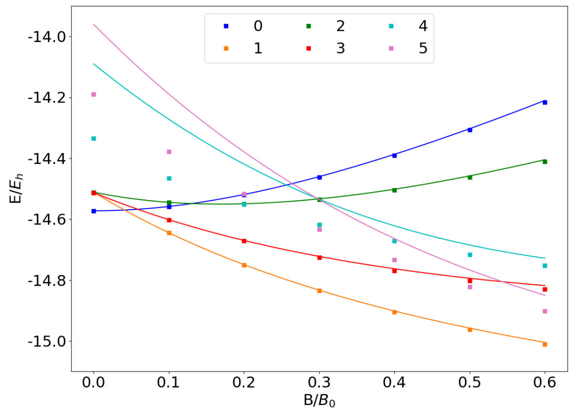

Figure S104: Total energies of all considered states of the Be atom in the cc-pVTZ basis set in fully uncontracted form (solid lines). The FEM values are shown by the squares of the same color.

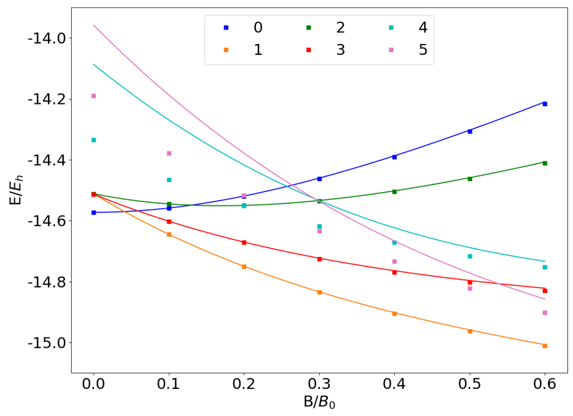

Figure S105: Total energies of all considered states of the Be atom in the cc-pVQZ basis set in fully uncontracted form (solid lines). The FEM values are shown by the squares of the same color.

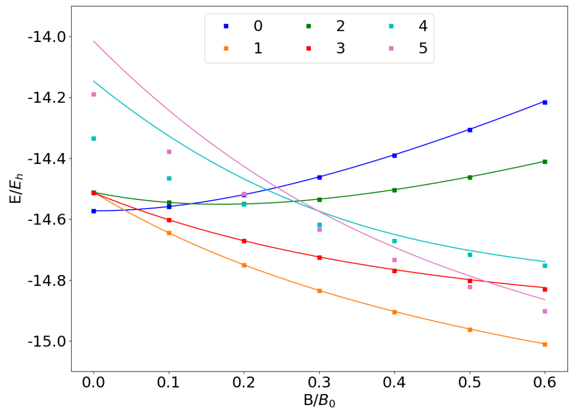

Figure S106: Total energies of all considered states of the Be atom in the cc-pV5Z basis set in fully uncontracted form (solid lines). The FEM values are shown by the squares of the same color.

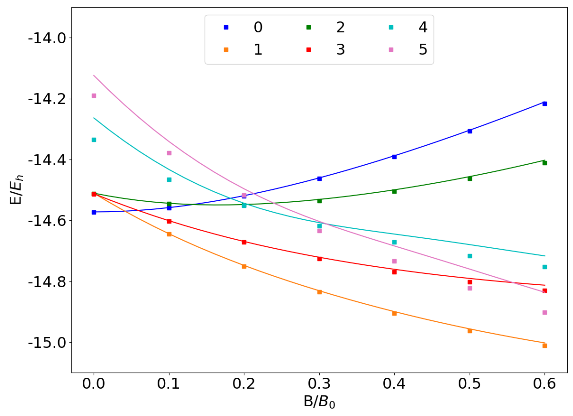

Figure S107: Total energies of all considered states of the Be atom in the aug-cc-pVDZ basis set in fully uncontracted form (solid lines). The FEM values are shown by the squares of the same color.

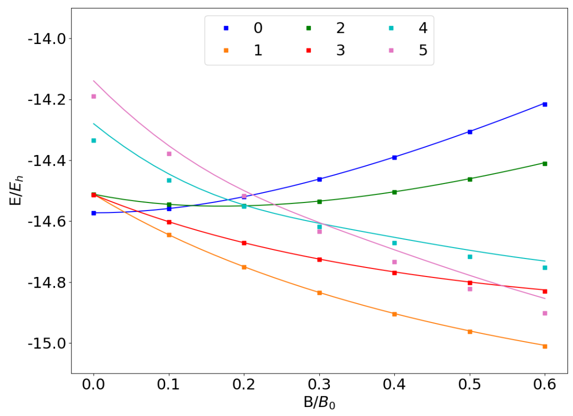

Figure S108: Total energies of all considered states of the Be atom in the aug-cc-pVTZ basis set in fully uncontracted form (solid lines). The FEM values are shown by the squares of the same color.

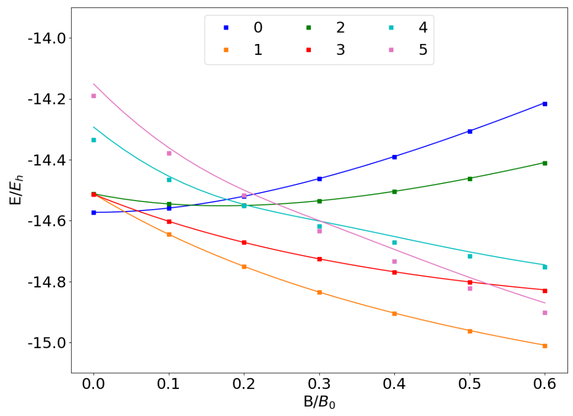

Figure S109: Total energies of all considered states of the Be atom in the aug-cc-pVQZ basis set in fully uncontracted form (solid lines). The FEM values are shown by the squares of the same color.

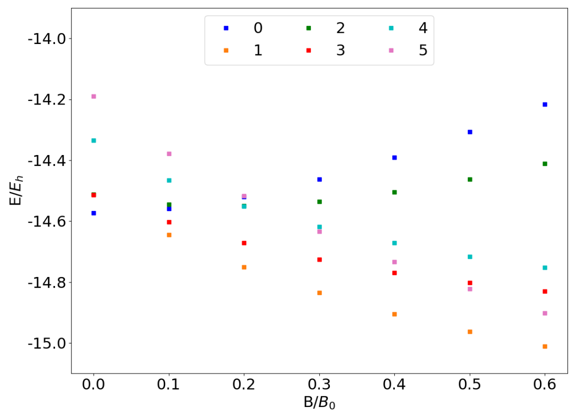

Figure S110: Total energies of all considered states of the Be atom in the aug-cc-pV5Z basis set in fully uncontracted form (solid lines). The FEM values are shown by the squares of the same color.

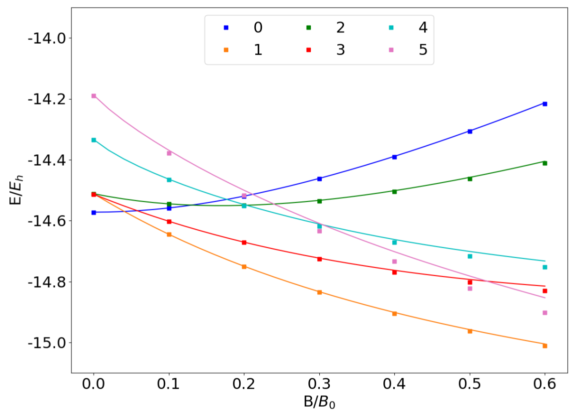

Figure S111: Total energies of all considered states of the Be atom in the HGBSP1-5 basis set in fully uncontracted form (solid lines). The FEM values are shown by the squares of the same color.

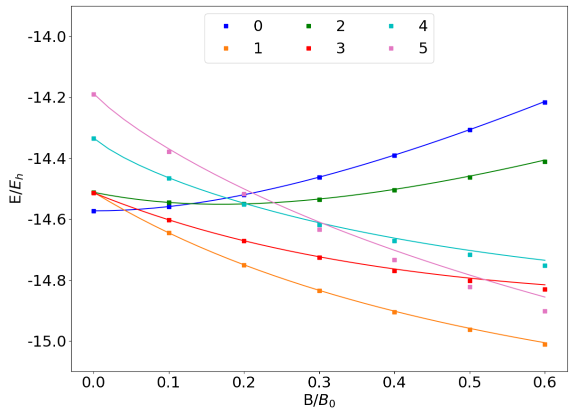

Figure S112: Total energies of all considered states of the Be atom in the HGBSP1-7 basis set in fully uncontracted form (solid lines). The FEM values are shown by the squares of the same color.

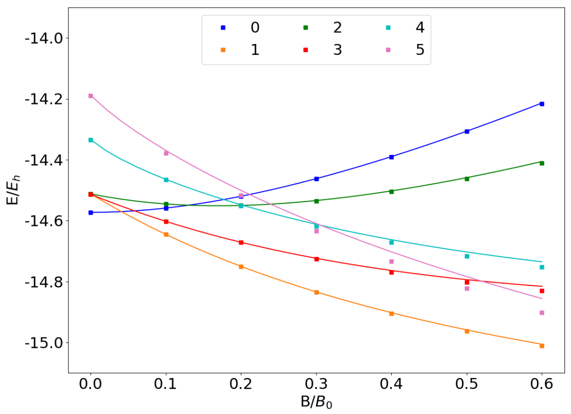

Figure S113: Total energies of all considered states of the Be atom in the HGBSP1-9 basis set in fully uncontracted form (solid lines). The FEM values are shown by the squares of the same color.

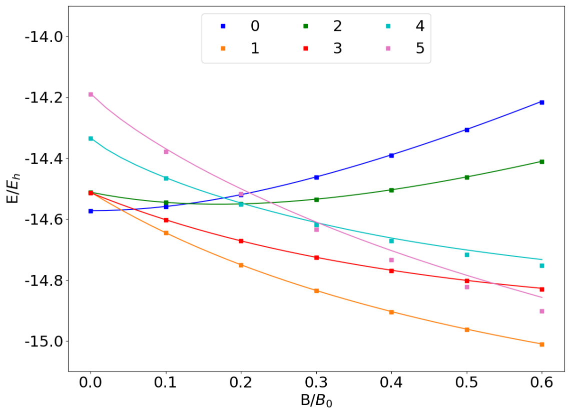

Figure S114: Total energies of all considered states of the Be atom in the HGBSP2-5 basis set in fully uncontracted form (solid lines). The FEM values are shown by the squares of the same color.

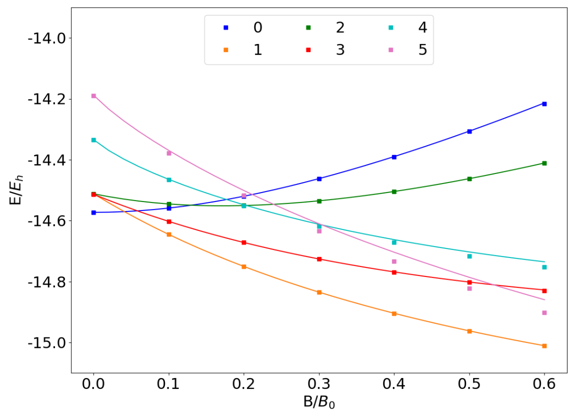

Figure S115: Total energies of all considered states of the Be atom in the HGBSP2-7 basis set in fully uncontracted form (solid lines). The FEM values are shown by the squares of the same color.

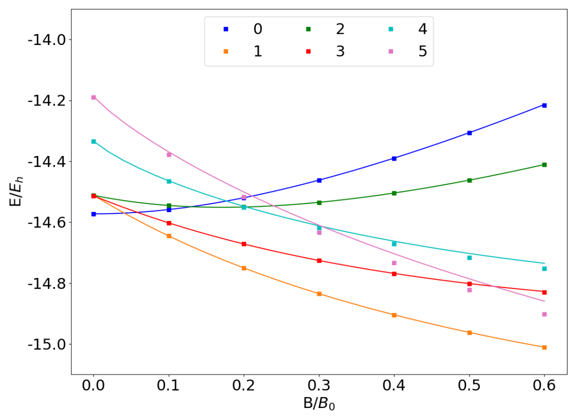

Figure S116: Total energies of all considered states of the Be atom in the HGBSP2-9 basis set in fully uncontracted form (solid lines). The FEM values are shown by the squares of the same color.

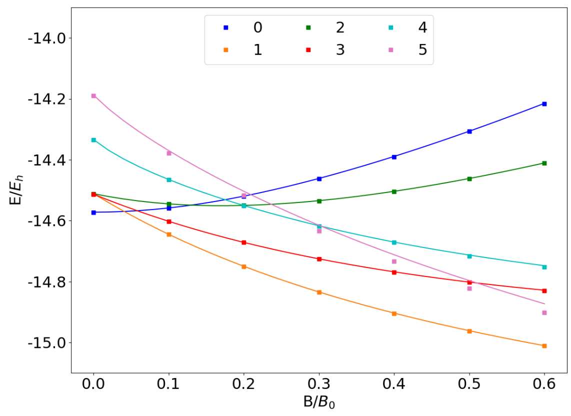

Figure S117: Total energies of all considered states of the Be atom in the HGBSP3-5 basis set in fully uncontracted form (solid lines). The FEM values are shown by the squares of the same color.

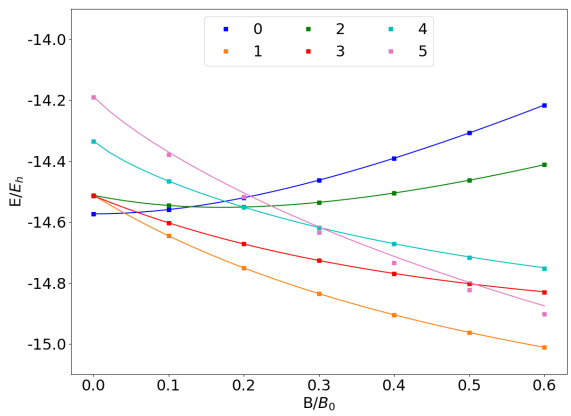

Figure S118: Total energies of all considered states of the Be atom in the HGBSP3-7 basis set in fully uncontracted form (solid lines). The FEM values are shown by the squares of the same color.

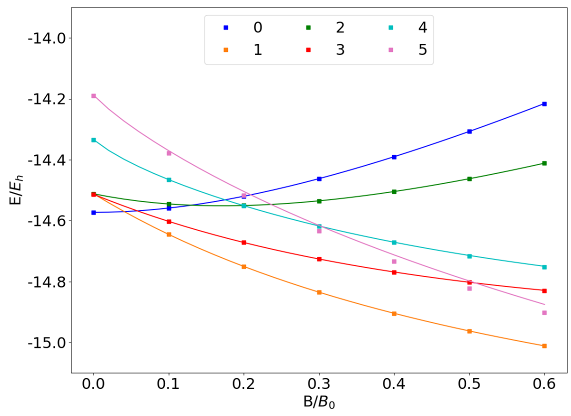

Figure S119: Total energies of all considered states of the Be atom in the HGBSP3-9 basis set in fully uncontracted form (solid lines). The FEM values are shown by the squares of the same color.

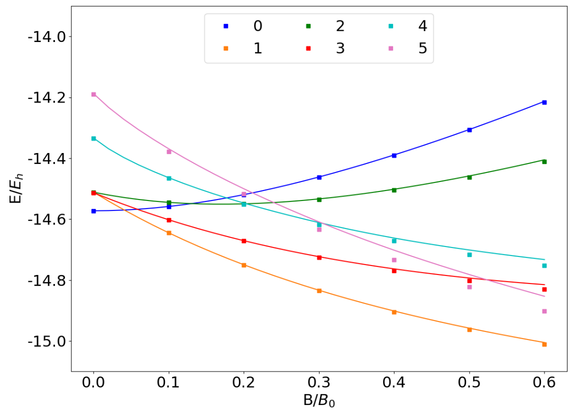

Figure S120: Total energies of all considered states of the Be atom in the AHGBSP1-5 basis set in fully uncontracted form (solid lines). The FEM values are shown by the squares of the same color.

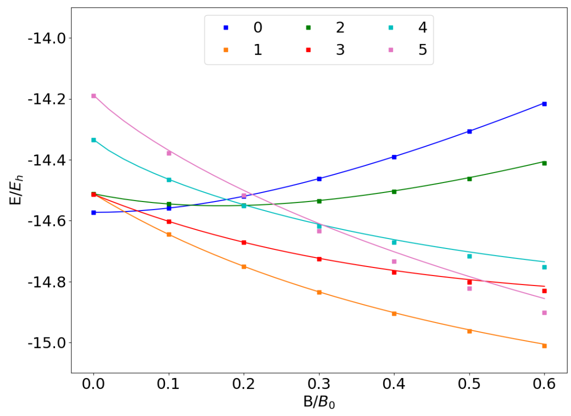

Figure S121: Total energies of all considered states of the Be atom in the AHGBSP1-7 basis set in fully uncontracted form (solid lines). The FEM values are shown by the squares of the same color.

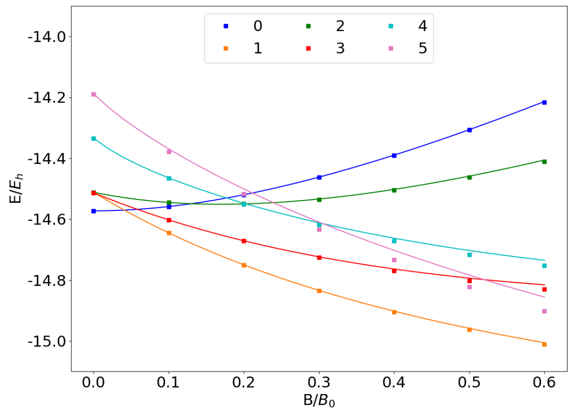

Figure S122: Total energies of all considered states of the Be atom in the AHGBSP1-9 basis set in fully uncontracted form (solid lines). The FEM values are shown by the squares of the same color.

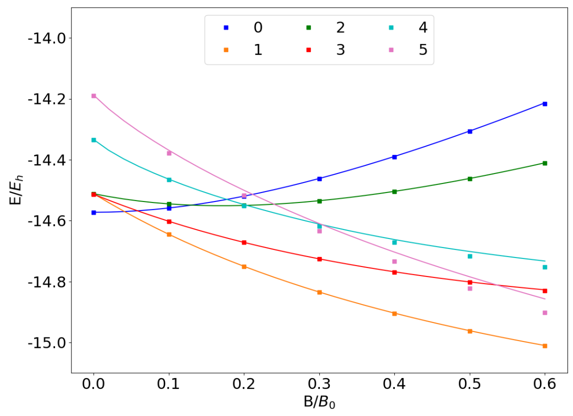

Figure S123: Total energies of all considered states of the Be atom in the AHGBSP2-5 basis set in fully uncontracted form (solid lines). The FEM values are shown by the squares of the same color.

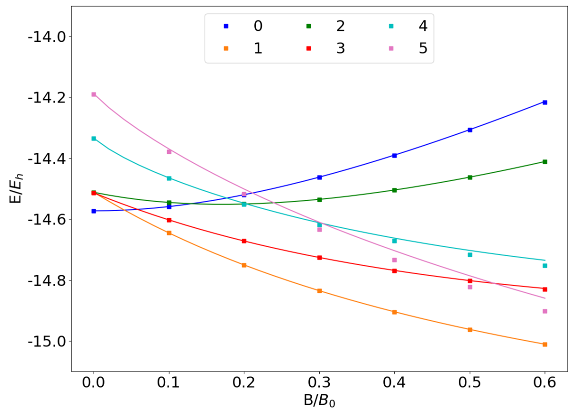

Figure S124: Total energies of all considered states of the Be atom in the AHGBSP2-7 basis set in fully uncontracted form (solid lines). The FEM values are shown by the squares of the same color.

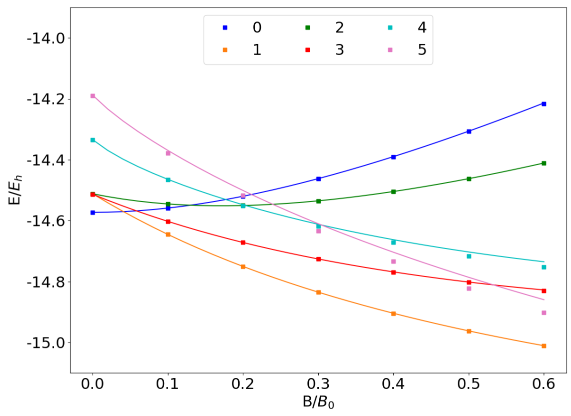

Figure S125: Total energies of all considered states of the Be atom in the AHGBSP2-9 basis set in fully uncontracted form (solid lines). The FEM values are shown by the squares of the same color.

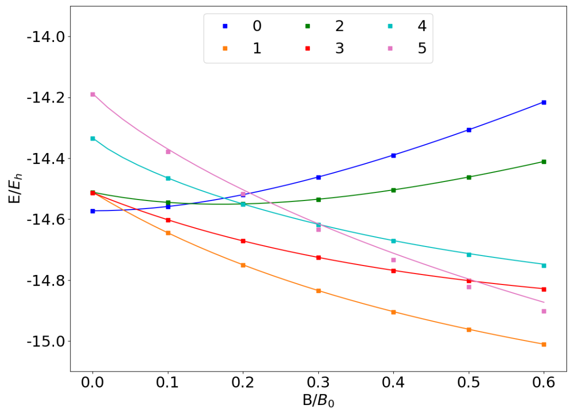

Figure S126: Total energies of all considered states of the Be atom in the AHGBSP3-5 basis set in fully uncontracted form (solid lines). The FEM values are shown by the squares of the same color.

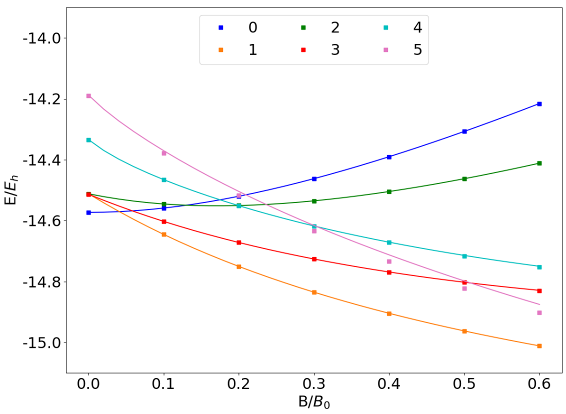

Figure S127: Total energies of all considered states of the Be atom in the AHGBSP3-7 basis set in fully uncontracted form (solid lines). The FEM values are shown by the squares of the same color.

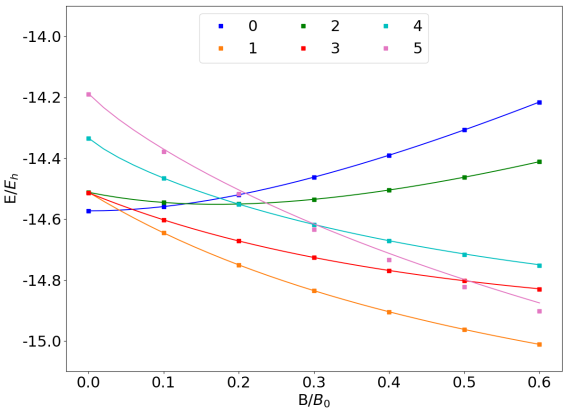

Figure S128: Total energies of all considered states of the Be atom in the AHGBSP3-9 basis set in fully uncontracted form (solid lines). The FEM values are shown by the squares of the same color.

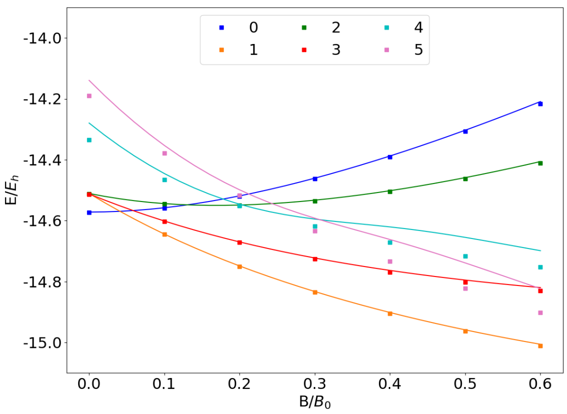

Figure S129: Total energies of all considered states of the Be atom in the 6-311++G(3df,3pd) basis set in fully uncontracted form (solid lines). The FEM values are shown by the squares of the same color.

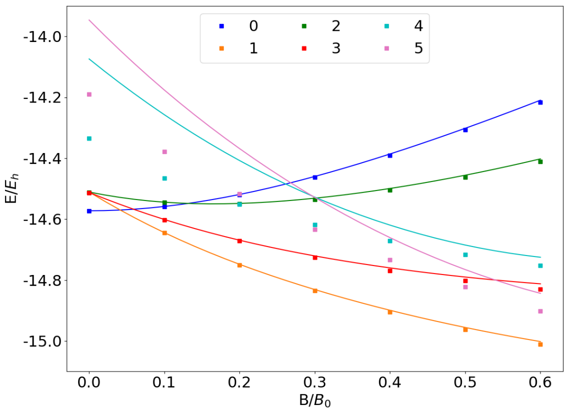

Figure S130: Total energies of all considered states of the Be atom in the def2-TZVP basis set in fully uncontracted form (solid lines). The FEM values are shown by the squares of the same color.

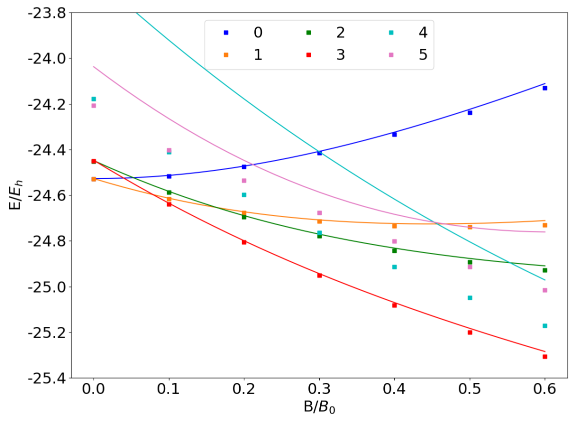

Figure S131: Total energies of all considered states of the B atom in the cc-pVDZ basis set in fully uncontracted form (solid lines). The FEM values are shown by the squares of the same color.

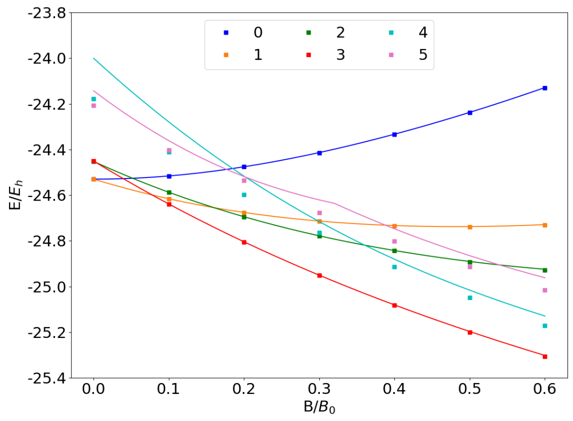

Figure S133: Total energies of all considered states of the B atom in the cc-pVQZ basis set in fully uncontracted form (solid lines). The FEM values are shown by the squares of the same color.

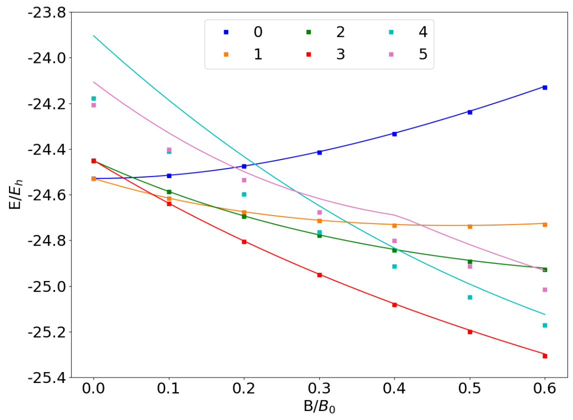

Figure S132: Total energies of all considered states of the B atom in the cc-pVTZ basis set in fully uncontracted form (solid lines). The FEM values are shown by the squares of the same color.

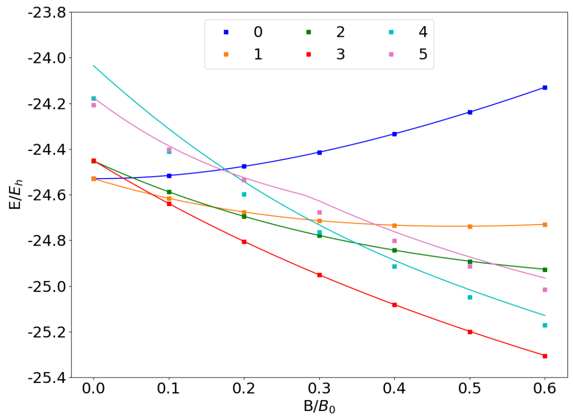

Figure S134: Total energies of all considered states of the B atom in the cc-pV5Z basis set in fully uncontracted form (solid lines). The FEM values are shown by the squares of the same color.

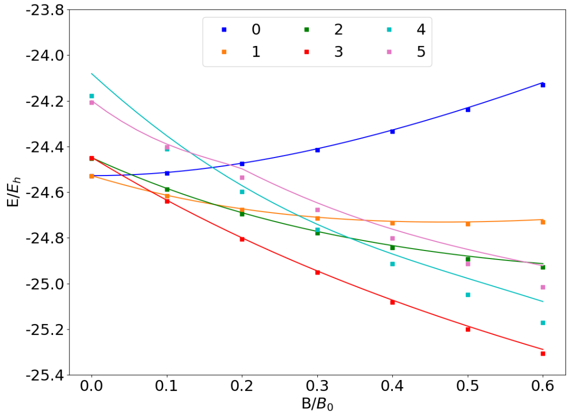

Figure S135: Total energies of all considered states of the B atom in the aug-cc-pVDZ basis set in fully uncontracted form (solid lines). The FEM values are shown by the squares of the same color.

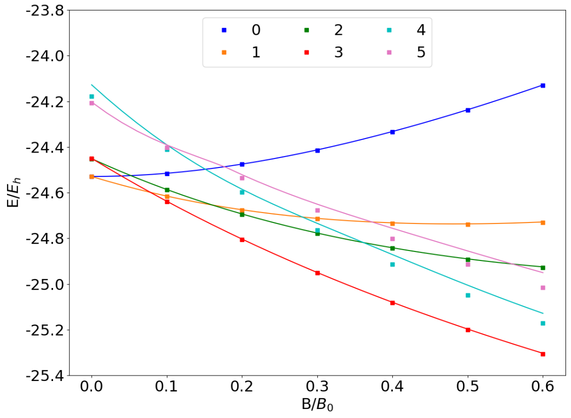

Figure S136: Total energies of all considered states of the B atom in the aug-cc-pVTZ basis set in fully uncontracted form (solid lines). The FEM values are shown by the squares of the same color.

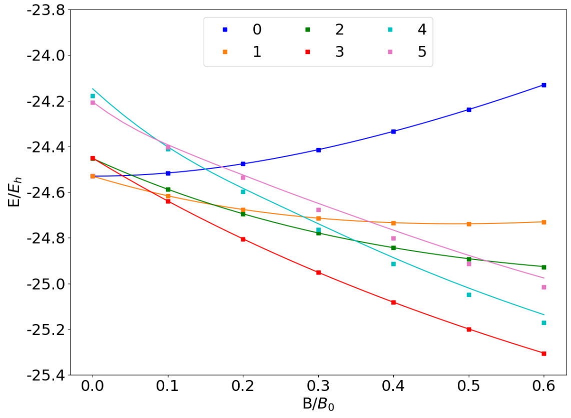

Figure S137: Total energies of all considered states of the B atom in the aug-cc-pVQZ basis set in fully uncontracted form (solid lines). The FEM values are shown by the squares of the same color.

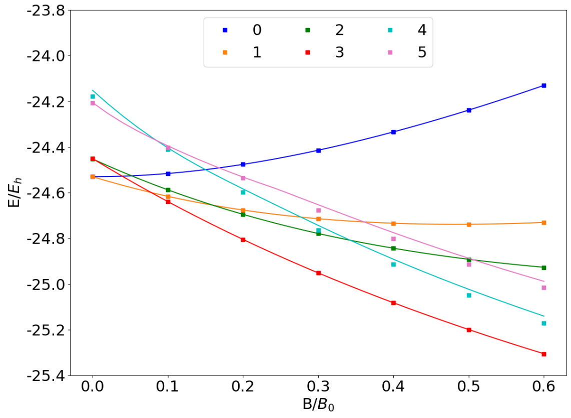

Figure S138: Total energies of all considered states of the B atom in the aug-cc-pV5Z basis set in fully uncontracted form (solid lines). The FEM values are shown by the squares of the same color.

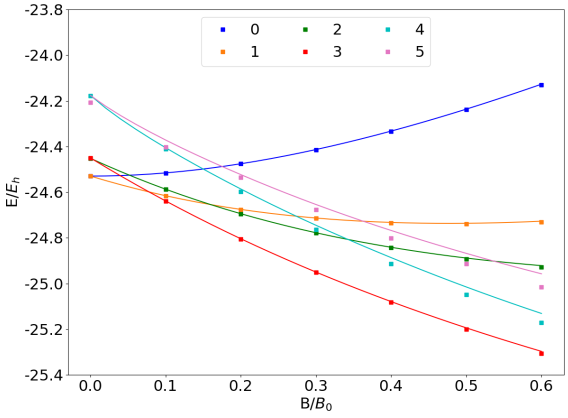

Figure S139: Total energies of all considered states of the B atom in the HGBSP1-5 basis set in fully uncontracted form (solid lines). The FEM values are shown by the squares of the same color.

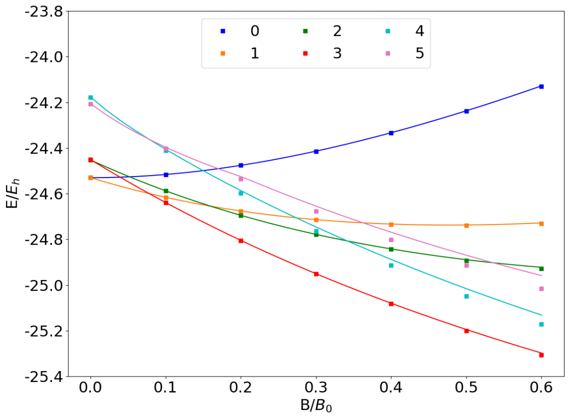

Figure S140: Total energies of all considered states of the B atom in the HGBSP1-7 basis set in fully uncontracted form (solid lines). The FEM values are shown by the squares of the same color.

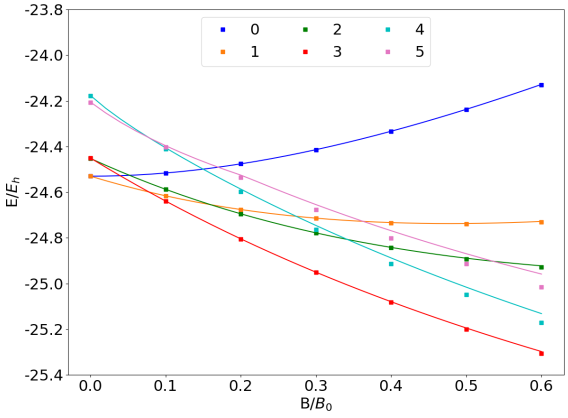

Figure S141: Total energies of all considered states of the B atom in the HGBSP1-9 basis set in fully uncontracted form (solid lines). The FEM values are shown by the squares of the same color.

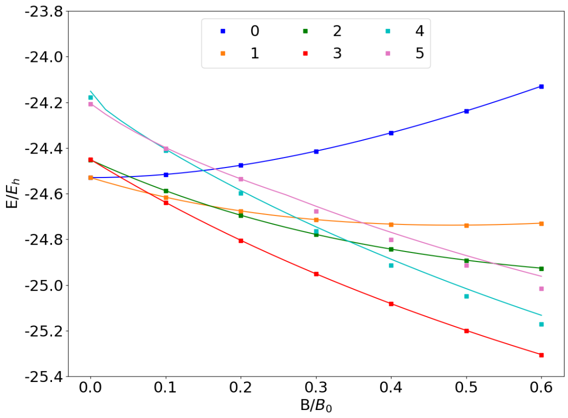

Figure S142: Total energies of all considered states of the B atom in the HGBSP2-5 basis set in fully uncontracted form (solid lines). The FEM values are shown by the squares of the same color.

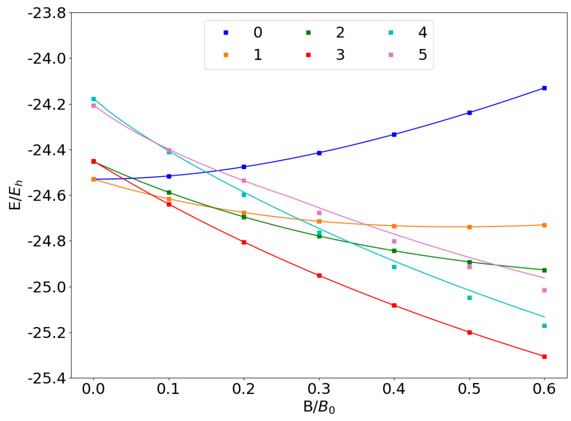

Figure S143: Total energies of all considered states of the B atom in the HGBSP2-7 basis set in fully uncontracted form (solid lines). The FEM values are shown by the squares of the same color.

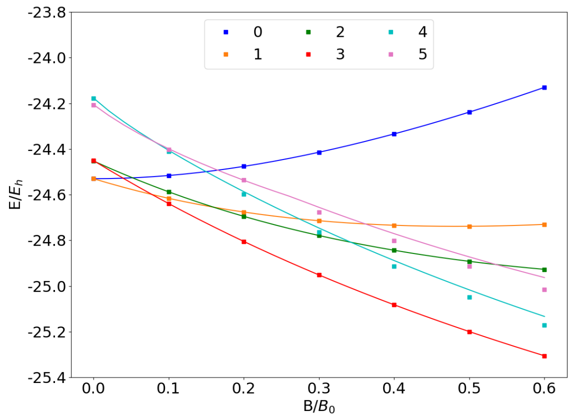

Figure S144: Total energies of all considered states of the B atom in the HGBSP2-9 basis set in fully uncontracted form (solid lines). The FEM values are shown by the squares of the same color.

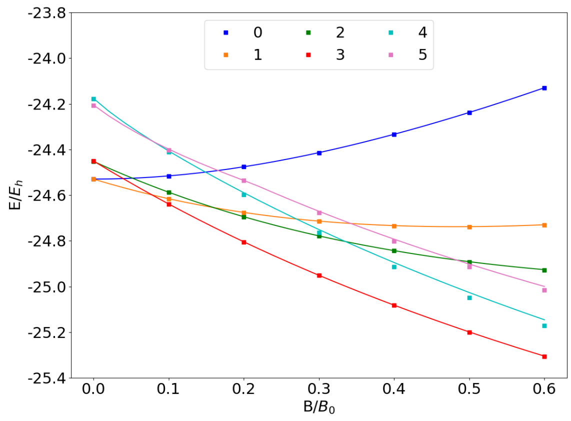

Figure S145: Total energies of all considered states of the B atom in the HGBSP3-5 basis set in fully uncontracted form (solid lines). The FEM values are shown by the squares of the same color.

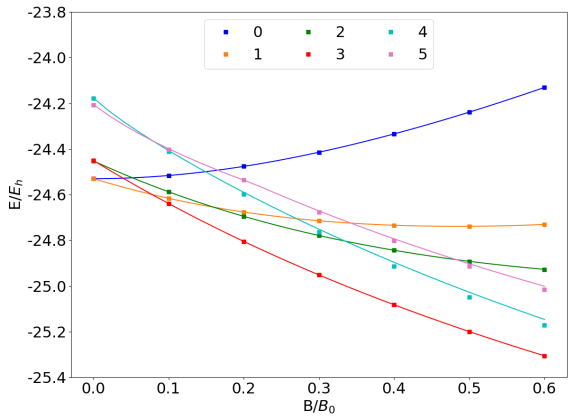

Figure S146: Total energies of all considered states of the B atom in the HGBSP3-7 basis set in fully uncontracted form (solid lines). The FEM values are shown by the squares of the same color.

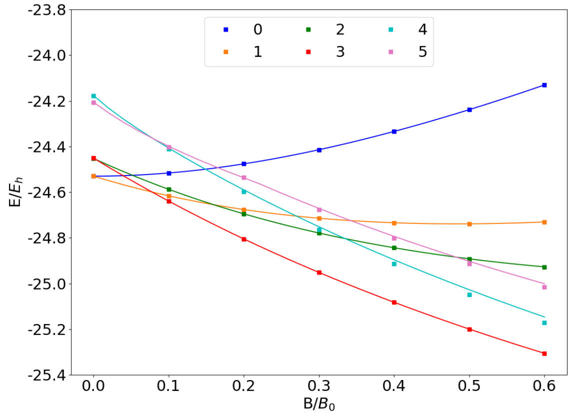

Figure S147: Total energies of all considered states of the B atom in the HGBSP3-9 basis set in fully uncontracted form (solid lines). The FEM values are shown by the squares of the same color.

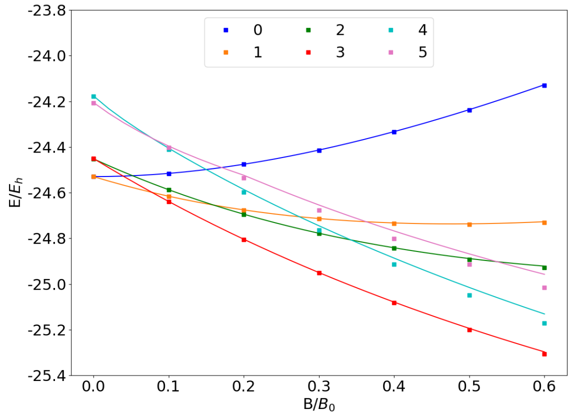

Figure S148: Total energies of all considered states of the B atom in the AHGBSP1-5 basis set in fully uncontracted form (solid lines). The FEM values are shown by the squares of the same color.

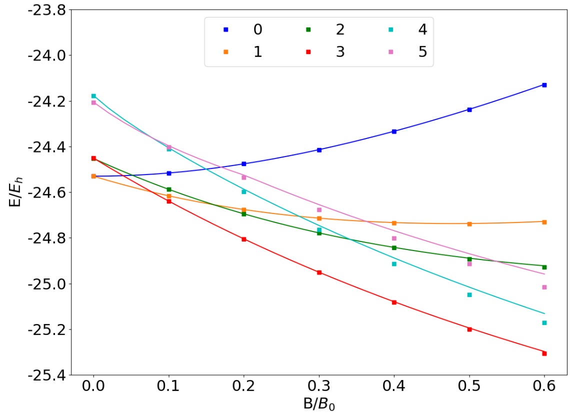

Figure S149: Total energies of all considered states of the B atom in the AHGBSP1-7 basis set in fully uncontracted form (solid lines). The FEM values are shown by the squares of the same color.

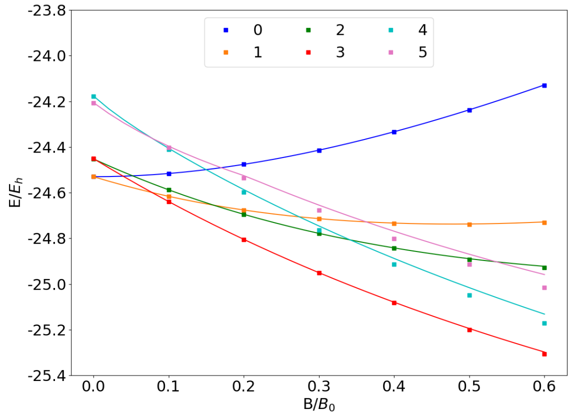

Figure S150: Total energies of all considered states of the B atom in the AHGBSP1-9 basis set in fully uncontracted form (solid lines). The FEM values are shown by the squares of the same color.

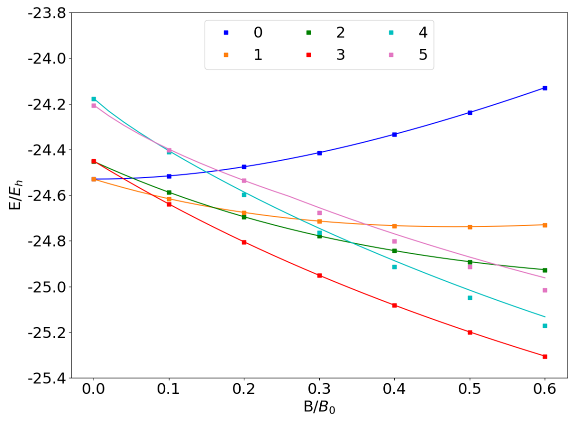

Figure S151: Total energies of all considered states of the B atom in the AHGBSP2-5 basis set in fully uncontracted form (solid lines). The FEM values are shown by the squares of the same color.

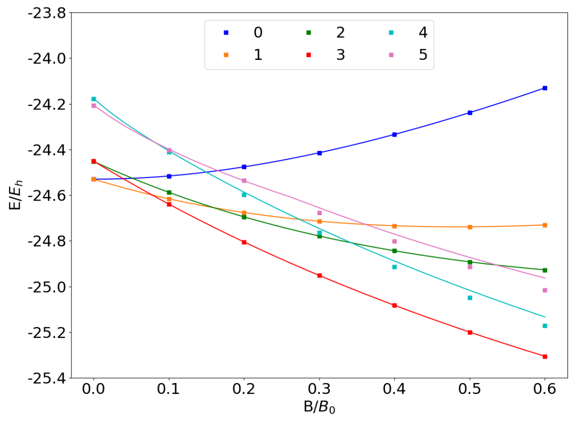

Figure S153: Total energies of all considered states of the B atom in the AHGBSP2-9 basis set in fully uncontracted form (solid lines). The FEM values are shown by the squares of the same color.

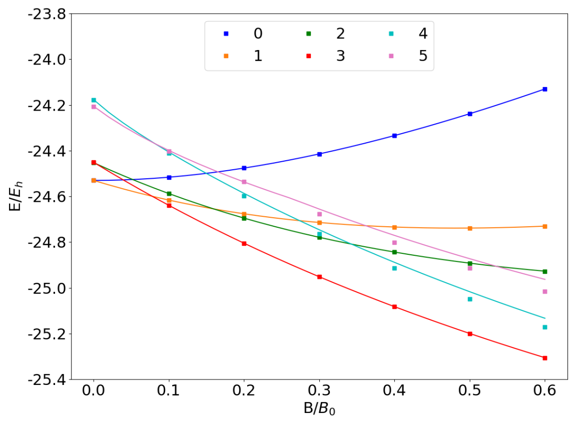

Figure S152: Total energies of all considered states of the B atom in the AHGBSP2-7 basis set in fully uncontracted form (solid lines). The FEM values are shown by the squares of the same color.

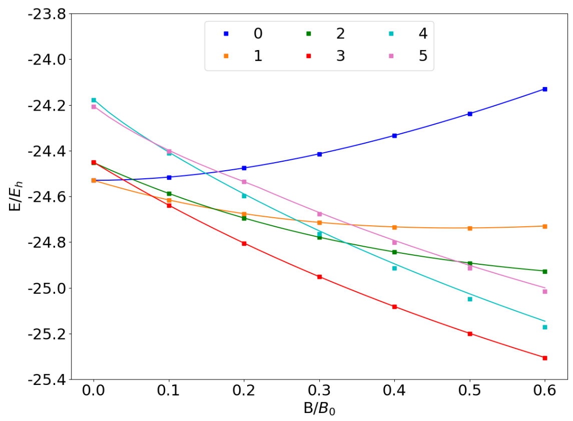

Figure S154: Total energies of all considered states of the B atom in the AHGBSP3-5 basis set in fully uncontracted form (solid lines). The FEM values are shown by the squares of the same color.

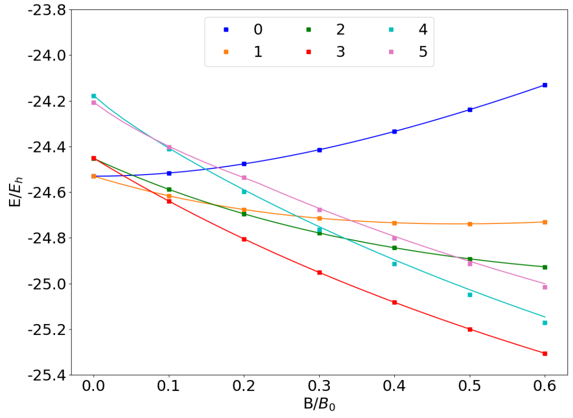

Figure S155: Total energies of all considered states of the B atom in the AHGBSP3-7 basis set in fully uncontracted form (solid lines). The FEM values are shown by the squares of the same color.

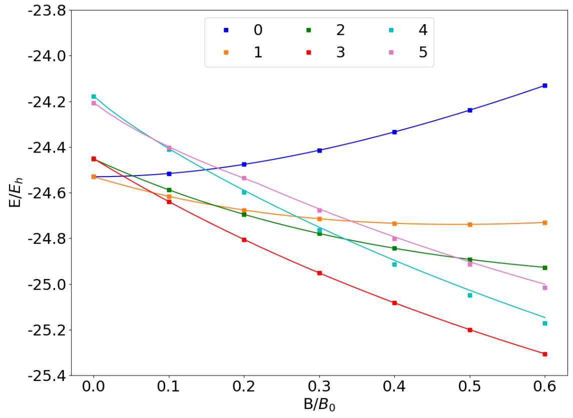

Figure S156: Total energies of all considered states of the B atom in the AHGBSP3-9 basis set in fully uncontracted form (solid lines). The FEM values are shown by the squares of the same color.

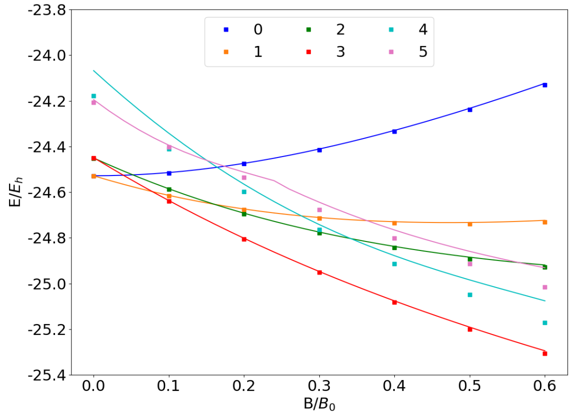

Figure S157: Total energies of all considered states of the B atom in the 6-311++G(3df,3pd) basis set in fully uncontracted form (solid lines). The FEM values are shown by the squares of the same color.

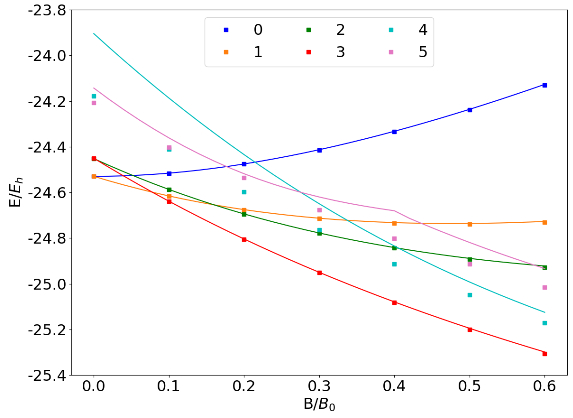

Figure S158: Total energies of all considered states of the B atom in the def2-TZVP basis set in fully uncontracted form (solid lines). The FEM values are shown by the squares of the same color.

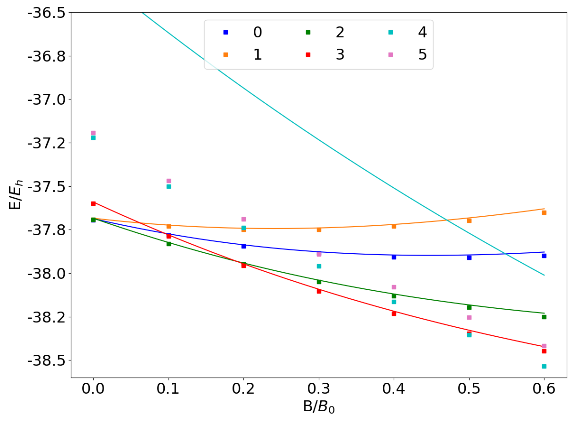

Figure S159: Total energies of all considered states of the C atom in the cc-pVDZ basis set in fully uncontracted form (solid lines). The FEM values are shown by the squares of the same color.

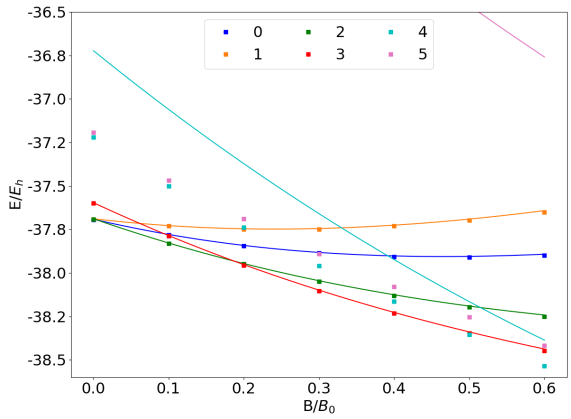

Figure S160: Total energies of all considered states of the C atom in the cc-pVTZ basis set in fully uncontracted form (solid lines). The FEM values are shown by the squares of the same color.

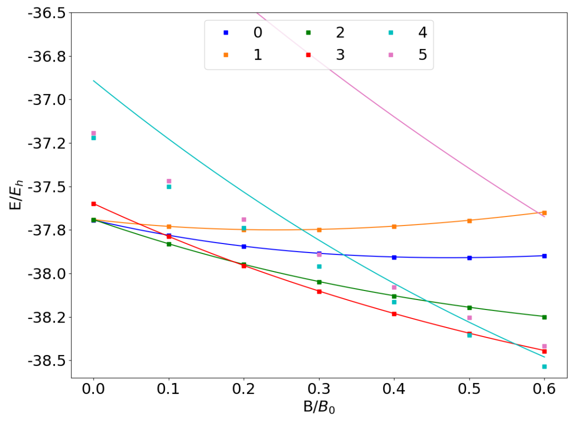

Figure S161: Total energies of all considered states of the C atom in the cc-pVQZ basis set in fully uncontracted form (solid lines). The FEM values are shown by the squares of the same color.

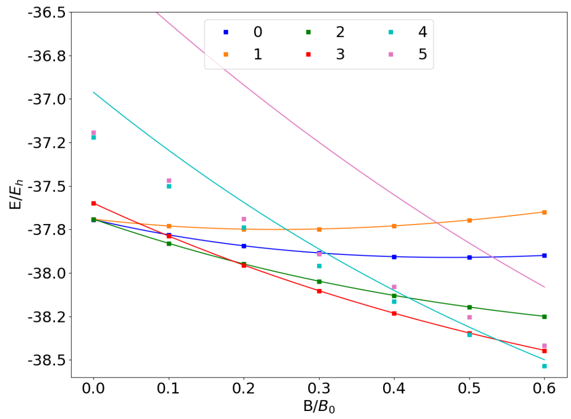

Figure S162: Total energies of all considered states of the C atom in the cc-pV5Z basis set in fully uncontracted form (solid lines). The FEM values are shown by the squares of the same color.

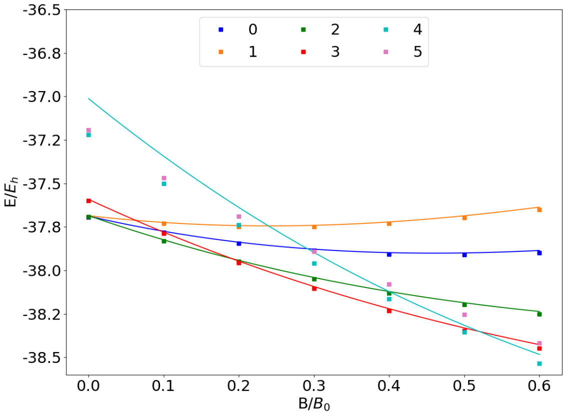

Figure S163: Total energies of all considered states of the C atom in the aug-cc-pVDZ basis set in fully uncontracted form (solid lines). The FEM values are shown by the squares of the same color.

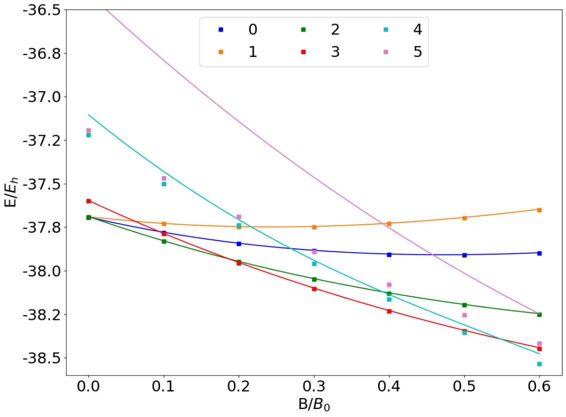

Figure S164: Total energies of all considered states of the C atom in the aug-cc-pVTZ basis set in fully uncontracted form (solid lines). The FEM values are shown by the squares of the same color.

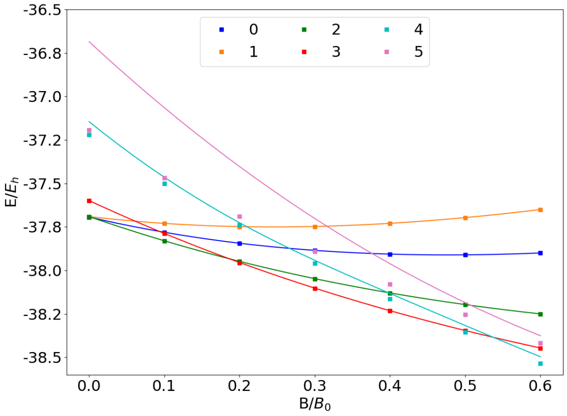

Figure S165: Total energies of all considered states of the C atom in the aug-cc-pVQZ basis set in fully uncontracted form (solid lines). The FEM values are shown by the squares of the same color.

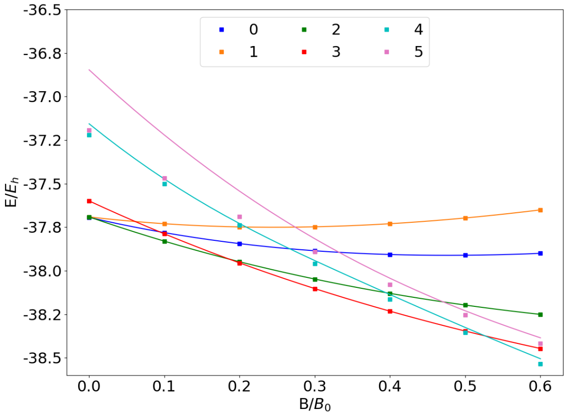

Figure S166: Total energies of all considered states of the C atom in the aug-cc-pV5Z basis set in fully uncontracted form (solid lines). The FEM values are shown by the squares of the same color.

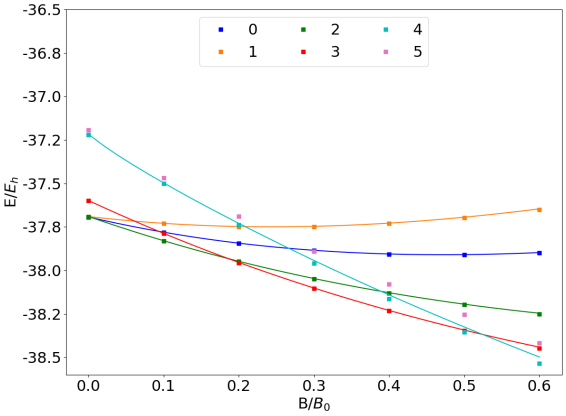

Figure S167: Total energies of all considered states of the C atom in the HGBSP1-5 basis set in fully uncontracted form (solid lines). The FEM values are shown by the squares of the same color.

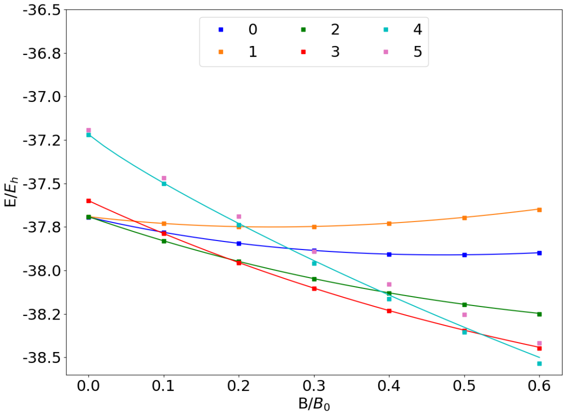

Figure S168: Total energies of all considered states of the C atom in the HGBSP1-7 basis set in fully uncontracted form (solid lines). The FEM values are shown by the squares of the same color.

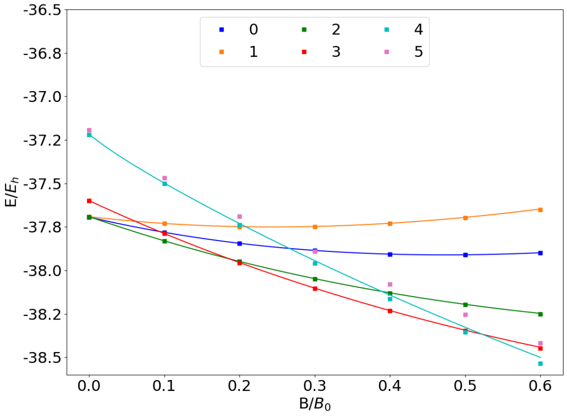

Figure S169: Total energies of all considered states of the C atom in the HGBSP1-9 basis set in fully uncontracted form (solid lines). The FEM values are shown by the squares of the same color.

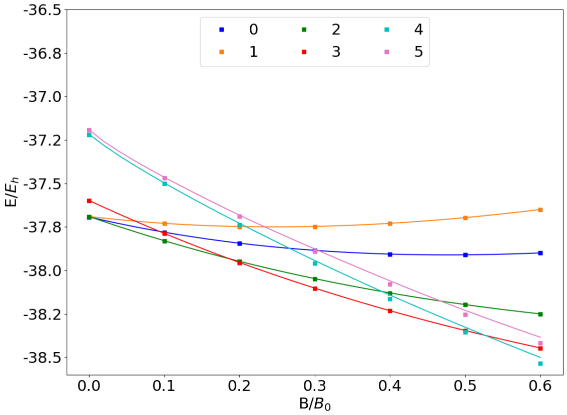

Figure S170: Total energies of all considered states of the C atom in the HGBSP2-5 basis set in fully uncontracted form (solid lines). The FEM values are shown by the squares of the same color.

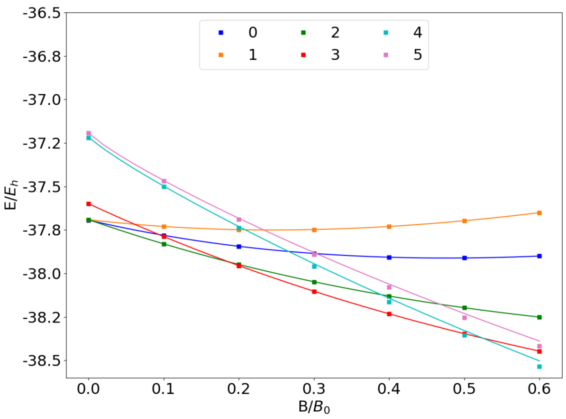

Figure S171: Total energies of all considered states of the C atom in the HGBSP2-7 basis set in fully uncontracted form (solid lines). The FEM values are shown by the squares of the same color.

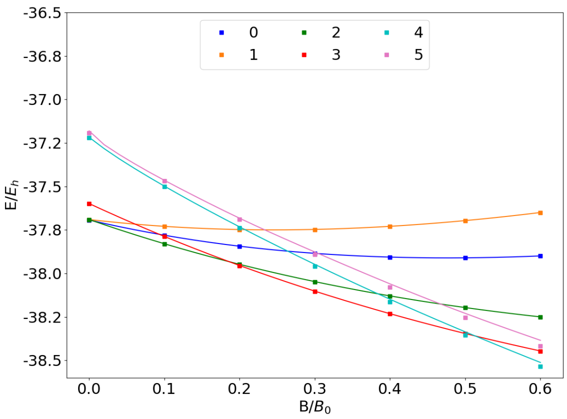

Figure S173: Total energies of all considered states of the C atom in the HGBSP3-5 basis set in fully uncontracted form (solid lines). The FEM values are shown by the squares of the same color.

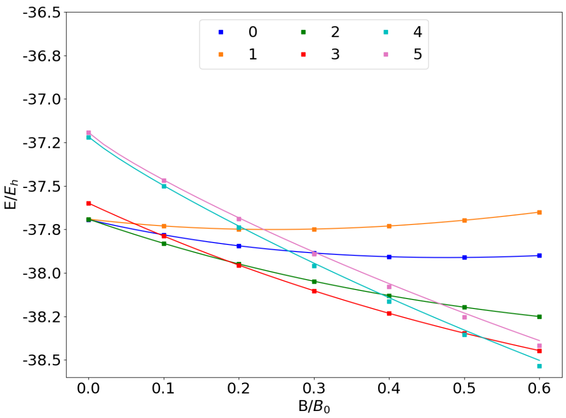

Figure S172: Total energies of all considered states of the C atom in the HGBSP2-9 basis set in fully uncontracted form (solid lines). The FEM values are shown by the squares of the same color.

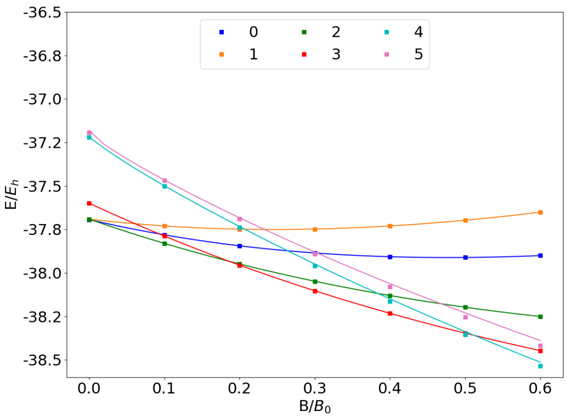

Figure S174: Total energies of all considered states of the C atom in the HGBSP3-7 basis set in fully uncontracted form (solid lines). The FEM values are shown by the squares of the same color.

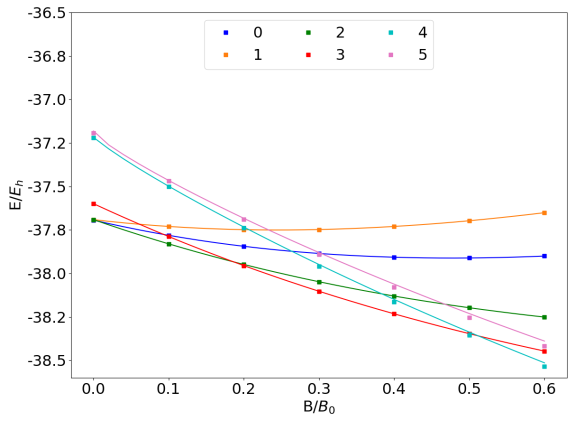

Figure S175: Total energies of all considered states of the C atom in the HGBSP3-9 basis set in fully uncontracted form (solid lines). The FEM values are shown by the squares of the same color.

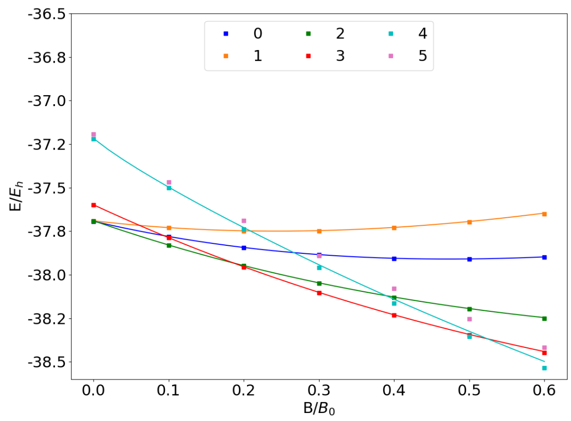

Figure S176: Total energies of all considered states of the C atom in the AHGBSP1-5 basis set in fully uncontracted form (solid lines). The FEM values are shown by the squares of the same color.

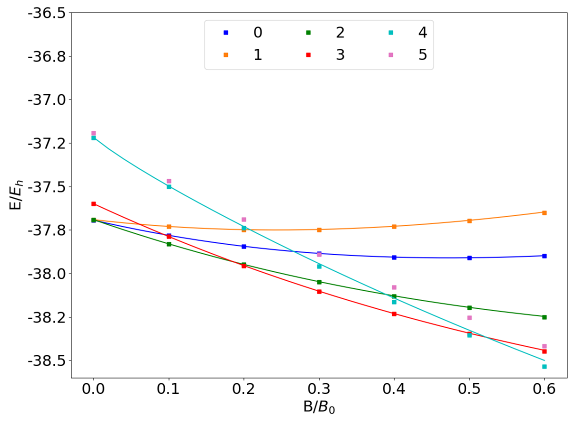

Figure S177: Total energies of all considered states of the C atom in the AHGBSP1-7 basis set in fully uncontracted form (solid lines). The FEM values are shown by the squares of the same color.

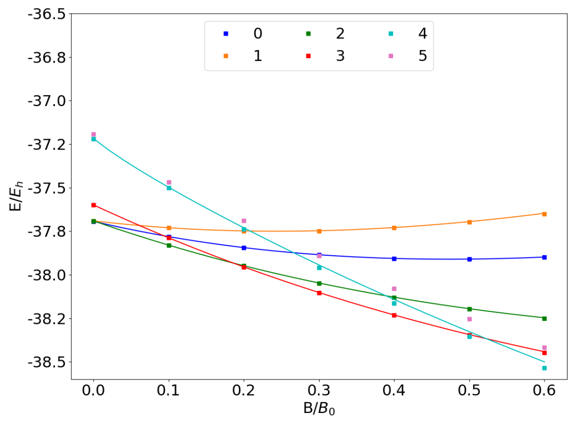

Figure S178: Total energies of all considered states of the C atom in the AHGBSP1-9 basis set in fully uncontracted form (solid lines). The FEM values are shown by the squares of the same color.

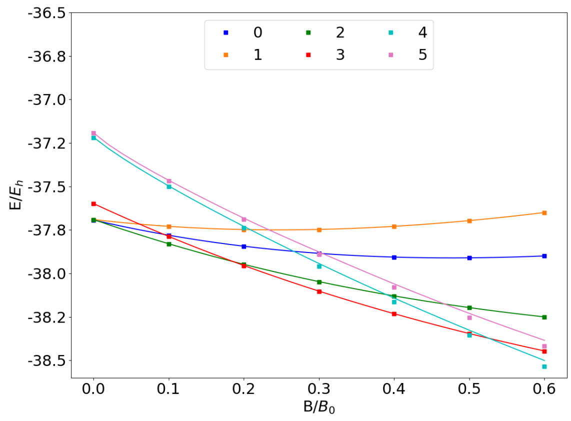

Figure S179: Total energies of all considered states of the C atom in the AHGBSP2-5 basis set in fully uncontracted form (solid lines). The FEM values are shown by the squares of the same color.

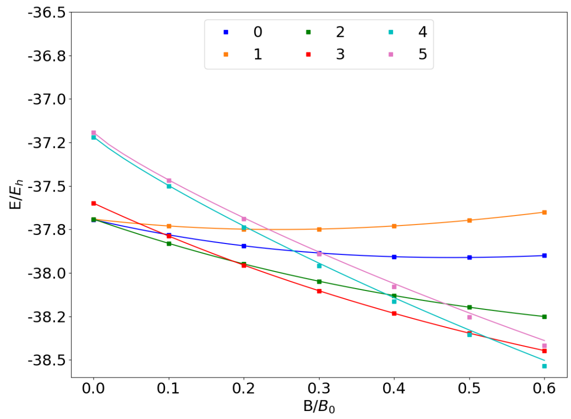

Figure S180: Total energies of all considered states of the C atom in the AHGBSP2-7 basis set in fully uncontracted form (solid lines). The FEM values are shown by the squares of the same color.

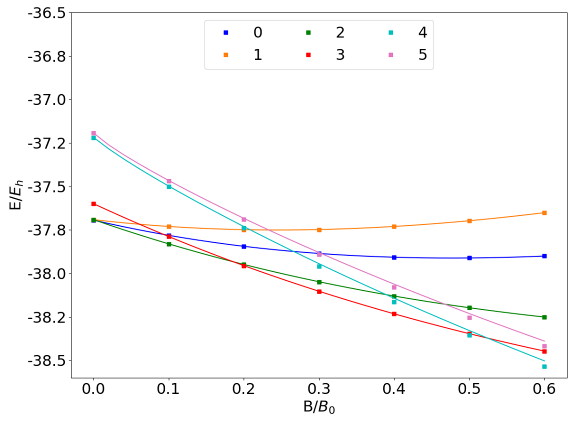

Figure S181: Total energies of all considered states of the C atom in the AHGBSP2-9 basis set in fully uncontracted form (solid lines). The FEM values are shown by the squares of the same color.

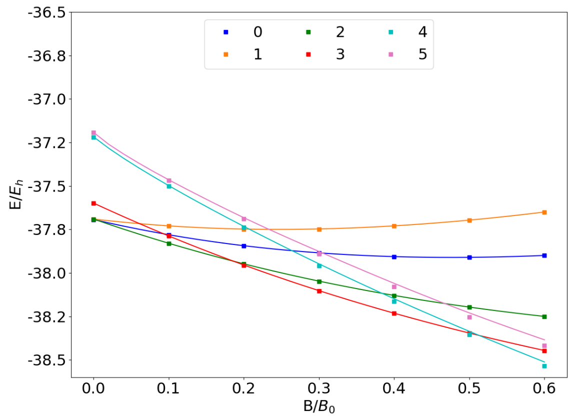

Figure S182: Total energies of all considered states of the C atom in the AHGBSP3-5 basis set in fully uncontracted form (solid lines). The FEM values are shown by the squares of the same color.

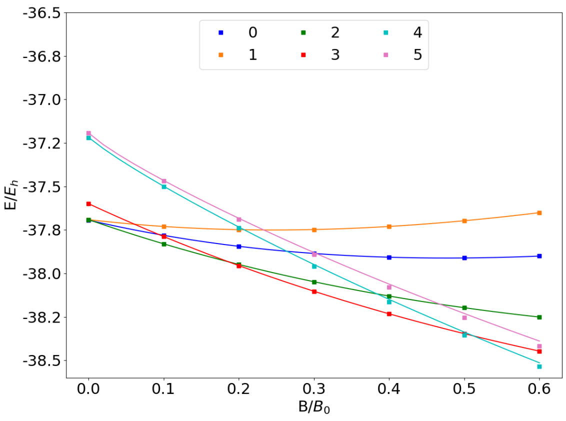

Figure S183: Total energies of all considered states of the C atom in the AHGBSP3-7 basis set in fully uncontracted form (solid lines). The FEM values are shown by the squares of the same color.

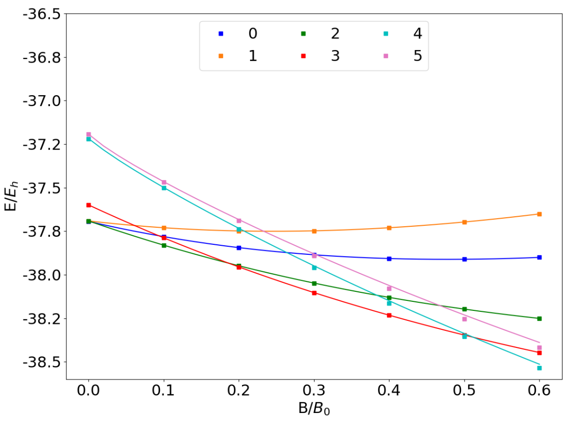

Figure S184: Total energies of all considered states of the C atom in the AHGBSP3-9 basis set in fully uncontracted form (solid lines). The FEM values are shown by the squares of the same color.

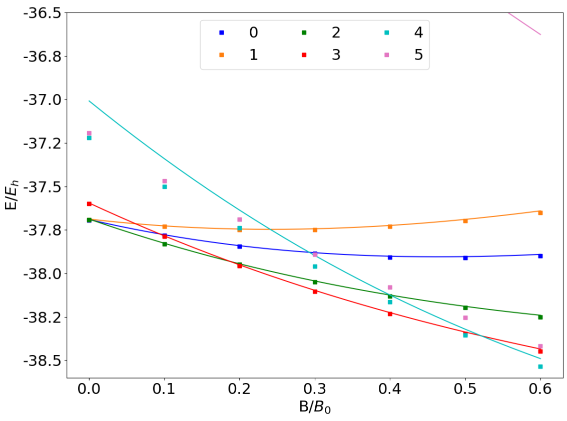

Figure S185: Total energies of all considered states of the C atom in the 6-311++G(3df,3pd) basis set in fully uncontracted form (solid lines). The FEM values are shown by the squares of the same color.

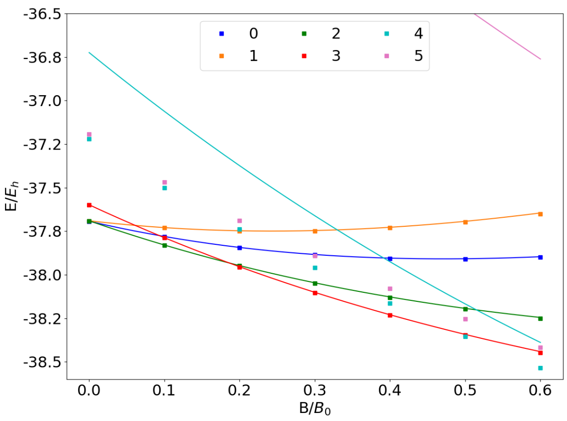

Figure S186: Total energies of all considered states of the C atom in the def2-TZVP basis set in fully uncontracted form (solid lines). The FEM values are shown by the squares of the same color.

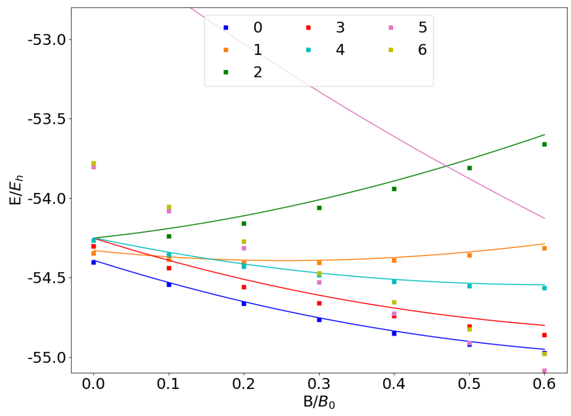

Figure S187: Total energies of all considered states of the N atom in the cc-pVDZ basis set in fully uncontracted form (solid lines). The FEM values are shown by the squares of the same color.

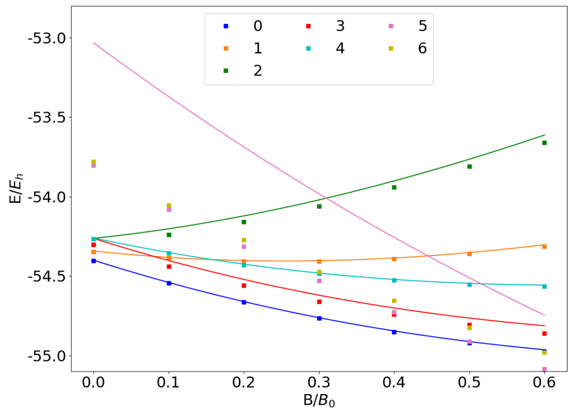

Figure S188: Total energies of all considered states of the N atom in the cc-pVTZ basis set in fully uncontracted form (solid lines). The FEM values are shown by the squares of the same color.

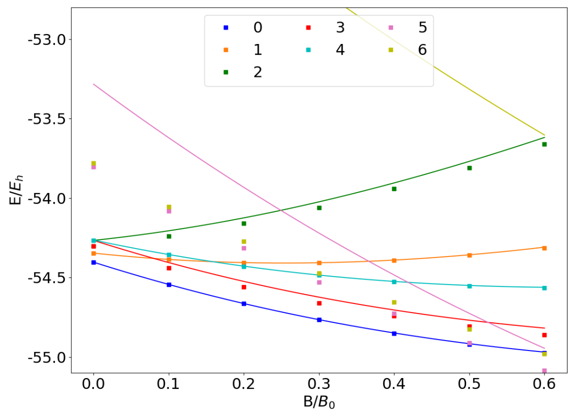

Figure S189: Total energies of all considered states of the N atom in the cc-pVQZ basis set in fully uncontracted form (solid lines). The FEM values are shown by the squares of the same color.

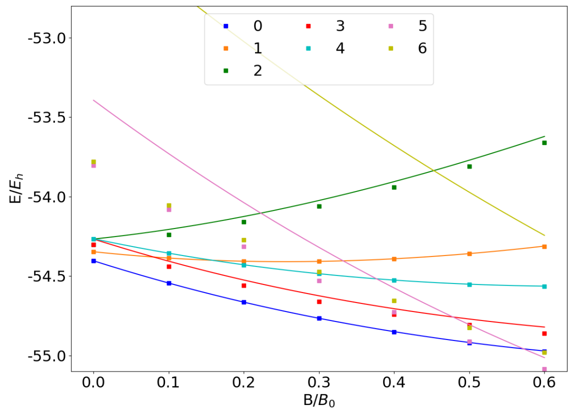

Figure S190: Total energies of all considered states of the N atom in the cc-pV5Z basis set in fully uncontracted form (solid lines). The FEM values are shown by the squares of the same color.

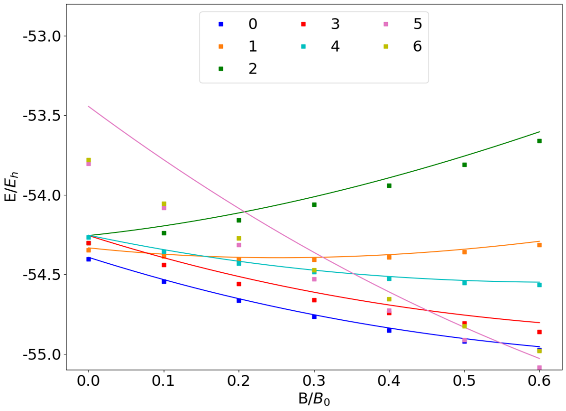

Figure S191: Total energies of all considered states of the N atom in the aug-cc-pVDZ basis set in fully uncontracted form (solid lines). The FEM values are shown by the squares of the same color.

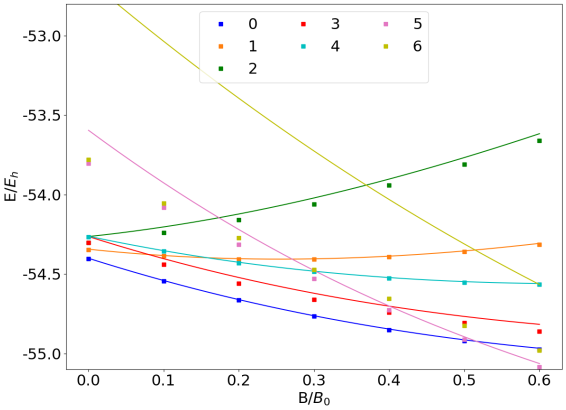

Figure S192: Total energies of all considered states of the N atom in the aug-cc-pVTZ basis set in fully uncontracted form (solid lines). The FEM values are shown by the squares of the same color.

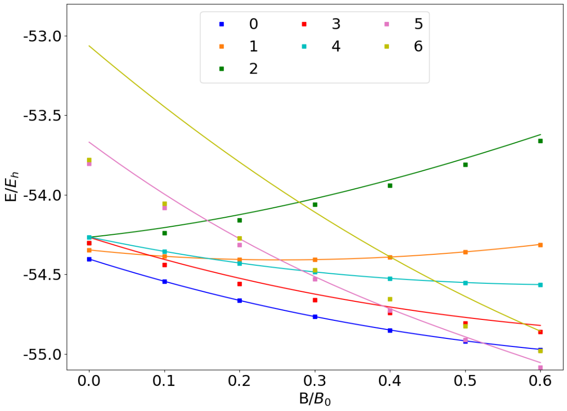

Figure S193: Total energies of all considered states of the N atom in the aug-cc-pVQZ basis set in fully uncontracted form (solid lines). The FEM values are shown by the squares of the same color.

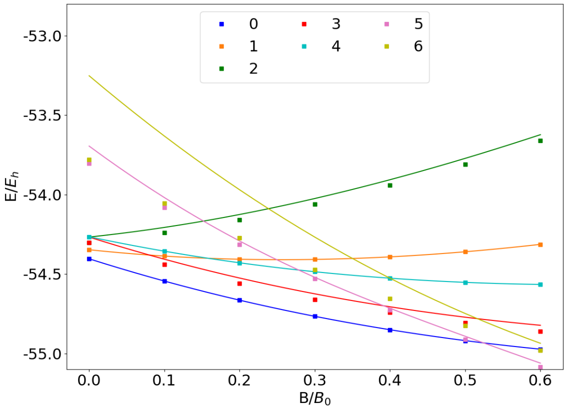

Figure S194: Total energies of all considered states of the N atom in the aug-cc-pV5Z basis set in fully uncontracted form (solid lines). The FEM values are shown by the squares of the same color.

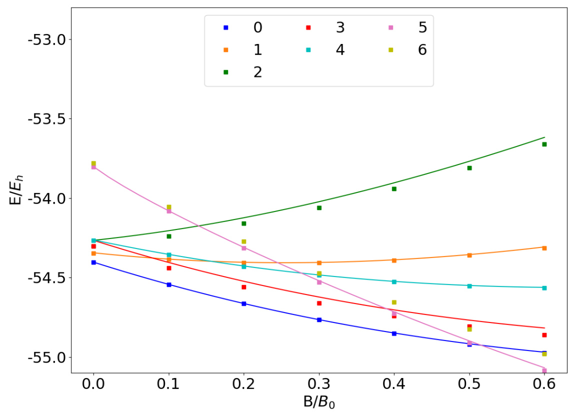

Figure S195: Total energies of all considered states of the N atom in the HGBSP1-5 basis set in fully uncontracted form (solid lines). The FEM values are shown by the squares of the same color.

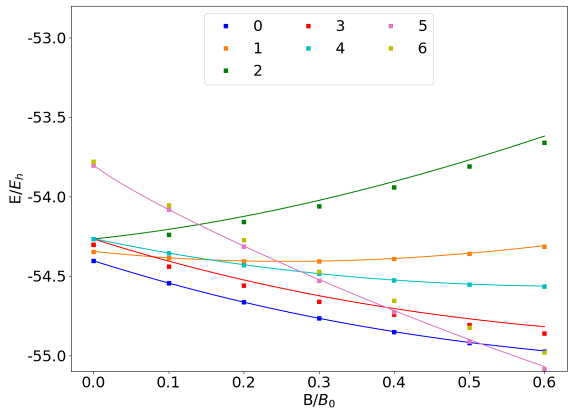

Figure S196: Total energies of all considered states of the N atom in the HGBSP1-7 basis set in fully uncontracted form (solid lines). The FEM values are shown by the squares of the same color.

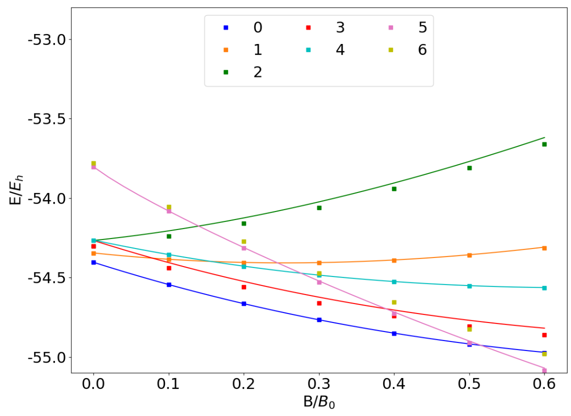

Figure S197: Total energies of all considered states of the N atom in the HGBSP1-9 basis set in fully uncontracted form (solid lines). The FEM values are shown by the squares of the same color.

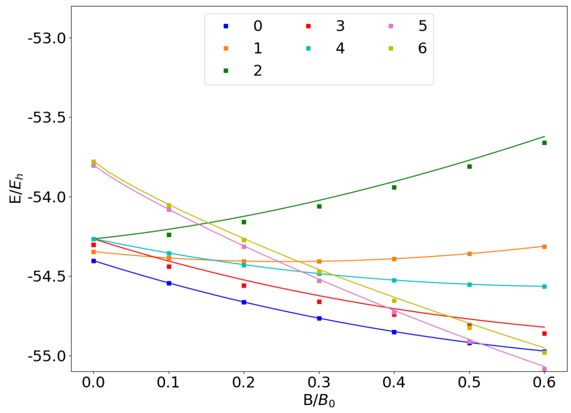

Figure S198: Total energies of all considered states of the N atom in the HGBSP2-5 basis set in fully uncontracted form (solid lines). The FEM values are shown by the squares of the same color.

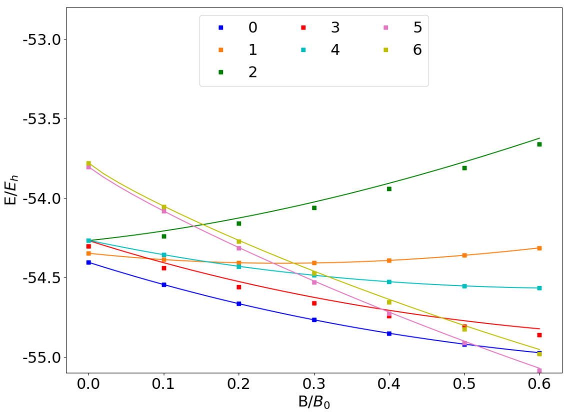

Figure S199: Total energies of all considered states of the N atom in the HGBSP2-7 basis set in fully uncontracted form (solid lines). The FEM values are shown by the squares of the same color.

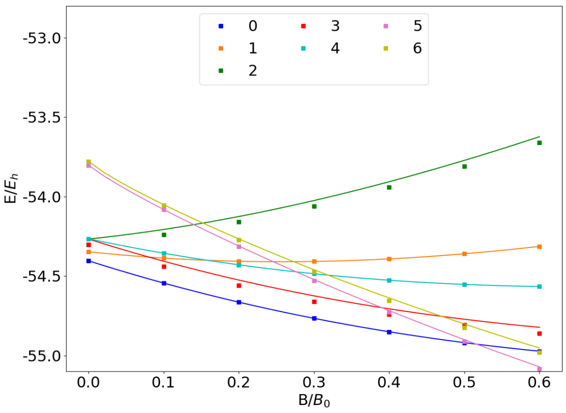

Figure S200: Total energies of all considered states of the N atom in the HGBSP2-9 basis set in fully uncontracted form (solid lines). The FEM values are shown by the squares of the same color.

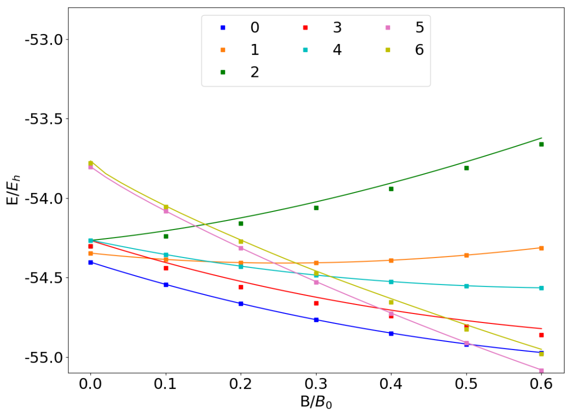

Figure S201: Total energies of all considered states of the N atom in the HGBSP3-5 basis set in fully uncontracted form (solid lines). The FEM values are shown by the squares of the same color.

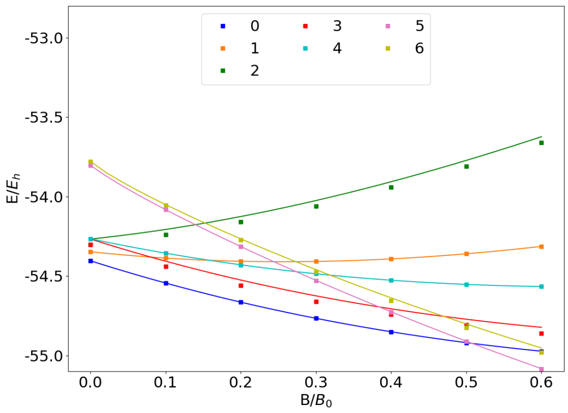

Figure S202: Total energies of all considered states of the N atom in the HGBSP3-7 basis set in fully uncontracted form (solid lines). The FEM values are shown by the squares of the same color.

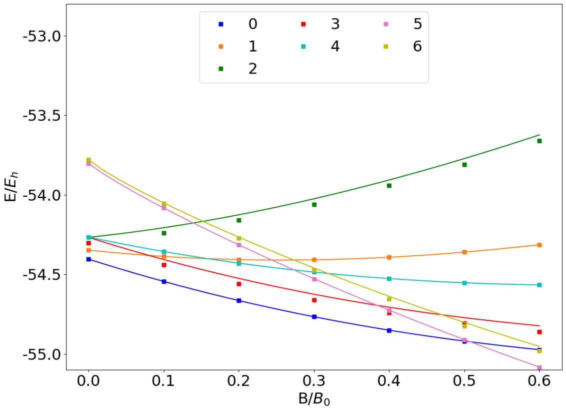

Figure S203: Total energies of all considered states of the N atom in the HGBSP3-9 basis set in fully uncontracted form (solid lines). The FEM values are shown by the squares of the same color.

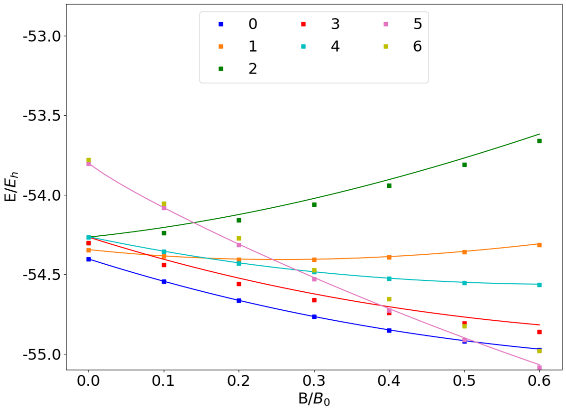

Figure S204: Total energies of all considered states of the N atom in the AHGBSP1-5 basis set in fully uncontracted form (solid lines). The FEM values are shown by the squares of the same color.

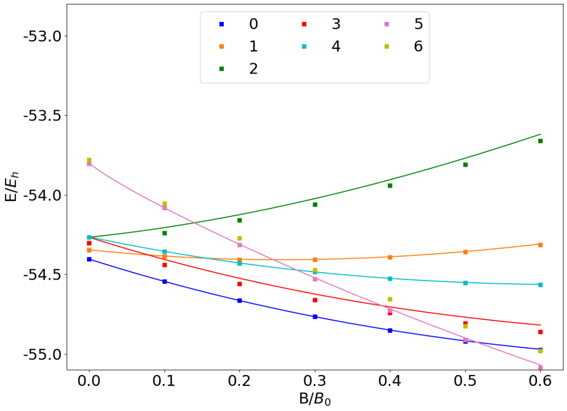

Figure S205: Total energies of all considered states of the N atom in the AHGBSP1-7 basis set in fully uncontracted form (solid lines). The FEM values are shown by the squares of the same color.

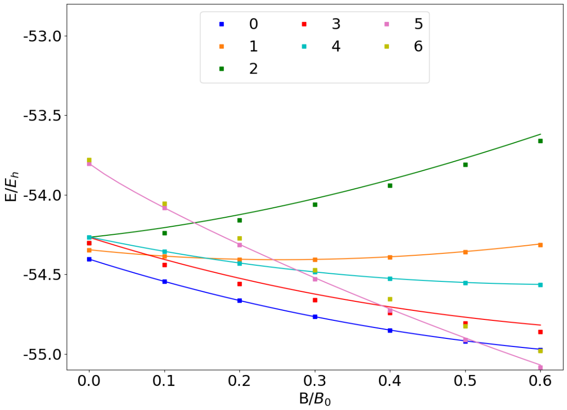

Figure S206: Total energies of all considered states of the N atom in the AHGBSP1-9 basis set in fully uncontracted form (solid lines). The FEM values are shown by the squares of the same color.

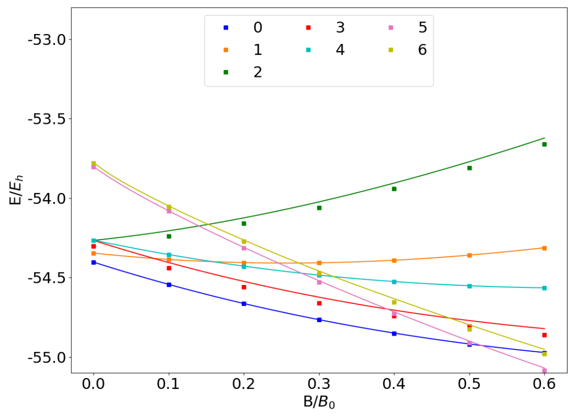

Figure S207: Total energies of all considered states of the N atom in the AHGBSP2-5 basis set in fully uncontracted form (solid lines). The FEM values are shown by the squares of the same color.

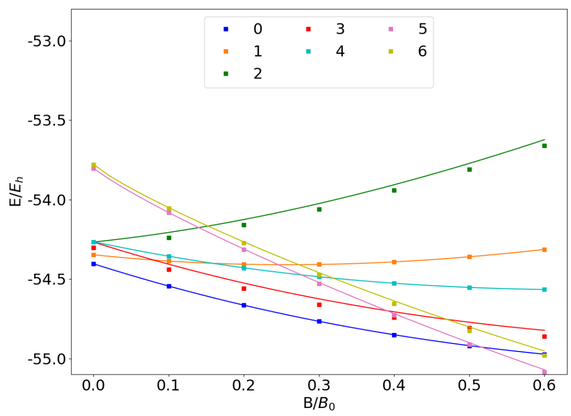

Figure S208: Total energies of all considered states of the N atom in the AHGBSP2-7 basis set in fully uncontracted form (solid lines). The FEM values are shown by the squares of the same color.

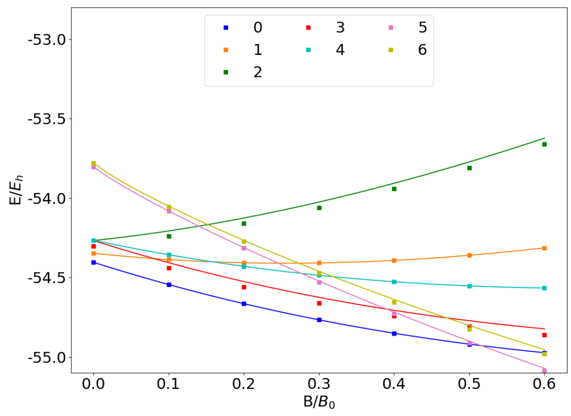

Figure S209: Total energies of all considered states of the N atom in the AHGBSP2-9 basis set in fully uncontracted form (solid lines). The FEM values are shown by the squares of the same color.

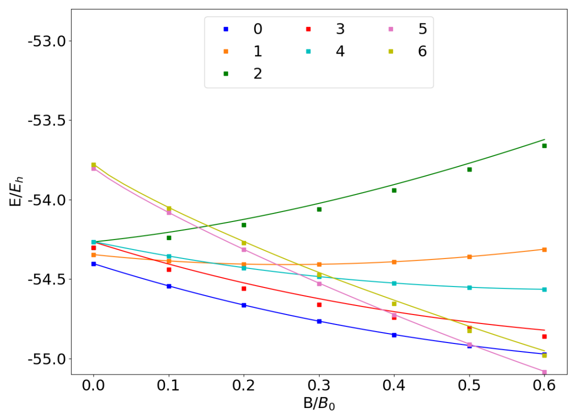

Figure S210: Total energies of all considered states of the N atom in the AHGBSP3-5 basis set in fully uncontracted form (solid lines). The FEM values are shown by the squares of the same color.

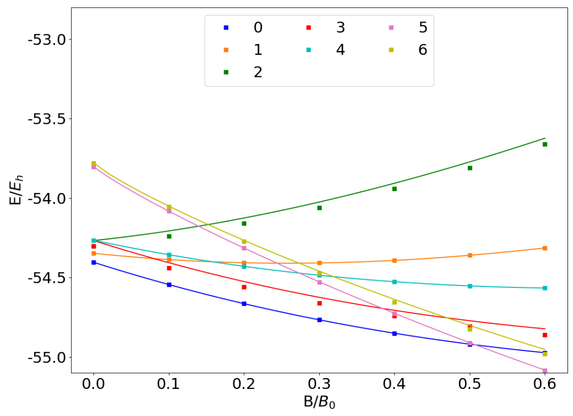

Figure S211: Total energies of all considered states of the N atom in the AHGBSP3-7 basis set in fully uncontracted form (solid lines). The FEM values are shown by the squares of the same color.

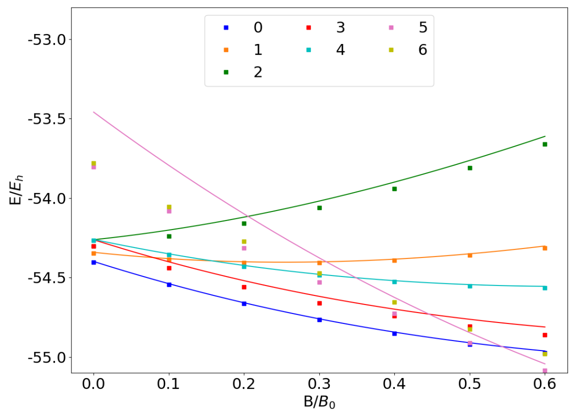

Figure S213: Total energies of all considered states of the N atom in the 6-311++G(3df,3pd) basis set in fully uncontracted form (solid lines). The FEM values are shown by the squares of the same color.

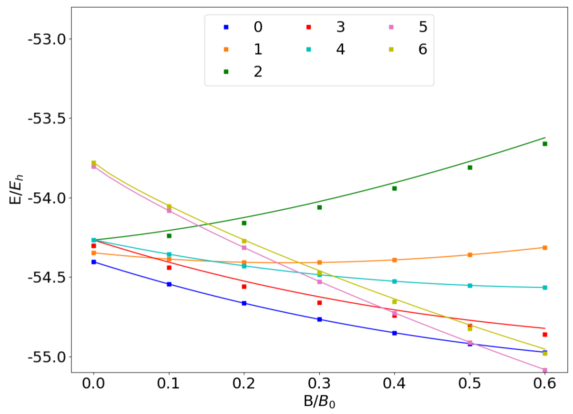

Figure S212: Total energies of all considered states of the N atom in the AHGBSP3-9 basis set in fully uncontracted form (solid lines). The FEM values are shown by the squares of the same color.

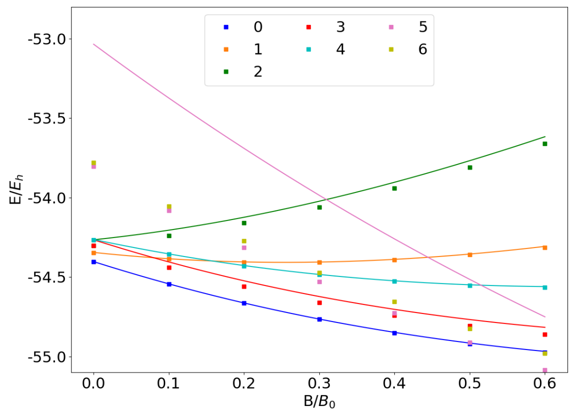

Figure S214: Total energies of all considered states of the N atom in the def2-TZVP basis set in fully uncontracted form (solid lines). The FEM values are shown by the squares of the same color.

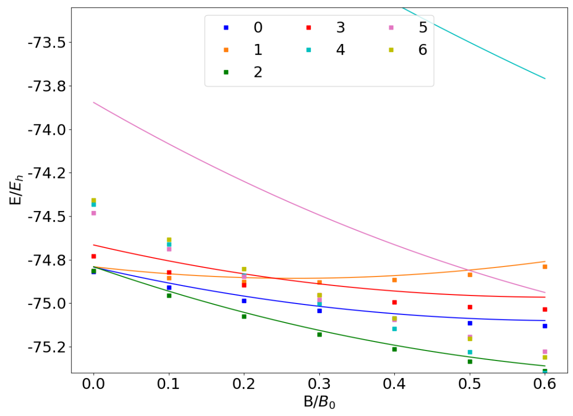

Figure S215: Total energies of all considered states of the O atom in the cc-pVDZ basis set in fully uncontracted form (solid lines). The FEM values are shown by the squares of the same color.

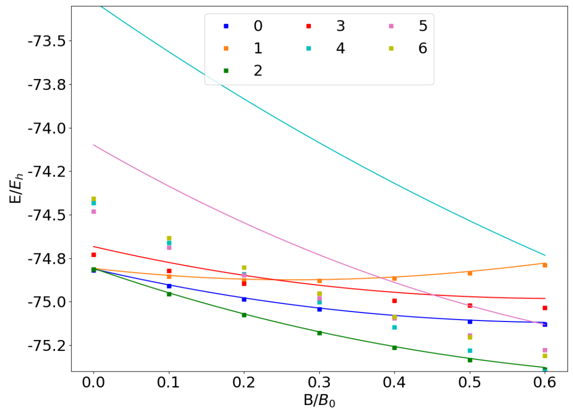

Figure S216: Total energies of all considered states of the O atom in the cc-pVTZ basis set in fully uncontracted form (solid lines). The FEM values are shown by the squares of the same color.

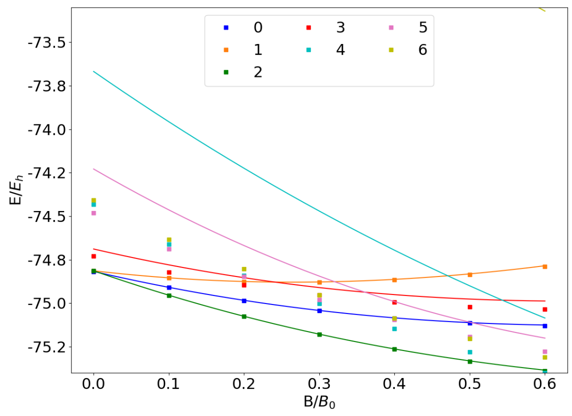

Figure S217: Total energies of all considered states of the O atom in the cc-pVQZ basis set in fully uncontracted form (solid lines). The FEM values are shown by the squares of the same color.

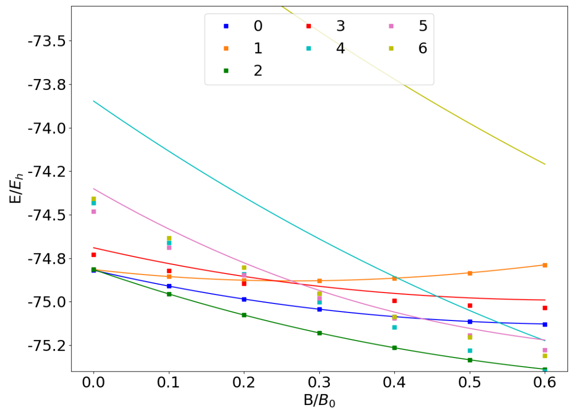

Figure S218: Total energies of all considered states of the O atom in the cc-pV5Z basis set in fully uncontracted form (solid lines). The FEM values are shown by the squares of the same color.

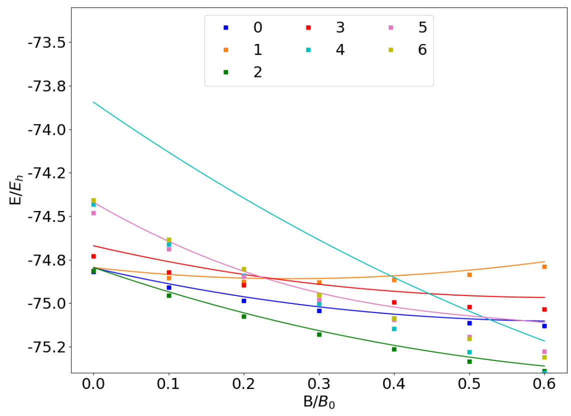

Figure S219: Total energies of all considered states of the O atom in the aug-cc-pVDZ basis set in fully uncontracted form (solid lines). The FEM values are shown by the squares of the same color.

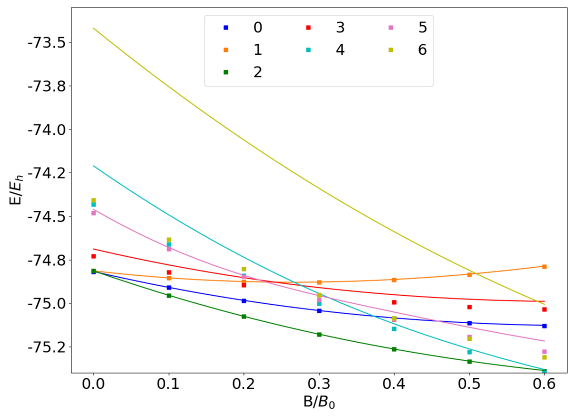

Figure S221: Total energies of all considered states of the O atom in the aug-cc-pVQZ basis set in fully uncontracted form (solid lines). The FEM values are shown by the squares of the same color.

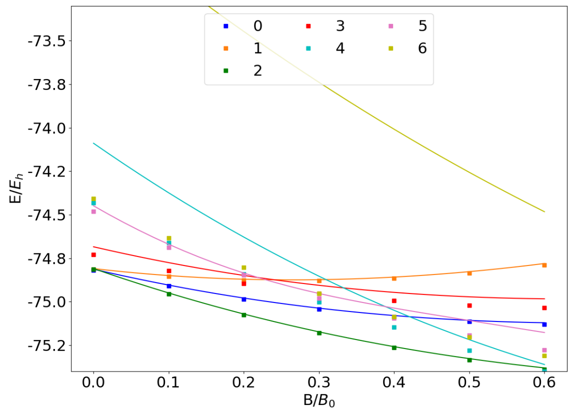

Figure S220: Total energies of all considered states of the O atom in the aug-cc-pVTZ basis set in fully uncontracted form (solid lines). The FEM values are shown by the squares of the same color.

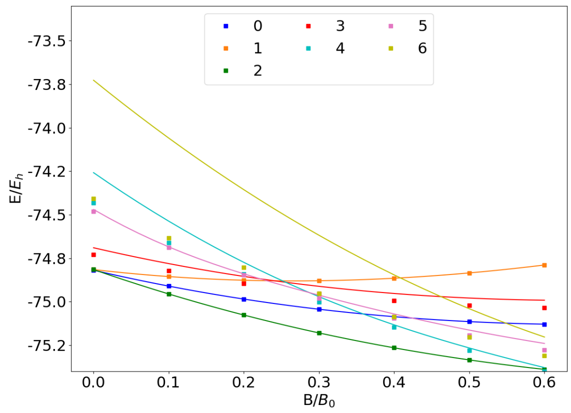

Figure S222: Total energies of all considered states of the O atom in the aug-cc-pV5Z basis set in fully uncontracted form (solid lines). The FEM values are shown by the squares of the same color.

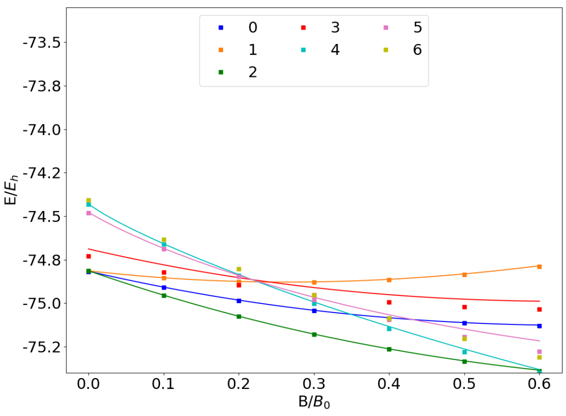

Figure S223: Total energies of all considered states of the O atom in the HGBSP1-5 basis set in fully uncontracted form (solid lines). The FEM values are shown by the squares of the same color.

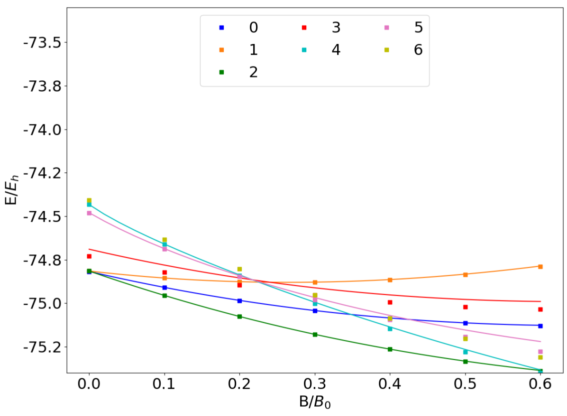

Figure S225: Total energies of all considered states of the O atom in the HGBSP1-9 basis set in fully uncontracted form (solid lines). The FEM values are shown by the squares of the same color.

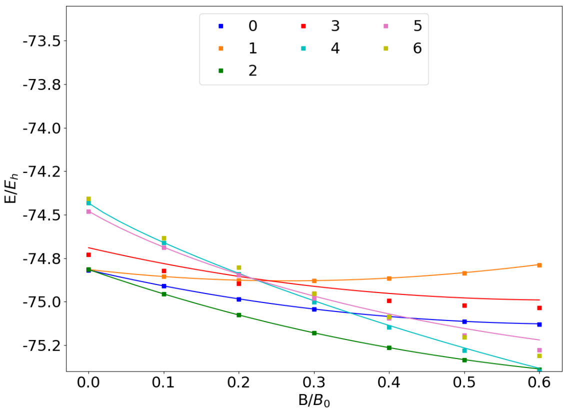

Figure S224: Total energies of all considered states of the O atom in the HGBSP1-7 basis set in fully uncontracted form (solid lines). The FEM values are shown by the squares of the same color.

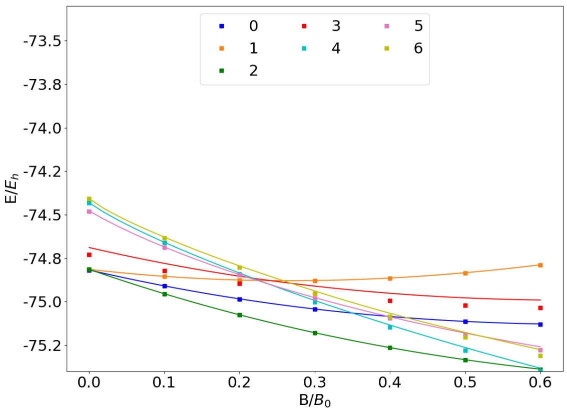

Figure S226: Total energies of all considered states of the O atom in the HGBSP2-5 basis set in fully uncontracted form (solid lines). The FEM values are shown by the squares of the same color.

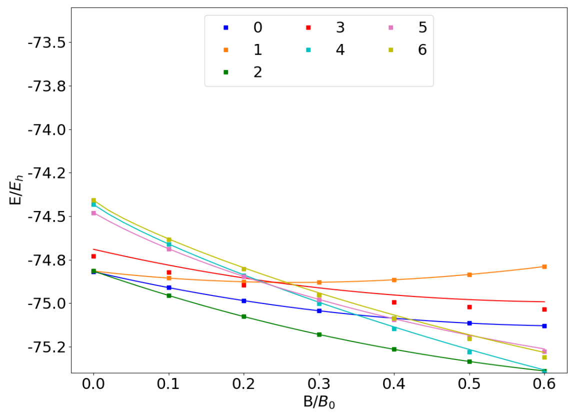

Figure S227: Total energies of all considered states of the O atom in the HGBSP2-7 basis set in fully uncontracted form (solid lines). The FEM values are shown by the squares of the same color.

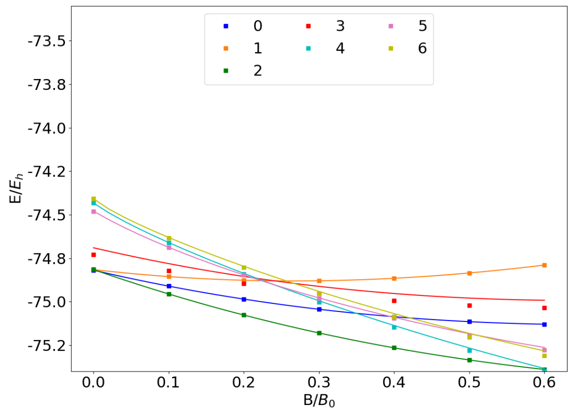

Figure S228: Total energies of all considered states of the O atom in the HGBSP2-9 basis set in fully uncontracted form (solid lines). The FEM values are shown by the squares of the same color.

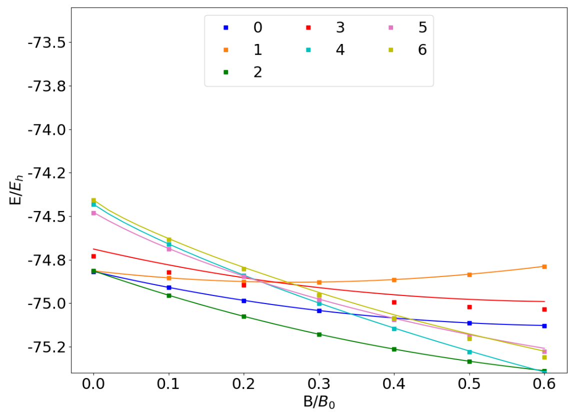

Figure S229: Total energies of all considered states of the O atom in the HGBSP3-5 basis set in fully uncontracted form (solid lines). The FEM values are shown by the squares of the same color.

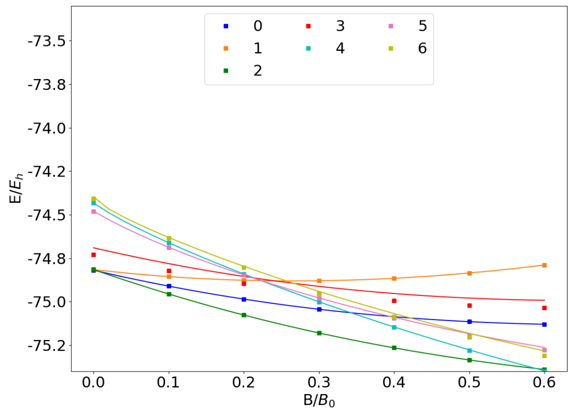

Figure S230: Total energies of all considered states of the O atom in the HGBSP3-7 basis set in fully uncontracted form (solid lines). The FEM values are shown by the squares of the same color.

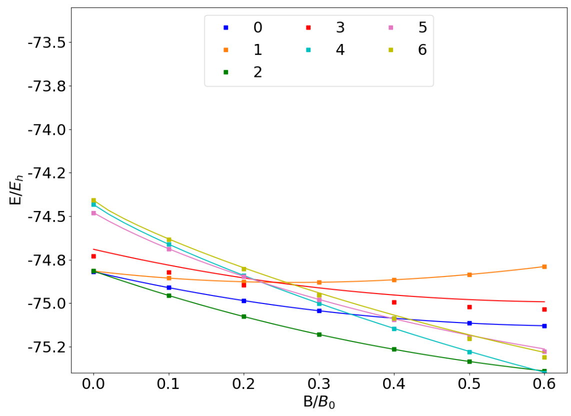

Figure S231: Total energies of all considered states of the O atom in the HGBSP3-9 basis set in fully uncontracted form (solid lines). The FEM values are shown by the squares of the same color.

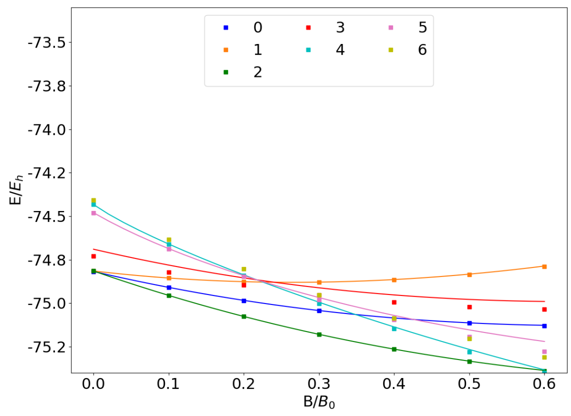

Figure S233: Total energies of all considered states of the O atom in the AHGBSP1-7 basis set in fully uncontracted form (solid lines). The FEM values are shown by the squares of the same color.

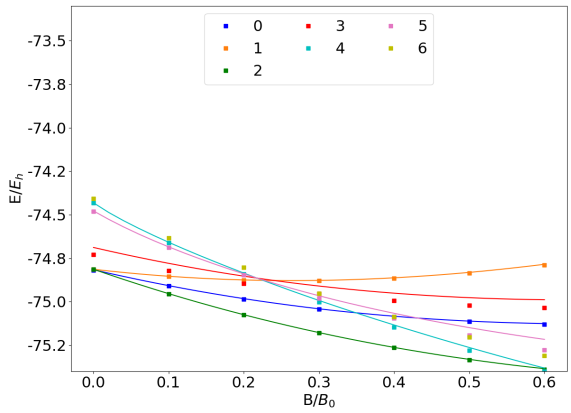

Figure S232: Total energies of all considered states of the O atom in the AHGBSP1-5 basis set in fully uncontracted form (solid lines). The FEM values are shown by the squares of the same color.

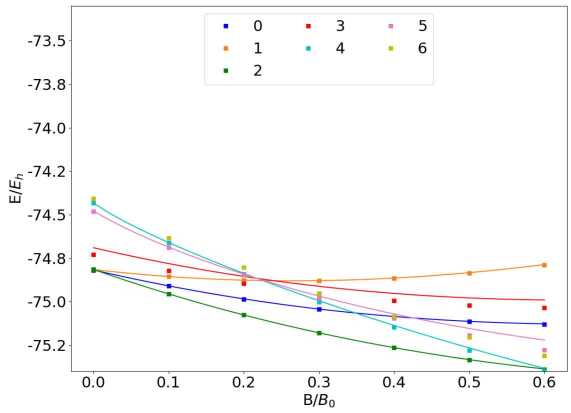

Figure S234: Total energies of all considered states of the O atom in the AHGBSP1-9 basis set in fully uncontracted form (solid lines). The FEM values are shown by the squares of the same color.

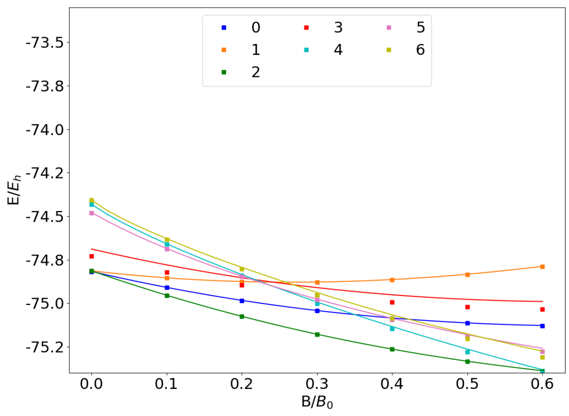

Figure S235: Total energies of all considered states of the O atom in the AHGBSP2-5 basis set in fully uncontracted form (solid lines). The FEM values are shown by the squares of the same color.

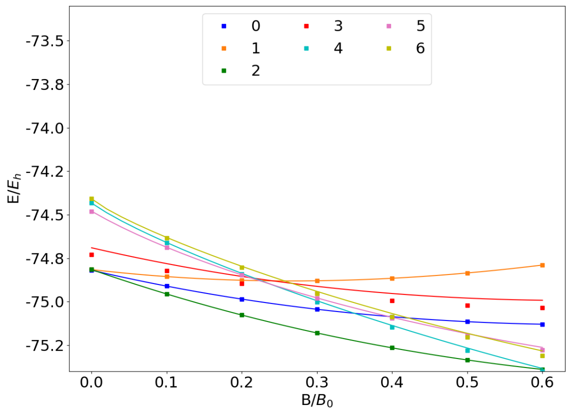

Figure S236: Total energies of all considered states of the O atom in the AHGBSP2-7 basis set in fully uncontracted form (solid lines). The FEM values are shown by the squares of the same color.

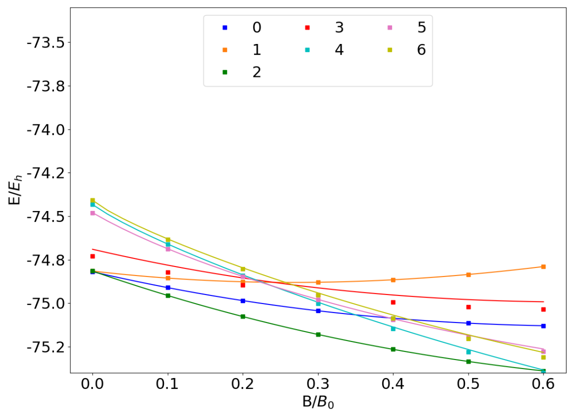

Figure S237: Total energies of all considered states of the O atom in the AHGBSP2-9 basis set in fully uncontracted form (solid lines). The FEM values are shown by the squares of the same color.

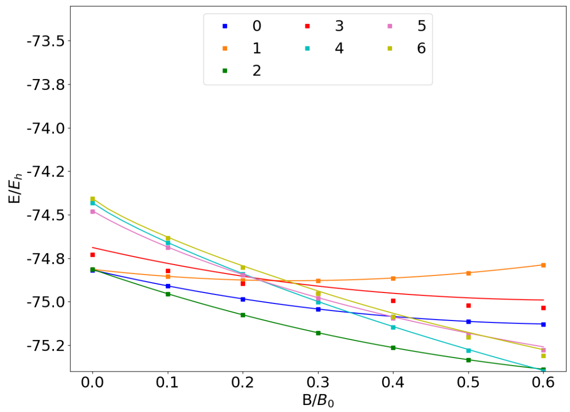

Figure S238: Total energies of all considered states of the O atom in the AHGBSP3-5 basis set in fully uncontracted form (solid lines). The FEM values are shown by the squares of the same color.

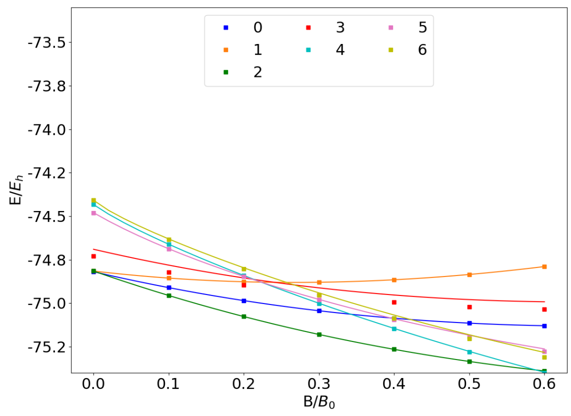

Figure S239: Total energies of all considered states of the O atom in the AHGBSP3-7 basis set in fully uncontracted form (solid lines). The FEM values are shown by the squares of the same color.

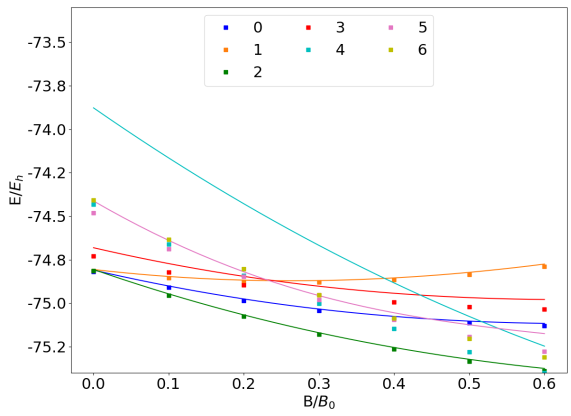

Figure S241: Total energies of all considered states of the O atom in the 6-311++G(3df,3pd) basis set in fully uncontracted form (solid lines). The FEM values are shown by the squares of the same color.

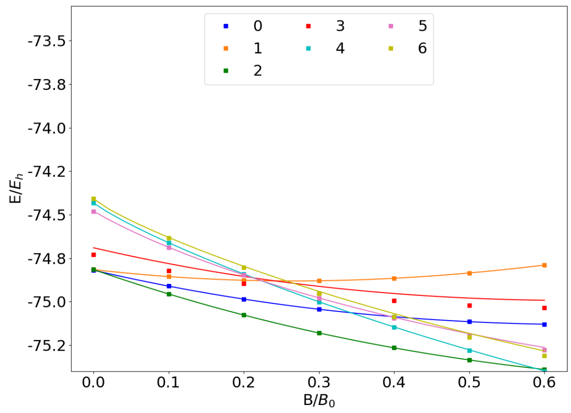

Figure S240: Total energies of all considered states of the O atom in the AHGBSP3-9 basis set in fully uncontracted form (solid lines). The FEM values are shown by the squares of the same color.

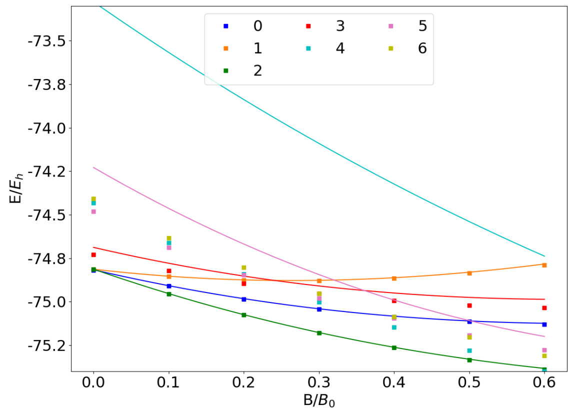

Figure S242: Total energies of all considered states of the O atom in the def2-TZVP basis set in fully uncontracted form (solid lines). The FEM values are shown by the squares of the same color.

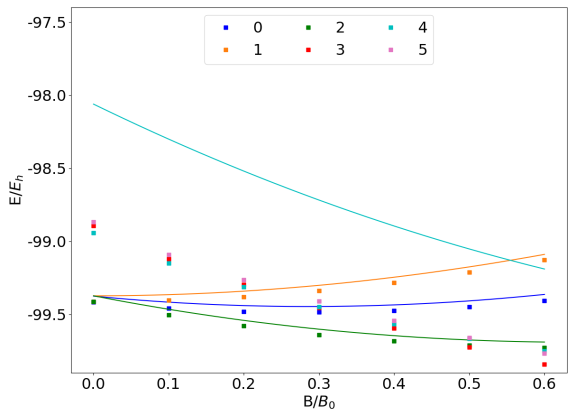

Figure S243: Total energies of all considered states of the F atom in the cc-pVDZ basis set in fully uncontracted form (solid lines). The FEM values are shown by the squares of the same color.

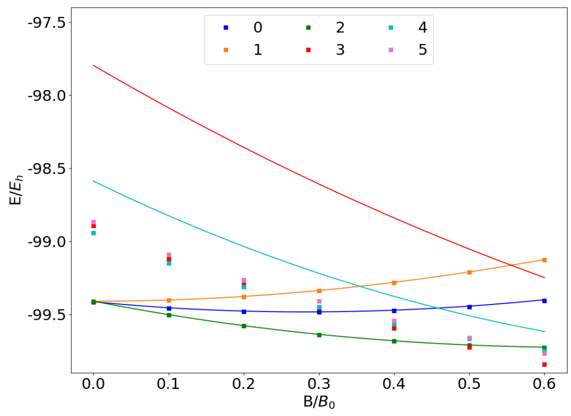

Figure S245: Total energies of all considered states of the F atom in the cc-pVQZ basis set in fully uncontracted form (solid lines). The FEM values are shown by the squares of the same color.

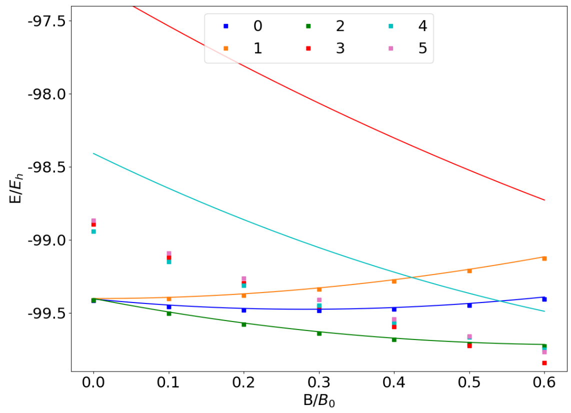

Figure S244: Total energies of all considered states of the F atom in the cc-pVTZ basis set in fully uncontracted form (solid lines). The FEM values are shown by the squares of the same color.

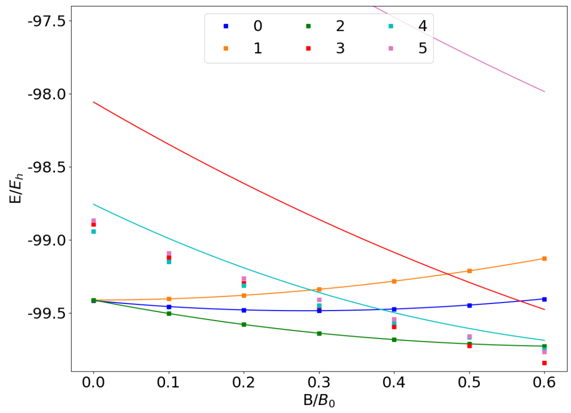

Figure S246: Total energies of all considered states of the F atom in the cc-pV5Z basis set in fully uncontracted form (solid lines). The FEM values are shown by the squares of the same color.

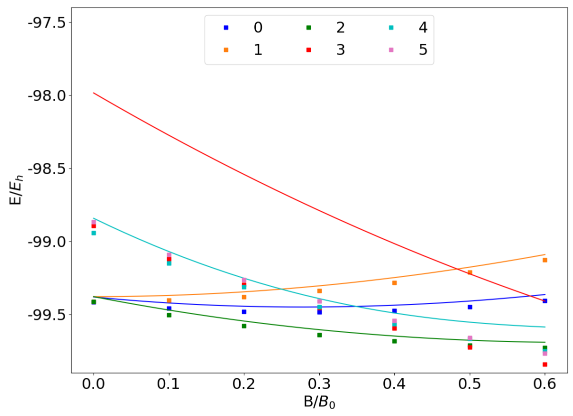

Figure S247: Total energies of all considered states of the F atom in the aug-cc-pVDZ basis set in fully uncontracted form (solid lines). The FEM values are shown by the squares of the same color.

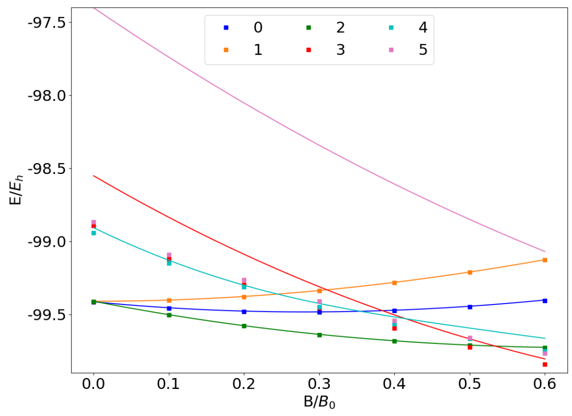

Figure S249: Total energies of all considered states of the F atom in the aug-cc-pVQZ basis set in fully uncontracted form (solid lines). The FEM values are shown by the squares of the same color.

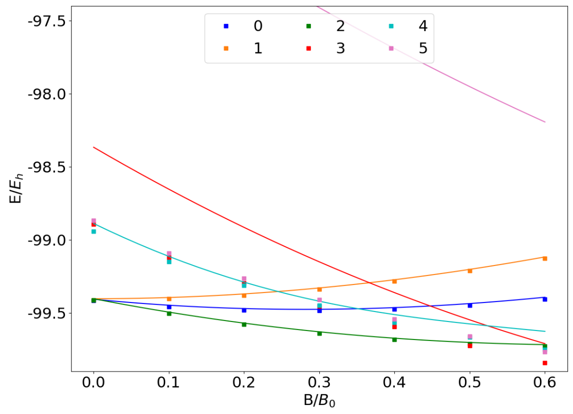

Figure S248: Total energies of all considered states of the F atom in the aug-cc-pVTZ basis set in fully uncontracted form (solid lines). The FEM values are shown by the squares of the same color.

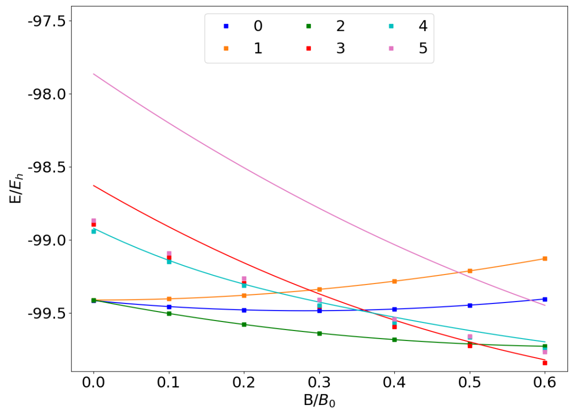

Figure S250: Total energies of all considered states of the F atom in the aug-cc-pV5Z basis set in fully uncontracted form (solid lines). The FEM values are shown by the squares of the same color.

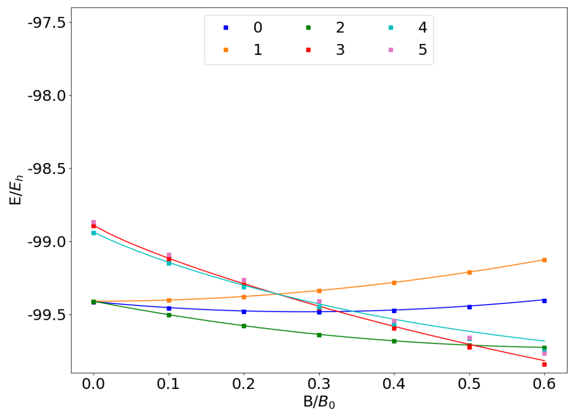

Figure S251: Total energies of all considered states of the F atom in the HGBSP1-5 basis set in fully uncontracted form (solid lines). The FEM values are shown by the squares of the same color.

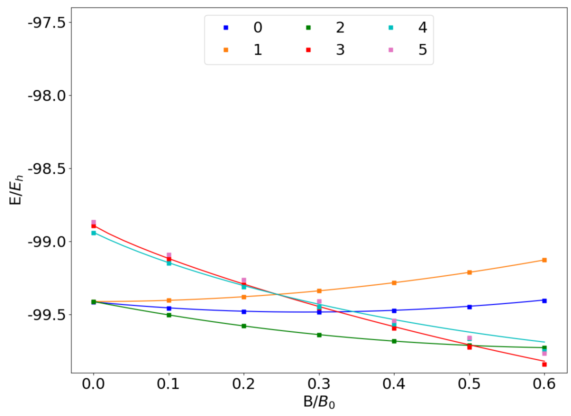

Figure S253: Total energies of all considered states of the F atom in the HGBSP1-9 basis set in fully uncontracted form (solid lines). The FEM values are shown by the squares of the same color.

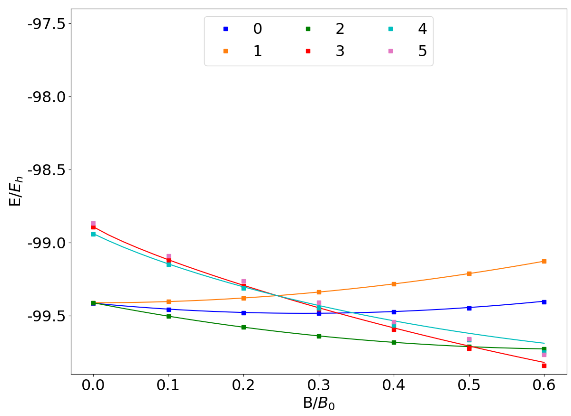

Figure S252: Total energies of all considered states of the F atom in the HGBSP1-7 basis set in fully uncontracted form (solid lines). The FEM values are shown by the squares of the same color.

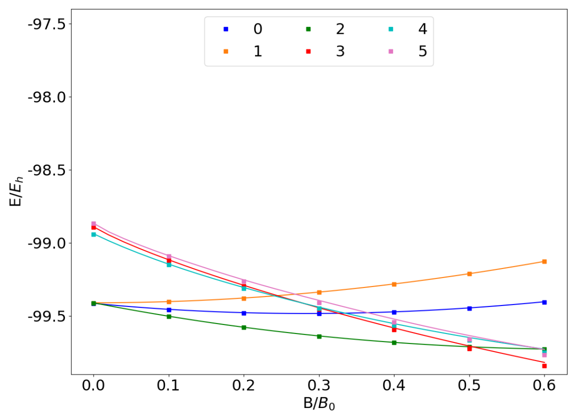

Figure S254: Total energies of all considered states of the F atom in the HGBSP2-5 basis set in fully uncontracted form (solid lines). The FEM values are shown by the squares of the same color.

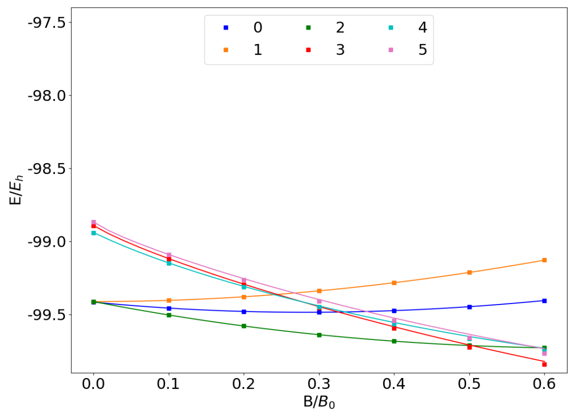

Figure S255: Total energies of all considered states of the F atom in the HGBSP2-7 basis set in fully uncontracted form (solid lines). The FEM values are shown by the squares of the same color.

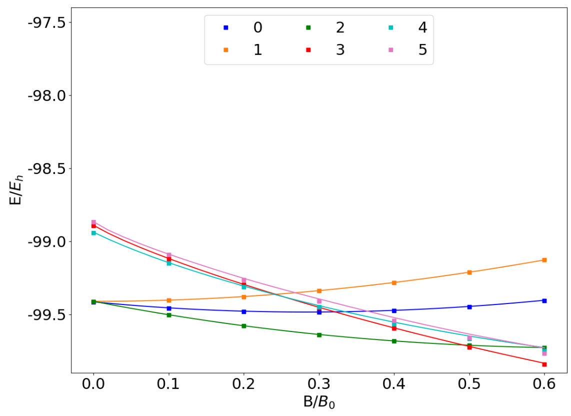

Figure S257: Total energies of all considered states of the F atom in the HGBSP3-5 basis set in fully uncontracted form (solid lines). The FEM values are shown by the squares of the same color.

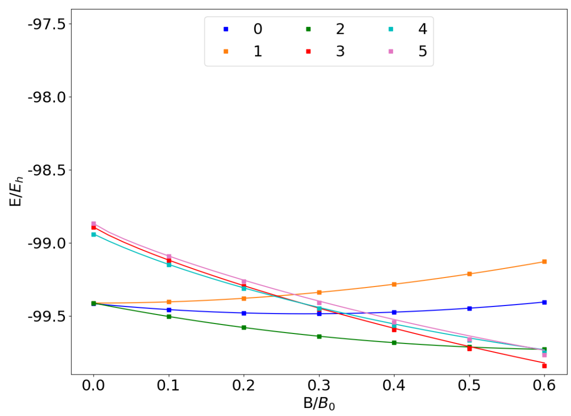

Figure S256: Total energies of all considered states of the F atom in the HGBSP2-9 basis set in fully uncontracted form (solid lines). The FEM values are shown by the squares of the same color.

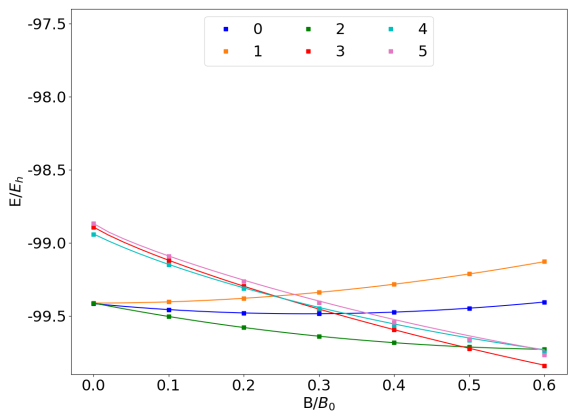

Figure S258: Total energies of all considered states of the F atom in the HGBSP3-7 basis set in fully uncontracted form (solid lines). The FEM values are shown by the squares of the same color.

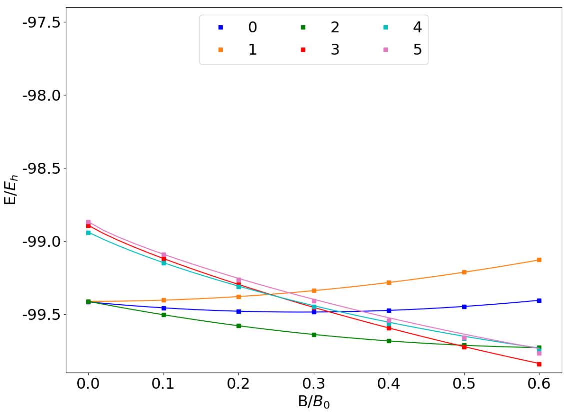

Figure S259: Total energies of all considered states of the F atom in the HGBSP3-9 basis set in fully uncontracted form (solid lines). The FEM values are shown by the squares of the same color.

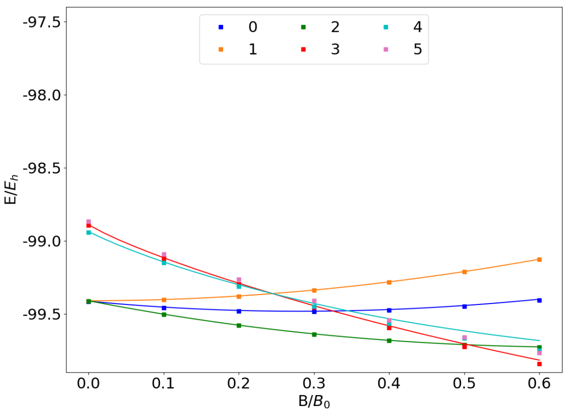

Figure S260: Total energies of all considered states of the F atom in the AHGBSP1-5 basis set in fully uncontracted form (solid lines). The FEM values are shown by the squares of the same color.

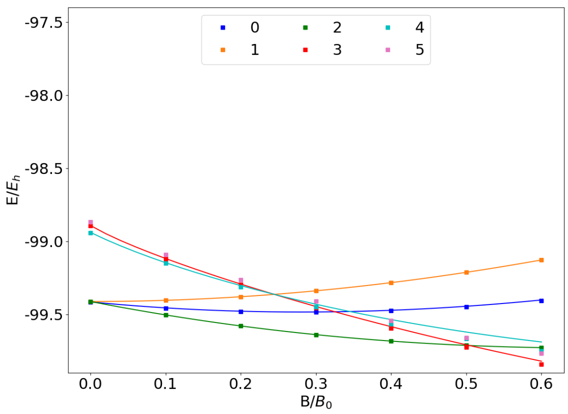

Figure S261: Total energies of all considered states of the F atom in the AHGBSP1-7 basis set in fully uncontracted form (solid lines). The FEM values are shown by the squares of the same color.

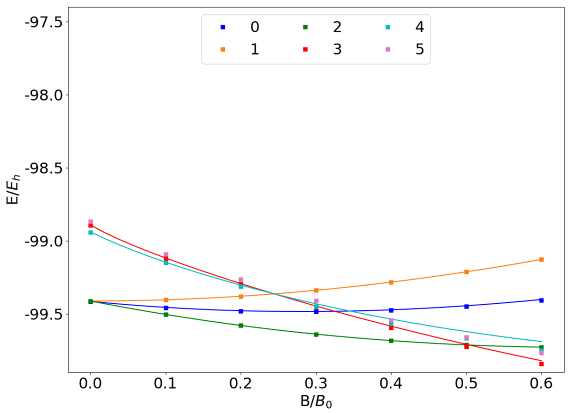

Figure S262: Total energies of all considered states of the F atom in the AHGBSP1-9 basis set in fully uncontracted form (solid lines). The FEM values are shown by the squares of the same color.

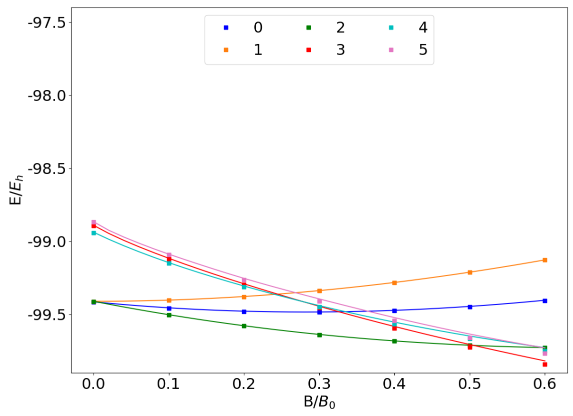

Figure S263: Total energies of all considered states of the F atom in the AHGBSP2-5 basis set in fully uncontracted form (solid lines). The FEM values are shown by the squares of the same color.

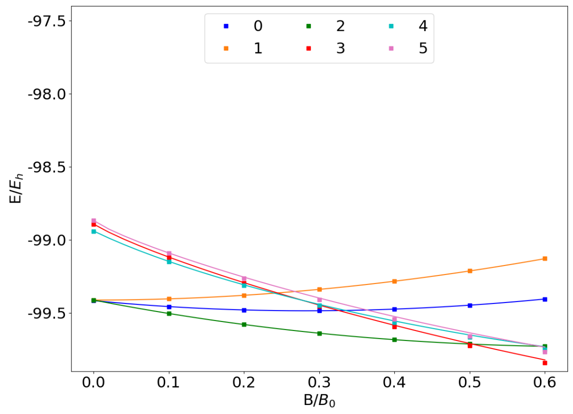

Figure S264: Total energies of all considered states of the F atom in the AHGBSP2-7 basis set in fully uncontracted form (solid lines). The FEM values are shown by the squares of the same color.

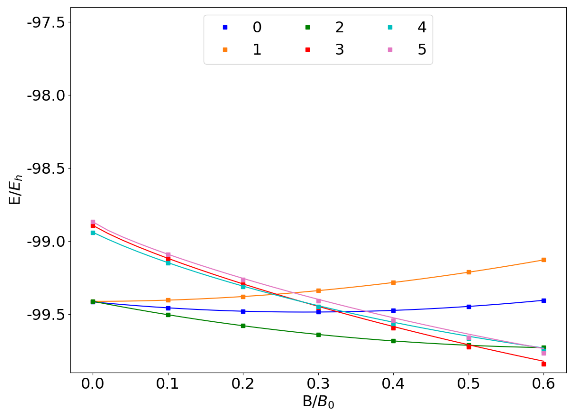

Figure S265: Total energies of all considered states of the F atom in the AHGBSP2-9 basis set in fully uncontracted form (solid lines). The FEM values are shown by the squares of the same color.

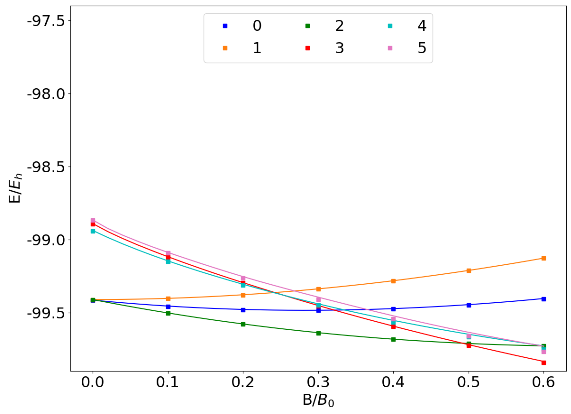

Figure S266: Total energies of all considered states of the F atom in the AHGBSP3-5 basis set in fully uncontracted form (solid lines). The FEM values are shown by the squares of the same color.

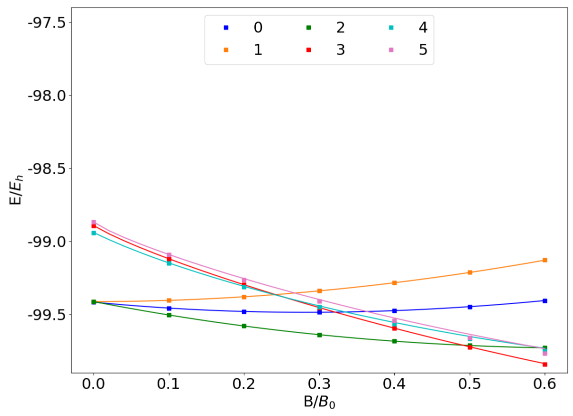

Figure S267: Total energies of all considered states of the F atom in the AHGBSP3-7 basis set in fully uncontracted form (solid lines). The FEM values are shown by the squares of the same color.

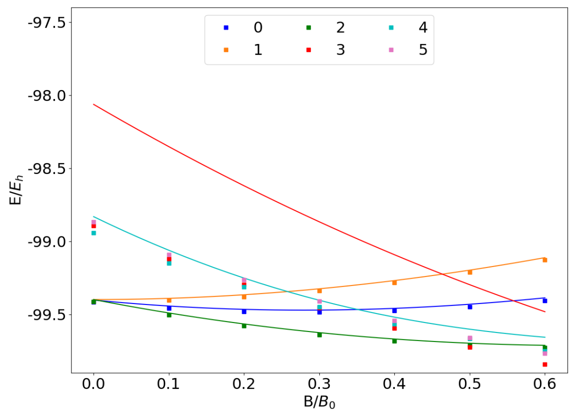

Figure S269: Total energies of all considered states of the F atom in the 6-311++G(3df,3pd) basis set in fully uncontracted form (solid lines). The FEM values are shown by the squares of the same color.

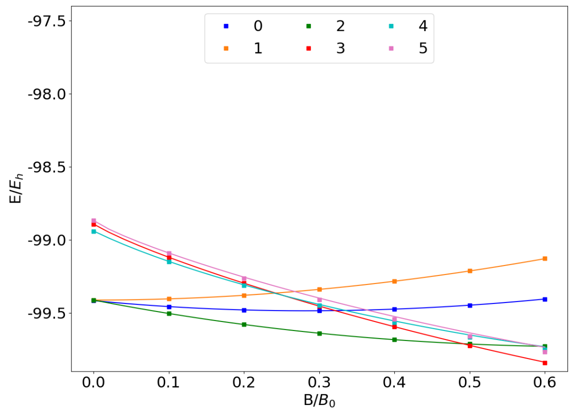

Figure S268: Total energies of all considered states of the F atom in the AHGBSP3-9 basis set in fully uncontracted form (solid lines). The FEM values are shown by the squares of the same color.

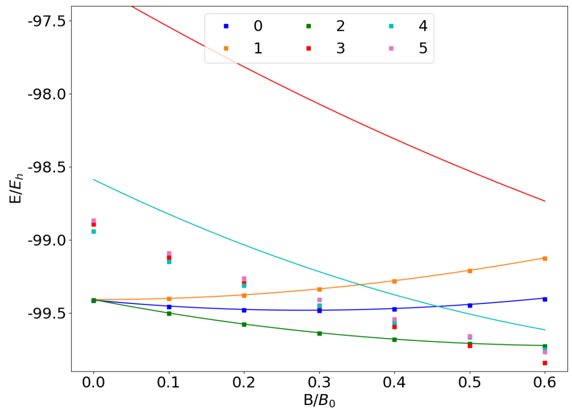

Figure S270: Total energies of all considered states of the F atom in the def2-TZVP basis set in fully uncontracted form (solid lines). The FEM values are shown by the squares of the same color.

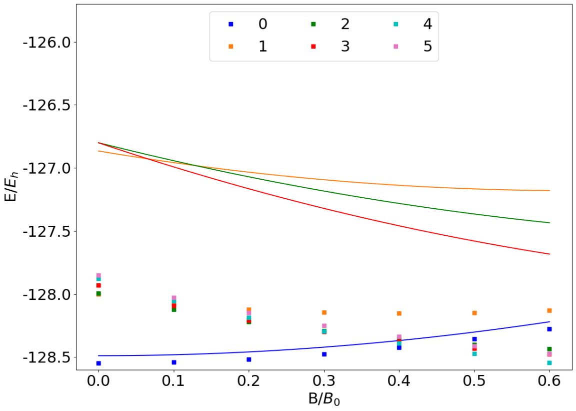

Figure S271: Total energies of all considered states of the Ne atom in the cc-pVDZ basis set in fully uncontracted form (solid lines). The FEM values are shown by the squares of the same color.

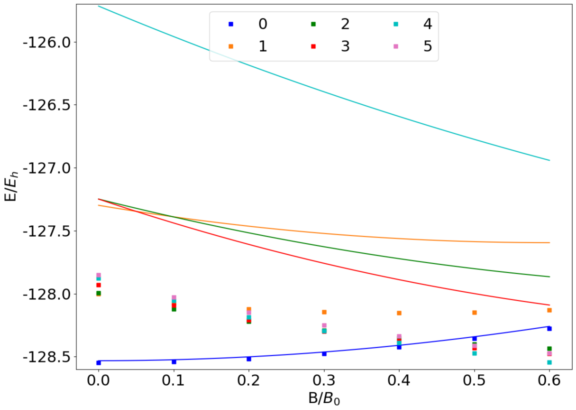

Figure S272: Total energies of all considered states of the Ne atom in the cc-pVTZ basis set in fully uncontracted form (solid lines). The FEM values are shown by the squares of the same color.

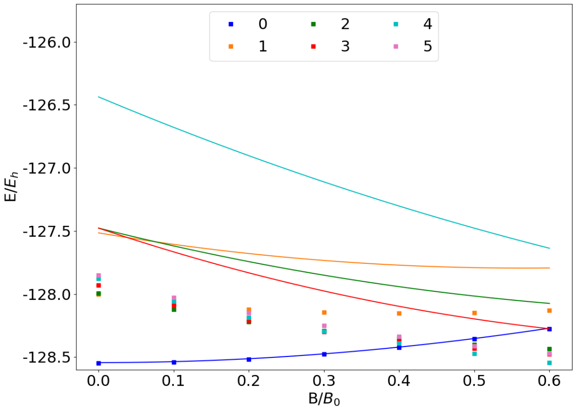

Figure S273: Total energies of all considered states of the Ne atom in the cc-pVQZ basis set in fully uncontracted form (solid lines). The FEM values are shown by the squares of the same color.

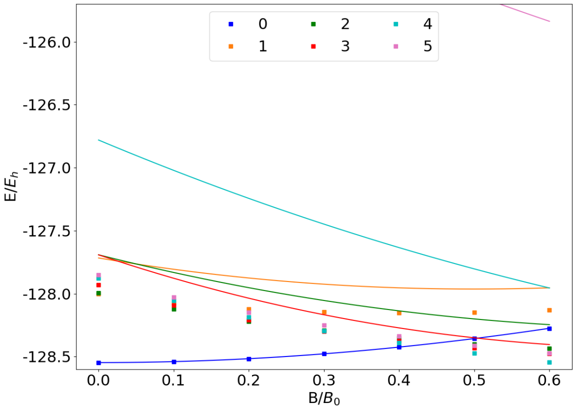

Figure S274: Total energies of all considered states of the Ne atom in the cc-pV5Z basis set in fully uncontracted form (solid lines). The FEM values are shown by the squares of the same color.

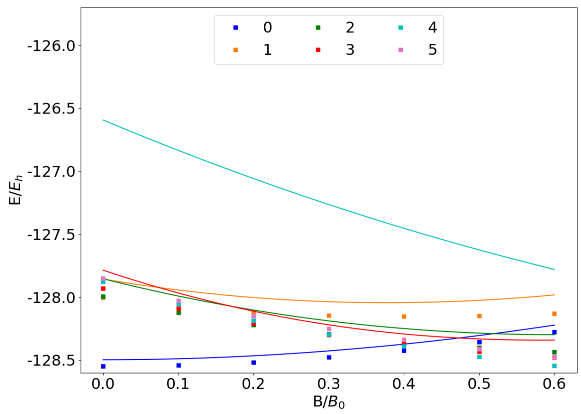

Figure S275: Total energies of all considered states of the Ne atom in the aug-cc-pVDZ basis set in fully uncontracted form (solid lines). The FEM values are shown by the squares of the same color.

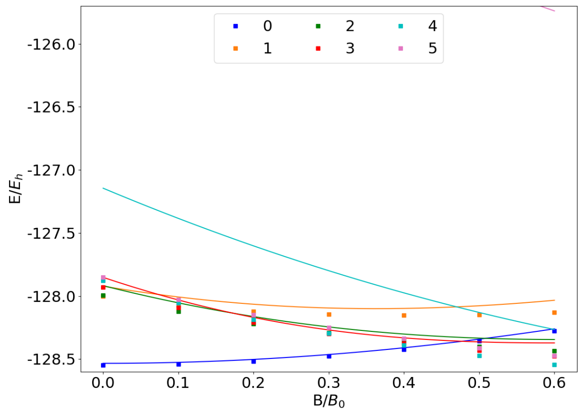

Figure S276: Total energies of all considered states of the Ne atom in the aug-cc-pVTZ basis set in fully uncontracted form (solid lines). The FEM values are shown by the squares of the same color.

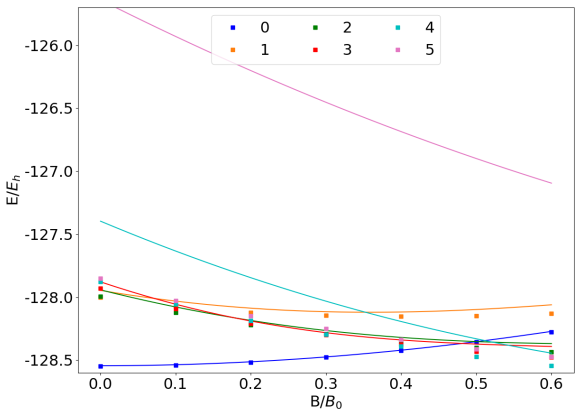

Figure S277: Total energies of all considered states of the Ne atom in the aug-cc-pVQZ basis set in fully uncontracted form (solid lines). The FEM values are shown by the squares of the same color.

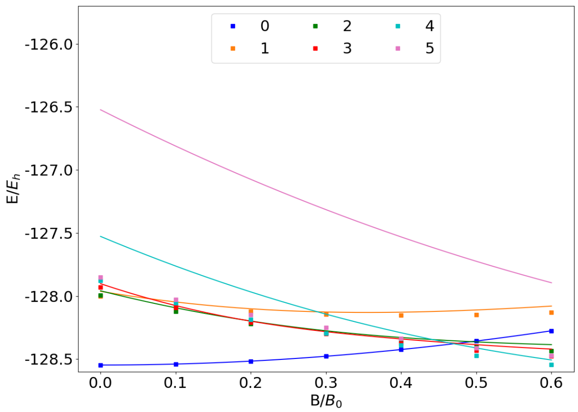

Figure S278: Total energies of all considered states of the Ne atom in the aug-cc-pV5Z basis set in fully uncontracted form (solid lines). The FEM values are shown by the squares of the same color.

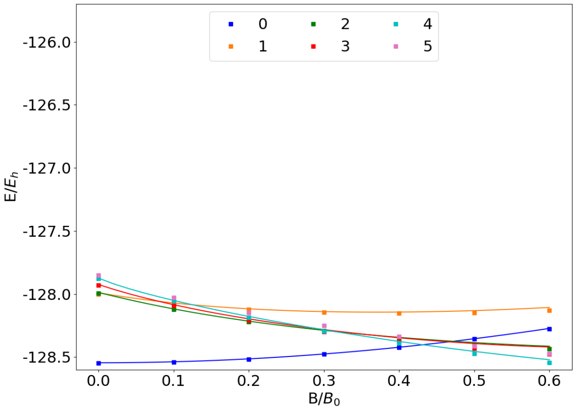

Figure S279: Total energies of all considered states of the Ne atom in the HGBSP1-5 basis set in fully uncontracted form (solid lines). The FEM values are shown by the squares of the same color.

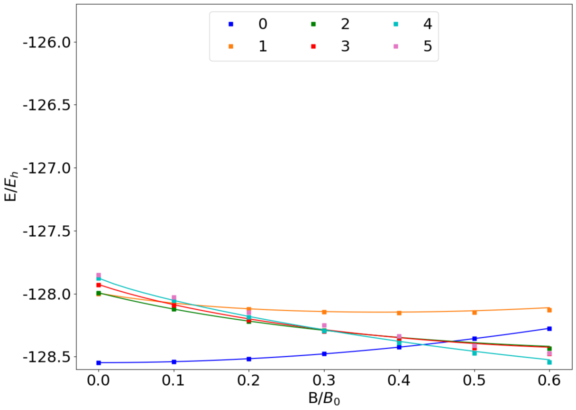

Figure S280: Total energies of all considered states of the Ne atom in the HGBSP1-7 basis set in fully uncontracted form (solid lines). The FEM values are shown by the squares of the same color.

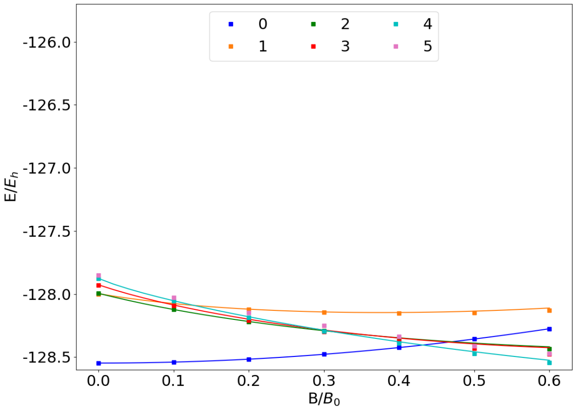

Figure S281: Total energies of all considered states of the Ne atom in the HGBSP1-9 basis set in fully uncontracted form (solid lines). The FEM values are shown by the squares of the same color.

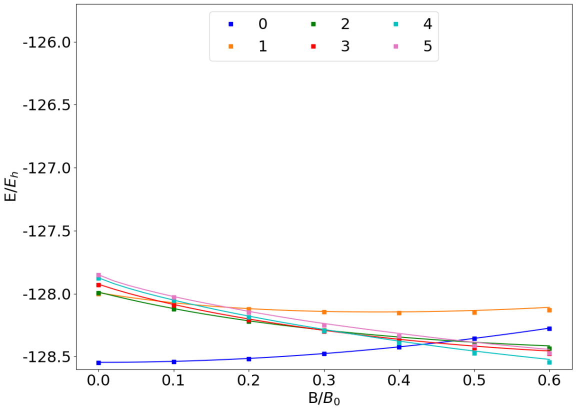

Figure S282: Total energies of all considered states of the Ne atom in the HGBSP2-5 basis set in fully uncontracted form (solid lines). The FEM values are shown by the squares of the same color.

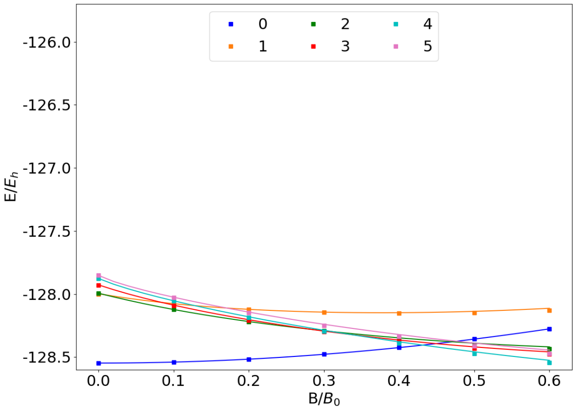

Figure S283: Total energies of all considered states of the Ne atom in the HGBSP2-7 basis set in fully uncontracted form (solid lines). The FEM values are shown by the squares of the same color.

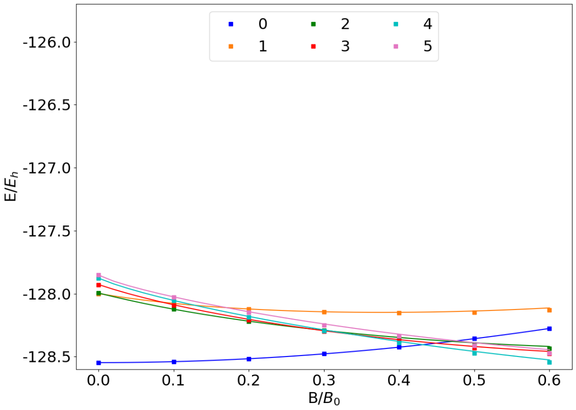

Figure S284: Total energies of all considered states of the Ne atom in the HGBSP2-9 basis set in fully uncontracted form (solid lines). The FEM values are shown by the squares of the same color.

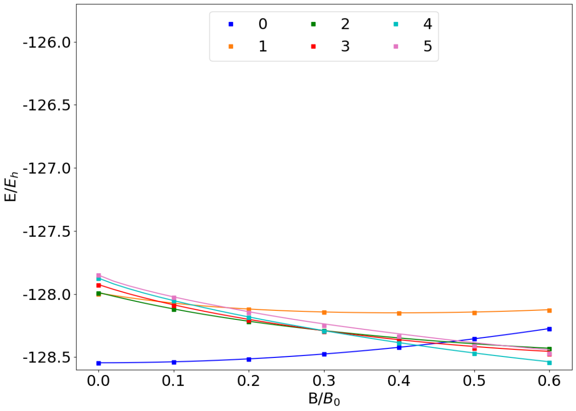

Figure S285: Total energies of all considered states of the Ne atom in the HGBSP3-5 basis set in fully uncontracted form (solid lines). The FEM values are shown by the squares of the same color.

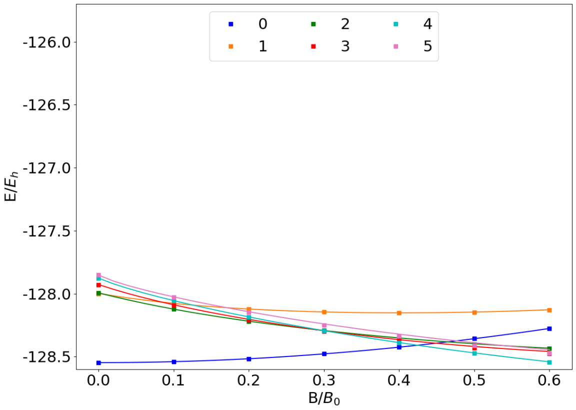

Figure S286: Total energies of all considered states of the Ne atom in the HGBSP3-7 basis set in fully uncontracted form (solid lines). The FEM values are shown by the squares of the same color.

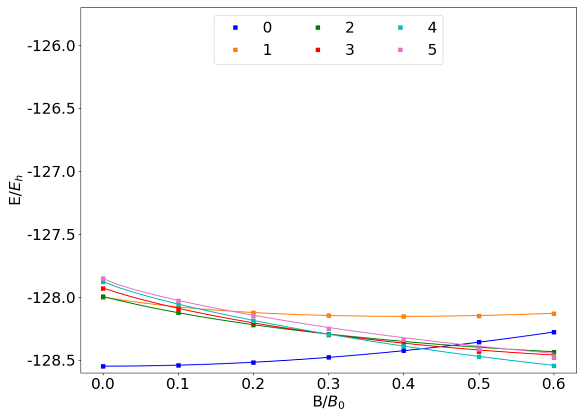

Figure S287: Total energies of all considered states of the Ne atom in the HGBSP3-9 basis set in fully uncontracted form (solid lines). The FEM values are shown by the squares of the same color.

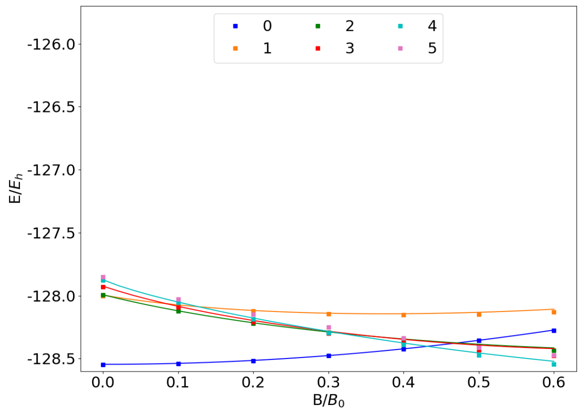

Figure S288: Total energies of all considered states of the Ne atom in the AHGBSP1-5 basis set in fully uncontracted form (solid lines). The FEM values are shown by the squares of the same color.

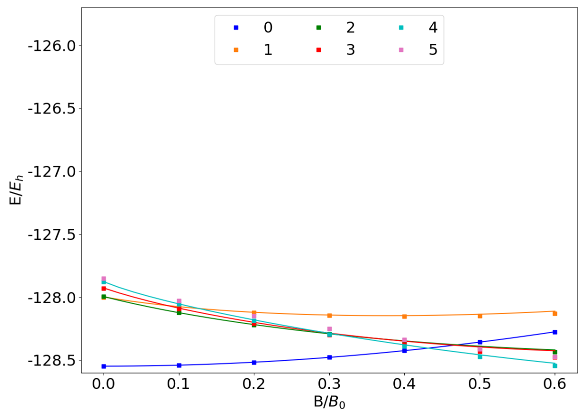

Figure S289: Total energies of all considered states of the Ne atom in the AHGBSP1-7 basis set in fully uncontracted form (solid lines). The FEM values are shown by the squares of the same color.

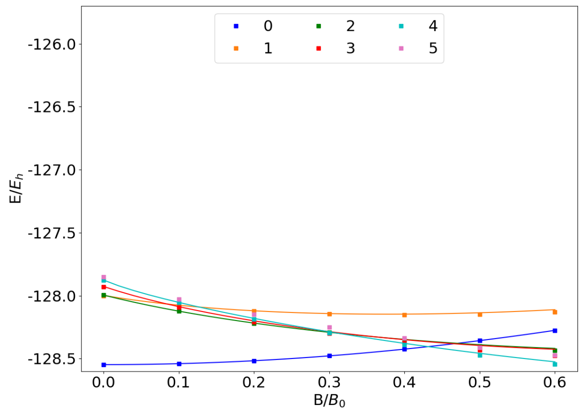

Figure S290: Total energies of all considered states of the Ne atom in the AHGBSP1-9 basis set in fully uncontracted form (solid lines). The FEM values are shown by the squares of the same color.

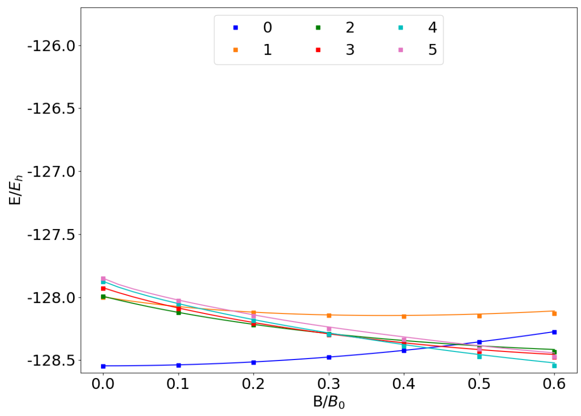

Figure S291: Total energies of all considered states of the Ne atom in the AHGBSP2-5 basis set in fully uncontracted form (solid lines). The FEM values are shown by the squares of the same color.

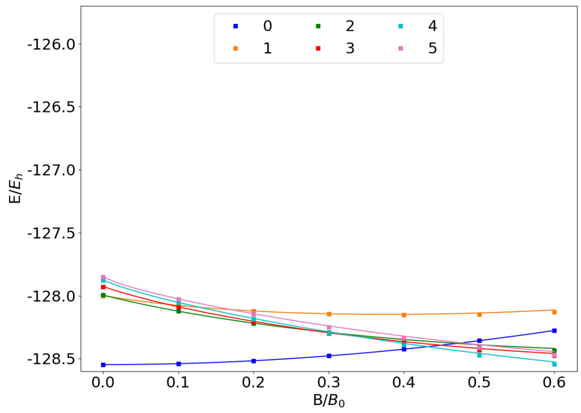

Figure S292: Total energies of all considered states of the Ne atom in the AHGBSP2-7 basis set in fully uncontracted form (solid lines). The FEM values are shown by the squares of the same color.

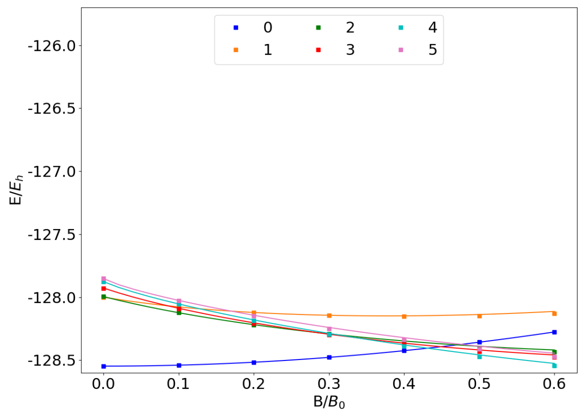

Figure S293: Total energies of all considered states of the Ne atom in the AHGBSP2-9 basis set in fully uncontracted form (solid lines). The FEM values are shown by the squares of the same color.

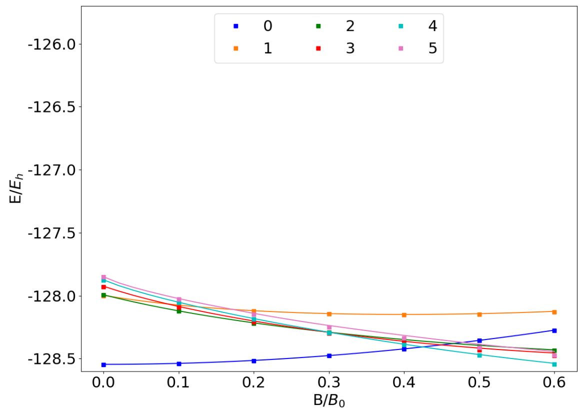

Figure S294: Total energies of all considered states of the Ne atom in the AHGBSP3-5 basis set in fully uncontracted form (solid lines). The FEM values are shown by the squares of the same color.

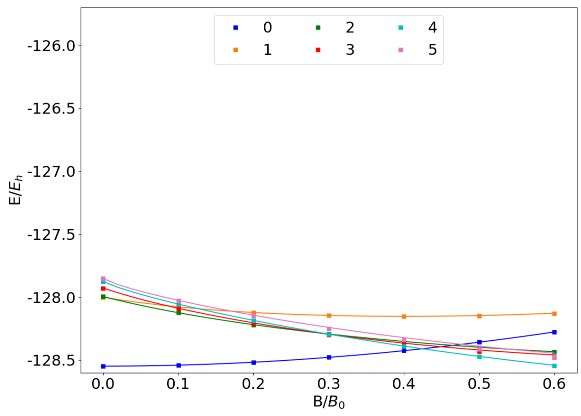

Figure S295: Total energies of all considered states of the Ne atom in the AHGBSP3-7 basis set in fully uncontracted form (solid lines). The FEM values are shown by the squares of the same color.

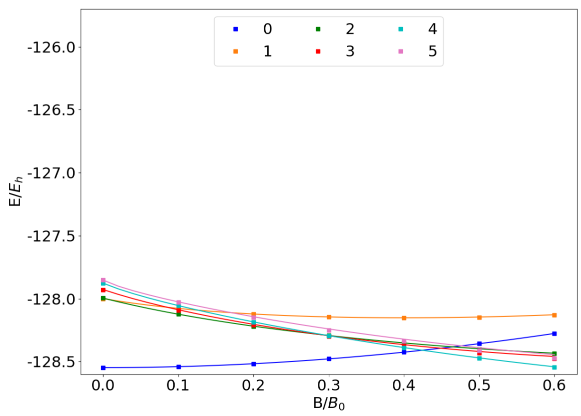

Figure S296: Total energies of all considered states of the Ne atom in the AHGBSP3-9 basis set in fully uncontracted form (solid lines). The FEM values are shown by the squares of the same color.

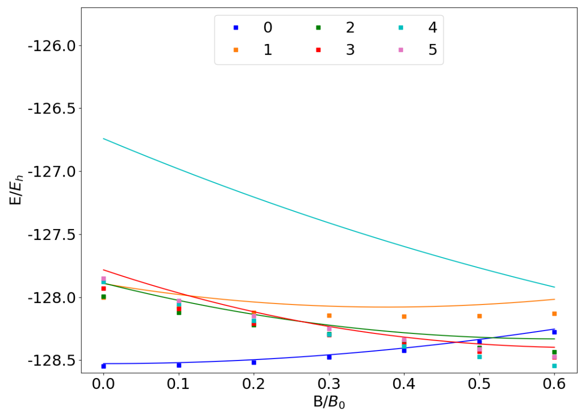

Figure S297: Total energies of all considered states of the Ne atom in the 6-311++G(3df,3pd) basis set in fully uncontracted form (solid lines). The FEM values are shown by the squares of the same color.

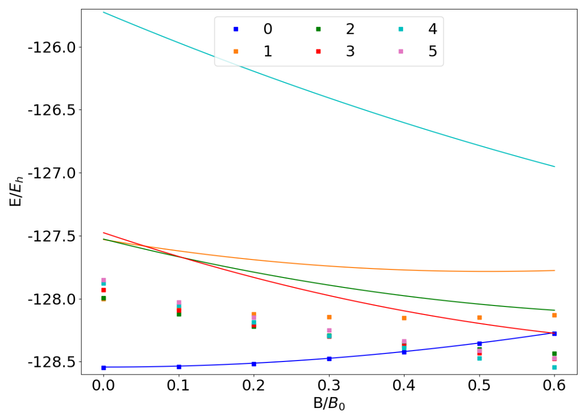

Figure S298: Total energies of all considered states of the Ne atom in the def2-TZVP basis set in fully uncontracted form (solid lines). The FEM values are shown by the squares of the same color.

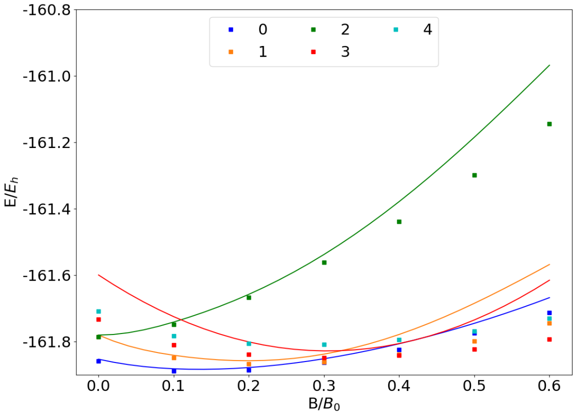

Figure S299: Total energies of all considered states of the Na atom in the cc-pVDZ basis set in fully uncontracted form (solid lines). The FEM values are shown by the squares of the same color.

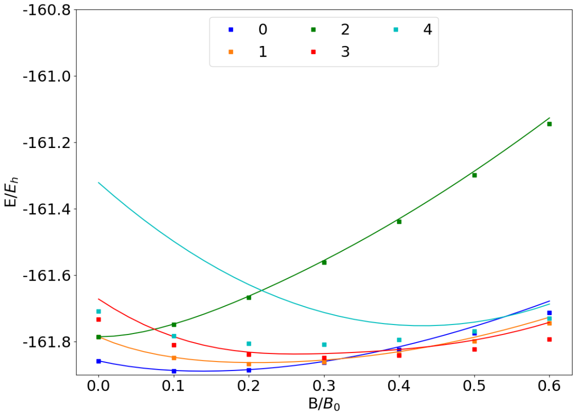

Figure S300: Total energies of all considered states of the Na atom in the cc-pVTZ basis set in fully uncontracted form (solid lines). The FEM values are shown by the squares of the same color.

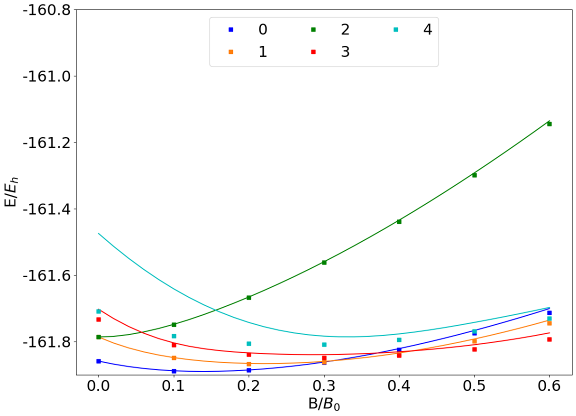

Figure S301: Total energies of all considered states of the Na atom in the cc-pVQZ basis set in fully uncontracted form (solid lines). The FEM values are shown by the squares of the same color.

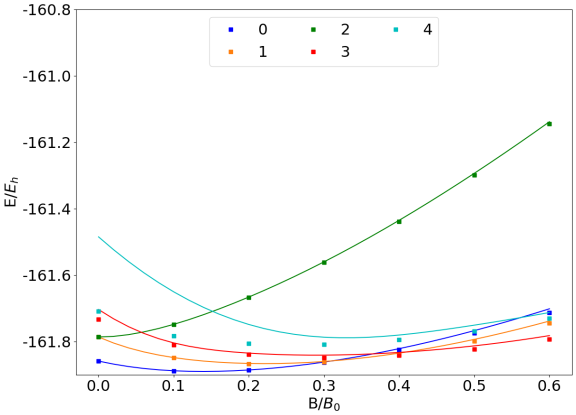

Figure S302: Total energies of all considered states of the Na atom in the cc-pV5Z basis set in fully uncontracted form (solid lines). The FEM values are shown by the squares of the same color.

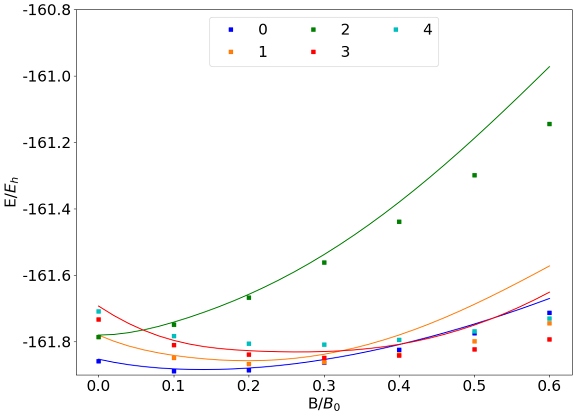

Figure S303: Total energies of all considered states of the Na atom in the aug-cc-pVDZ basis set in fully uncontracted form (solid lines). The FEM values are shown by the squares of the same color.

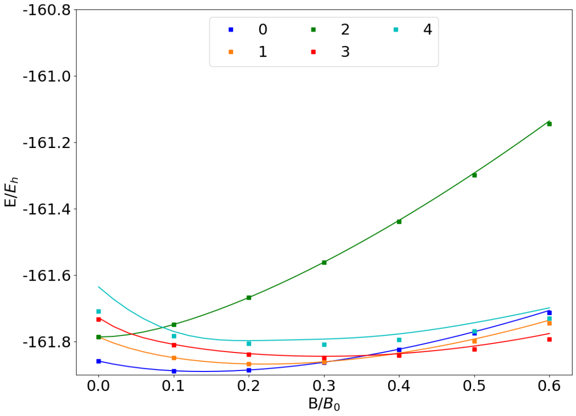

Figure S305: Total energies of all considered states of the Na atom in the aug-cc-pVQZ basis set in fully uncontracted form (solid lines). The FEM values are shown by the squares of the same color.

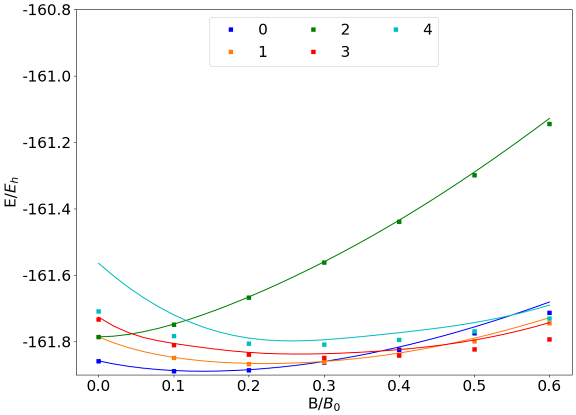

Figure S304: Total energies of all considered states of the Na atom in the aug-cc-pVTZ basis set in fully uncontracted form (solid lines). The FEM values are shown by the squares of the same color.

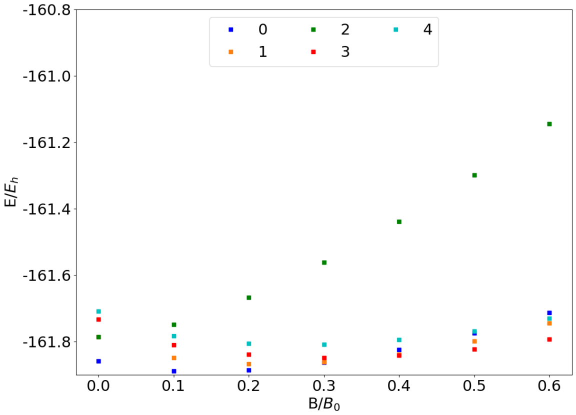

Figure S306: Total energies of all considered states of the Na atom in the aug-cc-pV5Z basis set in fully uncontracted form (solid lines). The FEM values are shown by the squares of the same color.

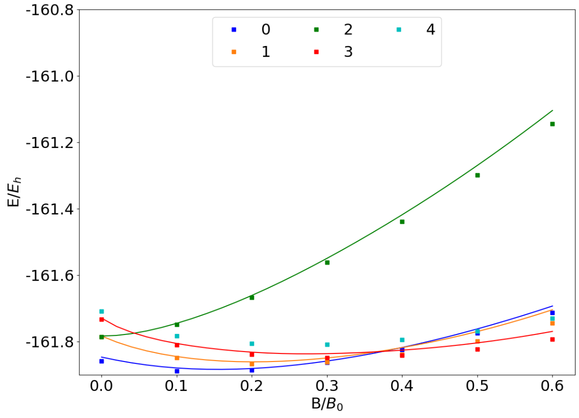

Figure S307: Total energies of all considered states of the Na atom in the HGBSP1-5 basis set in fully uncontracted form (solid lines). The FEM values are shown by the squares of the same color.

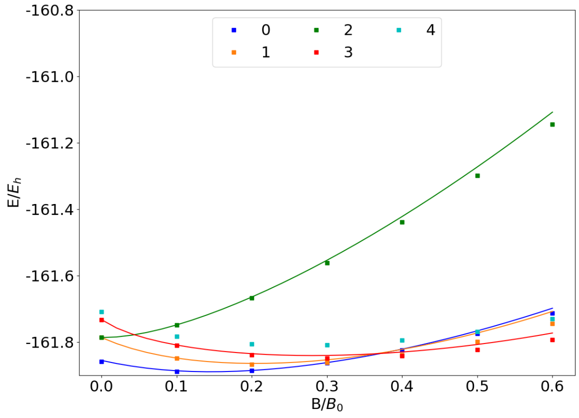

Figure S308: Total energies of all considered states of the Na atom in the HGBSP1-7 basis set in fully uncontracted form (solid lines). The FEM values are shown by the squares of the same color.

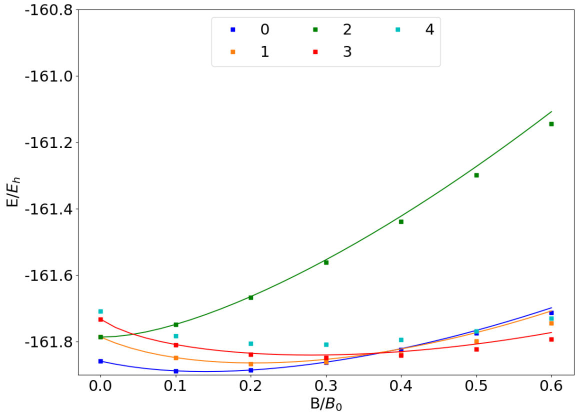

Figure S309: Total energies of all considered states of the Na atom in the HGBSP1-9 basis set in fully uncontracted form (solid lines). The FEM values are shown by the squares of the same color.

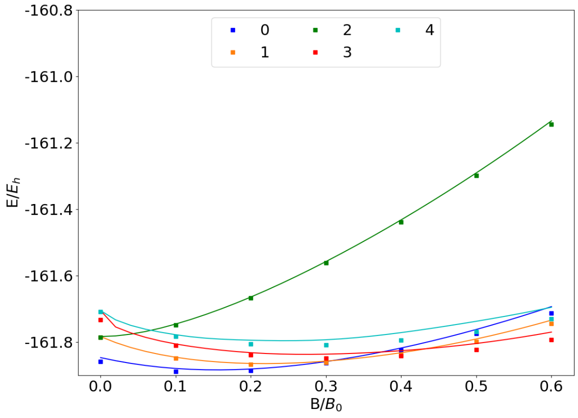

Figure S310: Total energies of all considered states of the Na atom in the HGBSP2-5 basis set in fully uncontracted form (solid lines). The FEM values are shown by the squares of the same color.

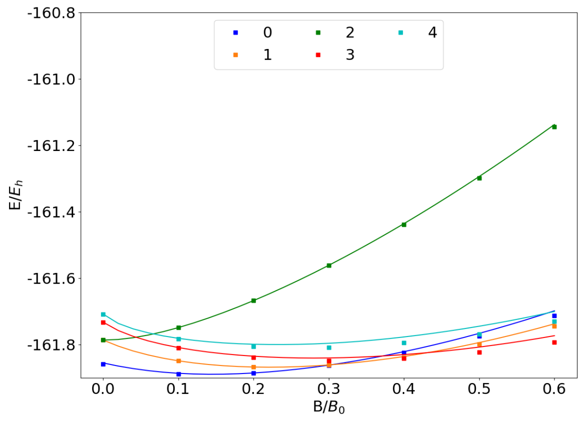

Figure S311: Total energies of all considered states of the Na atom in the HGBSP2-7 basis set in fully uncontracted form (solid lines). The FEM values are shown by the squares of the same color.

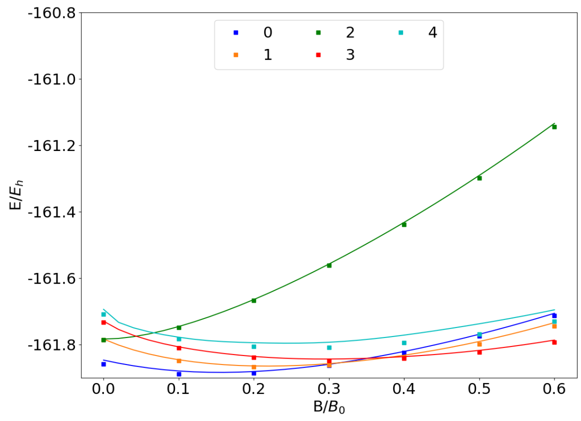

Figure S313: Total energies of all considered states of the Na atom in the HGBSP3-5 basis set in fully uncontracted form (solid lines). The FEM values are shown by the squares of the same color.

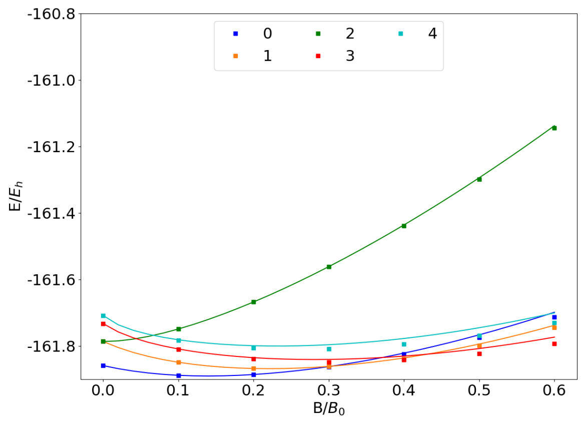

Figure S312: Total energies of all considered states of the Na atom in the HGBSP2-9 basis set in fully uncontracted form (solid lines). The FEM values are shown by the squares of the same color.

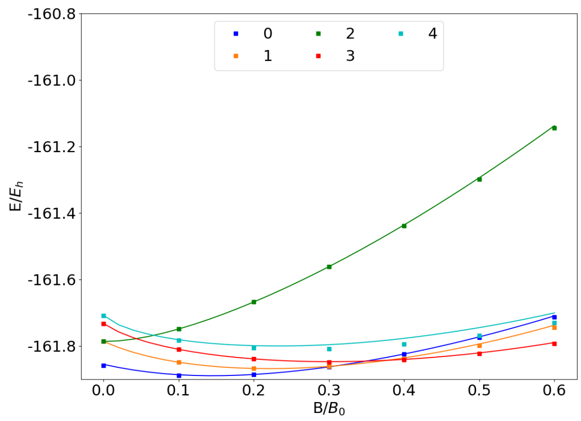

Figure S314: Total energies of all considered states of the Na atom in the HGBSP3-7 basis set in fully uncontracted form (solid lines). The FEM values are shown by the squares of the same color.

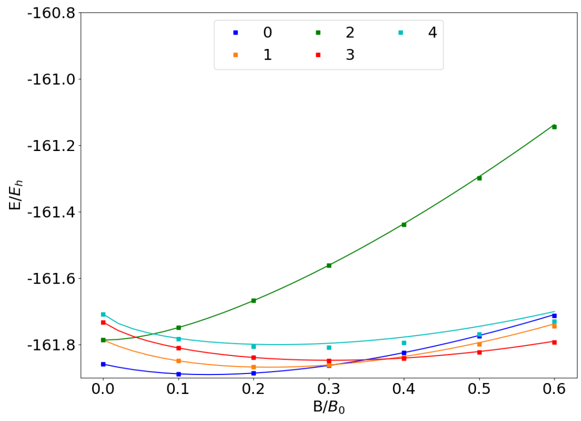

Figure S315: Total energies of all considered states of the Na atom in the HGBSP3-9 basis set in fully uncontracted form (solid lines). The FEM values are shown by the squares of the same color.

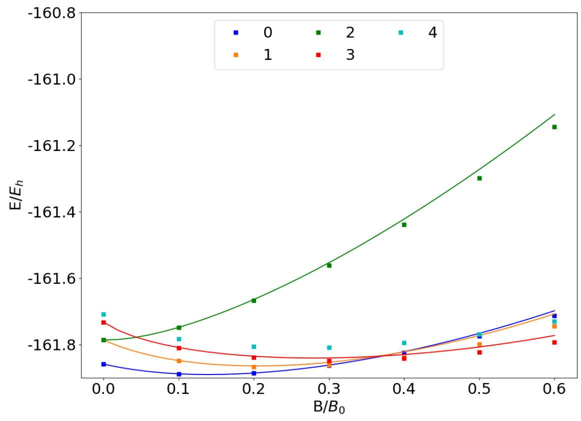

Figure S317: Total energies of all considered states of the Na atom in the AHGBSP1-7 basis set in fully uncontracted form (solid lines). The FEM values are shown by the squares of the same color.

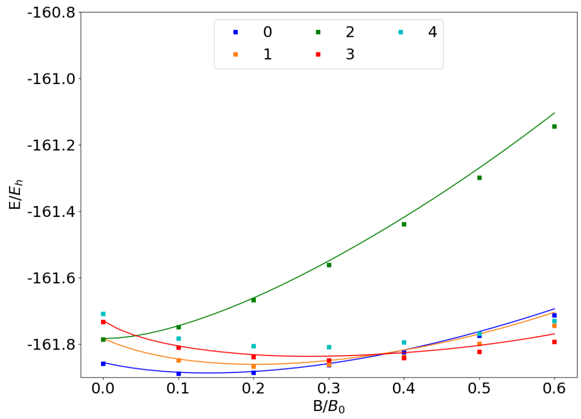

Figure S316: Total energies of all considered states of the Na atom in the AHGBSP1-5 basis set in fully uncontracted form (solid lines). The FEM values are shown by the squares of the same color.

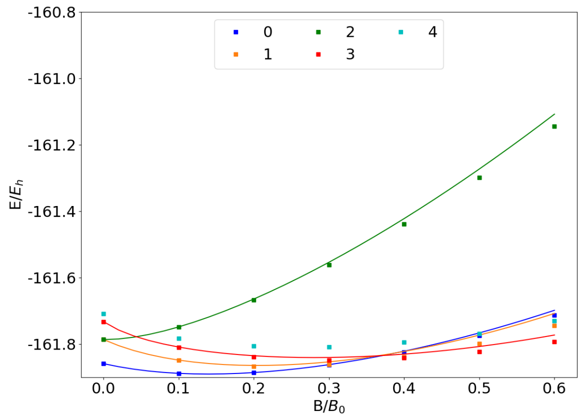

Figure S318: Total energies of all considered states of the Na atom in the AHGBSP1-9 basis set in fully uncontracted form (solid lines). The FEM values are shown by the squares of the same color.

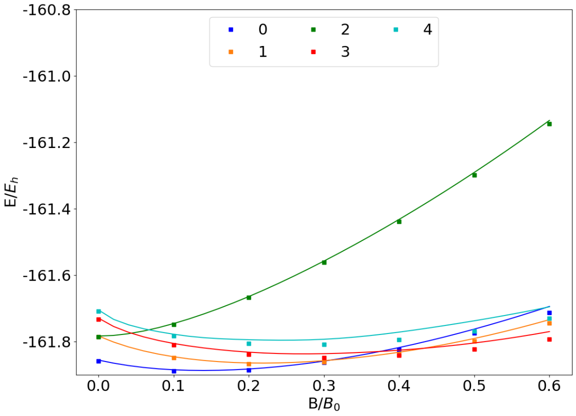

Figure S319: Total energies of all considered states of the Na atom in the AHGBSP2-5 basis set in fully uncontracted form (solid lines). The FEM values are shown by the squares of the same color.

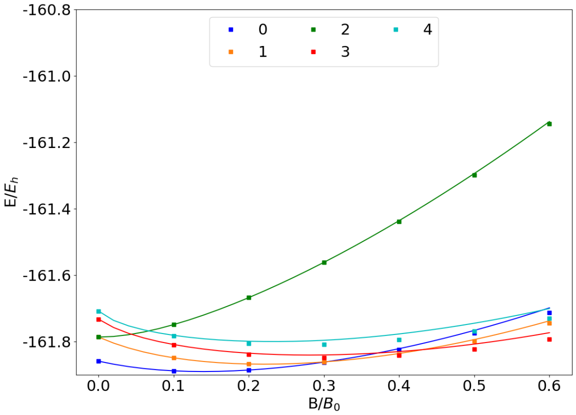

Figure S320: Total energies of all considered states of the Na atom in the AHGBSP2-7 basis set in fully uncontracted form (solid lines). The FEM values are shown by the squares of the same color.

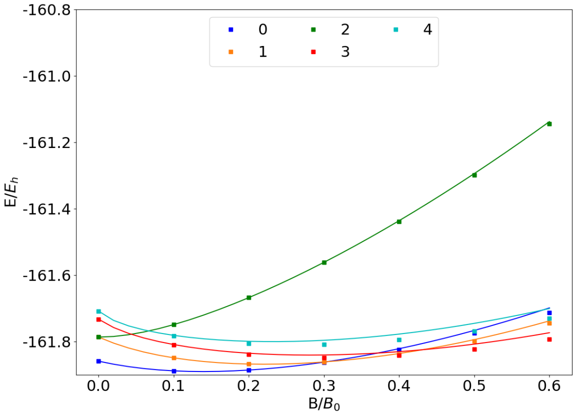

Figure S321: Total energies of all considered states of the Na atom in the AHGBSP2-9 basis set in fully uncontracted form (solid lines). The FEM values are shown by the squares of the same color.

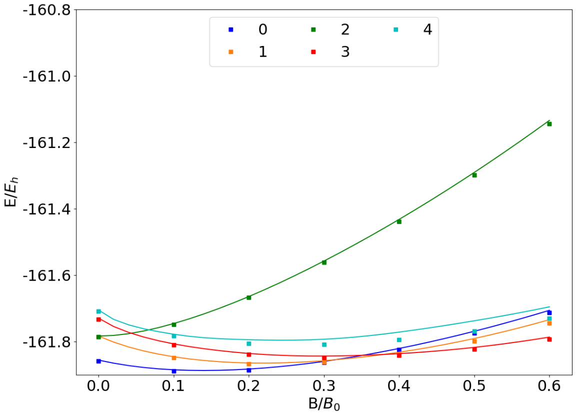

Figure S322: Total energies of all considered states of the Na atom in the AHGBSP3-5 basis set in fully uncontracted form (solid lines). The FEM values are shown by the squares of the same color.

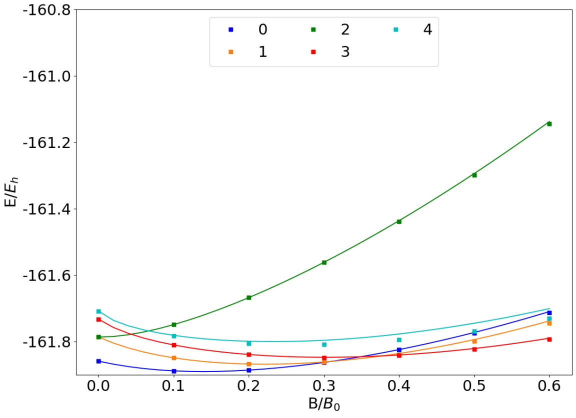

Figure S323: Total energies of all considered states of the Na atom in the AHGBSP3-7 basis set in fully uncontracted form (solid lines). The FEM values are shown by the squares of the same color.

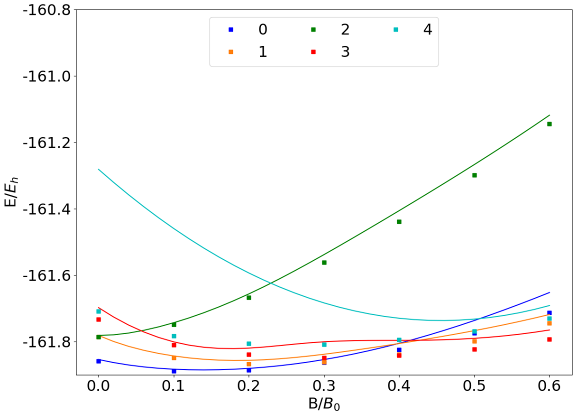

Figure S325: Total energies of all considered states of the Na atom in the 6-311++G(3df,3pd) basis set in fully uncontracted form (solid lines). The FEM values are shown by the squares of the same color.

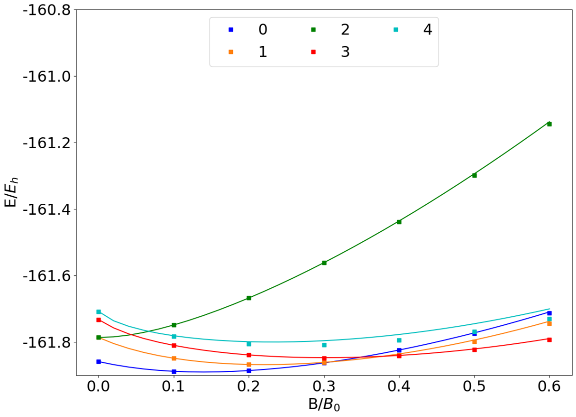

Figure S324: Total energies of all considered states of the Na atom in the AHGBSP3-9 basis set in fully uncontracted form (solid lines). The FEM values are shown by the squares of the same color.

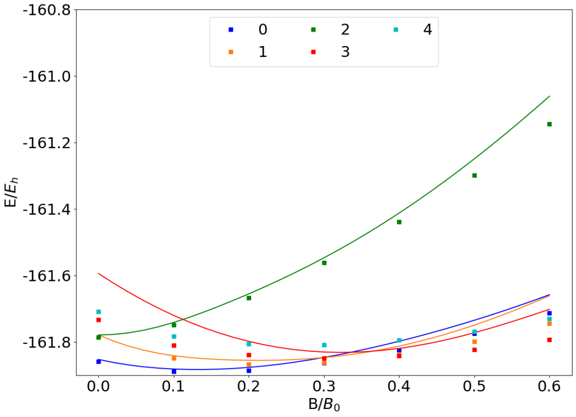

Figure S326: Total energies of all considered states of the Na atom in the def2-TZVP basis set in fully uncontracted form (solid lines). The FEM values are shown by the squares of the same color.

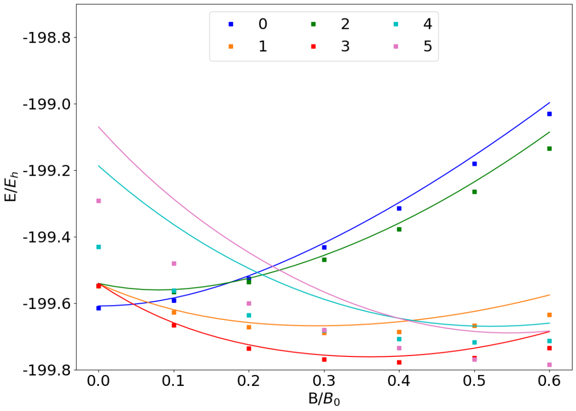

Figure S327: Total energies of all considered states of the Mg atom in the cc-pVDZ basis set in fully uncontracted form (solid lines). The FEM values are shown by the squares of the same color.

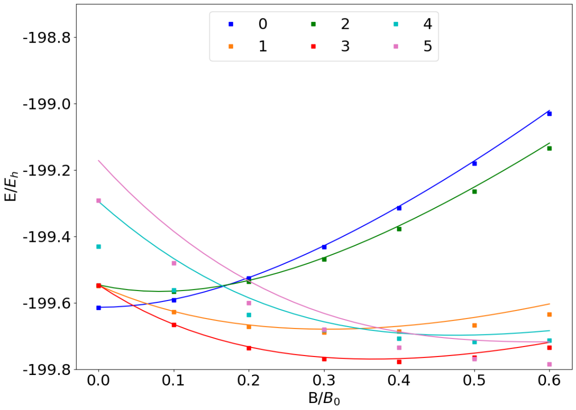

Figure S328: Total energies of all considered states of the Mg atom in the cc-pVTZ basis set in fully uncontracted form (solid lines). The FEM values are shown by the squares of the same color.

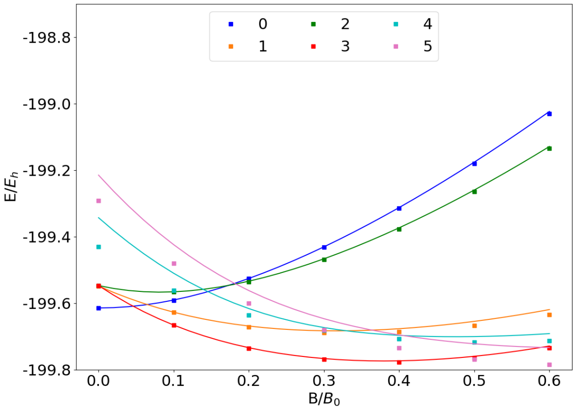

Figure S329: Total energies of all considered states of the Mg atom in the cc-pVQZ basis set in fully uncontracted form (solid lines). The FEM values are shown by the squares of the same color.

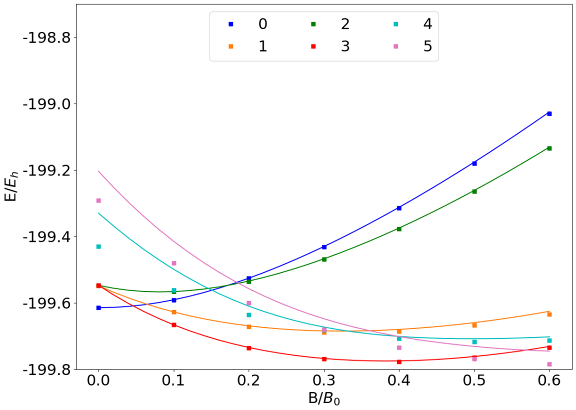

Figure S330: Total energies of all considered states of the Mg atom in the cc-pV5Z basis set in fully uncontracted form (solid lines). The FEM values are shown by the squares of the same color.

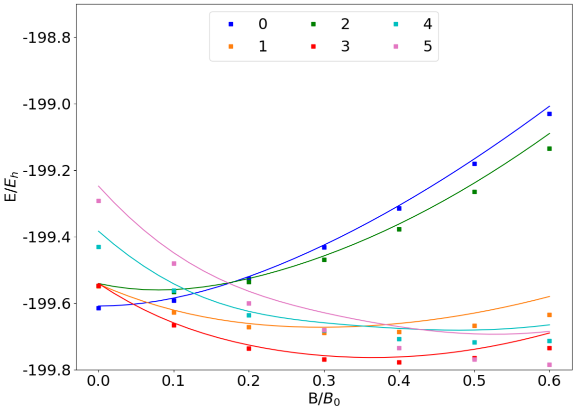

Figure S331: Total energies of all considered states of the Mg atom in the aug-cc-pVDZ basis set in fully uncontracted form (solid lines). The FEM values are shown by the squares of the same color.

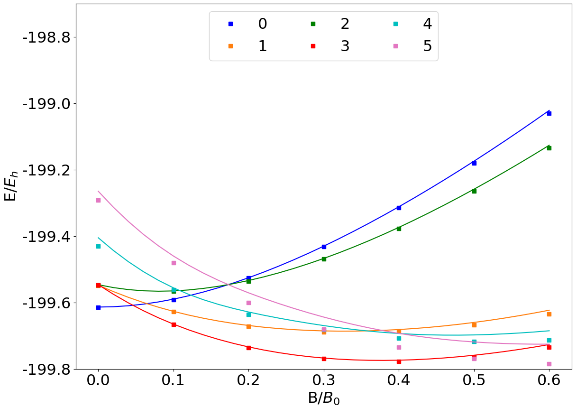

Figure S332: Total energies of all considered states of the Mg atom in the aug-cc-pVTZ basis set in fully uncontracted form (solid lines). The FEM values are shown by the squares of the same color.

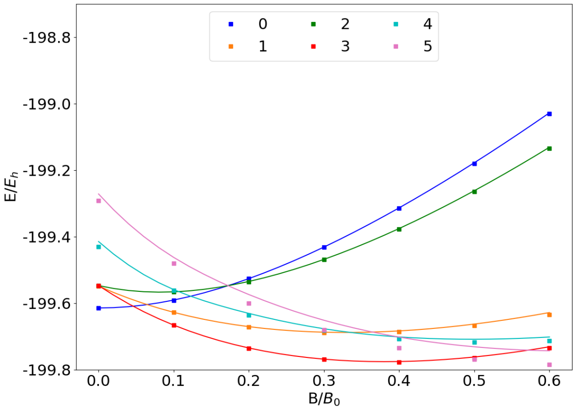

Figure S333: Total energies of all considered states of the Mg atom in the aug-cc-pVQZ basis set in fully uncontracted form (solid lines). The FEM values are shown by the squares of the same color.

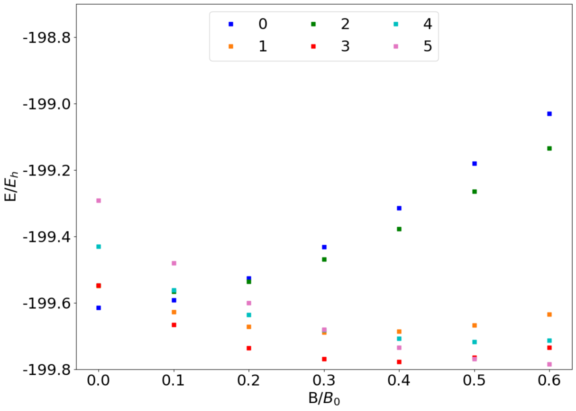

Figure S334: Total energies of all considered states of the Mg atom in the aug-cc-pV5Z basis set in fully uncontracted form (solid lines). The FEM values are shown by the squares of the same color.

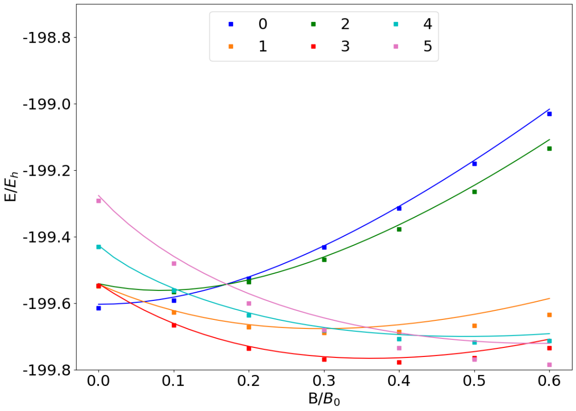

Figure S335: Total energies of all considered states of the Mg atom in the HGBSP1-5 basis set in fully uncontracted form (solid lines). The FEM values are shown by the squares of the same color.

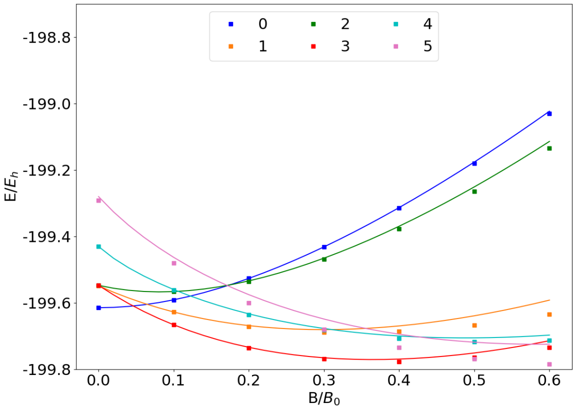

Figure S336: Total energies of all considered states of the Mg atom in the HGBSP1-7 basis set in fully uncontracted form (solid lines). The FEM values are shown by the squares of the same color.

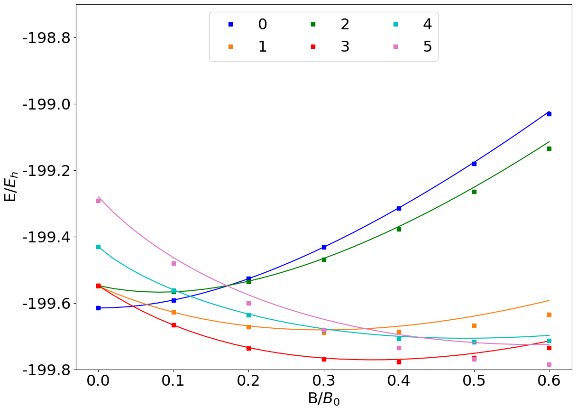

Figure S337: Total energies of all considered states of the Mg atom in the HGBSP1-9 basis set in fully uncontracted form (solid lines). The FEM values are shown by the squares of the same color.

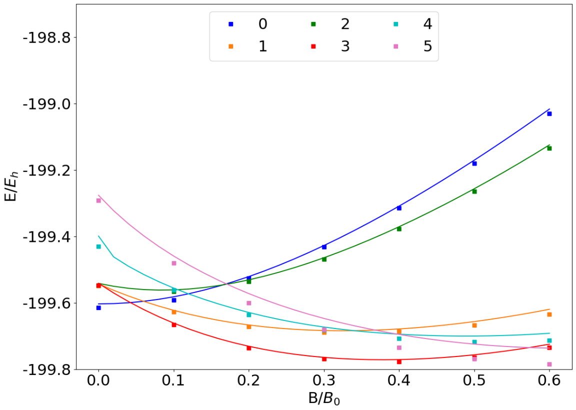

Figure S338: Total energies of all considered states of the Mg atom in the HGBSP2-5 basis set in fully uncontracted form (solid lines). The FEM values are shown by the squares of the same color.

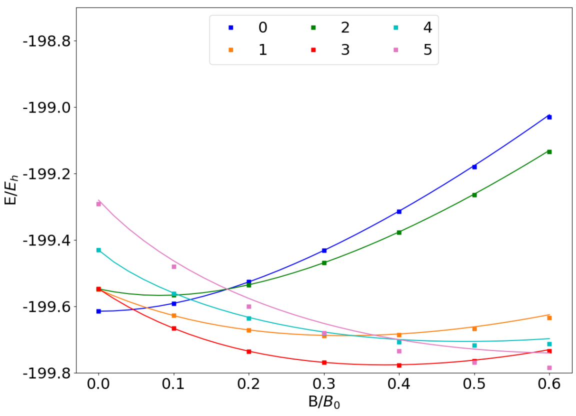

Figure S339: Total energies of all considered states of the Mg atom in the HGBSP2-7 basis set in fully uncontracted form (solid lines). The FEM values are shown by the squares of the same color.

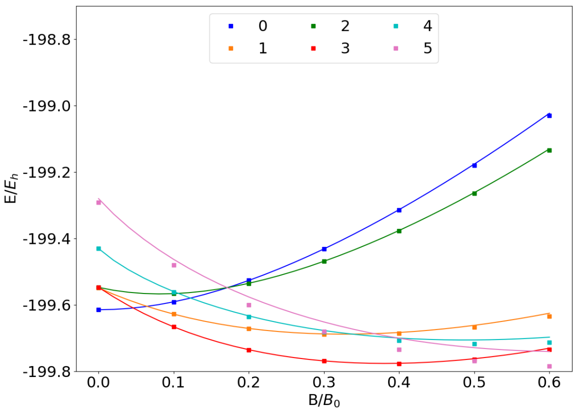

Figure S340: Total energies of all considered states of the Mg atom in the HGBSP2-9 basis set in fully uncontracted form (solid lines). The FEM values are shown by the squares of the same color.

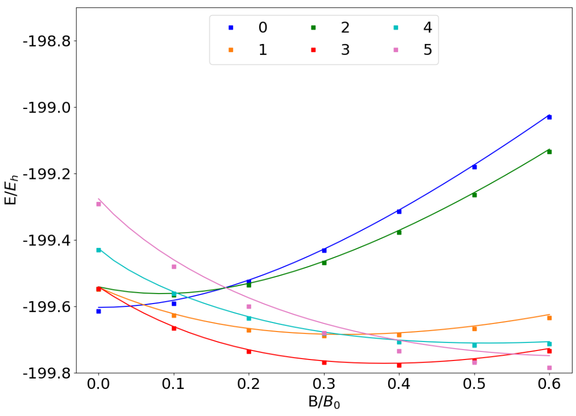

Figure S341: Total energies of all considered states of the Mg atom in the HGBSP3-5 basis set in fully uncontracted form (solid lines). The FEM values are shown by the squares of the same color.

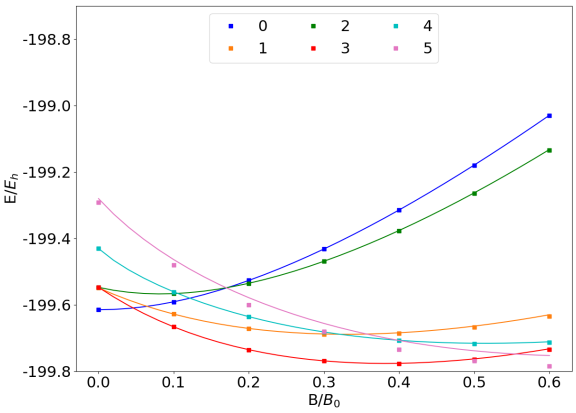

Figure S342: Total energies of all considered states of the Mg atom in the HGBSP3-7 basis set in fully uncontracted form (solid lines). The FEM values are shown by the squares of the same color.

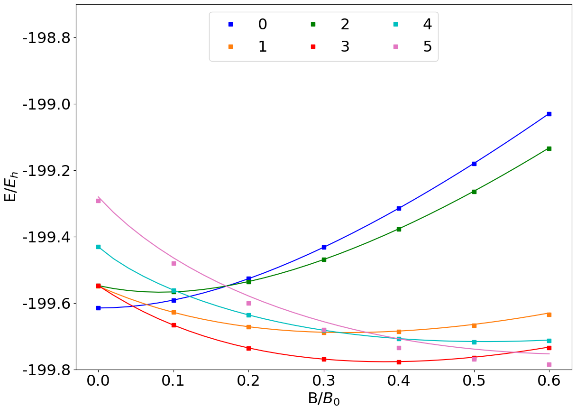

Figure S343: Total energies of all considered states of the Mg atom in the HGBSP3-9 basis set in fully uncontracted form (solid lines). The FEM values are shown by the squares of the same color.

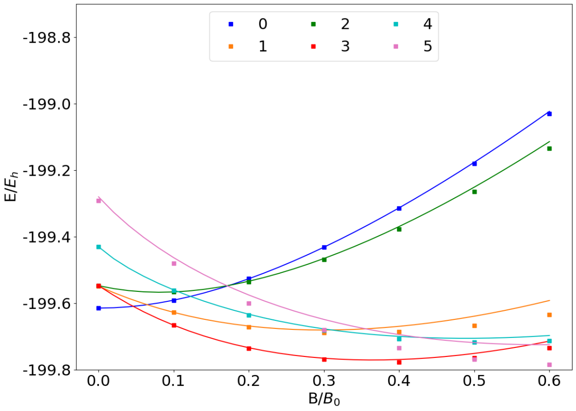

Figure S345: Total energies of all considered states of the Mg atom in the AHGBSP1-7 basis set in fully uncontracted form (solid lines). The FEM values are shown by the squares of the same color.

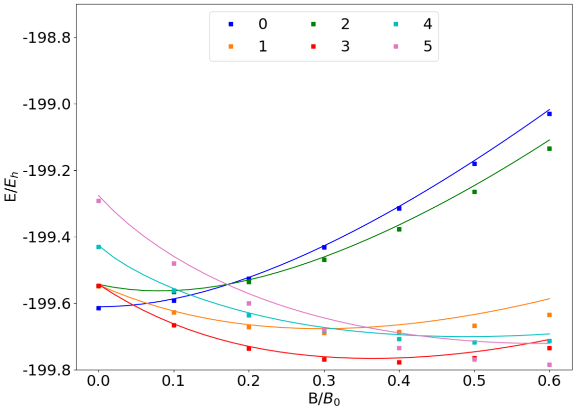

Figure S344: Total energies of all considered states of the Mg atom in the AHGBSP1-5 basis set in fully uncontracted form (solid lines). The FEM values are shown by the squares of the same color.

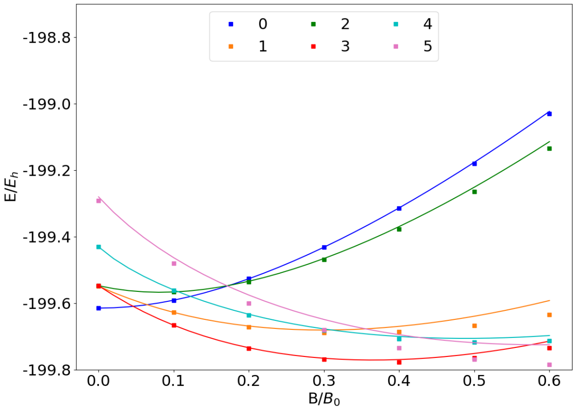

Figure S346: Total energies of all considered states of the Mg atom in the AHGBSP1-9 basis set in fully uncontracted form (solid lines). The FEM values are shown by the squares of the same color.

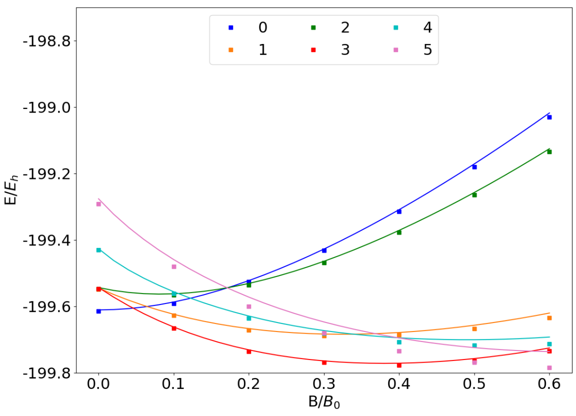

Figure S347: Total energies of all considered states of the Mg atom in the AHGBSP2-5 basis set in fully uncontracted form (solid lines). The FEM values are shown by the squares of the same color.

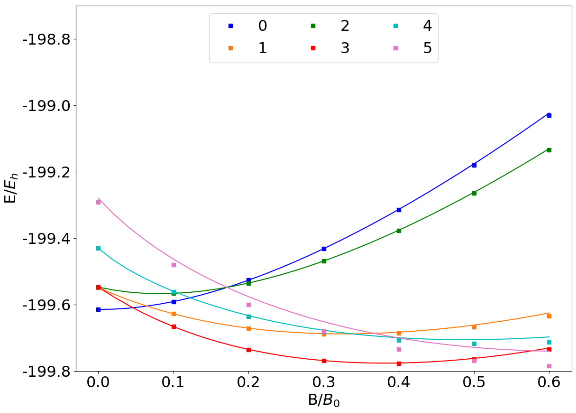

Figure S348: Total energies of all considered states of the Mg atom in the AHGBSP2-7 basis set in fully uncontracted form (solid lines). The FEM values are shown by the squares of the same color.

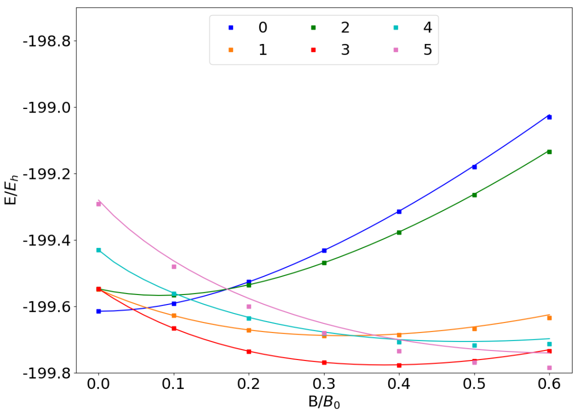

Figure S349: Total energies of all considered states of the Mg atom in the AHGBSP2-9 basis set in fully uncontracted form (solid lines). The FEM values are shown by the squares of the same color.

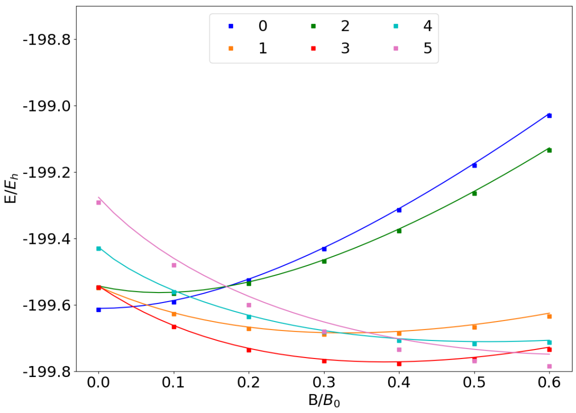

Figure S350: Total energies of all considered states of the Mg atom in the AHGBSP3-5 basis set in fully uncontracted form (solid lines). The FEM values are shown by the squares of the same color.

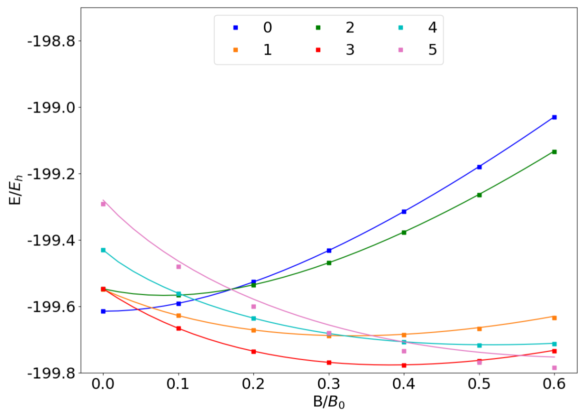

Figure S351: Total energies of all considered states of the Mg atom in the AHGBSP3-7 basis set in fully uncontracted form (solid lines). The FEM values are shown by the squares of the same color.

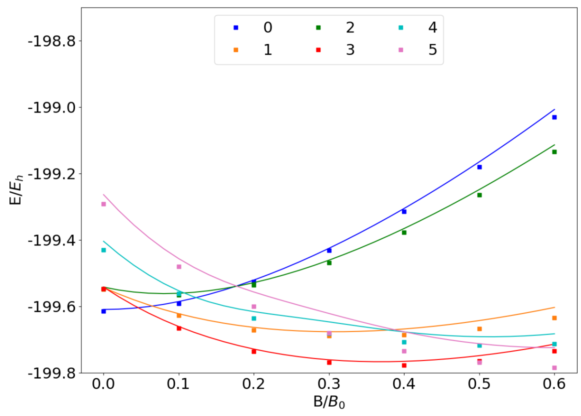

Figure S353: Total energies of all considered states of the Mg atom in the 6-311++G(3df,3pd) basis set in fully uncontracted form (solid lines). The FEM values are shown by the squares of the same color.

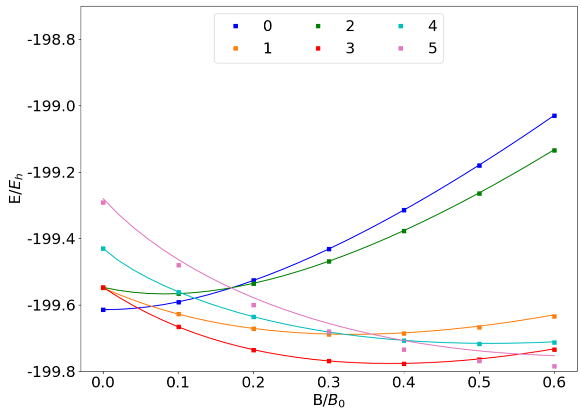

Figure S352: Total energies of all considered states of the Mg atom in the AHGBSP3-9 basis set in fully uncontracted form (solid lines). The FEM values are shown by the squares of the same color.

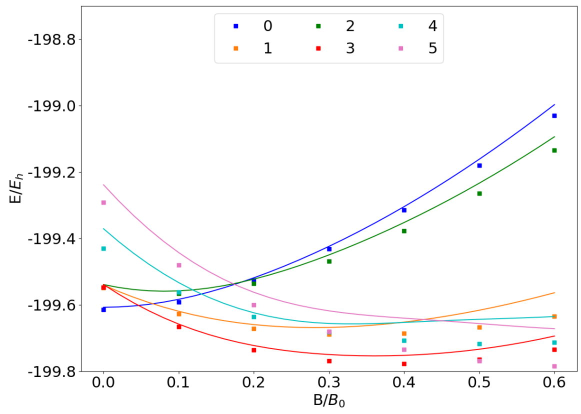

Figure S354: Total energies of all considered states of the Mg atom in the def2-TZVP basis set in fully uncontracted form (solid lines). The FEM values are shown by the squares of the same color.

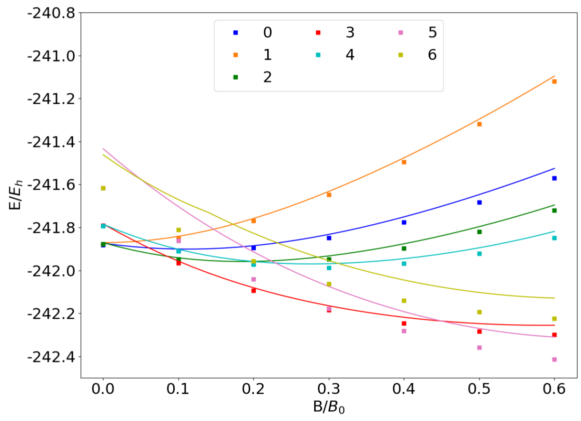

Figure S355: Total energies of all considered states of the Al atom in the cc-pVDZ basis set in fully uncontracted form (solid lines). The FEM values are shown by the squares of the same color.

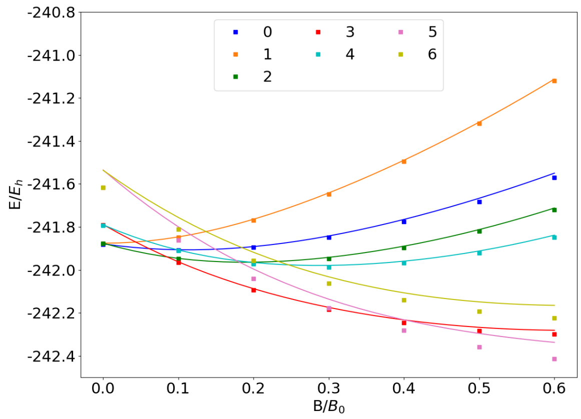

Figure S356: Total energies of all considered states of the Al atom in the cc-pVTZ basis set in fully uncontracted form (solid lines). The FEM values are shown by the squares of the same color.

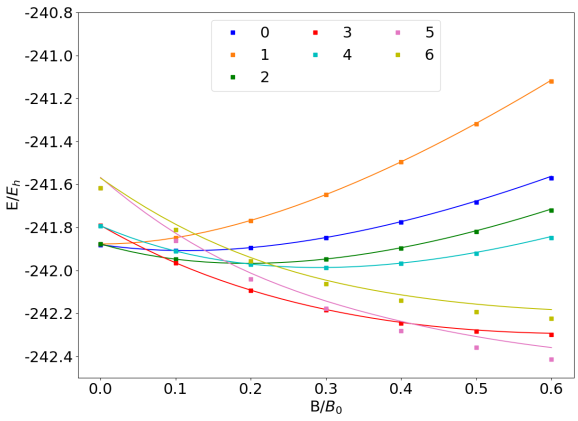

Figure S357: Total energies of all considered states of the Al atom in the cc-pVQZ basis set in fully uncontracted form (solid lines). The FEM values are shown by the squares of the same color.

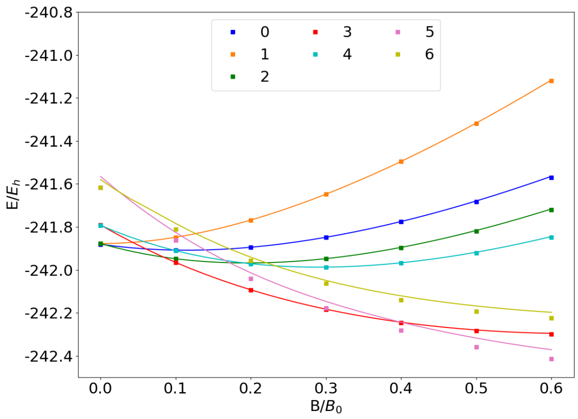

Figure S358: Total energies of all considered states of the Al atom in the cc-pV5Z basis set in fully uncontracted form (solid lines). The FEM values are shown by the squares of the same color.

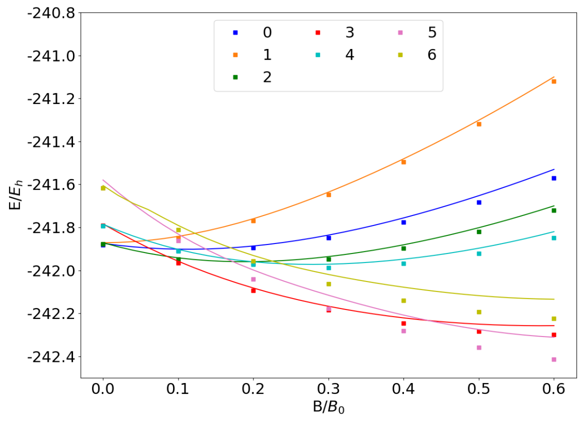

Figure S359: Total energies of all considered states of the Al atom in the aug-cc-pVDZ basis set in fully uncontracted form (solid lines). The FEM values are shown by the squares of the same color.

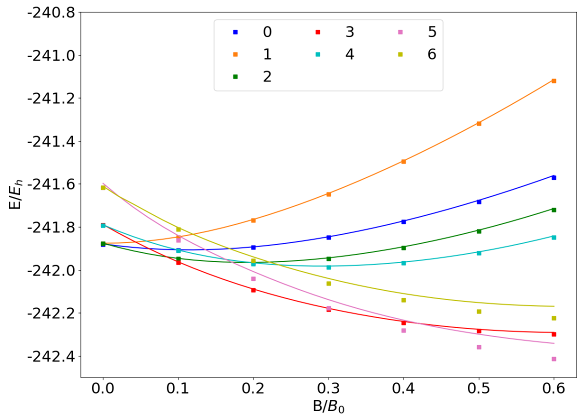

Figure S360: Total energies of all considered states of the Al atom in the aug-cc-pVTZ basis set in fully uncontracted form (solid lines). The FEM values are shown by the squares of the same color.

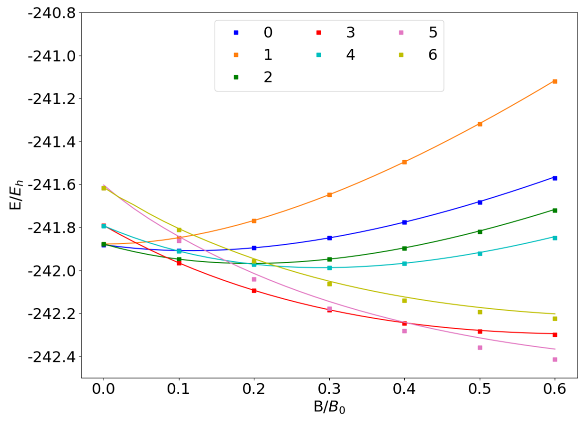

Figure S361: Total energies of all considered states of the Al atom in the aug-cc-pVQZ basis set in fully uncontracted form (solid lines). The FEM values are shown by the squares of the same color.

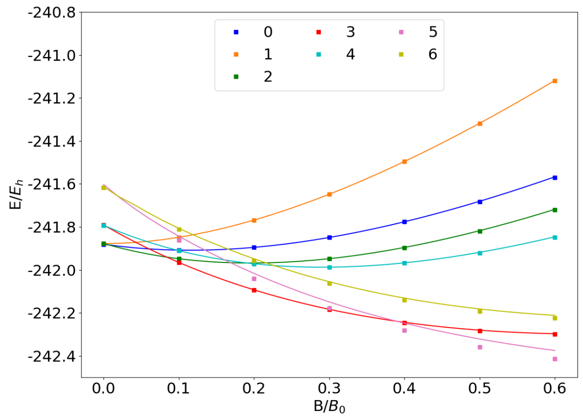

Figure S362: Total energies of all considered states of the Al atom in the aug-cc-pV5Z basis set in fully uncontracted form (solid lines). The FEM values are shown by the squares of the same color.

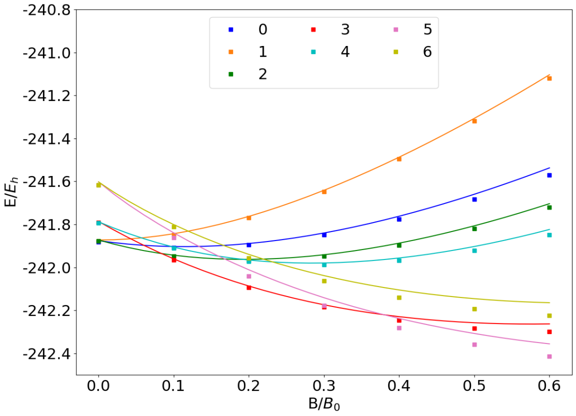

Figure S363: Total energies of all considered states of the Al atom in the HGBSP1-5 basis set in fully uncontracted form (solid lines). The FEM values are shown by the squares of the same color.

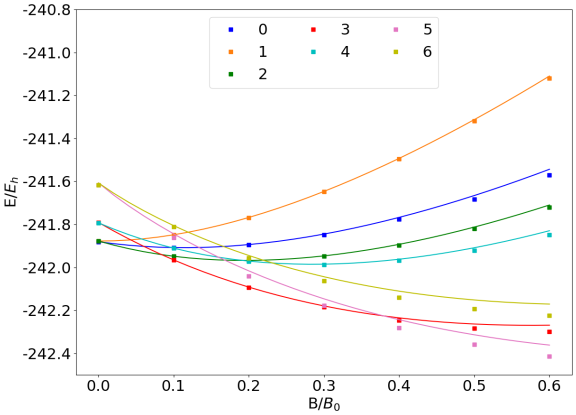

Figure S364: Total energies of all considered states of the Al atom in the HGBSP1-7 basis set in fully uncontracted form (solid lines). The FEM values are shown by the squares of the same color.

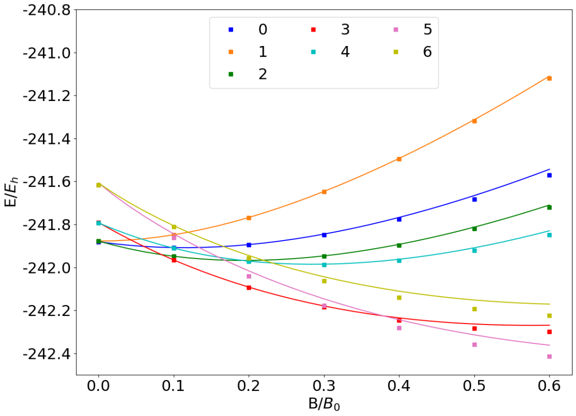

Figure S365: Total energies of all considered states of the Al atom in the HGBSP1-9 basis set in fully uncontracted form (solid lines). The FEM values are shown by the squares of the same color.

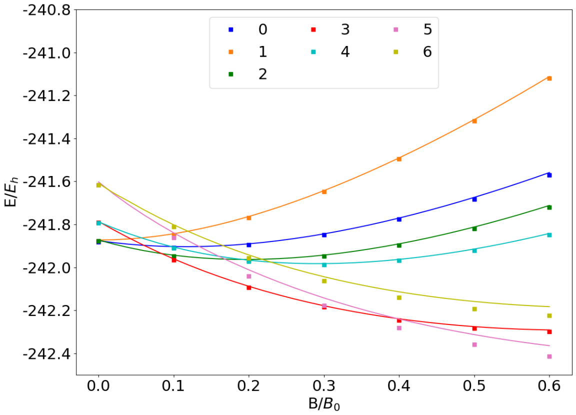

Figure S366: Total energies of all considered states of the Al atom in the HGBSP2-5 basis set in fully uncontracted form (solid lines). The FEM values are shown by the squares of the same color.

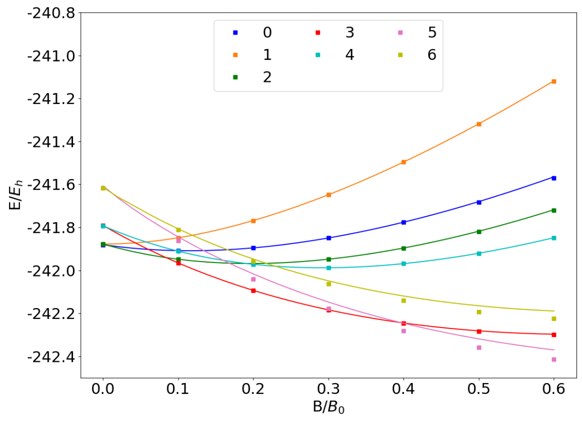

Figure S367: Total energies of all considered states of the Al atom in the HGBSP2-7 basis set in fully uncontracted form (solid lines). The FEM values are shown by the squares of the same color.

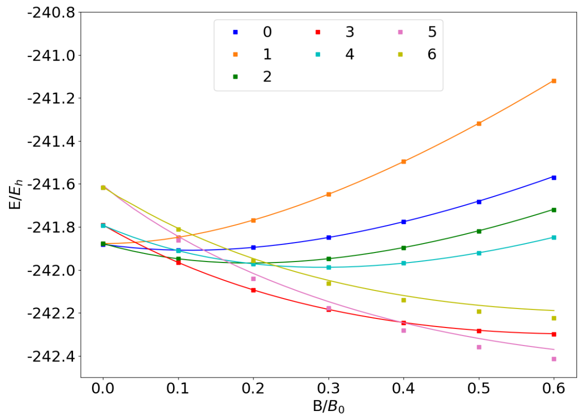

Figure S368: Total energies of all considered states of the Al atom in the HGBSP2-9 basis set in fully uncontracted form (solid lines). The FEM values are shown by the squares of the same color.

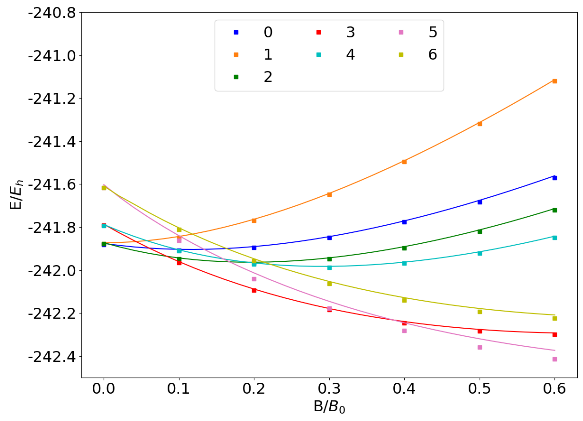

Figure S369: Total energies of all considered states of the Al atom in the HGBSP3-5 basis set in fully uncontracted form (solid lines). The FEM values are shown by the squares of the same color.

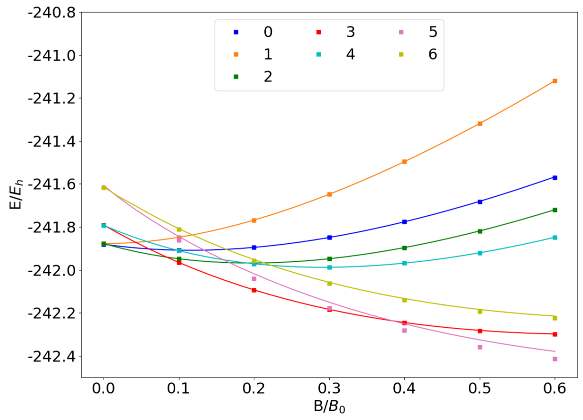

Figure S370: Total energies of all considered states of the Al atom in the HGBSP3-7 basis set in fully uncontracted form (solid lines). The FEM values are shown by the squares of the same color.

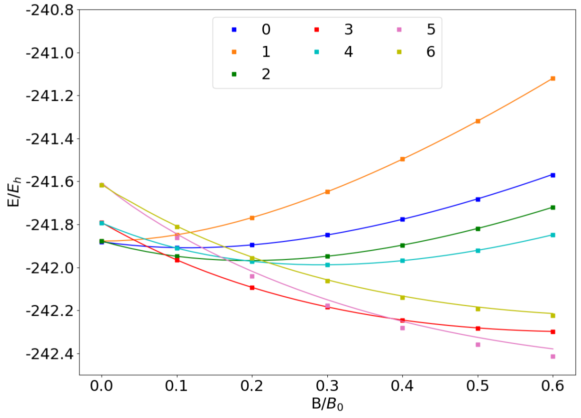

Figure S371: Total energies of all considered states of the Al atom in the HGBSP3-9 basis set in fully uncontracted form (solid lines). The FEM values are shown by the squares of the same color.

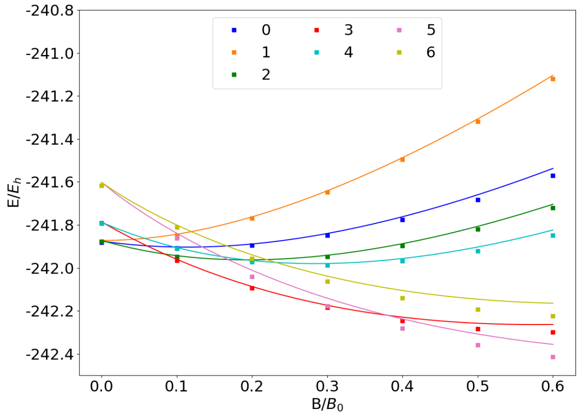

Figure S372: Total energies of all considered states of the Al atom in the AHGBSP1-5 basis set in fully uncontracted form (solid lines). The FEM values are shown by the squares of the same color.

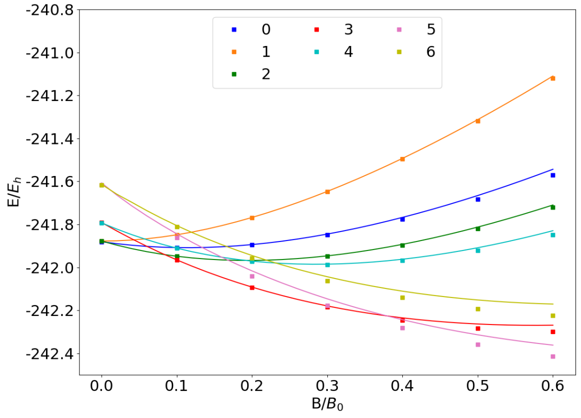

Figure S373: Total energies of all considered states of the Al atom in the AHGBSP1-7 basis set in fully uncontracted form (solid lines). The FEM values are shown by the squares of the same color.

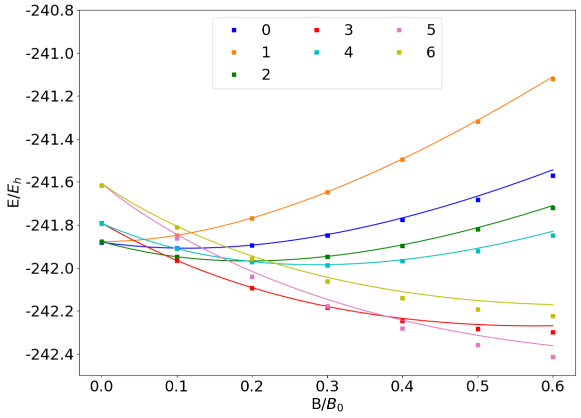

Figure S374: Total energies of all considered states of the Al atom in the AHGBSP1-9 basis set in fully uncontracted form (solid lines). The FEM values are shown by the squares of the same color.

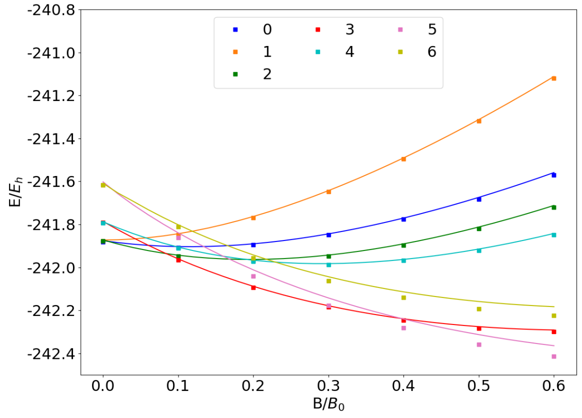

Figure S375: Total energies of all considered states of the Al atom in the AHGBSP2-5 basis set in fully uncontracted form (solid lines). The FEM values are shown by the squares of the same color.

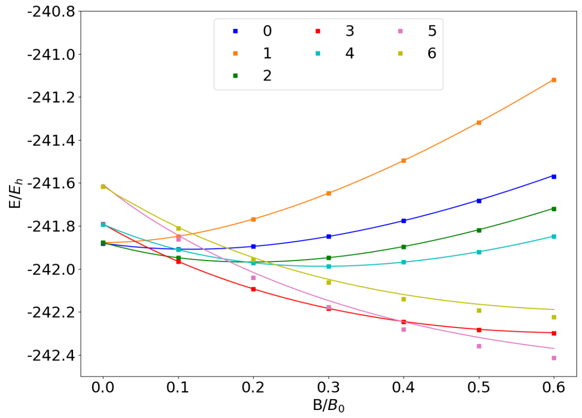

Figure S376: Total energies of all considered states of the Al atom in the AHGBSP2-7 basis set in fully uncontracted form (solid lines). The FEM values are shown by the squares of the same color.

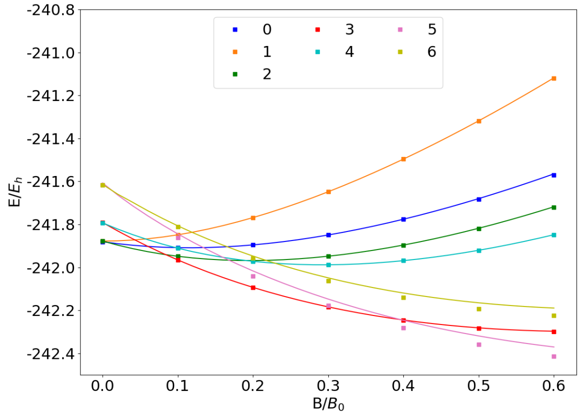

Figure S377: Total energies of all considered states of the Al atom in the AHGBSP2-9 basis set in fully uncontracted form (solid lines). The FEM values are shown by the squares of the same color.

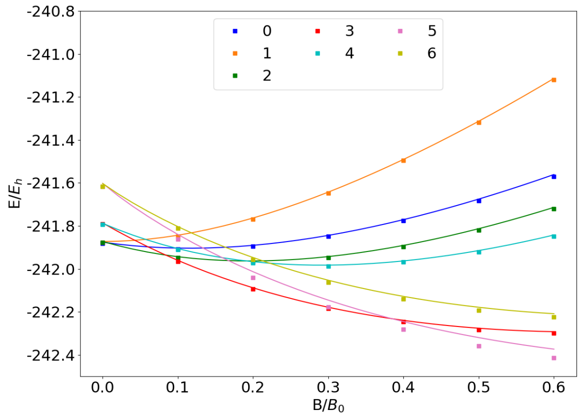

Figure S378: Total energies of all considered states of the Al atom in the AHGBSP3-5 basis set in fully uncontracted form (solid lines). The FEM values are shown by the squares of the same color.

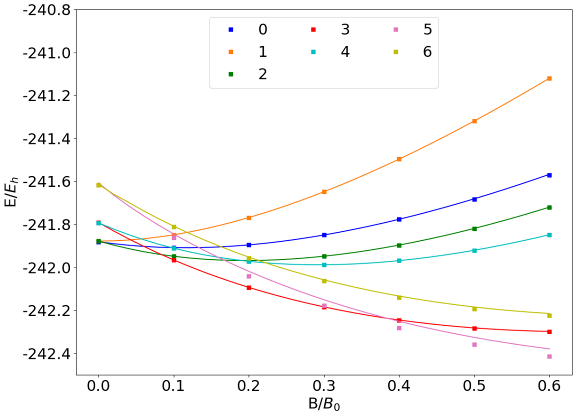

Figure S379: Total energies of all considered states of the Al atom in the AHGBSP3-7 basis set in fully uncontracted form (solid lines). The FEM values are shown by the squares of the same color.

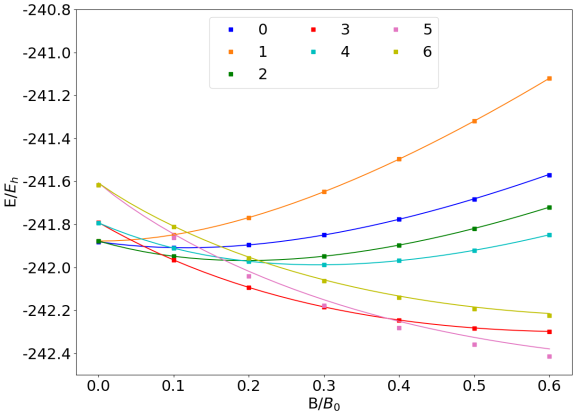

Figure S380: Total energies of all considered states of the Al atom in the AHGBSP3-9 basis set in fully uncontracted form (solid lines). The FEM values are shown by the squares of the same color.

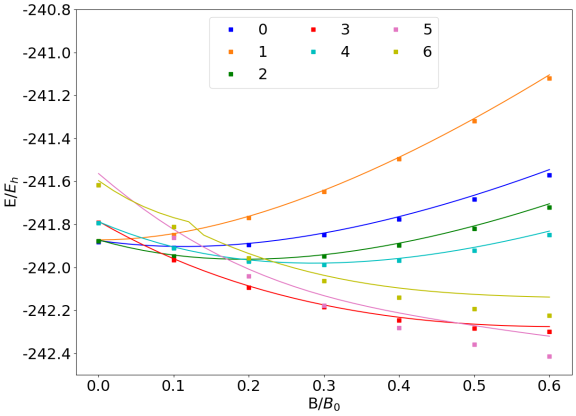

Figure S381: Total energies of all considered states of the Al atom in the 6-311++G(3df,3pd) basis set in fully uncontracted form (solid lines). The FEM values are shown by the squares of the same color.

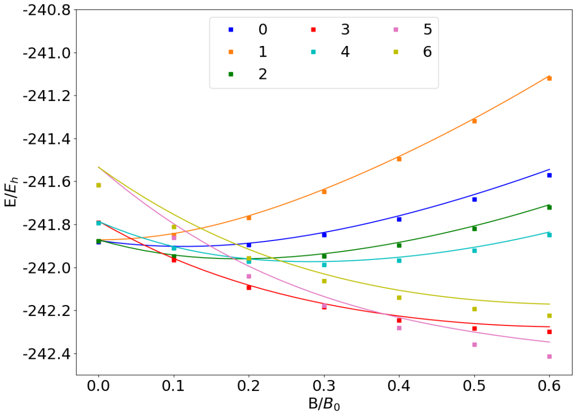

Figure S382: Total energies of all considered states of the Al atom in the def2-TZVP basis set in fully uncontracted form (solid lines). The FEM values are shown by the squares of the same color.

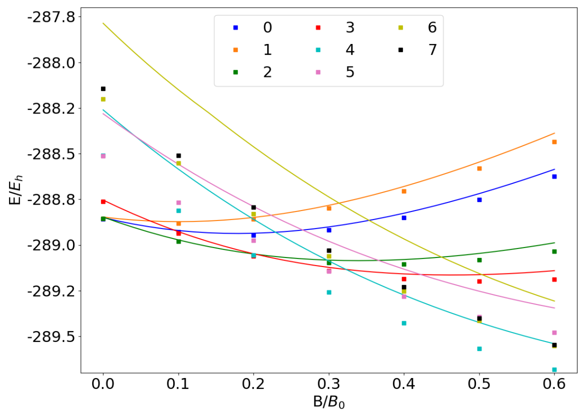

Figure S383: Total energies of all considered states of the Si atom in the cc-pVDZ basis set in fully uncontracted form (solid lines). The FEM values are shown by the squares of the same color.

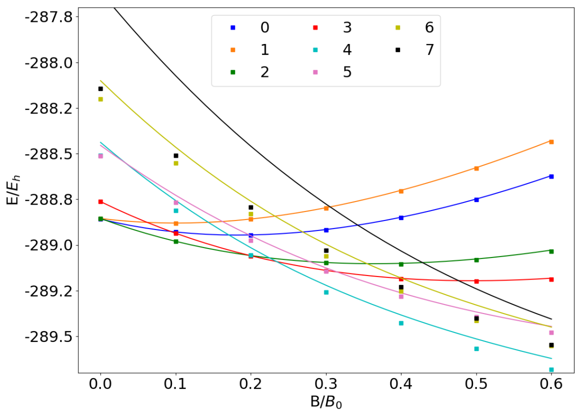

Figure S385: Total energies of all considered states of the Si atom in the cc-pVQZ basis set in fully uncontracted form (solid lines). The FEM values are shown by the squares of the same color.

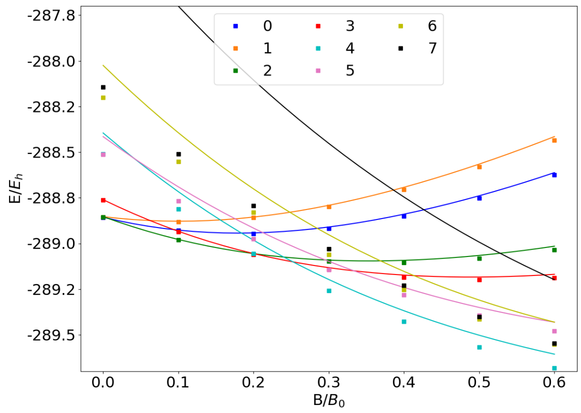

Figure S384: Total energies of all considered states of the Si atom in the cc-pVTZ basis set in fully uncontracted form (solid lines). The FEM values are shown by the squares of the same color.

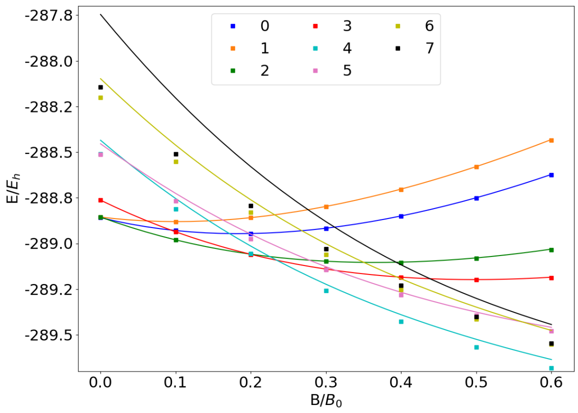

Figure S386: Total energies of all considered states of the Si atom in the cc-pV5Z basis set in fully uncontracted form (solid lines). The FEM values are shown by the squares of the same color.

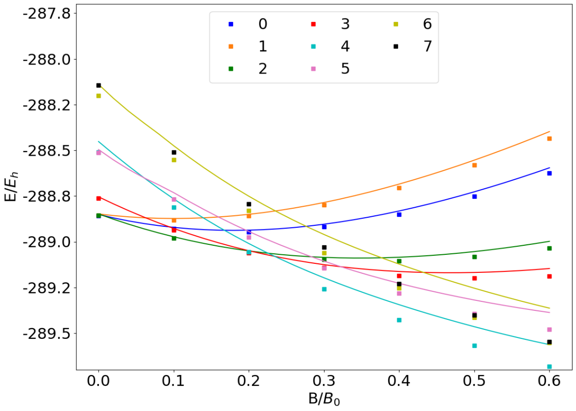

Figure S387: Total energies of all considered states of the Si atom in the aug-cc-pVDZ basis set in fully uncontracted form (solid lines). The FEM values are shown by the squares of the same color.

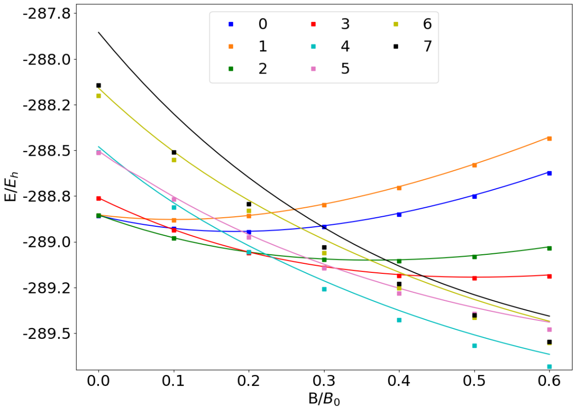

Figure S388: Total energies of all considered states of the Si atom in the aug-cc-pVTZ basis set in fully uncontracted form (solid lines). The FEM values are shown by the squares of the same color.

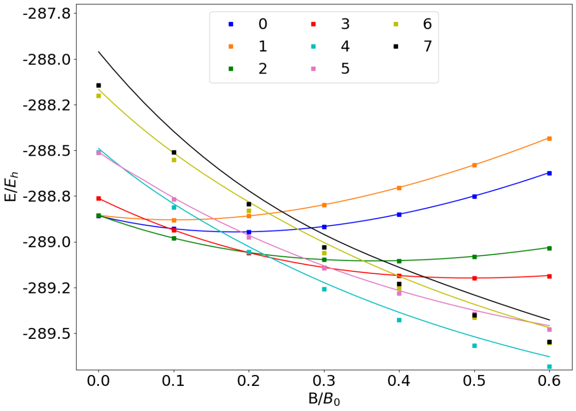

Figure S389: Total energies of all considered states of the Si atom in the aug-cc-pVQZ basis set in fully uncontracted form (solid lines). The FEM values are shown by the squares of the same color.

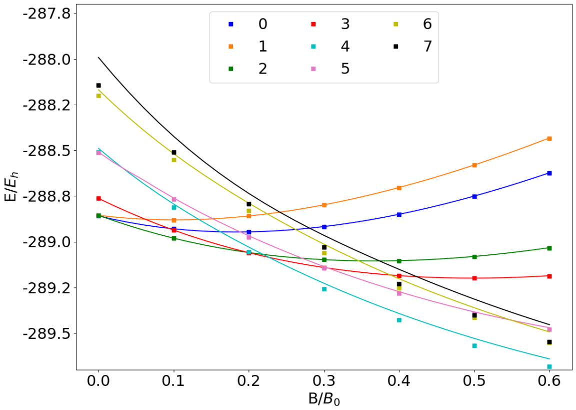

Figure S390: Total energies of all considered states of the Si atom in the aug-cc-pV5Z basis set in fully uncontracted form (solid lines). The FEM values are shown by the squares of the same color.

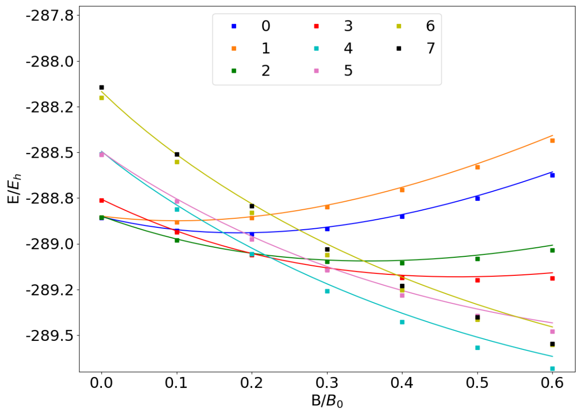

Figure S391: Total energies of all considered states of the Si atom in the HGBSP1-5 basis set in fully uncontracted form (solid lines). The FEM values are shown by the squares of the same color.

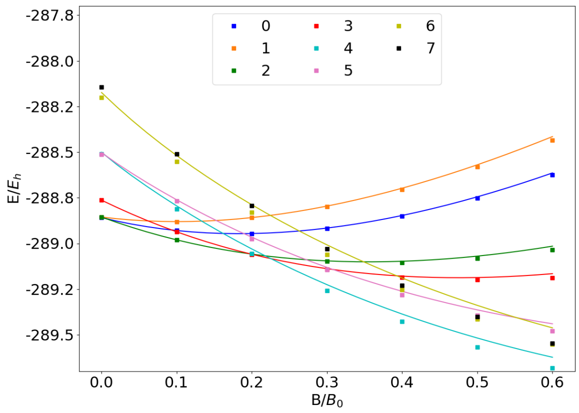

Figure S392: Total energies of all considered states of the Si atom in the HGBSP1-7 basis set in fully uncontracted form (solid lines). The FEM values are shown by the squares of the same color.

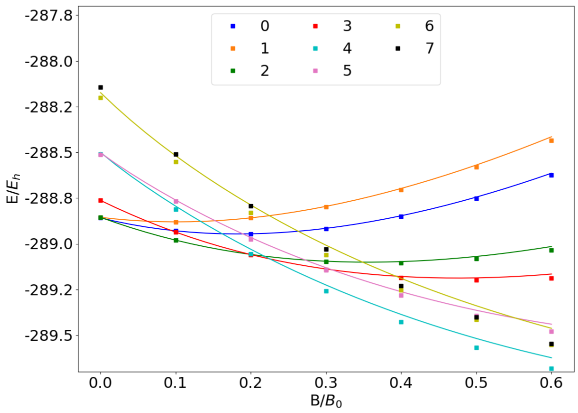

Figure S393: Total energies of all considered states of the Si atom in the HGBSP1-9 basis set in fully uncontracted form (solid lines). The FEM values are shown by the squares of the same color.

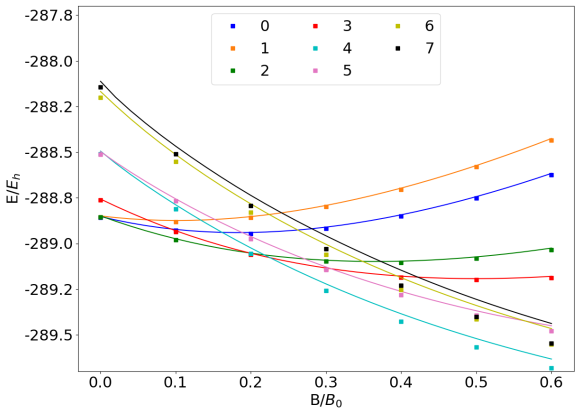

Figure S394: Total energies of all considered states of the Si atom in the HGBSP2-5 basis set in fully uncontracted form (solid lines). The FEM values are shown by the squares of the same color.

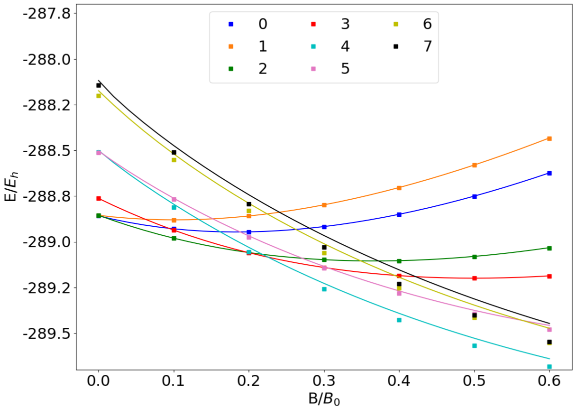

Figure S395: Total energies of all considered states of the Si atom in the HGBSP2-7 basis set in fully uncontracted form (solid lines). The FEM values are shown by the squares of the same color.

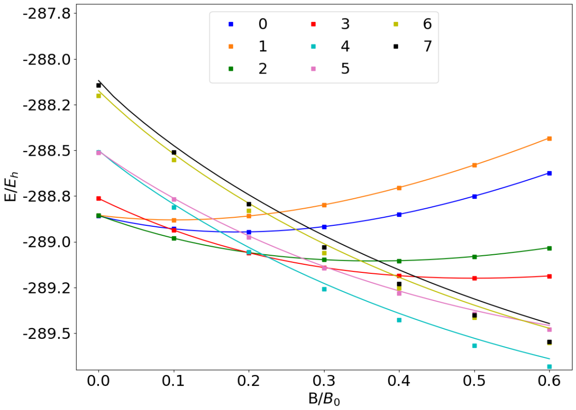

Figure S396: Total energies of all considered states of the Si atom in the HGBSP2-9 basis set in fully uncontracted form (solid lines). The FEM values are shown by the squares of the same color.

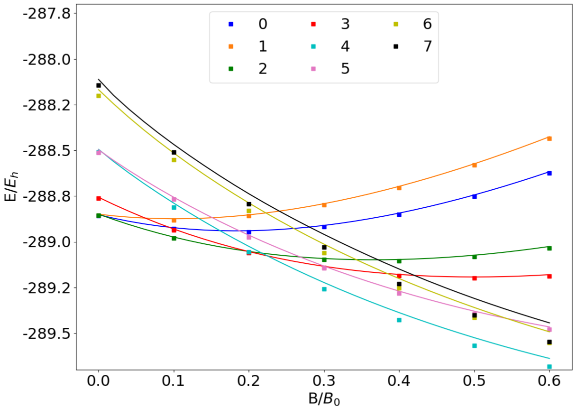

Figure S397: Total energies of all considered states of the Si atom in the HGBSP3-5 basis set in fully uncontracted form (solid lines). The FEM values are shown by the squares of the same color.

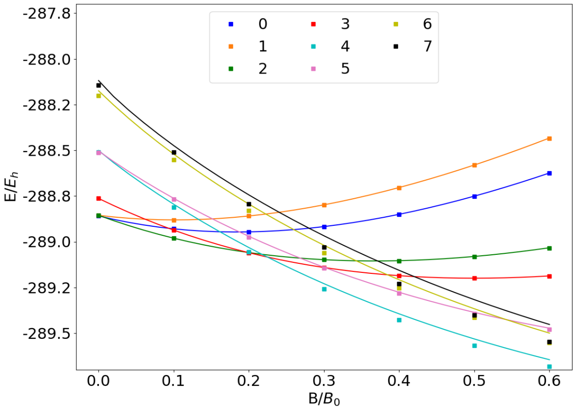

Figure S398: Total energies of all considered states of the Si atom in the HGBSP3-7 basis set in fully uncontracted form (solid lines). The FEM values are shown by the squares of the same color.

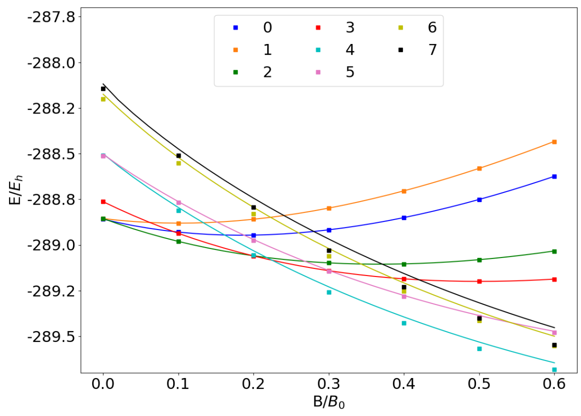

Figure S399: Total energies of all considered states of the Si atom in the HGBSP3-9 basis set in fully uncontracted form (solid lines). The FEM values are shown by the squares of the same color.

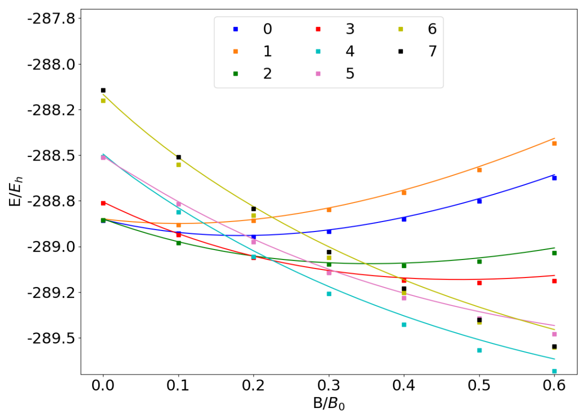

Figure S400: Total energies of all considered states of the Si atom in the AHGBSP1-5 basis set in fully uncontracted form (solid lines). The FEM values are shown by the squares of the same color.

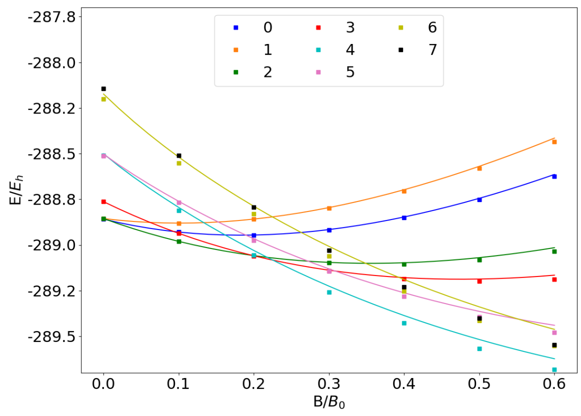

Figure S401: Total energies of all considered states of the Si atom in the AHGBSP1-7 basis set in fully uncontracted form (solid lines). The FEM values are shown by the squares of the same color.

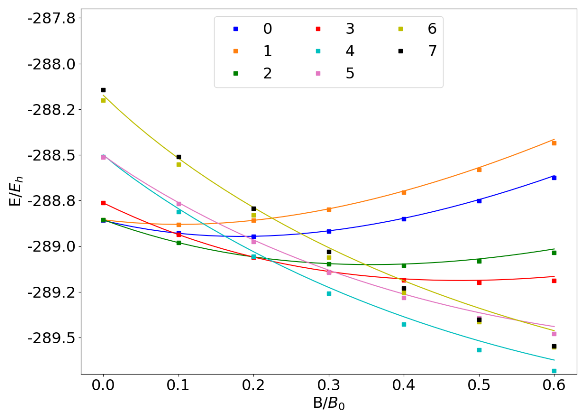

Figure S402: Total energies of all considered states of the Si atom in the AHGBSP1-9 basis set in fully uncontracted form (solid lines). The FEM values are shown by the squares of the same color.

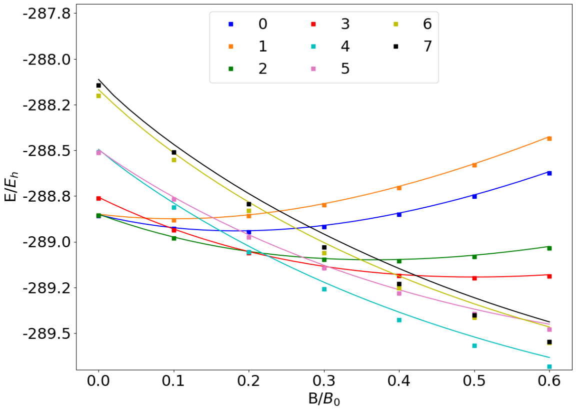

Figure S403: Total energies of all considered states of the Si atom in the AHGBSP2-5 basis set in fully uncontracted form (solid lines). The FEM values are shown by the squares of the same color.

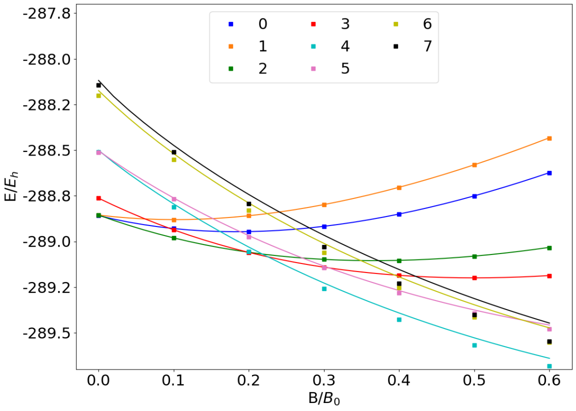

Figure S404: Total energies of all considered states of the Si atom in the AHGBSP2-7 basis set in fully uncontracted form (solid lines). The FEM values are shown by the squares of the same color.

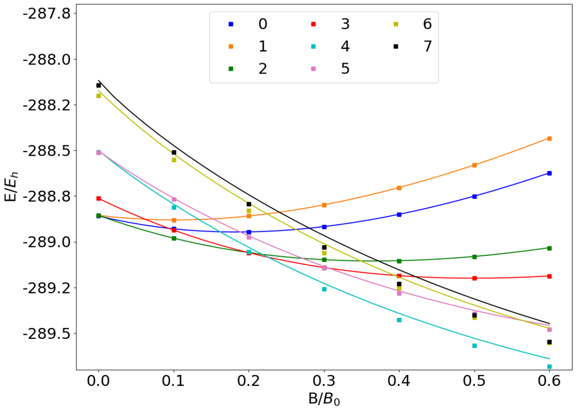

Figure S405: Total energies of all considered states of the Si atom in the AHGBSP2-9 basis set in fully uncontracted form (solid lines). The FEM values are shown by the squares of the same color.

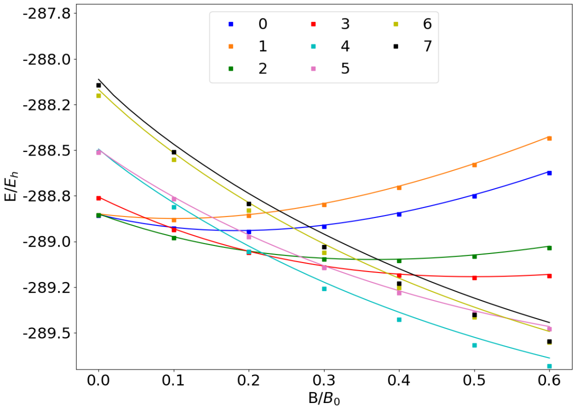

Figure S406: Total energies of all considered states of the Si atom in the AHGBSP3-5 basis set in fully uncontracted form (solid lines). The FEM values are shown by the squares of the same color.

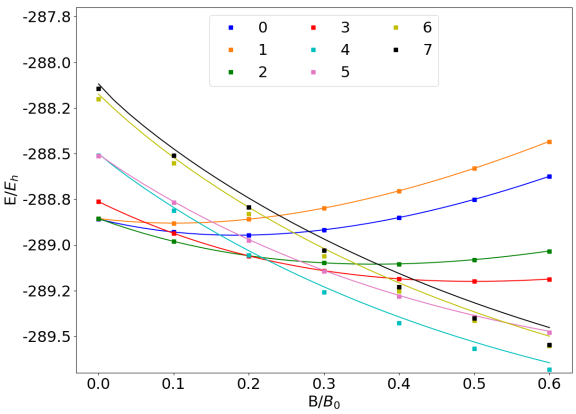

Figure S407: Total energies of all considered states of the Si atom in the AHGBSP3-7 basis set in fully uncontracted form (solid lines). The FEM values are shown by the squares of the same color.

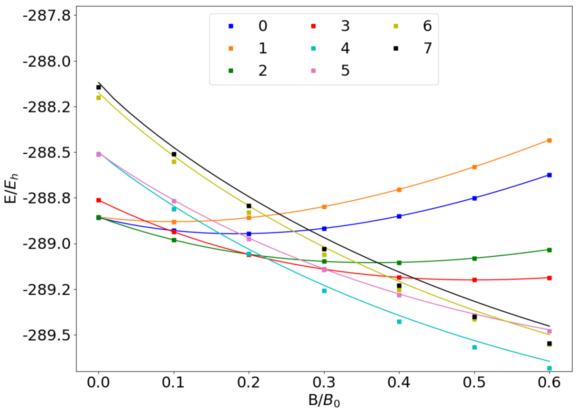

Figure S408: Total energies of all considered states of the Si atom in the AHGBSP3-9 basis set in fully uncontracted form (solid lines). The FEM values are shown by the squares of the same color.

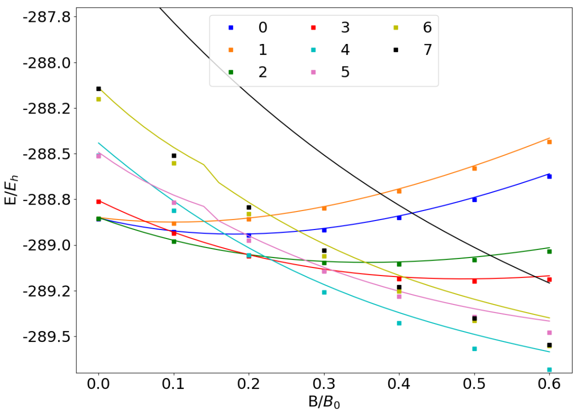

Figure S409: Total energies of all considered states of the Si atom in the 6-311++G(3df,3pd) basis set in fully uncontracted form (solid lines). The FEM values are shown by the squares of the same color.

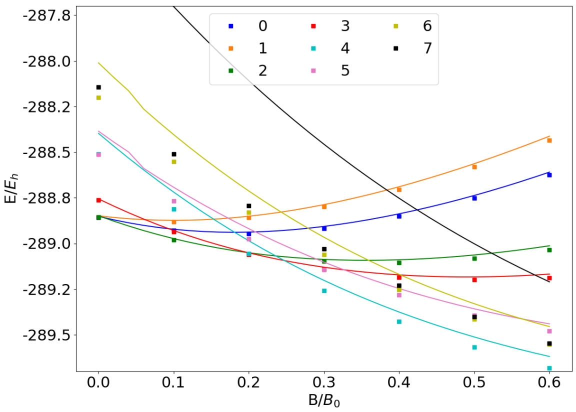

Figure S410: Total energies of all considered states of the Si atom in the def2-TZVP basis set in fully uncontracted form (solid lines). The FEM values are shown by the squares of the same color.

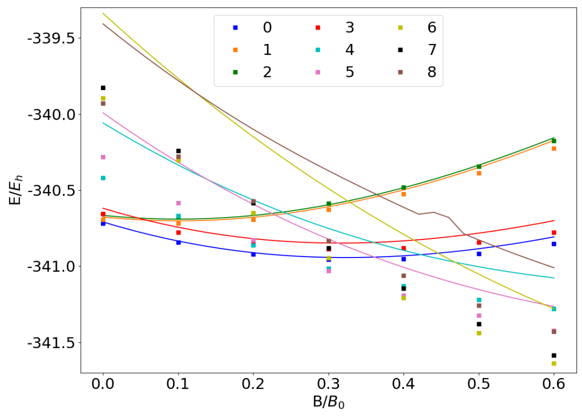

Figure S411: Total energies of all considered states of the P atom in the cc-pVDZ basis set in fully uncontracted form (solid lines). The FEM values are shown by the squares of the same color.

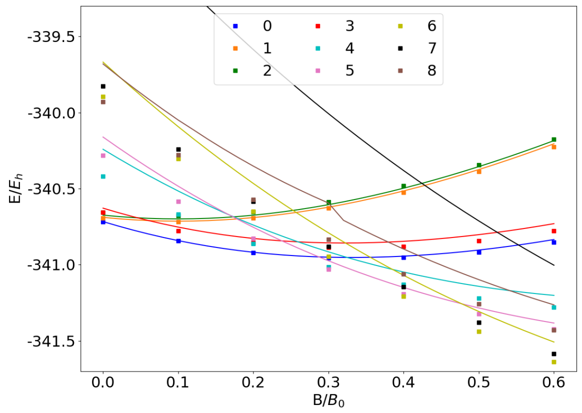

Figure S412: Total energies of all considered states of the P atom in the cc-pVTZ basis set in fully uncontracted form (solid lines). The FEM values are shown by the squares of the same color.

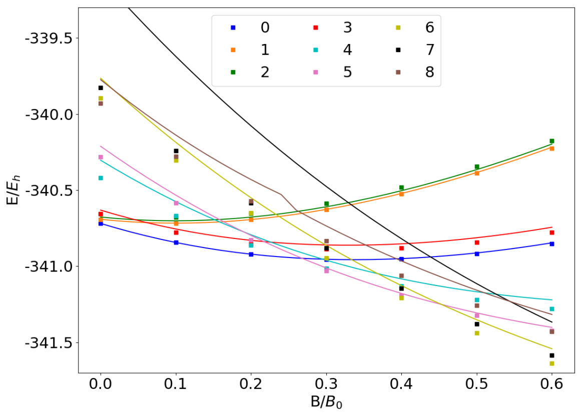

Figure S413: Total energies of all considered states of the P atom in the cc-pVQZ basis set in fully uncontracted form (solid lines). The FEM values are shown by the squares of the same color.

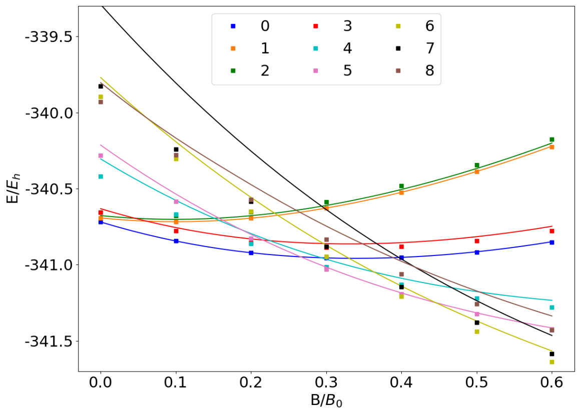

Figure S414: Total energies of all considered states of the P atom in the cc-pV5Z basis set in fully uncontracted form (solid lines). The FEM values are shown by the squares of the same color.

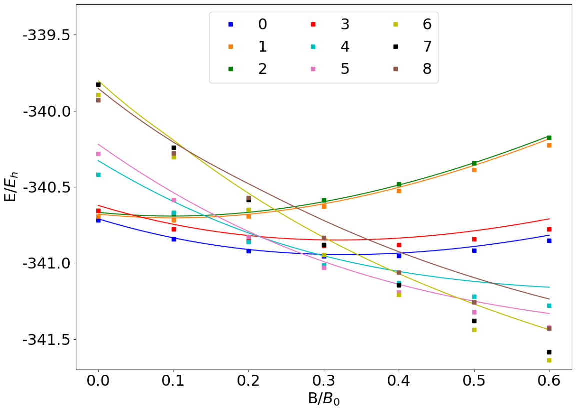

Figure S415: Total energies of all considered states of the P atom in the aug-cc-pVDZ basis set in fully uncontracted form (solid lines). The FEM values are shown by the squares of the same color.

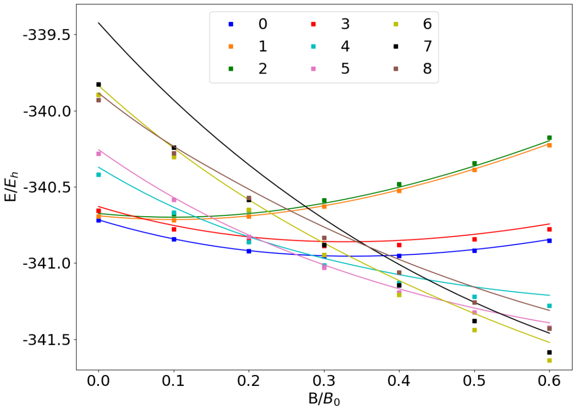

Figure S416: Total energies of all considered states of the P atom in the aug-cc-pVTZ basis set in fully uncontracted form (solid lines). The FEM values are shown by the squares of the same color.

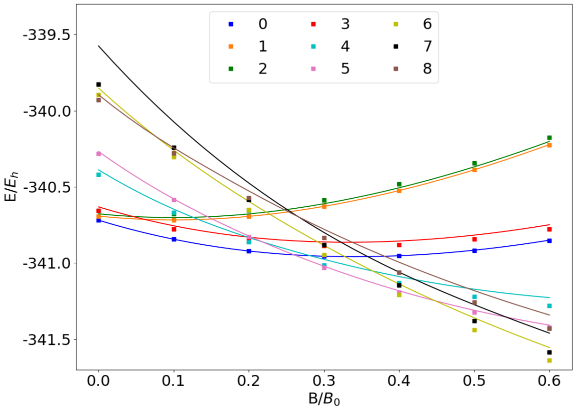

Figure S417: Total energies of all considered states of the P atom in the aug-cc-pVQZ basis set in fully uncontracted form (solid lines). The FEM values are shown by the squares of the same color.

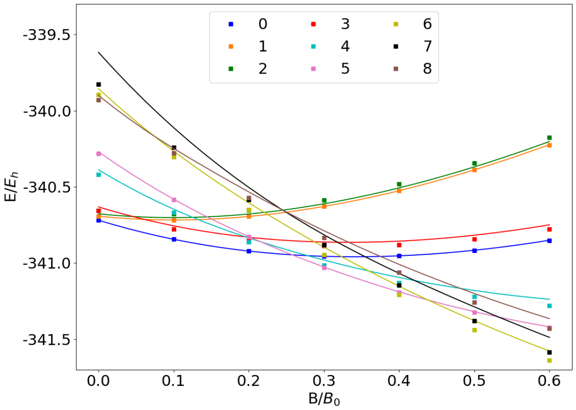

Figure S418: Total energies of all considered states of the P atom in the aug-cc-pV5Z basis set in fully uncontracted form (solid lines). The FEM values are shown by the squares of the same color.

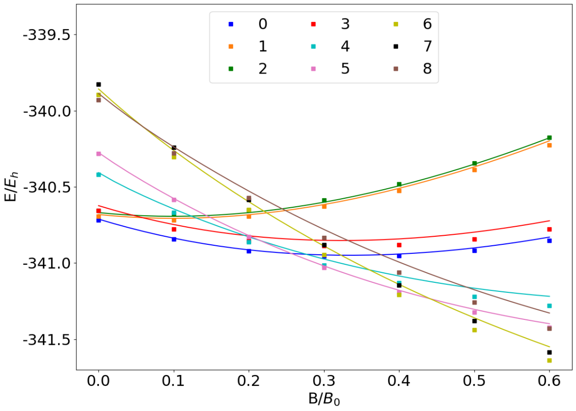

Figure S419: Total energies of all considered states of the P atom in the HGBSP1-5 basis set in fully uncontracted form (solid lines). The FEM values are shown by the squares of the same color.

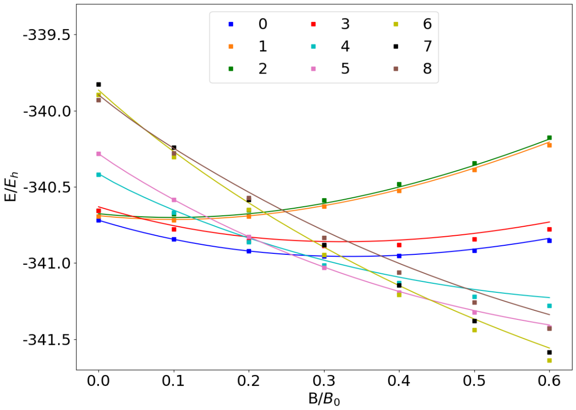

Figure S420: Total energies of all considered states of the P atom in the HGBSP1-7 basis set in fully uncontracted form (solid lines). The FEM values are shown by the squares of the same color.

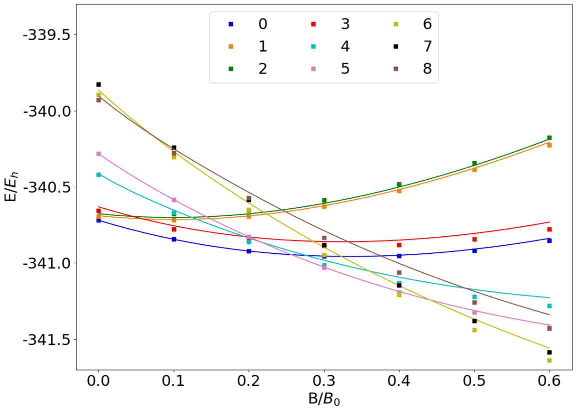

Figure S421: Total energies of all considered states of the P atom in the HGBSP1-9 basis set in fully uncontracted form (solid lines). The FEM values are shown by the squares of the same color.

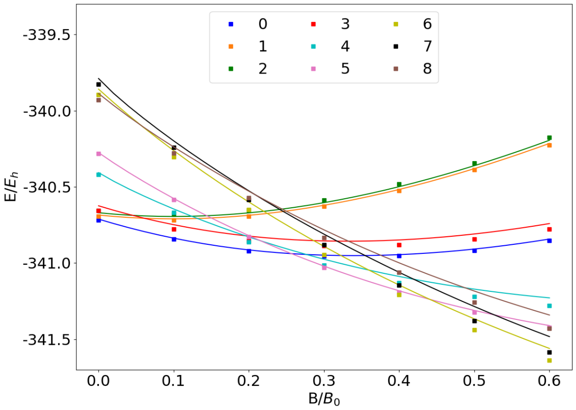

Figure S422: Total energies of all considered states of the P atom in the HGBSP2-5 basis set in fully uncontracted form (solid lines). The FEM values are shown by the squares of the same color.

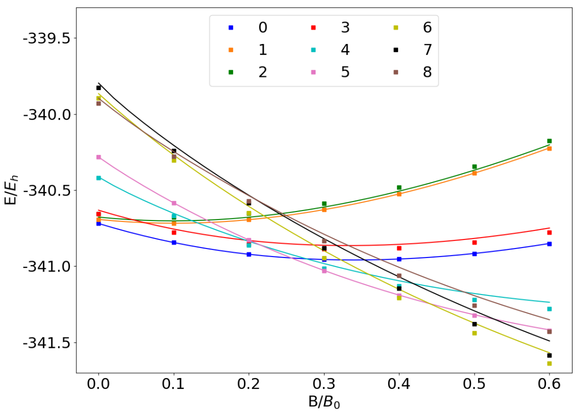

Figure S423: Total energies of all considered states of the P atom in the HGBSP2-7 basis set in fully uncontracted form (solid lines). The FEM values are shown by the squares of the same color.

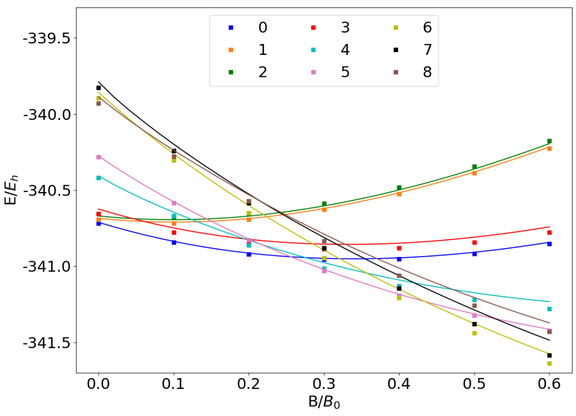

Figure S425: Total energies of all considered states of the P atom in the HGBSP3-5 basis set in fully uncontracted form (solid lines). The FEM values are shown by the squares of the same color.

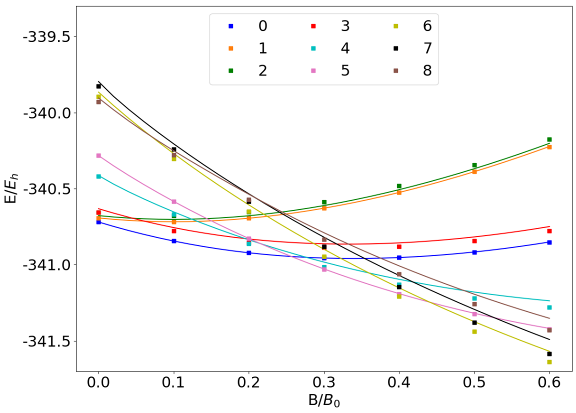

Figure S424: Total energies of all considered states of the P atom in the HGBSP2-9 basis set in fully uncontracted form (solid lines). The FEM values are shown by the squares of the same color.

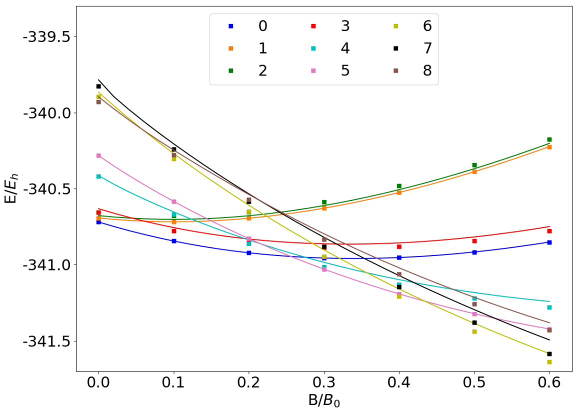

Figure S426: Total energies of all considered states of the P atom in the HGBSP3-7 basis set in fully uncontracted form (solid lines). The FEM values are shown by the squares of the same color.

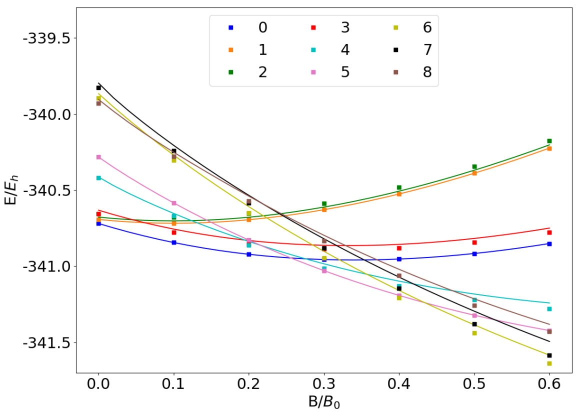

Figure S427: Total energies of all considered states of the P atom in the HGBSP3-9 basis set in fully uncontracted form (solid lines). The FEM values are shown by the squares of the same color.

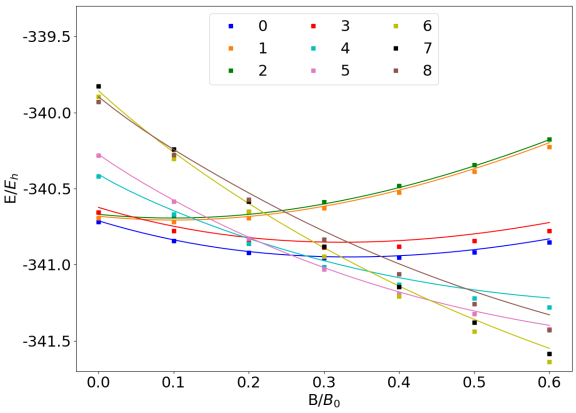

Figure S428: Total energies of all considered states of the P atom in the AHGBSP1-5 basis set in fully uncontracted form (solid lines). The FEM values are shown by the squares of the same color.

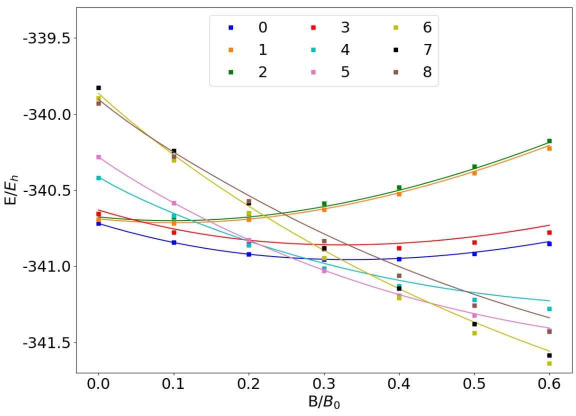

Figure S429: Total energies of all considered states of the P atom in the AHGBSP1-7 basis set in fully uncontracted form (solid lines). The FEM values are shown by the squares of the same color.

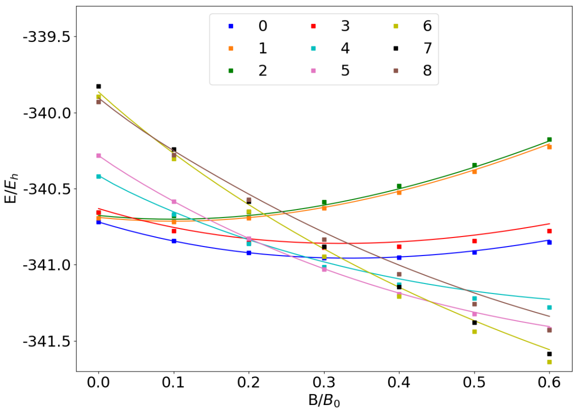

Figure S430: Total energies of all considered states of the P atom in the AHGBSP1-9 basis set in fully uncontracted form (solid lines). The FEM values are shown by the squares of the same color.

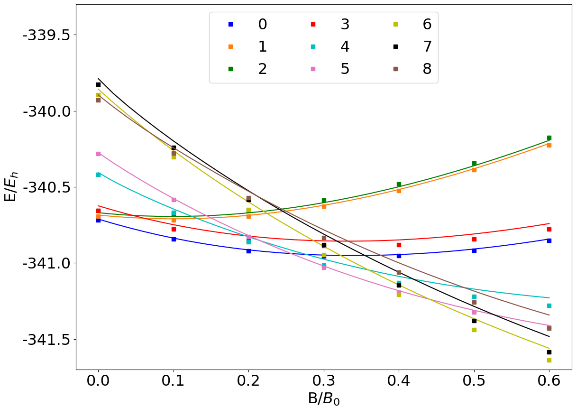

Figure S431: Total energies of all considered states of the P atom in the AHGBSP2-5 basis set in fully uncontracted form (solid lines). The FEM values are shown by the squares of the same color.

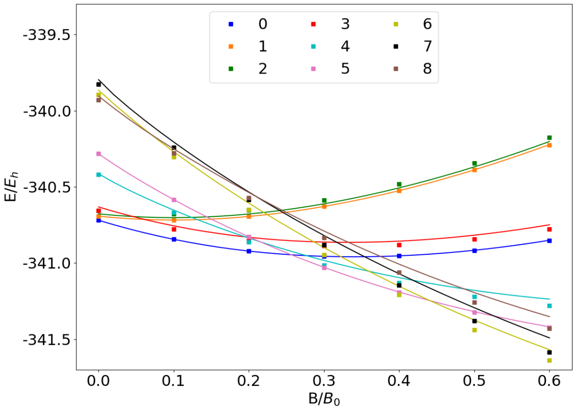

Figure S432: Total energies of all considered states of the P atom in the AHGBSP2-7 basis set in fully uncontracted form (solid lines). The FEM values are shown by the squares of the same color.

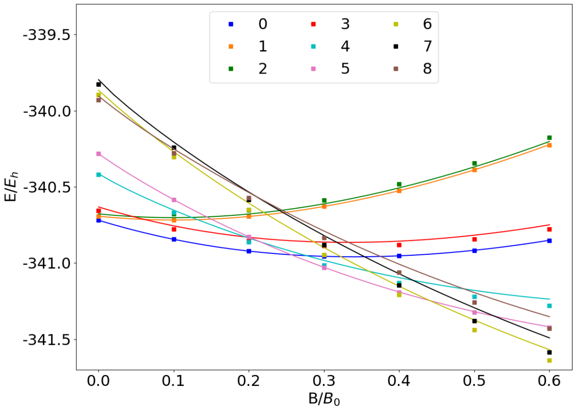

Figure S433: Total energies of all considered states of the P atom in the AHGBSP2-9 basis set in fully uncontracted form (solid lines). The FEM values are shown by the squares of the same color.

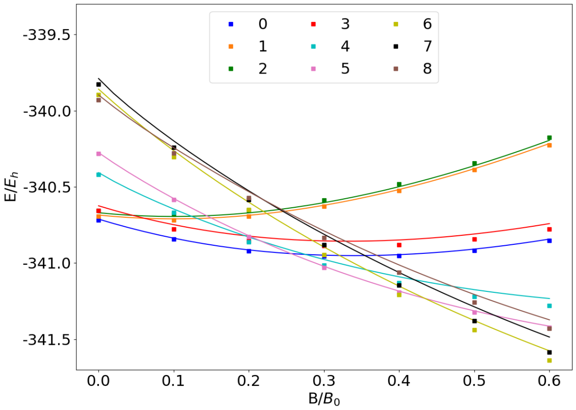

Figure S434: Total energies of all considered states of the P atom in the AHGBSP3-5 basis set in fully uncontracted form (solid lines). The FEM values are shown by the squares of the same color.

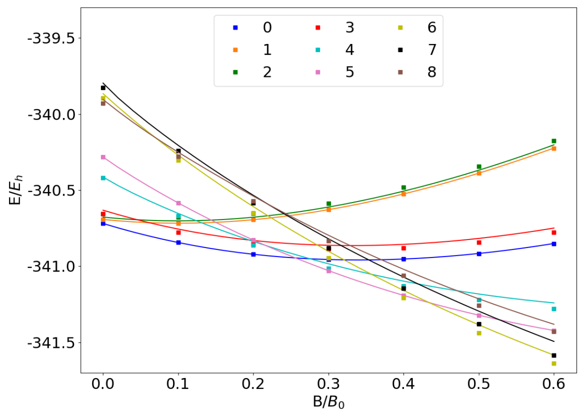

Figure S435: Total energies of all considered states of the P atom in the AHGBSP3-7 basis set in fully uncontracted form (solid lines). The FEM values are shown by the squares of the same color.

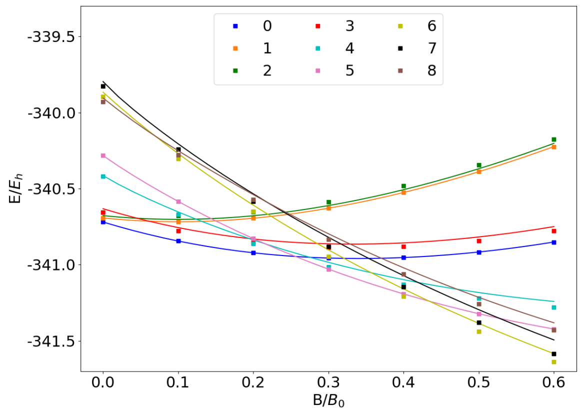

Figure S436: Total energies of all considered states of the P atom in the AHGBSP3-9 basis set in fully uncontracted form (solid lines). The FEM values are shown by the squares of the same color.

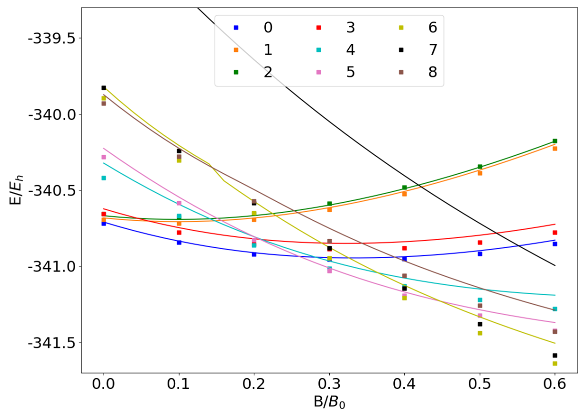

Figure S437: Total energies of all considered states of the P atom in the 6-311++G(3df,3pd) basis set in fully uncontracted form (solid lines). The FEM values are shown by the squares of the same color.

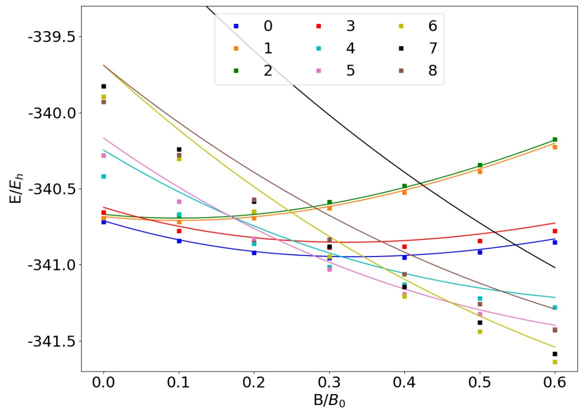

Figure S438: Total energies of all considered states of the P atom in the def2-TZVP basis set in fully uncontracted form (solid lines). The FEM values are shown by the squares of the same color.

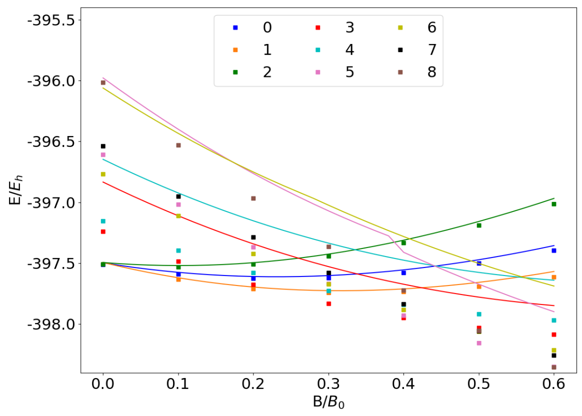

Figure S439: Total energies of all considered states of the S atom in the cc-pVDZ basis set in fully uncontracted form (solid lines). The FEM values are shown by the squares of the same color.

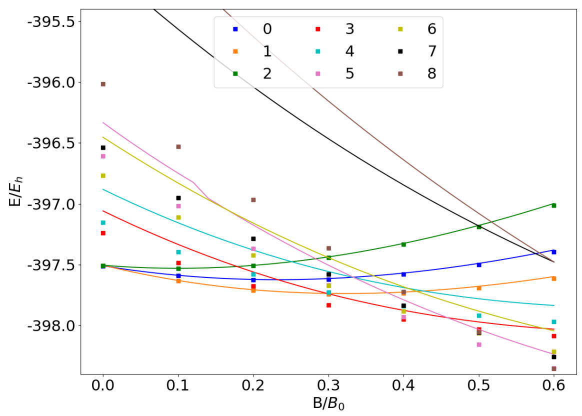

Figure S440: Total energies of all considered states of the S atom in the cc-pVTZ basis set in fully uncontracted form (solid lines). The FEM values are shown by the squares of the same color.

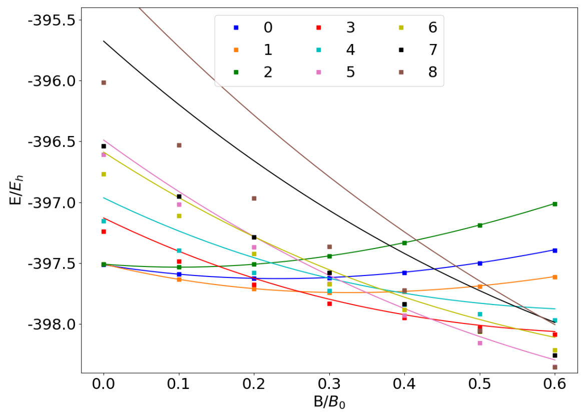

Figure S441: Total energies of all considered states of the S atom in the cc-pVQZ basis set in fully uncontracted form (solid lines). The FEM values are shown by the squares of the same color.

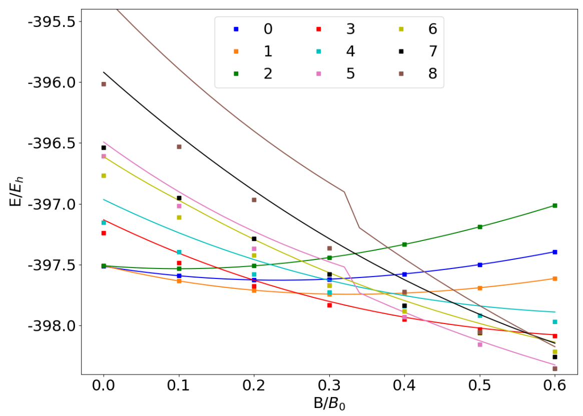

Figure S442: Total energies of all considered states of the S atom in the cc-pV5Z basis set in fully uncontracted form (solid lines). The FEM values are shown by the squares of the same color.

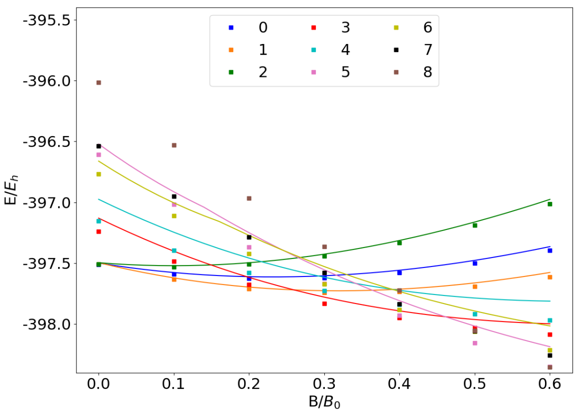

Figure S443: Total energies of all considered states of the S atom in the aug-cc-pVDZ basis set in fully uncontracted form (solid lines). The FEM values are shown by the squares of the same color.

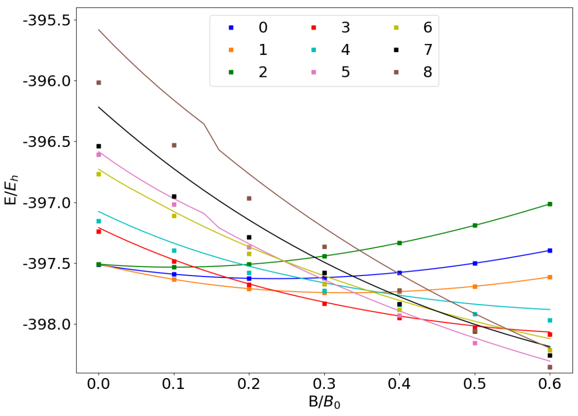

Figure S445: Total energies of all considered states of the S atom in the aug-cc-pVQZ basis set in fully uncontracted form (solid lines). The FEM values are shown by the squares of the same color.

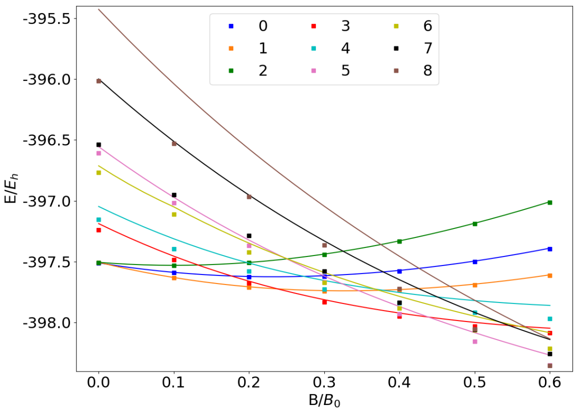

Figure S444: Total energies of all considered states of the S atom in the aug-cc-pVTZ basis set in fully uncontracted form (solid lines). The FEM values are shown by the squares of the same color.

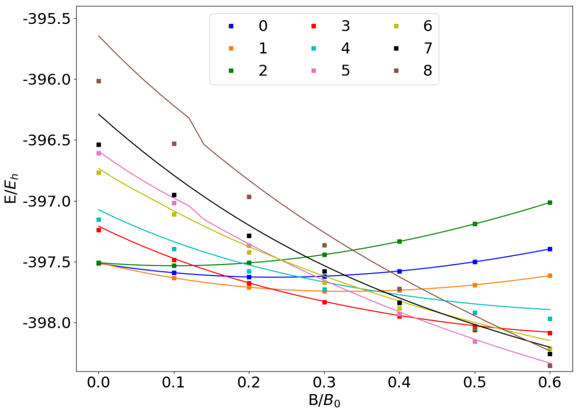

Figure S446: Total energies of all considered states of the S atom in the aug-cc-pV5Z basis set in fully uncontracted form (solid lines). The FEM values are shown by the squares of the same color.

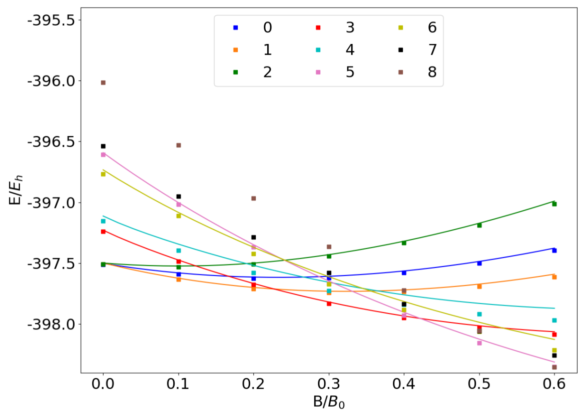

Figure S447: Total energies of all considered states of the S atom in the HGBSP1-5 basis set in fully uncontracted form (solid lines). The FEM values are shown by the squares of the same color.

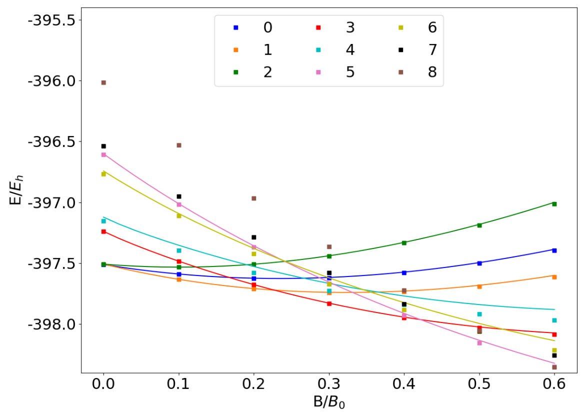

Figure S449: Total energies of all considered states of the S atom in the HGBSP1-9 basis set in fully uncontracted form (solid lines). The FEM values are shown by the squares of the same color.

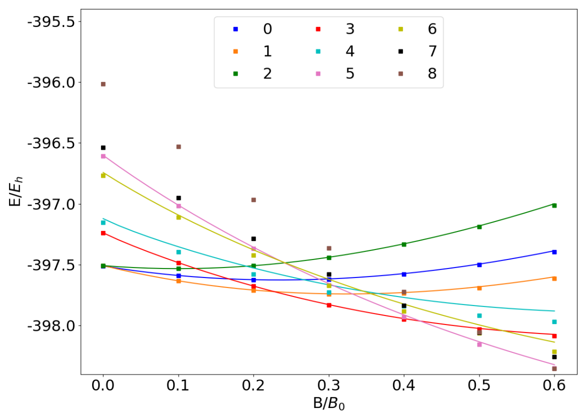

Figure S448: Total energies of all considered states of the S atom in the HGBSP1-7 basis set in fully uncontracted form (solid lines). The FEM values are shown by the squares of the same color.

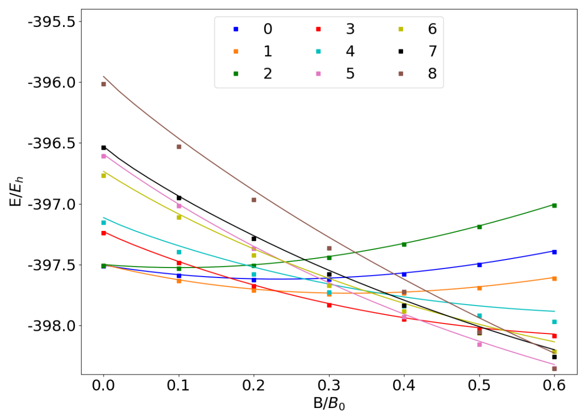

Figure S450: Total energies of all considered states of the S atom in the HGBSP2-5 basis set in fully uncontracted form (solid lines). The FEM values are shown by the squares of the same color.

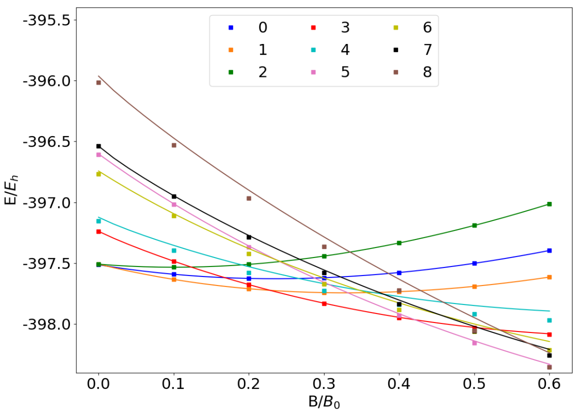

Figure S451: Total energies of all considered states of the S atom in the HGBSP2-7 basis set in fully uncontracted form (solid lines). The FEM values are shown by the squares of the same color.

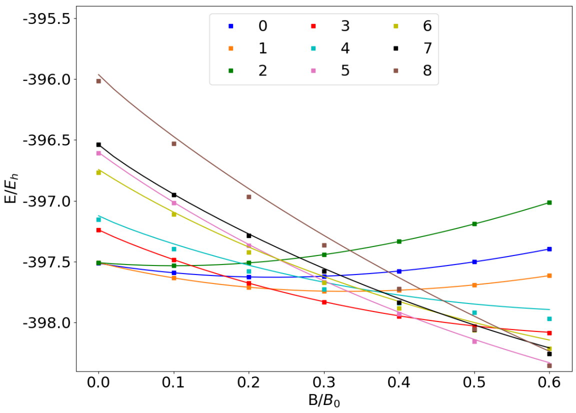

Figure S452: Total energies of all considered states of the S atom in the HGBSP2-9 basis set in fully uncontracted form (solid lines). The FEM values are shown by the squares of the same color.

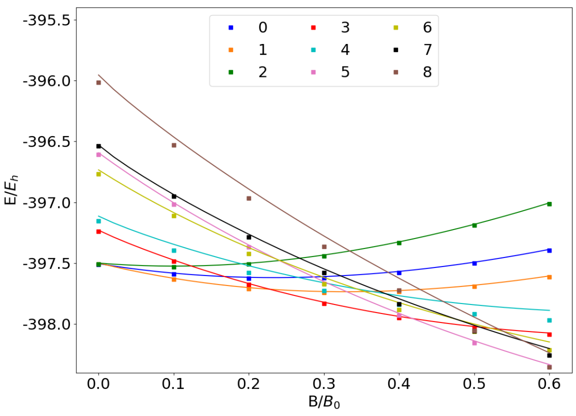

Figure S453: Total energies of all considered states of the S atom in the HGBSP3-5 basis set in fully uncontracted form (solid lines). The FEM values are shown by the squares of the same color.

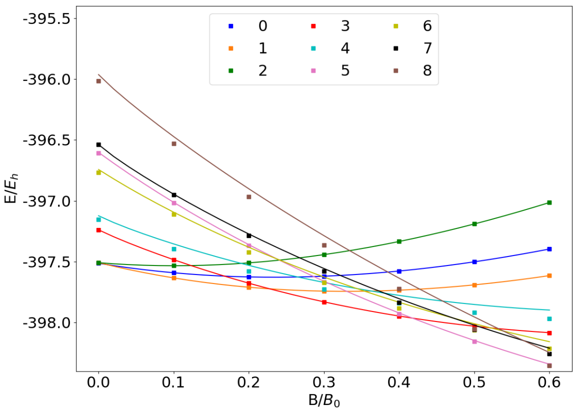

Figure S454: Total energies of all considered states of the S atom in the HGBSP3-7 basis set in fully uncontracted form (solid lines). The FEM values are shown by the squares of the same color.

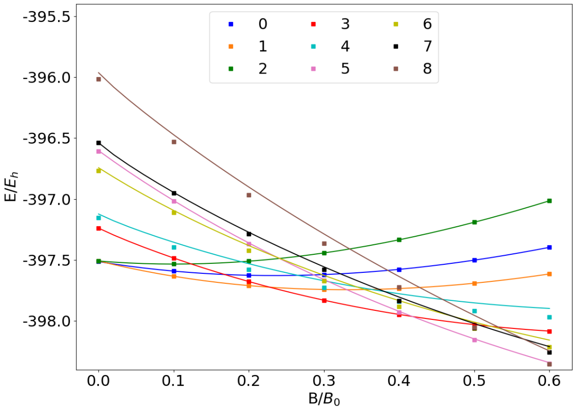

Figure S455: Total energies of all considered states of the S atom in the HGBSP3-9 basis set in fully uncontracted form (solid lines). The FEM values are shown by the squares of the same color.

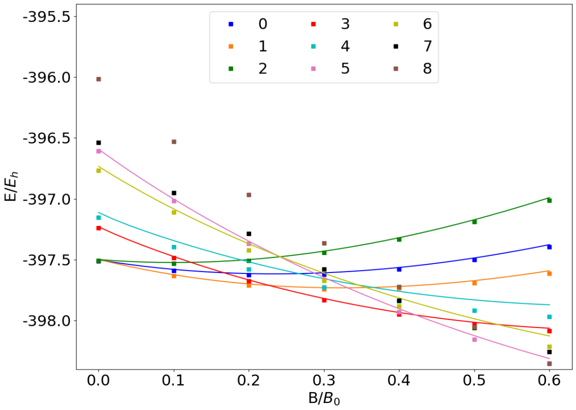

Figure S456: Total energies of all considered states of the S atom in the AHGBSP1-5 basis set in fully uncontracted form (solid lines). The FEM values are shown by the squares of the same color.

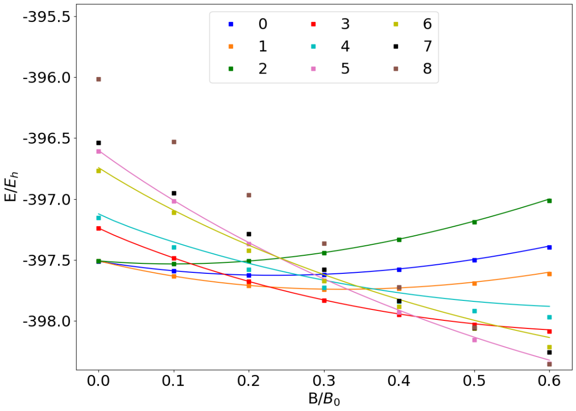

Figure S457: Total energies of all considered states of the S atom in the AHGBSP1-7 basis set in fully uncontracted form (solid lines). The FEM values are shown by the squares of the same color.

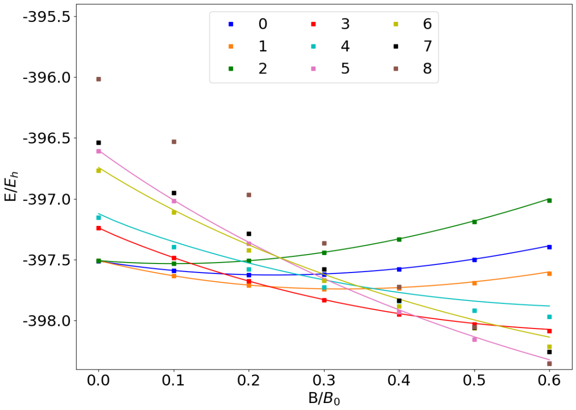

Figure S458: Total energies of all considered states of the S atom in the AHGBSP1-9 basis set in fully uncontracted form (solid lines). The FEM values are shown by the squares of the same color.

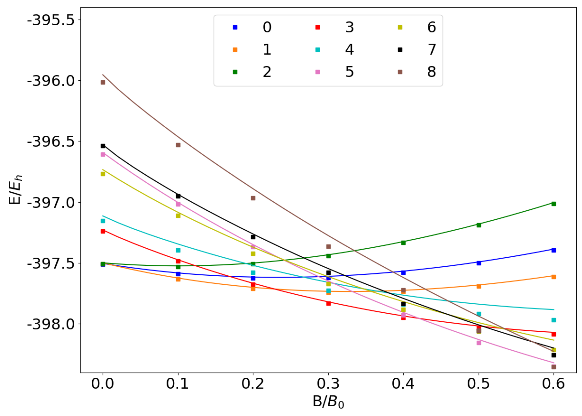

Figure S459: Total energies of all considered states of the S atom in the AHGBSP2-5 basis set in fully uncontracted form (solid lines). The FEM values are shown by the squares of the same color.

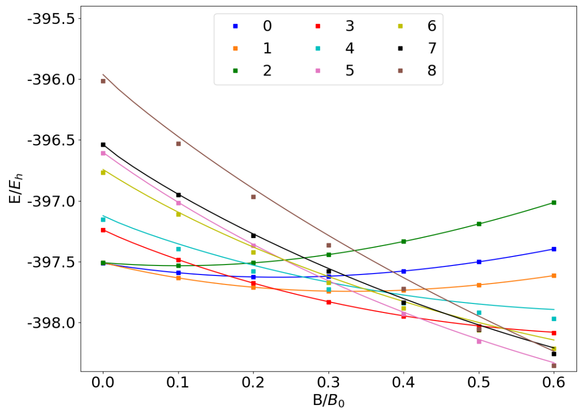

Figure S460: Total energies of all considered states of the S atom in the AHGBSP2-7 basis set in fully uncontracted form (solid lines). The FEM values are shown by the squares of the same color.

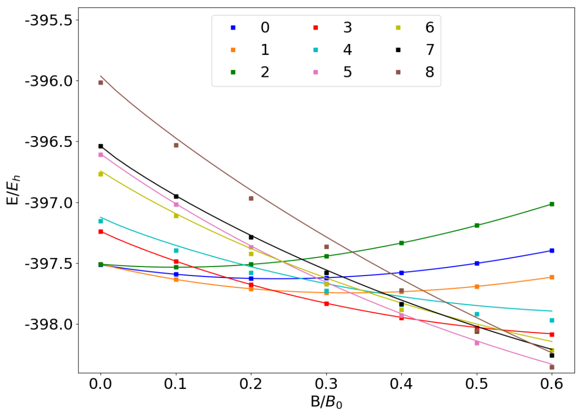

Figure S461: Total energies of all considered states of the S atom in the AHGBSP2-9 basis set in fully uncontracted form (solid lines). The FEM values are shown by the squares of the same color.

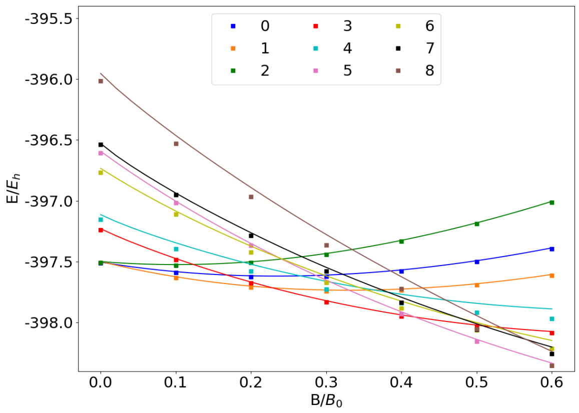

Figure S462: Total energies of all considered states of the S atom in the AHGBSP3-5 basis set in fully uncontracted form (solid lines). The FEM values are shown by the squares of the same color.

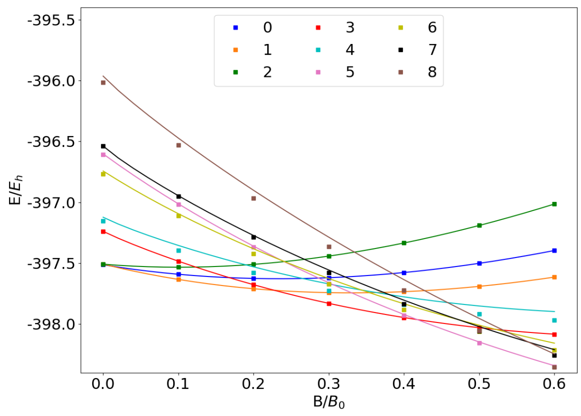

Figure S463: Total energies of all considered states of the S atom in the AHGBSP3-7 basis set in fully uncontracted form (solid lines). The FEM values are shown by the squares of the same color.

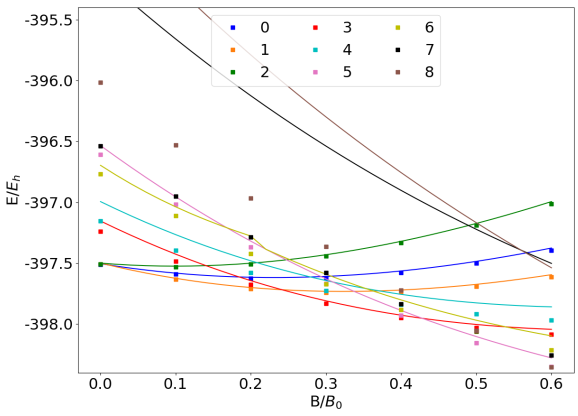

Figure S465: Total energies of all considered states of the S atom in the 6-311++G(3df,3pd) basis set in fully uncontracted form (solid lines). The FEM values are shown by the squares of the same color.

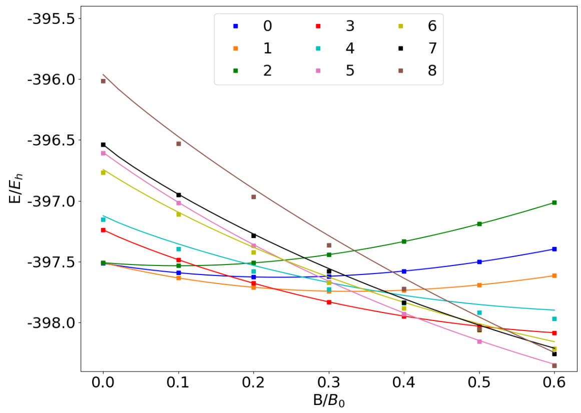

Figure S464: Total energies of all considered states of the S atom in the AHGBSP3-9 basis set in fully uncontracted form (solid lines). The FEM values are shown by the squares of the same color.

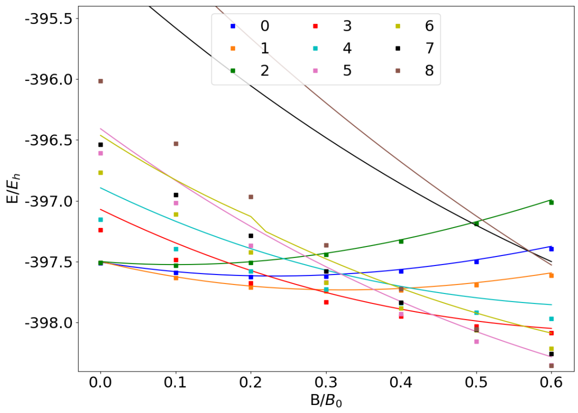

Figure S466: Total energies of all considered states of the S atom in the def2-TZVP basis set in fully uncontracted form (solid lines). The FEM values are shown by the squares of the same color.

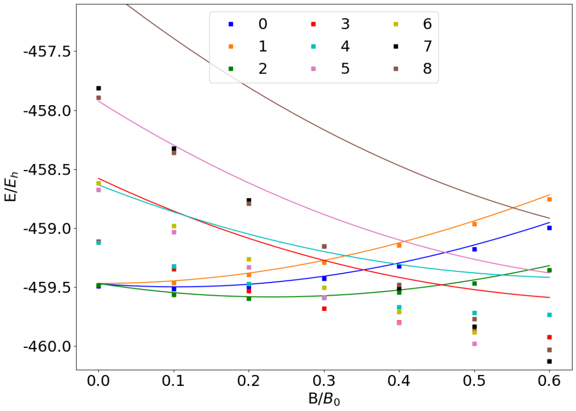

Figure S467: Total energies of all considered states of the Cl atom in the cc-pVDZ basis set in fully uncontracted form (solid lines). The FEM values are shown by the squares of the same color.

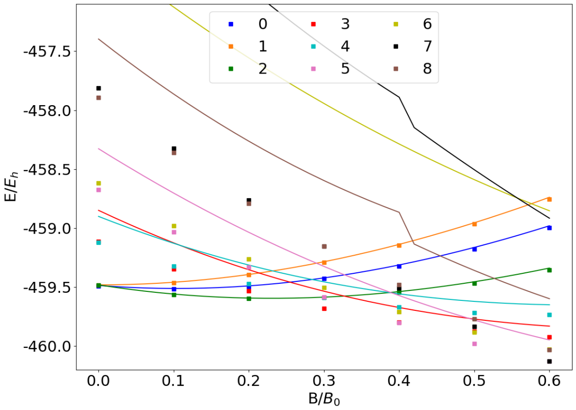

Figure S468: Total energies of all considered states of the Cl atom in the cc-pVTZ basis set in fully uncontracted form (solid lines). The FEM values are shown by the squares of the same color.

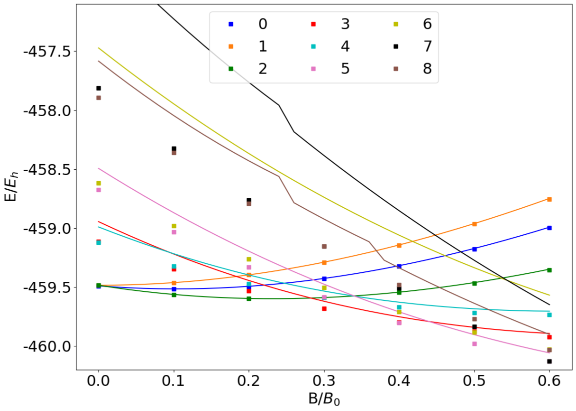

Figure S469: Total energies of all considered states of the Cl atom in the cc-pVQZ basis set in fully uncontracted form (solid lines). The FEM values are shown by the squares of the same color.

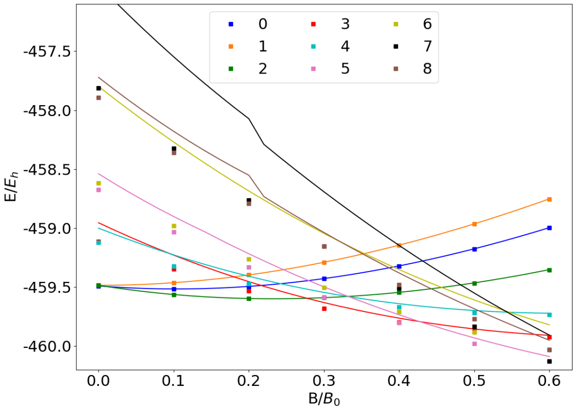

Figure S470: Total energies of all considered states of the Cl atom in the cc-pV5Z basis set in fully uncontracted form (solid lines). The FEM values are shown by the squares of the same color.

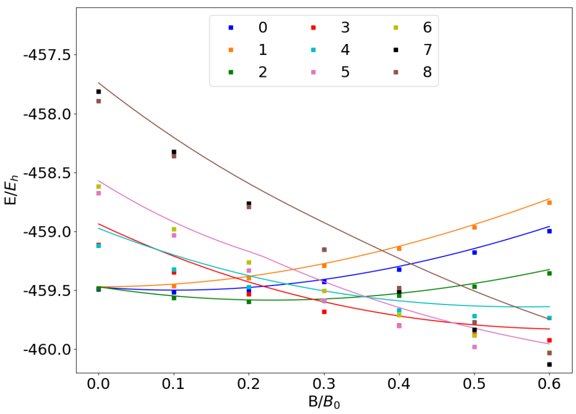

Figure S471: Total energies of all considered states of the Cl atom in the aug-cc-pVDZ basis set in fully uncontracted form (solid lines). The FEM values are shown by the squares of the same color.

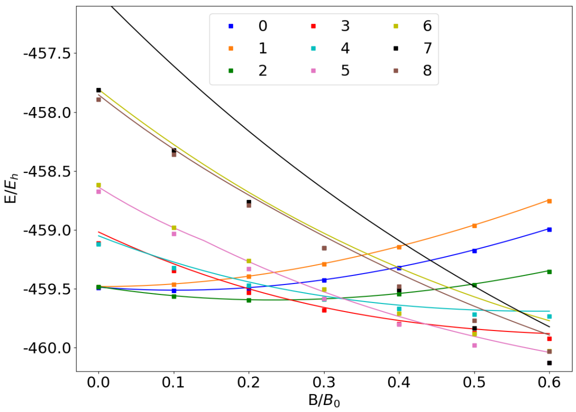

Figure S472: Total energies of all considered states of the Cl atom in the aug-cc-pVTZ basis set in fully uncontracted form (solid lines). The FEM values are shown by the squares of the same color.

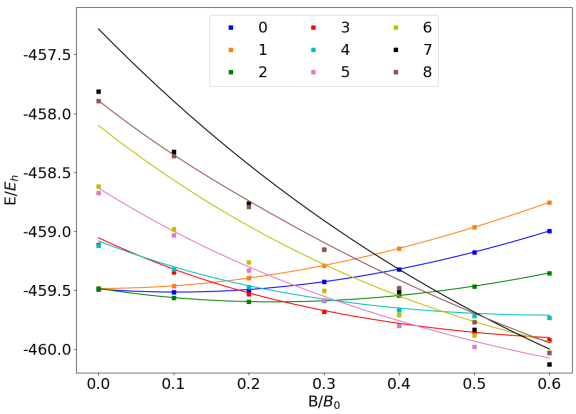

Figure S473: Total energies of all considered states of the Cl atom in the aug-cc-pVQZ basis set in fully uncontracted form (solid lines). The FEM values are shown by the squares of the same color.

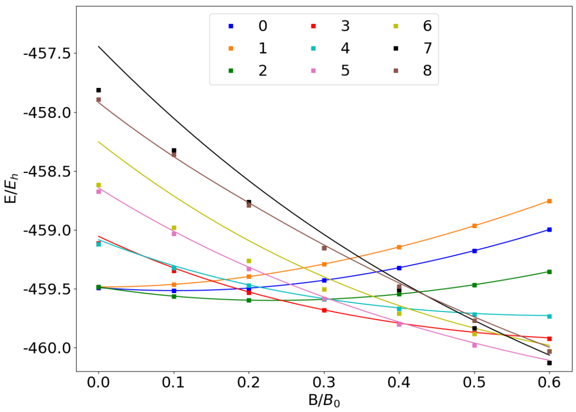

Figure S474: Total energies of all considered states of the Cl atom in the aug-cc-pV5Z basis set in fully uncontracted form (solid lines). The FEM values are shown by the squares of the same color.

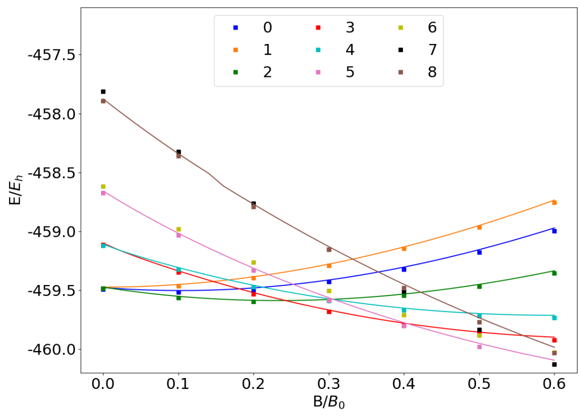

Figure S475: Total energies of all considered states of the Cl atom in the HGBSP1-5 basis set in fully uncontracted form (solid lines). The FEM values are shown by the squares of the same color.

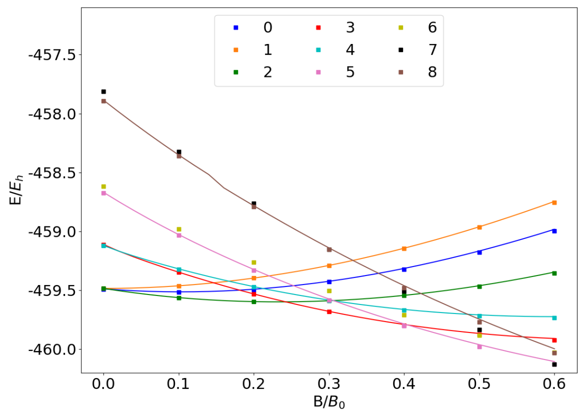

Figure S477: Total energies of all considered states of the Cl atom in the HGBSP1-9 basis set in fully uncontracted form (solid lines). The FEM values are shown by the squares of the same color.

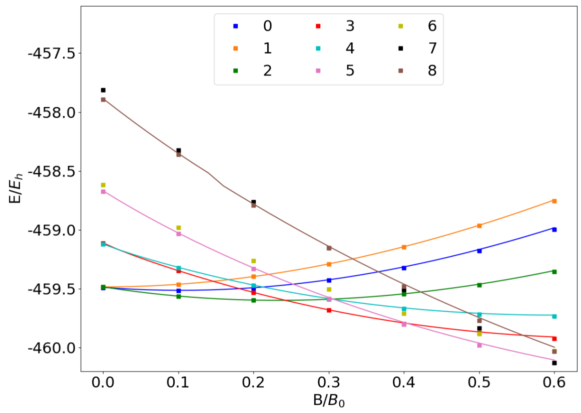

Figure S476: Total energies of all considered states of the Cl atom in the HGBSP1-7 basis set in fully uncontracted form (solid lines). The FEM values are shown by the squares of the same color.

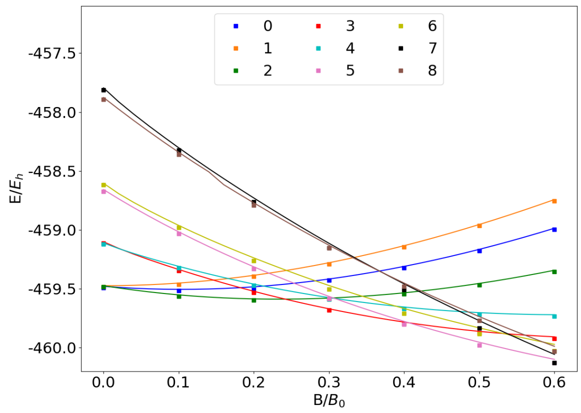

Figure S478: Total energies of all considered states of the Cl atom in the HGBSP2-5 basis set in fully uncontracted form (solid lines). The FEM values are shown by the squares of the same color.

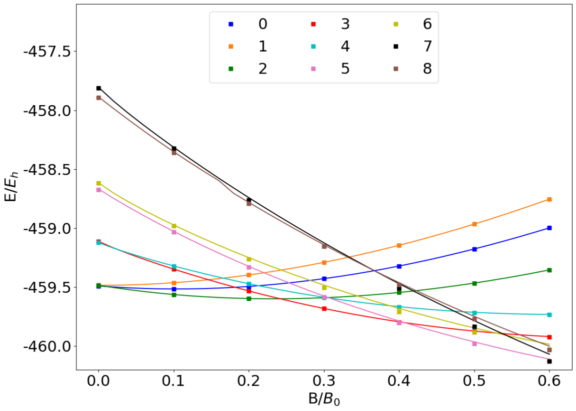

Figure S479: Total energies of all considered states of the Cl atom in the HGBSP2-7 basis set in fully uncontracted form (solid lines). The FEM values are shown by the squares of the same color.

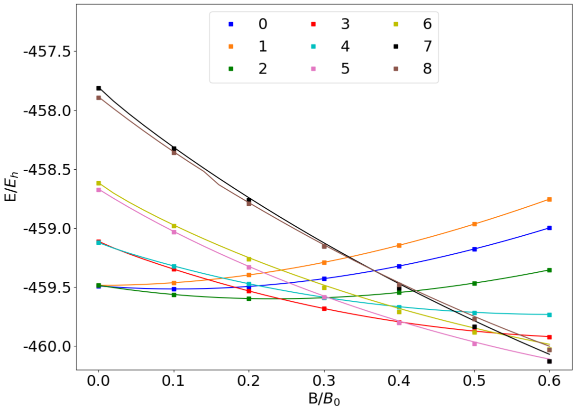

Figure S480: Total energies of all considered states of the Cl atom in the HGBSP2-9 basis set in fully uncontracted form (solid lines). The FEM values are shown by the squares of the same color.

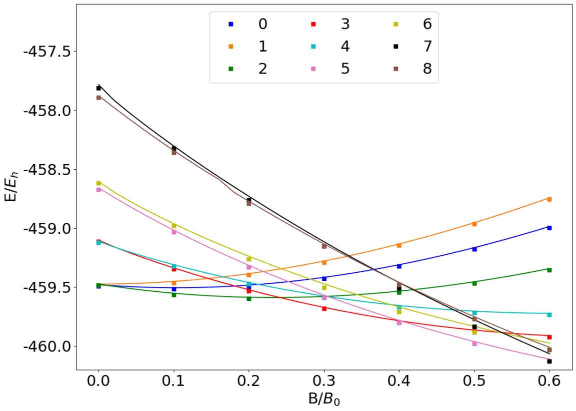

Figure S481: Total energies of all considered states of the Cl atom in the HGBSP3-5 basis set in fully uncontracted form (solid lines). The FEM values are shown by the squares of the same color.

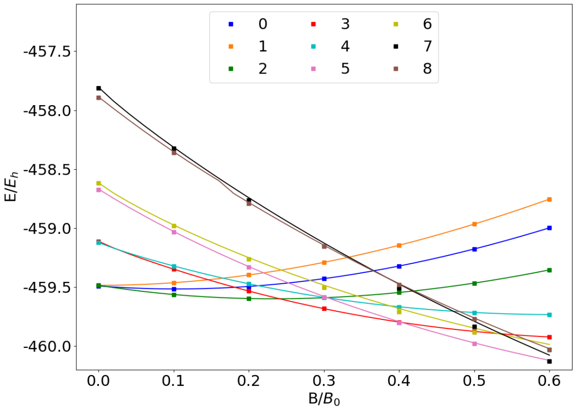

Figure S482: Total energies of all considered states of the Cl atom in the HGBSP3-7 basis set in fully uncontracted form (solid lines). The FEM values are shown by the squares of the same color.

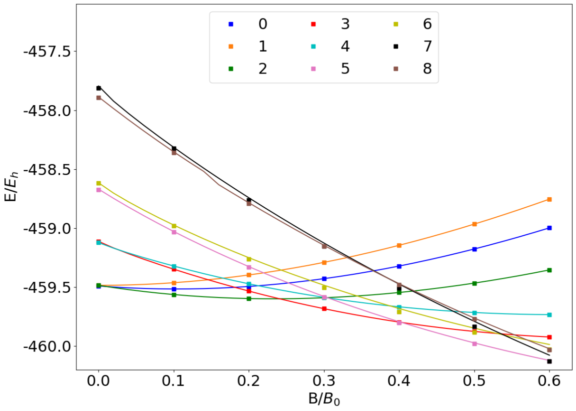

Figure S483: Total energies of all considered states of the Cl atom in the HGBSP3-9 basis set in fully uncontracted form (solid lines). The FEM values are shown by the squares of the same color.

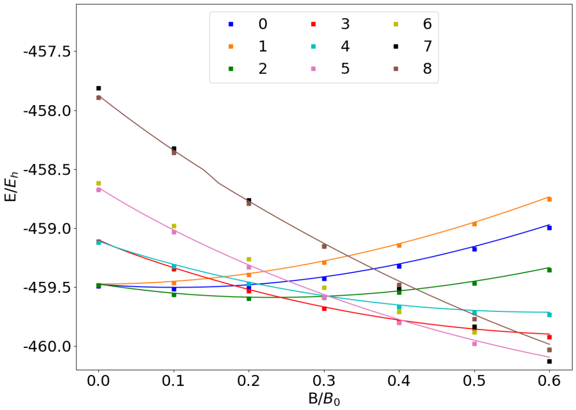

Figure S484: Total energies of all considered states of the Cl atom in the AHGBSP1-5 basis set in fully uncontracted form (solid lines). The FEM values are shown by the squares of the same color.

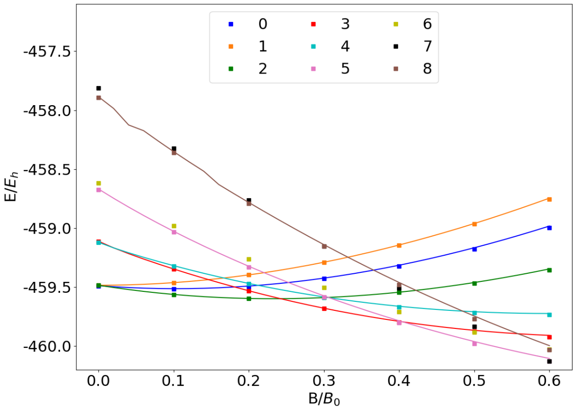

Figure S485: Total energies of all considered states of the Cl atom in the AHGBSP1-7 basis set in fully uncontracted form (solid lines). The FEM values are shown by the squares of the same color.

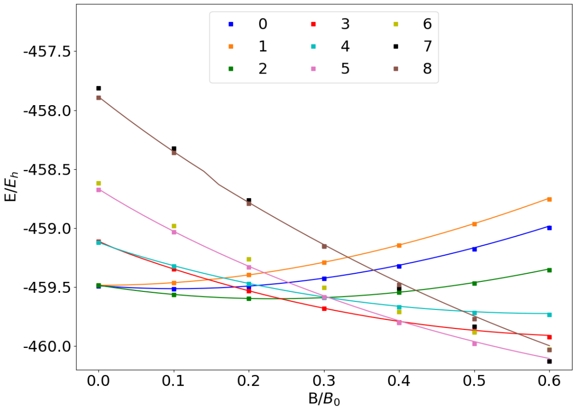

Figure S486: Total energies of all considered states of the Cl atom in the AHGBSP1-9 basis set in fully uncontracted form (solid lines). The FEM values are shown by the squares of the same color.

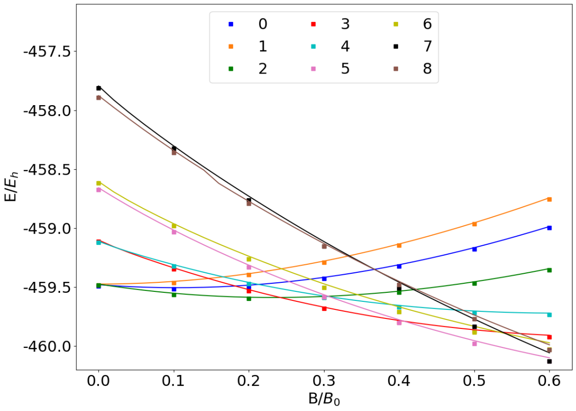

Figure S487: Total energies of all considered states of the Cl atom in the AHGBSP2-5 basis set in fully uncontracted form (solid lines). The FEM values are shown by the squares of the same color.

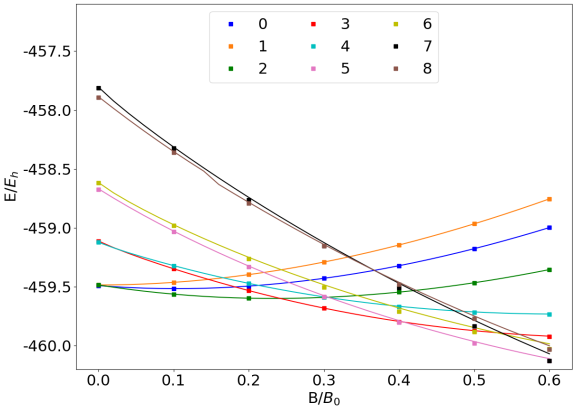

Figure S488: Total energies of all considered states of the Cl atom in the AHGBSP2-7 basis set in fully uncontracted form (solid lines). The FEM values are shown by the squares of the same color.

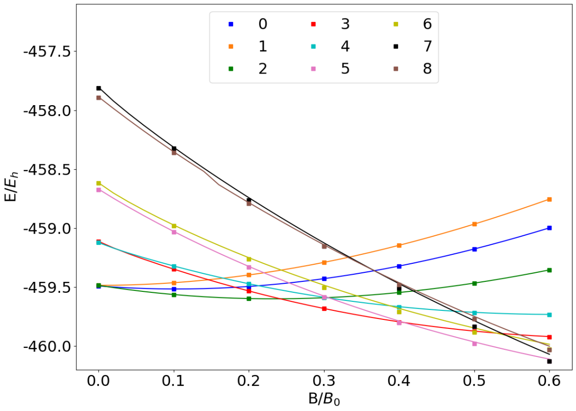

Figure S489: Total energies of all considered states of the Cl atom in the AHGBSP2-9 basis set in fully uncontracted form (solid lines). The FEM values are shown by the squares of the same color.

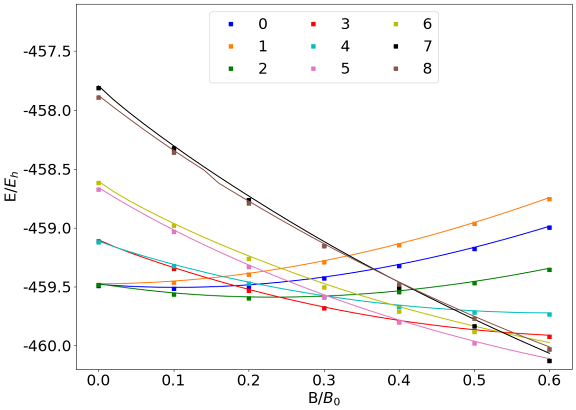

Figure S490: Total energies of all considered states of the Cl atom in the AHGBSP3-5 basis set in fully uncontracted form (solid lines). The FEM values are shown by the squares of the same color.

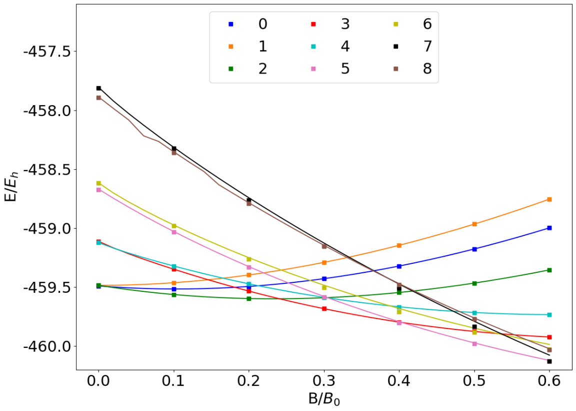

Figure S491: Total energies of all considered states of the Cl atom in the AHGBSP3-7 basis set in fully uncontracted form (solid lines). The FEM values are shown by the squares of the same color.

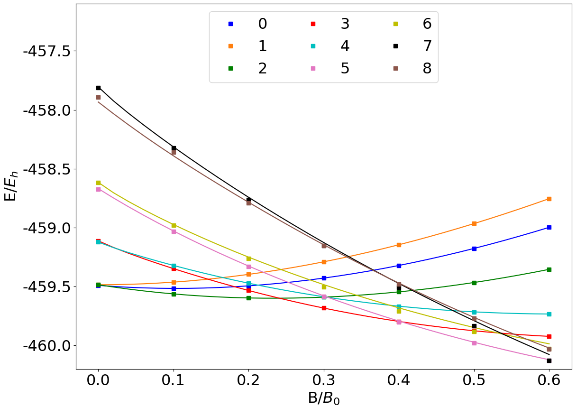

Figure S492: Total energies of all considered states of the Cl atom in the AHGBSP3-9 basis set in fully uncontracted form (solid lines). The FEM values are shown by the squares of the same color.

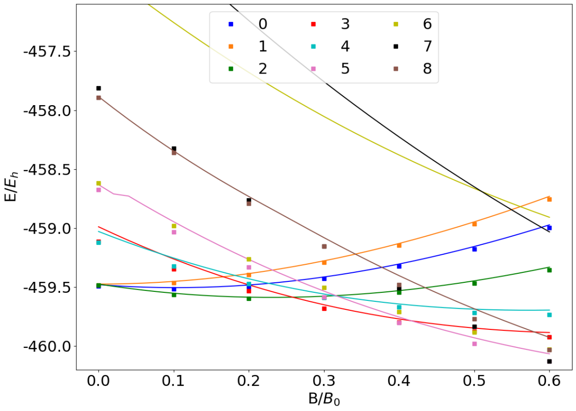

Figure S493: Total energies of all considered states of the Cl atom in the 6-311++G(3df,3pd) basis set in fully uncontracted form (solid lines). The FEM values are shown by the squares of the same color.

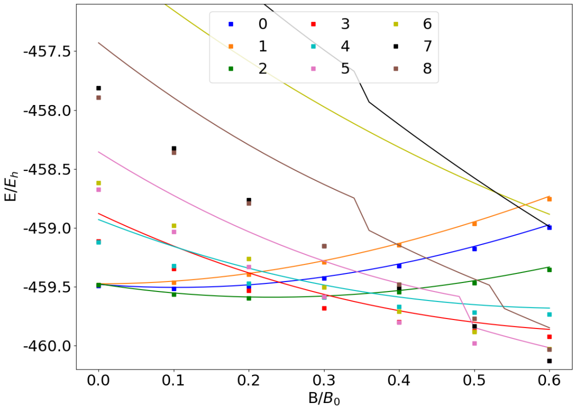

Figure S494: Total energies of all considered states of the Cl atom in the def2-TZVP basis set in fully uncontracted form (solid lines). The FEM values are shown by the squares of the same color.

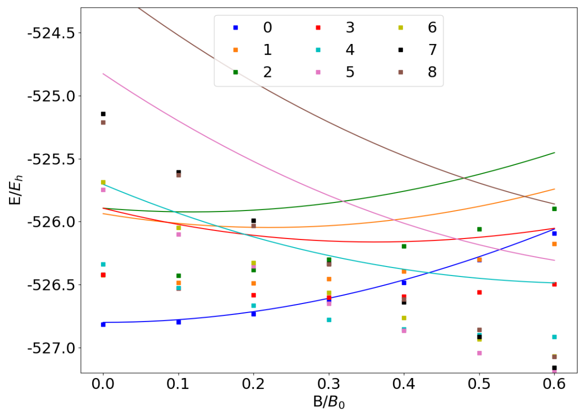

Figure S495: Total energies of all considered states of the Ar atom in the cc-pVDZ basis set in fully uncontracted form (solid lines). The FEM values are shown by the squares of the same color.

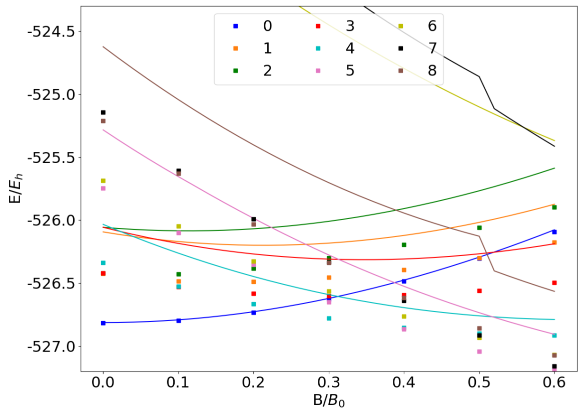

Figure S496: Total energies of all considered states of the Ar atom in the cc-pVTZ basis set in fully uncontracted form (solid lines). The FEM values are shown by the squares of the same color.

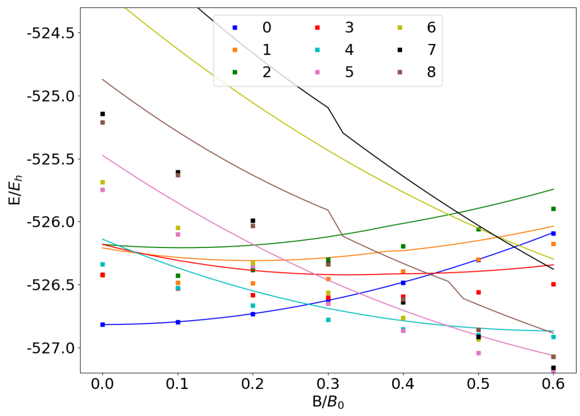

Figure S497: Total energies of all considered states of the Ar atom in the cc-pVQZ basis set in fully uncontracted form (solid lines). The FEM values are shown by the squares of the same color.

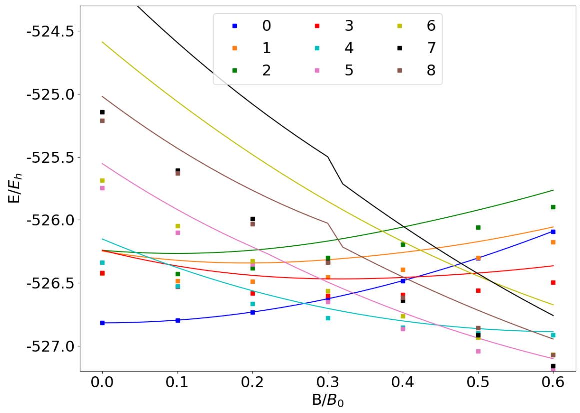

Figure S498: Total energies of all considered states of the Ar atom in the cc-pV5Z basis set in fully uncontracted form (solid lines). The FEM values are shown by the squares of the same color.

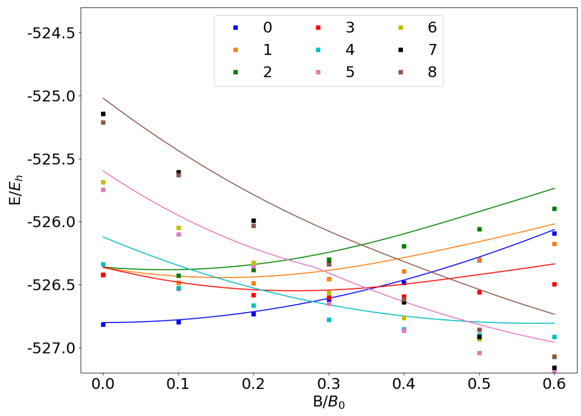

Figure S499: Total energies of all considered states of the Ar atom in the aug-cc-pVDZ basis set in fully uncontracted form (solid lines). The FEM values are shown by the squares of the same color.

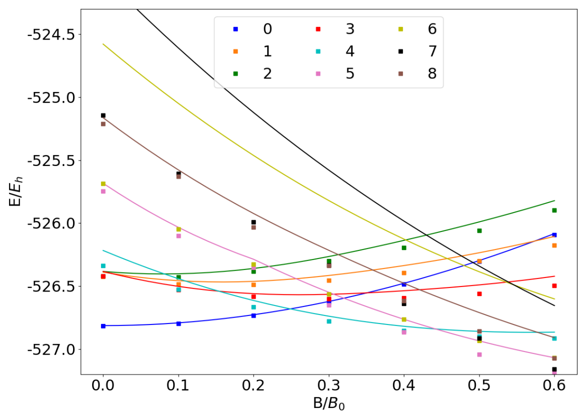

Figure S500: Total energies of all considered states of the Ar atom in the aug-cc-pVTZ basis set in fully uncontracted form (solid lines). The FEM values are shown by the squares of the same color.

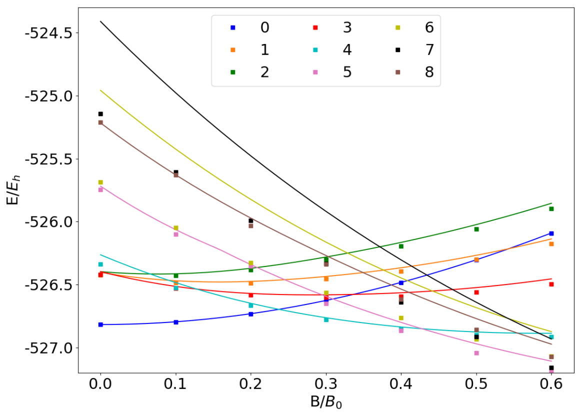

Figure S501: Total energies of all considered states of the Ar atom in the aug-cc-pVQZ basis set in fully uncontracted form (solid lines). The FEM values are shown by the squares of the same color.

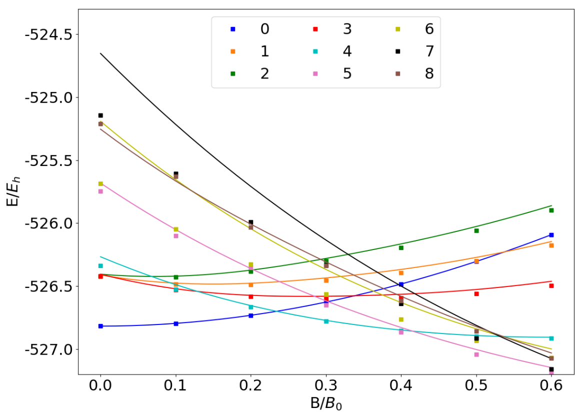

Figure S502: Total energies of all considered states of the Ar atom in the aug-cc-pV5Z basis set in fully uncontracted form (solid lines). The FEM values are shown by the squares of the same color.

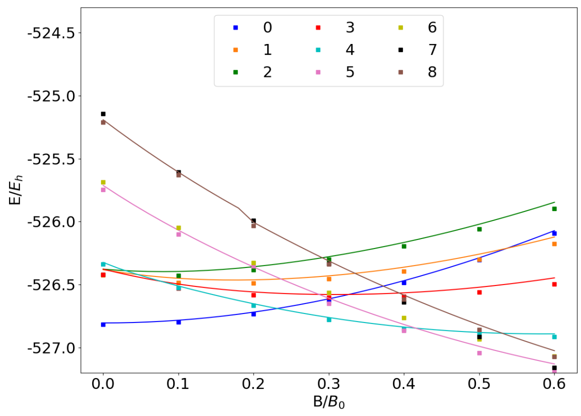

Figure S503: Total energies of all considered states of the Ar atom in the HGBSP1-5 basis set in fully uncontracted form (solid lines). The FEM values are shown by the squares of the same color.

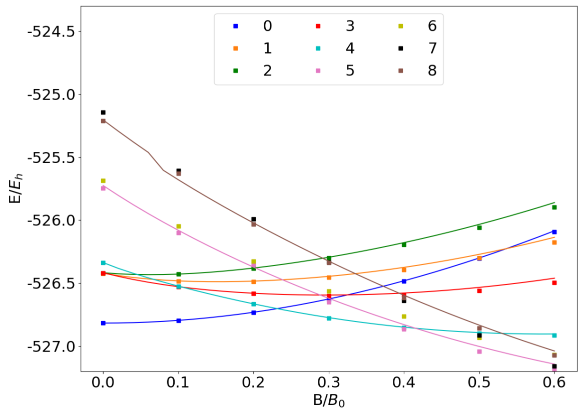

Figure S504: Total energies of all considered states of the Ar atom in the HGBSP1-7 basis set in fully uncontracted form (solid lines). The FEM values are shown by the squares of the same color.

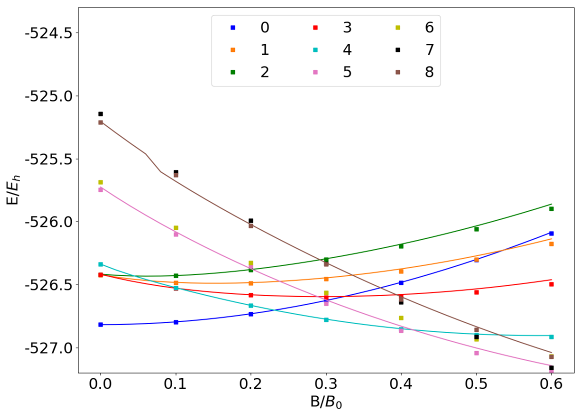

Figure S505: Total energies of all considered states of the Ar atom in the HGBSP1-9 basis set in fully uncontracted form (solid lines). The FEM values are shown by the squares of the same color.

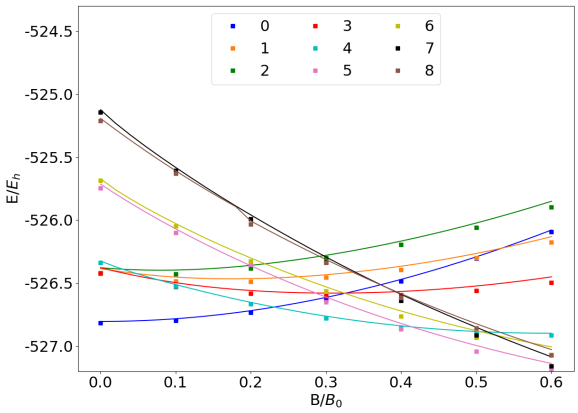

Figure S506: Total energies of all considered states of the Ar atom in the HGBSP2-5 basis set in fully uncontracted form (solid lines). The FEM values are shown by the squares of the same color.

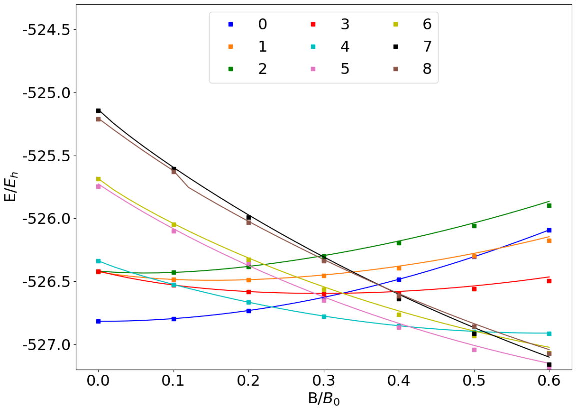

Figure S507: Total energies of all considered states of the Ar atom in the HGBSP2-7 basis set in fully uncontracted form (solid lines). The FEM values are shown by the squares of the same color.

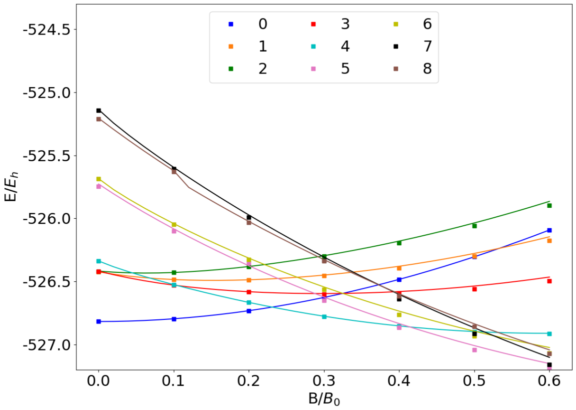

Figure S508: Total energies of all considered states of the Ar atom in the HGBSP2-9 basis set in fully uncontracted form (solid lines). The FEM values are shown by the squares of the same color.

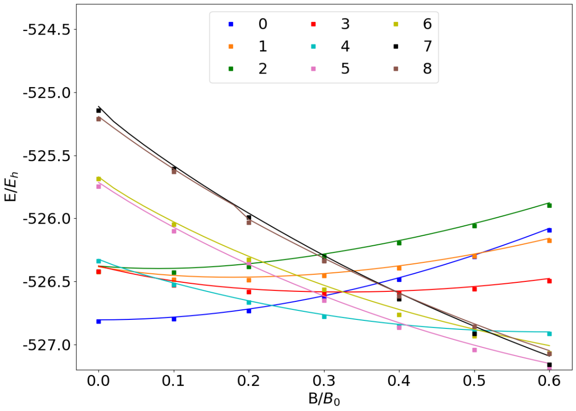

Figure S509: Total energies of all considered states of the Ar atom in the HGBSP3-5 basis set in fully uncontracted form (solid lines). The FEM values are shown by the squares of the same color.

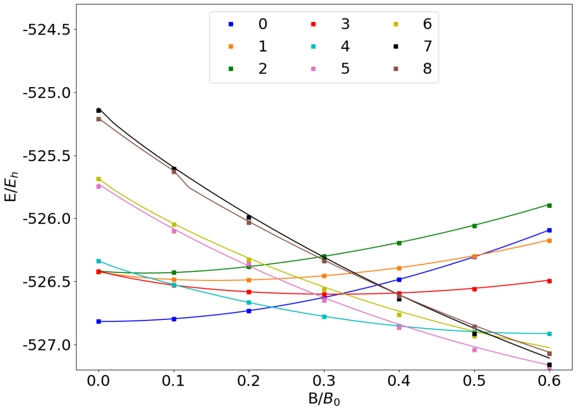

Figure S510: Total energies of all considered states of the Ar atom in the HGBSP3-7 basis set in fully uncontracted form (solid lines). The FEM values are shown by the squares of the same color.

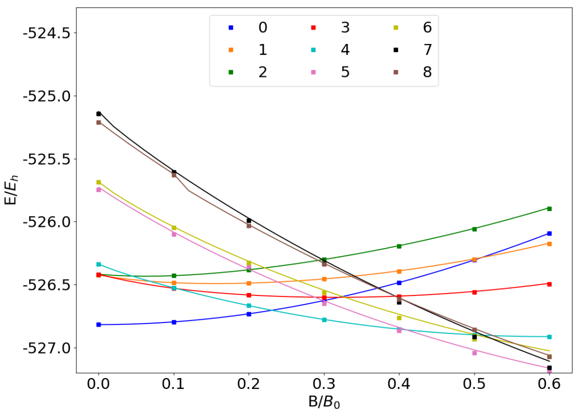

Figure S511: Total energies of all considered states of the Ar atom in the HGBSP3-9 basis set in fully uncontracted form (solid lines). The FEM values are shown by the squares of the same color.

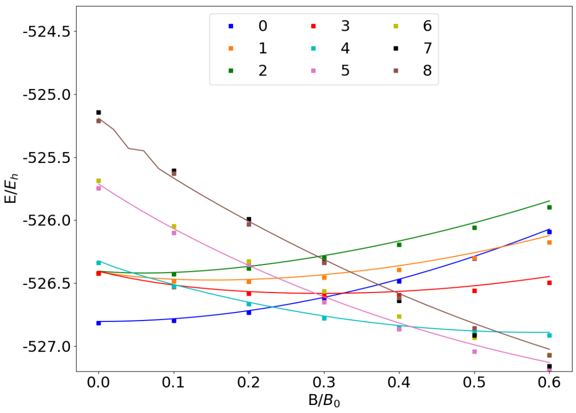

Figure S512: Total energies of all considered states of the Ar atom in the AHGBSP1-5 basis set in fully uncontracted form (solid lines). The FEM values are shown by the squares of the same color.

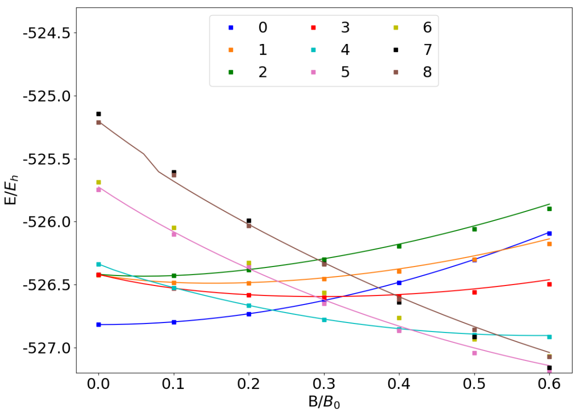

Figure S513: Total energies of all considered states of the Ar atom in the AHGBSP1-7 basis set in fully uncontracted form (solid lines). The FEM values are shown by the squares of the same color.

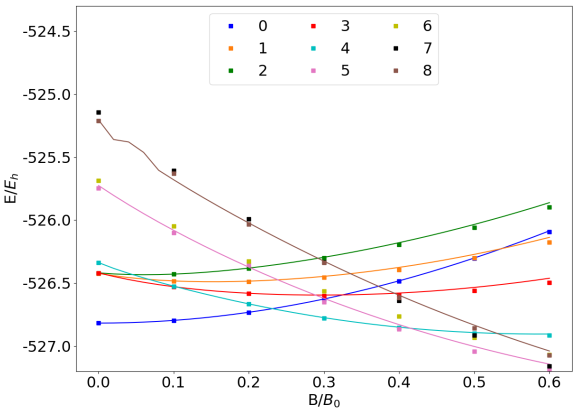

Figure S514: Total energies of all considered states of the Ar atom in the AHGBSP1-9 basis set in fully uncontracted form (solid lines). The FEM values are shown by the squares of the same color.

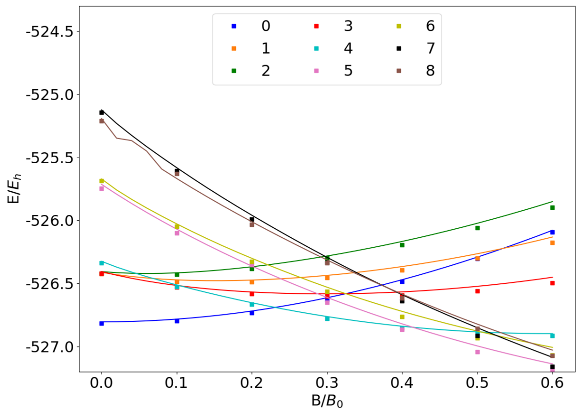

Figure S515: Total energies of all considered states of the Ar atom in the AHGBSP2-5 basis set in fully uncontracted form (solid lines). The FEM values are shown by the squares of the same color.

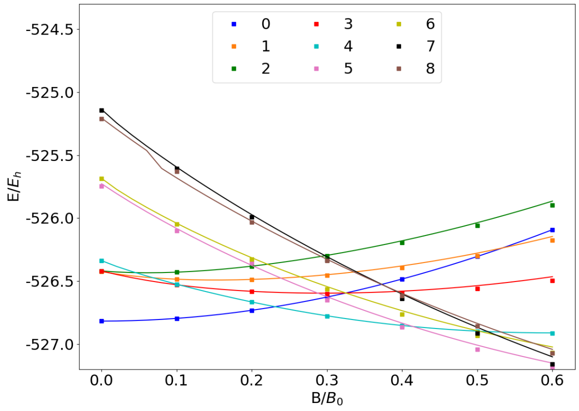

Figure S516: Total energies of all considered states of the Ar atom in the AHGBSP2-7 basis set in fully uncontracted form (solid lines). The FEM values are shown by the squares of the same color.

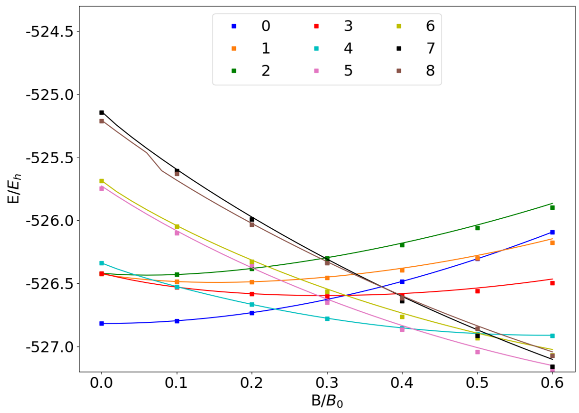

Figure S517: Total energies of all considered states of the Ar atom in the AHGBSP2-9 basis set in fully uncontracted form (solid lines). The FEM values are shown by the squares of the same color.

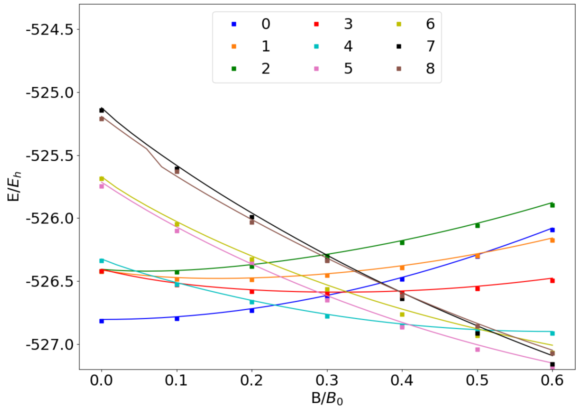

Figure S518: Total energies of all considered states of the Ar atom in the AHGBSP3-5 basis set in fully uncontracted form (solid lines). The FEM values are shown by the squares of the same color.

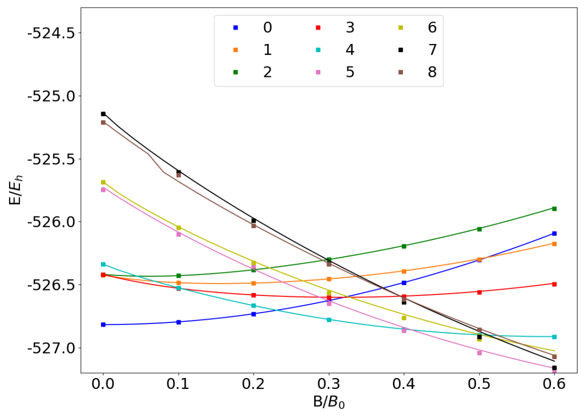

Figure S519: Total energies of all considered states of the Ar atom in the AHGBSP3-7 basis set in fully uncontracted form (solid lines). The FEM values are shown by the squares of the same color.

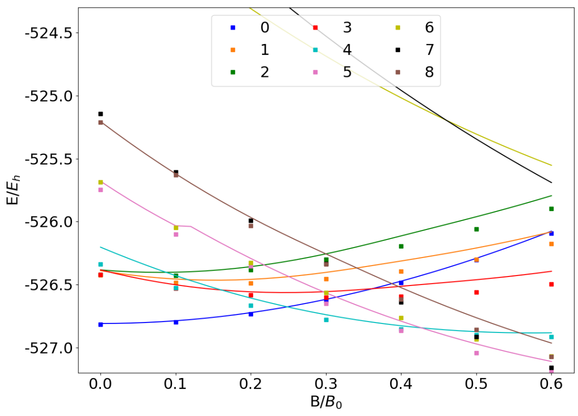

Figure S521: Total energies of all considered states of the Ar atom in the 6-311++G(3df,3pd) basis set in fully uncontracted form (solid lines). The FEM values are shown by the squares of the same color.

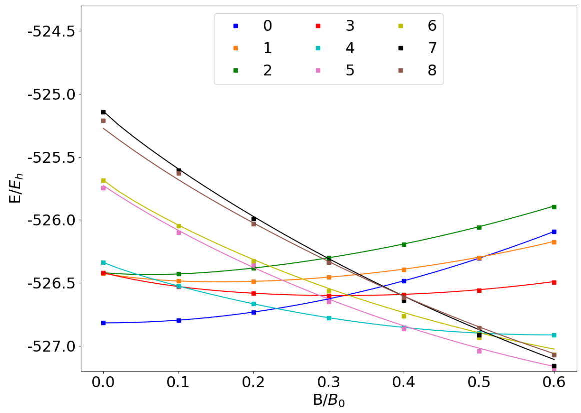

Figure S520: Total energies of all considered states of the Ar atom in the AHGBSP3-9 basis set in fully uncontracted form (solid lines). The FEM values are shown by the squares of the same color.

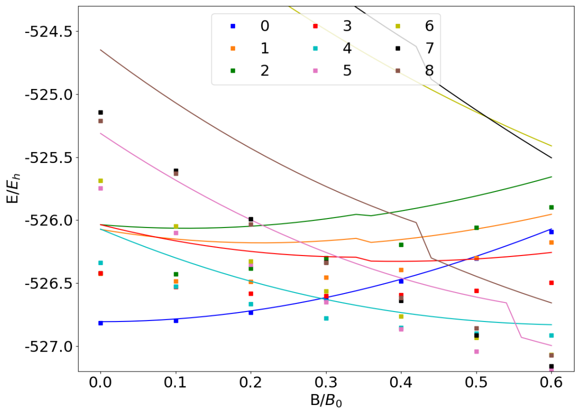

Figure S522: Total energies of all considered states of the Ar atom in the def2-TZVP basis set in fully uncontracted form (solid lines). The FEM values are shown by the squares of the same color.

Table S37: Total energies in E_h for the H atom in the cc-pVDZ basis set in fully uncontracted form, employing the real-orbital approximation.

	$0.00B_0$	$0.10B_0$	$0.20B_0$	$0.30B_0$	$0.40B_0$	$0.50B_0$	$0.60B_0$
$\sigma^{1,0}$	−0.499278	−0.546813	−0.589560	−0.627917	−0.662462	−0.693849	−0.722702
$\pi_-^{1,0}$	0.910419	0.812139	0.717297	0.625894	0.537929	0.453404	0.372317
$\pi_+^{1,0}$	0.910419	0.912139	0.917297	0.925894	0.937929	0.953404	0.972317

Table S38: Total energies in E_h for the H atom in the cc-pVTZ basis set in fully uncontracted form, employing the real-orbital approximation.

	$0.00B_0$	$0.10B_0$	$0.20B_0$	$0.30B_0$	$0.40B_0$	$0.50B_0$	$0.60B_0$
$\sigma^{1,0}$	−0.499810	−0.547333	−0.590089	−0.628571	−0.663435	−0.695334	−0.724804
$\pi_-^{1,0}$	0.298457	0.201831	0.111945	0.028778	−0.047709	−0.117569	−0.180873
$\pi_+^{1,0}$	0.298457	0.301831	0.311945	0.328778	0.352291	0.382431	0.419127

Table S39: Total energies in E_h for the H atom in the cc-pVQZ basis set in fully uncontracted form, employing the real-orbital approximation.

	$0.00B_0$	$0.10B_0$	$0.20B_0$	$0.30B_0$	$0.40B_0$	$0.50B_0$	$0.60B_0$
$\sigma^{1,0}$	−0.499946	−0.547467	−0.590244	−0.628821	−0.663869	−0.696007	−0.725715
$\pi_-^{1,0}$	0.135893	0.040609	−0.045279	−0.121870	−0.189335	−0.247922	−0.297958
$\pi_+^{1,0}$	0.135893	0.140609	0.154721	0.178130	0.210665	0.252078	0.302042

Table S40: Total energies in E_h for the H atom in the cc-pV5Z basis set in fully uncontracted form, employing the real-orbital approximation.

	$0.00B_0$	$0.10B_0$	$0.20B_0$	$0.30B_0$	$0.40B_0$	$0.50B_0$	$0.60B_0$
$\sigma^{1,0}$	−0.499995	−0.547517	−0.590319	−0.628977	−0.664146	−0.696412	−0.726249
$\pi_-^{1,0}$	0.068150	−0.026154	−0.109137	−0.181020	−0.242176	−0.293146	−0.334653
$\pi_+^{1,0}$	0.068150	0.073846	0.090863	0.118980	0.157824	0.206854	0.265347

Table S41: Total energies in E_h for the H atom in the aug-cc-pVDZ basis set in fully uncontracted form, employing the real-orbital approximation.

	$0.00B_0$	$0.10B_0$	$0.20B_0$	$0.30B_0$	$0.40B_0$	$0.50B_0$	$0.60B_0$
$\sigma^{1,0}$	−0.499337	−0.546840	−0.589562	−0.627997	−0.662722	−0.694319	−0.723341
$\pi_-^{1,0}$	−0.047865	−0.139180	−0.213234	−0.270360	−0.311163	−0.336601	−0.348103
$\pi_+^{1,0}$	−0.047865	−0.039180	−0.013234	0.029640	0.088837	0.163399	0.251897

Table S42: Total energies in E_h for the H atom in the aug-cc-pVTZ basis set in fully uncontracted form, employing the real-orbital approximation.

	$0.00B_0$	$0.10B_0$	$0.20B_0$	$0.30B_0$	$0.40B_0$	$0.50B_0$	$0.60B_0$
$\sigma^{1,0}$	−0.499821	−0.547338	−0.590135	−0.628801	−0.664030	−0.696471	−0.726650
$\pi_-^{1,0}$	−0.086914	−0.175082	−0.240204	−0.284265	−0.310938	−0.325801	−0.335694
$\pi_+^{1,0}$	−0.086914	−0.075082	−0.040204	0.015735	0.089062	0.174199	0.264306

Table S43: Total energies in E_h for the H atom in the aug-cc-pVQZ basis set in fully uncontracted form, employing the real-orbital approximation.

	$0.00B_0$	$0.10B_0$	$0.20B_0$	$0.30B_0$	$0.40B_0$	$0.50B_0$	$0.60B_0$
$\sigma^{1,0}$	−0.499948	−0.547470	−0.590298	−0.629047	−0.664416	−0.697010	−0.727283
$\pi_-^{1,0}$	−0.101001	−0.187012	−0.246542	−0.284317	−0.308316	−0.327682	−0.348417
$\pi_+^{1,0}$	−0.101001	−0.087012	−0.046542	0.015683	0.091684	0.172318	0.251583

Table S44: Total energies in E_h for the H atom in the aug-cc-pV5Z basis set in fully uncontracted form, employing the real-orbital approximation.

	$0.00B_0$	$0.10B_0$	$0.20B_0$	$0.30B_0$	$0.40B_0$	$0.50B_0$	$0.60B_0$
$\sigma^{1,0}$	−0.499995	−0.547520	−0.590366	−0.629159	−0.664575	−0.697179	−0.727406
$\pi_-^{1,0}$	−0.108260	−0.192646	−0.248359	−0.283164	−0.308302	−0.332582	−0.358331
$\pi_+^{1,0}$	−0.108260	−0.092646	−0.048359	0.016836	0.091698	0.167418	0.241669

Table S45: Total energies in E_h for the H atom in the HGBSP1-5 basis set in fully uncontracted form, employing the real-orbital approximation.

	$0.00B_0$	$0.10B_0$	$0.20B_0$	$0.30B_0$	$0.40B_0$	$0.50B_0$	$0.60B_0$
$\sigma^{1,0}$	−0.499989	−0.547508	−0.590288	−0.628874	−0.663905	−0.695933	−0.725389
$\pi_-^{1,0}$	−0.124992	−0.200506	−0.248509	−0.284941	−0.314431	−0.338567	−0.359289
$\pi_+^{1,0}$	−0.124992	−0.100506	−0.048509	0.015059	0.085569	0.161433	0.240711

Table S46: Total energies in E_h for the H atom in the HGBSP1-7 basis set in fully uncontracted form, employing the real-orbital approximation.

	$0.00B_0$	$0.10B_0$	$0.20B_0$	$0.30B_0$	$0.40B_0$	$0.50B_0$	$0.60B_0$
$\sigma^{1,0}$	−0.500000	−0.547521	−0.590317	−0.628928	−0.663962	−0.695964	−0.725396
$\pi_-^{1,0}$	−0.125000	−0.200516	−0.248798	−0.285066	−0.314519	−0.339277	−0.360638
$\pi_+^{1,0}$	−0.125000	−0.100516	−0.048798	0.014934	0.085481	0.160723	0.239362

Table S47: Total energies in E_h for the H atom in the HGBSP1-9 basis set in fully uncontracted form, employing the real-orbital approximation.

	$0.00B_0$	$0.10B_0$	$0.20B_0$	$0.30B_0$	$0.40B_0$	$0.50B_0$	$0.60B_0$
$\sigma^{1,0}$	−0.500000	−0.547522	−0.590317	−0.628928	−0.663964	−0.695973	−0.725409
$\pi_-^{1,0}$	−0.125000	−0.200522	−0.248807	−0.285107	−0.314521	−0.339326	−0.360793
$\pi_+^{1,0}$	−0.125000	−0.100522	−0.048807	0.014893	0.085479	0.160674	0.239207

Table S48: Total energies in E_h for the H atom in the HGBSP2-5 basis set in fully uncontracted form, employing the real-orbital approximation.

	$0.00B_0$	$0.10B_0$	$0.20B_0$	$0.30B_0$	$0.40B_0$	$0.50B_0$	$0.60B_0$
$\sigma^{1,0}$	−0.499989	−0.547513	−0.590354	−0.629139	−0.664549	−0.697152	−0.727378
$\pi_-^{1,0}$	−0.124992	−0.200506	−0.248509	−0.284941	−0.314431	−0.338567	−0.359289
$\pi_+^{1,0}$	−0.124992	−0.100506	−0.048509	0.015059	0.085569	0.161433	0.240711

Table S49: Total energies in E_h for the H atom in the HGBSP2-7 basis set in fully uncontracted form, employing the real-orbital approximation.

	$0.00B_0$	$0.10B_0$	$0.20B_0$	$0.30B_0$	$0.40B_0$	$0.50B_0$	$0.60B_0$
$\sigma^{1,0}$	−0.500000	−0.547526	−0.590381	−0.629185	−0.664599	−0.697191	−0.727422
$\pi_-^{1,0}$	−0.125000	−0.200516	−0.248798	−0.285066	−0.314519	−0.339277	−0.360638
$\pi_+^{1,0}$	−0.125000	−0.100516	−0.048798	0.014934	0.085481	0.160723	0.239362

Table S50: Total energies in E_h for the H atom in the HGBSP2-9 basis set in fully uncontracted form, employing the real-orbital approximation.

	$0.00B_0$	$0.10B_0$	$0.20B_0$	$0.30B_0$	$0.40B_0$	$0.50B_0$	$0.60B_0$
$\sigma^{1,0}$	−0.500000	−0.547526	−0.590381	−0.629186	−0.664601	−0.697196	−0.727430
$\pi_-^{1,0}$	−0.125000	−0.200522	−0.248807	−0.285107	−0.314521	−0.339326	−0.360793
$\pi_+^{1,0}$	−0.125000	−0.100522	−0.048807	0.014893	0.085479	0.160674	0.239207

Table S51: Total energies in E_h for the H atom in the HGBSP3-5 basis set in fully uncontracted form, employing the real-orbital approximation.

	$0.00B_0$	$0.10B_0$	$0.20B_0$	$0.30B_0$	$0.40B_0$	$0.50B_0$	$0.60B_0$
$\sigma^{1,0}$	−0.499989	−0.547513	−0.590354	−0.629139	−0.664549	−0.697152	−0.727378
$\pi_-^{1,0}$	−0.124992	−0.200821	−0.250005	−0.287045	−0.316259	−0.339911	−0.360233
$\pi_+^{1,0}$	−0.124992	−0.100821	−0.050005	0.012955	0.083741	0.160089	0.239767

Table S52: Total energies in E_h for the H atom in the HGBSP3-7 basis set in fully uncontracted form, employing the real-orbital approximation.

	$0.00B_0$	$0.10B_0$	$0.20B_0$	$0.30B_0$	$0.40B_0$	$0.50B_0$	$0.60B_0$
$\sigma^{1,0}$	−0.500000	−0.547526	−0.590381	−0.629185	−0.664599	−0.697191	−0.727422
$\pi_-^{1,0}$	−0.125000	−0.200837	−0.250472	−0.288844	−0.320853	−0.348526	−0.372859
$\pi_+^{1,0}$	−0.125000	−0.100837	−0.050472	0.011156	0.079147	0.151474	0.227141

Table S53: Total energies in E_h for the H atom in the HGBSP3-9 basis set in fully uncontracted form, employing the real-orbital approximation.

	$0.00B_0$	$0.10B_0$	$0.20B_0$	$0.30B_0$	$0.40B_0$	$0.50B_0$	$0.60B_0$
$\sigma^{1,0}$	−0.500000	−0.547526	−0.590381	−0.629186	−0.664601	−0.697196	−0.727430
$\pi_-^{1,0}$	−0.125000	−0.200842	−0.250480	−0.288874	−0.320867	−0.348602	−0.373256
$\pi_+^{1,0}$	−0.125000	−0.100842	−0.050480	0.011126	0.079133	0.151398	0.226744

Table S54: Total energies in E_h for the H atom in the AHGBSP1-5 basis set in fully uncontracted form, employing the real-orbital approximation.

	$0.00B_0$	$0.10B_0$	$0.20B_0$	$0.30B_0$	$0.40B_0$	$0.50B_0$	$0.60B_0$
$\sigma^{1,0}$	−0.499990	−0.547511	−0.590303	−0.628909	−0.663942	−0.695955	−0.725393
$\pi_-^{1,0}$	−0.124993	−0.200509	−0.248573	−0.284972	−0.314434	−0.338622	−0.359390
$\pi_+^{1,0}$	−0.124993	−0.100509	−0.048573	0.015028	0.085566	0.161378	0.240610

Table S55: Total energies in E_h for the H atom in the AHGBSP1-7 basis set in fully uncontracted form, employing the real-orbital approximation.

	$0.00B_0$	$0.10B_0$	$0.20B_0$	$0.30B_0$	$0.40B_0$	$0.50B_0$	$0.60B_0$
$\sigma^{1,0}$	−0.500000	−0.547522	−0.590317	−0.628928	−0.663963	−0.695968	−0.725403
$\pi_-^{1,0}$	−0.125000	−0.200519	−0.248800	−0.285074	−0.314520	−0.339281	−0.360651
$\pi_+^{1,0}$	−0.125000	−0.100519	−0.048800	0.014926	0.085480	0.160719	0.239349

Table S56: Total energies in E_h for the H atom in the AHGBSP1-9 basis set in fully uncontracted form, employing the real-orbital approximation.

	$0.00B_0$	$0.10B_0$	$0.20B_0$	$0.30B_0$	$0.40B_0$	$0.50B_0$	$0.60B_0$
$\sigma^{1,0}$	−0.500000	−0.547522	−0.590317	−0.628928	−0.663964	−0.695973	−0.725410
$\pi_-^{1,0}$	−0.125000	−0.200522	−0.248807	−0.285108	−0.314521	−0.339327	−0.360795
$\pi_+^{1,0}$	−0.125000	−0.100522	−0.048807	0.014892	0.085479	0.160673	0.239205

Table S57: Total energies in E_h for the H atom in the AHGBSP2-5 basis set in fully uncontracted form, employing the real-orbital approximation.

	$0.00B_0$	$0.10B_0$	$0.20B_0$	$0.30B_0$	$0.40B_0$	$0.50B_0$	$0.60B_0$
$\sigma^{1,0}$	−0.499990	−0.547515	−0.590367	−0.629166	−0.664577	−0.697168	−0.727383
$\pi_-^{1,0}$	−0.124993	−0.200509	−0.248573	−0.284972	−0.314434	−0.338622	−0.359390
$\pi_+^{1,0}$	−0.124993	−0.100509	−0.048573	0.015028	0.085566	0.161378	0.240610

Table S58: Total energies in E_h for the H atom in the AHGBSP2-7 basis set in fully uncontracted form, employing the real-orbital approximation.

	$0.00B_0$	$0.10B_0$	$0.20B_0$	$0.30B_0$	$0.40B_0$	$0.50B_0$	$0.60B_0$
$\sigma^{1,0}$	−0.500000	−0.547526	−0.590381	−0.629185	−0.664599	−0.697193	−0.727425
$\pi_-^{1,0}$	−0.125000	−0.200519	−0.248800	−0.285074	−0.314520	−0.339281	−0.360651
$\pi_+^{1,0}$	−0.125000	−0.100519	−0.048800	0.014926	0.085480	0.160719	0.239349

Table S59: Total energies in E_h for the H atom in the AHGBSP2-9 basis set in fully uncontracted form, employing the real-orbital approximation.

	$0.00B_0$	$0.10B_0$	$0.20B_0$	$0.30B_0$	$0.40B_0$	$0.50B_0$	$0.60B_0$
$\sigma^{1,0}$	−0.500000	−0.547526	−0.590381	−0.629186	−0.664601	−0.697196	−0.727430
$\pi_-^{1,0}$	−0.125000	−0.200522	−0.248807	−0.285108	−0.314521	−0.339327	−0.360795
$\pi_+^{1,0}$	−0.125000	−0.100522	−0.048807	0.014892	0.085479	0.160673	0.239205

Table S60: Total energies in E_h for the H atom in the AHGBSP3-5 basis set in fully uncontracted form, employing the real-orbital approximation.

	$0.00B_0$	$0.10B_0$	$0.20B_0$	$0.30B_0$	$0.40B_0$	$0.50B_0$	$0.60B_0$
$\sigma^{1,0}$	−0.499990	−0.547515	−0.590367	−0.629166	−0.664577	−0.697168	−0.727383
$\pi_-^{1,0}$	−0.124993	−0.200825	−0.250047	−0.287068	−0.316277	−0.339985	−0.360350
$\pi_+^{1,0}$	−0.124993	−0.100825	−0.050047	0.012932	0.083723	0.160015	0.239650

Table S61: Total energies in E_h for the H atom in the AHGBSP3-7 basis set in fully uncontracted form, employing the real-orbital approximation.

	$0.00B_0$	$0.10B_0$	$0.20B_0$	$0.30B_0$	$0.40B_0$	$0.50B_0$	$0.60B_0$
$\sigma^{1,0}$	-0.500000	-0.547526	-0.590381	-0.629185	-0.664599	-0.697193	-0.727425
$\pi_-^{1,0}$	-0.125000	-0.200839	-0.250472	-0.288849	-0.320854	-0.348528	-0.372866
$\pi_+^{1,0}$	-0.125000	-0.100839	-0.050472	0.011151	0.079146	0.151472	0.227134

Table S62: Total energies in E_h for the H atom in the AHGBSP3-9 basis set in fully uncontracted form, employing the real-orbital approximation.

	$0.00B_0$	$0.10B_0$	$0.20B_0$	$0.30B_0$	$0.40B_0$	$0.50B_0$	$0.60B_0$
$\sigma^{1,0}$	-0.500000	-0.547526	-0.590381	-0.629186	-0.664601	-0.697196	-0.727430
$\pi_-^{1,0}$	-0.125000	-0.200842	-0.250480	-0.288875	-0.320867	-0.348602	-0.373257
$\pi_+^{1,0}$	-0.125000	-0.100842	-0.050480	0.011125	0.079133	0.151398	0.226743

Table S63: Total energies in E_h for the H atom in the 6-311++G(3df,3pd) basis set in fully uncontracted form, employing the real-orbital approximation.

	$0.00B_0$	$0.10B_0$	$0.20B_0$	$0.30B_0$	$0.40B_0$	$0.50B_0$	$0.60B_0$
$\sigma^{1,0}$	-0.499818	-0.547334	-0.590108	-0.628673	-0.663649	-0.695626	-0.725110
$\pi_-^{1,0}$	0.006725	-0.086533	-0.166377	-0.233027	-0.286866	-0.328467	-0.358634
$\pi_+^{1,0}$	0.006725	0.013467	0.033623	0.066973	0.113134	0.171533	0.241366

Table S64: Total energies in E_h for the H atom in the def2-TZVP basis set in fully uncontracted form, employing the real-orbital approximation.

	$0.00B_0$	$0.10B_0$	$0.20B_0$	$0.30B_0$	$0.40B_0$	$0.50B_0$	$0.60B_0$
$\sigma^{1,0}$	-0.499810	-0.547334	-0.590088	-0.628563	-0.663407	-0.695265	-0.724665
$\pi_-^{1,0}$	1.048467	0.950030	0.854717	0.762530	0.673467	0.587530	0.504717
$\pi_+^{1,0}$	1.048467	1.050030	1.054717	1.062530	1.073467	1.087530	1.104717

Table S65: Total energies in E_h for the He atom in the cc-pVDZ basis set in fully uncontracted form, employing the real-orbital approximation.

	$0.00B_0$	$0.10B_0$	$0.20B_0$	$0.30B_0$	$0.40B_0$	$0.50B_0$	$0.60B_0$
$\sigma^{1,1}$	−2.855160	−2.853224	−2.847430	−2.837826	−2.824488	−2.807524	−2.787064
$\sigma^{2,0}$	−1.556455	−1.653027	−1.742744	−1.825610	−1.901630	−1.970815	−2.033176
$\sigma^{1,0}\pi_+^{1,0}$	−0.385211	−0.433401	−0.477979	−0.518964	−0.556393	−0.590310	−0.620775
$\sigma^{1,0}\pi_-^{1,0}$	−0.385211	−0.533401	−0.677979	−0.818964	−0.956393	−1.090310	−1.220775

Table S66: Total energies in E_h for the He atom in the cc-pVTZ basis set in fully uncontracted form, employing the real-orbital approximation.

	$0.00B_0$	$0.10B_0$	$0.20B_0$	$0.30B_0$	$0.40B_0$	$0.50B_0$	$0.60B_0$
$\sigma^{1,1}$	−2.861153	−2.859186	−2.853314	−2.843623	−2.830247	−2.813363	−2.793176
$\sigma^{2,0}$	−1.920969	−2.015758	−2.100136	−2.174148	−2.237866	−2.291394	−2.334871
$\sigma^{1,0}\pi_+^{1,0}$	−1.250399	−1.297942	−1.340581	−1.378342	−1.411268	−1.439415	−1.462850
$\sigma^{1,0}\pi_-^{1,0}$	−1.250399	−1.397942	−1.540581	−1.678342	−1.811268	−1.939415	−2.062850

Table S67: Total energies in E_h for the He atom in the cc-pVQZ basis set in fully uncontracted form, employing the real-orbital approximation.

	$0.00B_0$	$0.10B_0$	$0.20B_0$	$0.30B_0$	$0.40B_0$	$0.50B_0$	$0.60B_0$
$\sigma^{1,1}$	−2.861514	−2.859544	−2.853669	−2.843985	−2.830645	−2.813844	−2.793801
$\sigma^{2,0}$	−1.994059	−2.088025	−2.169953	−2.239939	−2.298147	−2.344814	−2.380261
$\sigma^{1,0}\pi_+^{1,0}$	−1.564499	−1.611388	−1.652069	−1.686575	−1.714964	−1.737309	−1.753703
$\sigma^{1,0}\pi_-^{1,0}$	−1.564499	−1.711388	−1.852069	−1.986575	−2.114964	−2.237309	−2.353703

Table S68: Total energies in E_h for the He atom in the cc-pV5Z basis set in fully uncontracted form, employing the real-orbital approximation.

	$0.00B_0$	$0.10B_0$	$0.20B_0$	$0.30B_0$	$0.40B_0$	$0.50B_0$	$0.60B_0$
$\sigma^{1,1}$	−2.861625	−2.859654	−2.853779	−2.844106	−2.830800	−2.814064	−2.794124
$\sigma^{2,0}$	−2.040788	−2.133973	−2.213589	−2.279823	−2.332992	−2.373565	−2.402179
$\sigma^{1,0}\pi_+^{1,0}$	−1.708534	−1.754871	−1.793898	−1.825668	−1.850262	−1.867795	−1.878409
$\sigma^{1,0}\pi_-^{1,0}$	−1.708534	−1.854871	−1.993898	−2.125668	−2.250262	−2.367795	−2.478409

Table S69: Total energies in E_h for the He atom in the aug-cc-pVDZ basis set in fully uncontracted form, employing the real-orbital approximation.

	$0.00B_0$	$0.10B_0$	$0.20B_0$	$0.30B_0$	$0.40B_0$	$0.50B_0$	$0.60B_0$
$\sigma^{1,1}$	−2.855730	−2.853740	−2.847806	−2.838027	−2.824545	−2.807524	−2.787134
$\sigma^{2,0}$	−2.150809	−2.239087	−2.304231	−2.347223	−2.369897	−2.375214	−2.367527
$\sigma^{1,0}\pi_+^{1,0}$	−1.954118	−1.998522	−2.031758	−2.053907	−2.065101	−2.065533	−2.055455
$\sigma^{1,0}\pi_-^{1,0}$	−1.954118	−2.098522	−2.231758	−2.353907	−2.465101	−2.565533	−2.655455

Table S70: Total energies in E_h for the He atom in the aug-cc-pVTZ basis set in fully uncontracted form, employing the real-orbital approximation.

	$0.00B_0$	$0.10B_0$	$0.20B_0$	$0.30B_0$	$0.40B_0$	$0.50B_0$	$0.60B_0$
$\sigma^{1,1}$	−2.861184	−2.859209	−2.853327	−2.843657	−2.830370	−2.813677	−2.793804
$\sigma^{2,0}$	−2.169704	−2.254785	−2.311541	−2.344784	−2.362764	−2.375088	−2.388171
$\sigma^{1,0}\pi_+^{1,0}$	−2.011899	−2.055113	−2.084840	−2.101335	−2.105036	−2.096589	−2.076869
$\sigma^{1,0}\pi_-^{1,0}$	−2.011899	−2.155113	−2.284840	−2.401335	−2.505036	−2.596589	−2.676869

Table S71: Total energies in E_h for the He atom in the aug-cc-pVQZ basis set in fully uncontracted form, employing the real-orbital approximation.

	$0.00B_0$	$0.10B_0$	$0.20B_0$	$0.30B_0$	$0.40B_0$	$0.50B_0$	$0.60B_0$
$\sigma^{1,1}$	−2.861522	−2.859550	−2.853677	−2.844028	−2.830785	−2.814166	−2.794403
$\sigma^{2,0}$	−2.171225	−2.255711	−2.311488	−2.345587	−2.368375	−2.388616	−2.409552
$\sigma^{1,0}\pi_+^{1,0}$	−2.050036	−2.091890	−2.117651	−2.127928	−2.123785	−2.106786	−2.079026
$\sigma^{1,0}\pi_-^{1,0}$	−2.050036	−2.191890	−2.317651	−2.427928	−2.523785	−2.606786	−2.679026

Table S72: Total energies in E_h for the He atom in the aug-cc-pV5Z basis set in fully uncontracted form, employing the real-orbital approximation.

	$0.00B_0$	$0.10B_0$	$0.20B_0$	$0.30B_0$	$0.40B_0$	$0.50B_0$	$0.60B_0$
$\sigma^{1,1}$	−2.861627	−2.859655	−2.853787	−2.844149	−2.830928	−2.814344	−2.794629
$\sigma^{2,0}$	−2.171824	−2.256030	−2.311747	−2.347780	−2.375100	−2.400640	−2.426090
$\sigma^{1,0}\pi_+^{1,0}$	−2.071382	−2.112081	−2.134536	−2.139855	−2.129958	−2.107614	−2.076295
$\sigma^{1,0}\pi_-^{1,0}$	−2.071382	−2.212081	−2.334536	−2.439855	−2.529958	−2.607614	−2.676295

Table S73: Total energies in E_h for the He atom in the HGBSP1-5 basis set in fully uncontracted form, employing the real-orbital approximation.

	$0.00B_0$	$0.10B_0$	$0.20B_0$	$0.30B_0$	$0.40B_0$	$0.50B_0$	$0.60B_0$
$\sigma^{1,1}$	−2.861636	−2.859665	−2.853788	−2.844114	−2.830802	−2.814040	−2.794029
$\sigma^{2,0}$	−2.131394	−2.256272	−2.312258	−2.350645	−2.405315	−2.455334	−2.501909
$\sigma^{1,0}\pi_+^{1,0}$	−2.131394	−2.158893	−2.160415	−2.149396	−2.130966	−2.107602	−2.079875
$\sigma^{1,0}\pi_-^{1,0}$	−2.131394	−2.258893	−2.360415	−2.449396	−2.530966	−2.607602	−2.679875

Table S74: Total energies in E_h for the He atom in the HGBSP1-7 basis set in fully uncontracted form, employing the real-orbital approximation.

	$0.00B_0$	$0.10B_0$	$0.20B_0$	$0.30B_0$	$0.40B_0$	$0.50B_0$	$0.60B_0$
$\sigma^{1,1}$	−2.861679	−2.859708	−2.853832	−2.844160	−2.830849	−2.814088	−2.794078
$\sigma^{2,0}$	−2.172447	−2.256151	−2.312809	−2.350686	−2.405573	−2.455864	−2.502406
$\sigma^{1,0}\pi_+^{1,0}$	−2.131437	−2.158980	−2.160452	−2.149732	−2.131506	−2.107794	−2.079840
$\sigma^{1,0}\pi_-^{1,0}$	−2.131437	−2.258980	−2.360452	−2.449732	−2.531506	−2.607794	−2.679840

Table S75: Total energies in E_h for the He atom in the HGBSP1-9 basis set in fully uncontracted form, employing the real-orbital approximation.

	$0.00B_0$	$0.10B_0$	$0.20B_0$	$0.30B_0$	$0.40B_0$	$0.50B_0$	$0.60B_0$
$\sigma^{1,1}$	−2.861680	−2.859709	−2.853833	−2.844160	−2.830849	−2.814089	−2.794079
$\sigma^{2,0}$	−2.173279	−2.256407	−2.312872	−2.350705	−2.405589	−2.455862	−2.502432
$\sigma^{1,0}\pi_+^{1,0}$	−2.131437	−2.158983	−2.160467	−2.149742	−2.131518	−2.107875	−2.079934
$\sigma^{1,0}\pi_-^{1,0}$	−2.131437	−2.258983	−2.360467	−2.449742	−2.531518	−2.607875	−2.679934

Table S76: Total energies in E_h for the He atom in the HGBSP2-5 basis set in fully uncontracted form, employing the real-orbital approximation.

	$0.00B_0$	$0.10B_0$	$0.20B_0$	$0.30B_0$	$0.40B_0$	$0.50B_0$	$0.60B_0$
$\sigma^{1,1}$	−2.861636	−2.859665	−2.853800	−2.844171	−2.830966	−2.814402	−2.794702
$\sigma^{2,0}$	−2.172556	−2.256744	−2.317044	−2.364971	−2.405536	−2.455705	−2.502485
$\sigma^{1,0}\pi_+^{1,0}$	−2.131398	−2.158900	−2.160425	−2.149405	−2.130971	−2.107604	−2.079882
$\sigma^{1,0}\pi_-^{1,0}$	−2.131398	−2.258900	−2.360425	−2.449405	−2.530971	−2.607604	−2.679882

Table S77: Total energies in E_h for the He atom in the HGBSP2-7 basis set in fully uncontracted form, employing the real-orbital approximation.

	$0.00B_0$	$0.10B_0$	$0.20B_0$	$0.30B_0$	$0.40B_0$	$0.50B_0$	$0.60B_0$
$\sigma^{1,1}$	−2.861679	−2.859709	−2.853844	−2.844216	−2.831013	−2.814449	−2.794751
$\sigma^{2,0}$	−2.172447	−2.256614	−2.317295	−2.365721	−2.405794	−2.456232	−2.502978
$\sigma^{1,0}\pi_+^{1,0}$	−2.131441	−2.158987	−2.160461	−2.149741	−2.131511	−2.107797	−2.079848
$\sigma^{1,0}\pi_-^{1,0}$	−2.131441	−2.258987	−2.360461	−2.449741	−2.531511	−2.607797	−2.679848

Table S78: Total energies in E_h for the He atom in the HGBSP2-9 basis set in fully uncontracted form, employing the real-orbital approximation.

	$0.00B_0$	$0.10B_0$	$0.20B_0$	$0.30B_0$	$0.40B_0$	$0.50B_0$	$0.60B_0$
$\sigma^{1,1}$	−2.861680	−2.859709	−2.853845	−2.844217	−2.831013	−2.814450	−2.794753
$\sigma^{2,0}$	−2.173279	−2.256908	−2.317306	−2.365802	−2.405811	−2.456231	−2.503005
$\sigma^{1,0}\pi_+^{1,0}$	−2.131442	−2.158990	−2.160477	−2.149751	−2.131523	−2.107878	−2.079942
$\sigma^{1,0}\pi_-^{1,0}$	−2.131442	−2.258990	−2.360477	−2.449751	−2.531523	−2.607878	−2.679942

Table S79: Total energies in E_h for the He atom in the HGBSP3-5 basis set in fully uncontracted form, employing the real-orbital approximation.

	$0.00B_0$	$0.10B_0$	$0.20B_0$	$0.30B_0$	$0.40B_0$	$0.50B_0$	$0.60B_0$
$\sigma^{1,1}$	−2.861636	−2.859665	−2.853800	−2.844171	−2.830966	−2.814402	−2.794702
$\sigma^{2,0}$	−2.172556	−2.256744	−2.317044	−2.358006	−2.418205	−2.474459	−2.527575
$\sigma^{1,0}\pi_+^{1,0}$	−2.131398	−2.159154	−2.161731	−2.152425	−2.135983	−2.114715	−2.089374
$\sigma^{1,0}\pi_-^{1,0}$	−2.131398	−2.259154	−2.361731	−2.452425	−2.535983	−2.614715	−2.689374

Table S80: Total energies in E_h for the He atom in the HGBSP3-7 basis set in fully uncontracted form, employing the real-orbital approximation.

	$0.00B_0$	$0.10B_0$	$0.20B_0$	$0.30B_0$	$0.40B_0$	$0.50B_0$	$0.60B_0$
$\sigma^{1,1}$	−2.861679	−2.859709	−2.853844	−2.844216	−2.831013	−2.814449	−2.794751
$\sigma^{2,0}$	−2.172447	−2.256614	−2.317295	−2.365721	−2.418289	−2.474652	−2.527880
$\sigma^{1,0}\pi_+^{1,0}$	−2.131441	−2.159236	−2.161775	−2.152677	−2.136423	−2.114960	−2.089430
$\sigma^{1,0}\pi_-^{1,0}$	−2.131441	−2.259236	−2.361775	−2.452677	−2.536423	−2.614960	−2.689430

Table S81: Total energies in E_h for the He atom in the HGBSP3-9 basis set in fully uncontracted form, employing the real-orbital approximation.

	$0.00B_0$	$0.10B_0$	$0.20B_0$	$0.30B_0$	$0.40B_0$	$0.50B_0$	$0.60B_0$
$\sigma^{1,1}$	−2.861680	−2.859709	−2.853845	−2.844217	−2.831013	−2.814450	−2.794753
$\sigma^{2,0}$	−2.173279	−2.256908	−2.317306	−2.365802	−2.418294	−2.474661	−2.527892
$\sigma^{1,0}\pi_+^{1,0}$	−2.131442	−2.159239	−2.161787	−2.152686	−2.136433	−2.115011	−2.089491
$\sigma^{1,0}\pi_-^{1,0}$	−2.131442	−2.259239	−2.361787	−2.452686	−2.536433	−2.615011	−2.689491

Table S82: Total energies in E_h for the He atom in the AHGBSP1-5 basis set in fully uncontracted form, employing the real-orbital approximation.

	$0.00B_0$	$0.10B_0$	$0.20B_0$	$0.30B_0$	$0.40B_0$	$0.50B_0$	$0.60B_0$
$\sigma^{1,1}$	−2.861636	−2.859665	−2.853789	−2.844115	−2.830803	−2.814041	−2.794030
$\sigma^{2,0}$	−2.174197	−2.256523	−2.312542	−2.350646	−2.405328	−2.455361	−2.501934
$\sigma^{1,0}\pi_+^{1,0}$	−2.131394	−2.158903	−2.160417	−2.149414	−2.130993	−2.107613	−2.079875
$\sigma^{1,0}\pi_-^{1,0}$	−2.131394	−2.258903	−2.360417	−2.449414	−2.530993	−2.607613	−2.679875

Table S83: Total energies in E_h for the He atom in the AHGBSP1-7 basis set in fully uncontracted form, employing the real-orbital approximation.

	$0.00B_0$	$0.10B_0$	$0.20B_0$	$0.30B_0$	$0.40B_0$	$0.50B_0$	$0.60B_0$
$\sigma^{1,1}$	−2.861679	−2.859708	−2.853832	−2.844160	−2.830849	−2.814088	−2.794078
$\sigma^{2,0}$	−2.174250	−2.256578	−2.312872	−2.350690	−2.405576	−2.455864	−2.502408
$\sigma^{1,0}\pi_+^{1,0}$	−2.131437	−2.158981	−2.160455	−2.149734	−2.131507	−2.107801	−2.079848
$\sigma^{1,0}\pi_-^{1,0}$	−2.131437	−2.258981	−2.360455	−2.449734	−2.531507	−2.607801	−2.679848

Table S84: Total energies in E_h for the He atom in the AHGBSP1-9 basis set in fully uncontracted form, employing the real-orbital approximation.

	$0.00B_0$	$0.10B_0$	$0.20B_0$	$0.30B_0$	$0.40B_0$	$0.50B_0$	$0.60B_0$
$\sigma^{1,1}$	−2.861680	−2.859709	−2.853833	−2.844160	−2.830849	−2.814089	−2.794079
$\sigma^{2,0}$	−2.174251	−2.256580	−2.312892	−2.350705	−2.405590	−2.455863	−2.502433
$\sigma^{1,0}\pi_+^{1,0}$	−2.131437	−2.158983	−2.160467	−2.149743	−2.131518	−2.107875	−2.079934
$\sigma^{1,0}\pi_-^{1,0}$	−2.131437	−2.258983	−2.360467	−2.449743	−2.531518	−2.607875	−2.679934

Table S85: Total energies in E_h for the He atom in the AHGBSP2-5 basis set in fully uncontracted form, employing the real-orbital approximation.

	$0.00B_0$	$0.10B_0$	$0.20B_0$	$0.30B_0$	$0.40B_0$	$0.50B_0$	$0.60B_0$
$\sigma^{1,1}$	−2.861636	−2.859666	−2.853801	−2.844172	−2.830967	−2.814402	−2.794702
$\sigma^{2,0}$	−2.174197	−2.257079	−2.317086	−2.365282	−2.405550	−2.455732	−2.502510
$\sigma^{1,0}\pi_+^{1,0}$	−2.131399	−2.158911	−2.160426	−2.149423	−2.130998	−2.107616	−2.079883
$\sigma^{1,0}\pi_-^{1,0}$	−2.131399	−2.258911	−2.360426	−2.449423	−2.530998	−2.607616	−2.679883

Table S86: Total energies in E_h for the He atom in the AHGBSP2-7 basis set in fully uncontracted form, employing the real-orbital approximation.

	$0.00B_0$	$0.10B_0$	$0.20B_0$	$0.30B_0$	$0.40B_0$	$0.50B_0$	$0.60B_0$
$\sigma^{1,1}$	−2.861679	−2.859709	−2.853844	−2.844216	−2.831013	−2.814449	−2.794752
$\sigma^{2,0}$	−2.174250	−2.257134	−2.317299	−2.365771	−2.405798	−2.456232	−2.502980
$\sigma^{1,0}\pi_+^{1,0}$	−2.131441	−2.158989	−2.160464	−2.149743	−2.131512	−2.107804	−2.079856
$\sigma^{1,0}\pi_-^{1,0}$	−2.131441	−2.258989	−2.360464	−2.449743	−2.531512	−2.607804	−2.679856

Table S87: Total energies in E_h for the He atom in the AHGBSP2-9 basis set in fully uncontracted form, employing the real-orbital approximation.

	$0.00B_0$	$0.10B_0$	$0.20B_0$	$0.30B_0$	$0.40B_0$	$0.50B_0$	$0.60B_0$
$\sigma^{1,1}$	−2.861680	−2.859709	−2.853845	−2.844217	−2.831013	−2.814450	−2.794753
$\sigma^{2,0}$	−2.174251	−2.257136	−2.317310	−2.365805	−2.405811	−2.456231	−2.503005
$\sigma^{1,0}\pi_+^{1,0}$	−2.131442	−2.158990	−2.160477	−2.149751	−2.131523	−2.107878	−2.079943
$\sigma^{1,0}\pi_-^{1,0}$	−2.131442	−2.258990	−2.360477	−2.449751	−2.531523	−2.607878	−2.679943

Table S88: Total energies in E_h for the He atom in the AHGBSP3-5 basis set in fully uncontracted form, employing the real-orbital approximation.

	$0.00B_0$	$0.10B_0$	$0.20B_0$	$0.30B_0$	$0.40B_0$	$0.50B_0$	$0.60B_0$
$\sigma^{1,1}$	−2.861636	−2.859666	−2.853801	−2.844172	−2.830967	−2.814402	−2.794702
$\sigma^{2,0}$	−2.174197	−2.257079	−2.317086	−2.365282	−2.418206	−2.474463	−2.527583
$\sigma^{1,0}\pi_+^{1,0}$	−2.131399	−2.159163	−2.161733	−2.152436	−2.136003	−2.114726	−2.089376
$\sigma^{1,0}\pi_-^{1,0}$	−2.131399	−2.259163	−2.361733	−2.452436	−2.536003	−2.614726	−2.689376

Table S89: Total energies in E_h for the He atom in the AHGBSP3-7 basis set in fully uncontracted form, employing the real-orbital approximation.

	$0.00B_0$	$0.10B_0$	$0.20B_0$	$0.30B_0$	$0.40B_0$	$0.50B_0$	$0.60B_0$
$\sigma^{1,1}$	−2.861679	−2.859709	−2.853844	−2.844216	−2.831013	−2.814449	−2.794752
$\sigma^{2,0}$	−2.174250	−2.257134	−2.317299	−2.365771	−2.418290	−2.474653	−2.527882
$\sigma^{1,0}\pi_+^{1,0}$	−2.131441	−2.159237	−2.161777	−2.152679	−2.136424	−2.114963	−2.089434
$\sigma^{1,0}\pi_-^{1,0}$	−2.131441	−2.259237	−2.361777	−2.452679	−2.536424	−2.614963	−2.689434

Table S90: Total energies in E_h for the He atom in the AHGBSP3-9 basis set in fully uncontracted form, employing the real-orbital approximation.

	$0.00B_0$	$0.10B_0$	$0.20B_0$	$0.30B_0$	$0.40B_0$	$0.50B_0$	$0.60B_0$
$\sigma^{1,1}$	−2.861680	−2.859709	−2.853845	−2.844217	−2.831013	−2.814450	−2.794753
$\sigma^{2,0}$	−2.174251	−2.257136	−2.317310	−2.365805	−2.418294	−2.474661	−2.527892
$\sigma^{1,0}\pi_+^{1,0}$	−2.131442	−2.159239	−2.161787	−2.152686	−2.136433	−2.115011	−2.089491
$\sigma^{1,0}\pi_-^{1,0}$	−2.131442	−2.259239	−2.361787	−2.452686	−2.536433	−2.615011	−2.689491

Table S91: Total energies in E_h for the He atom in the 6-311++G(3df,3pd) basis set in fully uncontracted form, employing the real-orbital approximation.

	$0.00B_0$	$0.10B_0$	$0.20B_0$	$0.30B_0$	$0.40B_0$	$0.50B_0$	$0.60B_0$
$\sigma^{1,1}$	−2.859985	−2.858008	−2.852114	−2.842406	−2.829035	−2.812182	−2.792041
$\sigma^{2,0}$	−2.146041	−2.235112	−2.302750	−2.350281	−2.380064	−2.395582	−2.401240
$\sigma^{1,0}\pi_+^{1,0}$	−2.023329	−2.066211	−2.094949	−2.109826	−2.111331	−2.100191	−2.077402
$\sigma^{1,0}\pi_-^{1,0}$	−2.023329	−2.166211	−2.294949	−2.409826	−2.511331	−2.600191	−2.677402

Table S92: Total energies in E_h for the He atom in the def2-TZVP basis set in fully uncontracted form, employing the real-orbital approximation.

	$0.00B_0$	$0.10B_0$	$0.20B_0$	$0.30B_0$	$0.40B_0$	$0.50B_0$	$0.60B_0$
$\sigma^{1,1}$	−2.859895	−2.857937	−2.852083	−2.842403	−2.829007	−2.812043	−2.791689
$\sigma^{2,0}$	−1.796441	−1.892098	−1.979077	−2.057391	−2.127067	−2.188141	−2.240662
$\sigma^{1,0}\pi_+^{1,0}$	−0.855649	−0.903602	−0.947468	−0.987272	−1.023052	−1.054862	−1.082761
$\sigma^{1,0}\pi_-^{1,0}$	−0.855649	−1.003602	−1.147468	−1.287272	−1.423052	−1.554862	−1.682761

Table S93: Total energies in E_h for the Li atom in the cc-pVDZ basis set in fully uncontracted form, employing the real-orbital approximation.

	$0.00B_0$	$0.10B_0$	$0.20B_0$	$0.30B_0$	$0.40B_0$	$0.50B_0$	$0.60B_0$
$\sigma^{2,1}$	−7.432444	−7.467911	−7.481965	−7.483823	−7.476811	−7.462909	−7.443458
$\sigma^{1,1}\pi_-^{1,0}$	−7.364596	−7.440559	−7.489864	−7.523437	−7.546651	−7.566623	−7.586517
$\sigma^{1,1}\pi_+^{1,0}$	−7.364596	−7.340559	−7.289864	−7.223437	−7.146651	−7.066623	−6.986517
$\sigma^{1,1}\delta_-^{1,0}$	−7.102531	−7.236655	−7.339029	−7.409660	−7.448556	−7.455731	−7.431200

Table S94: Total energies in E_h for the Li atom in the cc-pVTZ basis set in fully uncontracted form, employing the real-orbital approximation.

	$0.00B_0$	$0.10B_0$	$0.20B_0$	$0.30B_0$	$0.40B_0$	$0.50B_0$	$0.60B_0$
$\sigma^{2,1}$	−7.432703	−7.468279	−7.483278	−7.486882	−7.481420	−7.467911	−7.447665
$\sigma^{1,1}\pi_-^{1,0}$	−7.365000	−7.441319	−7.490338	−7.526307	−7.557208	−7.584515	−7.606909
$\sigma^{1,1}\pi_+^{1,0}$	−7.365000	−7.341319	−7.290338	−7.226307	−7.157208	−7.084515	−7.006909
$\sigma^{1,1}\delta_-^{1,0}$	−7.201630	−7.325695	−7.400480	−7.435386	−7.448856	−7.459496	−7.470237

Table S95: Total energies in E_h for the Li atom in the cc-pVQZ basis set in fully uncontracted form, employing the real-orbital approximation.

	$0.00B_0$	$0.10B_0$	$0.20B_0$	$0.30B_0$	$0.40B_0$	$0.50B_0$	$0.60B_0$
$\sigma^{2,1}$	−7.432719	−7.468443	−7.483714	−7.487355	−7.483190	−7.473189	−7.458963
$\sigma^{1,1}\pi_-^{1,0}$	−7.365038	−7.441468	−7.490971	−7.529318	−7.560925	−7.586099	−7.605855
$\sigma^{1,1}\pi_+^{1,0}$	−7.365038	−7.341468	−7.290971	−7.229318	−7.160925	−7.086099	−7.005855
$\sigma^{1,1}\delta_-^{1,0}$	−7.227207	−7.346060	−7.408546	−7.434734	−7.451556	−7.468624	−7.482573

Table S96: Total energies in E_h for the Li atom in the cc-pV5Z basis set in fully uncontracted form, employing the real-orbital approximation.

	$0.00B_0$	$0.10B_0$	$0.20B_0$	$0.30B_0$	$0.40B_0$	$0.50B_0$	$0.60B_0$
$\sigma^{2,1}$	−7.432747	−7.468499	−7.483833	−7.487508	−7.483673	−7.474739	−7.462426
$\sigma^{1,1}\pi_-^{1,0}$	−7.365067	−7.441523	−7.491406	−7.530099	−7.561253	−7.586549	−7.607764
$\sigma^{1,1}\pi_+^{1,0}$	−7.365067	−7.341523	−7.291406	−7.230099	−7.161253	−7.086549	−7.007764
$\sigma^{1,1}\delta_-^{1,0}$	−7.239537	−7.354899	−7.410670	−7.436260	−7.457927	−7.477360	−7.491327

Table S97: Total energies in E_h for the Li atom in the aug-cc-pVDZ basis set in fully uncontracted form, employing the real-orbital approximation.

	$0.00B_0$	$0.10B_0$	$0.20B_0$	$0.30B_0$	$0.40B_0$	$0.50B_0$	$0.60B_0$
$\sigma^{2,1}$	−7.432449	−7.468123	−7.483321	−7.486707	−7.480763	−7.466892	−7.446647
$\sigma^{1,1}\pi_-^{1,0}$	−7.364618	−7.440652	−7.489870	−7.523549	−7.546960	−7.566992	−7.586809
$\sigma^{1,1}\pi_+^{1,0}$	−7.364618	−7.340652	−7.289870	−7.223549	−7.146960	−7.066992	−6.986809
$\sigma^{1,1}\delta_-^{1,0}$	−7.221318	−7.340775	−7.405248	−7.434550	−7.451078	−7.457809	−7.447630

Table S98: Total energies in E_h for the Li atom in the aug-cc-pVTZ basis set in fully uncontracted form, employing the real-orbital approximation.

	$0.00B_0$	$0.10B_0$	$0.20B_0$	$0.30B_0$	$0.40B_0$	$0.50B_0$	$0.60B_0$
$\sigma^{2,1}$	−7.432706	−7.468462	−7.483814	−7.487101	−7.481526	−7.468712	−7.449847
$\sigma^{1,1}\pi_-^{1,0}$	−7.365002	−7.441483	−7.491444	−7.528915	−7.560012	−7.586439	−7.607825
$\sigma^{1,1}\pi_+^{1,0}$	−7.365002	−7.341483	−7.291444	−7.228915	−7.160012	−7.086439	−7.007825
$\sigma^{1,1}\delta_-^{1,0}$	−7.272382	−7.371457	−7.409366	−7.435386	−7.450834	−7.461776	−7.470984

Table S99: Total energies in E_h for the Li atom in the aug-cc-pVQZ basis set in fully uncontracted form, employing the real-orbital approximation.

	$0.00B_0$	$0.10B_0$	$0.20B_0$	$0.30B_0$	$0.40B_0$	$0.50B_0$	$0.60B_0$
$\sigma^{2,1}$	−7.432719	−7.468525	−7.483884	−7.487667	−7.484341	−7.476027	−7.463986
$\sigma^{1,1}\pi_-^{1,0}$	−7.365039	−7.441640	−7.491847	−7.530216	−7.561297	−7.586181	−7.605991
$\sigma^{1,1}\pi_+^{1,0}$	−7.365039	−7.341640	−7.291847	−7.230216	−7.161297	−7.086181	−7.005991
$\sigma^{1,1}\delta_-^{1,0}$	−7.283070	−7.371879	−7.411479	−7.440515	−7.461732	−7.479106	−7.491519

Table S100: Total energies in E_h for the Li atom in the aug-cc-pV5Z basis set in fully uncontracted form, employing the real-orbital approximation.

$\sigma^{2,1}$
$\sigma^{1,1}\pi_-^{1,0}$
$\sigma^{1,1}\pi_+^{1,0}$
$\sigma^{1,1}\delta_-^{1,0}$

Table S101: Total energies in E_h for the Li atom in the HGBSP1-5 basis set in fully uncontracted form, employing the real-orbital approximation.

	$0.00B_0$	$0.10B_0$	$0.20B_0$	$0.30B_0$	$0.40B_0$	$0.50B_0$	$0.60B_0$
$\sigma^{2,1}$	−7.432493	−7.468390	−7.483461	−7.486981	−7.482845	−7.472844	−7.458234
$\sigma^{1,1}\pi_-^{1,0}$	−7.364955	−7.441297	−7.490543	−7.526772	−7.555656	−7.579310	−7.598202
$\sigma^{1,1}\pi_+^{1,0}$	−7.364955	−7.341297	−7.290543	−7.226772	−7.155656	−7.079310	−6.998202
$\sigma^{1,1}\delta_-^{1,0}$	−7.291852	−7.372009	−7.410387	−7.434378	−7.452015	−7.465113	−7.472263

Table S102: Total energies in E_h for the Li atom in the HGBSP1-7 basis set in fully uncontracted form, employing the real-orbital approximation.

	$0.00B_0$	$0.10B_0$	$0.20B_0$	$0.30B_0$	$0.40B_0$	$0.50B_0$	$0.60B_0$
$\sigma^{2,1}$	−7.431942	−7.468363	−7.483899	−7.487337	−7.483093	−7.473192	−7.458790
$\sigma^{1,1}\pi_-^{1,0}$	−7.365079	−7.441459	−7.490690	−7.527272	−7.556165	−7.579426	−7.598324
$\sigma^{1,1}\pi_+^{1,0}$	−7.365079	−7.341459	−7.290690	−7.227272	−7.156165	−7.079426	−6.998324
$\sigma^{1,1}\delta_-^{1,0}$	−7.291975	−7.372260	−7.410444	−7.435602	−7.452986	−7.464939	−7.473074

Table S103: Total energies in E_h for the Li atom in the HGBSP1-9 basis set in fully uncontracted form, employing the real-orbital approximation.

	$0.00B_0$	$0.10B_0$	$0.20B_0$	$0.30B_0$	$0.40B_0$	$0.50B_0$	$0.60B_0$
$\sigma^{2,1}$	−7.432744	−7.468558	−7.483914	−7.487398	−7.483132	−7.473212	−7.458834
$\sigma^{1,1}\pi_-^{1,0}$	−7.365081	−7.441465	−7.490702	−7.527302	−7.556163	−7.579491	−7.598457
$\sigma^{1,1}\pi_+^{1,0}$	−7.365081	−7.341465	−7.290702	−7.227302	−7.156163	−7.079491	−6.998457
$\sigma^{1,1}\delta_-^{1,0}$	−7.291977	−7.372289	−7.410519	−7.435610	−7.453128	−7.465239	−7.473182

Table S104: Total energies in E_h for the Li atom in the HGBSP2-5 basis set in fully uncontracted form, employing the real-orbital approximation.

	$0.00B_0$	$0.10B_0$	$0.20B_0$	$0.30B_0$	$0.40B_0$	$0.50B_0$	$0.60B_0$
$\sigma^{2,1}$	−7.432493	−7.468390	−7.483461	−7.486981	−7.482845	−7.472844	−7.458234
$\sigma^{1,1}\pi_-^{1,0}$	−7.364955	−7.441577	−7.491989	−7.530097	−7.561076	−7.587005	−7.608576
$\sigma^{1,1}\pi_+^{1,0}$	−7.364955	−7.341577	−7.291989	−7.230097	−7.161076	−7.087005	−7.008576
$\sigma^{1,1}\delta_-^{1,0}$	−7.267540	−7.372009	−7.410387	−7.434378	−7.452015	−7.465113	−7.472263

Table S105: Total energies in E_h for the Li atom in the HGBSP2-7 basis set in fully uncontracted form, employing the real-orbital approximation.

	$0.00B_0$	$0.10B_0$	$0.20B_0$	$0.30B_0$	$0.40B_0$	$0.50B_0$	$0.60B_0$
$\sigma^{2,1}$	−7.431942	−7.468363	−7.483899	−7.487337	−7.483093	−7.473192	−7.458790
$\sigma^{1,1}\pi_-^{1,0}$	−7.365079	−7.441736	−7.492136	−7.530498	−7.561530	−7.587210	−7.608700
$\sigma^{1,1}\pi_+^{1,0}$	−7.365079	−7.341736	−7.292136	−7.230498	−7.161530	−7.087210	−7.008700
$\sigma^{1,1}\delta_-^{1,0}$	−7.291975	−7.372260	−7.410444	−7.435602	−7.452986	−7.464939	−7.473074

Table S106: Total energies in E_h for the Li atom in the HGBSP2-9 basis set in fully uncontracted form, employing the real-orbital approximation.

	$0.00B_0$	$0.10B_0$	$0.20B_0$	$0.30B_0$	$0.40B_0$	$0.50B_0$	$0.60B_0$
$\sigma^{2,1}$	−7.432744	−7.468558	−7.483914	−7.487398	−7.483132	−7.473212	−7.458834
$\sigma^{1,1}\pi_-^{1,0}$	−7.365081	−7.441741	−7.492145	−7.530521	−7.561532	−7.587251	−7.608784
$\sigma^{1,1}\pi_+^{1,0}$	−7.365081	−7.341741	−7.292145	−7.230521	−7.161532	−7.087251	−7.008784
$\sigma^{1,1}\delta_-^{1,0}$	−7.291977	−7.372289	−7.410519	−7.435610	−7.453128	−7.465239	−7.473182

Table S107: Total energies in E_h for the Li atom in the HGBSP3-5 basis set in fully uncontracted form, employing the real-orbital approximation.

	$0.00B_0$	$0.10B_0$	$0.20B_0$	$0.30B_0$	$0.40B_0$	$0.50B_0$	$0.60B_0$
$\sigma^{2,1}$	−7.432493	−7.468392	−7.483564	−7.487552	−7.484514	−7.476505	−7.464968
$\sigma^{1,1}\pi_-^{1,0}$	−7.364955	−7.441577	−7.491989	−7.530097	−7.561076	−7.587005	−7.608576
$\sigma^{1,1}\pi_+^{1,0}$	−7.364955	−7.341577	−7.291989	−7.230097	−7.161076	−7.087005	−7.008576
$\sigma^{1,1}\delta_-^{1,0}$	−7.291852	−7.373237	−7.414334	−7.442103	−7.463219	−7.479993	−7.491942

Table S108: Total energies in E_h for the Li atom in the HGBSP3-7 basis set in fully uncontracted form, employing the real-orbital approximation.

	$0.00B_0$	$0.10B_0$	$0.20B_0$	$0.30B_0$	$0.40B_0$	$0.50B_0$	$0.60B_0$
$\sigma^{2,1}$	−7.431942	−7.468365	−7.483980	−7.487867	−7.484720	−7.476818	−7.465497
$\sigma^{1,1}\pi_-^{1,0}$	−7.365079	−7.441736	−7.492136	−7.530499	−7.561531	−7.587210	−7.608700
$\sigma^{1,1}\pi_+^{1,0}$	−7.365079	−7.341736	−7.292136	−7.230499	−7.161531	−7.087210	−7.008700
$\sigma^{1,1}\delta_-^{1,0}$	−7.291975	−7.373469	−7.414462	−7.442998	−7.464241	−7.480191	−7.492348

Table S109: Total energies in E_h for the Li atom in the HGBSP3-9 basis set in fully uncontracted form, employing the real-orbital approximation.

	$0.00B_0$	$0.10B_0$	$0.20B_0$	$0.30B_0$	$0.40B_0$	$0.50B_0$	$0.60B_0$
$\sigma^{2,1}$	−7.432744	−7.468560	−7.483995	−7.487901	−7.484729	−7.476825	−7.465527
$\sigma^{1,1}\pi_-^{1,0}$	−7.365081	−7.441741	−7.492145	−7.530521	−7.561532	−7.587251	−7.608785
$\sigma^{1,1}\pi_+^{1,0}$	−7.365081	−7.341741	−7.292145	−7.230521	−7.161532	−7.087251	−7.008785
$\sigma^{1,1}\delta_-^{1,0}$	−7.291977	−7.373491	−7.414510	−7.443028	−7.464308	−7.480426	−7.492510

Table S110: Total energies in E_h for the Li atom in the AHGBSP1-5 basis set in fully uncontracted form, employing the real-orbital approximation.

	$0.00B_0$	$0.10B_0$	$0.20B_0$	$0.30B_0$	$0.40B_0$	$0.50B_0$	$0.60B_0$
$\sigma^{2,1}$	−7.432618	−7.468395	−7.483609	−7.487121	−7.482905	−7.472854	−7.458245
$\sigma^{1,1}\pi_-^{1,0}$	−7.364955	−7.441306	−7.490544	−7.526795	−7.555676	−7.579313	−7.598203
$\sigma^{1,1}\pi_+^{1,0}$	−7.364955	−7.341306	−7.290544	−7.226795	−7.155676	−7.079313	−6.998203
$\sigma^{1,1}\delta_-^{1,0}$	−7.291853	−7.372020	−7.410387	−7.434402	−7.452032	−7.465113	−7.472269

Table S111: Total energies in E_h for the Li atom in the AHGBSP1-7 basis set in fully uncontracted form, employing the real-orbital approximation.

	$0.00B_0$	$0.10B_0$	$0.20B_0$	$0.30B_0$	$0.40B_0$	$0.50B_0$	$0.60B_0$
$\sigma^{2,1}$	−7.432749	−7.468556	−7.483904	−7.487379	−7.483124	−7.473203	−7.458791
$\sigma^{1,1}\pi_-^{1,0}$	−7.365079	−7.441462	−7.490692	−7.527277	−7.556165	−7.579432	−7.598335
$\sigma^{1,1}\pi_+^{1,0}$	−7.365079	−7.341462	−7.290692	−7.227277	−7.156165	−7.079432	−6.998335
$\sigma^{1,1}\delta_-^{1,0}$	−7.291975	−7.372262	−7.410446	−7.435602	−7.452988	−7.464943	−7.473075

Table S112: Total energies in E_h for the Li atom in the AHGBSP1-9 basis set in fully uncontracted form, employing the real-orbital approximation.

	$0.00B_0$	$0.10B_0$	$0.20B_0$	$0.30B_0$	$0.40B_0$	$0.50B_0$	$0.60B_0$
$\sigma^{2,1}$	−7.432751	−7.468558	−7.483914	−7.487399	−7.483133	−7.473212	−7.458834
$\sigma^{1,1}\pi_-^{1,0}$	−7.365081	−7.441465	−7.490702	−7.527302	−7.556164	−7.579491	−7.598457
$\sigma^{1,1}\pi_+^{1,0}$	−7.365081	−7.341465	−7.290702	−7.227302	−7.156164	−7.079491	−6.998457
$\sigma^{1,1}\delta_-^{1,0}$	−7.291977	−7.372289	−7.410519	−7.435611	−7.453128	−7.465240	−7.473183

Table S113: Total energies in E_h for the Li atom in the AHGBSP2-5 basis set in fully uncontracted form, employing the real-orbital approximation.

	$0.00B_0$	$0.10B_0$	$0.20B_0$	$0.30B_0$	$0.40B_0$	$0.50B_0$	$0.60B_0$
$\sigma^{2,1}$	−7.432618	−7.468395	−7.483609	−7.487121	−7.482905	−7.472854	−7.458245
$\sigma^{1,1}\pi_-^{1,0}$	−7.364955	−7.441585	−7.491989	−7.530113	−7.561093	−7.587009	−7.608576
$\sigma^{1,1}\pi_+^{1,0}$	−7.364955	−7.341585	−7.291989	−7.230113	−7.161093	−7.087009	−7.008576
$\sigma^{1,1}\delta_-^{1,0}$	−7.291853	−7.372020	−7.410387	−7.434402	−7.452032	−7.465113	−7.472269

Table S114: Total energies in E_h for the Li atom in the AHGBSP2-7 basis set in fully uncontracted form, employing the real-orbital approximation.

	$0.00B_0$	$0.10B_0$	$0.20B_0$	$0.30B_0$	$0.40B_0$	$0.50B_0$	$0.60B_0$
$\sigma^{2,1}$	−7.432749	−7.468556	−7.483904	−7.487379	−7.483124	−7.473203	−7.458791
$\sigma^{1,1}\pi_-^{1,0}$	−7.365079	−7.441738	−7.492137	−7.530502	−7.561531	−7.587212	−7.608705
$\sigma^{1,1}\pi_+^{1,0}$	−7.365079	−7.341738	−7.292137	−7.230502	−7.161531	−7.087212	−7.008705
$\sigma^{1,1}\delta_-^{1,0}$	−7.291975	−7.372262	−7.410446	−7.435602	−7.452988	−7.464943	−7.473075

Table S115: Total energies in E_h for the Li atom in the AHGBSP2-9 basis set in fully uncontracted form, employing the real-orbital approximation.

	$0.00B_0$	$0.10B_0$	$0.20B_0$	$0.30B_0$	$0.40B_0$	$0.50B_0$	$0.60B_0$
$\sigma^{2,1}$	−7.432751	−7.468558	−7.483914	−7.487399	−7.483133	−7.473212	−7.458834
$\sigma^{1,1}\pi_-^{1,0}$	−7.365081	−7.441741	−7.492145	−7.530521	−7.561532	−7.587251	−7.608784
$\sigma^{1,1}\pi_+^{1,0}$	−7.365081	−7.341741	−7.292145	−7.230521	−7.161532	−7.087251	−7.008784
$\sigma^{1,1}\delta_-^{1,0}$	−7.291977	−7.372289	−7.410519	−7.435611	−7.453128	−7.465240	−7.473183

Table S116: Total energies in E_h for the Li atom in the AHGBSP3-5 basis set in fully uncontracted form, employing the real-orbital approximation.

	$0.00B_0$	$0.10B_0$	$0.20B_0$	$0.30B_0$	$0.40B_0$	$0.50B_0$	$0.60B_0$
$\sigma^{2,1}$	−7.432618	−7.468397	−7.483694	−7.487648	−7.484546	−7.476513	−7.464972
$\sigma^{1,1}\pi_-^{1,0}$	−7.364955	−7.441585	−7.491989	−7.530113	−7.561093	−7.587010	−7.608576
$\sigma^{1,1}\pi_+^{1,0}$	−7.364955	−7.341585	−7.291989	−7.230113	−7.161093	−7.087010	−7.008576
$\sigma^{1,1}\delta_-^{1,0}$	−7.291853	−7.373244	−7.414336	−7.442118	−7.463237	−7.479996	−7.491943

Table S117: Total energies in E_h for the Li atom in the AHGBSP3-7 basis set in fully uncontracted form, employing the real-orbital approximation.

	$0.00B_0$	$0.10B_0$	$0.20B_0$	$0.30B_0$	$0.40B_0$	$0.50B_0$	$0.60B_0$
$\sigma^{2,1}$	−7.432749	−7.468558	−7.483984	−7.487884	−7.484724	−7.476818	−7.465497
$\sigma^{1,1}\pi_-^{1,0}$	−7.365079	−7.441738	−7.492137	−7.530502	−7.561531	−7.587212	−7.608705
$\sigma^{1,1}\pi_+^{1,0}$	−7.365079	−7.341738	−7.292137	−7.230502	−7.161531	−7.087212	−7.008705
$\sigma^{1,1}\delta_-^{1,0}$	−7.291975	−7.373470	−7.414464	−7.442999	−7.464242	−7.480193	−7.492349

Table S118: Total energies in E_h for the Li atom in the AHGBSP3-9 basis set in fully uncontracted form, employing the real-orbital approximation.

	$0.00B_0$	$0.10B_0$	$0.20B_0$	$0.30B_0$	$0.40B_0$	$0.50B_0$	$0.60B_0$
$\sigma^{2,1}$	−7.432751	−7.468560	−7.483995	−7.487902	−7.484729	−7.476826	−7.465528
$\sigma^{1,1}\pi_-^{1,0}$	−7.365081	−7.441741	−7.492145	−7.530521	−7.561533	−7.587251	−7.608785
$\sigma^{1,1}\pi_+^{1,0}$	−7.365081	−7.341741	−7.292145	−7.230521	−7.161533	−7.087251	−7.008785
$\sigma^{1,1}\delta_-^{1,0}$	−7.291977	−7.373491	−7.414510	−7.443028	−7.464308	−7.480427	−7.492511

Table S119: Total energies in E_h for the Li atom in the 6-311++G(3df,3pd) basis set in fully uncontracted form, employing the real-orbital approximation.

	$0.00B_0$	$0.10B_0$	$0.20B_0$	$0.30B_0$	$0.40B_0$	$0.50B_0$	$0.60B_0$
$\sigma^{2,1}$	−7.432168	−7.467926	−7.483298	−7.486062	−7.479529	−7.466616	−7.449902
$\sigma^{1,1}\pi_-^{1,0}$	−7.364332	−7.440672	−7.489360	−7.523231	−7.550238	−7.575823	−7.600227
$\sigma^{1,1}\pi_+^{1,0}$	−7.364332	−7.340672	−7.289360	−7.223231	−7.150238	−7.075823	−7.000227
$\sigma^{1,1}\delta_-^{1,0}$	−7.251311	−7.363522	−7.405503	−7.402430	−7.406934	−7.433303	−7.459671

Table S120: Total energies in E_h for the Li atom in the def2-TZVP basis set in fully uncontracted form, employing the real-orbital approximation.

	$0.00B_0$	$0.10B_0$	$0.20B_0$	$0.30B_0$	$0.40B_0$	$0.50B_0$	$0.60B_0$
$\sigma^{2,1}$	−7.432693	−7.468048	−7.480931	−7.479010	−7.463458	−7.434282	−7.392471
$\sigma^{1,1}\pi_-^{1,0}$	−7.357343	−7.437778	−7.480815	−7.492889	−7.488426	−7.488325	−7.503526
$\sigma^{1,1}\pi_+^{1,0}$	−7.357343	−7.337778	−7.280815	−7.192889	−7.088426	−6.988325	−6.903526
$\sigma^{1,1}\delta_-^{1,0}$							

Table S121: Total energies in E_h for the Be atom in the cc-pVDZ basis set in fully uncontracted form, employing the real-orbital approximation.

	$0.00B_0$	$0.10B_0$	$0.20B_0$	$0.30B_0$	$0.40B_0$	$0.50B_0$	$0.60B_0$
$\sigma^{2,2}$	−14.572338	−14.558169	−14.517776	−14.456525	−14.380634	−14.294827	−14.201592
$\sigma^{2,1}\pi_-^{1,0}$	−14.510712	−14.644004	−14.747701	−14.829952	−14.897428	−14.953423	−14.999334
$\sigma^{2,1}\pi_+^{1,0}$	−14.510712	−14.544004	−14.547701	−14.529952	−14.497428	−14.453423	−14.399334
$\sigma^{3,1}$	−14.512007	−14.600567	−14.667995	−14.718599	−14.757083	−14.786752	−14.809192
$\sigma^{2,1}\delta_-^{1,0}$	−13.955146	−14.140475	−14.297537	−14.428911	−14.537296	−14.624547	−14.691619
$\sigma^{1,1}\pi_-^{1,0}\delta_-^{1,0}$	−13.830398	−14.062359	−14.261374	−14.433451	−14.582378	−14.709246	−14.814337

Table S122: Total energies in E_h for the Be atom in the cc-pVTZ basis set in fully uncontracted form, employing the real-orbital approximation.

	$0.00B_0$	$0.10B_0$	$0.20B_0$	$0.30B_0$	$0.40B_0$	$0.50B_0$	$0.60B_0$
$\sigma^{2,2}$	−14.572873	−14.558742	−14.519300	−14.460490	−14.387301	−14.303122	−14.210358
$\sigma^{2,1}\pi_-^{1,0}$	−14.511708	−14.645083	−14.749747	−14.833623	−14.901985	−14.958143	−15.004879
$\sigma^{2,1}\pi_+^{1,0}$	−14.511708	−14.545083	−14.549747	−14.533623	−14.501985	−14.458143	−14.404879
$\sigma^{3,1}$	−14.513157	−14.601893	−14.670219	−14.722635	−14.763157	−14.794482	−14.818447
$\sigma^{2,1}\delta_-^{1,0}$	−14.090488	−14.272451	−14.419632	−14.535035	−14.622244	−14.685266	−14.728715
$\sigma^{1,1}\pi_-^{1,0}\delta_-^{1,0}$	−13.960561	−14.189478	−14.379489	−14.536288	−14.664228	−14.767311	−14.850303

Table S123: Total energies in E_h for the Be atom in the cc-pVQZ basis set in fully uncontracted form, employing the real-orbital approximation.

	$0.00B_0$	$0.10B_0$	$0.20B_0$	$0.30B_0$	$0.40B_0$	$0.50B_0$	$0.60B_0$
$\sigma^{2,2}$	−14.572968	−14.558837	−14.519401	−14.460543	−14.387215	−14.302913	−14.210192
$\sigma^{2,1}\pi_-^{1,0}$	−14.511920	−14.645320	−14.750188	−14.834093	−14.902416	−14.959174	−15.007414
$\sigma^{2,1}\pi_+^{1,0}$	−14.511920	−14.545320	−14.550188	−14.534093	−14.502416	−14.459174	−14.407414
$\sigma^{3,1}$	−14.513429	−14.602191	−14.670728	−14.723595	−14.764726	−14.796906	−14.822161
$\sigma^{2,1}\delta_-^{1,0}$	−14.086992	−14.269321	−14.417571	−14.534619	−14.623806	−14.688757	−14.733573
$\sigma^{1,1}\pi_-^{1,0}\delta_-^{1,0}$	−13.958282	−14.187577	−14.378739	−14.537086	−14.666911	−14.772388	−14.857928

Table S124: Total energies in E_h for the Be atom in the cc-pV5Z basis set in fully uncontracted form, employing the real-orbital approximation.

	$0.00B_0$	$0.10B_0$	$0.20B_0$	$0.30B_0$	$0.40B_0$	$0.50B_0$	$0.60B_0$
$\sigma^{2,2}$	−14.573012	−14.558898	−14.519647	−14.461256	−14.388602	−14.305011	−14.212841
$\sigma^{2,1}\pi_-^{1,0}$	−14.512017	−14.645423	−14.750360	−14.834431	−14.903415	−14.961134	−15.009992
$\sigma^{2,1}\pi_+^{1,0}$	−14.512017	−14.545423	−14.550360	−14.534431	−14.503415	−14.461134	−14.409992
$\sigma^{3,1}$	−14.513601	−14.602421	−14.671204	−14.724472	−14.766141	−14.799055	−14.825167
$\sigma^{2,1}\delta_-^{1,0}$	−14.147282	−14.327139	−14.468240	−14.574374	−14.650641	−14.703313	−14.739603
$\sigma^{1,1}\pi_-^{1,0}\delta_-^{1,0}$	−14.015881	−14.242914	−14.427305	−14.575182	−14.692905	−14.787104	−14.864785

Table S125: Total energies in E_h for the Be atom in the aug-cc-pVDZ basis set in fully uncontracted form, employing the real-orbital approximation.

	$0.00B_0$	$0.10B_0$	$0.20B_0$	$0.30B_0$	$0.40B_0$	$0.50B_0$	$0.60B_0$
$\sigma^{2,2}$	−14.572380	−14.558262	−14.519085	−14.460667	−14.387925	−14.304106	−14.211171
$\sigma^{2,1}\pi_-^{1,0}$	−14.510887	−14.644110	−14.748153	−14.831299	−14.899793	−14.956473	−15.002579
$\sigma^{2,1}\pi_+^{1,0}$	−14.510887	−14.544110	−14.548153	−14.531299	−14.499793	−14.456473	−14.402579
$\sigma^{3,1}$	−14.512481	−14.601256	−14.669522	−14.721366	−14.760856	−14.790873	−14.813002
$\sigma^{2,1}\delta_-^{1,0}$	−14.263666	−14.432939	−14.544001	−14.607702	−14.645160	−14.679704	−14.716742
$\sigma^{1,1}\pi_-^{1,0}\delta_-^{1,0}$	−14.124187	−14.341134	−14.496579	−14.603651	−14.684145	−14.760115	−14.836250

Table S126: Total energies in E_h for the Be atom in the aug-cc-pVTZ basis set in fully uncontracted form, employing the real-orbital approximation.

	$0.00B_0$	$0.10B_0$	$0.20B_0$	$0.30B_0$	$0.40B_0$	$0.50B_0$	$0.60B_0$
$\sigma^{2,2}$	−14.572876	−14.558811	−14.519938	−14.462055	−14.389560	−14.305558	−14.212504
$\sigma^{2,1}\pi_-^{1,0}$	−14.511776	−14.645110	−14.749993	−14.834513	−14.903785	−14.960851	−15.008247
$\sigma^{2,1}\pi_+^{1,0}$	−14.511776	−14.545110	−14.549993	−14.534513	−14.503785	−14.460851	−14.408247
$\sigma^{3,1}$	−14.513404	−14.602282	−14.671232	−14.724848	−14.767020	−14.800251	−14.826160
$\sigma^{2,1}\delta_-^{1,0}$	−14.280232	−14.445362	−14.547304	−14.607333	−14.653440	−14.695865	−14.731339
$\sigma^{1,1}\pi_-^{1,0}\delta_-^{1,0}$	−14.139470	−14.352686	−14.500210	−14.604942	−14.694394	−14.778370	−14.854249

Table S127: Total energies in E_h for the Be atom in the aug-cc-pVQZ basis set in fully uncontracted form, employing the real-orbital approximation.

	$0.00B_0$	$0.10B_0$	$0.20B_0$	$0.30B_0$	$0.40B_0$	$0.50B_0$	$0.60B_0$
$\sigma^{2,2}$	−14.572969	−14.558909	−14.520042	−14.462084	−14.389459	−14.305525	−14.213046
$\sigma^{2,1}\pi_-^{1,0}$	−14.511970	−14.645349	−14.750479	−14.834980	−14.903899	−14.960991	−15.009304
$\sigma^{2,1}\pi_+^{1,0}$	−14.511970	−14.545349	−14.550479	−14.534980	−14.503899	−14.460991	−14.409304
$\sigma^{3,1}$	−14.513628	−14.602544	−14.671778	−14.725860	−14.768305	−14.801566	−14.827568
$\sigma^{2,1}\delta_-^{1,0}$	−14.293033	−14.454417	−14.547223	−14.601580	−14.652507	−14.703017	−14.745476
$\sigma^{1,1}\pi_-^{1,0}\delta_-^{1,0}$	−14.151304	−14.360997	−14.500169	−14.600306	−14.694857	−14.787015	−14.870488

Table S128: Total energies in E_h for the Be atom in the aug-cc-pV5Z basis set in fully uncontracted form, employing the real-orbital approximation.

$\sigma^{2,2}$
$\sigma^{2,1}\pi_-^{1,0}$
$\sigma^{2,1}\pi_+^{1,0}$
$\sigma^{3,1}$
$\sigma^{2,1}\delta_-^{1,0}$
$\sigma^{1,1}\pi_-^{1,0}\delta_-^{1,0}$

Table S129: Total energies in E_h for the Be atom in the HGBSP1-5 basis set in fully uncontracted form, employing the real-orbital approximation.

	$0.00B_0$	$0.10B_0$	$0.20B_0$	$0.30B_0$	$0.40B_0$	$0.50B_0$	$0.60B_0$
$\sigma^{2,2}$	−14.572431	−14.558603	−14.519752	−14.461489	−14.388931	−14.305466	−14.213139
$\sigma^{2,1}\pi_-^{1,0}$	−14.511764	−14.645151	−14.749918	−14.833612	−14.901776	−14.958147	−15.005276
$\sigma^{2,1}\pi_+^{1,0}$	−14.511764	−14.545151	−14.549918	−14.533612	−14.501776	−14.458147	−14.405276
$\sigma^{3,1}$	−14.513429	−14.602300	−14.670927	−14.723396	−14.763370	−14.793404	−14.815259
$\sigma^{2,1}\delta_-^{1,0}$	−14.333911	−14.464176	−14.547405	−14.611411	−14.662224	−14.701448	−14.732863
$\sigma^{1,1}\pi_-^{1,0}\delta_-^{1,0}$	−14.187906	−14.369110	−14.500518	−14.609241	−14.702218	−14.782172	−14.853477

Table S130: Total energies in E_h for the Be atom in the HGBSP1-7 basis set in fully uncontracted form, employing the real-orbital approximation.

	$0.00B_0$	$0.10B_0$	$0.20B_0$	$0.30B_0$	$0.40B_0$	$0.50B_0$	$0.60B_0$
$\sigma^{2,2}$	−14.573020	−14.558964	−14.520113	−14.462223	−14.389873	−14.306304	−14.213772
$\sigma^{2,1}\pi_-^{1,0}$	−14.512052	−14.645434	−14.750318	−14.834181	−14.902497	−14.958966	−15.006079
$\sigma^{2,1}\pi_+^{1,0}$	−14.512052	−14.545434	−14.550318	−14.534181	−14.502497	−14.458966	−14.406079
$\sigma^{3,1}$	−14.513717	−14.602578	−14.671297	−14.723912	−14.763936	−14.793966	−14.815874
$\sigma^{2,1}\delta_-^{1,0}$	−14.334208	−14.464588	−14.548057	−14.612170	−14.662602	−14.703023	−14.735302
$\sigma^{1,1}\pi_-^{1,0}\delta_-^{1,0}$	−14.188163	−14.369467	−14.501075	−14.609776	−14.702540	−14.783880	−14.856154

Table S131: Total energies in E_h for the Be atom in the HGBSP1-9 basis set in fully uncontracted form, employing the real-orbital approximation.

	$0.00B_0$	$0.10B_0$	$0.20B_0$	$0.30B_0$	$0.40B_0$	$0.50B_0$	$0.60B_0$
$\sigma^{2,2}$	−14.573023	−14.558970	−14.520118	−14.462236	−14.389916	−14.306365	−14.213823
$\sigma^{2,1}\pi_-^{1,0}$	−14.512055	−14.645438	−14.750322	−14.834197	−14.902542	−14.959014	−15.006101
$\sigma^{2,1}\pi_+^{1,0}$	−14.512055	−14.545438	−14.550322	−14.534197	−14.502542	−14.459014	−14.406101
$\sigma^{3,1}$	−14.513720	−14.602582	−14.671302	−14.723923	−14.763960	−14.794003	−14.815915
$\sigma^{2,1}\delta_-^{1,0}$	−14.334213	−14.464606	−14.548125	−14.612184	−14.662796	−14.703120	−14.735309
$\sigma^{1,1}\pi_-^{1,0}\delta_-^{1,0}$	−14.188166	−14.369481	−14.501132	−14.609788	−14.702720	−14.783968	−14.856152

Table S132: Total energies in E_h for the Be atom in the HGBSP2-5 basis set in fully uncontracted form, employing the real-orbital approximation.

	$0.00B_0$	$0.10B_0$	$0.20B_0$	$0.30B_0$	$0.40B_0$	$0.50B_0$	$0.60B_0$
$\sigma^{2,2}$	−14.572431	−14.558603	−14.519752	−14.461489	−14.388931	−14.305466	−14.213139
$\sigma^{2,1}\pi_-^{1,0}$	−14.511764	−14.645182	−14.750233	−14.834586	−14.903794	−14.961531	−15.010234
$\sigma^{2,1}\pi_+^{1,0}$	−14.511764	−14.545182	−14.550233	−14.534586	−14.503794	−14.461531	−14.410234
$\sigma^{3,1}$	−14.513433	−14.602386	−14.671646	−14.725646	−14.768069	−14.801427	−14.827386
$\sigma^{2,1}\delta_-^{1,0}$	−14.333911	−14.464176	−14.547405	−14.611411	−14.662224	−14.701448	−14.732863
$\sigma^{1,1}\pi_-^{1,0}\delta_-^{1,0}$	−14.187908	−14.369125	−14.500672	−14.609826	−14.703577	−14.784620	−14.857243

Table S133: Total energies in E_h for the Be atom in the HGBSP2-7 basis set in fully uncontracted form, employing the real-orbital approximation.

	$0.00B_0$	$0.10B_0$	$0.20B_0$	$0.30B_0$	$0.40B_0$	$0.50B_0$	$0.60B_0$
$\sigma^{2,2}$	−14.573020	−14.558964	−14.520113	−14.462223	−14.389873	−14.306304	−14.213772
$\sigma^{2,1}\pi_-^{1,0}$	−14.512053	−14.645464	−14.750629	−14.835152	−14.904488	−14.962276	−15.010952
$\sigma^{2,1}\pi_+^{1,0}$	−14.512053	−14.545464	−14.550629	−14.535152	−14.504488	−14.462276	−14.410952
$\sigma^{3,1}$	−14.513721	−14.602665	−14.672009	−14.726145	−14.768619	−14.801942	−14.827868
$\sigma^{2,1}\delta_-^{1,0}$	−14.334208	−14.464588	−14.548057	−14.612170	−14.662602	−14.703023	−14.735302
$\sigma^{1,1}\pi_-^{1,0}\delta_-^{1,0}$	−14.188165	−14.369482	−14.501229	−14.610362	−14.703887	−14.786280	−14.859851

Table S134: Total energies in E_h for the Be atom in the HGBSP2-9 basis set in fully uncontracted form, employing the real-orbital approximation.

	$0.00B_0$	$0.10B_0$	$0.20B_0$	$0.30B_0$	$0.40B_0$	$0.50B_0$	$0.60B_0$
$\sigma^{2,2}$	−14.573023	−14.558970	−14.520118	−14.462236	−14.389916	−14.306365	−14.213823
$\sigma^{2,1}\pi_-^{1,0}$	−14.512056	−14.645468	−14.750634	−14.835168	−14.904529	−14.962319	−15.010974
$\sigma^{2,1}\pi_+^{1,0}$	−14.512056	−14.545468	−14.550634	−14.535168	−14.504529	−14.462319	−14.410974
$\sigma^{3,1}$	−14.513724	−14.602669	−14.672013	−14.726155	−14.768641	−14.801970	−14.827892
$\sigma^{2,1}\delta_-^{1,0}$	−14.334213	−14.464606	−14.548125	−14.612184	−14.662796	−14.703120	−14.735309
$\sigma^{1,1}\pi_-^{1,0}\delta_-^{1,0}$	−14.188168	−14.369496	−14.501285	−14.610374	−14.704064	−14.786363	−14.859849

Table S135: Total energies in E_h for the Be atom in the HGBSP3-5 basis set in fully uncontracted form, employing the real-orbital approximation.

	$0.00B_0$	$0.10B_0$	$0.20B_0$	$0.30B_0$	$0.40B_0$	$0.50B_0$	$0.60B_0$
$\sigma^{2,2}$	−14.572431	−14.558603	−14.519761	−14.461582	−14.389304	−14.306412	−14.215040
$\sigma^{2,1}\pi_-^{1,0}$	−14.511764	−14.645182	−14.750234	−14.834600	−14.903870	−14.961765	−15.010786
$\sigma^{2,1}\pi_+^{1,0}$	−14.511764	−14.545182	−14.550234	−14.534600	−14.503870	−14.461765	−14.410786
$\sigma^{3,1}$	−14.513433	−14.602387	−14.671660	−14.725728	−14.768341	−14.802073	−14.828648
$\sigma^{2,1}\delta_-^{1,0}$	−14.333911	−14.465164	−14.550545	−14.616975	−14.670568	−14.713284	−14.748228
$\sigma^{1,1}\pi_-^{1,0}\delta_-^{1,0}$	−14.187913	−14.370137	−14.503853	−14.615562	−14.712315	−14.797031	−14.873271

Table S136: Total energies in E_h for the Be atom in the HGBSP3-7 basis set in fully uncontracted form, employing the real-orbital approximation.

	$0.00B_0$	$0.10B_0$	$0.20B_0$	$0.30B_0$	$0.40B_0$	$0.50B_0$	$0.60B_0$
$\sigma^{2,2}$	−14.573020	−14.558964	−14.520121	−14.462296	−14.390169	−14.307118	−14.215525
$\sigma^{2,1}\pi_-^{1,0}$	−14.512053	−14.645464	−14.750630	−14.835161	−14.904544	−14.962478	−15.011469
$\sigma^{2,1}\pi_+^{1,0}$	−14.512053	−14.545464	−14.550630	−14.535161	−14.504544	−14.462478	−14.411469
$\sigma^{3,1}$	−14.513721	−14.602666	−14.672021	−14.726219	−14.768866	−14.802545	−14.829080
$\sigma^{2,1}\delta_-^{1,0}$	−14.334208	−14.465566	−14.551137	−14.617732	−14.671012	−14.714452	−14.750130
$\sigma^{1,1}\pi_-^{1,0}\delta_-^{1,0}$	−14.188169	−14.370486	−14.504355	−14.616116	−14.712695	−14.798303	−14.875356

Table S137: Total energies in E_h for the Be atom in the HGBSP3-9 basis set in fully uncontracted form, employing the real-orbital approximation.

	$0.00B_0$	$0.10B_0$	$0.20B_0$	$0.30B_0$	$0.40B_0$	$0.50B_0$	$0.60B_0$
$\sigma^{2,2}$	−14.573023	−14.558970	−14.520126	−14.462310	−14.390214	−14.307174	−14.215559
$\sigma^{2,1}\pi_-^{1,0}$	−14.512056	−14.645468	−14.750634	−14.835177	−14.904585	−14.962519	−15.011485
$\sigma^{2,1}\pi_+^{1,0}$	−14.512056	−14.545468	−14.550634	−14.535177	−14.504585	−14.462519	−14.411485
$\sigma^{3,1}$	−14.513724	−14.602669	−14.672026	−14.726230	−14.768888	−14.802571	−14.829099
$\sigma^{2,1}\delta_-^{1,0}$	−14.334213	−14.465582	−14.551189	−14.617745	−14.671147	−14.714561	−14.750147
$\sigma^{1,1}\pi_-^{1,0}\delta_-^{1,0}$	−14.188172	−14.370498	−14.504399	−14.616126	−14.712819	−14.798401	−14.875367

Table S138: Total energies in E_h for the Be atom in the AHGBSP1-5 basis set in fully uncontracted form, employing the real-orbital approximation.

	$0.00B_0$	$0.10B_0$	$0.20B_0$	$0.30B_0$	$0.40B_0$	$0.50B_0$	$0.60B_0$
$\sigma^{2,2}$	−14.572758	−14.558699	−14.519792	−14.461764	−14.389348	−14.305848	−14.213398
$\sigma^{2,1}\pi_-^{1,0}$	−14.511786	−14.645158	−14.750001	−14.833802	−14.901997	−14.958325	−15.005384
$\sigma^{2,1}\pi_+^{1,0}$	−14.511786	−14.545158	−14.550001	−14.533802	−14.501997	−14.458325	−14.405384
$\sigma^{3,1}$	−14.513450	−14.602305	−14.670989	−14.723560	−14.763569	−14.793572	−14.815373
$\sigma^{2,1}\delta_-^{1,0}$	−14.333935	−14.464197	−14.547463	−14.611533	−14.662358	−14.701555	−14.732924
$\sigma^{1,1}\pi_-^{1,0}\delta_-^{1,0}$	−14.187907	−14.369117	−14.500526	−14.609246	−14.702229	−14.782204	−14.853517

Table S139: Total energies in E_h for the Be atom in the AHGBSP1-7 basis set in fully uncontracted form, employing the real-orbital approximation.

	$0.00B_0$	$0.10B_0$	$0.20B_0$	$0.30B_0$	$0.40B_0$	$0.50B_0$	$0.60B_0$
$\sigma^{2,2}$	−14.573020	−14.558966	−14.520115	−14.462226	−14.389888	−14.306326	−14.213790
$\sigma^{2,1}\pi_-^{1,0}$	−14.512052	−14.645434	−14.750318	−14.834185	−14.902510	−14.958979	−15.006087
$\sigma^{2,1}\pi_+^{1,0}$	−14.512052	−14.545434	−14.550318	−14.534185	−14.502510	−14.458979	−14.406087
$\sigma^{3,1}$	−14.513717	−14.602579	−14.671297	−14.723915	−14.763945	−14.793977	−14.815884
$\sigma^{2,1}\delta_-^{1,0}$	−14.334209	−14.464591	−14.548062	−14.612174	−14.662615	−14.703035	−14.735308
$\sigma^{1,1}\pi_-^{1,0}\delta_-^{1,0}$	−14.188163	−14.369469	−14.501079	−14.609777	−14.702547	−14.783884	−14.856155

Table S140: Total energies in E_h for the Be atom in the AHGBSP1-9 basis set in fully uncontracted form, employing the real-orbital approximation.

	$0.00B_0$	$0.10B_0$	$0.20B_0$	$0.30B_0$	$0.40B_0$	$0.50B_0$	$0.60B_0$
$\sigma^{2,2}$	−14.573023	−14.558970	−14.520118	−14.462236	−14.389916	−14.306366	−14.213823
$\sigma^{2,1}\pi_-^{1,0}$	−14.512055	−14.645438	−14.750322	−14.834198	−14.902542	−14.959015	−15.006101
$\sigma^{2,1}\pi_+^{1,0}$	−14.512055	−14.545438	−14.550322	−14.534198	−14.502542	−14.459015	−14.406101
$\sigma^{3,1}$	−14.513720	−14.602582	−14.671302	−14.723923	−14.763961	−14.794004	−14.815915
$\sigma^{2,1}\delta_-^{1,0}$	−14.334213	−14.464606	−14.548125	−14.612184	−14.662797	−14.703121	−14.735309
$\sigma^{1,1}\pi_-^{1,0}\delta_-^{1,0}$	−14.188166	−14.369481	−14.501132	−14.609789	−14.702720	−14.783968	−14.856152

Table S141: Total energies in E_h for the Be atom in the AHGBSP2-5 basis set in fully uncontracted form, employing the real-orbital approximation.

	$0.00B_0$	$0.10B_0$	$0.20B_0$	$0.30B_0$	$0.40B_0$	$0.50B_0$	$0.60B_0$
$\sigma^{2,2}$	−14.572758	−14.558699	−14.519792	−14.461764	−14.389348	−14.305848	−14.213398
$\sigma^{2,1}\pi_-^{1,0}$	−14.511787	−14.645188	−14.750314	−14.834773	−14.904009	−14.961701	−15.010335
$\sigma^{2,1}\pi_+^{1,0}$	−14.511787	−14.545188	−14.550314	−14.534773	−14.504009	−14.461701	−14.410335
$\sigma^{3,1}$	−14.513454	−14.602392	−14.671703	−14.725797	−14.768254	−14.801581	−14.827483
$\sigma^{2,1}\delta_-^{1,0}$	−14.333935	−14.464197	−14.547463	−14.611533	−14.662358	−14.701555	−14.732924
$\sigma^{1,1}\pi_-^{1,0}\delta_-^{1,0}$	−14.187909	−14.369133	−14.500680	−14.609832	−14.703586	−14.784646	−14.857277

Table S142: Total energies in E_h for the Be atom in the AHGBSP2-7 basis set in fully uncontracted form, employing the real-orbital approximation.

	$0.00B_0$	$0.10B_0$	$0.20B_0$	$0.30B_0$	$0.40B_0$	$0.50B_0$	$0.60B_0$
$\sigma^{2,2}$	−14.573020	−14.558966	−14.520115	−14.462226	−14.389888	−14.306326	−14.213790
$\sigma^{2,1}\pi_-^{1,0}$	−14.512053	−14.645465	−14.750630	−14.835156	−14.904500	−14.962288	−15.010959
$\sigma^{2,1}\pi_+^{1,0}$	−14.512053	−14.545465	−14.550630	−14.535156	−14.504500	−14.462288	−14.410959
$\sigma^{3,1}$	−14.513721	−14.602666	−14.672009	−14.726147	−14.768627	−14.801951	−14.827875
$\sigma^{2,1}\delta_-^{1,0}$	−14.334209	−14.464591	−14.548062	−14.612174	−14.662615	−14.703035	−14.735308
$\sigma^{1,1}\pi_-^{1,0}\delta_-^{1,0}$	−14.188165	−14.369484	−14.501233	−14.610363	−14.703893	−14.786283	−14.859851

Table S143: Total energies in E_h for the Be atom in the AHGBSP2-9 basis set in fully uncontracted form, employing the real-orbital approximation.

	$0.00B_0$	$0.10B_0$	$0.20B_0$	$0.30B_0$	$0.40B_0$	$0.50B_0$	$0.60B_0$
$\sigma^{2,2}$	−14.573023	−14.558970	−14.520118	−14.462236	−14.389916	−14.306366	−14.213823
$\sigma^{2,1}\pi_-^{1,0}$	−14.512056	−14.645468	−14.750634	−14.835168	−14.904529	−14.962319	−15.010974
$\sigma^{2,1}\pi_+^{1,0}$	−14.512056	−14.545468	−14.550634	−14.535168	−14.504529	−14.462319	−14.410974
$\sigma^{3,1}$	−14.513724	−14.602669	−14.672013	−14.726155	−14.768641	−14.801970	−14.827892
$\sigma^{2,1}\delta_-^{1,0}$	−14.334213	−14.464606	−14.548125	−14.612184	−14.662797	−14.703121	−14.735309
$\sigma^{1,1}\pi_-^{1,0}\delta_-^{1,0}$	−14.188168	−14.369496	−14.501285	−14.610374	−14.704064	−14.786363	−14.859849

Table S144: Total energies in E_h for the Be atom in the AHGBSP3-5 basis set in fully uncontracted form, employing the real-orbital approximation.

	$0.00B_0$	$0.10B_0$	$0.20B_0$	$0.30B_0$	$0.40B_0$	$0.50B_0$	$0.60B_0$
$\sigma^{2,2}$	−14.572758	−14.558699	−14.519800	−14.461840	−14.389657	−14.306684	−14.215184
$\sigma^{2,1}\pi_-^{1,0}$	−14.511787	−14.645188	−14.750315	−14.834783	−14.904068	−14.961908	−15.010861
$\sigma^{2,1}\pi_+^{1,0}$	−14.511787	−14.545188	−14.550315	−14.534783	−14.504068	−14.461908	−14.410861
$\sigma^{3,1}$	−14.513454	−14.602392	−14.671716	−14.725872	−14.768505	−14.802193	−14.828712
$\sigma^{2,1}\delta_-^{1,0}$	−14.333935	−14.465185	−14.550599	−14.617090	−14.670686	−14.713363	−14.748265
$\sigma^{1,1}\pi_-^{1,0}\delta_-^{1,0}$	−14.187913	−14.370143	−14.503858	−14.615568	−14.712323	−14.797054	−14.873301

Table S145: Total energies in E_h for the Be atom in the AHGBSP3-7 basis set in fully uncontracted form, employing the real-orbital approximation.

	$0.00B_0$	$0.10B_0$	$0.20B_0$	$0.30B_0$	$0.40B_0$	$0.50B_0$	$0.60B_0$
$\sigma^{2,2}$	−14.573020	−14.558966	−14.520123	−14.462299	−14.390186	−14.307138	−14.215535
$\sigma^{2,1}\pi_-^{1,0}$	−14.512053	−14.645465	−14.750630	−14.835165	−14.904556	−14.962489	−15.011474
$\sigma^{2,1}\pi_+^{1,0}$	−14.512053	−14.545465	−14.550630	−14.535165	−14.504556	−14.462489	−14.411474
$\sigma^{3,1}$	−14.513721	−14.602666	−14.672022	−14.726222	−14.768874	−14.802553	−14.829084
$\sigma^{2,1}\delta_-^{1,0}$	−14.334209	−14.465569	−14.551141	−14.617735	−14.671023	−14.714462	−14.750134
$\sigma^{1,1}\pi_-^{1,0}\delta_-^{1,0}$	−14.188169	−14.370488	−14.504358	−14.616117	−14.712699	−14.798306	−14.875357

Table S146: Total energies in E_h for the Be atom in the AHGBSP3-9 basis set in fully uncontracted form, employing the real-orbital approximation.

	$0.00B_0$	$0.10B_0$	$0.20B_0$	$0.30B_0$	$0.40B_0$	$0.50B_0$	$0.60B_0$
$\sigma^{2,2}$	−14.573023	−14.558970	−14.520126	−14.462310	−14.390215	−14.307174	−14.215560
$\sigma^{2,1}\pi_-^{1,0}$	−14.512056	−14.645468	−14.750634	−14.835177	−14.904586	−14.962519	−15.011486
$\sigma^{2,1}\pi_+^{1,0}$	−14.512056	−14.545468	−14.550634	−14.535177	−14.504586	−14.462519	−14.411486
$\sigma^{3,1}$	−14.513724	−14.602669	−14.672026	−14.726230	−14.768889	−14.802571	−14.829099
$\sigma^{2,1}\delta_-^{1,0}$	−14.334213	−14.465582	−14.551189	−14.617745	−14.671148	−14.714562	−14.750147
$\sigma^{1,1}\pi_-^{1,0}\delta_-^{1,0}$	−14.188172	−14.370499	−14.504399	−14.616126	−14.712819	−14.798402	−14.875367

Table S147: Total energies in E_h for the Be atom in the 6-311++G(3df,3pd) basis set in fully uncontracted form, employing the real-orbital approximation.

	$0.00B_0$	$0.10B_0$	$0.20B_0$	$0.30B_0$	$0.40B_0$	$0.50B_0$	$0.60B_0$
$\sigma^{2,2}$	−14.572032	−14.557947	−14.518951	−14.460777	−14.387893	−14.303281	−14.209164
$\sigma^{2,1}\pi_-^{1,0}$	−14.510968	−14.644297	−14.749027	−14.833031	−14.901945	−14.958944	−15.006209
$\sigma^{2,1}\pi_+^{1,0}$	−14.510968	−14.544297	−14.549027	−14.533031	−14.501945	−14.458944	−14.406209
$\sigma^{3,1}$	−14.512601	−14.601441	−14.670121	−14.722893	−14.763684	−14.795390	−14.819985
$\sigma^{2,1}\delta_-^{1,0}$	−14.279774	−14.445519	−14.546301	−14.595090	−14.620840	−14.655190	−14.698885
$\sigma^{1,1}\pi_-^{1,0}\delta_-^{1,0}$	−14.139576	−14.353192	−14.499005	−14.592177	−14.662216	−14.739098	−14.823530

Table S148: Total energies in E_h for the Be atom in the def2-TZVP basis set in fully uncontracted form, employing the real-orbital approximation.

	$0.00B_0$	$0.10B_0$	$0.20B_0$	$0.30B_0$	$0.40B_0$	$0.50B_0$	$0.60B_0$
$\sigma^{2,2}$	−14.572746	−14.558621	−14.519189	−14.460041	−14.386251	−14.301865	−14.209593
$\sigma^{2,1}\pi_-^{1,0}$	−14.511174	−14.644544	−14.748617	−14.831498	−14.899529	−14.955933	−15.002298
$\sigma^{2,1}\pi_+^{1,0}$	−14.511174	−14.544544	−14.548617	−14.531498	−14.499529	−14.455933	−14.402298
$\sigma^{3,1}$	−14.512597	−14.601350	−14.669456	−14.721048	−14.760335	−14.790350	−14.812727
$\sigma^{2,1}\delta_-^{1,0}$	−14.074141	−14.257285	−14.407799	−14.527927	−14.619869	−14.685336	−14.725409
$\sigma^{1,1}\pi_-^{1,0}\delta_-^{1,0}$	−13.946351	−14.176189	−14.368550	−14.528945	−14.660987	−14.765861	−14.843952

Table S149: Total energies in E_h for the B atom in the cc-pVDZ basis set in fully uncontracted form, employing the real-orbital approximation.

	$0.00B_0$	$0.10B_0$	$0.20B_0$	$0.30B_0$	$0.40B_0$	$0.50B_0$	$0.60B_0$
$\sigma^{2,2}\pi_+^{1,0}$	−24.527567	−24.513634	−24.472959	−24.408549	−24.324351	−24.224262	−24.111526
$\sigma^{2,2}\pi_-^{1,0}$	−24.527567	−24.613634	−24.672959	−24.708549	−24.724351	−24.724262	−24.711526
$\sigma^{2,1}\pi_+^{1,0}\pi_-^{1,0}$	−24.448689	−24.583630	−24.689857	−24.771091	−24.832102	−24.877285	−24.909789
$\sigma^{3,1}\pi_-^{1,0}$	−24.447413	−24.635288	−24.799900	−24.943865	−25.070593	−25.183382	−25.284864
$\sigma^{2,1}\pi_-^{1,0}\delta_-^{1,0}$	−23.635845	−23.921382	−24.178775	−24.410061	−24.617800	−24.804348	−24.971483
$\sigma^{2,1}\pi_-^{2,0}$	−24.037585	−24.265097	−24.447946	−24.587058	−24.683979	−24.740946	−24.761146

Table S150: Total energies in E_h for the B atom in the cc-pVTZ basis set in fully uncontracted form, employing the real-orbital approximation.

	$0.00B_0$	$0.10B_0$	$0.20B_0$	$0.30B_0$	$0.40B_0$	$0.50B_0$	$0.60B_0$
$\sigma^{2,2}\pi_+^{1,0}$	−24.529273	−24.515270	−24.474868	−24.411932	−24.330705	−24.234583	−24.125916
$\sigma^{2,2}\pi_-^{1,0}$	−24.529273	−24.615270	−24.674868	−24.711932	−24.730705	−24.734583	−24.725916
$\sigma^{2,1}\pi_+^{1,0}\pi_-^{1,0}$	−24.451411	−24.586424	−24.693276	−24.776283	−24.840019	−24.887881	−24.922074
$\sigma^{3,1}\pi_-^{1,0}$	−24.449931	−24.637921	−24.803165	−24.948730	−25.078007	−25.193757	−25.297961
$\sigma^{2,1}\pi_-^{1,0}\delta_-^{1,0}$	−23.904146	−24.186361	−24.433888	−24.648911	−24.834036	−24.991787	−25.124610
$\sigma^{2,1}\pi_-^{2,0}$	−24.107058	−24.329915	−24.499257	−24.617556	−24.689612	−24.818655	−24.932884

Table S151: Total energies in E_h for the B atom in the cc-pVQZ basis set in fully uncontracted form, employing the real-orbital approximation.

	$0.00B_0$	$0.10B_0$	$0.20B_0$	$0.30B_0$	$0.40B_0$	$0.50B_0$	$0.60B_0$
$\sigma^{2,2}\pi_+^{1,0}$	−24.530102	−24.516087	−24.475902	−24.413624	−24.333203	−24.237610	−24.129044
$\sigma^{2,2}\pi_-^{1,0}$	−24.530102	−24.616087	−24.675902	−24.713624	−24.733203	−24.737610	−24.729044
$\sigma^{2,1}\pi_+^{1,0}\pi_-^{1,0}$	−24.452474	−24.587530	−24.694690	−24.778316	−24.842573	−24.890582	−24.924781
$\sigma^{3,1}\pi_-^{1,0}$	−24.450960	−24.638999	−24.804527	−24.950707	−25.080745	−25.197219	−25.302174
$\sigma^{2,1}\pi_-^{1,0}\delta_-^{1,0}$	−24.000880	−24.279652	−24.517074	−24.716052	−24.880703	−25.016291	−25.129403
$\sigma^{2,1}\pi_-^{2,0}$	−24.143349	−24.361732	−24.518757	−24.620958	−24.747556	−24.865239	−24.961828

Table S152: Total energies in E_h for the B atom in the cc-pV5Z basis set in fully uncontracted form, employing the real-orbital approximation.

	$0.00B_0$	$0.10B_0$	$0.20B_0$	$0.30B_0$	$0.40B_0$	$0.50B_0$	$0.60B_0$
$\sigma^{2,2}\pi_+^{1,0}$	−24.530245	−24.516238	−24.476182	−24.414084	−24.333763	−24.238282	−24.129980
$\sigma^{2,2}\pi_-^{1,0}$	−24.530245	−24.616238	−24.676182	−24.714084	−24.733763	−24.738282	−24.729980
$\sigma^{2,1}\pi_+^{1,0}\pi_-^{1,0}$	−24.452698	−24.587780	−24.695085	−24.778868	−24.843239	−24.891596	−24.926602
$\sigma^{3,1}\pi_-^{1,0}$	−24.451173	−24.639235	−24.804905	−24.951339	−25.081721	−25.198740	−25.304534
$\sigma^{2,1}\pi_-^{1,0}\delta_-^{1,0}$	−24.035570	−24.312099	−24.543145	−24.732836	−24.887720	−25.016643	−25.129435
$\sigma^{2,1}\pi_-^{2,0}$	−24.176135	−24.386697	−24.525877	−24.627926	−24.762569	−24.873098	−24.965234

Table S153: Total energies in E_h for the B atom in the aug-cc-pVDZ basis set in fully uncontracted form, employing the real-orbital approximation.

	$0.00B_0$	$0.10B_0$	$0.20B_0$	$0.30B_0$	$0.40B_0$	$0.50B_0$	$0.60B_0$
$\sigma^{2,2}\pi_+^{1,0}$	−24.527941	−24.513834	−24.473318	−24.410049	−24.327914	−24.230377	−24.120115
$\sigma^{2,2}\pi_-^{1,0}$	−24.527941	−24.613834	−24.673318	−24.710049	−24.727914	−24.730377	−24.720115
$\sigma^{2,1}\pi_+^{1,0}\pi_-^{1,0}$	−24.448917	−24.583799	−24.690230	−24.772147	−24.834064	−24.880079	−24.913174
$\sigma^{3,1}\pi_-^{1,0}$	−24.447528	−24.635429	−24.800380	−24.945151	−25.072942	−25.186754	−25.288989
$\sigma^{2,1}\pi_-^{1,0}\delta_-^{1,0}$	−24.081120	−24.353346	−24.571621	−24.740960	−24.871008	−24.977643	−25.079014
$\sigma^{2,1}\pi_-^{2,0}$	−24.199849	−24.389627	−24.497749	−24.646878	−24.762255	−24.850805	−24.922166

Table S154: Total energies in E_h for the B atom in the aug-cc-pVTZ basis set in fully uncontracted form, employing the real-orbital approximation.

	$0.00B_0$	$0.10B_0$	$0.20B_0$	$0.30B_0$	$0.40B_0$	$0.50B_0$	$0.60B_0$
$\sigma^{2,2}\pi_+^{1,0}$	−24.529338	−24.515298	−24.475124	−24.412816	−24.332342	−24.236824	−24.128510
$\sigma^{2,2}\pi_-^{1,0}$	−24.529338	−24.615298	−24.675124	−24.712816	−24.732342	−24.736824	−24.728510
$\sigma^{2,1}\pi_+^{1,0}\pi_-^{1,0}$	−24.451442	−24.586468	−24.693575	−24.777152	−24.841618	−24.890269	−24.925269
$\sigma^{3,1}\pi_-^{1,0}$	−24.449947	−24.637973	−24.803530	−24.949867	−25.080353	−25.197644	−25.303595
$\sigma^{2,1}\pi_-^{1,0}\delta_-^{1,0}$	−24.127903	−24.390734	−24.585423	−24.734588	−24.871410	−25.005444	−25.128662
$\sigma^{2,1}\pi_-^{2,0}$	−24.203420	−24.391060	−24.521683	−24.650663	−24.756227	−24.855385	−24.950362

Table S155: Total energies in E_h for the B atom in the aug-cc-pVQZ basis set in fully uncontracted form, employing the real-orbital approximation.

	$0.00B_0$	$0.10B_0$	$0.20B_0$	$0.30B_0$	$0.40B_0$	$0.50B_0$	$0.60B_0$
$\sigma^{2,2}\pi_+^{1,0}$	−24.530114	−24.516100	−24.476068	−24.414053	−24.333832	−24.238337	−24.129848
$\sigma^{2,2}\pi_-^{1,0}$	−24.530114	−24.616100	−24.676068	−24.714053	−24.733832	−24.738337	−24.729848
$\sigma^{2,1}\pi_+^{1,0}\pi_-^{1,0}$	−24.452478	−24.587552	−24.694871	−24.778778	−24.843326	−24.891578	−24.925954
$\sigma^{3,1}\pi_-^{1,0}$	−24.450962	−24.639026	−24.804762	−24.951416	−25.082116	−25.199302	−25.304900
$\sigma^{2,1}\pi_-^{1,0}\delta_-^{1,0}$	−24.147422	−24.402199	−24.583348	−24.738369	−24.886851	−25.020085	−25.136697
$\sigma^{2,1}\pi_-^{2,0}$	−24.205032	−24.392540	−24.524997	−24.649194	−24.766531	−24.876900	−24.976156

Table S156: Total energies in E_h for the B atom in the aug-cc-pV5Z basis set in fully uncontracted form, employing the real-orbital approximation.

	$0.00B_0$	$0.10B_0$	$0.20B_0$	$0.30B_0$	$0.40B_0$	$0.50B_0$	$0.60B_0$
$\sigma^{2,2}\pi_+^{1,0}$	−24.530249	−24.516248	−24.476273	−24.414292	−24.334033	−24.238573	−24.130356
$\sigma^{2,2}\pi_-^{1,0}$	−24.530249	−24.616248	−24.676273	−24.714292	−24.734033	−24.738573	−24.730356
$\sigma^{2,1}\pi_+^{1,0}\pi_-^{1,0}$	−24.452699	−24.587791	−24.695172	−24.779081	−24.843550	−24.891933	−24.926919
$\sigma^{3,1}\pi_-^{1,0}$	−24.451173	−24.639251	−24.805040	−24.951720	−25.082380	−25.199613	−25.305539
$\sigma^{2,1}\pi_-^{1,0}\delta_-^{1,0}$	−24.152184	−24.404077	−24.583826	−24.743503	−24.891712	−25.023183	−25.140757
$\sigma^{2,1}\pi_-^{2,0}$	−24.205754	−24.395219	−24.531796	−24.653191	−24.775258	−24.887527	−24.988359

Table S157: Total energies in E_h for the B atom in the HGBSP1-5 basis set in fully uncontracted form, employing the real-orbital approximation.

	$0.00B_0$	$0.10B_0$	$0.20B_0$	$0.30B_0$	$0.40B_0$	$0.50B_0$	$0.60B_0$
$\sigma^{2,2}\pi_+^{1,0}$	−24.529819	−24.515814	−24.475744	−24.413480	−24.332731	−24.236528	−24.127303
$\sigma^{2,2}\pi_-^{1,0}$	−24.529819	−24.615814	−24.675744	−24.713480	−24.732731	−24.736528	−24.727303
$\sigma^{2,1}\pi_+^{1,0}\pi_-^{1,0}$	−24.452272	−24.587354	−24.694562	−24.777979	−24.841614	−24.888716	−24.921857
$\sigma^{3,1}\pi_-^{1,0}$	−24.450746	−24.638795	−24.804311	−24.950193	−25.079403	−25.194451	−25.297380
$\sigma^{2,1}\pi_-^{1,0}\delta_-^{1,0}$	−24.176694	−24.405695	−24.586422	−24.745254	−24.886824	−25.015313	−25.131320
$\sigma^{2,1}\pi_-^{2,0}$	−24.176769	−24.371977	−24.523364	−24.654250	−24.767904	−24.868218	−24.957698

Table S158: Total energies in E_h for the B atom in the HGBSP1-7 basis set in fully uncontracted form, employing the real-orbital approximation.

	$0.00B_0$	$0.10B_0$	$0.20B_0$	$0.30B_0$	$0.40B_0$	$0.50B_0$	$0.60B_0$
$\sigma^{2,2}\pi_+^{1,0}$	−24.530265	−24.516264	−24.476214	−24.414014	−24.333371	−24.237306	−24.128228
$\sigma^{2,2}\pi_-^{1,0}$	−24.530265	−24.616264	−24.676214	−24.714014	−24.733371	−24.737306	−24.728228
$\sigma^{2,1}\pi_+^{1,0}\pi_-^{1,0}$	−24.452724	−24.587811	−24.695044	−24.778500	−24.842179	−24.889372	−24.922696
$\sigma^{3,1}\pi_-^{1,0}$	−24.451197	−24.639251	−24.804787	−24.950706	−25.079957	−25.195062	−25.298085
$\sigma^{2,1}\pi_-^{1,0}\delta_-^{1,0}$	−24.177166	−24.406190	−24.587019	−24.746026	−24.888276	−25.016184	−25.131741
$\sigma^{2,1}\pi_-^{2,0}$	−24.205982	−24.396871	−24.525380	−24.654717	−24.768957	−24.869730	−24.958746

Table S159: Total energies in E_h for the B atom in the HGBSP1-9 basis set in fully uncontracted form, employing the real-orbital approximation.

	$0.00B_0$	$0.10B_0$	$0.20B_0$	$0.30B_0$	$0.40B_0$	$0.50B_0$	$0.60B_0$
$\sigma^{2,2}\pi_+^{1,0}$	−24.530271	−24.516270	−24.476223	−24.414022	−24.333381	−24.237337	−24.128293
$\sigma^{2,2}\pi_-^{1,0}$	−24.530271	−24.616270	−24.676223	−24.714022	−24.733381	−24.737337	−24.728293
$\sigma^{2,1}\pi_+^{1,0}\pi_-^{1,0}$	−24.452729	−24.587816	−24.695050	−24.778508	−24.842192	−24.889405	−24.922760
$\sigma^{3,1}\pi_-^{1,0}$	−24.451202	−24.639256	−24.804793	−24.950713	−25.079969	−25.195087	−25.298128
$\sigma^{2,1}\pi_-^{1,0}\delta_-^{1,0}$	−24.177173	−24.406198	−24.587027	−24.746129	−24.888304	−25.016240	−25.131999
$\sigma^{2,1}\pi_-^{2,0}$	−24.205990	−24.396885	−24.525578	−24.654746	−24.769072	−24.869757	−24.958790

Table S160: Total energies in E_h for the B atom in the HGBSP2-5 basis set in fully uncontracted form, employing the real-orbital approximation.

	$0.00B_0$	$0.10B_0$	$0.20B_0$	$0.30B_0$	$0.40B_0$	$0.50B_0$	$0.60B_0$
$\sigma^{2,2}\pi_+^{1,0}$	−24.529822	−24.515821	−24.475833	−24.413807	−24.333464	−24.237837	−24.129357
$\sigma^{2,2}\pi_-^{1,0}$	−24.529822	−24.615821	−24.675833	−24.713807	−24.733464	−24.737837	−24.729357
$\sigma^{2,1}\pi_+^{1,0}\pi_-^{1,0}$	−24.452275	−24.587363	−24.694723	−24.778613	−24.843118	−24.891517	−24.926391
$\sigma^{3,1}\pi_-^{1,0}$	−24.450747	−24.638822	−24.804595	−24.951249	−25.081908	−25.199139	−25.304996
$\sigma^{2,1}\pi_-^{1,0}\delta_-^{1,0}$	−24.151547	−24.405701	−24.586459	−24.745408	−24.887241	−25.016173	−25.132815
$\sigma^{2,1}\pi_-^{2,0}$	−24.205521	−24.398691	−24.535741	−24.655178	−24.769726	−24.871226	−24.962115

Table S161: Total energies in E_h for the B atom in the HGBSP2-7 basis set in fully uncontracted form, employing the real-orbital approximation.

	$0.00B_0$	$0.10B_0$	$0.20B_0$	$0.30B_0$	$0.40B_0$	$0.50B_0$	$0.60B_0$
$\sigma^{2,2}\pi_+^{1,0}$	−24.530268	−24.516270	−24.476303	−24.414339	−24.334104	−24.238615	−24.130265
$\sigma^{2,2}\pi_-^{1,0}$	−24.530268	−24.616270	−24.676303	−24.714339	−24.734104	−24.738615	−24.730265
$\sigma^{2,1}\pi_+^{1,0}\pi_-^{1,0}$	−24.452726	−24.587820	−24.695204	−24.779132	−24.843685	−24.892171	−24.927194
$\sigma^{3,1}\pi_-^{1,0}$	−24.451198	−24.639278	−24.805070	−24.951758	−25.082458	−25.199747	−25.305685
$\sigma^{2,1}\pi_-^{1,0}\delta_-^{1,0}$	−24.177166	−24.406195	−24.587056	−24.746179	−24.888693	−25.017043	−25.133226
$\sigma^{2,1}\pi_-^{2,0}$	−24.206002	−24.399235	−24.537046	−24.655646	−24.770779	−24.872709	−24.963115

Table S162: Total energies in E_h for the B atom in the HGBSP2-9 basis set in fully uncontracted form, employing the real-orbital approximation.

	$0.00B_0$	$0.10B_0$	$0.20B_0$	$0.30B_0$	$0.40B_0$	$0.50B_0$	$0.60B_0$
$\sigma^{2,2}\pi_+^{1,0}$	−24.530274	−24.516276	−24.476312	−24.414347	−24.334114	−24.238645	−24.130327
$\sigma^{2,2}\pi_-^{1,0}$	−24.530274	−24.616276	−24.676312	−24.714347	−24.734114	−24.738645	−24.730327
$\sigma^{2,1}\pi_+^{1,0}\pi_-^{1,0}$	−24.452731	−24.587826	−24.695210	−24.779139	−24.843698	−24.892203	−24.927251
$\sigma^{3,1}\pi_-^{1,0}$	−24.451203	−24.639283	−24.805076	−24.951765	−25.082469	−25.199771	−25.305724
$\sigma^{2,1}\pi_-^{1,0}\delta_-^{1,0}$	−24.177173	−24.406203	−24.587063	−24.746282	−24.888721	−25.017097	−25.133482
$\sigma^{2,1}\pi_-^{2,0}$	−24.206009	−24.399250	−24.537157	−24.655675	−24.770891	−24.872733	−24.963159

Table S163: Total energies in E_h for the B atom in the HGBSP3-5 basis set in fully uncontracted form, employing the real-orbital approximation.

	$0.00B_0$	$0.10B_0$	$0.20B_0$	$0.30B_0$	$0.40B_0$	$0.50B_0$	$0.60B_0$
$\sigma^{2,2}\pi_+^{1,0}$	−24.529822	−24.515821	−24.475834	−24.413816	−24.333507	−24.237975	−24.129698
$\sigma^{2,2}\pi_-^{1,0}$	−24.529822	−24.615821	−24.675834	−24.713816	−24.733507	−24.737975	−24.729698
$\sigma^{2,1}\pi_+^{1,0}\pi_-^{1,0}$	−24.452275	−24.587364	−24.694724	−24.778614	−24.843121	−24.891526	−24.926422
$\sigma^{3,1}\pi_-^{1,0}$	−24.450747	−24.638822	−24.804595	−24.951254	−25.081928	−25.199198	−25.305138
$\sigma^{2,1}\pi_-^{1,0}\delta_-^{1,0}$	−24.176696	−24.406760	−24.589541	−24.750851	−24.895205	−25.026513	−25.145878
$\sigma^{2,1}\pi_-^{2,0}$	−24.205521	−24.398691	−24.535742	−24.670742	−24.792871	−24.901915	−24.999991

Table S164: Total energies in E_h for the B atom in the HGBSP3-7 basis set in fully uncontracted form, employing the real-orbital approximation.

	$0.00B_0$	$0.10B_0$	$0.20B_0$	$0.30B_0$	$0.40B_0$	$0.50B_0$	$0.60B_0$
$\sigma^{2,2}\pi_+^{1,0}$	−24.530268	−24.516271	−24.476305	−24.414348	−24.334147	−24.238747	−24.130588
$\sigma^{2,2}\pi_-^{1,0}$	−24.530268	−24.616271	−24.676305	−24.714348	−24.734147	−24.738747	−24.730588
$\sigma^{2,1}\pi_+^{1,0}\pi_-^{1,0}$	−24.452726	−24.587821	−24.695205	−24.779133	−24.843687	−24.892179	−24.927221
$\sigma^{3,1}\pi_-^{1,0}$	−24.451198	−24.639278	−24.805070	−24.951762	−25.082477	−25.199805	−25.305823
$\sigma^{2,1}\pi_-^{1,0}\delta_-^{1,0}$	−24.177168	−24.407250	−24.590144	−24.751567	−24.896479	−25.027470	−25.146496
$\sigma^{2,1}\pi_-^{2,0}$	−24.206002	−24.399235	−24.537046	−24.671322	−24.793605	−24.902875	−25.001101

Table S165: Total energies in E_h for the B atom in the HGBSP3-9 basis set in fully uncontracted form, employing the real-orbital approximation.

	$0.00B_0$	$0.10B_0$	$0.20B_0$	$0.30B_0$	$0.40B_0$	$0.50B_0$	$0.60B_0$
$\sigma^{2,2}\pi_+^{1,0}$	−24.530274	−24.516276	−24.476314	−24.414357	−24.334157	−24.238779	−24.130653
$\sigma^{2,2}\pi_-^{1,0}$	−24.530274	−24.616276	−24.676314	−24.714357	−24.734157	−24.738779	−24.730653
$\sigma^{2,1}\pi_+^{1,0}\pi_-^{1,0}$	−24.452732	−24.587826	−24.695211	−24.779141	−24.843700	−24.892211	−24.927278
$\sigma^{3,1}\pi_-^{1,0}$	−24.451203	−24.639283	−24.805076	−24.951769	−25.082488	−25.199829	−25.305862
$\sigma^{2,1}\pi_-^{1,0}\delta_-^{1,0}$	−24.177175	−24.407259	−24.590156	−24.751640	−24.896522	−25.027507	−25.146661
$\sigma^{2,1}\pi_-^{2,0}$	−24.206010	−24.399250	−24.537158	−24.671338	−24.793644	−24.902943	−25.001143

Table S166: Total energies in E_h for the B atom in the AHGBSP1-5 basis set in fully uncontracted form, employing the real-orbital approximation.

	$0.00B_0$	$0.10B_0$	$0.20B_0$	$0.30B_0$	$0.40B_0$	$0.50B_0$	$0.60B_0$
$\sigma^{2,2}\pi_+^{1,0}$	−24.529820	−24.515816	−24.475749	−24.413499	−24.332772	−24.236593	−24.127383
$\sigma^{2,2}\pi_-^{1,0}$	−24.529820	−24.615816	−24.675749	−24.713499	−24.732772	−24.736593	−24.727383
$\sigma^{2,1}\pi_+^{1,0}\pi_-^{1,0}$	−24.452276	−24.587358	−24.694571	−24.777997	−24.841642	−24.888752	−24.921897
$\sigma^{3,1}\pi_-^{1,0}$	−24.450749	−24.638799	−24.804319	−24.950210	−25.079429	−25.194484	−25.297416
$\sigma^{2,1}\pi_-^{1,0}\delta_-^{1,0}$	−24.176702	−24.405702	−24.586436	−24.745272	−24.886860	−25.015341	−25.131343
$\sigma^{2,1}\pi_-^{2,0}$	−24.205511	−24.396323	−24.524004	−24.654259	−24.767931	−24.868255	−24.957726

Table S167: Total energies in E_h for the B atom in the AHGBSP1-7 basis set in fully uncontracted form, employing the real-orbital approximation.

	$0.00B_0$	$0.10B_0$	$0.20B_0$	$0.30B_0$	$0.40B_0$	$0.50B_0$	$0.60B_0$
$\sigma^{2,2}\pi_+^{1,0}$	−24.530266	−24.516264	−24.476218	−24.414016	−24.333372	−24.237314	−24.128246
$\sigma^{2,2}\pi_-^{1,0}$	−24.530266	−24.616264	−24.676218	−24.714016	−24.733372	−24.737314	−24.728246
$\sigma^{2,1}\pi_+^{1,0}\pi_-^{1,0}$	−24.452724	−24.587811	−24.695044	−24.778500	−24.842182	−24.889380	−24.922711
$\sigma^{3,1}\pi_-^{1,0}$	−24.451197	−24.639251	−24.804787	−24.950706	−25.079959	−25.195069	−25.298096
$\sigma^{2,1}\pi_-^{1,0}\delta_-^{1,0}$	−24.177166	−24.406191	−24.587020	−24.746029	−24.888279	−25.016191	−25.131754
$\sigma^{2,1}\pi_-^{2,0}$	−24.205984	−24.396876	−24.525414	−24.654718	−24.768962	−24.869735	−24.958754

Table S168: Total energies in E_h for the B atom in the AHGBSP1-9 basis set in fully uncontracted form, employing the real-orbital approximation.

	$0.00B_0$	$0.10B_0$	$0.20B_0$	$0.30B_0$	$0.40B_0$	$0.50B_0$	$0.60B_0$
$\sigma^{2,2}\pi_+^{1,0}$	−24.530271	−24.516270	−24.476224	−24.414023	−24.333381	−24.237337	−24.128294
$\sigma^{2,2}\pi_-^{1,0}$	−24.530271	−24.616270	−24.676224	−24.714023	−24.733381	−24.737337	−24.728294
$\sigma^{2,1}\pi_+^{1,0}\pi_-^{1,0}$	−24.452729	−24.587816	−24.695050	−24.778508	−24.842192	−24.889406	−24.922760
$\sigma^{3,1}\pi_-^{1,0}$	−24.451202	−24.639256	−24.804793	−24.950713	−25.079969	−25.195087	−25.298129
$\sigma^{2,1}\pi_-^{1,0}\delta_-^{1,0}$	−24.177173	−24.406198	−24.587027	−24.746129	−24.888304	−25.016240	−25.131999
$\sigma^{2,1}\pi_-^{2,0}$	−24.205990	−24.396886	−24.525581	−24.654746	−24.769072	−24.869757	−24.958791

Table S169: Total energies in E_h for the B atom in the AHGBSP2-5 basis set in fully uncontracted form, employing the real-orbital approximation.

	$0.00B_0$	$0.10B_0$	$0.20B_0$	$0.30B_0$	$0.40B_0$	$0.50B_0$	$0.60B_0$
$\sigma^{2,2}\pi_+^{1,0}$	−24.529823	−24.515822	−24.475838	−24.413825	−24.333505	−24.237901	−24.129436
$\sigma^{2,2}\pi_-^{1,0}$	−24.529823	−24.615822	−24.675838	−24.713825	−24.733505	−24.737901	−24.729436
$\sigma^{2,1}\pi_+^{1,0}\pi_-^{1,0}$	−24.452278	−24.587368	−24.694732	−24.778631	−24.843147	−24.891552	−24.926428
$\sigma^{3,1}\pi_-^{1,0}$	−24.450750	−24.638826	−24.804603	−24.951265	−25.081933	−25.199170	−25.305029
$\sigma^{2,1}\pi_-^{1,0}\delta_-^{1,0}$	−24.176702	−24.405708	−24.586473	−24.745426	−24.887276	−25.016201	−25.132838
$\sigma^{2,1}\pi_-^{2,0}$	−24.205530	−24.398701	−24.535868	−24.655186	−24.769754	−24.871263	−24.962141

Table S170: Total energies in E_h for the B atom in the AHGBSP2-7 basis set in fully uncontracted form, employing the real-orbital approximation.

	$0.00B_0$	$0.10B_0$	$0.20B_0$	$0.30B_0$	$0.40B_0$	$0.50B_0$	$0.60B_0$
$\sigma^{2,2}\pi_+^{1,0}$	−24.530268	−24.516271	−24.476306	−24.414341	−24.334105	−24.238622	−24.130283
$\sigma^{2,2}\pi_-^{1,0}$	−24.530268	−24.616271	−24.676306	−24.714341	−24.734105	−24.738622	−24.730283
$\sigma^{2,1}\pi_+^{1,0}\pi_-^{1,0}$	−24.452726	−24.587820	−24.695204	−24.779132	−24.843687	−24.892179	−24.927208
$\sigma^{3,1}\pi_-^{1,0}$	−24.451198	−24.639278	−24.805070	−24.951758	−25.082460	−25.199753	−25.305695
$\sigma^{2,1}\pi_-^{1,0}\delta_-^{1,0}$	−24.177167	−24.406196	−24.587056	−24.746183	−24.888696	−25.017049	−25.133239
$\sigma^{2,1}\pi_-^{2,0}$	−24.206003	−24.399240	−24.537062	−24.655647	−24.770783	−24.872714	−24.963122

Table S171: Total energies in E_h for the B atom in the AHGBSP2-9 basis set in fully uncontracted form, employing the real-orbital approximation.

	$0.00B_0$	$0.10B_0$	$0.20B_0$	$0.30B_0$	$0.40B_0$	$0.50B_0$	$0.60B_0$
$\sigma^{2,2}\pi_+^{1,0}$	−24.530274	−24.516276	−24.476312	−24.414347	−24.334114	−24.238645	−24.130328
$\sigma^{2,2}\pi_-^{1,0}$	−24.530274	−24.616276	−24.676312	−24.714347	−24.734114	−24.738645	−24.730328
$\sigma^{2,1}\pi_+^{1,0}\pi_-^{1,0}$	−24.452731	−24.587826	−24.695210	−24.779139	−24.843698	−24.892203	−24.927251
$\sigma^{3,1}\pi_-^{1,0}$	−24.451203	−24.639283	−24.805076	−24.951765	−25.082469	−25.199771	−25.305724
$\sigma^{2,1}\pi_-^{1,0}\delta_-^{1,0}$	−24.177173	−24.406203	−24.587063	−24.746283	−24.888721	−25.017098	−25.133483
$\sigma^{2,1}\pi_-^{2,0}$	−24.206010	−24.399251	−24.537158	−24.655675	−24.770891	−24.872734	−24.963159

Table S172: Total energies in E_h for the B atom in the AHGBSP3-5 basis set in fully uncontracted form, employing the real-orbital approximation.

	$0.00B_0$	$0.10B_0$	$0.20B_0$	$0.30B_0$	$0.40B_0$	$0.50B_0$	$0.60B_0$
$\sigma^{2,2}\pi_+^{1,0}$	−24.529823	−24.515822	−24.475839	−24.413835	−24.333549	−24.238039	−24.129771
$\sigma^{2,2}\pi_-^{1,0}$	−24.529823	−24.615822	−24.675839	−24.713835	−24.733549	−24.738039	−24.729771
$\sigma^{2,1}\pi_+^{1,0}\pi_-^{1,0}$	−24.452278	−24.587368	−24.694733	−24.778632	−24.843149	−24.891561	−24.926458
$\sigma^{3,1}\pi_-^{1,0}$	−24.450750	−24.638826	−24.804603	−24.951270	−25.081953	−25.199229	−25.305169
$\sigma^{2,1}\pi_-^{1,0}\delta_-^{1,0}$	−24.176705	−24.406766	−24.589556	−24.750863	−24.895235	−25.026541	−25.145899
$\sigma^{2,1}\pi_-^{2,0}$	−24.205530	−24.398701	−24.535869	−24.670756	−24.792884	−24.901936	−25.000017

Table S173: Total energies in E_h for the B atom in the AHGBSP3-7 basis set in fully uncontracted form, employing the real-orbital approximation.

	$0.00B_0$	$0.10B_0$	$0.20B_0$	$0.30B_0$	$0.40B_0$	$0.50B_0$	$0.60B_0$
$\sigma^{2,2}\pi_+^{1,0}$	−24.530268	−24.516271	−24.476308	−24.414350	−24.334148	−24.238756	−24.130608
$\sigma^{2,2}\pi_-^{1,0}$	−24.530268	−24.616271	−24.676308	−24.714350	−24.734148	−24.738756	−24.730608
$\sigma^{2,1}\pi_+^{1,0}\pi_-^{1,0}$	−24.452726	−24.587821	−24.695205	−24.779134	−24.843690	−24.892187	−24.927235
$\sigma^{3,1}\pi_-^{1,0}$	−24.451198	−24.639278	−24.805071	−24.951762	−25.082479	−25.199811	−25.305834
$\sigma^{2,1}\pi_-^{1,0}\delta_-^{1,0}$	−24.177169	−24.407251	−24.590144	−24.751569	−24.896482	−25.027476	−25.146507
$\sigma^{2,1}\pi_-^{2,0}$	−24.206003	−24.399240	−24.537062	−24.671323	−24.793608	−24.902881	−25.001108

Table S174: Total energies in E_h for the B atom in the AHGBSP3-9 basis set in fully uncontracted form, employing the real-orbital approximation.

	$0.00B_0$	$0.10B_0$	$0.20B_0$	$0.30B_0$	$0.40B_0$	$0.50B_0$	$0.60B_0$
$\sigma^{2,2}\pi_+^{1,0}$	−24.530274	−24.516276	−24.476314	−24.414357	−24.334157	−24.238779	−24.130654
$\sigma^{2,2}\pi_-^{1,0}$	−24.530274	−24.616276	−24.676314	−24.714357	−24.734157	−24.738779	−24.730654
$\sigma^{2,1}\pi_+^{1,0}\pi_-^{1,0}$	−24.452732	−24.587826	−24.695211	−24.779141	−24.843700	−24.892211	−24.927279
$\sigma^{3,1}\pi_-^{1,0}$	−24.451203	−24.639283	−24.805076	−24.951769	−25.082488	−25.199829	−25.305863
$\sigma^{2,1}\pi_-^{1,0}\delta_-^{1,0}$	−24.177175	−24.407259	−24.590156	−24.751640	−24.896522	−25.027508	−25.146661
$\sigma^{2,1}\pi_-^{2,0}$	−24.206010	−24.399251	−24.537159	−24.671338	−24.793645	−24.902943	−25.001144

Table S175: Total energies in E_h for the B atom in the 6-311++G(3df,3pd) basis set in fully uncontracted form, employing the real-orbital approximation.

	$0.00B_0$	$0.10B_0$	$0.20B_0$	$0.30B_0$	$0.40B_0$	$0.50B_0$	$0.60B_0$
$\sigma^{2,2}\pi_+^{1,0}$	−24.528366	−24.514306	−24.473990	−24.411169	−24.329703	−24.232979	−24.123596
$\sigma^{2,2}\pi_-^{1,0}$	−24.528366	−24.614306	−24.673990	−24.711169	−24.729703	−24.732979	−24.723596
$\sigma^{2,1}\pi_+^{1,0}\pi_-^{1,0}$	−24.450473	−24.585464	−24.692310	−24.775009	−24.837922	−24.884968	−24.919104
$\sigma^{3,1}\pi_-^{1,0}$	−24.448972	−24.636956	−24.802223	−24.947626	−25.076334	−25.191287	−25.294890
$\sigma^{2,1}\pi_-^{1,0}\delta_-^{1,0}$	−24.068099	−24.342163	−24.565618	−24.742169	−24.878299	−24.984528	−25.076019
$\sigma^{2,1}\pi_-^{2,0}$	−24.195306	−24.394578	−24.512639	−24.644200	−24.765855	−24.859398	−24.931446

Table S176: Total energies in E_h for the B atom in the def2-TZVP basis set in fully uncontracted form, employing the real-orbital approximation.

	$0.00B_0$	$0.10B_0$	$0.20B_0$	$0.30B_0$	$0.40B_0$	$0.50B_0$	$0.60B_0$
$\sigma^{2,2}\pi_+^{1,0}$	−24.529794	−24.515776	−24.475558	−24.413140	−24.332377	−24.236238	−24.127071
$\sigma^{2,2}\pi_-^{1,0}$	−24.529794	−24.615776	−24.675558	−24.713140	−24.732377	−24.736238	−24.727071
$\sigma^{2,1}\pi_+^{1,0}\pi_-^{1,0}$	−24.452197	−24.587265	−24.694413	−24.777884	−24.841737	−24.889072	−24.922412
$\sigma^{3,1}\pi_-^{1,0}$	−24.450715	−24.638746	−24.804207	−24.950149	−25.079660	−25.195254	−25.298988
$\sigma^{2,1}\pi_-^{1,0}\delta_-^{1,0}$	−23.904753	−24.187015	−24.434678	−24.649836	−24.834944	−24.992492	−25.125089
$\sigma^{2,1}\pi_-^{2,0}$	−24.143044	−24.361428	−24.518406	−24.620311	−24.680564	−24.819356	−24.933357

Table S177: Total energies in E_h for the C atom in the cc-pVDZ basis set in fully uncontracted form, employing the real-orbital approximation.

	$0.00B_0$	$0.10B_0$	$0.20B_0$	$0.30B_0$	$0.40B_0$	$0.50B_0$	$0.60B_0$
$\sigma^{2,2}\pi_+^{1,0}\pi_-^{1,0}$	−37.686625	−37.774174	−37.837260	−37.877142	−37.895736	−37.895375	−37.878530
$\sigma^{3,2}\pi_+^{1,0}$	−37.684267	−37.723609	−37.741954	−37.740219	−37.719811	−37.682465	−37.630076
$\sigma^{3,2}\pi_-^{1,0}$	−37.684267	−37.823609	−37.941954	−38.040219	−38.119811	−38.182465	−38.230076
$\sigma^{3,1}\pi_+^{1,0}\pi_-^{1,0}$	−37.591238	−37.779981	−37.946611	−38.092278	−38.218727	−38.328069	−38.422524
$\sigma^{3,1}\pi_-^{1,0}\delta_-^{1,0}$	−36.279217	−36.618387	−36.936141	−37.233183	−37.510571	−37.769584	−38.011569
$\sigma^{3,1}\pi_-^{1,0}\phi_-^{1,0}$							

Table S178: Total energies in E_h for the C atom in the cc-pVTZ basis set in fully uncontracted form, employing the real-orbital approximation.

	$0.00B_0$	$0.10B_0$	$0.20B_0$	$0.30B_0$	$0.40B_0$	$0.50B_0$	$0.60B_0$
$\sigma^{2,2}\pi_+^{1,0}\pi_-^{1,0}$	−37.691622	−37.779056	−37.842016	−37.882297	−37.902400	−37.904999	−37.892520
$\sigma^{3,2}\pi_+^{1,0}$	−37.688887	−37.728130	−37.746343	−37.744863	−37.725585	−37.690604	−37.641926
$\sigma^{3,2}\pi_-^{1,0}$	−37.688887	−37.828130	−37.946343	−38.044863	−38.125585	−38.190604	−38.241926
$\sigma^{3,1}\pi_+^{1,0}\pi_-^{1,0}$	−37.596857	−37.785614	−37.952445	−38.098886	−38.227066	−38.339250	−38.437495
$\sigma^{3,1}\pi_-^{1,0}\delta_-^{1,0}$	−36.723228	−37.060392	−37.372187	−37.659447	−37.923354	−38.165231	−38.386382
$\sigma^{3,1}\pi_-^{1,0}\phi_-^{1,0}$	−34.716336	−35.105604	−35.473769	−35.821820	−36.151111	−36.463077	−36.759019

Table S179: Total energies in E_h for the C atom in the cc-pVQZ basis set in fully uncontracted form, employing the real-orbital approximation.

	$0.00B_0$	$0.10B_0$	$0.20B_0$	$0.30B_0$	$0.40B_0$	$0.50B_0$	$0.60B_0$
$\sigma^{2,2}\pi_+^{1,0}\pi_-^{1,0}$	−37.693313	−37.780713	−37.843708	−37.884375	−37.905350	−37.909167	−37.897930
$\sigma^{3,2}\pi_+^{1,0}$	−37.690523	−37.729740	−37.747983	−37.746821	−37.728307	−37.694517	−37.647278
$\sigma^{3,2}\pi_-^{1,0}$	−37.690523	−37.829740	−37.947983	−38.046821	−38.128307	−38.194517	−38.247278
$\sigma^{3,1}\pi_+^{1,0}\pi_-^{1,0}$	−37.598759	−37.787530	−37.954502	−38.101396	−38.230414	−38.343694	−38.443061
$\sigma^{3,1}\pi_-^{1,0}\delta_-^{1,0}$	−36.892703	−37.227597	−37.532647	−37.808863	−38.057710	−38.280939	−38.480524
$\sigma^{3,1}\pi_-^{1,0}\phi_-^{1,0}$	−35.690372	−36.078124	−36.441706	−36.781973	−37.100047	−37.397077	−37.674118

Table S180: Total energies in E_h for the C atom in the cc-pV5Z basis set in fully uncontracted form, employing the real-orbital approximation.

	$0.00B_0$	$0.10B_0$	$0.20B_0$	$0.30B_0$	$0.40B_0$	$0.50B_0$	$0.60B_0$
$\sigma^{2,2}\pi_+^{1,0}\pi_-^{1,0}$	−37.693677	−37.781069	−37.844144	−37.885081	−37.906418	−37.910513	−37.899422
$\sigma^{3,2}\pi_+^{1,0}$	−37.690876	−37.730086	−37.748392	−37.747474	−37.729372	−37.696063	−37.649304
$\sigma^{3,2}\pi_-^{1,0}$	−37.690876	−37.830086	−37.948392	−38.047474	−38.129372	−38.196063	−38.249304
$\sigma^{3,1}\pi_+^{1,0}\pi_-^{1,0}$	−37.599202	−37.787984	−37.955063	−38.102231	−38.231626	−38.345290	−38.445049
$\sigma^{3,1}\pi_-^{1,0}\delta_-^{1,0}$	−36.961955	−37.295077	−37.594916	−37.862814	−38.100835	−38.311708	−38.498839
$\sigma^{3,1}\pi_-^{1,0}\phi_-^{1,0}$	−36.177597	−36.563152	−36.920117	−37.249280	−37.551697	−37.828532	−38.080970

Table S181: Total energies in E_h for the C atom in the aug-cc-pVDZ basis set in fully uncontracted form, employing the real-orbital approximation.

	$0.00B_0$	$0.10B_0$	$0.20B_0$	$0.30B_0$	$0.40B_0$	$0.50B_0$	$0.60B_0$
$\sigma^{2,2}\pi_+^{1,0}\pi_-^{1,0}$	−37.687845	−37.775142	−37.837884	−37.878004	−37.897744	−37.899422	−37.885325
$\sigma^{3,2}\pi_+^{1,0}$	−37.685261	−37.724390	−37.742415	−37.740821	−37.721356	−37.685817	−37.635968
$\sigma^{3,2}\pi_-^{1,0}$	−37.685261	−37.824390	−37.942415	−38.040821	−38.121356	−38.185817	−38.235968
$\sigma^{3,1}\pi_+^{1,0}\pi_-^{1,0}$	−37.591521	−37.780179	−37.946783	−38.092831	−38.220168	−38.330784	−38.426693
$\sigma^{3,1}\pi_-^{1,0}\delta_-^{1,0}$	−37.012580	−37.343869	−37.638161	−37.896697	−38.121510	−38.315543	−38.482891
$\sigma^{3,1}\pi_-^{1,0}\phi_-^{1,0}$							

Table S182: Total energies in E_h for the C atom in the aug-cc-pVTZ basis set in fully uncontracted form, employing the real-orbital approximation.

	$0.00B_0$	$0.10B_0$	$0.20B_0$	$0.30B_0$	$0.40B_0$	$0.50B_0$	$0.60B_0$
$\sigma^{2,2}\pi_+^{1,0}\pi_-^{1,0}$	−37.691835	−37.779190	−37.842160	−37.882866	−37.903836	−37.907577	−37.896308
$\sigma^{3,2}\pi_+^{1,0}$	−37.689072	−37.728256	−37.746501	−37.745464	−37.727157	−37.693604	−37.646660
$\sigma^{3,2}\pi_-^{1,0}$	−37.689072	−37.828256	−37.946501	−38.045464	−38.127157	−38.193604	−38.246660
$\sigma^{3,1}\pi_+^{1,0}\pi_-^{1,0}$	−37.596895	−37.785641	−37.952601	−38.099531	−38.228618	−38.342083	−38.441914
$\sigma^{3,1}\pi_-^{1,0}\delta_-^{1,0}$	−37.105060	−37.430250	−37.707101	−37.939834	−38.136758	−38.311009	−38.476671
$\sigma^{3,1}\pi_-^{1,0}\phi_-^{1,0}$	−36.408369	−36.792136	−37.143724	−37.463907	−37.753767	−38.014535	−38.247499

Table S183: Total energies in E_h for the C atom in the aug-cc-pVQZ basis set in fully uncontracted form, employing the real-orbital approximation.

	$0.00B_0$	$0.10B_0$	$0.20B_0$	$0.30B_0$	$0.40B_0$	$0.50B_0$	$0.60B_0$
$\sigma^{2,2}\pi_+^{1,0}\pi_-^{1,0}$	−37.693358	−37.780736	−37.843807	−37.884771	−37.906192	−37.910467	−37.899608
$\sigma^{3,2}\pi_+^{1,0}$	−37.690565	−37.729766	−37.748096	−37.747283	−37.729386	−37.696384	−37.649992
$\sigma^{3,2}\pi_-^{1,0}$	−37.690565	−37.829766	−37.948096	−38.047283	−38.129386	−38.196384	−38.249992
$\sigma^{3,1}\pi_+^{1,0}\pi_-^{1,0}$	−37.598764	−37.787540	−37.954629	−38.101868	−38.231461	−38.345496	−38.445731
$\sigma^{3,1}\pi_-^{1,0}\delta_-^{1,0}$	−37.144994	−37.464491	−37.726250	−37.941811	−38.132370	−38.316507	−38.496168
$\sigma^{3,1}\pi_-^{1,0}\phi_-^{1,0}$	−36.685479	−37.064807	−37.403152	−37.701536	−37.961543	−38.185292	−38.375572

Table S184: Total energies in E_h for the C atom in the aug-cc-pV5Z basis set in fully uncontracted form, employing the real-orbital approximation.

	$0.00B_0$	$0.10B_0$	$0.20B_0$	$0.30B_0$	$0.40B_0$	$0.50B_0$	$0.60B_0$
$\sigma^{2,2}\pi_+^{1,0}\pi_-^{1,0}$	−37.693690	−37.781080	−37.844206	−37.885289	−37.906822	−37.911102	−37.900141
$\sigma^{3,2}\pi_+^{1,0}$	−37.690887	−37.730097	−37.748473	−37.747767	−37.730000	−37.697073	−37.650670
$\sigma^{3,2}\pi_-^{1,0}$	−37.690887	−37.830097	−37.948473	−38.047767	−38.130000	−38.197073	−38.250670
$\sigma^{3,1}\pi_+^{1,0}\pi_-^{1,0}$	−37.599202	−37.787992	−37.955138	−38.102492	−38.232189	−38.346226	−38.446361
$\sigma^{3,1}\pi_-^{1,0}\delta_-^{1,0}$	−37.156434	−37.473317	−37.729148	−37.941656	−38.136604	−38.325496	−38.505177
$\sigma^{3,1}\pi_-^{1,0}\phi_-^{1,0}$	−36.846656	−37.220633	−37.543187	−37.816231	−38.043228	−38.229861	−38.385076

Table S185: Total energies in E_h for the C atom in the HGBSP1-5 basis set in fully uncontracted form, employing the real-orbital approximation.

	$0.00B_0$	$0.10B_0$	$0.20B_0$	$0.30B_0$	$0.40B_0$	$0.50B_0$	$0.60B_0$
$\sigma^{2,2}\pi_+^{1,0}\pi_-^{1,0}$	−37.693030	−37.780424	−37.843512	−37.884410	−37.905549	−37.909219	−37.897419
$\sigma^{3,2}\pi_+^{1,0}$	−37.690233	−37.729431	−37.747703	−37.746657	−37.728187	−37.694130	−37.646147
$\sigma^{3,2}\pi_-^{1,0}$	−37.690233	−37.829431	−37.947703	−38.046657	−38.128187	−38.194130	−38.246147
$\sigma^{3,1}\pi_+^{1,0}\pi_-^{1,0}$	−37.598554	−37.787334	−37.954368	−38.101323	−38.230184	−38.342879	−38.441126
$\sigma^{3,1}\pi_-^{1,0}\delta_-^{1,0}$	−37.217633	−37.497192	−37.729537	−37.942802	−38.140955	−38.325001	−38.497898
$\sigma^{3,1}\pi_-^{1,0}\phi_-^{1,0}$							

Table S186: Total energies in E_h for the C atom in the HGBSP1-7 basis set in fully uncontracted form, employing the real-orbital approximation.

	$0.00B_0$	$0.10B_0$	$0.20B_0$	$0.30B_0$	$0.40B_0$	$0.50B_0$	$0.60B_0$
$\sigma^{2,2}\pi_+^{1,0}\pi_-^{1,0}$	−37.693718	−37.781114	−37.844213	−37.885140	−37.906326	−37.910063	−37.898360
$\sigma^{3,2}\pi_+^{1,0}$	−37.690921	−37.730121	−37.748402	−37.747380	−37.728956	−37.694973	−37.647088
$\sigma^{3,2}\pi_-^{1,0}$	−37.690921	−37.830121	−37.948402	−38.047380	−38.128956	−38.194973	−38.247088
$\sigma^{3,1}\pi_+^{1,0}\pi_-^{1,0}$	−37.599247	−37.788030	−37.955075	−38.102053	−38.230944	−38.343670	−38.441958
$\sigma^{3,1}\pi_-^{1,0}\delta_-^{1,0}$	−37.218353	−37.498017	−37.730608	−37.943738	−38.141768	−38.326782	−38.500136
$\sigma^{3,1}\pi_-^{1,0}\phi_-^{1,0}$							

Table S187: Total energies in E_h for the C atom in the HGBSP1-9 basis set in fully uncontracted form, employing the real-orbital approximation.

	$0.00B_0$	$0.10B_0$	$0.20B_0$	$0.30B_0$	$0.40B_0$	$0.50B_0$	$0.60B_0$
$\sigma^{2,2}\pi_+^{1,0}\pi_-^{1,0}$	−37.693726	−37.781123	−37.844222	−37.885150	−37.906337	−37.910075	−37.898376
$\sigma^{3,2}\pi_+^{1,0}$	−37.690930	−37.730130	−37.748411	−37.747389	−37.728967	−37.694985	−37.647104
$\sigma^{3,2}\pi_-^{1,0}$	−37.690930	−37.830130	−37.948411	−38.047389	−38.128967	−38.194985	−38.247104
$\sigma^{3,1}\pi_+^{1,0}\pi_-^{1,0}$	−37.599255	−37.788038	−37.955083	−38.102062	−38.230955	−38.343682	−38.441975
$\sigma^{3,1}\pi_-^{1,0}\delta_-^{1,0}$	−37.218363	−37.498045	−37.730669	−37.943748	−38.141878	−38.326947	−38.500162
$\sigma^{3,1}\pi_-^{1,0}\phi_-^{1,0}$							

Table S188: Total energies in E_h for the C atom in the HGBSP2-5 basis set in fully uncontracted form, employing the real-orbital approximation.

	$0.00B_0$	$0.10B_0$	$0.20B_0$	$0.30B_0$	$0.40B_0$	$0.50B_0$	$0.60B_0$
$\sigma^{2,2}\pi_+^{1,0}\pi_-^{1,0}$	−37.693043	−37.780433	−37.843559	−37.884625	−37.906118	−37.910351	−37.899330
$\sigma^{3,2}\pi_+^{1,0}$	−37.690237	−37.729448	−37.747828	−37.747117	−37.729317	−37.696329	−37.649838
$\sigma^{3,2}\pi_-^{1,0}$	−37.690237	−37.829448	−37.947828	−38.047117	−38.129317	−38.196329	−38.249838
$\sigma^{3,1}\pi_+^{1,0}\pi_-^{1,0}$	−37.598554	−37.787343	−37.954487	−38.101826	−38.231496	−38.345524	−38.445681
$\sigma^{3,1}\pi_-^{1,0}\delta_-^{1,0}$	−37.217636	−37.497211	−37.729627	−37.943088	−38.141650	−38.326413	−38.500403
$\sigma^{3,1}\pi_-^{1,0}\phi_-^{1,0}$	−37.192164	−37.464776	−37.682654	−37.876787	−38.059980	−38.229621	−38.384397

Table S189: Total energies in E_h for the C atom in the HGBSP2-7 basis set in fully uncontracted form, employing the real-orbital approximation.

	$0.00B_0$	$0.10B_0$	$0.20B_0$	$0.30B_0$	$0.40B_0$	$0.50B_0$	$0.60B_0$
$\sigma^{2,2}\pi_+^{1,0}\pi_-^{1,0}$	−37.693732	−37.781123	−37.844260	−37.885353	−37.906892	−37.911193	−37.900273
$\sigma^{3,2}\pi_+^{1,0}$	−37.690925	−37.730138	−37.748527	−37.747839	−37.730083	−37.697165	−37.650771
$\sigma^{3,2}\pi_-^{1,0}$	−37.690925	−37.830138	−37.948527	−38.047839	−38.130083	−38.197165	−38.250771
$\sigma^{3,1}\pi_+^{1,0}\pi_-^{1,0}$	−37.599247	−37.788038	−37.955194	−38.102554	−38.232253	−38.346310	−38.446510
$\sigma^{3,1}\pi_-^{1,0}\delta_-^{1,0}$	−37.218356	−37.498035	−37.730699	−37.944023	−38.142464	−38.328194	−38.502637
$\sigma^{3,1}\pi_-^{1,0}\phi_-^{1,0}$	−37.192884	−37.465822	−37.683320	−37.880045	−38.061476	−38.230243	−38.388763

Table S190: Total energies in E_h for the C atom in the HGBSP2-9 basis set in fully uncontracted form, employing the real-orbital approximation.

	$0.00B_0$	$0.10B_0$	$0.20B_0$	$0.30B_0$	$0.40B_0$	$0.50B_0$	$0.60B_0$
$\sigma^{2,2}\pi_+^{1,0}\pi_-^{1,0}$	−37.693740	−37.781132	−37.844269	−37.885363	−37.906903	−37.911204	−37.900290
$\sigma^{3,2}\pi_+^{1,0}$	−37.690933	−37.730147	−37.748536	−37.747848	−37.730093	−37.697176	−37.650786
$\sigma^{3,2}\pi_-^{1,0}$	−37.690933	−37.830147	−37.948536	−38.047848	−38.130093	−38.197176	−38.250786
$\sigma^{3,1}\pi_+^{1,0}\pi_-^{1,0}$	−37.599255	−37.788047	−37.955202	−38.102563	−38.232263	−38.346322	−38.446526
$\sigma^{3,1}\pi_-^{1,0}\delta_-^{1,0}$	−37.218365	−37.498064	−37.730759	−37.944033	−38.142574	−38.328359	−38.502662
$\sigma^{3,1}\pi_-^{1,0}\phi_-^{1,0}$	−37.192893	−37.465866	−37.683407	−37.880109	−38.061692	−38.230709	−38.388867

Table S191: Total energies in E_h for the C atom in the HGBSP3-5 basis set in fully uncontracted form, employing the real-orbital approximation.

	$0.00B_0$	$0.10B_0$	$0.20B_0$	$0.30B_0$	$0.40B_0$	$0.50B_0$	$0.60B_0$
$\sigma^{2,2}\pi_+^{1,0}\pi_-^{1,0}$	−37.693044	−37.780433	−37.843561	−37.884630	−37.906132	−37.910390	−37.899423
$\sigma^{3,2}\pi_+^{1,0}$	−37.690237	−37.729448	−37.747828	−37.747118	−37.729323	−37.696350	−37.649895
$\sigma^{3,2}\pi_-^{1,0}$	−37.690237	−37.829448	−37.947828	−38.047118	−38.129323	−38.196350	−38.249895
$\sigma^{3,1}\pi_+^{1,0}\pi_-^{1,0}$	−37.598554	−37.787343	−37.954487	−38.101826	−38.231497	−38.345529	−38.445695
$\sigma^{3,1}\pi_-^{1,0}\delta_-^{1,0}$	−37.217637	−37.498138	−37.732472	−37.947843	−38.148506	−38.335643	−38.511836
$\sigma^{3,1}\pi_-^{1,0}\phi_-^{1,0}$	−37.180845	−37.464776	−37.682654	−37.876788	−38.059983	−38.229628	−38.384416

Table S192: Total energies in E_h for the C atom in the HGBSP3-7 basis set in fully uncontracted form, employing the real-orbital approximation.

	$0.00B_0$	$0.10B_0$	$0.20B_0$	$0.30B_0$	$0.40B_0$	$0.50B_0$	$0.60B_0$
$\sigma^{2,2}\pi_+^{1,0}\pi_-^{1,0}$	−37.693732	−37.781124	−37.844262	−37.885358	−37.906907	−37.911233	−37.900366
$\sigma^{3,2}\pi_+^{1,0}$	−37.690925	−37.730138	−37.748527	−37.747840	−37.730089	−37.697187	−37.650828
$\sigma^{3,2}\pi_-^{1,0}$	−37.690925	−37.830138	−37.948527	−38.047840	−38.130089	−38.197187	−38.250828
$\sigma^{3,1}\pi_+^{1,0}\pi_-^{1,0}$	−37.599247	−37.788038	−37.955194	−38.102554	−38.232254	−38.346315	−38.446523
$\sigma^{3,1}\pi_-^{1,0}\delta_-^{1,0}$	−37.218357	−37.498957	−37.733488	−37.948790	−38.149338	−38.337193	−38.513795
$\sigma^{3,1}\pi_-^{1,0}\phi_-^{1,0}$	−37.181564	−37.465822	−37.683320	−37.880045	−38.061479	−38.230250	−38.388781

Table S193: Total energies in E_h for the C atom in the HGBSP3-9 basis set in fully uncontracted form, employing the real-orbital approximation.

	$0.00B_0$	$0.10B_0$	$0.20B_0$	$0.30B_0$	$0.40B_0$	$0.50B_0$	$0.60B_0$
$\sigma^{2,2}\pi_+^{1,0}\pi_-^{1,0}$	−37.693740	−37.781132	−37.844271	−37.885368	−37.906917	−37.911244	−37.900382
$\sigma^{3,2}\pi_+^{1,0}$	−37.690933	−37.730147	−37.748536	−37.747849	−37.730099	−37.697198	−37.650844
$\sigma^{3,2}\pi_-^{1,0}$	−37.690933	−37.830147	−37.948536	−38.047849	−38.130099	−38.197198	−38.250844
$\sigma^{3,1}\pi_+^{1,0}\pi_-^{1,0}$	−37.599255	−37.788047	−37.955202	−38.102564	−38.232264	−38.346326	−38.446540
$\sigma^{3,1}\pi_-^{1,0}\delta_-^{1,0}$	−37.218366	−37.498982	−37.733535	−37.948810	−38.149410	−38.337323	−38.513850
$\sigma^{3,1}\pi_-^{1,0}\phi_-^{1,0}$	−37.181574	−37.465866	−37.683407	−37.880110	−38.061694	−38.230716	−38.388885

Table S194: Total energies in E_h for the C atom in the AHGBSP1-5 basis set in fully uncontracted form, employing the real-orbital approximation.

	$0.00B_0$	$0.10B_0$	$0.20B_0$	$0.30B_0$	$0.40B_0$	$0.50B_0$	$0.60B_0$
$\sigma^{2,2}\pi_+^{1,0}\pi_-^{1,0}$	−37.693033	−37.780427	−37.843517	−37.884420	−37.905569	−37.909254	−37.897472
$\sigma^{3,2}\pi_+^{1,0}$	−37.690236	−37.729434	−37.747708	−37.746665	−37.728205	−37.694164	−37.646201
$\sigma^{3,2}\pi_-^{1,0}$	−37.690236	−37.829434	−37.947708	−38.046665	−38.128205	−38.194164	−38.246201
$\sigma^{3,1}\pi_+^{1,0}\pi_-^{1,0}$	−37.598558	−37.787339	−37.954375	−38.101335	−38.230203	−38.342905	−38.441159
$\sigma^{3,1}\pi_-^{1,0}\delta_-^{1,0}$	−37.217642	−37.497207	−37.729551	−37.942812	−38.140965	−38.325021	−38.497923
$\sigma^{3,1}\pi_-^{1,0}\phi_-^{1,0}$							

Table S195: Total energies in E_h for the C atom in the AHGBSP1-7 basis set in fully uncontracted form, employing the real-orbital approximation.

	$0.00B_0$	$0.10B_0$	$0.20B_0$	$0.30B_0$	$0.40B_0$	$0.50B_0$	$0.60B_0$
$\sigma^{2,2}\pi_+^{1,0}\pi_-^{1,0}$	−37.693718	−37.781114	−37.844214	−37.885140	−37.906327	−37.910064	−37.898361
$\sigma^{3,2}\pi_+^{1,0}$	−37.690921	−37.730121	−37.748402	−37.747380	−37.728957	−37.694974	−37.647089
$\sigma^{3,2}\pi_-^{1,0}$	−37.690921	−37.830121	−37.948402	−38.047380	−38.128957	−38.194974	−38.247089
$\sigma^{3,1}\pi_+^{1,0}\pi_-^{1,0}$	−37.599247	−37.788030	−37.955075	−38.102053	−38.230944	−38.343671	−38.441960
$\sigma^{3,1}\pi_-^{1,0}\delta_-^{1,0}$	−37.218354	−37.498018	−37.730610	−37.943739	−38.141770	−38.326784	−38.500138
$\sigma^{3,1}\pi_-^{1,0}\phi_-^{1,0}$							

Table S196: Total energies in E_h for the C atom in the AHGBSP1-9 basis set in fully uncontracted form, employing the real-orbital approximation.

	$0.00B_0$	$0.10B_0$	$0.20B_0$	$0.30B_0$	$0.40B_0$	$0.50B_0$	$0.60B_0$
$\sigma^{2,2}\pi_+^{1,0}\pi_-^{1,0}$	−37.693726	−37.781123	−37.844222	−37.885150	−37.906337	−37.910075	−37.898376
$\sigma^{3,2}\pi_+^{1,0}$	−37.690930	−37.730130	−37.748411	−37.747389	−37.728967	−37.694985	−37.647104
$\sigma^{3,2}\pi_-^{1,0}$	−37.690930	−37.830130	−37.948411	−38.047389	−38.128967	−38.194985	−38.247104
$\sigma^{3,1}\pi_+^{1,0}\pi_-^{1,0}$	−37.599255	−37.788038	−37.955083	−38.102062	−38.230955	−38.343682	−38.441975
$\sigma^{3,1}\pi_-^{1,0}\delta_-^{1,0}$	−37.218363	−37.498045	−37.730669	−37.943749	−38.141878	−38.326947	−38.500162
$\sigma^{3,1}\pi_-^{1,0}\phi_-^{1,0}$							

Table S197: Total energies in E_h for the C atom in the AHGBSP2-5 basis set in fully uncontracted form, employing the real-orbital approximation.

	$0.00B_0$	$0.10B_0$	$0.20B_0$	$0.30B_0$	$0.40B_0$	$0.50B_0$	$0.60B_0$
$\sigma^{2,2}\pi_+^{1,0}\pi_-^{1,0}$	−37.693046	−37.780436	−37.843564	−37.884634	−37.906137	−37.910385	−37.899383
$\sigma^{3,2}\pi_+^{1,0}$	−37.690240	−37.729451	−37.747832	−37.747125	−37.729334	−37.696362	−37.649890
$\sigma^{3,2}\pi_-^{1,0}$	−37.690240	−37.829451	−37.947832	−38.047125	−38.129334	−38.196362	−38.249890
$\sigma^{3,1}\pi_+^{1,0}\pi_-^{1,0}$	−37.598558	−37.787347	−37.954494	−38.101837	−38.231514	−38.345549	−38.445712
$\sigma^{3,1}\pi_-^{1,0}\delta_-^{1,0}$	−37.217644	−37.497226	−37.729641	−37.943097	−38.141660	−38.326433	−38.500428
$\sigma^{3,1}\pi_-^{1,0}\phi_-^{1,0}$	−37.192172	−37.464789	−37.682662	−37.876804	−38.059993	−38.229633	−38.384415

Table S198: Total energies in E_h for the C atom in the AHGBSP2-7 basis set in fully uncontracted form, employing the real-orbital approximation.

	$0.00B_0$	$0.10B_0$	$0.20B_0$	$0.30B_0$	$0.40B_0$	$0.50B_0$	$0.60B_0$
$\sigma^{2,2}\pi_+^{1,0}\pi_-^{1,0}$	−37.693732	−37.781123	−37.844261	−37.885354	−37.906893	−37.911194	−37.900274
$\sigma^{3,2}\pi_+^{1,0}$	−37.690925	−37.730138	−37.748527	−37.747839	−37.730084	−37.697165	−37.650772
$\sigma^{3,2}\pi_-^{1,0}$	−37.690925	−37.830138	−37.948527	−38.047839	−38.130084	−38.197165	−38.250772
$\sigma^{3,1}\pi_+^{1,0}\pi_-^{1,0}$	−37.599247	−37.788038	−37.955194	−38.102554	−38.232253	−38.346311	−38.446511
$\sigma^{3,1}\pi_-^{1,0}\delta_-^{1,0}$	−37.218356	−37.498037	−37.730701	−37.944024	−38.142466	−38.328196	−38.502639
$\sigma^{3,1}\pi_-^{1,0}\phi_-^{1,0}$	−37.192884	−37.465824	−37.683322	−37.880046	−38.061478	−38.230246	−38.388764

Table S199: Total energies in E_h for the C atom in the AHGBSP2-9 basis set in fully uncontracted form, employing the real-orbital approximation.

	$0.00B_0$	$0.10B_0$	$0.20B_0$	$0.30B_0$	$0.40B_0$	$0.50B_0$	$0.60B_0$
$\sigma^{2,2}\pi_+^{1,0}\pi_-^{1,0}$	−37.693740	−37.781132	−37.844269	−37.885363	−37.906903	−37.911205	−37.900290
$\sigma^{3,2}\pi_+^{1,0}$	−37.690933	−37.730147	−37.748536	−37.747848	−37.730093	−37.697176	−37.650786
$\sigma^{3,2}\pi_-^{1,0}$	−37.690933	−37.830147	−37.948536	−38.047848	−38.130093	−38.197176	−38.250786
$\sigma^{3,1}\pi_+^{1,0}\pi_-^{1,0}$	−37.599255	−37.788047	−37.955202	−38.102563	−38.232263	−38.346322	−38.446526
$\sigma^{3,1}\pi_-^{1,0}\delta_-^{1,0}$	−37.218365	−37.498064	−37.730759	−37.944034	−38.142574	−38.328359	−38.502663
$\sigma^{3,1}\pi_-^{1,0}\phi_-^{1,0}$	−37.192893	−37.465866	−37.683407	−37.880109	−38.061692	−38.230709	−38.388867

Table S200: Total energies in E_h for the C atom in the AHGBSP3-5 basis set in fully uncontracted form, employing the real-orbital approximation.

	$0.00B_0$	$0.10B_0$	$0.20B_0$	$0.30B_0$	$0.40B_0$	$0.50B_0$	$0.60B_0$
$\sigma^{2,2}\pi_+^{1,0}\pi_-^{1,0}$	−37.693046	−37.780436	−37.843565	−37.884639	−37.906151	−37.910425	−37.899476
$\sigma^{3,2}\pi_+^{1,0}$	−37.690240	−37.729451	−37.747833	−37.747126	−37.729341	−37.696384	−37.649948
$\sigma^{3,2}\pi_-^{1,0}$	−37.690240	−37.829451	−37.947833	−38.047126	−38.129341	−38.196384	−38.249948
$\sigma^{3,1}\pi_+^{1,0}\pi_-^{1,0}$	−37.598558	−37.787347	−37.954494	−38.101837	−38.231516	−38.345554	−38.445726
$\sigma^{3,1}\pi_-^{1,0}\delta_-^{1,0}$	−37.217646	−37.498152	−37.732483	−37.947853	−38.148516	−38.335660	−38.511859
$\sigma^{3,1}\pi_-^{1,0}\phi_-^{1,0}$	−37.192172	−37.464789	−37.682663	−37.876805	−38.059995	−38.229641	−38.384433

Table S201: Total energies in E_h for the C atom in the AHGBSP3-7 basis set in fully uncontracted form, employing the real-orbital approximation.

	$0.00B_0$	$0.10B_0$	$0.20B_0$	$0.30B_0$	$0.40B_0$	$0.50B_0$	$0.60B_0$
$\sigma^{2,2}\pi_+^{1,0}\pi_-^{1,0}$	−37.693732	−37.781124	−37.844262	−37.885359	−37.906907	−37.911233	−37.900367
$\sigma^{3,2}\pi_+^{1,0}$	−37.690925	−37.730138	−37.748527	−37.747840	−37.730090	−37.697187	−37.650829
$\sigma^{3,2}\pi_-^{1,0}$	−37.690925	−37.830138	−37.948527	−38.047840	−38.130090	−38.197187	−38.250829
$\sigma^{3,1}\pi_+^{1,0}\pi_-^{1,0}$	−37.599247	−37.788038	−37.955194	−38.102554	−38.232254	−38.346315	−38.446525
$\sigma^{3,1}\pi_-^{1,0}\delta_-^{1,0}$	−37.218357	−37.498959	−37.733489	−37.948790	−38.149339	−38.337194	−38.513796
$\sigma^{3,1}\pi_-^{1,0}\phi_-^{1,0}$	−37.192884	−37.465824	−37.683322	−37.880047	−38.061480	−38.230253	−38.388783

Table S202: Total energies in E_h for the C atom in the AHGBSP3-9 basis set in fully uncontracted form, employing the real-orbital approximation.

	$0.00B_0$	$0.10B_0$	$0.20B_0$	$0.30B_0$	$0.40B_0$	$0.50B_0$	$0.60B_0$
$\sigma^{2,2}\pi_+^{1,0}\pi_-^{1,0}$	−37.693740	−37.781132	−37.844271	−37.885368	−37.906918	−37.911244	−37.900382
$\sigma^{3,2}\pi_+^{1,0}$	−37.690933	−37.730147	−37.748536	−37.747849	−37.730100	−37.697198	−37.650844
$\sigma^{3,2}\pi_-^{1,0}$	−37.690933	−37.830147	−37.948536	−38.047849	−38.130100	−38.197198	−38.250844
$\sigma^{3,1}\pi_+^{1,0}\pi_-^{1,0}$	−37.599255	−37.788047	−37.955202	−38.102564	−38.232264	−38.346326	−38.446540
$\sigma^{3,1}\pi_-^{1,0}\delta_-^{1,0}$	−37.218366	−37.498982	−37.733535	−37.948810	−38.149411	−38.337323	−38.513850
$\sigma^{3,1}\pi_-^{1,0}\phi_-^{1,0}$	−37.192893	−37.465866	−37.683407	−37.880110	−38.061694	−38.230717	−38.388886

Table S203: Total energies in E_h for the C atom in the 6-311++G(3df,3pd) basis set in fully uncontracted form, employing the real-orbital approximation.

	$0.00B_0$	$0.10B_0$	$0.20B_0$	$0.30B_0$	$0.40B_0$	$0.50B_0$	$0.60B_0$
$\sigma^{2,2}\pi_+^{1,0}\pi_-^{1,0}$	−37.690407	−37.777738	−37.840586	−37.880939	−37.901134	−37.903567	−37.890547
$\sigma^{3,2}\pi_+^{1,0}$	−37.687649	−37.726799	−37.744893	−37.743455	−37.724315	−37.689363	−37.640427
$\sigma^{3,2}\pi_-^{1,0}$	−37.687649	−37.826799	−37.944893	−38.043455	−38.124315	−38.189363	−38.240427
$\sigma^{3,1}\pi_+^{1,0}\pi_-^{1,0}$	−37.595426	−37.784134	−37.950909	−38.097325	−38.225323	−38.336962	−38.434271
$\sigma^{3,1}\pi_-^{1,0}\delta_-^{1,0}$	−37.008868	−37.340609	−37.636221	−37.896814	−38.124149	−38.320661	−38.489623
$\sigma^{3,1}\pi_-^{1,0}\phi_-^{1,0}$	−34.581113	−34.970435	−35.338842	−35.687382	−36.017336	−36.330039	−36.626788

Table S204: Total energies in E_h for the C atom in the def2-TZVP basis set in fully uncontracted form, employing the real-orbital approximation.

	$0.00B_0$	$0.10B_0$	$0.20B_0$	$0.30B_0$	$0.40B_0$	$0.50B_0$	$0.60B_0$
$\sigma^{2,2}\pi_+^{1,0}\pi_-^{1,0}$	−37.692874	−37.780269	−37.843239	−37.883830	−37.904627	−37.908108	−37.896330
$\sigma^{3,2}\pi_+^{1,0}$	−37.690143	−37.729346	−37.747538	−37.746257	−37.727500	−37.693272	−37.645341
$\sigma^{3,2}\pi_-^{1,0}$	−37.690143	−37.829346	−37.947538	−38.046257	−38.127500	−38.193272	−38.245341
$\sigma^{3,1}\pi_+^{1,0}\pi_-^{1,0}$	−37.598459	−37.787229	−37.954183	−38.100994	−38.229795	−38.342655	−38.441335
$\sigma^{3,1}\pi_-^{1,0}\delta_-^{1,0}$	−36.724598	−37.061797	−37.373711	−37.661191	−37.925382	−38.167513	−38.388782
$\sigma^{3,1}\pi_-^{1,0}\phi_-^{1,0}$	−34.717572	−35.106844	−35.475073	−35.823332	−36.152978	−36.465331	−36.761539

Table S205: Total energies in E_h for the N atom in the cc-pVDZ basis set in fully uncontracted form, employing the real-orbital approximation.

	$0.00B_0$	$0.10B_0$	$0.20B_0$	$0.30B_0$	$0.40B_0$	$0.50B_0$	$0.60B_0$
$\sigma^{3,2}\pi_+^{1,0}\pi_-^{1,0}$	−54.391307	−54.531459	−54.652073	−54.753608	−54.836801	−54.902621	−54.952212
$\sigma^{2,3}\pi_+^{1,0}\pi_-^{1,0}$	−54.330049	−54.370107	−54.390442	−54.391525	−54.374107	−54.339177	−54.287902
$\sigma^{3,2}\pi_+^{1,1}$	−54.252036	−54.191845	−54.111433	−54.011279	−53.892155	−53.755082	−53.601280
$\sigma^{3,2}\pi_-^{1,1}$	−54.252036	−54.391845	−54.511433	−54.611279	−54.692155	−54.755082	−54.801280
$\sigma^{3,3}\pi_-^{1,0}$	−54.249820	−54.341055	−54.414877	−54.471632	−54.511878	−54.536354	−54.545942
$\sigma^{3,1}\pi_+^{1,0}\pi_-^{1,0}\delta_-^{1,0}$	−52.367348	−52.707527	−53.028196	−53.329737	−53.612760	−53.878062	−54.126570
$\sigma^{3,1}\pi_+^{1,0}\pi_-^{1,0}\phi_-^{1,0}$							

Table S206: Total energies in E_h for the N atom in the cc-pVTZ basis set in fully uncontracted form, employing the real-orbital approximation.

	$0.00B_0$	$0.10B_0$	$0.20B_0$	$0.30B_0$	$0.40B_0$	$0.50B_0$	$0.60B_0$
$\sigma^{3,2}\pi_+^{1,0}\pi_-^{1,0}$	−54.400805	−54.540836	−54.661170	−54.762503	−54.845902	−54.912682	−54.964277
$\sigma^{2,3}\pi_+^{1,0}\pi_-^{1,0}$	−54.342931	−54.382854	−54.402874	−54.403712	−54.386471	−54.352502	−54.303274
$\sigma^{3,2}\pi_+^{1,1}$	−54.263098	−54.202669	−54.121653	−54.020835	−53.901419	−53.764899	−53.612897
$\sigma^{3,2}\pi_-^{1,1}$	−54.263098	−54.402669	−54.521653	−54.620835	−54.701419	−54.764899	−54.812897
$\sigma^{3,3}\pi_-^{1,0}$	−54.261009	−54.352077	−54.425469	−54.481724	−54.521675	−54.546371	−54.556983
$\sigma^{3,1}\pi_+^{1,0}\pi_-^{1,0}\delta_-^{1,0}$	−53.032096	−53.370948	−53.687673	−53.982759	−54.256948	−54.511153	−54.746365
$\sigma^{3,1}\pi_+^{1,0}\pi_-^{1,0}\phi_-^{1,0}$	−50.139865	−50.529981	−50.900530	−51.252082	−51.585502	−51.901833	−52.202185

Table S207: Total energies in E_h for the N atom in the cc-pVQZ basis set in fully uncontracted form, employing the real-orbital approximation.

	$0.00B_0$	$0.10B_0$	$0.20B_0$	$0.30B_0$	$0.40B_0$	$0.50B_0$	$0.60B_0$
$\sigma^{3,2}\pi_+^{1,0}\pi_-^{1,0}$	−54.403732	−54.543724	−54.664004	−54.765422	−54.849221	−54.916836	−54.969718
$\sigma^{2,3}\pi_+^{1,0}\pi_-^{1,0}$	−54.346437	−54.386311	−54.406250	−54.407146	−54.390293	−54.357171	−54.309266
$\sigma^{3,2}\pi_+^{1,1}$	−54.266490	−54.205948	−54.124692	−54.023749	−53.904607	−53.768960	−53.618472
$\sigma^{3,2}\pi_-^{1,1}$	−54.266490	−54.405948	−54.524692	−54.623749	−54.704607	−54.768960	−54.818472
$\sigma^{3,3}\pi_-^{1,0}$	−54.264483	−54.355478	−54.428702	−54.484834	−54.524882	−54.550048	−54.561598
$\sigma^{3,1}\pi_+^{1,0}\pi_-^{1,0}\delta_-^{1,0}$	−53.283016	−53.620373	−53.932643	−54.220388	−54.484449	−54.725846	−54.945685
$\sigma^{3,1}\pi_+^{1,0}\pi_-^{1,0}\phi_-^{1,0}$	−51.578296	−51.967414	−52.334962	−52.681478	−53.007746	−53.314667	−53.603148

Table S208: Total energies in E_h for the N atom in the cc-pV5Z basis set in fully uncontracted form, employing the real-orbital approximation.

	$0.00B_0$	$0.10B_0$	$0.20B_0$	$0.30B_0$	$0.40B_0$	$0.50B_0$	$0.60B_0$
$\sigma^{3,2}\pi_+^{1,0}\pi_-^{1,0}$	−54.404443	−54.544420	−54.664709	−54.766276	−54.850428	−54.918574	−54.972073
$\sigma^{2,3}\pi_+^{1,0}\pi_-^{1,0}$	−54.347280	−54.387129	−54.407062	−54.408103	−54.391626	−54.359084	−54.311864
$\sigma^{3,2}\pi_+^{1,1}$	−54.267410	−54.206790	−54.125421	−54.024573	−53.905885	−53.770999	−53.621395
$\sigma^{3,2}\pi_-^{1,1}$	−54.267410	−54.406790	−54.525421	−54.624573	−54.705885	−54.770999	−54.821395
$\sigma^{3,3}\pi_-^{1,0}$	−54.265432	−54.356375	−54.429500	−54.485621	−54.525871	−54.551512	−54.563793
$\sigma^{3,1}\pi_+^{1,0}\pi_-^{1,0}\delta_-^{1,0}$	−53.393785	−53.729859	−54.038317	−54.319827	−54.575399	−54.806297	−55.013987
$\sigma^{3,1}\pi_+^{1,0}\pi_-^{1,0}\phi_-^{1,0}$	−52.273715	−52.661357	−53.024464	−53.363539	−53.679296	−53.972550	−54.244152

Table S209: Total energies in E_h for the N atom in the aug-cc-pVDZ basis set in fully uncontracted form, employing the real-orbital approximation.

	$0.00B_0$	$0.10B_0$	$0.20B_0$	$0.30B_0$	$0.40B_0$	$0.50B_0$	$0.60B_0$
$\sigma^{3,2}\pi_+^{1,0}\pi_-^{1,0}$	−54.393362	−54.533260	−54.653303	−54.754372	−54.837581	−54.904114	−54.955175
$\sigma^{2,3}\pi_+^{1,0}\pi_-^{1,0}$	−54.333693	−54.373465	−54.393162	−54.393720	−54.376289	−54.342066	−54.292262
$\sigma^{3,2}\pi_+^{1,1}$	−54.256233	−54.195502	−54.113858	−54.012557	−53.892951	−53.756301	−53.603832
$\sigma^{3,2}\pi_-^{1,1}$	−54.256233	−54.395502	−54.513858	−54.612557	−54.692951	−54.756301	−54.803832
$\sigma^{3,3}\pi_-^{1,0}$	−54.253893	−54.344815	−54.417883	−54.473869	−54.513751	−54.538540	−54.549230
$\sigma^{3,1}\pi_+^{1,0}\pi_-^{1,0}\delta_-^{1,0}$	−53.444407	−53.779575	−54.085281	−54.362094	−54.610887	−54.832804	−55.029246
$\sigma^{3,1}\pi_+^{1,0}\pi_-^{1,0}\phi_-^{1,0}$							

Table S210: Total energies in E_h for the N atom in the aug-cc-pVTZ basis set in fully uncontracted form, employing the real-orbital approximation.

	$0.00B_0$	$0.10B_0$	$0.20B_0$	$0.30B_0$	$0.40B_0$	$0.50B_0$	$0.60B_0$
$\sigma^{3,2}\pi_+^{1,0}\pi_-^{1,0}$	−54.401212	−54.541157	−54.661367	−54.762807	−54.846744	−54.914563	−54.967671
$\sigma^{2,3}\pi_+^{1,0}\pi_-^{1,0}$	−54.343921	−54.383739	−54.403597	−54.404512	−54.387798	−54.354880	−54.307199
$\sigma^{3,2}\pi_+^{1,1}$	−54.264301	−54.203639	−54.122212	−54.021308	−53.902424	−53.767089	−53.616836
$\sigma^{3,2}\pi_-^{1,1}$	−54.264301	−54.403639	−54.522212	−54.621308	−54.702424	−54.767089	−54.816836
$\sigma^{3,3}\pi_-^{1,0}$	−54.262319	−54.353225	−54.426272	−54.482312	−54.522465	−54.547952	−54.560002
$\sigma^{3,1}\pi_+^{1,0}\pi_-^{1,0}\delta_-^{1,0}$	−53.595559	−53.926629	−54.220276	−54.477840	−54.701664	−54.895302	−55.063777
$\sigma^{3,1}\pi_+^{1,0}\pi_-^{1,0}\phi_-^{1,0}$	−52.649191	−53.035363	−53.394053	−53.725732	−54.031094	−54.310967	−54.566248

Table S211: Total energies in E_h for the N atom in the aug-cc-pVQZ basis set in fully uncontracted form, employing the real-orbital approximation.

	$0.00B_0$	$0.10B_0$	$0.20B_0$	$0.30B_0$	$0.40B_0$	$0.50B_0$	$0.60B_0$
$\sigma^{3,2}\pi_+^{1,0}\pi_-^{1,0}$	−54.403837	−54.543799	−54.664077	−54.765681	−54.849938	−54.918270	−54.972060
$\sigma^{2,3}\pi_+^{1,0}\pi_-^{1,0}$	−54.346694	−54.386530	−54.406458	−54.407550	−54.391193	−54.358863	−54.311981
$\sigma^{3,2}\pi_+^{1,1}$	−54.266863	−54.206217	−54.124847	−54.024086	−53.905523	−53.770785	−53.621416
$\sigma^{3,2}\pi_-^{1,1}$	−54.266863	−54.406217	−54.524847	−54.624086	−54.705523	−54.770785	−54.821416
$\sigma^{3,3}\pi_-^{1,0}$	−54.264893	−54.355806	−54.428896	−54.485071	−54.525525	−54.551538	−54.564359
$\sigma^{3,1}\pi_+^{1,0}\pi_-^{1,0}\delta_-^{1,0}$	−53.669064	−53.995640	−54.276382	−54.514604	−54.716718	−54.892908	−55.055291
$\sigma^{3,1}\pi_+^{1,0}\pi_-^{1,0}\phi_-^{1,0}$	−53.063767	−53.446423	−53.794582	−54.108796	−54.389902	−54.638966	−54.857252

Table S212: Total energies in E_h for the N atom in the aug-cc-pV5Z basis set in fully uncontracted form, employing the real-orbital approximation.

	$0.00B_0$	$0.10B_0$	$0.20B_0$	$0.30B_0$	$0.40B_0$	$0.50B_0$	$0.60B_0$
$\sigma^{3,2}\pi_+^{1,0}\pi_-^{1,0}$	−54.404470	−54.544441	−54.664757	−54.766451	−54.850862	−54.919379	−54.973317
$\sigma^{2,3}\pi_+^{1,0}\pi_-^{1,0}$	−54.347342	−54.387187	−54.407157	−54.408352	−54.392178	−54.360074	−54.313384
$\sigma^{3,2}\pi_+^{1,1}$	−54.267467	−54.206829	−54.125493	−54.024834	−53.906492	−53.772061	−53.622971
$\sigma^{3,2}\pi_-^{1,1}$	−54.267467	−54.406829	−54.525493	−54.624834	−54.706492	−54.772061	−54.822971
$\sigma^{3,3}\pi_-^{1,0}$	−54.265504	−54.356423	−54.429545	−54.485810	−54.526437	−54.552692	−54.565772
$\sigma^{3,1}\pi_+^{1,0}\pi_-^{1,0}\delta_-^{1,0}$	−53.694876	−54.018774	−54.292169	−54.520786	−54.715706	−54.892104	−55.061270
$\sigma^{3,1}\pi_+^{1,0}\pi_-^{1,0}\phi_-^{1,0}$	−53.252109	−53.631433	−53.969679	−54.267653	−54.526690	−54.748690	−54.936269

Table S213: Total energies in E_h for the N atom in the HGBSP1-5 basis set in fully uncontracted form, employing the real-orbital approximation.

	$0.00B_0$	$0.10B_0$	$0.20B_0$	$0.30B_0$	$0.40B_0$	$0.50B_0$	$0.60B_0$
$\sigma^{3,2}\pi_+^{1,0}\pi_-^{1,0}$	−54.403540	−54.543507	−54.663781	−54.765313	−54.849341	−54.917176	−54.970092
$\sigma^{2,3}\pi_+^{1,0}\pi_-^{1,0}$	−54.344465	−54.384310	−54.404239	−54.405253	−54.388636	−54.355735	−54.307848
$\sigma^{3,2}\pi_+^{1,1}$	−54.266030	−54.205359	−54.123888	−54.022900	−53.903912	−53.768432	−53.617866
$\sigma^{3,2}\pi_-^{1,1}$	−54.266030	−54.405359	−54.523888	−54.622900	−54.703912	−54.768432	−54.817866
$\sigma^{3,3}\pi_-^{1,0}$	−54.263521	−54.354499	−54.427741	−54.484045	−54.524458	−54.550100	−54.562071
$\sigma^{3,1}\pi_+^{1,0}\pi_-^{1,0}\delta_-^{1,0}$	−53.802559	−54.080442	−54.310548	−54.520173	−54.716046	−54.898948	−55.068425
$\sigma^{3,1}\pi_+^{1,0}\pi_-^{1,0}\phi_-^{1,0}$							

Table S214: Total energies in E_h for the N atom in the HGBSP1-7 basis set in fully uncontracted form, employing the real-orbital approximation.

	$0.00B_0$	$0.10B_0$	$0.20B_0$	$0.30B_0$	$0.40B_0$	$0.50B_0$	$0.60B_0$
$\sigma^{3,2}\pi_+^{1,0}\pi_-^{1,0}$	−54.404536	−54.544506	−54.664785	−54.766329	−54.850379	−54.918251	−54.971217
$\sigma^{2,3}\pi_+^{1,0}\pi_-^{1,0}$	−54.345459	−54.385305	−54.405239	−54.406268	−54.389680	−54.356825	−54.308999
$\sigma^{3,2}\pi_+^{1,1}$	−54.267022	−54.206352	−54.124886	−54.023913	−53.904963	−53.769541	−53.619043
$\sigma^{3,2}\pi_-^{1,1}$	−54.267022	−54.406352	−54.524886	−54.623913	−54.704963	−54.769541	−54.819043
$\sigma^{3,3}\pi_-^{1,0}$	−54.264513	−54.355492	−54.428739	−54.485055	−54.525493	−54.551179	−54.563217
$\sigma^{3,1}\pi_+^{1,0}\pi_-^{1,0}\delta_-^{1,0}$	−53.803594	−54.081607	−54.311589	−54.521882	−54.717559	−54.899946	−55.069956
$\sigma^{3,1}\pi_+^{1,0}\pi_-^{1,0}\phi_-^{1,0}$							

Table S215: Total energies in E_h for the N atom in the HGBSP1-9 basis set in fully uncontracted form, employing the real-orbital approximation.

	$0.00B_0$	$0.10B_0$	$0.20B_0$	$0.30B_0$	$0.40B_0$	$0.50B_0$	$0.60B_0$
$\sigma^{3,2}\pi_+^{1,0}\pi_-^{1,0}$	−54.404548	−54.544518	−54.664797	−54.766342	−54.850394	−54.918266	−54.971233
$\sigma^{2,3}\pi_+^{1,0}\pi_-^{1,0}$	−54.345471	−54.385317	−54.405251	−54.406280	−54.389694	−54.356840	−54.309015
$\sigma^{3,2}\pi_+^{1,1}$	−54.267034	−54.206364	−54.124898	−54.023926	−53.904977	−53.769557	−53.619061
$\sigma^{3,2}\pi_-^{1,1}$	−54.267034	−54.406364	−54.524898	−54.623926	−54.704977	−54.769557	−54.819061
$\sigma^{3,3}\pi_-^{1,0}$	−54.264525	−54.355504	−54.428751	−54.485067	−54.525506	−54.551193	−54.563232
$\sigma^{3,1}\pi_+^{1,0}\pi_-^{1,0}\delta_-^{1,0}$	−53.803607	−54.081622	−54.311604	−54.521999	−54.717591	−54.900003	−55.070188
$\sigma^{3,1}\pi_+^{1,0}\pi_-^{1,0}\phi_-^{1,0}$							

Table S216: Total energies in E_h for the N atom in the HGBSP2-5 basis set in fully uncontracted form, employing the real-orbital approximation.

	$0.00B_0$	$0.10B_0$	$0.20B_0$	$0.30B_0$	$0.40B_0$	$0.50B_0$	$0.60B_0$
$\sigma^{3,2}\pi_+^{1,0}\pi_-^{1,0}$	−54.403540	−54.543511	−54.663832	−54.765536	−54.849947	−54.918446	−54.972351
$\sigma^{2,3}\pi_+^{1,0}\pi_-^{1,0}$	−54.346409	−54.386255	−54.406234	−54.407443	−54.391267	−54.359127	−54.312372
$\sigma^{3,2}\pi_+^{1,1}$	−54.266539	−54.205902	−54.124580	−54.023946	−53.905604	−53.771125	−53.621952
$\sigma^{3,2}\pi_-^{1,1}$	−54.266539	−54.405902	−54.524580	−54.623946	−54.705604	−54.771125	−54.821952
$\sigma^{3,3}\pi_-^{1,0}$	−54.264576	−54.355496	−54.428634	−54.484936	−54.525597	−54.551852	−54.564882
$\sigma^{3,1}\pi_+^{1,0}\pi_-^{1,0}\delta_-^{1,0}$	−53.802560	−54.080452	−54.310597	−54.520330	−54.716437	−54.899759	−55.069908
$\sigma^{3,1}\pi_+^{1,0}\pi_-^{1,0}\phi_-^{1,0}$	−53.777599	−54.049665	−54.264160	−54.459014	−54.633357	−54.797847	−54.952333

Table S217: Total energies in E_h for the N atom in the HGBSP2-7 basis set in fully uncontracted form, employing the real-orbital approximation.

	$0.00B_0$	$0.10B_0$	$0.20B_0$	$0.30B_0$	$0.40B_0$	$0.50B_0$	$0.60B_0$
$\sigma^{3,2}\pi_+^{1,0}\pi_-^{1,0}$	−54.404536	−54.544509	−54.664836	−54.766552	−54.850985	−54.919517	−54.973470
$\sigma^{2,3}\pi_+^{1,0}\pi_-^{1,0}$	−54.347413	−54.387261	−54.407245	−54.408469	−54.392322	−54.360225	−54.313526
$\sigma^{3,2}\pi_+^{1,1}$	−54.267534	−54.206898	−54.125581	−54.024963	−53.906657	−53.772231	−53.623119
$\sigma^{3,2}\pi_-^{1,1}$	−54.267534	−54.406898	−54.525581	−54.624963	−54.706657	−54.772231	−54.823119
$\sigma^{3,3}\pi_-^{1,0}$	−54.265575	−54.356496	−54.429638	−54.485952	−54.526638	−54.552934	−54.566020
$\sigma^{3,1}\pi_+^{1,0}\pi_-^{1,0}\delta_-^{1,0}$	−53.803595	−54.081616	−54.311638	−54.522040	−54.717949	−54.900757	−55.071440
$\sigma^{3,1}\pi_+^{1,0}\pi_-^{1,0}\phi_-^{1,0}$	−53.778635	−54.050765	−54.266427	−54.460130	−54.637826	−54.801899	−54.953230

Table S218: Total energies in E_h for the N atom in the HGBSP2-9 basis set in fully uncontracted form, employing the real-orbital approximation.

	$0.00B_0$	$0.10B_0$	$0.20B_0$	$0.30B_0$	$0.40B_0$	$0.50B_0$	$0.60B_0$
$\sigma^{3,2}\pi_+^{1,0}\pi_-^{1,0}$	−54.404548	−54.544521	−54.664848	−54.766565	−54.850999	−54.919532	−54.973485
$\sigma^{2,3}\pi_+^{1,0}\pi_-^{1,0}$	−54.347425	−54.387273	−54.407257	−54.408482	−54.392335	−54.360239	−54.313541
$\sigma^{3,2}\pi_+^{1,1}$	−54.267546	−54.206910	−54.125593	−54.024975	−53.906670	−53.772246	−53.623136
$\sigma^{3,2}\pi_-^{1,1}$	−54.267546	−54.406910	−54.525593	−54.624975	−54.706670	−54.772246	−54.823136
$\sigma^{3,3}\pi_-^{1,0}$	−54.265587	−54.356508	−54.429650	−54.485965	−54.526651	−54.552948	−54.566035
$\sigma^{3,1}\pi_+^{1,0}\pi_-^{1,0}\delta_-^{1,0}$	−53.803608	−54.081631	−54.311653	−54.522156	−54.717981	−54.900815	−55.071671
$\sigma^{3,1}\pi_+^{1,0}\pi_-^{1,0}\phi_-^{1,0}$	−53.778648	−54.050800	−54.266516	−54.460373	−54.637996	−54.801952	−54.953803

Table S219: Total energies in E_h for the N atom in the HGBSP3-5 basis set in fully uncontracted form, employing the real-orbital approximation.

	$0.00B_0$	$0.10B_0$	$0.20B_0$	$0.30B_0$	$0.40B_0$	$0.50B_0$	$0.60B_0$
$\sigma^{3,2}\pi_+^{1,0}\pi_-^{1,0}$	−54.403540	−54.543511	−54.663832	−54.765536	−54.849948	−54.918450	−54.972364
$\sigma^{2,3}\pi_+^{1,0}\pi_-^{1,0}$	−54.346412	−54.386259	−54.406238	−54.407450	−54.391279	−54.359151	−54.312418
$\sigma^{3,2}\pi_+^{1,1}$	−54.266539	−54.205903	−54.124580	−54.023946	−53.905606	−53.771130	−53.621965
$\sigma^{3,2}\pi_-^{1,1}$	−54.266539	−54.405903	−54.524580	−54.623946	−54.705606	−54.771130	−54.821965
$\sigma^{3,3}\pi_-^{1,0}$	−54.264576	−54.355496	−54.428634	−54.484936	−54.525599	−54.551859	−54.564900
$\sigma^{3,1}\pi_+^{1,0}\pi_-^{1,0}\delta_-^{1,0}$	−53.802560	−54.081516	−54.313721	−54.525804	−54.723927	−54.909318	−55.081859
$\sigma^{3,1}\pi_+^{1,0}\pi_-^{1,0}\phi_-^{1,0}$	−53.766340	−54.049665	−54.264160	−54.459014	−54.633357	−54.797848	−54.952335

Table S220: Total energies in E_h for the N atom in the HGBSP3-7 basis set in fully uncontracted form, employing the real-orbital approximation.

	$0.00B_0$	$0.10B_0$	$0.20B_0$	$0.30B_0$	$0.40B_0$	$0.50B_0$	$0.60B_0$
$\sigma^{3,2}\pi_+^{1,0}\pi_-^{1,0}$	−54.404536	−54.544509	−54.664836	−54.766552	−54.850986	−54.919521	−54.973483
$\sigma^{2,3}\pi_+^{1,0}\pi_-^{1,0}$	−54.347417	−54.387264	−54.407249	−54.408476	−54.392334	−54.360249	−54.313572
$\sigma^{3,2}\pi_+^{1,1}$	−54.267534	−54.206899	−54.125581	−54.024963	−53.906659	−53.772236	−53.623132
$\sigma^{3,2}\pi_-^{1,1}$	−54.267534	−54.406899	−54.525581	−54.624963	−54.706659	−54.772236	−54.823132
$\sigma^{3,3}\pi_-^{1,0}$	−54.265575	−54.356496	−54.429639	−54.485953	−54.526640	−54.552941	−54.566038
$\sigma^{3,1}\pi_+^{1,0}\pi_-^{1,0}\delta_-^{1,0}$	−53.803595	−54.082657	−54.314770	−54.527404	−54.725428	−54.910411	−55.083312
$\sigma^{3,1}\pi_+^{1,0}\pi_-^{1,0}\phi_-^{1,0}$	−53.778635	−54.050765	−54.266427	−54.460130	−54.637826	−54.801900	−54.953232

Table S221: Total energies in E_h for the N atom in the HGBSP3-9 basis set in fully uncontracted form, employing the real-orbital approximation.

	$0.00B_0$	$0.10B_0$	$0.20B_0$	$0.30B_0$	$0.40B_0$	$0.50B_0$	$0.60B_0$
$\sigma^{3,2}\pi_+^{1,0}\pi_-^{1,0}$	−54.404548	−54.544521	−54.664848	−54.766565	−54.851000	−54.919536	−54.973499
$\sigma^{2,3}\pi_+^{1,0}\pi_-^{1,0}$	−54.347429	−54.387276	−54.407261	−54.408489	−54.392347	−54.360264	−54.313587
$\sigma^{3,2}\pi_+^{1,1}$	−54.267546	−54.206910	−54.125593	−54.024976	−53.906672	−53.772251	−53.623149
$\sigma^{3,2}\pi_-^{1,1}$	−54.267546	−54.406910	−54.525593	−54.624976	−54.706672	−54.772251	−54.823149
$\sigma^{3,3}\pi_-^{1,0}$	−54.265587	−54.356508	−54.429651	−54.485965	−54.526653	−54.552954	−54.566052
$\sigma^{3,1}\pi_+^{1,0}\pi_-^{1,0}\delta_-^{1,0}$	−53.803608	−54.082672	−54.314789	−54.527490	−54.725474	−54.910454	−55.083464
$\sigma^{3,1}\pi_+^{1,0}\pi_-^{1,0}\phi_-^{1,0}$	−53.778648	−54.050800	−54.266516	−54.460373	−54.637996	−54.801952	−54.953805

Table S222: Total energies in E_h for the N atom in the AHGBSP1-5 basis set in fully uncontracted form, employing the real-orbital approximation.

	$0.00B_0$	$0.10B_0$	$0.20B_0$	$0.30B_0$	$0.40B_0$	$0.50B_0$	$0.60B_0$
$\sigma^{3,2}\pi_+^{1,0}\pi_-^{1,0}$	−54.403545	−54.543513	−54.663788	−54.765322	−54.849353	−54.917195	−54.970124
$\sigma^{2,3}\pi_+^{1,0}\pi_-^{1,0}$	−54.344469	−54.384314	−54.404243	−54.405259	−54.388647	−54.355756	−54.307885
$\sigma^{3,2}\pi_+^{1,1}$	−54.266033	−54.205362	−54.123892	−54.022905	−53.903923	−53.768454	−53.617904
$\sigma^{3,2}\pi_-^{1,1}$	−54.266033	−54.405362	−54.523892	−54.622905	−54.703923	−54.768454	−54.817904
$\sigma^{3,3}\pi_-^{1,0}$	−54.263524	−54.354502	−54.427745	−54.484051	−54.524469	−54.550119	−54.562106
$\sigma^{3,1}\pi_+^{1,0}\pi_-^{1,0}\delta_-^{1,0}$	−53.802571	−54.080463	−54.310559	−54.520200	−54.716066	−54.898960	−55.068447
$\sigma^{3,1}\pi_+^{1,0}\pi_-^{1,0}\phi_-^{1,0}$							

Table S223: Total energies in E_h for the N atom in the AHGBSP1-7 basis set in fully uncontracted form, employing the real-orbital approximation.

	$0.00B_0$	$0.10B_0$	$0.20B_0$	$0.30B_0$	$0.40B_0$	$0.50B_0$	$0.60B_0$
$\sigma^{3,2}\pi_+^{1,0}\pi_-^{1,0}$	−54.404536	−54.544506	−54.664785	−54.766329	−54.850380	−54.918252	−54.971218
$\sigma^{2,3}\pi_+^{1,0}\pi_-^{1,0}$	−54.345459	−54.385305	−54.405239	−54.406268	−54.389681	−54.356826	−54.308999
$\sigma^{3,2}\pi_+^{1,1}$	−54.267022	−54.206352	−54.124886	−54.023913	−53.904963	−53.769542	−53.619043
$\sigma^{3,2}\pi_-^{1,1}$	−54.267022	−54.406352	−54.524886	−54.623913	−54.704963	−54.769542	−54.819043
$\sigma^{3,3}\pi_-^{1,0}$	−54.264513	−54.355492	−54.428739	−54.485055	−54.525493	−54.551179	−54.563217
$\sigma^{3,1}\pi_+^{1,0}\pi_-^{1,0}\delta_-^{1,0}$	−53.803594	−54.081607	−54.311589	−54.521886	−54.717560	−54.899947	−55.069959
$\sigma^{3,1}\pi_+^{1,0}\pi_-^{1,0}\phi_-^{1,0}$							

Table S224: Total energies in E_h for the N atom in the AHGBSP1-9 basis set in fully uncontracted form, employing the real-orbital approximation.

	$0.00B_0$	$0.10B_0$	$0.20B_0$	$0.30B_0$	$0.40B_0$	$0.50B_0$	$0.60B_0$
$\sigma^{3,2}\pi_+^{1,0}\pi_-^{1,0}$	−54.404548	−54.544518	−54.664797	−54.766342	−54.850394	−54.918266	−54.971233
$\sigma^{2,3}\pi_+^{1,0}\pi_-^{1,0}$	−54.345471	−54.385317	−54.405251	−54.406280	−54.389694	−54.356840	−54.309015
$\sigma^{3,2}\pi_+^{1,1}$	−54.267034	−54.206364	−54.124898	−54.023926	−53.904977	−53.769558	−53.619061
$\sigma^{3,2}\pi_-^{1,1}$	−54.267034	−54.406364	−54.524898	−54.623926	−54.704977	−54.769558	−54.819061
$\sigma^{3,3}\pi_-^{1,0}$	−54.264525	−54.355504	−54.428751	−54.485067	−54.525506	−54.551193	−54.563233
$\sigma^{3,1}\pi_+^{1,0}\pi_-^{1,0}\delta_-^{1,0}$	−53.803607	−54.081622	−54.311604	−54.521999	−54.717591	−54.900004	−55.070188
$\sigma^{3,1}\pi_+^{1,0}\pi_-^{1,0}\phi_-^{1,0}$							

Table S225: Total energies in E_h for the N atom in the AHGBSP2-5 basis set in fully uncontracted form, employing the real-orbital approximation.

	$0.00B_0$	$0.10B_0$	$0.20B_0$	$0.30B_0$	$0.40B_0$	$0.50B_0$	$0.60B_0$
$\sigma^{3,2}\pi_+^{1,0}\pi_-^{1,0}$	−54.403545	−54.543517	−54.663839	−54.765545	−54.849959	−54.918464	−54.972383
$\sigma^{2,3}\pi_+^{1,0}\pi_-^{1,0}$	−54.346412	−54.386259	−54.406238	−54.407449	−54.391278	−54.359148	−54.312409
$\sigma^{3,2}\pi_+^{1,1}$	−54.266542	−54.205906	−54.124584	−54.023951	−53.905615	−53.771146	−53.621990
$\sigma^{3,2}\pi_-^{1,1}$	−54.266542	−54.405906	−54.524584	−54.623951	−54.705615	−54.771146	−54.821990
$\sigma^{3,3}\pi_-^{1,0}$	−54.264579	−54.355499	−54.428638	−54.484942	−54.525607	−54.551871	−54.564916
$\sigma^{3,1}\pi_+^{1,0}\pi_-^{1,0}\delta_-^{1,0}$	−53.802572	−54.080472	−54.310608	−54.520357	−54.716456	−54.899771	−55.069929
$\sigma^{3,1}\pi_+^{1,0}\pi_-^{1,0}\phi_-^{1,0}$	−53.777611	−54.049681	−54.264188	−54.459028	−54.633385	−54.797871	−54.952351

Table S226: Total energies in E_h for the N atom in the AHGBSP2-7 basis set in fully uncontracted form, employing the real-orbital approximation.

	$0.00B_0$	$0.10B_0$	$0.20B_0$	$0.30B_0$	$0.40B_0$	$0.50B_0$	$0.60B_0$
$\sigma^{3,2}\pi_+^{1,0}\pi_-^{1,0}$	−54.404536	−54.544509	−54.664836	−54.766552	−54.850985	−54.919518	−54.973471
$\sigma^{2,3}\pi_+^{1,0}\pi_-^{1,0}$	−54.347413	−54.387261	−54.407245	−54.408470	−54.392322	−54.360225	−54.313526
$\sigma^{3,2}\pi_+^{1,1}$	−54.267534	−54.206898	−54.125581	−54.024963	−53.906657	−53.772231	−53.623119
$\sigma^{3,2}\pi_-^{1,1}$	−54.267534	−54.406898	−54.525581	−54.624963	−54.706657	−54.772231	−54.823119
$\sigma^{3,3}\pi_-^{1,0}$	−54.265575	−54.356496	−54.429638	−54.485953	−54.526638	−54.552934	−54.566020
$\sigma^{3,1}\pi_+^{1,0}\pi_-^{1,0}\delta_-^{1,0}$	−53.803595	−54.081616	−54.311638	−54.522043	−54.717950	−54.900758	−55.071443
$\sigma^{3,1}\pi_+^{1,0}\pi_-^{1,0}\phi_-^{1,0}$	−53.778635	−54.050767	−54.266429	−54.460133	−54.637828	−54.801900	−54.953234

Table S227: Total energies in E_h for the N atom in the AHGBSP2-9 basis set in fully uncontracted form, employing the real-orbital approximation.

	$0.00B_0$	$0.10B_0$	$0.20B_0$	$0.30B_0$	$0.40B_0$	$0.50B_0$	$0.60B_0$
$\sigma^{3,2}\pi_+^{1,0}\pi_-^{1,0}$	−54.404548	−54.544521	−54.664848	−54.766565	−54.850999	−54.919532	−54.973485
$\sigma^{2,3}\pi_+^{1,0}\pi_-^{1,0}$	−54.347425	−54.387273	−54.407257	−54.408482	−54.392335	−54.360239	−54.313541
$\sigma^{3,2}\pi_+^{1,1}$	−54.267546	−54.206910	−54.125593	−54.024975	−53.906670	−53.772246	−53.623136
$\sigma^{3,2}\pi_-^{1,1}$	−54.267546	−54.406910	−54.525593	−54.624975	−54.706670	−54.772246	−54.823136
$\sigma^{3,3}\pi_-^{1,0}$	−54.265587	−54.356508	−54.429650	−54.485965	−54.526651	−54.552948	−54.566035
$\sigma^{3,1}\pi_+^{1,0}\pi_-^{1,0}\delta_-^{1,0}$	−53.803608	−54.081631	−54.311653	−54.522156	−54.717981	−54.900815	−55.071672
$\sigma^{3,1}\pi_+^{1,0}\pi_-^{1,0}\phi_-^{1,0}$	−53.778648	−54.050800	−54.266516	−54.460373	−54.637996	−54.801952	−54.953803

Table S228: Total energies in E_h for the N atom in the AHGBSP3-5 basis set in fully uncontracted form, employing the real-orbital approximation.

	$0.00B_0$	$0.10B_0$	$0.20B_0$	$0.30B_0$	$0.40B_0$	$0.50B_0$	$0.60B_0$
$\sigma^{3,2}\pi_+^{1,0}\pi_-^{1,0}$	−54.403545	−54.543517	−54.663839	−54.765545	−54.849960	−54.918469	−54.972396
$\sigma^{2,3}\pi_+^{1,0}\pi_-^{1,0}$	−54.346415	−54.386262	−54.406242	−54.407456	−54.391290	−54.359172	−54.312455
$\sigma^{3,2}\pi_+^{1,1}$	−54.266542	−54.205906	−54.124584	−54.023952	−53.905617	−53.771151	−53.622003
$\sigma^{3,2}\pi_-^{1,1}$	−54.266542	−54.405906	−54.524584	−54.623952	−54.705617	−54.771151	−54.822003
$\sigma^{3,3}\pi_-^{1,0}$	−54.264579	−54.355499	−54.428638	−54.484942	−54.525609	−54.551878	−54.564934
$\sigma^{3,1}\pi_+^{1,0}\pi_-^{1,0}\delta_-^{1,0}$	−53.802572	−54.081534	−54.313733	−54.525825	−54.723946	−54.909331	−55.081877
$\sigma^{3,1}\pi_+^{1,0}\pi_-^{1,0}\phi_-^{1,0}$	−53.777611	−54.049681	−54.264188	−54.459028	−54.633385	−54.797872	−54.952353

Table S229: Total energies in E_h for the N atom in the AHGBSP3-7 basis set in fully uncontracted form, employing the real-orbital approximation.

	$0.00B_0$	$0.10B_0$	$0.20B_0$	$0.30B_0$	$0.40B_0$	$0.50B_0$	$0.60B_0$
$\sigma^{3,2}\pi_+^{1,0}\pi_-^{1,0}$	−54.404536	−54.544509	−54.664836	−54.766552	−54.850987	−54.919522	−54.973484
$\sigma^{2,3}\pi_+^{1,0}\pi_-^{1,0}$	−54.347417	−54.387265	−54.407249	−54.408476	−54.392334	−54.360250	−54.313572
$\sigma^{3,2}\pi_+^{1,1}$	−54.267534	−54.206899	−54.125581	−54.024963	−53.906659	−53.772236	−53.623133
$\sigma^{3,2}\pi_-^{1,1}$	−54.267534	−54.406899	−54.525581	−54.624963	−54.706659	−54.772236	−54.823133
$\sigma^{3,3}\pi_-^{1,0}$	−54.265575	−54.356496	−54.429639	−54.485953	−54.526640	−54.552941	−54.566038
$\sigma^{3,1}\pi_+^{1,0}\pi_-^{1,0}\delta_-^{1,0}$	−53.803595	−54.082657	−54.314771	−54.527406	−54.725429	−54.910412	−55.083314
$\sigma^{3,1}\pi_+^{1,0}\pi_-^{1,0}\phi_-^{1,0}$	−53.778635	−54.050767	−54.266429	−54.460133	−54.637829	−54.801901	−54.953236

Table S230: Total energies in E_h for the N atom in the AHGBSP3-9 basis set in fully uncontracted form, employing the real-orbital approximation.

	$0.00B_0$	$0.10B_0$	$0.20B_0$	$0.30B_0$	$0.40B_0$	$0.50B_0$	$0.60B_0$
$\sigma^{3,2}\pi_+^{1,0}\pi_-^{1,0}$	−54.404548	−54.544521	−54.664848	−54.766565	−54.851000	−54.919537	−54.973499
$\sigma^{2,3}\pi_+^{1,0}\pi_-^{1,0}$	−54.347429	−54.387276	−54.407261	−54.408489	−54.392347	−54.360264	−54.313587
$\sigma^{3,2}\pi_+^{1,1}$	−54.267546	−54.206910	−54.125593	−54.024976	−53.906672	−53.772252	−53.623149
$\sigma^{3,2}\pi_-^{1,1}$	−54.267546	−54.406910	−54.525593	−54.624976	−54.706672	−54.772252	−54.823149
$\sigma^{3,3}\pi_-^{1,0}$	−54.265587	−54.356508	−54.429651	−54.485965	−54.526653	−54.552955	−54.566052
$\sigma^{3,1}\pi_+^{1,0}\pi_-^{1,0}\delta_-^{1,0}$	−53.803608	−54.082672	−54.314789	−54.527490	−54.725474	−54.910454	−55.083464
$\sigma^{3,1}\pi_+^{1,0}\pi_-^{1,0}\phi_-^{1,0}$	−53.778648	−54.050800	−54.266516	−54.460373	−54.637997	−54.801952	−54.953805

Table S231: Total energies in E_h for the N atom in the 6-311++G(3df,3pd) basis set in fully uncontracted form, employing the real-orbital approximation.

	$0.00B_0$	$0.10B_0$	$0.20B_0$	$0.30B_0$	$0.40B_0$	$0.50B_0$	$0.60B_0$
$\sigma^{3,2}\pi_+^{1,0}\pi_-^{1,0}$	−54.399108	−54.539031	−54.659146	−54.760346	−54.843800	−54.910775	−54.962568
$\sigma^{2,3}\pi_+^{1,0}\pi_-^{1,0}$	−54.341545	−54.381347	−54.401122	−54.401815	−54.384631	−54.350861	−54.301814
$\sigma^{3,2}\pi_+^{1,1}$	−54.262225	−54.201535	−54.119981	−54.018794	−53.899396	−53.763175	−53.611495
$\sigma^{3,2}\pi_-^{1,1}$	−54.262225	−54.401535	−54.519981	−54.618794	−54.699396	−54.763175	−54.811495
$\sigma^{3,3}\pi_-^{1,0}$	−54.260188	−54.351107	−54.424159	−54.480103	−54.519943	−54.544758	−54.555633
$\sigma^{3,1}\pi_+^{1,0}\pi_-^{1,0}\delta_-^{1,0}$	−53.458995	−53.794168	−54.099886	−54.376714	−54.625511	−54.847381	−55.043651
$\sigma^{3,1}\pi_+^{1,0}\pi_-^{1,0}\phi_-^{1,0}$	−50.457023	−50.846978	−51.217087	−51.567983	−51.900514	−52.215616	−52.514264

Table S232: Total energies in E_h for the N atom in the def2-TZVP basis set in fully uncontracted form, employing the real-orbital approximation.

	$0.00B_0$	$0.10B_0$	$0.20B_0$	$0.30B_0$	$0.40B_0$	$0.50B_0$	$0.60B_0$
$\sigma^{3,2}\pi_+^{1,0}\pi_-^{1,0}$	−54.403297	−54.543279	−54.663525	−54.764868	−54.848526	−54.915899	−54.968394
$\sigma^{2,3}\pi_+^{1,0}\pi_-^{1,0}$	−54.345355	−54.385224	−54.405146	−54.405990	−54.389019	−54.355673	−54.307393
$\sigma^{3,2}\pi_+^{1,1}$	−54.265850	−54.205300	−54.124010	−54.022992	−53.903704	−53.767800	−53.616898
$\sigma^{3,2}\pi_-^{1,1}$	−54.265850	−54.405300	−54.524010	−54.622992	−54.703704	−54.767800	−54.816898
$\sigma^{3,3}\pi_-^{1,0}$	−54.263756	−54.354753	−54.427979	−54.484091	−54.524066	−54.549062	−54.560293
$\sigma^{3,1}\pi_+^{1,0}\pi_-^{1,0}\delta_-^{1,0}$	−53.034674	−53.373560	−53.690401	−53.985720	−54.260283	−54.514982	−54.750738
$\sigma^{3,1}\pi_+^{1,0}\pi_-^{1,0}\phi_-^{1,0}$	−50.142245	−50.532354	−50.902919	−51.254595	−51.588325	−51.905171	−52.206175

Table S233: Total energies in E_h for the O atom in the cc-pVDZ basis set in fully uncontracted form, employing the real-orbital approximation.

	$0.00B_0$	$0.10B_0$	$0.20B_0$	$0.30B_0$	$0.40B_0$	$0.50B_0$	$0.60B_0$
$\sigma^{3,3}\pi_+^{1,0}\pi_-^{1,0}$	−74.792436	−74.884093	−74.959142	−75.017802	−75.060434	−75.087522	−75.099659
$\sigma^{3,2}\pi_+^{1,1}\pi_-^{1,0}$	−74.790318	−74.830889	−74.852695	−74.856015	−74.841306	−74.809183	−74.760400
$\sigma^{3,2}\pi_+^{1,0}\pi_-^{1,1}$	−74.790318	−74.930889	−75.052695	−75.156015	−75.241306	−75.309183	−75.360400
$\sigma^{3,3}\pi_-^{1,1}$	−74.665361	−74.756807	−74.831221	−74.888835	−74.930021	−74.955289	−74.965260
$\sigma^{3,2}\pi_+^{1,0}\pi_-^{1,0}\delta_-^{1,0}$	−72.214698	−72.506012	−72.780024	−73.036934	−73.277068	−73.500862	−73.708850
$\sigma^{3,2}\pi_+^{1,0}\pi_-^{2,0}$	−73.846104	−74.084641	−74.300302	−74.493228	−74.663651	−74.811886	−74.938316
$\sigma^{3,2}\pi_+^{1,0}\pi_-^{1,0}\phi_-^{1,0}$							

Table S234: Total energies in E_h for the O atom in the cc-pVTZ basis set in fully uncontracted form, employing the real-orbital approximation.

	$0.00B_0$	$0.10B_0$	$0.20B_0$	$0.30B_0$	$0.40B_0$	$0.50B_0$	$0.60B_0$
$\sigma^{3,3}\pi_+^{1,0}\pi_-^{1,0}$	−74.811819	−74.903363	−74.978111	−75.036403	−75.078777	−75.105930	−75.118672
$\sigma^{3,2}\pi_+^{1,1}\pi_-^{1,0}$	−74.808451	−74.848851	−74.870208	−74.872976	−74.857874	−74.825834	−74.777932
$\sigma^{3,2}\pi_+^{1,0}\pi_-^{1,1}$	−74.808451	−74.948851	−75.070208	−75.172976	−75.257874	−75.325834	−75.377932
$\sigma^{3,3}\pi_-^{1,1}$	−74.683224	−74.774489	−74.848411	−74.905369	−74.945962	−74.970967	−74.981286
$\sigma^{3,2}\pi_+^{1,0}\pi_-^{1,0}\delta_-^{1,0}$	−73.273142	−73.563440	−73.834427	−74.086376	−74.319717	−74.535007	−74.732891
$\sigma^{3,2}\pi_+^{1,0}\pi_-^{2,0}$	−74.099220	−74.336008	−74.546448	−74.730765	−74.889321	−75.022603	−75.131210
$\sigma^{3,2}\pi_+^{1,0}\pi_-^{1,0}\phi_-^{1,0}$	−69.560091	−69.901204	−70.224649	−70.530740	−70.819967	−71.092964	−71.350455

Table S235: Total energies in E_h for the O atom in the cc-pVQZ basis set in fully uncontracted form, employing the real-orbital approximation.

	$0.00B_0$	$0.10B_0$	$0.20B_0$	$0.30B_0$	$0.40B_0$	$0.50B_0$	$0.60B_0$
$\sigma^{3,3}\pi_+^{1,0}\pi_-^{1,0}$	−74.817303	−74.908805	−74.983460	−75.041694	−75.084162	−75.111678	−75.125146
$\sigma^{3,2}\pi_+^{1,1}\pi_-^{1,0}$	−74.813707	−74.854037	−74.875238	−74.877906	−74.862951	−74.831482	−74.784694
$\sigma^{3,2}\pi_+^{1,0}\pi_-^{1,1}$	−74.813707	−74.954037	−75.075238	−75.177906	−75.262951	−75.331482	−75.384694
$\sigma^{3,3}\pi_-^{1,1}$	−74.688414	−74.779603	−74.853346	−74.910141	−74.950753	−74.976120	−74.987266
$\sigma^{3,2}\pi_+^{1,0}\pi_-^{1,0}\delta_-^{1,0}$	−73.667999	−73.957076	−74.224417	−74.470337	−74.695326	−74.900005	−75.085075
$\sigma^{3,2}\pi_+^{1,0}\pi_-^{2,0}$	−74.228685	−74.463669	−74.668741	−74.844255	−74.990802	−75.109204	−75.200532
$\sigma^{3,2}\pi_+^{1,0}\pi_-^{1,0}\phi_-^{1,0}$	−71.560731	−71.901019	−72.221990	−72.523957	−72.807395	−73.072895	−73.321104

Table S236: Total energies in E_h for the O atom in the cc-pV5Z basis set in fully uncontracted form, employing the real-orbital approximation.

	$0.00B_0$	$0.10B_0$	$0.20B_0$	$0.30B_0$	$0.40B_0$	$0.50B_0$	$0.60B_0$
$\sigma^{3,3}\pi_+^{1,0}\pi_-^{1,0}$	−74.818782	−74.910260	−74.984875	−75.043132	−75.085772	−75.113665	−75.127723
$\sigma^{3,2}\pi_+^{1,1}\pi_-^{1,0}$	−74.815146	−74.855434	−74.876570	−74.879302	−74.864671	−74.833825	−74.787886
$\sigma^{3,2}\pi_+^{1,0}\pi_-^{1,1}$	−74.815146	−74.955434	−75.076570	−75.179302	−75.264671	−75.333825	−75.387886
$\sigma^{3,3}\pi_-^{1,1}$	−74.689810	−74.780955	−74.854619	−74.911427	−74.952268	−74.978146	−74.990064
$\sigma^{3,2}\pi_+^{1,0}\pi_-^{1,0}\delta_-^{1,0}$	−73.846865	−74.134784	−74.398669	−74.638890	−74.856022	−75.050793	−75.224055
$\sigma^{3,2}\pi_+^{1,0}\pi_-^{2,0}$	−74.350763	−74.582112	−74.776510	−74.935047	−75.059657	−75.153302	−75.220150
$\sigma^{3,2}\pi_+^{1,0}\pi_-^{1,0}\phi_-^{1,0}$	−72.494634	−72.833672	−73.150893	−73.446592	−73.721215	−73.975302	−74.209442

Table S237: Total energies in E_h for the O atom in the aug-cc-pVDZ basis set in fully uncontracted form, employing the real-orbital approximation.

	$0.00B_0$	$0.10B_0$	$0.20B_0$	$0.30B_0$	$0.40B_0$	$0.50B_0$	$0.60B_0$
$\sigma^{3,3}\pi_+^{1,0}\pi_-^{1,0}$	−74.796853	−74.888285	−74.962754	−75.020726	−75.062840	−75.089808	−75.102366
$\sigma^{3,2}\pi_+^{1,1}\pi_-^{1,0}$	−74.794404	−74.834583	−74.855412	−74.857623	−74.842107	−74.809756	−74.761442
$\sigma^{3,2}\pi_+^{1,0}\pi_-^{1,1}$	−74.794404	−74.934583	−75.055412	−75.157623	−75.242107	−75.309756	−75.361442
$\sigma^{3,3}\pi_-^{1,1}$	−74.669896	−74.760995	−74.834529	−74.891099	−74.931466	−74.956409	−74.966696
$\sigma^{3,2}\pi_+^{1,0}\pi_-^{1,0}\delta_-^{1,0}$	−73.844446	−74.132305	−74.395986	−74.635786	−74.852161	−75.045689	−75.217051
$\sigma^{3,2}\pi_+^{1,0}\pi_-^{2,0}$	−74.420444	−74.644263	−74.816575	−74.940319	−75.021776	−75.072684	−75.110369
$\sigma^{3,2}\pi_+^{1,0}\pi_-^{1,0}\phi_-^{1,0}$							

Table S238: Total energies in E_h for the O atom in the aug-cc-pVTZ basis set in fully uncontracted form, employing the real-orbital approximation.

	$0.00B_0$	$0.10B_0$	$0.20B_0$	$0.30B_0$	$0.40B_0$	$0.50B_0$	$0.60B_0$
$\sigma^{3,3}\pi_+^{1,0}\pi_-^{1,0}$	−74.813032	−74.904477	−74.979000	−75.037115	−75.079545	−75.107128	−75.120756
$\sigma^{3,2}\pi_+^{1,1}\pi_-^{1,0}$	−74.809476	−74.849722	−74.870761	−74.873351	−74.858457	−74.827120	−74.780421
$\sigma^{3,2}\pi_+^{1,0}\pi_-^{1,1}$	−74.809476	−74.949722	−75.070761	−75.173351	−75.258457	−75.327120	−75.380421
$\sigma^{3,3}\pi_-^{1,1}$	−74.684294	−74.775409	−74.848999	−74.905701	−74.946350	−74.971867	−74.983213
$\sigma^{3,2}\pi_+^{1,0}\pi_-^{1,0}\delta_-^{1,0}$	−74.089325	−74.374111	−74.628654	−74.853503	−75.049568	−75.218133	−75.360896
$\sigma^{3,2}\pi_+^{1,0}\pi_-^{2,0}$	−74.448830	−74.669972	−74.835476	−74.952721	−75.036879	−75.108047	−75.176927
$\sigma^{3,2}\pi_+^{1,0}\pi_-^{1,0}\phi_-^{1,0}$	−72.793405	−73.131743	−73.446856	−73.739028	−74.008689	−74.256370	−74.482669

Table S239: Total energies in E_h for the O atom in the aug-cc-pVQZ basis set in fully uncontracted form, employing the real-orbital approximation.

	$0.00B_0$	$0.10B_0$	$0.20B_0$	$0.30B_0$	$0.40B_0$	$0.50B_0$	$0.60B_0$
$\sigma^{3,3}\pi_+^{1,0}\pi_-^{1,0}$	−74.817635	−74.909094	−74.983673	−75.041925	−75.084625	−75.112671	−75.126995
$\sigma^{3,2}\pi_+^{1,1}\pi_-^{1,0}$	−74.813997	−74.854264	−74.875374	−74.878115	−74.863514	−74.832691	−74.786790
$\sigma^{3,2}\pi_+^{1,0}\pi_-^{1,1}$	−74.813997	−74.954264	−75.075374	−75.178115	−75.263514	−75.332691	−75.386790
$\sigma^{3,3}\pi_-^{1,1}$	−74.688679	−74.779805	−74.853441	−74.910260	−74.951164	−74.977155	−74.989260
$\sigma^{3,2}\pi_+^{1,0}\pi_-^{1,0}\delta_-^{1,0}$	−74.210765	−74.491982	−74.736028	−74.944135	−75.118517	−75.262630	−75.381435
$\sigma^{3,2}\pi_+^{1,0}\pi_-^{2,0}$	−74.460591	−74.679392	−74.839898	−74.956199	−75.050905	−75.138387	−75.217282
$\sigma^{3,2}\pi_+^{1,0}\pi_-^{1,0}\phi_-^{1,0}$	−73.419651	−73.755102	−74.061565	−74.339355	−74.588959	−74.811004	−75.006225

Table S240: Total energies in E_h for the O atom in the aug-cc-pV5Z basis set in fully uncontracted form, employing the real-orbital approximation.

	$0.00B_0$	$0.10B_0$	$0.20B_0$	$0.30B_0$	$0.40B_0$	$0.50B_0$	$0.60B_0$
$\sigma^{3,3}\pi_+^{1,0}\pi_-^{1,0}$	−74.818839	−74.910307	−74.984923	−75.043262	−75.086126	−75.114417	−75.129043
$\sigma^{3,2}\pi_+^{1,1}\pi_-^{1,0}$	−74.815187	−74.855465	−74.876616	−74.879457	−74.865052	−74.834535	−74.789002
$\sigma^{3,2}\pi_+^{1,0}\pi_-^{1,1}$	−74.815187	−74.955465	−75.076616	−75.179457	−75.265052	−75.334535	−75.389002
$\sigma^{3,3}\pi_-^{1,1}$	−74.689834	−74.780968	−74.854639	−74.911552	−74.952649	−74.978947	−74.991441
$\sigma^{3,2}\pi_+^{1,0}\pi_-^{1,0}\delta_-^{1,0}$	−74.258205	−74.536943	−74.773848	−74.971139	−75.132968	−75.265915	−75.378747
$\sigma^{3,2}\pi_+^{1,0}\pi_-^{2,0}$	−74.469859	−74.684748	−74.839941	−74.962583	−75.070424	−75.163173	−75.239832
$\sigma^{3,2}\pi_+^{1,0}\pi_-^{1,0}\phi_-^{1,0}$	−73.727077	−74.059311	−74.356164	−74.618084	−74.845803	−75.040335	−75.203003

Table S241: Total energies in E_h for the O atom in the HGBSP1-5 basis set in fully uncontracted form, employing the real-orbital approximation.

	$0.00B_0$	$0.10B_0$	$0.20B_0$	$0.30B_0$	$0.40B_0$	$0.50B_0$	$0.60B_0$
$\sigma^{3,3}\pi_+^{1,0}\pi_-^{1,0}$	−74.816156	−74.907661	−74.982363	−75.040758	−75.083545	−75.111516	−75.125496
$\sigma^{3,2}\pi_+^{1,1}\pi_-^{1,0}$	−74.813531	−74.853784	−74.874841	−74.877466	−74.862646	−74.831433	−74.784869
$\sigma^{3,2}\pi_+^{1,0}\pi_-^{1,1}$	−74.813531	−74.953784	−75.074841	−75.177466	−75.262646	−75.331433	−75.384869
$\sigma^{3,3}\pi_-^{1,1}$	−74.688277	−74.779436	−74.853152	−74.910053	−74.950970	−74.976805	−74.988465
$\sigma^{3,2}\pi_+^{1,0}\pi_-^{1,0}\delta_-^{1,0}$	−74.431629	−74.658488	−74.835474	−74.992846	−75.133868	−75.263139	−75.381333
$\sigma^{3,2}\pi_+^{1,0}\pi_-^{2,0}$	−74.478402	−74.684813	−74.840159	−74.965834	−75.066632	−75.148859	−75.216317
$\sigma^{3,2}\pi_+^{1,0}\pi_-^{1,0}\phi_-^{1,0}$							

Table S242: Total energies in E_h for the O atom in the HGBSP1-7 basis set in fully uncontracted form, employing the real-orbital approximation.

	$0.00B_0$	$0.10B_0$	$0.20B_0$	$0.30B_0$	$0.40B_0$	$0.50B_0$	$0.60B_0$
$\sigma^{3,3}\pi_+^{1,0}\pi_-^{1,0}$	−74.817530	−74.909036	−74.983741	−75.042142	−75.084941	−75.112934	−75.126946
$\sigma^{3,2}\pi_+^{1,1}\pi_-^{1,0}$	−74.814904	−74.855158	−74.876218	−74.878850	−74.864048	−74.832867	−74.786350
$\sigma^{3,2}\pi_+^{1,0}\pi_-^{1,1}$	−74.814904	−74.955158	−75.076218	−75.178850	−75.264048	−75.332867	−75.386350
$\sigma^{3,3}\pi_-^{1,1}$	−74.689648	−74.780808	−74.854527	−74.911434	−74.952366	−74.978231	−74.989935
$\sigma^{3,2}\pi_+^{1,0}\pi_-^{1,0}\delta_-^{1,0}$	−74.433044	−74.659891	−74.837240	−74.994367	−75.136320	−75.265608	−75.382999
$\sigma^{3,2}\pi_+^{1,0}\pi_-^{2,0}$	−74.479821	−74.686339	−74.841760	−74.967521	−75.070273	−75.153394	−75.219802
$\sigma^{3,2}\pi_+^{1,0}\pi_-^{1,0}\phi_-^{1,0}$							

Table S243: Total energies in E_h for the O atom in the HGBSP1-9 basis set in fully uncontracted form, employing the real-orbital approximation.

	$0.00B_0$	$0.10B_0$	$0.20B_0$	$0.30B_0$	$0.40B_0$	$0.50B_0$	$0.60B_0$
$\sigma^{3,3}\pi_+^{1,0}\pi_-^{1,0}$	−74.817546	−74.909053	−74.983757	−75.042159	−75.084958	−75.112952	−75.126965
$\sigma^{3,2}\pi_+^{1,1}\pi_-^{1,0}$	−74.814921	−74.855175	−74.876235	−74.878867	−74.864065	−74.832886	−74.786371
$\sigma^{3,2}\pi_+^{1,0}\pi_-^{1,1}$	−74.814921	−74.955175	−75.076235	−75.178867	−75.264065	−75.332886	−75.386371
$\sigma^{3,3}\pi_-^{1,1}$	−74.689665	−74.780824	−74.854543	−74.911451	−74.952383	−74.978249	−74.989954
$\sigma^{3,2}\pi_+^{1,0}\pi_-^{1,0}\delta_-^{1,0}$	−74.433061	−74.659926	−74.837327	−74.994392	−75.136505	−75.265716	−75.383005
$\sigma^{3,2}\pi_+^{1,0}\pi_-^{2,0}$	−74.479839	−74.686365	−74.841855	−74.967555	−75.070402	−75.153866	−75.220319
$\sigma^{3,2}\pi_+^{1,0}\pi_-^{1,0}\phi_-^{1,0}$							

Table S244: Total energies in E_h for the O atom in the HGBSP2-5 basis set in fully uncontracted form, employing the real-orbital approximation.

	$0.00B_0$	$0.10B_0$	$0.20B_0$	$0.30B_0$	$0.40B_0$	$0.50B_0$	$0.60B_0$
$\sigma^{3,3}\pi_+^{1,0}\pi_-^{1,0}$	−74.817578	−74.909047	−74.983673	−75.042039	−75.084940	−75.113258	−75.127890
$\sigma^{3,2}\pi_+^{1,1}\pi_-^{1,0}$	−74.813930	−74.854209	−74.875371	−74.878236	−74.863855	−74.833335	−74.787762
$\sigma^{3,2}\pi_+^{1,0}\pi_-^{1,1}$	−74.813930	−74.954209	−75.075371	−75.178236	−75.263855	−75.333335	−75.387762
$\sigma^{3,3}\pi_-^{1,1}$	−74.688580	−74.779715	−74.853399	−74.910344	−74.951481	−74.977799	−74.990277
$\sigma^{3,2}\pi_+^{1,0}\pi_-^{1,0}\delta_-^{1,0}$	−74.431629	−74.658492	−74.835500	−74.992928	−75.134067	−75.263551	−75.382089
$\sigma^{3,2}\pi_+^{1,0}\pi_-^{2,0}$	−74.478404	−74.685680	−74.844664	−74.976632	−75.087014	−75.180346	−75.259289
$\sigma^{3,2}\pi_+^{1,0}\pi_-^{1,0}\phi_-^{1,0}$	−74.406947	−74.628590	−74.794052	−74.938167	−75.066394	−75.176298	−75.275898

Table S245: Total energies in E_h for the O atom in the HGBSP2-7 basis set in fully uncontracted form, employing the real-orbital approximation.

	$0.00B_0$	$0.10B_0$	$0.20B_0$	$0.30B_0$	$0.40B_0$	$0.50B_0$	$0.60B_0$
$\sigma^{3,3}\pi_+^{1,0}\pi_-^{1,0}$	−74.818963	−74.910433	−74.985062	−75.043434	−75.086347	−75.114687	−75.129349
$\sigma^{3,2}\pi_+^{1,1}\pi_-^{1,0}$	−74.815307	−74.855587	−74.876752	−74.879624	−74.865260	−74.834771	−74.789241
$\sigma^{3,2}\pi_+^{1,0}\pi_-^{1,1}$	−74.815307	−74.955587	−75.076752	−75.179624	−75.265260	−75.334771	−75.389241
$\sigma^{3,3}\pi_-^{1,1}$	−74.689953	−74.781090	−74.854776	−74.911728	−74.952880	−74.979227	−74.991743
$\sigma^{3,2}\pi_+^{1,0}\pi_-^{1,0}\delta_-^{1,0}$	−74.433044	−74.659896	−74.837265	−74.994449	−75.136519	−75.266020	−75.383754
$\sigma^{3,2}\pi_+^{1,0}\pi_-^{2,0}$	−74.479823	−74.687189	−74.846283	−74.978258	−75.089693	−75.183732	−75.262711
$\sigma^{3,2}\pi_+^{1,0}\pi_-^{1,0}\phi_-^{1,0}$	−74.408366	−74.630604	−74.796500	−74.940672	−75.067933	−75.181469	−75.282843

Table S246: Total energies in E_h for the O atom in the HGBSP2-9 basis set in fully uncontracted form, employing the real-orbital approximation.

	$0.00B_0$	$0.10B_0$	$0.20B_0$	$0.30B_0$	$0.40B_0$	$0.50B_0$	$0.60B_0$
$\sigma^{3,3}\pi_+^{1,0}\pi_-^{1,0}$	−74.818980	−74.910450	−74.985078	−75.043451	−75.086364	−75.114705	−75.129367
$\sigma^{3,2}\pi_+^{1,1}\pi_-^{1,0}$	−74.815323	−74.855603	−74.876768	−74.879641	−74.865277	−74.834790	−74.789261
$\sigma^{3,2}\pi_+^{1,0}\pi_-^{1,1}$	−74.815323	−74.955603	−75.076768	−75.179641	−75.265277	−75.334790	−75.389261
$\sigma^{3,3}\pi_-^{1,1}$	−74.689970	−74.781106	−74.854793	−74.911745	−74.952897	−74.979245	−74.991763
$\sigma^{3,2}\pi_+^{1,0}\pi_-^{1,0}\delta_-^{1,0}$	−74.433062	−74.659930	−74.837352	−74.994474	−75.136704	−75.266128	−75.383760
$\sigma^{3,2}\pi_+^{1,0}\pi_-^{2,0}$	−74.479841	−74.687214	−74.846345	−74.978306	−75.089733	−75.183935	−75.263042
$\sigma^{3,2}\pi_+^{1,0}\pi_-^{1,0}\phi_-^{1,0}$	−74.408384	−74.630675	−74.796648	−74.940690	−75.068287	−75.181848	−75.282833

Table S247: Total energies in E_h for the O atom in the HGBSP3-5 basis set in fully uncontracted form, employing the real-orbital approximation.

	$0.00B_0$	$0.10B_0$	$0.20B_0$	$0.30B_0$	$0.40B_0$	$0.50B_0$	$0.60B_0$
$\sigma^{3,3}\pi_+^{1,0}\pi_-^{1,0}$	−74.817579	−74.909047	−74.983673	−75.042039	−75.084941	−75.113261	−75.127897
$\sigma^{3,2}\pi_+^{1,1}\pi_-^{1,0}$	−74.813930	−74.854209	−74.875371	−74.878236	−74.863855	−74.833335	−74.787764
$\sigma^{3,2}\pi_+^{1,0}\pi_-^{1,1}$	−74.813930	−74.954209	−75.075371	−75.178236	−75.263855	−75.333335	−75.387764
$\sigma^{3,3}\pi_-^{1,1}$	−74.688580	−74.779715	−74.853399	−74.910344	−74.951481	−74.977800	−74.990280
$\sigma^{3,2}\pi_+^{1,0}\pi_-^{1,0}\delta_-^{1,0}$	−74.431629	−74.659594	−74.838974	−74.998925	−75.142794	−75.274559	−75.395219
$\sigma^{3,2}\pi_+^{1,0}\pi_-^{2,0}$	−74.478404	−74.685680	−74.844664	−74.976632	−75.087014	−75.180346	−75.259290
$\sigma^{3,2}\pi_+^{1,0}\pi_-^{1,0}\phi_-^{1,0}$	−74.406947	−74.628590	−74.794052	−74.938167	−75.066394	−75.176298	−75.275899

Table S248: Total energies in E_h for the O atom in the HGBSP3-7 basis set in fully uncontracted form, employing the real-orbital approximation.

	$0.00B_0$	$0.10B_0$	$0.20B_0$	$0.30B_0$	$0.40B_0$	$0.50B_0$	$0.60B_0$
$\sigma^{3,3}\pi_+^{1,0}\pi_-^{1,0}$	−74.818963	−74.910433	−74.985062	−75.043434	−75.086348	−75.114690	−75.129356
$\sigma^{3,2}\pi_+^{1,1}\pi_-^{1,0}$	−74.815307	−74.855587	−74.876752	−74.879624	−74.865260	−74.834772	−74.789243
$\sigma^{3,2}\pi_+^{1,0}\pi_-^{1,1}$	−74.815307	−74.955587	−75.076752	−75.179624	−75.265260	−75.334772	−75.389243
$\sigma^{3,3}\pi_-^{1,1}$	−74.689953	−74.781090	−74.854776	−74.911729	−74.952881	−74.979228	−74.991747
$\sigma^{3,2}\pi_+^{1,0}\pi_-^{1,0}\delta_-^{1,0}$	−74.433044	−74.661004	−74.840723	−75.000422	−75.145056	−75.276924	−75.397002
$\sigma^{3,2}\pi_+^{1,0}\pi_-^{2,0}$	−74.479823	−74.687189	−74.846283	−74.978258	−75.089693	−75.183733	−75.262713
$\sigma^{3,2}\pi_+^{1,0}\pi_-^{1,0}\phi_-^{1,0}$	−74.397115	−74.630604	−74.796500	−74.940672	−75.067933	−75.181469	−75.282844

Table S249: Total energies in E_h for the O atom in the HGBSP3-9 basis set in fully uncontracted form, employing the real-orbital approximation.

	$0.00B_0$	$0.10B_0$	$0.20B_0$	$0.30B_0$	$0.40B_0$	$0.50B_0$	$0.60B_0$
$\sigma^{3,3}\pi_+^{1,0}\pi_-^{1,0}$	−74.818980	−74.910450	−74.985079	−75.043451	−75.086366	−75.114708	−75.129375
$\sigma^{3,2}\pi_+^{1,1}\pi_-^{1,0}$	−74.815323	−74.855603	−74.876768	−74.879641	−74.865278	−74.834790	−74.789263
$\sigma^{3,2}\pi_+^{1,0}\pi_-^{1,1}$	−74.815323	−74.955603	−75.076768	−75.179641	−75.265278	−75.334790	−75.389263
$\sigma^{3,3}\pi_-^{1,1}$	−74.689970	−74.781106	−74.854793	−74.911745	−74.952898	−74.979246	−74.991766
$\sigma^{3,2}\pi_+^{1,0}\pi_-^{1,0}\delta_-^{1,0}$	−74.433062	−74.661036	−74.840794	−75.000453	−75.145186	−75.277033	−75.397037
$\sigma^{3,2}\pi_+^{1,0}\pi_-^{2,0}$	−74.479841	−74.687214	−74.846345	−74.978306	−75.089733	−75.183936	−75.263043
$\sigma^{3,2}\pi_+^{1,0}\pi_-^{1,0}\phi_-^{1,0}$	−74.408384	−74.630675	−74.796648	−74.940690	−75.068287	−75.181848	−75.282834

Table S250: Total energies in E_h for the O atom in the AHGBSP1-5 basis set in fully uncontracted form, employing the real-orbital approximation.

	$0.00B_0$	$0.10B_0$	$0.20B_0$	$0.30B_0$	$0.40B_0$	$0.50B_0$	$0.60B_0$
$\sigma^{3,3}\pi_+^{1,0}\pi_-^{1,0}$	−74.816157	−74.907663	−74.982365	−75.040760	−75.083547	−75.111520	−75.125501
$\sigma^{3,2}\pi_+^{1,1}\pi_-^{1,0}$	−74.813532	−74.853785	−74.874842	−74.877468	−74.862648	−74.831437	−74.784875
$\sigma^{3,2}\pi_+^{1,0}\pi_-^{1,1}$	−74.813532	−74.953785	−75.074842	−75.177468	−75.262648	−75.331437	−75.384875
$\sigma^{3,3}\pi_-^{1,1}$	−74.688278	−74.779437	−74.853153	−74.910054	−74.950972	−74.976809	−74.988471
$\sigma^{3,2}\pi_+^{1,0}\pi_-^{1,0}\delta_-^{1,0}$	−74.431633	−74.658493	−74.835504	−74.992852	−75.133898	−75.263164	−75.381343
$\sigma^{3,2}\pi_+^{1,0}\pi_-^{2,0}$	−74.478408	−74.684836	−74.840200	−74.965852	−75.066750	−75.149005	−75.216404
$\sigma^{3,2}\pi_+^{1,0}\pi_-^{1,0}\phi_-^{1,0}$							

Table S251: Total energies in E_h for the O atom in the AHGBSP1-7 basis set in fully uncontracted form, employing the real-orbital approximation.

	$0.00B_0$	$0.10B_0$	$0.20B_0$	$0.30B_0$	$0.40B_0$	$0.50B_0$	$0.60B_0$
$\sigma^{3,3}\pi_+^{1,0}\pi_-^{1,0}$	−74.817530	−74.909036	−74.983741	−75.042142	−75.084941	−75.112934	−75.126946
$\sigma^{3,2}\pi_+^{1,1}\pi_-^{1,0}$	−74.814904	−74.855158	−74.876218	−74.878851	−74.864048	−74.832867	−74.786350
$\sigma^{3,2}\pi_+^{1,0}\pi_-^{1,1}$	−74.814904	−74.955158	−75.076218	−75.178851	−75.264048	−75.332867	−75.386350
$\sigma^{3,3}\pi_-^{1,1}$	−74.689648	−74.780808	−74.854527	−74.911434	−74.952367	−74.978231	−74.989935
$\sigma^{3,2}\pi_+^{1,0}\pi_-^{1,0}\delta_-^{1,0}$	−74.433044	−74.659895	−74.837245	−74.994367	−75.136325	−75.265610	−75.382999
$\sigma^{3,2}\pi_+^{1,0}\pi_-^{2,0}$	−74.479821	−74.686344	−74.841774	−74.967525	−75.070282	−75.153430	−75.219834
$\sigma^{3,2}\pi_+^{1,0}\pi_-^{1,0}\phi_-^{1,0}$							

Table S252: Total energies in E_h for the O atom in the AHGBSP1-9 basis set in fully uncontracted form, employing the real-orbital approximation.

	$0.00B_0$	$0.10B_0$	$0.20B_0$	$0.30B_0$	$0.40B_0$	$0.50B_0$	$0.60B_0$
$\sigma^{3,3}\pi_+^{1,0}\pi_-^{1,0}$	−74.817546	−74.909053	−74.983757	−75.042159	−75.084958	−75.112952	−75.126965
$\sigma^{3,2}\pi_+^{1,1}\pi_-^{1,0}$	−74.814921	−74.855175	−74.876235	−74.878867	−74.864065	−74.832886	−74.786371
$\sigma^{3,2}\pi_+^{1,0}\pi_-^{1,1}$	−74.814921	−74.955175	−75.076235	−75.178867	−75.264065	−75.332886	−75.386371
$\sigma^{3,3}\pi_-^{1,1}$	−74.689665	−74.780824	−74.854543	−74.911451	−74.952383	−74.978249	−74.989954
$\sigma^{3,2}\pi_+^{1,0}\pi_-^{1,0}\delta_-^{1,0}$	−74.433061	−74.659926	−74.837327	−74.994392	−75.136505	−75.265716	−75.383005
$\sigma^{3,2}\pi_+^{1,0}\pi_-^{2,0}$	−74.479839	−74.686366	−74.841855	−74.967557	−75.070402	−75.153870	−75.220323
$\sigma^{3,2}\pi_+^{1,0}\pi_-^{1,0}\phi_-^{1,0}$							

Table S253: Total energies in E_h for the O atom in the AHGBSP2-5 basis set in fully uncontracted form, employing the real-orbital approximation.

	$0.00B_0$	$0.10B_0$	$0.20B_0$	$0.30B_0$	$0.40B_0$	$0.50B_0$	$0.60B_0$
$\sigma^{3,3}\pi_+^{1,0}\pi_-^{1,0}$	−74.817580	−74.909049	−74.983675	−75.042041	−75.084942	−75.113261	−75.127895
$\sigma^{3,2}\pi_+^{1,1}\pi_-^{1,0}$	−74.813931	−74.854211	−74.875373	−74.878238	−74.863857	−74.833338	−74.787768
$\sigma^{3,2}\pi_+^{1,0}\pi_-^{1,1}$	−74.813931	−74.954211	−75.075373	−75.178238	−75.263857	−75.333338	−75.387768
$\sigma^{3,3}\pi_-^{1,1}$	−74.688581	−74.779716	−74.853400	−74.910346	−74.951483	−74.977803	−74.990282
$\sigma^{3,2}\pi_+^{1,0}\pi_-^{1,0}\delta_-^{1,0}$	−74.431633	−74.658498	−74.835530	−74.992934	−75.134097	−75.263577	−75.382098
$\sigma^{3,2}\pi_+^{1,0}\pi_-^{2,0}$	−74.478409	−74.685696	−74.844705	−74.976638	−75.087061	−75.180437	−75.259383
$\sigma^{3,2}\pi_+^{1,0}\pi_-^{1,0}\phi_-^{1,0}$	−74.406954	−74.628624	−74.794074	−74.938184	−75.066401	−75.176321	−75.275924

Table S254: Total energies in E_h for the O atom in the AHGBSP2-7 basis set in fully uncontracted form, employing the real-orbital approximation.

	$0.00B_0$	$0.10B_0$	$0.20B_0$	$0.30B_0$	$0.40B_0$	$0.50B_0$	$0.60B_0$
$\sigma^{3,3}\pi_+^{1,0}\pi_-^{1,0}$	−74.818963	−74.910433	−74.985062	−75.043434	−75.086347	−75.114687	−75.129349
$\sigma^{3,2}\pi_+^{1,1}\pi_-^{1,0}$	−74.815307	−74.855587	−74.876752	−74.879624	−74.865260	−74.834771	−74.789241
$\sigma^{3,2}\pi_+^{1,0}\pi_-^{1,1}$	−74.815307	−74.955587	−75.076752	−75.179624	−75.265260	−75.334771	−75.389241
$\sigma^{3,3}\pi_-^{1,1}$	−74.689953	−74.781090	−74.854776	−74.911728	−74.952880	−74.979227	−74.991744
$\sigma^{3,2}\pi_+^{1,0}\pi_-^{1,0}\delta_-^{1,0}$	−74.433044	−74.659899	−74.837271	−74.994449	−75.136525	−75.266022	−75.383754
$\sigma^{3,2}\pi_+^{1,0}\pi_-^{2,0}$	−74.479823	−74.687193	−74.846291	−74.978264	−75.089694	−75.183742	−75.262727
$\sigma^{3,2}\pi_+^{1,0}\pi_-^{1,0}\phi_-^{1,0}$	−74.408366	−74.630606	−74.796502	−74.940673	−75.067934	−75.181471	−75.282843

Table S255: Total energies in E_h for the O atom in the AHGBSP2-9 basis set in fully uncontracted form, employing the real-orbital approximation.

	$0.00B_0$	$0.10B_0$	$0.20B_0$	$0.30B_0$	$0.40B_0$	$0.50B_0$	$0.60B_0$
$\sigma^{3,3}\pi_+^{1,0}\pi_-^{1,0}$	−74.818980	−74.910450	−74.985079	−75.043451	−75.086364	−75.114705	−75.129367
$\sigma^{3,2}\pi_+^{1,1}\pi_-^{1,0}$	−74.815323	−74.855603	−74.876768	−74.879641	−74.865277	−74.834790	−74.789261
$\sigma^{3,2}\pi_+^{1,0}\pi_-^{1,1}$	−74.815323	−74.955603	−75.076768	−75.179641	−75.265277	−75.334790	−75.389261
$\sigma^{3,3}\pi_-^{1,1}$	−74.689970	−74.781106	−74.854793	−74.911745	−74.952897	−74.979245	−74.991763
$\sigma^{3,2}\pi_+^{1,0}\pi_-^{1,0}\delta_-^{1,0}$	−74.433062	−74.659931	−74.837353	−74.994474	−75.136704	−75.266128	−75.383760
$\sigma^{3,2}\pi_+^{1,0}\pi_-^{2,0}$	−74.479841	−74.687214	−74.846345	−74.978307	−75.089733	−75.183935	−75.263043
$\sigma^{3,2}\pi_+^{1,0}\pi_-^{1,0}\phi_-^{1,0}$	−74.408384	−74.630675	−74.796649	−74.940690	−75.068287	−75.181848	−75.282833

Table S256: Total energies in E_h for the O atom in the AHGBSP3-5 basis set in fully uncontracted form, employing the real-orbital approximation.

	$0.00B_0$	$0.10B_0$	$0.20B_0$	$0.30B_0$	$0.40B_0$	$0.50B_0$	$0.60B_0$
$\sigma^{3,3}\pi_+^{1,0}\pi_-^{1,0}$	−74.817580	−74.909049	−74.983675	−75.042041	−75.084943	−75.113264	−75.127902
$\sigma^{3,2}\pi_+^{1,1}\pi_-^{1,0}$	−74.813931	−74.854211	−74.875373	−74.878238	−74.863857	−74.833339	−74.787770
$\sigma^{3,2}\pi_+^{1,0}\pi_-^{1,1}$	−74.813931	−74.954211	−75.075373	−75.178238	−75.263857	−75.333339	−75.387770
$\sigma^{3,3}\pi_-^{1,1}$	−74.688581	−74.779716	−74.853400	−74.910346	−74.951483	−74.977804	−74.990285
$\sigma^{3,2}\pi_+^{1,0}\pi_-^{1,0}\delta_-^{1,0}$	−74.431633	−74.659601	−74.839001	−74.998929	−75.142813	−75.274581	−75.395232
$\sigma^{3,2}\pi_+^{1,0}\pi_-^{2,0}$	−74.478409	−74.685696	−74.844705	−74.976638	−75.087061	−75.180438	−75.259385
$\sigma^{3,2}\pi_+^{1,0}\pi_-^{1,0}\phi_-^{1,0}$	−74.406954	−74.628624	−74.794074	−74.938184	−75.066401	−75.176322	−75.275926

Table S257: Total energies in E_h for the O atom in the AHGBSP3-7 basis set in fully uncontracted form, employing the real-orbital approximation.

	$0.00B_0$	$0.10B_0$	$0.20B_0$	$0.30B_0$	$0.40B_0$	$0.50B_0$	$0.60B_0$
$\sigma^{3,3}\pi_+^{1,0}\pi_-^{1,0}$	−74.818963	−74.910433	−74.985062	−75.043434	−75.086348	−75.114690	−75.129356
$\sigma^{3,2}\pi_+^{1,1}\pi_-^{1,0}$	−74.815307	−74.855587	−74.876752	−74.879624	−74.865260	−74.834772	−74.789243
$\sigma^{3,2}\pi_+^{1,0}\pi_-^{1,1}$	−74.815307	−74.955587	−75.076752	−75.179624	−75.265260	−75.334772	−75.389243
$\sigma^{3,3}\pi_-^{1,1}$	−74.689953	−74.781090	−74.854776	−74.911729	−74.952881	−74.979228	−74.991747
$\sigma^{3,2}\pi_+^{1,0}\pi_-^{1,0}\delta_-^{1,0}$	−74.433044	−74.661007	−74.840727	−75.000422	−75.145059	−75.276927	−75.397002
$\sigma^{3,2}\pi_+^{1,0}\pi_-^{2,0}$	−74.479823	−74.687193	−74.846291	−74.978264	−75.089694	−75.183743	−75.262729
$\sigma^{3,2}\pi_+^{1,0}\pi_-^{1,0}\phi_-^{1,0}$	−74.408366	−74.630606	−74.796502	−74.940673	−75.067934	−75.181471	−75.282844

Table S258: Total energies in E_h for the O atom in the AHGBSP3-9 basis set in fully uncontracted form, employing the real-orbital approximation.

	$0.00B_0$	$0.10B_0$	$0.20B_0$	$0.30B_0$	$0.40B_0$	$0.50B_0$	$0.60B_0$
$\sigma^{3,3}\pi_+^{1,0}\pi_-^{1,0}$	−74.818980	−74.910450	−74.985079	−75.043451	−75.086366	−75.114708	−75.129375
$\sigma^{3,2}\pi_+^{1,1}\pi_-^{1,0}$	−74.815323	−74.855603	−74.876768	−74.879641	−74.865278	−74.834790	−74.789263
$\sigma^{3,2}\pi_+^{1,0}\pi_-^{1,1}$	−74.815323	−74.955603	−75.076768	−75.179641	−75.265278	−75.334790	−75.389263
$\sigma^{3,3}\pi_-^{1,1}$	−74.689970	−74.781106	−74.854793	−74.911745	−74.952898	−74.979246	−74.991766
$\sigma^{3,2}\pi_+^{1,0}\pi_-^{1,0}\delta_-^{1,0}$	−74.433062	−74.661036	−74.840794	−75.000453	−75.145187	−75.277033	−75.397037
$\sigma^{3,2}\pi_+^{1,0}\pi_-^{2,0}$	−74.479841	−74.687214	−74.846345	−74.978307	−75.089733	−75.183936	−75.263045
$\sigma^{3,2}\pi_+^{1,0}\pi_-^{1,0}\phi_-^{1,0}$	−74.408384	−74.630675	−74.796649	−74.940690	−75.068287	−75.181848	−75.282834

Table S259: Total energies in E_h for the O atom in the 6-311++G(3df,3pd) basis set in fully uncontracted form, employing the real-orbital approximation.

	$0.00B_0$	$0.10B_0$	$0.20B_0$	$0.30B_0$	$0.40B_0$	$0.50B_0$	$0.60B_0$
$\sigma^{3,3}\pi_+^{1,0}\pi_-^{1,0}$	−74.809602	−74.901053	−74.975574	−75.033622	−75.075848	−75.102999	−75.115868
$\sigma^{3,2}\pi_+^{1,1}\pi_-^{1,0}$	−74.806185	−74.846412	−74.867366	−74.869753	−74.854493	−74.822556	−74.774909
$\sigma^{3,2}\pi_+^{1,0}\pi_-^{1,1}$	−74.806185	−74.946412	−75.067366	−75.169753	−75.254493	−75.322556	−75.374909
$\sigma^{3,3}\pi_-^{1,1}$	−74.681108	−74.772226	−74.845801	−74.902415	−74.942846	−74.967931	−74.978515
$\sigma^{3,2}\pi_+^{1,0}\pi_-^{1,0}\delta_-^{1,0}$	−73.877289	−74.165055	−74.428455	−74.667793	−74.883531	−75.076256	−75.246657
$\sigma^{3,2}\pi_+^{1,0}\pi_-^{2,0}$	−74.412521	−74.639063	−74.819489	−74.956432	−75.055065	−75.123972	−75.174629
$\sigma^{3,2}\pi_+^{1,0}\pi_-^{1,0}\phi_-^{1,0}$	−69.653777	−69.994815	−70.318071	−70.623942	−70.912986	−71.185847	−71.443210

Table S260: Total energies in E_h for the O atom in the def2-TZVP basis set in fully uncontracted form, employing the real-orbital approximation.

	$0.00B_0$	$0.10B_0$	$0.20B_0$	$0.30B_0$	$0.40B_0$	$0.50B_0$	$0.60B_0$
$\sigma^{3,3}\pi_+^{1,0}\pi_-^{1,0}$	−74.816369	−74.907873	−74.982528	−75.040749	−75.083169	−75.110578	−75.123846
$\sigma^{3,2}\pi_+^{1,1}\pi_-^{1,0}$	−74.813025	−74.853348	−74.874523	−74.877137	−74.862081	−74.830441	−74.783382
$\sigma^{3,2}\pi_+^{1,0}\pi_-^{1,1}$	−74.813025	−74.953348	−75.074523	−75.177137	−75.262081	−75.330441	−75.383382
$\sigma^{3,3}\pi_-^{1,1}$	−74.687732	−74.778921	−74.852659	−74.909434	−74.949988	−74.975234	−74.986164
$\sigma^{3,2}\pi_+^{1,0}\pi_-^{1,0}\delta_-^{1,0}$	−73.276894	−73.567206	−73.838249	−74.090330	−74.323919	−74.539603	−74.738029
$\sigma^{3,2}\pi_+^{1,0}\pi_-^{2,0}$	−74.228684	−74.463652	−74.668672	−74.844091	−74.990483	−75.108653	−75.199644
$\sigma^{3,2}\pi_+^{1,0}\pi_-^{1,0}\phi_-^{1,0}$	−69.563712	−69.904799	−70.228190	−70.534262	−70.823584	−71.096856	−71.354836

Table S261: Total energies in E_h for the F atom in the cc-pVDZ basis set in fully uncontracted form, employing the real-orbital approximation.

	$0.00B_0$	$0.10B_0$	$0.20B_0$	$0.30B_0$	$0.40B_0$	$0.50B_0$	$0.60B_0$
$\sigma^{3,2}\pi_+^{1,1}\pi_-^{1,1}$	−99.375362	−99.416547	−99.440160	−99.446365	−99.435435	−99.407744	−99.363760
$\sigma^{3,3}\pi_+^{1,1}\pi_-^{1,0}$	−99.373267	−99.365254	−99.341261	−99.301429	−99.245990	−99.175260	−99.089635
$\sigma^{3,3}\pi_+^{1,0}\pi_-^{1,1}$	−99.373267	−99.465254	−99.541261	−99.601429	−99.645990	−99.675260	−99.689635
$\sigma^{3,2}\pi_+^{1,0}\pi_-^{1,1}\delta_-^{1,0}$	−95.676977	−95.968674	−96.243812	−96.502528	−96.745047	−96.971676	−97.182799
$\sigma^{3,2}\pi_+^{1,0}\pi_-^{2,1}$	−98.061540	−98.301116	−98.519879	−98.717937	−98.895462	−99.052695	−99.189930
$\sigma^{3,2}\pi_+^{1,0}\pi_-^{1,1}\phi_-^{1,0}$							

Table S262: Total energies in E_h for the F atom in the cc-pVTZ basis set in fully uncontracted form, employing the real-orbital approximation.

	$0.00B_0$	$0.10B_0$	$0.20B_0$	$0.30B_0$	$0.40B_0$	$0.50B_0$	$0.60B_0$
$\sigma^{3,2}\pi_+^{1,1}\pi_-^{1,1}$	−99.405546	−99.446561	−99.469702	−99.475244	−99.463634	−99.435469	−99.391465
$\sigma^{3,3}\pi_+^{1,1}\pi_-^{1,0}$	−99.402185	−99.394026	−99.369626	−99.329216	−99.273166	−99.201972	−99.116229
$\sigma^{3,3}\pi_+^{1,0}\pi_-^{1,1}$	−99.402185	−99.494026	−99.569626	−99.629216	−99.673166	−99.701972	−99.716229
$\sigma^{3,2}\pi_+^{1,0}\pi_-^{1,1}\delta_-^{1,0}$	−97.246455	−97.537427	−97.810411	−98.065603	−98.303322	−98.523989	−98.728109
$\sigma^{3,2}\pi_+^{1,0}\pi_-^{2,1}$	−98.409661	−98.647831	−98.862397	−99.053522	−99.221468	−99.366589	−99.489314
$\sigma^{3,2}\pi_+^{1,0}\pi_-^{1,1}\phi_-^{1,0}$	−92.245470	−92.586916	−92.911333	−93.218952	−93.510144	−93.785401	−94.045312

Table S263: Total energies in E_h for the F atom in the cc-pVQZ basis set in fully uncontracted form, employing the real-orbital approximation.

	$0.00B_0$	$0.10B_0$	$0.20B_0$	$0.30B_0$	$0.40B_0$	$0.50B_0$	$0.60B_0$
$\sigma^{3,2}\pi_+^{1,1}\pi_-^{1,1}$	−99.413773	−99.454723	−99.477703	−99.483085	−99.471452	−99.443554	−99.400250
$\sigma^{3,3}\pi_+^{1,1}\pi_-^{1,0}$	−99.410178	−99.401964	−99.377426	−99.336869	−99.280772	−99.209750	−99.124511
$\sigma^{3,3}\pi_+^{1,0}\pi_-^{1,1}$	−99.410178	−99.501964	−99.577426	−99.636869	−99.680772	−99.709750	−99.724511
$\sigma^{3,2}\pi_+^{1,0}\pi_-^{1,1}\delta_-^{1,0}$	−97.797219	−98.087312	−98.357668	−98.608519	−98.840225	−99.053255	−99.248150
$\sigma^{3,2}\pi_+^{1,0}\pi_-^{2,1}$	−98.588603	−98.825289	−99.035425	−99.219242	−99.377114	−99.509551	−99.617187
$\sigma^{3,2}\pi_+^{1,0}\pi_-^{1,1}\phi_-^{1,0}$	−94.962087	−95.303043	−95.625997	−95.931197	−96.219035	−96.490007	−96.744681

Table S264: Total energies in E_h for the F atom in the cc-pV5Z basis set in fully uncontracted form, employing the real-orbital approximation.

	$0.00B_0$	$0.10B_0$	$0.20B_0$	$0.30B_0$	$0.40B_0$	$0.50B_0$	$0.60B_0$
$\sigma^{3,2}\pi_+^{1,1}\pi_-^{1,1}$	−99.416046	−99.456966	−99.479893	−99.485304	−99.473903	−99.446522	−99.404032
$\sigma^{3,3}\pi_+^{1,1}\pi_-^{1,0}$	−99.412401	−99.404159	−99.379570	−99.339016	−99.283070	−99.212417	−99.127792
$\sigma^{3,3}\pi_+^{1,0}\pi_-^{1,1}$	−99.412401	−99.504159	−99.579570	−99.639016	−99.683070	−99.712417	−99.727792
$\sigma^{3,2}\pi_+^{1,0}\pi_-^{1,1}\delta_-^{1,0}$	−98.057634	−98.346825	−98.614490	−98.860888	−99.086422	−99.291599	−99.476996
$\sigma^{3,2}\pi_+^{1,0}\pi_-^{2,1}$	−98.757246	−98.990837	−99.191804	−99.360738	−99.498657	−99.607058	−99.688001
$\sigma^{3,2}\pi_+^{1,0}\pi_-^{1,1}\phi_-^{1,0}$	−96.234172	−96.574278	−96.894682	−97.195632	−97.477498	−97.740734	−97.985832

Table S265: Total energies in E_h for the F atom in the aug-cc-pVDZ basis set in fully uncontracted form, employing the real-orbital approximation.

	$0.00B_0$	$0.10B_0$	$0.20B_0$	$0.30B_0$	$0.40B_0$	$0.50B_0$	$0.60B_0$
$\sigma^{3,2}\pi_+^{1,1}\pi_-^{1,1}$	−99.381284	−99.422099	−99.444722	−99.449625	−99.437440	−99.408837	−99.364472
$\sigma^{3,3}\pi_+^{1,1}\pi_-^{1,0}$	−99.378823	−99.370521	−99.345753	−99.304889	−99.248437	−99.176955	−99.091007
$\sigma^{3,3}\pi_+^{1,0}\pi_-^{1,1}$	−99.378823	−99.470521	−99.545753	−99.604889	−99.648437	−99.676955	−99.691007
$\sigma^{3,2}\pi_+^{1,0}\pi_-^{1,1}\delta_-^{1,0}$	−97.985069	−98.274441	−98.542634	−98.789867	−99.016465	−99.222823	−99.409387
$\sigma^{3,2}\pi_+^{1,0}\pi_-^{2,1}$	−98.842671	−99.070161	−99.253024	−99.392536	−99.491202	−99.553437	−99.586626
$\sigma^{3,2}\pi_+^{1,0}\pi_-^{1,1}\phi_-^{1,0}$							

Table S266: Total energies in E_h for the F atom in the aug-cc-pVTZ basis set in fully uncontracted form, employing the real-orbital approximation.

	$0.00B_0$	$0.10B_0$	$0.20B_0$	$0.30B_0$	$0.40B_0$	$0.50B_0$	$0.60B_0$
$\sigma^{3,2}\pi_+^{1,1}\pi_-^{1,1}$	−99.406909	−99.447791	−99.470617	−99.475871	−99.464212	−99.436378	−99.393149
$\sigma^{3,3}\pi_+^{1,1}\pi_-^{1,0}$	−99.403355	−99.395082	−99.370405	−99.329712	−99.273547	−99.202537	−99.117351
$\sigma^{3,3}\pi_+^{1,0}\pi_-^{1,1}$	−99.403355	−99.495082	−99.570405	−99.629712	−99.673547	−99.702537	−99.717351
$\sigma^{3,2}\pi_+^{1,0}\pi_-^{1,1}\delta_-^{1,0}$	−98.366936	−98.653978	−98.915206	−99.150923	−99.361612	−99.547920	−99.710652
$\sigma^{3,2}\pi_+^{1,0}\pi_-^{2,1}$	−98.888596	−99.113669	−99.289845	−99.420359	−99.511855	−99.575726	−99.626560
$\sigma^{3,2}\pi_+^{1,0}\pi_-^{1,1}\phi_-^{1,0}$	−96.452542	−96.792375	−97.111958	−97.411523	−97.691413	−97.952048	−98.193900

Table S267: Total energies in E_h for the F atom in the aug-cc-pVQZ basis set in fully uncontracted form, employing the real-orbital approximation.

	$0.00B_0$	$0.10B_0$	$0.20B_0$	$0.30B_0$	$0.40B_0$	$0.50B_0$	$0.60B_0$
$\sigma^{3,2}\pi_+^{1,1}\pi_-^{1,1}$	−99.414089	−99.454992	−99.477890	−99.483285	−99.471879	−99.444473	−99.401923
$\sigma^{3,3}\pi_+^{1,1}\pi_-^{1,0}$	−99.410443	−99.402184	−99.377559	−99.336978	−99.281027	−99.210387	−99.125797
$\sigma^{3,3}\pi_+^{1,0}\pi_-^{1,1}$	−99.410443	−99.502184	−99.577559	−99.636978	−99.681027	−99.710387	−99.725797
$\sigma^{3,2}\pi_+^{1,0}\pi_-^{1,1}\delta_-^{1,0}$	−98.551872	−98.836133	−99.089103	−99.311339	−99.503787	−99.667825	−99.805334
$\sigma^{3,2}\pi_+^{1,0}\pi_-^{2,1}$	−98.907247	−99.130147	−99.300775	−99.425737	−99.517910	−99.593945	−99.663936
$\sigma^{3,2}\pi_+^{1,0}\pi_-^{1,1}\phi_-^{1,0}$	−97.402612	−97.740393	−98.053817	−98.343107	−98.608610	−98.850762	−99.070067

Table S268: Total energies in E_h for the F atom in the aug-cc-pV5Z basis set in fully uncontracted form, employing the real-orbital approximation.

	$0.00B_0$	$0.10B_0$	$0.20B_0$	$0.30B_0$	$0.40B_0$	$0.50B_0$	$0.60B_0$
$\sigma^{3,2}\pi_+^{1,1}\pi_-^{1,1}$	−99.416088	−99.457004	−99.479946	−99.485441	−99.474225	−99.447135	−99.405032
$\sigma^{3,3}\pi_+^{1,1}\pi_-^{1,0}$	−99.412426	−99.404177	−99.379589	−99.339093	−99.283307	−99.212942	−99.128743
$\sigma^{3,3}\pi_+^{1,0}\pi_-^{1,1}$	−99.412426	−99.504177	−99.579589	−99.639093	−99.683307	−99.712942	−99.728743
$\sigma^{3,2}\pi_+^{1,0}\pi_-^{1,1}\delta_-^{1,0}$	−98.629298	−98.911423	−99.158106	−99.370309	−99.549733	−99.698975	−99.821721
$\sigma^{3,2}\pi_+^{1,0}\pi_-^{2,1}$	−98.921772	−99.140949	−99.303786	−99.426687	−99.529791	−99.620977	−99.698328
$\sigma^{3,2}\pi_+^{1,0}\pi_-^{1,1}\phi_-^{1,0}$	−97.865656	−98.201037	−98.507274	−98.784633	−99.033541	−99.254553	−99.448346

Table S269: Total energies in E_h for the F atom in the HGBSP1-5 basis set in fully uncontracted form, employing the real-orbital approximation.

	$0.00B_0$	$0.10B_0$	$0.20B_0$	$0.30B_0$	$0.40B_0$	$0.50B_0$	$0.60B_0$
$\sigma^{3,2}\pi_+^{1,1}\pi_-^{1,1}$	−99.413006	−99.453891	−99.476728	−99.482013	−99.470433	−99.442761	−99.399794
$\sigma^{3,3}\pi_+^{1,1}\pi_-^{1,0}$	−99.410374	−99.402139	−99.377578	−99.337081	−99.281202	−99.210576	−99.125868
$\sigma^{3,3}\pi_+^{1,0}\pi_-^{1,1}$	−99.410374	−99.502139	−99.577578	−99.637081	−99.681202	−99.710576	−99.725868
$\sigma^{3,2}\pi_+^{1,0}\pi_-^{1,1}\delta_-^{1,0}$	−98.890502	−99.116063	−99.290579	−99.444275	−99.582110	−99.705127	−99.816799
$\sigma^{3,2}\pi_+^{1,0}\pi_-^{2,1}$	−98.937714	−99.144788	−99.300892	−99.428920	−99.533442	−99.616577	−99.682688
$\sigma^{3,2}\pi_+^{1,0}\pi_-^{1,1}\phi_-^{1,0}$							

Table S270: Total energies in E_h for the F atom in the HGBSP1-7 basis set in fully uncontracted form, employing the real-orbital approximation.

	$0.00B_0$	$0.10B_0$	$0.20B_0$	$0.30B_0$	$0.40B_0$	$0.50B_0$	$0.60B_0$
$\sigma^{3,2}\pi_+^{1,1}\pi_-^{1,1}$	−99.414847	−99.455732	−99.478571	−99.483861	−99.472291	−99.444640	−99.401710
$\sigma^{3,3}\pi_+^{1,1}\pi_-^{1,0}$	−99.412214	−99.403980	−99.379420	−99.338929	−99.283058	−99.212448	−99.127769
$\sigma^{3,3}\pi_+^{1,0}\pi_-^{1,1}$	−99.412214	−99.503980	−99.579420	−99.638929	−99.683058	−99.712448	−99.727769
$\sigma^{3,2}\pi_+^{1,0}\pi_-^{1,1}\delta_-^{1,0}$	−98.892384	−99.118073	−99.292955	−99.446389	−99.584208	−99.708330	−99.820450
$\sigma^{3,2}\pi_+^{1,0}\pi_-^{2,1}$	−98.939600	−99.146662	−99.303490	−99.430815	−99.535771	−99.621204	−99.689037
$\sigma^{3,2}\pi_+^{1,0}\pi_-^{1,1}\phi_-^{1,0}$							

Table S271: Total energies in E_h for the F atom in the HGBSP1-9 basis set in fully uncontracted form, employing the real-orbital approximation.

	$0.00B_0$	$0.10B_0$	$0.20B_0$	$0.30B_0$	$0.40B_0$	$0.50B_0$	$0.60B_0$
$\sigma^{3,2}\pi_+^{1,1}\pi_-^{1,1}$	−99.414869	−99.455754	−99.478594	−99.483883	−99.472313	−99.444663	−99.401734
$\sigma^{3,3}\pi_+^{1,1}\pi_-^{1,0}$	−99.412236	−99.404002	−99.379443	−99.338951	−99.283081	−99.212471	−99.127793
$\sigma^{3,3}\pi_+^{1,0}\pi_-^{1,1}$	−99.412236	−99.504002	−99.579443	−99.638951	−99.683081	−99.712471	−99.727793
$\sigma^{3,2}\pi_+^{1,0}\pi_-^{1,1}\delta_-^{1,0}$	−98.892407	−99.118117	−99.293008	−99.446485	−99.584254	−99.708578	−99.820696
$\sigma^{3,2}\pi_+^{1,0}\pi_-^{2,1}$	−98.939624	−99.146705	−99.303525	−99.431003	−99.535817	−99.621321	−99.689560
$\sigma^{3,2}\pi_+^{1,0}\pi_-^{1,1}\phi_-^{1,0}$							

Table S272: Total energies in E_h for the F atom in the HGBSP2-5 basis set in fully uncontracted form, employing the real-orbital approximation.

	$0.00B_0$	$0.10B_0$	$0.20B_0$	$0.30B_0$	$0.40B_0$	$0.50B_0$	$0.60B_0$
$\sigma^{3,2}\pi_+^{1,1}\pi_-^{1,1}$	−99.414429	−99.455347	−99.478301	−99.483826	−99.472654	−99.445605	−99.403516
$\sigma^{3,3}\pi_+^{1,1}\pi_-^{1,0}$	−99.410772	−99.402525	−99.377947	−99.337481	−99.281745	−99.211438	−99.127284
$\sigma^{3,3}\pi_+^{1,0}\pi_-^{1,1}$	−99.410772	−99.502525	−99.577947	−99.637481	−99.681745	−99.711438	−99.727284
$\sigma^{3,2}\pi_+^{1,0}\pi_-^{1,1}\delta_-^{1,0}$	−98.890936	−99.116531	−99.291126	−99.444959	−99.583009	−99.706349	−99.818480
$\sigma^{3,2}\pi_+^{1,0}\pi_-^{2,1}$	−98.938176	−99.146190	−99.306233	−99.440235	−99.553401	−99.648254	−99.727569
$\sigma^{3,2}\pi_+^{1,0}\pi_-^{1,1}\phi_-^{1,0}$	−98.866551	−99.088188	−99.253935	−99.394347	−99.522244	−99.634805	−99.730021

Table S273: Total energies in E_h for the F atom in the HGBSP2-7 basis set in fully uncontracted form, employing the real-orbital approximation.

	$0.00B_0$	$0.10B_0$	$0.20B_0$	$0.30B_0$	$0.40B_0$	$0.50B_0$	$0.60B_0$
$\sigma^{3,2}\pi_+^{1,1}\pi_-^{1,1}$	−99.416283	−99.457202	−99.480159	−99.485688	−99.474528	−99.447500	−99.405446
$\sigma^{3,3}\pi_+^{1,1}\pi_-^{1,0}$	−99.412616	−99.404370	−99.379794	−99.339332	−99.283605	−99.213315	−99.129189
$\sigma^{3,3}\pi_+^{1,0}\pi_-^{1,1}$	−99.412616	−99.504370	−99.579794	−99.639332	−99.683605	−99.713315	−99.729189
$\sigma^{3,2}\pi_+^{1,0}\pi_-^{1,1}\delta_-^{1,0}$	−98.892824	−99.118546	−99.293508	−99.447079	−99.585114	−99.709558	−99.822134
$\sigma^{3,2}\pi_+^{1,0}\pi_-^{2,1}$	−98.940068	−99.148079	−99.308663	−99.442321	−99.555613	−99.651539	−99.731850
$\sigma^{3,2}\pi_+^{1,0}\pi_-^{1,1}\phi_-^{1,0}$	−98.868439	−99.090447	−99.255848	−99.398684	−99.525235	−99.636622	−99.735121

Table S274: Total energies in E_h for the F atom in the HGBSP2-9 basis set in fully uncontracted form, employing the real-orbital approximation.

	$0.00B_0$	$0.10B_0$	$0.20B_0$	$0.30B_0$	$0.40B_0$	$0.50B_0$	$0.60B_0$
$\sigma^{3,2}\pi_+^{1,1}\pi_-^{1,1}$	−99.416306	−99.457224	−99.480181	−99.485711	−99.474550	−99.447523	−99.405470
$\sigma^{3,3}\pi_+^{1,1}\pi_-^{1,0}$	−99.412639	−99.404392	−99.379816	−99.339355	−99.283628	−99.213338	−99.129213
$\sigma^{3,3}\pi_+^{1,0}\pi_-^{1,1}$	−99.412639	−99.504392	−99.579816	−99.639355	−99.683628	−99.713338	−99.729213
$\sigma^{3,2}\pi_+^{1,0}\pi_-^{1,1}\delta_-^{1,0}$	−98.892847	−99.118590	−99.293561	−99.447175	−99.585160	−99.709805	−99.822379
$\sigma^{3,2}\pi_+^{1,0}\pi_-^{2,1}$	−98.940092	−99.148116	−99.308692	−99.442437	−99.555689	−99.651571	−99.732013
$\sigma^{3,2}\pi_+^{1,0}\pi_-^{1,1}\phi_-^{1,0}$	−98.868462	−99.090471	−99.255871	−99.398989	−99.525237	−99.637035	−99.735778

Table S275: Total energies in E_h for the F atom in the HGBSP3-5 basis set in fully uncontracted form, employing the real-orbital approximation.

	$0.00B_0$	$0.10B_0$	$0.20B_0$	$0.30B_0$	$0.40B_0$	$0.50B_0$	$0.60B_0$
$\sigma^{3,2}\pi_+^{1,1}\pi_-^{1,1}$	−99.414429	−99.455347	−99.478301	−99.483826	−99.472654	−99.445606	−99.403517
$\sigma^{3,3}\pi_+^{1,1}\pi_-^{1,0}$	−99.410772	−99.402525	−99.377947	−99.337481	−99.281745	−99.211438	−99.127285
$\sigma^{3,3}\pi_+^{1,0}\pi_-^{1,1}$	−99.410772	−99.502525	−99.577947	−99.637481	−99.681745	−99.711438	−99.727285
$\sigma^{3,2}\pi_+^{1,0}\pi_-^{1,1}\delta_-^{1,0}$	−98.890936	−99.117735	−99.295029	−99.451664	−99.592773	−99.719338	−99.834255
$\sigma^{3,2}\pi_+^{1,0}\pi_-^{2,1}$	−98.938175	−99.146190	−99.306233	−99.440235	−99.553401	−99.648254	−99.727569
$\sigma^{3,2}\pi_+^{1,0}\pi_-^{1,1}\phi_-^{1,0}$	−98.866550	−99.088188	−99.253935	−99.394347	−99.522244	−99.634805	−99.730022

Table S276: Total energies in E_h for the F atom in the HGBSP3-7 basis set in fully uncontracted form, employing the real-orbital approximation.

	$0.00B_0$	$0.10B_0$	$0.20B_0$	$0.30B_0$	$0.40B_0$	$0.50B_0$	$0.60B_0$
$\sigma^{3,2}\pi_+^{1,1}\pi_-^{1,1}$	−99.416283	−99.457202	−99.480159	−99.485689	−99.474528	−99.447501	−99.405447
$\sigma^{3,3}\pi_+^{1,1}\pi_-^{1,0}$	−99.412616	−99.404370	−99.379794	−99.339332	−99.283605	−99.213315	−99.129190
$\sigma^{3,3}\pi_+^{1,0}\pi_-^{1,1}$	−99.412616	−99.504370	−99.579794	−99.639332	−99.683605	−99.713315	−99.729190
$\sigma^{3,2}\pi_+^{1,0}\pi_-^{1,1}\delta_-^{1,0}$	−98.892824	−99.119744	−99.297310	−99.453825	−99.594826	−99.722276	−99.837646
$\sigma^{3,2}\pi_+^{1,0}\pi_-^{2,1}$	−98.940068	−99.148079	−99.308663	−99.442321	−99.555613	−99.651540	−99.731850
$\sigma^{3,2}\pi_+^{1,0}\pi_-^{1,1}\phi_-^{1,0}$	−98.868439	−99.090447	−99.255848	−99.398684	−99.525235	−99.636622	−99.735121

Table S277: Total energies in E_h for the F atom in the HGBSP3-9 basis set in fully uncontracted form, employing the real-orbital approximation.

	$0.00B_0$	$0.10B_0$	$0.20B_0$	$0.30B_0$	$0.40B_0$	$0.50B_0$	$0.60B_0$
$\sigma^{3,2}\pi_+^{1,1}\pi_-^{1,1}$	−99.416306	−99.457225	−99.480181	−99.485711	−99.474551	−99.447524	−99.405471
$\sigma^{3,3}\pi_+^{1,1}\pi_-^{1,0}$	−99.412639	−99.404392	−99.379816	−99.339355	−99.283628	−99.213338	−99.129214
$\sigma^{3,3}\pi_+^{1,0}\pi_-^{1,1}$	−99.412639	−99.504392	−99.579816	−99.639355	−99.683628	−99.713338	−99.729214
$\sigma^{3,2}\pi_+^{1,0}\pi_-^{1,1}\delta_-^{1,0}$	−98.892848	−99.119784	−99.297356	−99.453911	−99.594875	−99.722442	−99.837844
$\sigma^{3,2}\pi_+^{1,0}\pi_-^{2,1}$	−98.940092	−99.148116	−99.308692	−99.442437	−99.555689	−99.651571	−99.732013
$\sigma^{3,2}\pi_+^{1,0}\pi_-^{1,1}\phi_-^{1,0}$	−98.868462	−99.090471	−99.255871	−99.398989	−99.525237	−99.637035	−99.735778

Table S278: Total energies in E_h for the F atom in the AHGBSP1-5 basis set in fully uncontracted form, employing the real-orbital approximation.

	$0.00B_0$	$0.10B_0$	$0.20B_0$	$0.30B_0$	$0.40B_0$	$0.50B_0$	$0.60B_0$
$\sigma^{3,2}\pi_+^{1,1}\pi_-^{1,1}$	−99.413008	−99.453892	−99.476730	−99.482015	−99.470435	−99.442764	−99.399799
$\sigma^{3,3}\pi_+^{1,1}\pi_-^{1,0}$	−99.410376	−99.402141	−99.377579	−99.337083	−99.281204	−99.210578	−99.125872
$\sigma^{3,3}\pi_+^{1,0}\pi_-^{1,1}$	−99.410376	−99.502141	−99.577579	−99.637083	−99.681204	−99.710578	−99.725872
$\sigma^{3,2}\pi_+^{1,0}\pi_-^{1,1}\delta_-^{1,0}$	−98.890507	−99.116076	−99.290593	−99.444284	−99.582117	−99.705140	−99.816814
$\sigma^{3,2}\pi_+^{1,0}\pi_-^{2,1}$	−98.937720	−99.144793	−99.301039	−99.428955	−99.533479	−99.616765	−99.682965
$\sigma^{3,2}\pi_+^{1,0}\pi_-^{1,1}\phi_-^{1,0}$							

Table S279: Total energies in E_h for the F atom in the AHGBSP1-7 basis set in fully uncontracted form, employing the real-orbital approximation.

	$0.00B_0$	$0.10B_0$	$0.20B_0$	$0.30B_0$	$0.40B_0$	$0.50B_0$	$0.60B_0$
$\sigma^{3,2}\pi_+^{1,1}\pi_-^{1,1}$	−99.414847	−99.455732	−99.478571	−99.483861	−99.472291	−99.444640	−99.401710
$\sigma^{3,3}\pi_+^{1,1}\pi_-^{1,0}$	−99.412214	−99.403980	−99.379421	−99.338929	−99.283058	−99.212449	−99.127769
$\sigma^{3,3}\pi_+^{1,0}\pi_-^{1,1}$	−99.412214	−99.503980	−99.579421	−99.638929	−99.683058	−99.712449	−99.727769
$\sigma^{3,2}\pi_+^{1,0}\pi_-^{1,1}\delta_-^{1,0}$	−98.892384	−99.118074	−99.292956	−99.446392	−99.584209	−99.708333	−99.820453
$\sigma^{3,2}\pi_+^{1,0}\pi_-^{2,1}$	−98.939600	−99.146670	−99.303492	−99.430845	−99.535776	−99.621212	−99.689077
$\sigma^{3,2}\pi_+^{1,0}\pi_-^{1,1}\phi_-^{1,0}$							

Table S280: Total energies in E_h for the F atom in the AHGBSP1-9 basis set in fully uncontracted form, employing the real-orbital approximation.

	$0.00B_0$	$0.10B_0$	$0.20B_0$	$0.30B_0$	$0.40B_0$	$0.50B_0$	$0.60B_0$
$\sigma^{3,2}\pi_+^{1,1}\pi_-^{1,1}$	−99.414869	−99.455754	−99.478594	−99.483883	−99.472313	−99.444663	−99.401734
$\sigma^{3,3}\pi_+^{1,1}\pi_-^{1,0}$	−99.412236	−99.404002	−99.379443	−99.338951	−99.283081	−99.212471	−99.127793
$\sigma^{3,3}\pi_+^{1,0}\pi_-^{1,1}$	−99.412236	−99.504002	−99.579443	−99.638951	−99.683081	−99.712471	−99.727793
$\sigma^{3,2}\pi_+^{1,0}\pi_-^{1,1}\delta_-^{1,0}$	−98.892407	−99.118117	−99.293008	−99.446485	−99.584254	−99.708578	−99.820696
$\sigma^{3,2}\pi_+^{1,0}\pi_-^{2,1}$	−98.939624	−99.146705	−99.303525	−99.431004	−99.535818	−99.621321	−99.689562
$\sigma^{3,2}\pi_+^{1,0}\pi_-^{1,1}\phi_-^{1,0}$							

Table S281: Total energies in E_h for the F atom in the AHGBSP2-5 basis set in fully uncontracted form, employing the real-orbital approximation.

	$0.00B_0$	$0.10B_0$	$0.20B_0$	$0.30B_0$	$0.40B_0$	$0.50B_0$	$0.60B_0$
$\sigma^{3,2}\pi_+^{1,1}\pi_-^{1,1}$	−99.414431	−99.455349	−99.478303	−99.483827	−99.472656	−99.445608	−99.403520
$\sigma^{3,3}\pi_+^{1,1}\pi_-^{1,0}$	−99.410774	−99.402527	−99.377949	−99.337483	−99.281747	−99.211441	−99.127288
$\sigma^{3,3}\pi_+^{1,0}\pi_-^{1,1}$	−99.410774	−99.502527	−99.577949	−99.637483	−99.681747	−99.711441	−99.727288
$\sigma^{3,2}\pi_+^{1,0}\pi_-^{1,1}\delta_-^{1,0}$	−98.890941	−99.116543	−99.291140	−99.444969	−99.583016	−99.706362	−99.818495
$\sigma^{3,2}\pi_+^{1,0}\pi_-^{2,1}$	−98.938182	−99.146198	−99.306330	−99.440290	−99.553407	−99.648305	−99.727696
$\sigma^{3,2}\pi_+^{1,0}\pi_-^{1,1}\phi_-^{1,0}$	−98.866556	−99.088197	−99.253940	−99.394361	−99.522252	−99.634812	−99.730033

Table S282: Total energies in E_h for the F atom in the AHGBSP2-7 basis set in fully uncontracted form, employing the real-orbital approximation.

	$0.00B_0$	$0.10B_0$	$0.20B_0$	$0.30B_0$	$0.40B_0$	$0.50B_0$	$0.60B_0$
$\sigma^{3,2}\pi_+^{1,1}\pi_-^{1,1}$	−99.416283	−99.457202	−99.480159	−99.485688	−99.474528	−99.447500	−99.405446
$\sigma^{3,3}\pi_+^{1,1}\pi_-^{1,0}$	−99.412616	−99.404370	−99.379794	−99.339332	−99.283605	−99.213315	−99.129189
$\sigma^{3,3}\pi_+^{1,0}\pi_-^{1,1}$	−99.412616	−99.504370	−99.579794	−99.639332	−99.683605	−99.713315	−99.729189
$\sigma^{3,2}\pi_+^{1,0}\pi_-^{1,1}\delta_-^{1,0}$	−98.892824	−99.118548	−99.293509	−99.447082	−99.585115	−99.709561	−99.822137
$\sigma^{3,2}\pi_+^{1,0}\pi_-^{2,1}$	−98.940068	−99.148084	−99.308663	−99.442336	−99.555621	−99.651539	−99.731856
$\sigma^{3,2}\pi_+^{1,0}\pi_-^{1,1}\phi_-^{1,0}$	−98.868439	−99.090447	−99.255848	−99.398687	−99.525235	−99.636624	−99.735123

Table S283: Total energies in E_h for the F atom in the AHGBSP2-9 basis set in fully uncontracted form, employing the real-orbital approximation.

	$0.00B_0$	$0.10B_0$	$0.20B_0$	$0.30B_0$	$0.40B_0$	$0.50B_0$	$0.60B_0$
$\sigma^{3,2}\pi_+^{1,1}\pi_-^{1,1}$	−99.416306	−99.457224	−99.480181	−99.485711	−99.474550	−99.447523	−99.405470
$\sigma^{3,3}\pi_+^{1,1}\pi_-^{1,0}$	−99.412639	−99.404392	−99.379816	−99.339355	−99.283628	−99.213338	−99.129213
$\sigma^{3,3}\pi_+^{1,0}\pi_-^{1,1}$	−99.412639	−99.504392	−99.579816	−99.639355	−99.683628	−99.713338	−99.729213
$\sigma^{3,2}\pi_+^{1,0}\pi_-^{1,1}\delta_-^{1,0}$	−98.892847	−99.118590	−99.293561	−99.447175	−99.585160	−99.709806	−99.822379
$\sigma^{3,2}\pi_+^{1,0}\pi_-^{2,1}$	−98.940092	−99.148116	−99.308692	−99.442437	−99.555689	−99.651571	−99.732013
$\sigma^{3,2}\pi_+^{1,0}\pi_-^{1,1}\phi_-^{1,0}$	−98.868462	−99.090471	−99.255871	−99.398989	−99.525237	−99.637035	−99.735778

Table S284: Total energies in E_h for the F atom in the AHGBSP3-5 basis set in fully uncontracted form, employing the real-orbital approximation.

	$0.00B_0$	$0.10B_0$	$0.20B_0$	$0.30B_0$	$0.40B_0$	$0.50B_0$	$0.60B_0$
$\sigma^{3,2}\pi_+^{1,1}\pi_-^{1,1}$	−99.414431	−99.455349	−99.478303	−99.483828	−99.472656	−99.445609	−99.403521
$\sigma^{3,3}\pi_+^{1,1}\pi_-^{1,0}$	−99.410774	−99.402527	−99.377949	−99.337483	−99.281747	−99.211441	−99.127289
$\sigma^{3,3}\pi_+^{1,0}\pi_-^{1,1}$	−99.410774	−99.502527	−99.577949	−99.637483	−99.681747	−99.711441	−99.727289
$\sigma^{3,2}\pi_+^{1,0}\pi_-^{1,1}\delta_-^{1,0}$	−98.890941	−99.117747	−99.295040	−99.451673	−99.592779	−99.719348	−99.834268
$\sigma^{3,2}\pi_+^{1,0}\pi_-^{2,1}$	−98.938182	−99.146198	−99.306330	−99.440290	−99.553407	−99.648305	−99.727696
$\sigma^{3,2}\pi_+^{1,0}\pi_-^{1,1}\phi_-^{1,0}$	−98.866555	−99.088197	−99.253940	−99.394361	−99.522252	−99.634812	−99.730033

Table S285: Total energies in E_h for the F atom in the AHGBSP3-7 basis set in fully uncontracted form, employing the real-orbital approximation.

	$0.00B_0$	$0.10B_0$	$0.20B_0$	$0.30B_0$	$0.40B_0$	$0.50B_0$	$0.60B_0$
$\sigma^{3,2}\pi_+^{1,1}\pi_-^{1,1}$	−99.416283	−99.457202	−99.480159	−99.485689	−99.474528	−99.447501	−99.405447
$\sigma^{3,3}\pi_+^{1,1}\pi_-^{1,0}$	−99.412616	−99.404370	−99.379794	−99.339333	−99.283605	−99.213315	−99.129190
$\sigma^{3,3}\pi_+^{1,0}\pi_-^{1,1}$	−99.412616	−99.504370	−99.579794	−99.639333	−99.683605	−99.713315	−99.729190
$\sigma^{3,2}\pi_+^{1,0}\pi_-^{1,1}\delta_-^{1,0}$	−98.892824	−99.119745	−99.297311	−99.453826	−99.594827	−99.722278	−99.837648
$\sigma^{3,2}\pi_+^{1,0}\pi_-^{2,1}$	−98.940068	−99.148084	−99.308663	−99.442336	−99.555622	−99.651540	−99.731857
$\sigma^{3,2}\pi_+^{1,0}\pi_-^{1,1}\phi_-^{1,0}$	−98.868439	−99.090447	−99.255848	−99.398688	−99.525235	−99.636624	−99.735124

Table S286: Total energies in E_h for the F atom in the AHGBSP3-9 basis set in fully uncontracted form, employing the real-orbital approximation.

	$0.00B_0$	$0.10B_0$	$0.20B_0$	$0.30B_0$	$0.40B_0$	$0.50B_0$	$0.60B_0$
$\sigma^{3,2}\pi_+^{1,1}\pi_-^{1,1}$	−99.416306	−99.457225	−99.480181	−99.485711	−99.474551	−99.447524	−99.405471
$\sigma^{3,3}\pi_+^{1,1}\pi_-^{1,0}$	−99.412639	−99.404392	−99.379816	−99.339355	−99.283628	−99.213338	−99.129214
$\sigma^{3,3}\pi_+^{1,0}\pi_-^{1,1}$	−99.412639	−99.504392	−99.579816	−99.639355	−99.683628	−99.713338	−99.729214
$\sigma^{3,2}\pi_+^{1,0}\pi_-^{1,1}\delta_-^{1,0}$	−98.892848	−99.119784	−99.297356	−99.453911	−99.594875	−99.722442	−99.837844
$\sigma^{3,2}\pi_+^{1,0}\pi_-^{2,1}$	−98.940092	−99.148116	−99.308692	−99.442437	−99.555689	−99.651571	−99.732013
$\sigma^{3,2}\pi_+^{1,0}\pi_-^{1,1}\phi_-^{1,0}$	−98.868462	−99.090471	−99.255871	−99.398989	−99.525238	−99.637036	−99.735778

Table S287: Total energies in E_h for the F atom in the 6-311++G(3df,3pd) basis set in fully uncontracted form, employing the real-orbital approximation.

	$0.00B_0$	$0.10B_0$	$0.20B_0$	$0.30B_0$	$0.40B_0$	$0.50B_0$	$0.60B_0$
$\sigma^{3,2}\pi_+^{1,1}\pi_-^{1,1}$	−99.402173	−99.443043	−99.465814	−99.470933	−99.459031	−99.430817	−99.387016
$\sigma^{3,3}\pi_+^{1,1}\pi_-^{1,0}$	−99.398752	−99.390481	−99.365794	−99.325047	−99.268745	−99.197475	−99.111849
$\sigma^{3,3}\pi_+^{1,0}\pi_-^{1,1}$	−99.398752	−99.490481	−99.565794	−99.625047	−99.668745	−99.697475	−99.711849
$\sigma^{3,2}\pi_+^{1,0}\pi_-^{1,1}\delta_-^{1,0}$	−98.063344	−98.352556	−98.620270	−98.866706	−99.092197	−99.297158	−99.482058
$\sigma^{3,2}\pi_+^{1,0}\pi_-^{2,1}$	−98.831581	−99.061559	−99.251859	−99.403645	−99.519080	−99.601669	−99.656669
$\sigma^{3,2}\pi_+^{1,0}\pi_-^{1,1}\phi_-^{1,0}$	−92.473374	−92.814649	−93.138624	−93.445701	−93.736428	−94.011400	−94.271210

Table S288: Total energies in E_h for the F atom in the def2-TZVP basis set in fully uncontracted form, employing the real-orbital approximation.

	$0.00B_0$	$0.10B_0$	$0.20B_0$	$0.30B_0$	$0.40B_0$	$0.50B_0$	$0.60B_0$
$\sigma^{3,2}\pi_+^{1,1}\pi_-^{1,1}$	−99.412698	−99.453642	−99.476601	−99.481939	−99.470232	−99.442215	−99.398729
$\sigma^{3,3}\pi_+^{1,1}\pi_-^{1,0}$	−99.409345	−99.401131	−99.376591	−99.336023	−99.279895	−99.208807	−99.123448
$\sigma^{3,3}\pi_+^{1,0}\pi_-^{1,1}$	−99.409345	−99.501131	−99.576591	−99.636023	−99.679895	−99.708807	−99.723448
$\sigma^{3,2}\pi_+^{1,0}\pi_-^{1,1}\delta_-^{1,0}$	−97.252671	−97.543629	−97.816587	−98.071790	−98.309618	−98.530562	−98.735177
$\sigma^{3,2}\pi_+^{1,0}\pi_-^{2,1}$	−98.588053	−98.824729	−99.034836	−99.218598	−99.376382	−99.508684	−99.616124
$\sigma^{3,2}\pi_+^{1,0}\pi_-^{1,1}\phi_-^{1,0}$	−92.251927	−92.593296	−92.917510	−93.224876	−93.515878	−93.791125	−94.051308

Table S289: Total energies in E_h for the Ne atom in the cc-pVDZ basis set in fully uncontracted form, employing the real-orbital approximation.

	$0.00B_0$	$0.10B_0$	$0.20B_0$	$0.30B_0$	$0.40B_0$	$0.50B_0$	$0.60B_0$
$\sigma^{3,3}\pi_+^{1,1}\pi_-^{1,1}$	−128.488776	−128.481214	−128.458558	−128.420896	−128.368374	−128.301193	−128.219606
$\sigma^{4,2}\pi_+^{1,1}\pi_-^{1,1}$	−126.865877	−126.957842	−127.033762	−127.093719	−127.137844	−127.166319	−127.179373
$\sigma^{4,3}\pi_+^{1,0}\pi_-^{1,1}$	−126.800245	−126.942804	−127.070502	−127.183407	−127.281630	−127.365325	−127.434683
$\sigma^{3,3}\pi_+^{1,0}\pi_-^{2,1}$	−126.800868	−126.991982	−127.165343	−127.321008	−127.459073	−127.579668	−127.682957
$\sigma^{3,3}\pi_+^{1,0}\pi_-^{1,1}\delta_-^{1,0}$	−123.494431	−123.737280	−123.965854	−124.180226	−124.380517	−124.566895	−124.739571
$\sigma^{3,3}\pi_+^{1,0}\pi_-^{1,1}\phi_-^{1,0}$							

Table S290: Total energies in E_h for the Ne atom in the cc-pVTZ basis set in fully uncontracted form, employing the real-orbital approximation.

	$0.00B_0$	$0.10B_0$	$0.20B_0$	$0.30B_0$	$0.40B_0$	$0.50B_0$	$0.60B_0$
$\sigma^{3,3}\pi_+^{1,1}\pi_-^{1,1}$	−128.531862	−128.524146	−128.501049	−128.462718	−128.409395	−128.341407	−128.259159
$\sigma^{4,2}\pi_+^{1,1}\pi_-^{1,1}$	−127.298272	−127.389732	−127.464154	−127.521652	−127.562419	−127.586713	−127.594850
$\sigma^{4,3}\pi_+^{1,0}\pi_-^{1,1}$	−127.248724	−127.390760	−127.516902	−127.627248	−127.721955	−127.801239	−127.865364
$\sigma^{3,3}\pi_+^{1,0}\pi_-^{2,1}$	−127.248950	−127.438894	−127.608757	−127.758629	−127.888655	−127.999035	−128.090018
$\sigma^{3,3}\pi_+^{1,0}\pi_-^{1,1}\delta_-^{1,0}$	−125.716931	−125.959177	−126.185951	−126.397362	−126.593587	−126.774864	−126.941488
$\sigma^{3,3}\pi_+^{1,0}\pi_-^{1,1}\phi_-^{1,0}$	−119.039533	−119.332160	−119.610085	−119.873432	−120.122405	−120.357279	−120.578393

Table S291: Total energies in E_h for the Ne atom in the cc-pVQZ basis set in fully uncontracted form, employing the real-orbital approximation.

	$0.00B_0$	$0.10B_0$	$0.20B_0$	$0.30B_0$	$0.40B_0$	$0.50B_0$	$0.60B_0$
$\sigma^{3,3}\pi_+^{1,1}\pi_-^{1,1}$	−128.543470	−128.535699	−128.512454	−128.473934	−128.420461	−128.352457	−128.270427
$\sigma^{4,2}\pi_+^{1,1}\pi_-^{1,1}$	−127.514949	−127.605876	−127.678707	−127.733580	−127.770721	−127.790427	−127.793056
$\sigma^{4,3}\pi_+^{1,0}\pi_-^{1,1}$	−127.476924	−127.618403	−127.742879	−127.850468	−127.941358	−128.015796	−128.074082
$\sigma^{3,3}\pi_+^{1,0}\pi_-^{2,1}$	−127.477018	−127.665734	−127.831926	−127.975721	−128.097328	−128.197034	−128.275198
$\sigma^{3,3}\pi_+^{1,0}\pi_-^{1,1}\delta_-^{1,0}$	−126.436761	−126.678306	−126.902986	−127.110929	−127.302344	−127.477507	−127.636749
$\sigma^{3,3}\pi_+^{1,0}\pi_-^{1,1}\phi_-^{1,0}$	−122.638892	−122.931155	−123.207993	−123.469547	−123.716040	−123.947770	−124.165089

Table S292: Total energies in E_h for the Ne atom in the cc-pV5Z basis set in fully uncontracted form, employing the real-orbital approximation.

	$0.00B_0$	$0.10B_0$	$0.20B_0$	$0.30B_0$	$0.40B_0$	$0.50B_0$	$0.60B_0$
$\sigma^{3,3}\pi_+^{1,1}\pi_-^{1,1}$	−128.546770	−128.538972	−128.515669	−128.477119	−128.423722	−128.355977	−128.274443
$\sigma^{4,2}\pi_+^{1,1}\pi_-^{1,1}$	−127.716283	−127.806028	−127.875328	−127.924369	−127.953452	−127.962980	−127.953444
$\sigma^{4,3}\pi_+^{1,0}\pi_-^{1,1}$	−127.691210	−127.831460	−127.952264	−128.053782	−128.136276	−128.200096	−128.245676
$\sigma^{3,3}\pi_+^{1,0}\pi_-^{2,1}$	−127.691225	−127.877361	−128.035874	−128.167085	−128.271536	−128.350013	−128.403569
$\sigma^{3,3}\pi_+^{1,0}\pi_-^{1,1}\delta_-^{1,0}$	−126.780171	−127.020988	−127.243490	−127.447827	−127.634235	−127.803020	−127.954543
$\sigma^{3,3}\pi_+^{1,0}\pi_-^{1,1}\phi_-^{1,0}$	−124.334526	−124.626157	−124.901098	−125.159496	−125.401577	−125.627628	−125.837980

Table S293: Total energies in E_h for the Ne atom in the aug-cc-pVDZ basis set in fully uncontracted form, employing the real-orbital approximation.

	$0.00B_0$	$0.10B_0$	$0.20B_0$	$0.30B_0$	$0.40B_0$	$0.50B_0$	$0.60B_0$
$\sigma^{3,3}\pi_+^{1,1}\pi_-^{1,1}$	−128.496519	−128.488656	−128.465161	−128.426291	−128.372415	−128.303962	−128.221375
$\sigma^{4,2}\pi_+^{1,1}\pi_-^{1,1}$	−127.856195	−127.942764	−128.002557	−128.035828	−128.043002	−128.024680	−127.981664
$\sigma^{4,3}\pi_+^{1,0}\pi_-^{1,1}$	−127.853507	−127.990615	−128.102014	−128.187936	−128.248765	−128.285054	−128.297540
$\sigma^{3,3}\pi_+^{1,0}\pi_-^{2,1}$	−127.785398	−127.966796	−128.111188	−128.219195	−128.291966	−128.331374	−128.340347
$\sigma^{3,3}\pi_+^{1,0}\pi_-^{1,1}\delta_-^{1,0}$	−126.593645	−126.834803	−127.058322	−127.264329	−127.453017	−127.624631	−127.779445
$\sigma^{3,3}\pi_+^{1,0}\pi_-^{1,1}\phi_-^{1,0}$							

Table S294: Total energies in E_h for the Ne atom in the aug-cc-pVTZ basis set in fully uncontracted form, employing the real-orbital approximation.

	$0.00B_0$	$0.10B_0$	$0.20B_0$	$0.30B_0$	$0.40B_0$	$0.50B_0$	$0.60B_0$
$\sigma^{3,3}\pi_+^{1,1}\pi_-^{1,1}$	−128.533287	−128.525459	−128.502069	−128.463380	−128.409777	−128.341714	−128.259683
$\sigma^{4,2}\pi_+^{1,1}\pi_-^{1,1}$	−127.921455	−128.007215	−128.064707	−128.094592	−128.098031	−128.076787	−128.033369
$\sigma^{4,3}\pi_+^{1,0}\pi_-^{1,1}$	−127.917605	−128.053823	−128.162677	−128.244778	−128.301202	−128.333590	−128.344283
$\sigma^{3,3}\pi_+^{1,0}\pi_-^{2,1}$	−127.851725	−128.031056	−128.169526	−128.268687	−128.331545	−128.363189	−128.371365
$\sigma^{3,3}\pi_+^{1,0}\pi_-^{1,1}\delta_-^{1,0}$	−127.144948	−127.384246	−127.602197	−127.798963	−127.974805	−128.130070	−128.265186
$\sigma^{3,3}\pi_+^{1,0}\pi_-^{1,1}\phi_-^{1,0}$	−124.235353	−124.527003	−124.802004	−125.060498	−125.302700	−125.528880	−125.739346

Table S295: Total energies in E_h for the Ne atom in the aug-cc-pVQZ basis set in fully uncontracted form, employing the real-orbital approximation.

	$0.00B_0$	$0.10B_0$	$0.20B_0$	$0.30B_0$	$0.40B_0$	$0.50B_0$	$0.60B_0$
$\sigma^{3,3}\pi_+^{1,1}\pi_-^{1,1}$	−128.543757	−128.535944	−128.512605	−128.474017	−128.420588	−128.352810	−128.271223
$\sigma^{4,2}\pi_+^{1,1}\pi_-^{1,1}$	−127.945897	−128.031070	−128.087007	−128.115015	−128.117409	−128.097640	−128.060224
$\sigma^{4,3}\pi_+^{1,0}\pi_-^{1,1}$	−127.941914	−128.077479	−128.184568	−128.264407	−128.319174	−128.352155	−128.367744
$\sigma^{3,3}\pi_+^{1,0}\pi_-^{2,1}$	−127.878282	−128.055757	−128.189141	−128.281606	−128.339319	−128.371968	−128.390793
$\sigma^{3,3}\pi_+^{1,0}\pi_-^{1,1}\delta_-^{1,0}$	−127.396561	−127.633716	−127.845275	−128.031512	−128.192888	−128.330052	−128.443854
$\sigma^{3,3}\pi_+^{1,0}\pi_-^{1,1}\phi_-^{1,0}$	−125.640169	−125.930450	−126.201337	−126.452964	−126.685540	−126.899334	−127.094660

Table S296: Total energies in E_h for the Ne atom in the aug-cc-pV5Z basis set in fully uncontracted form, employing the real-orbital approximation.

	$0.00B_0$	$0.10B_0$	$0.20B_0$	$0.30B_0$	$0.40B_0$	$0.50B_0$	$0.60B_0$
$\sigma^{3,3}\pi_+^{1,1}\pi_-^{1,1}$	−128.546786	−128.538983	−128.515680	−128.477171	−128.423894	−128.356371	−128.275176
$\sigma^{4,2}\pi_+^{1,1}\pi_-^{1,1}$	−127.962424	−128.046785	−128.100624	−128.126278	−128.127723	−128.110260	−128.079315
$\sigma^{4,3}\pi_+^{1,0}\pi_-^{1,1}$	−127.958423	−128.093126	−128.197953	−128.275128	−128.328481	−128.363242	−128.384998
$\sigma^{3,3}\pi_+^{1,0}\pi_-^{2,1}$	−127.901807	−128.075402	−128.199106	−128.282352	−128.340191	−128.385230	−128.420435
$\sigma^{3,3}\pi_+^{1,0}\pi_-^{1,1}\delta_-^{1,0}$	−127.527034	−127.762081	−127.967383	−128.143426	−128.291049	−128.411493	−128.506477
$\sigma^{3,3}\pi_+^{1,0}\pi_-^{1,1}\phi_-^{1,0}$	−126.522985	−126.810939	−127.074852	−127.314877	−127.531254	−127.724301	−127.894402

Table S297: Total energies in E_h for the Ne atom in the HGBSP1-5 basis set in fully uncontracted form, employing the real-orbital approximation.

	$0.00B_0$	$0.10B_0$	$0.20B_0$	$0.30B_0$	$0.40B_0$	$0.50B_0$	$0.60B_0$
$\sigma^{3,3}\pi_+^{1,1}\pi_-^{1,1}$	−128.544666	−128.536864	−128.513555	−128.475011	−128.421631	−128.353889	−128.272300
$\sigma^{4,2}\pi_+^{1,1}\pi_-^{1,1}$	−127.990045	−128.070475	−128.117009	−128.138923	−128.142501	−128.130997	−128.106095
$\sigma^{4,3}\pi_+^{1,0}\pi_-^{1,1}$	−127.987143	−128.117725	−128.214787	−128.287826	−128.343334	−128.384663	−128.413535
$\sigma^{3,3}\pi_+^{1,0}\pi_-^{2,1}$	−127.924416	−128.084380	−128.196660	−128.281502	−128.346477	−128.392073	−128.419705
$\sigma^{3,3}\pi_+^{1,0}\pi_-^{1,1}\delta_-^{1,0}$	−127.873831	−128.050405	−128.177557	−128.283153	−128.375505	−128.454363	−128.520510
$\sigma^{3,3}\pi_+^{1,0}\pi_-^{1,1}\phi_-^{1,0}$							

Table S298: Total energies in E_h for the Ne atom in the HGBSP1-7 basis set in fully uncontracted form, employing the real-orbital approximation.

	$0.00B_0$	$0.10B_0$	$0.20B_0$	$0.30B_0$	$0.40B_0$	$0.50B_0$	$0.60B_0$
$\sigma^{3,3}\pi_+^{1,1}\pi_-^{1,1}$	−128.547069	−128.539267	−128.515960	−128.477420	−128.424045	−128.356314	−128.274743
$\sigma^{4,2}\pi_+^{1,1}\pi_-^{1,1}$	−127.995849	−128.074825	−128.119704	−128.141598	−128.145942	−128.135131	−128.110532
$\sigma^{4,3}\pi_+^{1,0}\pi_-^{1,1}$	−127.992956	−128.122070	−128.217472	−128.290511	−128.346796	−128.388814	−128.417983
$\sigma^{3,3}\pi_+^{1,0}\pi_-^{2,1}$	−127.926891	−128.086897	−128.199625	−128.284806	−128.348821	−128.395061	−128.425006
$\sigma^{3,3}\pi_+^{1,0}\pi_-^{1,1}\delta_-^{1,0}$	−127.876277	−128.053059	−128.180101	−128.286448	−128.378097	−128.457080	−128.524382
$\sigma^{3,3}\pi_+^{1,0}\pi_-^{1,1}\phi_-^{1,0}$							

Table S299: Total energies in E_h for the Ne atom in the HGBSP1-9 basis set in fully uncontracted form, employing the real-orbital approximation.

	$0.00B_0$	$0.10B_0$	$0.20B_0$	$0.30B_0$	$0.40B_0$	$0.50B_0$	$0.60B_0$
$\sigma^{3,3}\pi_+^{1,1}\pi_-^{1,1}$	−128.547098	−128.539296	−128.515989	−128.477449	−128.424074	−128.356343	−128.274773
$\sigma^{4,2}\pi_+^{1,1}\pi_-^{1,1}$	−127.997136	−128.075320	−128.119751	−128.141704	−128.146086	−128.135266	−128.110618
$\sigma^{4,3}\pi_+^{1,0}\pi_-^{1,1}$	−127.994247	−128.122562	−128.217519	−128.290619	−128.346938	−128.388950	−128.418074
$\sigma^{3,3}\pi_+^{1,0}\pi_-^{2,1}$	−127.926921	−128.086931	−128.199660	−128.284931	−128.349035	−128.395117	−128.425088
$\sigma^{3,3}\pi_+^{1,0}\pi_-^{1,1}\delta_-^{1,0}$	−127.876307	−128.053088	−128.180135	−128.286605	−128.378185	−128.457138	−128.524659
$\sigma^{3,3}\pi_+^{1,0}\pi_-^{1,1}\phi_-^{1,0}$							

Table S300: Total energies in E_h for the Ne atom in the HGBSP2-5 basis set in fully uncontracted form, employing the real-orbital approximation.

	$0.00B_0$	$0.10B_0$	$0.20B_0$	$0.30B_0$	$0.40B_0$	$0.50B_0$	$0.60B_0$
$\sigma^{3,3}\pi_+^{1,1}\pi_-^{1,1}$	−128.544666	−128.536865	−128.513572	−128.475091	−128.421864	−128.354412	−128.273295
$\sigma^{4,2}\pi_+^{1,1}\pi_-^{1,1}$	−127.991519	−128.071956	−128.118511	−128.140479	−128.144160	−128.132830	−128.108193
$\sigma^{4,3}\pi_+^{1,0}\pi_-^{1,1}$	−127.987557	−128.118136	−128.215192	−128.288226	−128.343742	−128.385111	−128.414076
$\sigma^{3,3}\pi_+^{1,0}\pi_-^{2,1}$	−127.924820	−128.085453	−128.200767	−128.290547	−128.361725	−128.415829	−128.454438
$\sigma^{3,3}\pi_+^{1,0}\pi_-^{1,1}\delta_-^{1,0}$	−127.874242	−128.050799	−128.177931	−128.283528	−128.375926	−128.454903	−128.521272
$\sigma^{3,3}\pi_+^{1,0}\pi_-^{1,1}\phi_-^{1,0}$	−127.849652	−128.022727	−128.139963	−128.236387	−128.315074	−128.384995	−128.441636

Table S301: Total energies in E_h for the Ne atom in the HGBSP2-7 basis set in fully uncontracted form, employing the real-orbital approximation.

	$0.00B_0$	$0.10B_0$	$0.20B_0$	$0.30B_0$	$0.40B_0$	$0.50B_0$	$0.60B_0$
$\sigma^{3,3}\pi_+^{1,1}\pi_-^{1,1}$	−128.547069	−128.539268	−128.515977	−128.477499	−128.424278	−128.356838	−128.275739
$\sigma^{4,2}\pi_+^{1,1}\pi_-^{1,1}$	−127.997348	−128.076329	−128.121230	−128.143178	−128.147626	−128.136989	−128.112654
$\sigma^{4,3}\pi_+^{1,0}\pi_-^{1,1}$	−127.993377	−128.122488	−128.217884	−128.290918	−128.347211	−128.389268	−128.418529
$\sigma^{3,3}\pi_+^{1,0}\pi_-^{2,1}$	−127.927302	−128.087973	−128.203591	−128.293716	−128.364382	−128.418695	−128.458341
$\sigma^{3,3}\pi_+^{1,0}\pi_-^{1,1}\delta_-^{1,0}$	−127.876695	−128.053459	−128.180481	−128.286829	−128.378524	−128.457626	−128.525150
$\sigma^{3,3}\pi_+^{1,0}\pi_-^{1,1}\phi_-^{1,0}$	−127.852105	−128.025146	−128.142993	−128.239875	−128.320862	−128.388805	−128.443825

Table S302: Total energies in E_h for the Ne atom in the HGBSP2-9 basis set in fully uncontracted form, employing the real-orbital approximation.

	$0.00B_0$	$0.10B_0$	$0.20B_0$	$0.30B_0$	$0.40B_0$	$0.50B_0$	$0.60B_0$
$\sigma^{3,3}\pi_+^{1,1}\pi_-^{1,1}$	−128.547098	−128.539297	−128.516006	−128.477528	−128.424307	−128.356867	−128.275769
$\sigma^{4,2}\pi_+^{1,1}\pi_-^{1,1}$	−127.998637	−128.076825	−128.121278	−128.143284	−128.147770	−128.137123	−128.112740
$\sigma^{4,3}\pi_+^{1,0}\pi_-^{1,1}$	−127.994669	−128.122981	−128.217931	−128.291025	−128.347353	−128.389403	−128.418621
$\sigma^{3,3}\pi_+^{1,0}\pi_-^{2,1}$	−127.927332	−128.088006	−128.203629	−128.293788	−128.364526	−128.418790	−128.458377
$\sigma^{3,3}\pi_+^{1,0}\pi_-^{1,1}\delta_-^{1,0}$	−127.876725	−128.053489	−128.180516	−128.286986	−128.378612	−128.457685	−128.525427
$\sigma^{3,3}\pi_+^{1,0}\pi_-^{1,1}\phi_-^{1,0}$	−127.852135	−128.025216	−128.143147	−128.240039	−128.321205	−128.388806	−128.444216

Table S303: Total energies in E_h for the Ne atom in the HGBSP3-5 basis set in fully uncontracted form, employing the real-orbital approximation.

	$0.00B_0$	$0.10B_0$	$0.20B_0$	$0.30B_0$	$0.40B_0$	$0.50B_0$	$0.60B_0$
$\sigma^{3,3}\pi_+^{1,1}\pi_-^{1,1}$	−128.544666	−128.536865	−128.513572	−128.475091	−128.421864	−128.354412	−128.273295
$\sigma^{4,2}\pi_+^{1,1}\pi_-^{1,1}$	−127.991519	−128.071958	−128.118683	−128.141814	−128.148534	−128.142125	−128.123868
$\sigma^{4,3}\pi_+^{1,0}\pi_-^{1,1}$	−127.987557	−128.118138	−128.215333	−128.289427	−128.347844	−128.394036	−128.429355
$\sigma^{3,3}\pi_+^{1,0}\pi_-^{2,1}$	−127.924820	−128.085453	−128.200766	−128.290547	−128.361725	−128.415829	−128.454438
$\sigma^{3,3}\pi_+^{1,0}\pi_-^{1,1}\delta_-^{1,0}$	−127.874242	−128.051951	−128.181558	−128.290085	−128.385145	−128.467102	−128.536577
$\sigma^{3,3}\pi_+^{1,0}\pi_-^{1,1}\phi_-^{1,0}$	−127.849652	−128.022727	−128.139963	−128.236386	−128.315074	−128.384994	−128.441636

Table S304: Total energies in E_h for the Ne atom in the HGBSP3-7 basis set in fully uncontracted form, employing the real-orbital approximation.

	$0.00B_0$	$0.10B_0$	$0.20B_0$	$0.30B_0$	$0.40B_0$	$0.50B_0$	$0.60B_0$
$\sigma^{3,3}\pi_+^{1,1}\pi_-^{1,1}$	−128.547069	−128.539268	−128.515977	−128.477499	−128.424278	−128.356838	−128.275739
$\sigma^{4,2}\pi_+^{1,1}\pi_-^{1,1}$	−127.997348	−128.076333	−128.121441	−128.144501	−128.151565	−128.145291	−128.127030
$\sigma^{4,3}\pi_+^{1,0}\pi_-^{1,1}$	−127.993377	−128.122491	−128.218061	−128.292106	−128.350873	−128.397185	−128.432481
$\sigma^{3,3}\pi_+^{1,0}\pi_-^{2,1}$	−127.927302	−128.087973	−128.203591	−128.293716	−128.364382	−128.418695	−128.458342
$\sigma^{3,3}\pi_+^{1,0}\pi_-^{1,1}\delta_-^{1,0}$	−127.876695	−128.054577	−128.184068	−128.293285	−128.387817	−128.469760	−128.540142
$\sigma^{3,3}\pi_+^{1,0}\pi_-^{1,1}\phi_-^{1,0}$	−127.852105	−128.025146	−128.142993	−128.239875	−128.320862	−128.388805	−128.443825

Table S305: Total energies in E_h for the Ne atom in the HGBSP3-9 basis set in fully uncontracted form, employing the real-orbital approximation.

	$0.00B_0$	$0.10B_0$	$0.20B_0$	$0.30B_0$	$0.40B_0$	$0.50B_0$	$0.60B_0$
$\sigma^{3,3}\pi_+^{1,1}\pi_-^{1,1}$	−128.547098	−128.539297	−128.516006	−128.477528	−128.424307	−128.356867	−128.275769
$\sigma^{4,2}\pi_+^{1,1}\pi_-^{1,1}$	−127.998637	−128.076830	−128.121497	−128.144565	−128.151607	−128.145333	−128.127095
$\sigma^{4,3}\pi_+^{1,0}\pi_-^{1,1}$	−127.994669	−128.122984	−128.218116	−128.292172	−128.350914	−128.397226	−128.432546
$\sigma^{3,3}\pi_+^{1,0}\pi_-^{2,1}$	−127.927332	−128.088006	−128.203629	−128.293788	−128.364526	−128.418791	−128.458378
$\sigma^{3,3}\pi_+^{1,0}\pi_-^{1,1}\delta_-^{1,0}$	−127.876725	−128.054607	−128.184108	−128.293406	−128.387910	−128.469824	−128.540325
$\sigma^{3,3}\pi_+^{1,0}\pi_-^{1,1}\phi_-^{1,0}$	−127.852135	−128.025216	−128.143147	−128.240039	−128.321205	−128.388806	−128.444216

Table S306: Total energies in E_h for the Ne atom in the AHGBSP1-5 basis set in fully uncontracted form, employing the real-orbital approximation.

	$0.00B_0$	$0.10B_0$	$0.20B_0$	$0.30B_0$	$0.40B_0$	$0.50B_0$	$0.60B_0$
$\sigma^{3,3}\pi_+^{1,1}\pi_-^{1,1}$	−128.544667	−128.536865	−128.513557	−128.475013	−128.421633	−128.353891	−128.272303
$\sigma^{4,2}\pi_+^{1,1}\pi_-^{1,1}$	−127.995299	−128.072883	−128.117256	−128.138968	−128.142862	−128.131662	−128.106918
$\sigma^{4,3}\pi_+^{1,0}\pi_-^{1,1}$	−127.992413	−128.120123	−128.215024	−128.287874	−128.343696	−128.385324	−128.414356
$\sigma^{3,3}\pi_+^{1,0}\pi_-^{2,1}$	−127.924448	−128.084431	−128.196776	−128.281670	−128.346498	−128.392119	−128.419904
$\sigma^{3,3}\pi_+^{1,0}\pi_-^{1,1}\delta_-^{1,0}$	−127.873837	−128.050422	−128.177564	−128.283174	−128.375514	−128.454370	−128.520525
$\sigma^{3,3}\pi_+^{1,0}\pi_-^{1,1}\phi_-^{1,0}$							

Table S307: Total energies in E_h for the Ne atom in the AHGBSP1-7 basis set in fully uncontracted form, employing the real-orbital approximation.

	$0.00B_0$	$0.10B_0$	$0.20B_0$	$0.30B_0$	$0.40B_0$	$0.50B_0$	$0.60B_0$
$\sigma^{3,3}\pi_+^{1,1}\pi_-^{1,1}$	−128.547069	−128.539267	−128.515960	−128.477420	−128.424045	−128.356314	−128.274743
$\sigma^{4,2}\pi_+^{1,1}\pi_-^{1,1}$	−127.997755	−128.075405	−128.119712	−128.141646	−128.146029	−128.135202	−128.110562
$\sigma^{4,3}\pi_+^{1,0}\pi_-^{1,1}$	−127.994869	−128.122645	−128.217479	−128.290560	−128.346881	−128.388885	−128.418015
$\sigma^{3,3}\pi_+^{1,0}\pi_-^{2,1}$	−127.926891	−128.086898	−128.199626	−128.284811	−128.348831	−128.395063	−128.425007
$\sigma^{3,3}\pi_+^{1,0}\pi_-^{1,1}\delta_-^{1,0}$	−127.876277	−128.053059	−128.180101	−128.286452	−128.378098	−128.457081	−128.524385
$\sigma^{3,3}\pi_+^{1,0}\pi_-^{1,1}\phi_-^{1,0}$							

Table S308: Total energies in E_h for the Ne atom in the AHGBSP1-9 basis set in fully uncontracted form, employing the real-orbital approximation.

	$0.00B_0$	$0.10B_0$	$0.20B_0$	$0.30B_0$	$0.40B_0$	$0.50B_0$	$0.60B_0$
$\sigma^{3,3}\pi_+^{1,1}\pi_-^{1,1}$	−128.547098	−128.539296	−128.515989	−128.477449	−128.424074	−128.356343	−128.274773
$\sigma^{4,2}\pi_+^{1,1}\pi_-^{1,1}$	−127.997786	−128.075437	−128.119752	−128.141707	−128.146089	−128.135266	−128.110620
$\sigma^{4,3}\pi_+^{1,0}\pi_-^{1,1}$	−127.994899	−128.122677	−128.217520	−128.290622	−128.346940	−128.388950	−128.418075
$\sigma^{3,3}\pi_+^{1,0}\pi_-^{2,1}$	−127.926921	−128.086932	−128.199661	−128.284931	−128.349036	−128.395119	−128.425088
$\sigma^{3,3}\pi_+^{1,0}\pi_-^{1,1}\delta_-^{1,0}$	−127.876307	−128.053089	−128.180136	−128.286606	−128.378186	−128.457139	−128.524660
$\sigma^{3,3}\pi_+^{1,0}\pi_-^{1,1}\phi_-^{1,0}$							

Table S309: Total energies in E_h for the Ne atom in the AHGBSP2-5 basis set in fully uncontracted form, employing the real-orbital approximation.

	$0.00B_0$	$0.10B_0$	$0.20B_0$	$0.30B_0$	$0.40B_0$	$0.50B_0$	$0.60B_0$
$\sigma^{3,3}\pi_+^{1,1}\pi_-^{1,1}$	−128.544668	−128.536867	−128.513574	−128.475093	−128.421866	−128.354415	−128.273298
$\sigma^{4,2}\pi_+^{1,1}\pi_-^{1,1}$	−127.996776	−128.074364	−128.118758	−128.140524	−128.144521	−128.133495	−128.109015
$\sigma^{4,3}\pi_+^{1,0}\pi_-^{1,1}$	−127.992828	−128.120535	−128.215429	−128.288274	−128.344104	−128.385772	−128.414897
$\sigma^{3,3}\pi_+^{1,0}\pi_-^{2,1}$	−127.924852	−128.085501	−128.200826	−128.290680	−128.361779	−128.415836	−128.454482
$\sigma^{3,3}\pi_+^{1,0}\pi_-^{1,1}\delta_-^{1,0}$	−127.874248	−128.050816	−128.177938	−128.283548	−128.375935	−128.454910	−128.521287
$\sigma^{3,3}\pi_+^{1,0}\pi_-^{1,1}\phi_-^{1,0}$	−127.849658	−128.022733	−128.139976	−128.236398	−128.315094	−128.385005	−128.441644

Table S310: Total energies in E_h for the Ne atom in the AHGBSP2-7 basis set in fully uncontracted form, employing the real-orbital approximation.

	$0.00B_0$	$0.10B_0$	$0.20B_0$	$0.30B_0$	$0.40B_0$	$0.50B_0$	$0.60B_0$
$\sigma^{3,3}\pi_+^{1,1}\pi_-^{1,1}$	−128.547069	−128.539268	−128.515977	−128.477499	−128.424278	−128.356838	−128.275739
$\sigma^{4,2}\pi_+^{1,1}\pi_-^{1,1}$	−127.999256	−128.076910	−128.121238	−128.143226	−128.147713	−128.137060	−128.112684
$\sigma^{4,3}\pi_+^{1,0}\pi_-^{1,1}$	−127.995290	−128.123064	−128.217891	−128.290967	−128.347296	−128.389339	−128.418561
$\sigma^{3,3}\pi_+^{1,0}\pi_-^{2,1}$	−127.927302	−128.087973	−128.203593	−128.293717	−128.364388	−128.418698	−128.458342
$\sigma^{3,3}\pi_+^{1,0}\pi_-^{1,1}\delta_-^{1,0}$	−127.876695	−128.053459	−128.180482	−128.286833	−128.378525	−128.457627	−128.525153
$\sigma^{3,3}\pi_+^{1,0}\pi_-^{1,1}\phi_-^{1,0}$	−127.852105	−128.025150	−128.142997	−128.239877	−128.320865	−128.388805	−128.443828

Table S311: Total energies in E_h for the Ne atom in the AHGBSP2-9 basis set in fully uncontracted form, employing the real-orbital approximation.

	$0.00B_0$	$0.10B_0$	$0.20B_0$	$0.30B_0$	$0.40B_0$	$0.50B_0$	$0.60B_0$
$\sigma^{3,3}\pi_+^{1,1}\pi_-^{1,1}$	−128.547098	−128.539297	−128.516006	−128.477528	−128.424307	−128.356867	−128.275769
$\sigma^{4,2}\pi_+^{1,1}\pi_-^{1,1}$	−127.999286	−128.076942	−128.121278	−128.143287	−128.147773	−128.137124	−128.112742
$\sigma^{4,3}\pi_+^{1,0}\pi_-^{1,1}$	−127.995321	−128.123096	−128.217931	−128.291029	−128.347355	−128.389404	−128.418622
$\sigma^{3,3}\pi_+^{1,0}\pi_-^{2,1}$	−127.927332	−128.088006	−128.203629	−128.293788	−128.364527	−128.418791	−128.458378
$\sigma^{3,3}\pi_+^{1,0}\pi_-^{1,1}\delta_-^{1,0}$	−127.876725	−128.053489	−128.180516	−128.286986	−128.378613	−128.457685	−128.525428
$\sigma^{3,3}\pi_+^{1,0}\pi_-^{1,1}\phi_-^{1,0}$	−127.852135	−128.025216	−128.143148	−128.240039	−128.321205	−128.388807	−128.444216

Table S312: Total energies in E_h for the Ne atom in the AHGBSP3-5 basis set in fully uncontracted form, employing the real-orbital approximation.

	$0.00B_0$	$0.10B_0$	$0.20B_0$	$0.30B_0$	$0.40B_0$	$0.50B_0$	$0.60B_0$
$\sigma^{3,3}\pi_+^{1,1}\pi_-^{1,1}$	−128.544667	−128.536867	−128.513574	−128.475093	−128.421866	−128.354415	−128.273298
$\sigma^{4,2}\pi_+^{1,1}\pi_-^{1,1}$	−127.996776	−128.074372	−128.118981	−128.141819	−128.148582	−128.142220	−128.124021
$\sigma^{4,3}\pi_+^{1,0}\pi_-^{1,1}$	−127.992828	−128.120541	−128.215617	−128.289432	−128.347891	−128.394120	−128.429489
$\sigma^{3,3}\pi_+^{1,0}\pi_-^{2,1}$	−127.924852	−128.085501	−128.200826	−128.290680	−128.361779	−128.415836	−128.454482
$\sigma^{3,3}\pi_+^{1,0}\pi_-^{1,1}\delta_-^{1,0}$	−127.874248	−128.051965	−128.181564	−128.290102	−128.385155	−128.467108	−128.536588
$\sigma^{3,3}\pi_+^{1,0}\pi_-^{1,1}\phi_-^{1,0}$	−127.849658	−128.022732	−128.139976	−128.236398	−128.315094	−128.385005	−128.441644

Table S313: Total energies in E_h for the Ne atom in the AHGBSP3-7 basis set in fully uncontracted form, employing the real-orbital approximation.

	$0.00B_0$	$0.10B_0$	$0.20B_0$	$0.30B_0$	$0.40B_0$	$0.50B_0$	$0.60B_0$
$\sigma^{3,3}\pi_+^{1,1}\pi_-^{1,1}$	−128.547069	−128.539268	−128.515977	−128.477499	−128.424278	−128.356838	−128.275739
$\sigma^{4,2}\pi_+^{1,1}\pi_-^{1,1}$	−127.999256	−128.076916	−128.121459	−128.144508	−128.151568	−128.145296	−128.127043
$\sigma^{4,3}\pi_+^{1,0}\pi_-^{1,1}$	−127.995290	−128.123068	−128.218078	−128.292114	−128.350876	−128.397188	−128.432493
$\sigma^{3,3}\pi_+^{1,0}\pi_-^{2,1}$	−127.927302	−128.087973	−128.203593	−128.293717	−128.364388	−128.418699	−128.458342
$\sigma^{3,3}\pi_+^{1,0}\pi_-^{1,1}\delta_-^{1,0}$	−127.876695	−128.054577	−128.184069	−128.293287	−128.387819	−128.469760	−128.540143
$\sigma^{3,3}\pi_+^{1,0}\pi_-^{1,1}\phi_-^{1,0}$	−127.852105	−128.025150	−128.142997	−128.239877	−128.320865	−128.388805	−128.443828

Table S314: Total energies in E_h for the Ne atom in the AHGBSP3-9 basis set in fully uncontracted form, employing the real-orbital approximation.

	$0.00B_0$	$0.10B_0$	$0.20B_0$	$0.30B_0$	$0.40B_0$	$0.50B_0$	$0.60B_0$
$\sigma^{3,3}\pi_+^{1,1}\pi_-^{1,1}$	−128.547098	−128.539297	−128.516006	−128.477528	−128.424307	−128.356867	−128.275769
$\sigma^{4,2}\pi_+^{1,1}\pi_-^{1,1}$	−127.999286	−128.076948	−128.121500	−128.144567	−128.151608	−128.145333	−128.127100
$\sigma^{4,3}\pi_+^{1,0}\pi_-^{1,1}$	−127.995321	−128.123100	−128.218119	−128.292174	−128.350916	−128.397226	−128.432552
$\sigma^{3,3}\pi_+^{1,0}\pi_-^{2,1}$	−127.927332	−128.088006	−128.203629	−128.293788	−128.364527	−128.418791	−128.458378
$\sigma^{3,3}\pi_+^{1,0}\pi_-^{1,1}\delta_-^{1,0}$	−127.876725	−128.054607	−128.184108	−128.293407	−128.387911	−128.469824	−128.540326
$\sigma^{3,3}\pi_+^{1,0}\pi_-^{1,1}\phi_-^{1,0}$	−127.852135	−128.025216	−128.143148	−128.240039	−128.321205	−128.388807	−128.444216

Table S315: Total energies in E_h for the Ne atom in the 6-311++G(3df,3pd) basis set in fully uncontracted form, employing the real-orbital approximation.

	$0.00B_0$	$0.10B_0$	$0.20B_0$	$0.30B_0$	$0.40B_0$	$0.50B_0$	$0.60B_0$
$\sigma^{3,3}\pi_+^{1,1}\pi_-^{1,1}$	−128.527158	−128.519328	−128.495922	−128.457184	−128.403473	−128.335222	−128.252897
$\sigma^{4,2}\pi_+^{1,1}\pi_-^{1,1}$	−127.892135	−127.978577	−128.038019	−128.070818	−128.077576	−128.059170	−128.016794
$\sigma^{4,3}\pi_+^{1,0}\pi_-^{1,1}$	−127.888401	−128.025361	−128.136351	−128.221698	−128.281961	−128.317959	−128.330806
$\sigma^{3,3}\pi_+^{1,0}\pi_-^{2,1}$	−127.783217	−127.966297	−128.115734	−128.232144	−128.316629	−128.370913	−128.397523
$\sigma^{3,3}\pi_+^{1,0}\pi_-^{1,1}\delta_-^{1,0}$	−126.741486	−126.982435	−127.205324	−127.410280	−127.597499	−127.767230	−127.919762
$\sigma^{3,3}\pi_+^{1,0}\pi_-^{1,1}\phi_-^{1,0}$	−119.187025	−119.479532	−119.757132	−120.020040	−120.268573	−120.503103	−120.724030

Table S316: Total energies in E_h for the Ne atom in the def2-TZVP basis set in fully uncontracted form, employing the real-orbital approximation.

	$0.00B_0$	$0.10B_0$	$0.20B_0$	$0.30B_0$	$0.40B_0$	$0.50B_0$	$0.60B_0$
$\sigma^{3,3}\pi_+^{1,1}\pi_-^{1,1}$	−128.542563	−128.534790	−128.511538	−128.473003	−128.419500	−128.351444	−128.269330
$\sigma^{4,2}\pi_+^{1,1}\pi_-^{1,1}$	−127.530559	−127.620571	−127.690658	−127.740970	−127.771749	−127.783317	−127.776065
$\sigma^{4,3}\pi_+^{1,0}\pi_-^{1,1}$	−127.526884	−127.667449	−127.789186	−127.892223	−127.976761	−128.043074	−128.091494
$\sigma^{3,3}\pi_+^{1,0}\pi_-^{2,1}$	−127.476275	−127.664988	−127.831168	−127.974940	−128.096508	−128.196152	−128.274223
$\sigma^{3,3}\pi_+^{1,0}\pi_-^{1,1}\delta_-^{1,0}$	−125.726267	−125.968500	−126.195246	−126.406640	−126.602897	−126.784306	−126.951207
$\sigma^{3,3}\pi_+^{1,0}\pi_-^{1,1}\phi_-^{1,0}$	−119.049073	−119.341641	−119.619401	−119.882521	−120.131268	−120.365991	−120.587109

Table S317: Total energies in E_h for the Na atom in the cc-pVDZ basis set in fully uncontracted form, employing the real-orbital approximation.

	$0.00B_0$	$0.10B_0$	$0.20B_0$	$0.30B_0$	$0.40B_0$	$0.50B_0$	$0.60B_0$
$\sigma^{4,3}\pi_+^{1,1}\pi_-^{1,1}$	−161.853066	−161.881902	−161.878202	−161.851678	−161.806474	−161.745032	−161.667673
$\sigma^{3,3}\pi_+^{1,1}\pi_-^{2,1}$	−161.780395	−161.841158	−161.857698	−161.837723	−161.778511	−161.684839	−161.567905
$\sigma^{3,3}\pi_+^{2,1}\pi_-^{1,1}$	−161.780395	−161.741158	−161.657698	−161.537723	−161.378511	−161.184839	−160.967905
$\sigma^{3,3}\pi_+^{1,1}\pi_-^{1,1}\delta_-^{1,0}$	−161.599547	−161.724919	−161.801059	−161.828030	−161.805937	−161.734910	−161.615109
$\sigma^{3,3}\pi_+^{1,1}\pi_-^{1,1}\phi_-^{1,0}$							

Table S318: Total energies in E_h for the Na atom in the cc-pVTZ basis set in fully uncontracted form, employing the real-orbital approximation.

	$0.00B_0$	$0.10B_0$	$0.20B_0$	$0.30B_0$	$0.40B_0$	$0.50B_0$	$0.60B_0$
$\sigma^{4,3}\pi_+^{1,1}\pi_-^{1,1}$	−161.858038	−161.887030	−161.884546	−161.859875	−161.815656	−161.754259	−161.677986
$\sigma^{3,3}\pi_+^{1,1}\pi_-^{2,1}$	−161.785487	−161.847339	−161.863150	−161.855662	−161.830331	−161.786916	−161.726629
$\sigma^{3,3}\pi_+^{2,1}\pi_-^{1,1}$	−161.785487	−161.747339	−161.663150	−161.555662	−161.430331	−161.286916	−161.126629
$\sigma^{3,3}\pi_+^{1,1}\pi_-^{1,1}\delta_-^{1,0}$	−161.671917	−161.785124	−161.831858	−161.836109	−161.823805	−161.795410	−161.742079
$\sigma^{3,3}\pi_+^{1,1}\pi_-^{1,1}\phi_-^{1,0}$	−161.321448	−161.498206	−161.628503	−161.712410	−161.750036	−161.741523	−161.687042

Table S319: Total energies in E_h for the Na atom in the cc-pVQZ basis set in fully uncontracted form, employing the real-orbital approximation.

	$0.00B_0$	$0.10B_0$	$0.20B_0$	$0.30B_0$	$0.40B_0$	$0.50B_0$	$0.60B_0$
$\sigma^{4,3}\pi_+^{1,1}\pi_-^{1,1}$	−161.858719	−161.887871	−161.885501	−161.861706	−161.821082	−161.766599	−161.700575
$\sigma^{3,3}\pi_+^{1,1}\pi_-^{2,1}$	−161.786186	−161.848119	−161.865719	−161.859704	−161.834344	−161.792190	−161.735351
$\sigma^{3,3}\pi_+^{2,1}\pi_-^{1,1}$	−161.786186	−161.748119	−161.665719	−161.559704	−161.434344	−161.292190	−161.135351
$\sigma^{3,3}\pi_+^{1,1}\pi_-^{1,1}\delta_-^{1,0}$	−161.702623	−161.803861	−161.834026	−161.839205	−161.830706	−161.809403	−161.773783
$\sigma^{3,3}\pi_+^{1,1}\pi_-^{1,1}\phi_-^{1,0}$	−161.474531	−161.641138	−161.742411	−161.783653	−161.776865	−161.742249	−161.697204

Table S320: Total energies in E_h for the Na atom in the cc-pV5Z basis set in fully uncontracted form, employing the real-orbital approximation.

	$0.00B_0$	$0.10B_0$	$0.20B_0$	$0.30B_0$	$0.40B_0$	$0.50B_0$	$0.60B_0$
$\sigma^{4,3}\pi_+^{1,1}\pi_-^{1,1}$	−161.858719	−161.887873	−161.885503	−161.861755	−161.821269	−161.767006	−161.701271
$\sigma^{3,3}\pi_+^{1,1}\pi_-^{2,1}$	−161.786187	−161.848135	−161.865827	−161.859940	−161.834804	−161.793350	−161.738101
$\sigma^{3,3}\pi_+^{2,1}\pi_-^{1,1}$	−161.786187	−161.748135	−161.665827	−161.559940	−161.434804	−161.293350	−161.138101
$\sigma^{3,3}\pi_+^{1,1}\pi_-^{1,1}\delta_-^{1,0}$	−161.703474	−161.803982	−161.834725	−161.840815	−161.832659	−161.812859	−161.781956
$\sigma^{3,3}\pi_+^{1,1}\pi_-^{1,1}\phi_-^{1,0}$	−161.484749	−161.650176	−161.748480	−161.786828	−161.780267	−161.750492	−161.712431

Table S321: Total energies in E_h for the Na atom in the aug-cc-pVDZ basis set in fully uncontracted form, employing the real-orbital approximation.

	$0.00B_0$	$0.10B_0$	$0.20B_0$	$0.30B_0$	$0.40B_0$	$0.50B_0$	$0.60B_0$
$\sigma^{4,3}\pi_+^{1,1}\pi_-^{1,1}$	−161.853080	−161.882202	−161.879714	−161.854171	−161.808994	−161.747152	−161.670182
$\sigma^{3,3}\pi_+^{1,1}\pi_-^{2,1}$	−161.780443	−161.841488	−161.857714	−161.838136	−161.780077	−161.687898	−161.572275
$\sigma^{3,3}\pi_+^{2,1}\pi_-^{1,1}$	−161.780443	−161.741488	−161.657714	−161.538136	−161.380077	−161.187898	−160.972275
$\sigma^{3,3}\pi_+^{1,1}\pi_-^{1,1}\delta_-^{1,0}$	−161.692995	−161.796443	−161.827471	−161.830044	−161.808300	−161.750318	−161.651215
$\sigma^{3,3}\pi_+^{1,1}\pi_-^{1,1}\phi_-^{1,0}$							

Table S322: Total energies in E_h for the Na atom in the aug-cc-pVTZ basis set in fully uncontracted form, employing the real-orbital approximation.

	$0.00B_0$	$0.10B_0$	$0.20B_0$	$0.30B_0$	$0.40B_0$	$0.50B_0$	$0.60B_0$
$\sigma^{4,3}\pi_+^{1,1}\pi_-^{1,1}$	−161.858040	−161.887190	−161.884725	−161.859964	−161.816302	−161.755969	−161.680887
$\sigma^{3,3}\pi_+^{1,1}\pi_-^{2,1}$	−161.785490	−161.847601	−161.865521	−161.859868	−161.834474	−161.789798	−161.728025
$\sigma^{3,3}\pi_+^{2,1}\pi_-^{1,1}$	−161.785490	−161.747601	−161.665521	−161.559868	−161.434474	−161.289798	−161.128025
$\sigma^{3,3}\pi_+^{1,1}\pi_-^{1,1}\delta_-^{1,0}$	−161.726447	−161.805562	−161.832511	−161.836489	−161.824087	−161.795453	−161.742987
$\sigma^{3,3}\pi_+^{1,1}\pi_-^{1,1}\phi_-^{1,0}$	−161.564217	−161.719350	−161.789512	−161.794581	−161.773186	−161.742857	−161.690166

Table S323: Total energies in E_h for the Na atom in the aug-cc-pVQZ basis set in fully uncontracted form, employing the real-orbital approximation.

	$0.00B_0$	$0.10B_0$	$0.20B_0$	$0.30B_0$	$0.40B_0$	$0.50B_0$	$0.60B_0$
$\sigma^{4,3}\pi_+^{1,1}\pi_-^{1,1}$	−161.858719	−161.887901	−161.885578	−161.862272	−161.822938	−161.770363	−161.706185
$\sigma^{3,3}\pi_+^{1,1}\pi_-^{2,1}$	−161.786187	−161.848529	−161.867249	−161.860837	−161.834752	−161.792346	−161.735585
$\sigma^{3,3}\pi_+^{2,1}\pi_-^{1,1}$	−161.786187	−161.748529	−161.667249	−161.560837	−161.434752	−161.292346	−161.135585
$\sigma^{3,3}\pi_+^{1,1}\pi_-^{1,1}\delta_-^{1,0}$	−161.728262	−161.808372	−161.836220	−161.844256	−161.836338	−161.813359	−161.775680
$\sigma^{3,3}\pi_+^{1,1}\pi_-^{1,1}\phi_-^{1,0}$	−161.635234	−161.769547	−161.797226	−161.792476	−161.776909	−161.743270	−161.698162

Table S324: Total energies in E_h for the Na atom in the aug-cc-pV5Z basis set in fully uncontracted form, employing the real-orbital approximation.

$\sigma^{4,3}\pi_+^{1,1}\pi_-^{1,1}$
$\sigma^{3,3}\pi_+^{1,1}\pi_-^{2,1}$
$\sigma^{3,3}\pi_+^{2,1}\pi_-^{1,1}$
$\sigma^{3,3}\pi_+^{1,1}\pi_-^{1,1}\delta_-^{1,0}$
$\sigma^{3,3}\pi_+^{1,1}\pi_-^{1,1}\phi_-^{1,0}$

Table S325: Total energies in E_h for the Na atom in the HGBSP1-5 basis set in fully uncontracted form, employing the real-orbital approximation.

	$0.00B_0$	$0.10B_0$	$0.20B_0$	$0.30B_0$	$0.40B_0$	$0.50B_0$	$0.60B_0$
$\sigma^{4,3}\pi_+^{1,1}\pi_-^{1,1}$	−161.846732	−161.879446	−161.881084	−161.858607	−161.817724	−161.761839	−161.692951
$\sigma^{3,3}\pi_+^{1,1}\pi_-^{2,1}$	−161.783267	−161.844997	−161.861023	−161.849340	−161.818076	−161.769694	−161.704131
$\sigma^{3,3}\pi_+^{2,1}\pi_-^{1,1}$	−161.783267	−161.744997	−161.661023	−161.549340	−161.418076	−161.269694	−161.104131
$\sigma^{3,3}\pi_+^{1,1}\pi_-^{1,1}\delta_-^{1,0}$	−161.729475	−161.805676	−161.831824	−161.836279	−161.825809	−161.803909	−161.769353
$\sigma^{3,3}\pi_+^{1,1}\pi_-^{1,1}\phi_-^{1,0}$							

Table S326: Total energies in E_h for the Na atom in the HGBSP1-7 basis set in fully uncontracted form, employing the real-orbital approximation.

	$0.00B_0$	$0.10B_0$	$0.20B_0$	$0.30B_0$	$0.40B_0$	$0.50B_0$	$0.60B_0$
$\sigma^{4,3}\pi_+^{1,1}\pi_-^{1,1}$	−161.855337	−161.886451	−161.885504	−161.861859	−161.821189	−161.766118	−161.698051
$\sigma^{3,3}\pi_+^{1,1}\pi_-^{2,1}$	−161.786384	−161.848211	−161.864221	−161.853518	−161.821892	−161.772634	−161.707669
$\sigma^{3,3}\pi_+^{2,1}\pi_-^{1,1}$	−161.786384	−161.748211	−161.664221	−161.553518	−161.421892	−161.272634	−161.107669
$\sigma^{3,3}\pi_+^{1,1}\pi_-^{1,1}\delta_-^{1,0}$	−161.732591	−161.808865	−161.834933	−161.840170	−161.829949	−161.807185	−161.772694
$\sigma^{3,3}\pi_+^{1,1}\pi_-^{1,1}\phi_-^{1,0}$							

Table S327: Total energies in E_h for the Na atom in the HGBSP1-9 basis set in fully uncontracted form, employing the real-orbital approximation.

	$0.00B_0$	$0.10B_0$	$0.20B_0$	$0.30B_0$	$0.40B_0$	$0.50B_0$	$0.60B_0$
$\sigma^{4,3}\pi_+^{1,1}\pi_-^{1,1}$	−161.858939	−161.888136	−161.885743	−161.861928	−161.821337	−161.766322	−161.698264
$\sigma^{3,3}\pi_+^{1,1}\pi_-^{2,1}$	−161.786422	−161.848249	−161.864302	−161.853558	−161.822121	−161.772903	−161.707760
$\sigma^{3,3}\pi_+^{2,1}\pi_-^{1,1}$	−161.786422	−161.748249	−161.664302	−161.553558	−161.422121	−161.272903	−161.107760
$\sigma^{3,3}\pi_+^{1,1}\pi_-^{1,1}\delta_-^{1,0}$	−161.732629	−161.808915	−161.835041	−161.840240	−161.830189	−161.807292	−161.772750
$\sigma^{3,3}\pi_+^{1,1}\pi_-^{1,1}\phi_-^{1,0}$							

Table S328: Total energies in E_h for the Na atom in the HGBSP2-5 basis set in fully uncontracted form, employing the real-orbital approximation.

	$0.00B_0$	$0.10B_0$	$0.20B_0$	$0.30B_0$	$0.40B_0$	$0.50B_0$	$0.60B_0$
$\sigma^{4,3}\pi_+^{1,1}\pi_-^{1,1}$	−161.846732	−161.879447	−161.881086	−161.858618	−161.817763	−161.761934	−161.693150
$\sigma^{3,3}\pi_+^{1,1}\pi_-^{2,1}$	−161.783269	−161.845634	−161.864333	−161.857387	−161.831975	−161.790580	−161.733971
$\sigma^{3,3}\pi_+^{2,1}\pi_-^{1,1}$	−161.783269	−161.745634	−161.664333	−161.557387	−161.431975	−161.290580	−161.133971
$\sigma^{3,3}\pi_+^{1,1}\pi_-^{1,1}\delta_-^{1,0}$	−161.705057	−161.805679	−161.831842	−161.836339	−161.825957	−161.804208	−161.769889
$\sigma^{3,3}\pi_+^{1,1}\pi_-^{1,1}\phi_-^{1,0}$	−161.705057	−161.777782	−161.794363	−161.792963	−161.771424	−161.737327	−161.695256

Table S329: Total energies in E_h for the Na atom in the HGBSP2-7 basis set in fully uncontracted form, employing the real-orbital approximation.

	$0.00B_0$	$0.10B_0$	$0.20B_0$	$0.30B_0$	$0.40B_0$	$0.50B_0$	$0.60B_0$
$\sigma^{4,3}\pi_+^{1,1}\pi_-^{1,1}$	−161.855337	−161.886451	−161.885506	−161.861871	−161.821227	−161.766214	−161.698251
$\sigma^{3,3}\pi_+^{1,1}\pi_-^{2,1}$	−161.786386	−161.848835	−161.867524	−161.861277	−161.835776	−161.793921	−161.737518
$\sigma^{3,3}\pi_+^{2,1}\pi_-^{1,1}$	−161.786386	−161.748835	−161.667524	−161.561277	−161.435776	−161.293921	−161.137518
$\sigma^{3,3}\pi_+^{1,1}\pi_-^{1,1}\delta_-^{1,0}$	−161.732591	−161.808868	−161.834952	−161.840230	−161.830095	−161.807483	−161.773230
$\sigma^{3,3}\pi_+^{1,1}\pi_-^{1,1}\phi_-^{1,0}$	−161.708174	−161.781120	−161.798970	−161.796089	−161.776974	−161.744685	−161.700452

Table S330: Total energies in E_h for the Na atom in the HGBSP2-9 basis set in fully uncontracted form, employing the real-orbital approximation.

	$0.00B_0$	$0.10B_0$	$0.20B_0$	$0.30B_0$	$0.40B_0$	$0.50B_0$	$0.60B_0$
$\sigma^{4,3}\pi_+^{1,1}\pi_-^{1,1}$	−161.858939	−161.888136	−161.885745	−161.861940	−161.821375	−161.766417	−161.698463
$\sigma^{3,3}\pi_+^{1,1}\pi_-^{2,1}$	−161.786424	−161.848874	−161.867592	−161.861318	−161.835897	−161.794106	−161.737655
$\sigma^{3,3}\pi_+^{2,1}\pi_-^{1,1}$	−161.786424	−161.748874	−161.667592	−161.561318	−161.435897	−161.294106	−161.137655
$\sigma^{3,3}\pi_+^{1,1}\pi_-^{1,1}\delta_-^{1,0}$	−161.732629	−161.808918	−161.835059	−161.840300	−161.830335	−161.807590	−161.773286
$\sigma^{3,3}\pi_+^{1,1}\pi_-^{1,1}\phi_-^{1,0}$	−161.708212	−161.781217	−161.799149	−161.796133	−161.777356	−161.745086	−161.700461

Table S331: Total energies in E_h for the Na atom in the HGBSP3-5 basis set in fully uncontracted form, employing the real-orbital approximation.

	$0.00B_0$	$0.10B_0$	$0.20B_0$	$0.30B_0$	$0.40B_0$	$0.50B_0$	$0.60B_0$
$\sigma^{4,3}\pi_+^{1,1}\pi_-^{1,1}$	−161.846732	−161.879447	−161.881165	−161.859366	−161.820639	−161.768836	−161.705756
$\sigma^{3,3}\pi_+^{1,1}\pi_-^{2,1}$	−161.783269	−161.845633	−161.864333	−161.857387	−161.831975	−161.790580	−161.733971
$\sigma^{3,3}\pi_+^{2,1}\pi_-^{1,1}$	−161.783269	−161.745633	−161.664333	−161.557387	−161.431975	−161.290580	−161.133971
$\sigma^{3,3}\pi_+^{1,1}\pi_-^{1,1}\delta_-^{1,0}$	−161.729475	−161.806874	−161.835622	−161.843431	−161.836182	−161.817394	−161.786394
$\sigma^{3,3}\pi_+^{1,1}\pi_-^{1,1}\phi_-^{1,0}$	−161.693809	−161.777782	−161.794362	−161.792962	−161.771424	−161.737327	−161.695256

Table S332: Total energies in E_h for the Na atom in the HGBSP3-7 basis set in fully uncontracted form, employing the real-orbital approximation.

	$0.00B_0$	$0.10B_0$	$0.20B_0$	$0.30B_0$	$0.40B_0$	$0.50B_0$	$0.60B_0$
$\sigma^{4,3}\pi_+^{1,1}\pi_-^{1,1}$	−161.855337	−161.886452	−161.885620	−161.862738	−161.824066	−161.772531	−161.709596
$\sigma^{3,3}\pi_+^{1,1}\pi_-^{2,1}$	−161.786386	−161.848835	−161.867524	−161.861277	−161.835776	−161.793921	−161.737518
$\sigma^{3,3}\pi_+^{2,1}\pi_-^{1,1}$	−161.786386	−161.748835	−161.667524	−161.561277	−161.435776	−161.293921	−161.137518
$\sigma^{3,3}\pi_+^{1,1}\pi_-^{1,1}\delta_-^{1,0}$	−161.732591	−161.810042	−161.838772	−161.847118	−161.840255	−161.820781	−161.789695
$\sigma^{3,3}\pi_+^{1,1}\pi_-^{1,1}\phi_-^{1,0}$	−161.708174	−161.781120	−161.798970	−161.796089	−161.776974	−161.744685	−161.700452

Table S333: Total energies in E_h for the Na atom in the HGBSP3-9 basis set in fully uncontracted form, employing the real-orbital approximation.

	$0.00B_0$	$0.10B_0$	$0.20B_0$	$0.30B_0$	$0.40B_0$	$0.50B_0$	$0.60B_0$
$\sigma^{4,3}\pi_+^{1,1}\pi_-^{1,1}$	−161.858939	−161.888140	−161.885890	−161.862823	−161.824142	−161.772580	−161.709644
$\sigma^{3,3}\pi_+^{1,1}\pi_-^{2,1}$	−161.786424	−161.848874	−161.867592	−161.861318	−161.835897	−161.794106	−161.737655
$\sigma^{3,3}\pi_+^{2,1}\pi_-^{1,1}$	−161.786424	−161.748874	−161.667592	−161.561318	−161.435897	−161.294106	−161.137655
$\sigma^{3,3}\pi_+^{1,1}\pi_-^{1,1}\delta_-^{1,0}$	−161.732629	−161.810091	−161.838867	−161.847183	−161.840438	−161.820901	−161.789772
$\sigma^{3,3}\pi_+^{1,1}\pi_-^{1,1}\phi_-^{1,0}$	−161.708212	−161.781217	−161.799149	−161.796133	−161.777356	−161.745086	−161.700461

Table S334: Total energies in E_h for the Na atom in the AHGBSP1-5 basis set in fully uncontracted form, employing the real-orbital approximation.

	$0.00B_0$	$0.10B_0$	$0.20B_0$	$0.30B_0$	$0.40B_0$	$0.50B_0$	$0.60B_0$
$\sigma^{4,3}\pi_+^{1,1}\pi_-^{1,1}$	−161.855796	−161.884934	−161.882557	−161.858720	−161.817823	−161.762323	−161.693854
$\sigma^{3,3}\pi_+^{1,1}\pi_-^{2,1}$	−161.783274	−161.845023	−161.861036	−161.849400	−161.818119	−161.769705	−161.704148
$\sigma^{3,3}\pi_+^{2,1}\pi_-^{1,1}$	−161.783274	−161.745023	−161.661036	−161.549400	−161.418119	−161.269705	−161.104148
$\sigma^{3,3}\pi_+^{1,1}\pi_-^{1,1}\delta_-^{1,0}$	−161.729483	−161.805689	−161.831836	−161.836305	−161.825840	−161.803921	−161.769363
$\sigma^{3,3}\pi_+^{1,1}\pi_-^{1,1}\phi_-^{1,0}$							

Table S335: Total energies in E_h for the Na atom in the AHGBSP1-7 basis set in fully uncontracted form, employing the real-orbital approximation.

	$0.00B_0$	$0.10B_0$	$0.20B_0$	$0.30B_0$	$0.40B_0$	$0.50B_0$	$0.60B_0$
$\sigma^{4,3}\pi_+^{1,1}\pi_-^{1,1}$	−161.858915	−161.888094	−161.885703	−161.861864	−161.821260	−161.766241	−161.698173
$\sigma^{3,3}\pi_+^{1,1}\pi_-^{2,1}$	−161.786384	−161.848211	−161.864229	−161.853519	−161.821907	−161.772651	−161.707673
$\sigma^{3,3}\pi_+^{2,1}\pi_-^{1,1}$	−161.786384	−161.748211	−161.664229	−161.553519	−161.421907	−161.272651	−161.107673
$\sigma^{3,3}\pi_+^{1,1}\pi_-^{1,1}\delta_-^{1,0}$	−161.732591	−161.808868	−161.834939	−161.840171	−161.829955	−161.807187	−161.772695
$\sigma^{3,3}\pi_+^{1,1}\pi_-^{1,1}\phi_-^{1,0}$							

Table S336: Total energies in E_h for the Na atom in the AHGBSP1-9 basis set in fully uncontracted form, employing the real-orbital approximation.

	$0.00B_0$	$0.10B_0$	$0.20B_0$	$0.30B_0$	$0.40B_0$	$0.50B_0$	$0.60B_0$
$\sigma^{4,3}\pi_+^{1,1}\pi_-^{1,1}$	−161.858953	−161.888136	−161.885743	−161.861929	−161.821337	−161.766322	−161.698264
$\sigma^{3,3}\pi_+^{1,1}\pi_-^{2,1}$	−161.786422	−161.848249	−161.864303	−161.853559	−161.822121	−161.772906	−161.707762
$\sigma^{3,3}\pi_+^{2,1}\pi_-^{1,1}$	−161.786422	−161.748249	−161.664303	−161.553559	−161.422121	−161.272906	−161.107762
$\sigma^{3,3}\pi_+^{1,1}\pi_-^{1,1}\delta_-^{1,0}$	−161.732629	−161.808915	−161.835041	−161.840240	−161.830189	−161.807292	−161.772750
$\sigma^{3,3}\pi_+^{1,1}\pi_-^{1,1}\phi_-^{1,0}$							

Table S337: Total energies in E_h for the Na atom in the AHGBSP2-5 basis set in fully uncontracted form, employing the real-orbital approximation.

	$0.00B_0$	$0.10B_0$	$0.20B_0$	$0.30B_0$	$0.40B_0$	$0.50B_0$	$0.60B_0$
$\sigma^{4,3}\pi_+^{1,1}\pi_-^{1,1}$	−161.855796	−161.884934	−161.882560	−161.858731	−161.817861	−161.762418	−161.694054
$\sigma^{3,3}\pi_+^{1,1}\pi_-^{2,1}$	−161.783276	−161.845656	−161.864342	−161.857424	−161.832015	−161.790601	−161.733982
$\sigma^{3,3}\pi_+^{2,1}\pi_-^{1,1}$	−161.783276	−161.745656	−161.664342	−161.557424	−161.432015	−161.290601	−161.133982
$\sigma^{3,3}\pi_+^{1,1}\pi_-^{1,1}\delta_-^{1,0}$	−161.729484	−161.805692	−161.831854	−161.836365	−161.825988	−161.804220	−161.769898
$\sigma^{3,3}\pi_+^{1,1}\pi_-^{1,1}\phi_-^{1,0}$	−161.705065	−161.777801	−161.794392	−161.792971	−161.771445	−161.737352	−161.695272

Table S338: Total energies in E_h for the Na atom in the AHGBSP2-7 basis set in fully uncontracted form, employing the real-orbital approximation.

	$0.00B_0$	$0.10B_0$	$0.20B_0$	$0.30B_0$	$0.40B_0$	$0.50B_0$	$0.60B_0$
$\sigma^{4,3}\pi_+^{1,1}\pi_-^{1,1}$	−161.858915	−161.888094	−161.885705	−161.861876	−161.821298	−161.766336	−161.698371
$\sigma^{3,3}\pi_+^{1,1}\pi_-^{2,1}$	−161.786386	−161.848835	−161.867529	−161.861277	−161.835782	−161.793930	−161.737524
$\sigma^{3,3}\pi_+^{2,1}\pi_-^{1,1}$	−161.786386	−161.748835	−161.667529	−161.561277	−161.435782	−161.293930	−161.137524
$\sigma^{3,3}\pi_+^{1,1}\pi_-^{1,1}\delta_-^{1,0}$	−161.732591	−161.808870	−161.834957	−161.840231	−161.830102	−161.807485	−161.773231
$\sigma^{3,3}\pi_+^{1,1}\pi_-^{1,1}\phi_-^{1,0}$	−161.708174	−161.781122	−161.798971	−161.796089	−161.776976	−161.744686	−161.700452

Table S339: Total energies in E_h for the Na atom in the AHGBSP2-9 basis set in fully uncontracted form, employing the real-orbital approximation.

	$0.00B_0$	$0.10B_0$	$0.20B_0$	$0.30B_0$	$0.40B_0$	$0.50B_0$	$0.60B_0$
$\sigma^{4,3}\pi_+^{1,1}\pi_-^{1,1}$	−161.858953	−161.888136	−161.885746	−161.861941	−161.821375	−161.766417	−161.698463
$\sigma^{3,3}\pi_+^{1,1}\pi_-^{2,1}$	−161.786424	−161.848874	−161.867592	−161.861319	−161.835897	−161.794106	−161.737656
$\sigma^{3,3}\pi_+^{2,1}\pi_-^{1,1}$	−161.786424	−161.748874	−161.667592	−161.561319	−161.435897	−161.294106	−161.137656
$\sigma^{3,3}\pi_+^{1,1}\pi_-^{1,1}\delta_-^{1,0}$	−161.732629	−161.808918	−161.835060	−161.840300	−161.830336	−161.807590	−161.773286
$\sigma^{3,3}\pi_+^{1,1}\pi_-^{1,1}\phi_-^{1,0}$	−161.708212	−161.781217	−161.799149	−161.796133	−161.777356	−161.745086	−161.700461

Table S340: Total energies in E_h for the Na atom in the AHGBSP3-5 basis set in fully uncontracted form, employing the real-orbital approximation.

	$0.00B_0$	$0.10B_0$	$0.20B_0$	$0.30B_0$	$0.40B_0$	$0.50B_0$	$0.60B_0$
$\sigma^{4,3}\pi_+^{1,1}\pi_-^{1,1}$	−161.855796	−161.884938	−161.882710	−161.859606	−161.820672	−161.768867	−161.705813
$\sigma^{3,3}\pi_+^{1,1}\pi_-^{2,1}$	−161.783276	−161.845656	−161.864342	−161.857424	−161.832015	−161.790601	−161.733982
$\sigma^{3,3}\pi_+^{2,1}\pi_-^{1,1}$	−161.783276	−161.745656	−161.664342	−161.557424	−161.432015	−161.290601	−161.133982
$\sigma^{3,3}\pi_+^{1,1}\pi_-^{1,1}\delta_-^{1,0}$	−161.729483	−161.806884	−161.835635	−161.843447	−161.836207	−161.817408	−161.786403
$\sigma^{3,3}\pi_+^{1,1}\pi_-^{1,1}\phi_-^{1,0}$	−161.705065	−161.777801	−161.794392	−161.792970	−161.771444	−161.737352	−161.695271

Table S341: Total energies in E_h for the Na atom in the AHGBSP3-7 basis set in fully uncontracted form, employing the real-orbital approximation.

	$0.00B_0$	$0.10B_0$	$0.20B_0$	$0.30B_0$	$0.40B_0$	$0.50B_0$	$0.60B_0$
$\sigma^{4,3}\pi_+^{1,1}\pi_-^{1,1}$	−161.858915	−161.888098	−161.885850	−161.862757	−161.824072	−161.772532	−161.709599
$\sigma^{3,3}\pi_+^{1,1}\pi_-^{2,1}$	−161.786386	−161.848835	−161.867529	−161.861277	−161.835782	−161.793930	−161.737524
$\sigma^{3,3}\pi_+^{2,1}\pi_-^{1,1}$	−161.786386	−161.748835	−161.667529	−161.561277	−161.435782	−161.293930	−161.137524
$\sigma^{3,3}\pi_+^{1,1}\pi_-^{1,1}\delta_-^{1,0}$	−161.732591	−161.810044	−161.838776	−161.847118	−161.840259	−161.820784	−161.789695
$\sigma^{3,3}\pi_+^{1,1}\pi_-^{1,1}\phi_-^{1,0}$	−161.708174	−161.781122	−161.798971	−161.796089	−161.776976	−161.744686	−161.700452

Table S342: Total energies in E_h for the Na atom in the AHGBSP3-9 basis set in fully uncontracted form, employing the real-orbital approximation.

	$0.00B_0$	$0.10B_0$	$0.20B_0$	$0.30B_0$	$0.40B_0$	$0.50B_0$	$0.60B_0$
$\sigma^{4,3}\pi_+^{1,1}\pi_-^{1,1}$	−161.858953	−161.888140	−161.885890	−161.862824	−161.824142	−161.772581	−161.709644
$\sigma^{3,3}\pi_+^{1,1}\pi_-^{2,1}$	−161.786424	−161.848874	−161.867592	−161.861319	−161.835897	−161.794106	−161.737656
$\sigma^{3,3}\pi_+^{2,1}\pi_-^{1,1}$	−161.786424	−161.748874	−161.667592	−161.561319	−161.435897	−161.294106	−161.137656
$\sigma^{3,3}\pi_+^{1,1}\pi_-^{1,1}\delta_-^{1,0}$	−161.732629	−161.810091	−161.838867	−161.847183	−161.840438	−161.820901	−161.789772
$\sigma^{3,3}\pi_+^{1,1}\pi_-^{1,1}\phi_-^{1,0}$	−161.708212	−161.781217	−161.799149	−161.796133	−161.777356	−161.745086	−161.700462

Table S343: Total energies in E_h for the Na atom in the 6-311++G(3df,3pd) basis set in fully uncontracted form, employing the real-orbital approximation.

	$0.00B_0$	$0.10B_0$	$0.20B_0$	$0.30B_0$	$0.40B_0$	$0.50B_0$	$0.60B_0$
$\sigma^{4,3}\pi_+^{1,1}\pi_-^{1,1}$	−161.854042	−161.883170	−161.880677	−161.854133	−161.805168	−161.736548	−161.652266
$\sigma^{3,3}\pi_+^{1,1}\pi_-^{2,1}$	−161.781508	−161.843218	−161.856450	−161.838159	−161.805810	−161.767064	−161.718453
$\sigma^{3,3}\pi_+^{2,1}\pi_-^{1,1}$	−161.781508	−161.743218	−161.656450	−161.538159	−161.405810	−161.267064	−161.118453
$\sigma^{3,3}\pi_+^{1,1}\pi_-^{1,1}\delta_-^{1,0}$	−161.697817	−161.800797	−161.819629	−161.800604	−161.796375	−161.790216	−161.765060
$\sigma^{3,3}\pi_+^{1,1}\pi_-^{1,1}\phi_-^{1,0}$	−161.281461	−161.459449	−161.593440	−161.683507	−161.729766	−161.732366	−161.691483

Table S344: Total energies in E_h for the Na atom in the def2-TZVP basis set in fully uncontracted form, employing the real-orbital approximation.

	$0.00B_0$	$0.10B_0$	$0.20B_0$	$0.30B_0$	$0.40B_0$	$0.50B_0$	$0.60B_0$
$\sigma^{4,3}\pi_+^{1,1}\pi_-^{1,1}$	−161.852507	−161.881322	−161.876383	−161.846463	−161.797804	−161.734637	−161.657844
$\sigma^{3,3}\pi_+^{1,1}\pi_-^{2,1}$	−161.778869	−161.841185	−161.855164	−161.846838	−161.812246	−161.749282	−161.660964
$\sigma^{3,3}\pi_+^{2,1}\pi_-^{1,1}$	−161.778869	−161.741185	−161.655164	−161.546838	−161.412246	−161.249282	−161.060964
$\sigma^{3,3}\pi_+^{1,1}\pi_-^{1,1}\delta_-^{1,0}$	−161.594101	−161.720007	−161.798175	−161.830144	−161.819248	−161.772209	−161.701737
$\sigma^{3,3}\pi_+^{1,1}\pi_-^{1,1}\phi_-^{1,0}$							

Table S345: Total energies in E_h for the Mg atom in the cc-pVDZ basis set in fully uncontracted form, employing the real-orbital approximation.

	$0.00B_0$	$0.10B_0$	$0.20B_0$	$0.30B_0$	$0.40B_0$	$0.50B_0$	$0.60B_0$
$\sigma^{4,4}\pi_+^{1,1}\pi_-^{1,1}$	−199.608297	−199.584207	−199.517367	−199.418904	−199.297096	−199.155728	−198.996997
$\sigma^{5,3}\pi_+^{1,1}\pi_-^{1,1}$	−199.541365	−199.619370	−199.658162	−199.667762	−199.655650	−199.624568	−199.575167
$\sigma^{4,3}\pi_+^{2,1}\pi_-^{1,1}$	−199.540430	−199.558935	−199.525019	−199.456040	−199.359061	−199.235084	−199.085355
$\sigma^{4,3}\pi_+^{1,1}\pi_-^{2,1}$	−199.540430	−199.658935	−199.725019	−199.756040	−199.759061	−199.735084	−199.685355
$\sigma^{4,3}\pi_+^{1,1}\pi_-^{1,1}\delta_-^{1,0}$	−199.187013	−199.363383	−199.495292	−199.588013	−199.645174	−199.668657	−199.659837
$\sigma^{3,3}\pi_+^{1,1}\pi_-^{2,1}\delta_-^{1,0}$	−199.069920	−199.286893	−199.447520	−199.566015	−199.645559	−199.684834	−199.683533

Table S346: Total energies in E_h for the Mg atom in the cc-pVTZ basis set in fully uncontracted form, employing the real-orbital approximation.

	$0.00B_0$	$0.10B_0$	$0.20B_0$	$0.30B_0$	$0.40B_0$	$0.50B_0$	$0.60B_0$
$\sigma^{4,4}\pi_+^{1,1}\pi_-^{1,1}$	−199.613347	−199.589394	−199.523970	−199.427950	−199.309197	−199.172598	−199.020693
$\sigma^{5,3}\pi_+^{1,1}\pi_-^{1,1}$	−199.547248	−199.625647	−199.666493	−199.679237	−199.670274	−199.643860	−199.603181
$\sigma^{4,3}\pi_+^{2,1}\pi_-^{1,1}$	−199.545926	−199.564630	−199.532468	−199.463960	−199.367878	−199.251298	−199.118883
$\sigma^{4,3}\pi_+^{1,1}\pi_-^{2,1}$	−199.545926	−199.664630	−199.732468	−199.763960	−199.767878	−199.751298	−199.718883
$\sigma^{4,3}\pi_+^{1,1}\pi_-^{1,1}\delta_-^{1,0}$	−199.294842	−199.466549	−199.584922	−199.656969	−199.691059	−199.696768	−199.683643
$\sigma^{3,3}\pi_+^{1,1}\pi_-^{2,1}\delta_-^{1,0}$	−199.171578	−199.384732	−199.534025	−199.631630	−199.687189	−199.712077	−199.717244

Table S347: Total energies in E_h for the Mg atom in the cc-pVQZ basis set in fully uncontracted form, employing the real-orbital approximation.

	$0.00B_0$	$0.10B_0$	$0.20B_0$	$0.30B_0$	$0.40B_0$	$0.50B_0$	$0.60B_0$
$\sigma^{4,4}\pi_+^{1,1}\pi_-^{1,1}$	−199.614233	−199.590342	−199.525437	−199.430312	−199.312240	−199.175627	−199.023248
$\sigma^{5,3}\pi_+^{1,1}\pi_-^{1,1}$	−199.548349	−199.626935	−199.668574	−199.682757	−199.676573	−199.654497	−199.619022
$\sigma^{4,3}\pi_+^{2,1}\pi_-^{1,1}$	−199.546883	−199.565669	−199.533727	−199.466344	−199.373163	−199.259512	−199.128567
$\sigma^{4,3}\pi_+^{1,1}\pi_-^{2,1}$	−199.546883	−199.665669	−199.733727	−199.766344	−199.773163	−199.759512	−199.728567
$\sigma^{4,3}\pi_+^{1,1}\pi_-^{1,1}\delta_-^{1,0}$	−199.342757	−199.509896	−199.616073	−199.673120	−199.696542	−199.700245	−199.691271
$\sigma^{3,3}\pi_+^{1,1}\pi_-^{2,1}\delta_-^{1,0}$	−199.214773	−199.423911	−199.561901	−199.646510	−199.695277	−199.721929	−199.733331

Table S348: Total energies in E_h for the Mg atom in the cc-pV5Z basis set in fully uncontracted form, employing the real-orbital approximation.

	$0.00B_0$	$0.10B_0$	$0.20B_0$	$0.30B_0$	$0.40B_0$	$0.50B_0$	$0.60B_0$
$\sigma^{4,4}\pi_+^{1,1}\pi_-^{1,1}$	−199.614605	−199.590699	−199.525647	−199.430266	−199.312197	−199.176169	−199.025061
$\sigma^{5,3}\pi_+^{1,1}\pi_-^{1,1}$	−199.548696	−199.627297	−199.669230	−199.684436	−199.679868	−199.659435	−199.625321
$\sigma^{4,3}\pi_+^{2,1}\pi_-^{1,1}$	−199.547252	−199.566069	−199.534323	−199.467392	−199.374639	−199.261457	−199.131002
$\sigma^{4,3}\pi_+^{1,1}\pi_-^{2,1}$	−199.547252	−199.666069	−199.734323	−199.767392	−199.774639	−199.761457	−199.731002
$\sigma^{4,3}\pi_+^{1,1}\pi_-^{1,1}\delta_-^{1,0}$	−199.329894	−199.498852	−199.609863	−199.672870	−199.701039	−199.707895	−199.702245
$\sigma^{3,3}\pi_+^{1,1}\pi_-^{2,1}\delta_-^{1,0}$	−199.203800	−199.414593	−199.557298	−199.647887	−199.701368	−199.731088	−199.745227

Table S349: Total energies in E_h for the Mg atom in the aug-cc-pVDZ basis set in fully uncontracted form, employing the real-orbital approximation.

	$0.00B_0$	$0.10B_0$	$0.20B_0$	$0.30B_0$	$0.40B_0$	$0.50B_0$	$0.60B_0$
$\sigma^{4,4}\pi_+^{1,1}\pi_-^{1,1}$	−199.608340	−199.584486	−199.519803	−199.424892	−199.305959	−199.166022	−199.007522
$\sigma^{5,3}\pi_+^{1,1}\pi_-^{1,1}$	−199.542241	−199.620685	−199.661048	−199.672239	−199.660801	−199.629607	−199.579722
$\sigma^{4,3}\pi_+^{2,1}\pi_-^{1,1}$	−199.540722	−199.559099	−199.525743	−199.457600	−199.361540	−199.238476	−199.089542
$\sigma^{4,3}\pi_+^{1,1}\pi_-^{2,1}$	−199.540722	−199.659099	−199.725743	−199.757600	−199.761540	−199.738476	−199.689542
$\sigma^{4,3}\pi_+^{1,1}\pi_-^{1,1}\delta_-^{1,0}$	−199.383442	−199.541588	−199.624490	−199.658699	−199.675742	−199.680599	−199.664963
$\sigma^{3,3}\pi_+^{1,1}\pi_-^{2,1}\delta_-^{1,0}$	−199.247818	−199.448316	−199.564134	−199.628499	−199.670517	−199.692118	−199.684669

Table S350: Total energies in E_h for the Mg atom in the aug-cc-pVTZ basis set in fully uncontracted form, employing the real-orbital approximation.

	$0.00B_0$	$0.10B_0$	$0.20B_0$	$0.30B_0$	$0.40B_0$	$0.50B_0$	$0.60B_0$
$\sigma^{4,4}\pi_+^{1,1}\pi_-^{1,1}$	−199.613351	−199.589550	−199.525039	−199.429940	−199.311276	−199.174020	−199.021286
$\sigma^{5,3}\pi_+^{1,1}\pi_-^{1,1}$	−199.547555	−199.626288	−199.668864	−199.685155	−199.681035	−199.659617	−199.622852
$\sigma^{4,3}\pi_+^{2,1}\pi_-^{1,1}$	−199.546007	−199.564729	−199.533408	−199.466948	−199.373338	−199.258387	−199.126158
$\sigma^{4,3}\pi_+^{1,1}\pi_-^{2,1}$	−199.546007	−199.664729	−199.733408	−199.766948	−199.773338	−199.758387	−199.726158
$\sigma^{4,3}\pi_+^{1,1}\pi_-^{1,1}\delta_-^{1,0}$	−199.404855	−199.555504	−199.628237	−199.669114	−199.692956	−199.697245	−199.684782
$\sigma^{3,3}\pi_+^{1,1}\pi_-^{2,1}\delta_-^{1,0}$	−199.264823	−199.459749	−199.570605	−199.643512	−199.692657	−199.718434	−199.725084

Table S351: Total energies in E_h for the Mg atom in the aug-cc-pVQZ basis set in fully uncontracted form, employing the real-orbital approximation.

	$0.00B_0$	$0.10B_0$	$0.20B_0$	$0.30B_0$	$0.40B_0$	$0.50B_0$	$0.60B_0$
$\sigma^{4,4}\pi_+^{1,1}\pi_-^{1,1}$	−199.614234	−199.590440	−199.525927	−199.430994	−199.313127	−199.177468	−199.027320
$\sigma^{5,3}\pi_+^{1,1}\pi_-^{1,1}$	−199.548473	−199.627310	−199.670390	−199.687073	−199.683214	−199.662613	−199.627963
$\sigma^{4,3}\pi_+^{2,1}\pi_-^{1,1}$	−199.546913	−199.565755	−199.534526	−199.468096	−199.375292	−199.261632	−199.130912
$\sigma^{4,3}\pi_+^{1,1}\pi_-^{2,1}$	−199.546913	−199.665755	−199.734526	−199.768096	−199.775292	−199.761632	−199.730912
$\sigma^{4,3}\pi_+^{1,1}\pi_-^{1,1}\delta_-^{1,0}$	−199.414467	−199.558940	−199.630562	−199.676731	−199.701353	−199.708254	−199.701518
$\sigma^{3,3}\pi_+^{1,1}\pi_-^{2,1}\delta_-^{1,0}$	−199.271390	−199.462145	−199.573848	−199.651188	−199.701830	−199.730968	−199.742965

Table S352: Total energies in E_h for the Mg atom in the aug-cc-pV5Z basis set in fully uncontracted form, employing the real-orbital approximation.

$\sigma^{4,4}\pi_+^{1,1}\pi_-^{1,1}$
$\sigma^{5,3}\pi_+^{1,1}\pi_-^{1,1}$
$\sigma^{4,3}\pi_+^{2,1}\pi_-^{1,1}$
$\sigma^{4,3}\pi_+^{1,1}\pi_-^{2,1}$
$\sigma^{4,3}\pi_+^{1,1}\pi_-^{1,1}\delta_-^{1,0}$
$\sigma^{3,3}\pi_+^{1,1}\pi_-^{2,1}\delta_-^{1,0}$

Table S353: Total energies in E_h for the Mg atom in the HGBSP1-5 basis set in fully uncontracted form, employing the real-orbital approximation.

	$0.00B_0$	$0.10B_0$	$0.20B_0$	$0.30B_0$	$0.40B_0$	$0.50B_0$	$0.60B_0$
$\sigma^{4,4}\pi_+^{1,1}\pi_-^{1,1}$	−199.603043	−199.581718	−199.520780	−199.427259	−199.308551	−199.170326	−199.016345
$\sigma^{5,3}\pi_+^{1,1}\pi_-^{1,1}$	−199.542797	−199.622087	−199.664240	−199.676387	−199.664516	−199.633239	−199.585663
$\sigma^{4,3}\pi_+^{2,1}\pi_-^{1,1}$	−199.541254	−199.560806	−199.529604	−199.460764	−199.363997	−199.245363	−199.107955
$\sigma^{4,3}\pi_+^{1,1}\pi_-^{2,1}$	−199.541254	−199.660806	−199.729604	−199.760764	−199.763997	−199.745363	−199.707955
$\sigma^{4,3}\pi_+^{1,1}\pi_-^{1,1}\delta_-^{1,0}$	−199.426089	−199.555507	−199.628879	−199.672848	−199.694296	−199.699775	−199.691429
$\sigma^{3,3}\pi_+^{1,1}\pi_-^{2,1}\delta_-^{1,0}$	−199.276085	−199.458974	−199.571246	−199.644042	−199.689149	−199.714030	−199.720637

Table S354: Total energies in E_h for the Mg atom in the HGBSP1-7 basis set in fully uncontracted form, employing the real-orbital approximation.

	$0.00B_0$	$0.10B_0$	$0.20B_0$	$0.30B_0$	$0.40B_0$	$0.50B_0$	$0.60B_0$
$\sigma^{4,4}\pi_+^{1,1}\pi_-^{1,1}$	−199.614585	−199.590789	−199.526295	−199.431366	−199.313080	−199.175988	−199.023036
$\sigma^{5,3}\pi_+^{1,1}\pi_-^{1,1}$	−199.548821	−199.627427	−199.668564	−199.680556	−199.669387	−199.638914	−199.591717
$\sigma^{4,3}\pi_+^{2,1}\pi_-^{1,1}$	−199.547267	−199.566012	−199.533843	−199.465328	−199.369187	−199.250848	−199.113904
$\sigma^{4,3}\pi_+^{1,1}\pi_-^{2,1}$	−199.547267	−199.666012	−199.733843	−199.765328	−199.769187	−199.750848	−199.713904
$\sigma^{4,3}\pi_+^{1,1}\pi_-^{1,1}\delta_-^{1,0}$	−199.430369	−199.559909	−199.633100	−199.677162	−199.699692	−199.705359	−199.697004
$\sigma^{3,3}\pi_+^{1,1}\pi_-^{2,1}\delta_-^{1,0}$	−199.280036	−199.462941	−199.575305	−199.648688	−199.694334	−199.718398	−199.724560

Table S355: Total energies in E_h for the Mg atom in the HGBSP1-9 basis set in fully uncontracted form, employing the real-orbital approximation.

	$0.00B_0$	$0.10B_0$	$0.20B_0$	$0.30B_0$	$0.40B_0$	$0.50B_0$	$0.60B_0$
$\sigma^{4,4}\pi_+^{1,1}\pi_-^{1,1}$	−199.614633	−199.590845	−199.526347	−199.431448	−199.313203	−199.176105	−199.023141
$\sigma^{5,3}\pi_+^{1,1}\pi_-^{1,1}$	−199.548871	−199.627478	−199.668620	−199.680632	−199.669473	−199.639025	−199.591883
$\sigma^{4,3}\pi_+^{2,1}\pi_-^{1,1}$	−199.547317	−199.566063	−199.533906	−199.465409	−199.369349	−199.251059	−199.114053
$\sigma^{4,3}\pi_+^{1,1}\pi_-^{2,1}$	−199.547317	−199.666063	−199.733906	−199.765409	−199.769349	−199.751059	−199.714053
$\sigma^{4,3}\pi_+^{1,1}\pi_-^{1,1}\delta_-^{1,0}$	−199.430420	−199.559973	−199.633197	−199.677232	−199.699878	−199.705564	−199.697097
$\sigma^{3,3}\pi_+^{1,1}\pi_-^{2,1}\delta_-^{1,0}$	−199.280085	−199.462997	−199.575396	−199.648737	−199.694542	−199.718702	−199.724727

Table S356: Total energies in E_h for the Mg atom in the HGBSP2-5 basis set in fully uncontracted form, employing the real-orbital approximation.

	$0.00B_0$	$0.10B_0$	$0.20B_0$	$0.30B_0$	$0.40B_0$	$0.50B_0$	$0.60B_0$
$\sigma^{4,4}\pi_+^{1,1}\pi_-^{1,1}$	−199.603044	−199.581718	−199.520781	−199.427262	−199.308559	−199.170347	−199.016390
$\sigma^{5,3}\pi_+^{1,1}\pi_-^{1,1}$	−199.542831	−199.622417	−199.666582	−199.683268	−199.678465	−199.656284	−199.619339
$\sigma^{4,3}\pi_+^{2,1}\pi_-^{1,1}$	−199.541263	−199.560941	−199.530711	−199.464099	−199.370681	−199.256347	−199.124344
$\sigma^{4,3}\pi_+^{1,1}\pi_-^{2,1}$	−199.541263	−199.660941	−199.730711	−199.764099	−199.770681	−199.756347	−199.724344
$\sigma^{4,3}\pi_+^{1,1}\pi_-^{1,1}\delta_-^{1,0}$	−199.398995	−199.555509	−199.628887	−199.672868	−199.694341	−199.699862	−199.691582
$\sigma^{3,3}\pi_+^{1,1}\pi_-^{2,1}\delta_-^{1,0}$	−199.276104	−199.459047	−199.572091	−199.646878	−199.695132	−199.724173	−199.736114

Table S357: Total energies in E_h for the Mg atom in the HGBSP2-7 basis set in fully uncontracted form, employing the real-orbital approximation.

	$0.00B_0$	$0.10B_0$	$0.20B_0$	$0.30B_0$	$0.40B_0$	$0.50B_0$	$0.60B_0$
$\sigma^{4,4}\pi_+^{1,1}\pi_-^{1,1}$	−199.614585	−199.590789	−199.526295	−199.431368	−199.313088	−199.176009	−199.023081
$\sigma^{5,3}\pi_+^{1,1}\pi_-^{1,1}$	−199.548855	−199.627762	−199.670940	−199.687406	−199.683033	−199.661460	−199.625029
$\sigma^{4,3}\pi_+^{2,1}\pi_-^{1,1}$	−199.547275	−199.566148	−199.534954	−199.468580	−199.375775	−199.261879	−199.130352
$\sigma^{4,3}\pi_+^{1,1}\pi_-^{2,1}$	−199.547275	−199.666148	−199.734954	−199.768580	−199.775775	−199.761879	−199.730352
$\sigma^{4,3}\pi_+^{1,1}\pi_-^{1,1}\delta_-^{1,0}$	−199.430369	−199.559910	−199.633107	−199.677182	−199.699737	−199.705445	−199.697157
$\sigma^{3,3}\pi_+^{1,1}\pi_-^{2,1}\delta_-^{1,0}$	−199.280055	−199.463013	−199.576146	−199.651458	−199.700256	−199.728637	−199.740163

Table S358: Total energies in E_h for the Mg atom in the HGBSP2-9 basis set in fully uncontracted form, employing the real-orbital approximation.

	$0.00B_0$	$0.10B_0$	$0.20B_0$	$0.30B_0$	$0.40B_0$	$0.50B_0$	$0.60B_0$
$\sigma^{4,4}\pi_+^{1,1}\pi_-^{1,1}$	−199.614633	−199.590845	−199.526347	−199.431450	−199.313211	−199.176125	−199.023185
$\sigma^{5,3}\pi_+^{1,1}\pi_-^{1,1}$	−199.548905	−199.627813	−199.670994	−199.687475	−199.683114	−199.661541	−199.625114
$\sigma^{4,3}\pi_+^{2,1}\pi_-^{1,1}$	−199.547325	−199.566200	−199.535015	−199.468661	−199.375909	−199.262038	−199.130486
$\sigma^{4,3}\pi_+^{1,1}\pi_-^{2,1}$	−199.547325	−199.666200	−199.735015	−199.768661	−199.775909	−199.762038	−199.730486
$\sigma^{4,3}\pi_+^{1,1}\pi_-^{1,1}\delta_-^{1,0}$	−199.430421	−199.559975	−199.633204	−199.677252	−199.699922	−199.705650	−199.697249
$\sigma^{3,3}\pi_+^{1,1}\pi_-^{2,1}\delta_-^{1,0}$	−199.280105	−199.463069	−199.576236	−199.651508	−199.700439	−199.728885	−199.740311

Table S359: Total energies in E_h for the Mg atom in the HGBSP3-5 basis set in fully uncontracted form, employing the real-orbital approximation.

	$0.00B_0$	$0.10B_0$	$0.20B_0$	$0.30B_0$	$0.40B_0$	$0.50B_0$	$0.60B_0$
$\sigma^{4,4}\pi_+^{1,1}\pi_-^{1,1}$	−199.603043	−199.581718	−199.520802	−199.427512	−199.309728	−199.173672	−199.023410
$\sigma^{5,3}\pi_+^{1,1}\pi_-^{1,1}$	−199.542831	−199.622419	−199.666639	−199.683595	−199.679565	−199.658940	−199.624487
$\sigma^{4,3}\pi_+^{2,1}\pi_-^{1,1}$	−199.541263	−199.560941	−199.530711	−199.464134	−199.370956	−199.257328	−199.126704
$\sigma^{4,3}\pi_+^{1,1}\pi_-^{2,1}$	−199.541263	−199.660941	−199.730711	−199.764134	−199.770956	−199.757328	−199.726704
$\sigma^{4,3}\pi_+^{1,1}\pi_-^{1,1}\delta_-^{1,0}$	−199.426089	−199.556293	−199.631314	−199.677400	−199.701489	−199.710161	−199.706106
$\sigma^{3,3}\pi_+^{1,1}\pi_-^{2,1}\delta_-^{1,0}$	−199.276116	−199.459703	−199.574373	−199.651411	−199.702214	−199.733766	−199.748380

Table S360: Total energies in E_h for the Mg atom in the HGBSP3-7 basis set in fully uncontracted form, employing the real-orbital approximation.

	$0.00B_0$	$0.10B_0$	$0.20B_0$	$0.30B_0$	$0.40B_0$	$0.50B_0$	$0.60B_0$
$\sigma^{4,4}\pi_+^{1,1}\pi_-^{1,1}$	−199.614585	−199.590790	−199.526333	−199.431659	−199.314195	−199.178887	−199.029026
$\sigma^{5,3}\pi_+^{1,1}\pi_-^{1,1}$	−199.548855	−199.627765	−199.671004	−199.687742	−199.684074	−199.663875	−199.629681
$\sigma^{4,3}\pi_+^{2,1}\pi_-^{1,1}$	−199.547275	−199.566149	−199.534956	−199.468614	−199.375991	−199.262624	−199.132207
$\sigma^{4,3}\pi_+^{1,1}\pi_-^{2,1}$	−199.547275	−199.666149	−199.734956	−199.768614	−199.775991	−199.762624	−199.732207
$\sigma^{4,3}\pi_+^{1,1}\pi_-^{1,1}\delta_-^{1,0}$	−199.430369	−199.560684	−199.635539	−199.681648	−199.706703	−199.715505	−199.711159
$\sigma^{3,3}\pi_+^{1,1}\pi_-^{2,1}\delta_-^{1,0}$	−199.280066	−199.463666	−199.578438	−199.655914	−199.707220	−199.738253	−199.752487

Table S361: Total energies in E_h for the Mg atom in the HGBSP3-9 basis set in fully uncontracted form, employing the real-orbital approximation.

	$0.00B_0$	$0.10B_0$	$0.20B_0$	$0.30B_0$	$0.40B_0$	$0.50B_0$	$0.60B_0$
$\sigma^{4,4}\pi_+^{1,1}\pi_-^{1,1}$	−199.614634	−199.590845	−199.526384	−199.431743	−199.314317	−199.178978	−199.029080
$\sigma^{5,3}\pi_+^{1,1}\pi_-^{1,1}$	−199.548905	−199.627816	−199.671058	−199.687811	−199.684154	−199.663945	−199.629745
$\sigma^{4,3}\pi_+^{2,1}\pi_-^{1,1}$	−199.547325	−199.566200	−199.535017	−199.468696	−199.376124	−199.262774	−199.132326
$\sigma^{4,3}\pi_+^{1,1}\pi_-^{2,1}$	−199.547325	−199.666200	−199.735017	−199.768696	−199.776124	−199.762774	−199.732326
$\sigma^{4,3}\pi_+^{1,1}\pi_-^{1,1}\delta_-^{1,0}$	−199.430421	−199.560746	−199.635627	−199.681722	−199.706860	−199.715672	−199.711246
$\sigma^{3,3}\pi_+^{1,1}\pi_-^{2,1}\delta_-^{1,0}$	−199.280116	−199.463721	−199.578522	−199.655968	−199.707380	−199.738478	−199.752647

Table S362: Total energies in E_h for the Mg atom in the AHGBSP1-5 basis set in fully uncontracted form, employing the real-orbital approximation.

	$0.00B_0$	$0.10B_0$	$0.20B_0$	$0.30B_0$	$0.40B_0$	$0.50B_0$	$0.60B_0$
$\sigma^{4,4}\pi_+^{1,1}\pi_-^{1,1}$	−199.610642	−199.586836	−199.522339	−199.427363	−199.308751	−199.171085	−199.017597
$\sigma^{5,3}\pi_+^{1,1}\pi_-^{1,1}$	−199.544875	−199.623475	−199.664597	−199.676414	−199.664777	−199.633877	−199.586589
$\sigma^{4,3}\pi_+^{2,1}\pi_-^{1,1}$	−199.543321	−199.562059	−199.529812	−199.460849	−199.364437	−199.246136	−199.108906
$\sigma^{4,3}\pi_+^{1,1}\pi_-^{2,1}$	−199.543321	−199.662059	−199.729812	−199.760849	−199.764437	−199.746136	−199.708906
$\sigma^{4,3}\pi_+^{1,1}\pi_-^{1,1}\delta_-^{1,0}$	−199.426383	−199.555943	−199.629011	−199.672894	−199.694595	−199.700350	−199.692205
$\sigma^{3,3}\pi_+^{1,1}\pi_-^{2,1}\delta_-^{1,0}$	−199.276097	−199.458988	−199.571267	−199.644085	−199.689206	−199.714060	−199.720649

Table S363: Total energies in E_h for the Mg atom in the AHGBSP1-7 basis set in fully uncontracted form, employing the real-orbital approximation.

	$0.00B_0$	$0.10B_0$	$0.20B_0$	$0.30B_0$	$0.40B_0$	$0.50B_0$	$0.60B_0$
$\sigma^{4,4}\pi_+^{1,1}\pi_-^{1,1}$	−199.614586	−199.590794	−199.526296	−199.431372	−199.313099	−199.176010	−199.023053
$\sigma^{5,3}\pi_+^{1,1}\pi_-^{1,1}$	−199.548822	−199.627428	−199.668565	−199.680563	−199.669399	−199.638929	−199.591734
$\sigma^{4,3}\pi_+^{2,1}\pi_-^{1,1}$	−199.547267	−199.566013	−199.533846	−199.465338	−199.369207	−199.250870	−199.113917
$\sigma^{4,3}\pi_+^{1,1}\pi_-^{2,1}$	−199.547267	−199.666013	−199.733846	−199.765338	−199.769207	−199.750870	−199.713917
$\sigma^{4,3}\pi_+^{1,1}\pi_-^{1,1}\delta_-^{1,0}$	−199.430370	−199.559910	−199.633102	−199.677169	−199.699705	−199.705371	−199.697011
$\sigma^{3,3}\pi_+^{1,1}\pi_-^{2,1}\delta_-^{1,0}$	−199.280036	−199.462942	−199.575308	−199.648689	−199.694341	−199.718411	−199.724569

Table S364: Total energies in E_h for the Mg atom in the AHGBSP1-9 basis set in fully uncontracted form, employing the real-orbital approximation.

	$0.00B_0$	$0.10B_0$	$0.20B_0$	$0.30B_0$	$0.40B_0$	$0.50B_0$	$0.60B_0$
$\sigma^{4,4}\pi_+^{1,1}\pi_-^{1,1}$	−199.614636	−199.590845	−199.526347	−199.431449	−199.313204	−199.176105	−199.023141
$\sigma^{5,3}\pi_+^{1,1}\pi_-^{1,1}$	−199.548872	−199.627478	−199.668620	−199.680633	−199.669474	−199.639026	−199.591883
$\sigma^{4,3}\pi_+^{2,1}\pi_-^{1,1}$	−199.547317	−199.566064	−199.533907	−199.465411	−199.369349	−199.251060	−199.114056
$\sigma^{4,3}\pi_+^{1,1}\pi_-^{2,1}$	−199.547317	−199.666064	−199.733907	−199.765411	−199.769349	−199.751060	−199.714056
$\sigma^{4,3}\pi_+^{1,1}\pi_-^{1,1}\delta_-^{1,0}$	−199.430421	−199.559973	−199.633197	−199.677233	−199.699878	−199.705564	−199.697097
$\sigma^{3,3}\pi_+^{1,1}\pi_-^{2,1}\delta_-^{1,0}$	−199.280085	−199.462997	−199.575397	−199.648738	−199.694542	−199.718703	−199.724729

Table S365: Total energies in E_h for the Mg atom in the AHGBSP2-5 basis set in fully uncontracted form, employing the real-orbital approximation.

	$0.00B_0$	$0.10B_0$	$0.20B_0$	$0.30B_0$	$0.40B_0$	$0.50B_0$	$0.60B_0$
$\sigma^{4,4}\pi_+^{1,1}\pi_-^{1,1}$	−199.610642	−199.586836	−199.522340	−199.427366	−199.308759	−199.171106	−199.017642
$\sigma^{5,3}\pi_+^{1,1}\pi_-^{1,1}$	−199.544908	−199.623810	−199.666967	−199.683292	−199.678636	−199.656755	−199.620079
$\sigma^{4,3}\pi_+^{2,1}\pi_-^{1,1}$	−199.543329	−199.562196	−199.530925	−199.464169	−199.371090	−199.257092	−199.125272
$\sigma^{4,3}\pi_+^{1,1}\pi_-^{2,1}$	−199.543329	−199.662196	−199.730925	−199.764169	−199.771090	−199.757092	−199.725272
$\sigma^{4,3}\pi_+^{1,1}\pi_-^{1,1}\delta_-^{1,0}$	−199.426383	−199.555945	−199.629018	−199.672914	−199.694640	−199.700437	−199.692358
$\sigma^{3,3}\pi_+^{1,1}\pi_-^{2,1}\delta_-^{1,0}$	−199.276116	−199.459061	−199.572112	−199.646913	−199.695185	−199.724206	−199.736128

Table S366: Total energies in E_h for the Mg atom in the AHGBSP2-7 basis set in fully uncontracted form, employing the real-orbital approximation.

	$0.00B_0$	$0.10B_0$	$0.20B_0$	$0.30B_0$	$0.40B_0$	$0.50B_0$	$0.60B_0$
$\sigma^{4,4}\pi_+^{1,1}\pi_-^{1,1}$	−199.614586	−199.590794	−199.526296	−199.431375	−199.313107	−199.176030	−199.023097
$\sigma^{5,3}\pi_+^{1,1}\pi_-^{1,1}$	−199.548855	−199.627763	−199.670940	−199.687411	−199.683042	−199.661470	−199.625037
$\sigma^{4,3}\pi_+^{2,1}\pi_-^{1,1}$	−199.547276	−199.566149	−199.534956	−199.468589	−199.375792	−199.261896	−199.130362
$\sigma^{4,3}\pi_+^{1,1}\pi_-^{2,1}$	−199.547276	−199.666149	−199.734956	−199.768589	−199.775792	−199.761896	−199.730362
$\sigma^{4,3}\pi_+^{1,1}\pi_-^{1,1}\delta_-^{1,0}$	−199.430370	−199.559912	−199.633109	−199.677189	−199.699749	−199.705457	−199.697164
$\sigma^{3,3}\pi_+^{1,1}\pi_-^{2,1}\delta_-^{1,0}$	−199.280055	−199.463014	−199.576149	−199.651459	−199.700260	−199.728645	−199.740170

Table S367: Total energies in E_h for the Mg atom in the AHGBSP2-9 basis set in fully uncontracted form, employing the real-orbital approximation.

	$0.00B_0$	$0.10B_0$	$0.20B_0$	$0.30B_0$	$0.40B_0$	$0.50B_0$	$0.60B_0$
$\sigma^{4,4}\pi_+^{1,1}\pi_-^{1,1}$	−199.614636	−199.590845	−199.526347	−199.431451	−199.313212	−199.176125	−199.023185
$\sigma^{5,3}\pi_+^{1,1}\pi_-^{1,1}$	−199.548905	−199.627813	−199.670994	−199.687475	−199.683114	−199.661541	−199.625114
$\sigma^{4,3}\pi_+^{2,1}\pi_-^{1,1}$	−199.547326	−199.566200	−199.535015	−199.468662	−199.375909	−199.262039	−199.130487
$\sigma^{4,3}\pi_+^{1,1}\pi_-^{2,1}$	−199.547326	−199.666200	−199.735015	−199.768662	−199.775909	−199.762039	−199.730487
$\sigma^{4,3}\pi_+^{1,1}\pi_-^{1,1}\delta_-^{1,0}$	−199.430421	−199.559975	−199.633204	−199.677253	−199.699922	−199.705650	−199.697249
$\sigma^{3,3}\pi_+^{1,1}\pi_-^{2,1}\delta_-^{1,0}$	−199.280105	−199.463069	−199.576236	−199.651509	−199.700440	−199.728886	−199.740312

Table S368: Total energies in E_h for the Mg atom in the AHGBSP3-5 basis set in fully uncontracted form, employing the real-orbital approximation.

	$0.00B_0$	$0.10B_0$	$0.20B_0$	$0.30B_0$	$0.40B_0$	$0.50B_0$	$0.60B_0$
$\sigma^{4,4}\pi_+^{1,1}\pi_-^{1,1}$	−199.610642	−199.586836	−199.522378	−199.427653	−199.309843	−199.174025	−199.023853
$\sigma^{5,3}\pi_+^{1,1}\pi_-^{1,1}$	−199.544908	−199.623813	−199.667031	−199.683626	−199.679681	−199.659217	−199.624862
$\sigma^{4,3}\pi_+^{2,1}\pi_-^{1,1}$	−199.543329	−199.562196	−199.530927	−199.464201	−199.371304	−199.257872	−199.127257
$\sigma^{4,3}\pi_+^{1,1}\pi_-^{2,1}$	−199.543329	−199.662196	−199.730927	−199.764201	−199.771304	−199.757872	−199.727257
$\sigma^{4,3}\pi_+^{1,1}\pi_-^{1,1}\delta_-^{1,0}$	−199.426383	−199.556717	−199.631440	−199.677442	−199.701722	−199.710521	−199.706468
$\sigma^{3,3}\pi_+^{1,1}\pi_-^{2,1}\delta_-^{1,0}$	−199.276128	−199.459716	−199.574394	−199.651443	−199.702260	−199.733798	−199.748395

Table S369: Total energies in E_h for the Mg atom in the AHGBSP3-7 basis set in fully uncontracted form, employing the real-orbital approximation.

	$0.00B_0$	$0.10B_0$	$0.20B_0$	$0.30B_0$	$0.40B_0$	$0.50B_0$	$0.60B_0$
$\sigma^{4,4}\pi_+^{1,1}\pi_-^{1,1}$	−199.614586	−199.590794	−199.526333	−199.431668	−199.314212	−199.178896	−199.029027
$\sigma^{5,3}\pi_+^{1,1}\pi_-^{1,1}$	−199.548855	−199.627766	−199.671004	−199.687747	−199.684082	−199.663880	−199.629683
$\sigma^{4,3}\pi_+^{2,1}\pi_-^{1,1}$	−199.547276	−199.566150	−199.534958	−199.468623	−199.376006	−199.262636	−199.132214
$\sigma^{4,3}\pi_+^{1,1}\pi_-^{2,1}$	−199.547276	−199.666150	−199.734958	−199.768623	−199.776006	−199.762636	−199.732214
$\sigma^{4,3}\pi_+^{1,1}\pi_-^{1,1}\delta_-^{1,0}$	−199.430370	−199.560685	−199.635541	−199.681654	−199.706713	−199.715512	−199.711160
$\sigma^{3,3}\pi_+^{1,1}\pi_-^{2,1}\delta_-^{1,0}$	−199.280066	−199.463666	−199.578441	−199.655914	−199.707224	−199.738261	−199.752493

Table S370: Total energies in E_h for the Mg atom in the AHGBSP3-9 basis set in fully uncontracted form, employing the real-orbital approximation.

	$0.00B_0$	$0.10B_0$	$0.20B_0$	$0.30B_0$	$0.40B_0$	$0.50B_0$	$0.60B_0$
$\sigma^{4,4}\pi_+^{1,1}\pi_-^{1,1}$	−199.614636	−199.590846	−199.526384	−199.431745	−199.314317	−199.178979	−199.029083
$\sigma^{5,3}\pi_+^{1,1}\pi_-^{1,1}$	−199.548905	−199.627816	−199.671058	−199.687812	−199.684154	−199.663946	−199.629745
$\sigma^{4,3}\pi_+^{2,1}\pi_-^{1,1}$	−199.547326	−199.566200	−199.535017	−199.468697	−199.376124	−199.262775	−199.132329
$\sigma^{4,3}\pi_+^{1,1}\pi_-^{2,1}$	−199.547326	−199.666200	−199.735017	−199.768697	−199.776124	−199.762775	−199.732329
$\sigma^{4,3}\pi_+^{1,1}\pi_-^{1,1}\delta_-^{1,0}$	−199.430421	−199.560746	−199.635628	−199.681723	−199.706860	−199.715672	−199.711247
$\sigma^{3,3}\pi_+^{1,1}\pi_-^{2,1}\delta_-^{1,0}$	−199.280116	−199.463721	−199.578522	−199.655968	−199.707380	−199.738478	−199.752648

Table S371: Total energies in E_h for the Mg atom in the 6-311++G(3df,3pd) basis set in fully uncontracted form, employing the real-orbital approximation.

	$0.00B_0$	$0.10B_0$	$0.20B_0$	$0.30B_0$	$0.40B_0$	$0.50B_0$	$0.60B_0$
$\sigma^{4,4}\pi_+^{1,1}\pi_-^{1,1}$	−199.609310	−199.585469	−199.520695	−199.425103	−199.305178	−199.164857	−199.007127
$\sigma^{5,3}\pi_+^{1,1}\pi_-^{1,1}$	−199.543454	−199.622016	−199.663214	−199.676290	−199.668030	−199.642688	−199.603163
$\sigma^{4,3}\pi_+^{2,1}\pi_-^{1,1}$	−199.541949	−199.560619	−199.528563	−199.460829	−199.365515	−199.248309	−199.113779
$\sigma^{4,3}\pi_+^{1,1}\pi_-^{2,1}$	−199.541949	−199.660619	−199.728563	−199.760829	−199.765515	−199.748309	−199.713779
$\sigma^{4,3}\pi_+^{1,1}\pi_-^{1,1}\delta_-^{1,0}$	−199.403999	−199.552915	−199.615729	−199.646666	−199.676878	−199.691314	−199.682544
$\sigma^{3,3}\pi_+^{1,1}\pi_-^{2,1}\delta_-^{1,0}$	−199.263576	−199.456212	−199.556751	−199.621353	−199.676530	−199.712322	−199.724359

Table S372: Total energies in E_h for the Mg atom in the def2-TZVP basis set in fully uncontracted form, employing the real-orbital approximation.

	$0.00B_0$	$0.10B_0$	$0.20B_0$	$0.30B_0$	$0.40B_0$	$0.50B_0$	$0.60B_0$
$\sigma^{4,4}\pi_+^{1,1}\pi_-^{1,1}$	−199.607186	−199.583327	−199.518550	−199.423793	−199.304194	−199.161415	−198.997210
$\sigma^{5,3}\pi_+^{1,1}\pi_-^{1,1}$	−199.540415	−199.619270	−199.659354	−199.667935	−199.651864	−199.616097	−199.563477
$\sigma^{4,3}\pi_+^{2,1}\pi_-^{1,1}$	−199.538845	−199.557825	−199.522469	−199.449541	−199.351994	−199.233524	−199.093862
$\sigma^{4,3}\pi_+^{1,1}\pi_-^{2,1}$	−199.538845	−199.657825	−199.722469	−199.749541	−199.751994	−199.733524	−199.693862
$\sigma^{4,3}\pi_+^{1,1}\pi_-^{1,1}\delta_-^{1,0}$	−199.370797	−199.532944	−199.623833	−199.655967	−199.652827	−199.643473	−199.635164
$\sigma^{3,3}\pi_+^{1,1}\pi_-^{2,1}\delta_-^{1,0}$	−199.238483	−199.442539	−199.562107	−199.617983	−199.640035	−199.656244	−199.671729

Table S373: Total energies in E_h for the Al atom in the cc-pVDZ basis set in fully uncontracted form, employing the real-orbital approximation.

	$0.00B_0$	$0.10B_0$	$0.20B_0$	$0.30B_0$	$0.40B_0$	$0.50B_0$	$0.60B_0$
$\sigma^{5,4}\pi_+^{1,1}\pi_-^{1,1}$	−241.873568	−241.900932	−241.885319	−241.832835	−241.751273	−241.647531	−241.525945
$\sigma^{4,4}\pi_+^{2,1}\pi_-^{1,1}$	−241.871044	−241.841754	−241.758532	−241.632825	−241.476469	−241.296679	−241.096136
$\sigma^{4,4}\pi_+^{1,1}\pi_-^{2,1}$	−241.871044	−241.941754	−241.958532	−241.932825	−241.876469	−241.796679	−241.696136
$\sigma^{5,3}\pi_+^{1,1}\pi_-^{2,1}$	−241.783993	−241.957256	−242.081010	−242.164804	−242.218400	−242.247889	−242.255617
$\sigma^{4,3}\pi_+^{2,1}\pi_-^{2,1}$	−241.785283	−241.902312	−241.959091	−241.969166	−241.945588	−241.895040	−241.818867
$\sigma^{4,3}\pi_+^{1,1}\pi_-^{2,1}\delta_-^{1,0}$	−241.434059	−241.703283	−241.914302	−242.074883	−242.192289	−242.270396	−242.310410
$\sigma^{4,3}\pi_+^{1,1}\pi_-^{3,1}$	−241.462299	−241.670688	−241.827522	−241.954635	−242.045154	−242.102933	−242.129162

Table S374: Total energies in E_h for the Al atom in the cc-pVTZ basis set in fully uncontracted form, employing the real-orbital approximation.

	$0.00B_0$	$0.10B_0$	$0.20B_0$	$0.30B_0$	$0.40B_0$	$0.50B_0$	$0.60B_0$
$\sigma^{5,4}\pi_+^{1,1}\pi_-^{1,1}$	−241.879054	−241.906498	−241.891826	−241.842219	−241.765342	−241.666838	−241.550095
$\sigma^{4,4}\pi_+^{2,1}\pi_-^{1,1}$	−241.876117	−241.846748	−241.764434	−241.641919	−241.489701	−241.312565	−241.112408
$\sigma^{4,4}\pi_+^{1,1}\pi_-^{2,1}$	−241.876117	−241.946748	−241.964434	−241.941919	−241.889701	−241.812565	−241.712408
$\sigma^{5,3}\pi_+^{1,1}\pi_-^{2,1}$	−241.789209	−241.962692	−242.087771	−242.174750	−242.232887	−242.267479	−242.281110
$\sigma^{4,3}\pi_+^{2,1}\pi_-^{2,1}$	−241.790669	−241.907819	−241.965874	−241.979223	−241.959589	−241.912086	−241.838453
$\sigma^{4,3}\pi_+^{1,1}\pi_-^{2,1}\delta_-^{1,0}$	−241.535663	−241.799726	−241.996425	−242.136510	−242.232318	−242.295912	−242.337482
$\sigma^{4,3}\pi_+^{1,1}\pi_-^{3,1}$	−241.536671	−241.756137	−241.918476	−242.032522	−242.106979	−242.149023	−242.165303

Table S375: Total energies in E_h for the Al atom in the cc-pVQZ basis set in fully uncontracted form, employing the real-orbital approximation.

	$0.00B_0$	$0.10B_0$	$0.20B_0$	$0.30B_0$	$0.40B_0$	$0.50B_0$	$0.60B_0$
$\sigma^{5,4}\pi_+^{1,1}\pi_-^{1,1}$	−241.880444	−241.907953	−241.894297	−241.847261	−241.773436	−241.677309	−241.562231
$\sigma^{4,4}\pi_+^{2,1}\pi_-^{1,1}$	−241.877463	−241.848142	−241.767565	−241.647271	−241.494816	−241.315725	−241.114706
$\sigma^{4,4}\pi_+^{1,1}\pi_-^{2,1}$	−241.877463	−241.948142	−241.967565	−241.947271	−241.894816	−241.815725	−241.714706
$\sigma^{5,3}\pi_+^{1,1}\pi_-^{2,1}$	−241.791220	−241.965149	−242.092325	−242.182222	−242.242171	−242.277620	−242.292793
$\sigma^{4,3}\pi_+^{2,1}\pi_-^{2,1}$	−241.792716	−241.910447	−241.971067	−241.986800	−241.966336	−241.916361	−241.842420
$\sigma^{4,3}\pi_+^{1,1}\pi_-^{2,1}\delta_-^{1,0}$	−241.568019	−241.827452	−242.013090	−242.142921	−242.237144	−242.308039	−242.359306
$\sigma^{4,3}\pi_+^{1,1}\pi_-^{3,1}$	−241.570459	−241.785769	−241.940855	−242.045834	−242.114064	−242.157310	−242.182441

Table S376: Total energies in E_h for the Al atom in the cc-pV5Z basis set in fully uncontracted form, employing the real-orbital approximation.

	$0.00B_0$	$0.10B_0$	$0.20B_0$	$0.30B_0$	$0.40B_0$	$0.50B_0$	$0.60B_0$
$\sigma^{5,4}\pi_+^{1,1}\pi_-^{1,1}$	−241.880742	−241.908284	−241.894911	−241.848412	−241.775114	−241.679401	−241.564698
$\sigma^{4,4}\pi_+^{2,1}\pi_-^{1,1}$	−241.877742	−241.848454	−241.768068	−241.647825	−241.495440	−241.316808	−241.116702
$\sigma^{4,4}\pi_+^{1,1}\pi_-^{2,1}$	−241.877742	−241.948454	−241.968068	−241.947825	−241.895440	−241.816808	−241.716702
$\sigma^{5,3}\pi_+^{1,1}\pi_-^{2,1}$	−241.791520	−241.965500	−242.092926	−242.183190	−242.243630	−242.279741	−242.295639
$\sigma^{4,3}\pi_+^{2,1}\pi_-^{2,1}$	−241.793035	−241.910822	−241.971609	−241.987397	−241.967170	−241.917948	−241.845129
$\sigma^{4,3}\pi_+^{1,1}\pi_-^{2,1}\delta_-^{1,0}$	−241.565542	−241.825902	−242.014028	−242.146911	−242.243866	−242.317386	−242.371549
$\sigma^{4,3}\pi_+^{1,1}\pi_-^{3,1}$	−241.579933	−241.784226	−241.941325	−242.049258	−242.121001	−242.168138	−242.197572

Table S377: Total energies in E_h for the Al atom in the aug-cc-pVDZ basis set in fully uncontracted form, employing the real-orbital approximation.

	$0.00B_0$	$0.10B_0$	$0.20B_0$	$0.30B_0$	$0.40B_0$	$0.50B_0$	$0.60B_0$
$\sigma^{5,4}\pi_+^{1,1}\pi_-^{1,1}$	−241.874218	−241.901522	−241.886659	−241.835932	−241.755978	−241.652697	−241.530328
$\sigma^{4,4}\pi_+^{2,1}\pi_-^{1,1}$	−241.871448	−241.841922	−241.759656	−241.635892	−241.481026	−241.301465	−241.100032
$\sigma^{4,4}\pi_+^{1,1}\pi_-^{2,1}$	−241.871448	−241.941922	−241.959656	−241.935892	−241.881026	−241.801465	−241.700032
$\sigma^{5,3}\pi_+^{1,1}\pi_-^{2,1}$	−241.784117	−241.957567	−242.082254	−242.167188	−242.221283	−242.250444	−242.257365
$\sigma^{4,3}\pi_+^{2,1}\pi_-^{2,1}$	−241.785521	−241.902661	−241.960326	−241.971396	−241.948031	−241.896959	−241.820037
$\sigma^{4,3}\pi_+^{1,1}\pi_-^{2,1}\delta_-^{1,0}$	−241.579740	−241.831278	−241.998559	−242.115809	−242.208302	−242.275345	−242.311419
$\sigma^{4,3}\pi_+^{1,1}\pi_-^{3,1}$	−241.605483	−241.790944	−241.930927	−242.019185	−242.078397	−242.117658	−242.134446

Table S378: Total energies in E_h for the Al atom in the aug-cc-pVTZ basis set in fully uncontracted form, employing the real-orbital approximation.

	$0.00B_0$	$0.10B_0$	$0.20B_0$	$0.30B_0$	$0.40B_0$	$0.50B_0$	$0.60B_0$
$\sigma^{5,4}\pi_+^{1,1}\pi_-^{1,1}$	−241.879183	−241.906689	−241.893185	−241.846242	−241.772490	−241.676417	−241.560926
$\sigma^{4,4}\pi_+^{2,1}\pi_-^{1,1}$	−241.876250	−241.846806	−241.765442	−241.644206	−241.492498	−241.315193	−241.114698
$\sigma^{4,4}\pi_+^{1,1}\pi_-^{2,1}$	−241.876250	−241.946806	−241.965442	−241.944206	−241.892498	−241.815193	−241.714698
$\sigma^{5,3}\pi_+^{1,1}\pi_-^{2,1}$	−241.789220	−241.962923	−242.089455	−242.179162	−242.240174	−242.276756	−242.291164
$\sigma^{4,3}\pi_+^{2,1}\pi_-^{2,1}$	−241.790688	−241.908061	−241.967400	−241.982445	−241.963764	−241.916312	−241.842251
$\sigma^{4,3}\pi_+^{1,1}\pi_-^{2,1}\delta_-^{1,0}$	−241.597621	−241.840523	−242.008086	−242.138735	−242.234176	−242.299466	−242.341983
$\sigma^{4,3}\pi_+^{1,1}\pi_-^{3,1}$	−241.610971	−241.803344	−241.939849	−242.040074	−242.110592	−242.152513	−242.169614

Table S379: Total energies in E_h for the Al atom in the aug-cc-pVQZ basis set in fully uncontracted form, employing the real-orbital approximation.

	$0.00B_0$	$0.10B_0$	$0.20B_0$	$0.30B_0$	$0.40B_0$	$0.50B_0$	$0.60B_0$
$\sigma^{5,4}\pi_+^{1,1}\pi_-^{1,1}$	−241.880456	−241.908066	−241.895084	−241.848939	−241.775675	−241.679862	−241.565208
$\sigma^{4,4}\pi_+^{2,1}\pi_-^{1,1}$	−241.877472	−241.848213	−241.767941	−241.647779	−241.495393	−241.316635	−241.116446
$\sigma^{4,4}\pi_+^{1,1}\pi_-^{2,1}$	−241.877472	−241.948213	−241.967941	−241.947779	−241.895393	−241.816635	−241.716446
$\sigma^{5,3}\pi_+^{1,1}\pi_-^{2,1}$	−241.791224	−241.965250	−242.092945	−242.183492	−242.243819	−242.279376	−242.294626
$\sigma^{4,3}\pi_+^{2,1}\pi_-^{2,1}$	−241.792721	−241.910533	−241.971450	−241.987377	−241.966943	−241.916958	−241.843106
$\sigma^{4,3}\pi_+^{1,1}\pi_-^{2,1}\delta_-^{1,0}$	−241.602924	−241.842644	−242.014797	−242.145032	−242.241806	−242.314215	−242.366283
$\sigma^{4,3}\pi_+^{1,1}\pi_-^{3,1}$	−241.613579	−241.805847	−241.946269	−242.050484	−242.123766	−242.172831	−242.202467

Table S380: Total energies in E_h for the Al atom in the aug-cc-pV5Z basis set in fully uncontracted form, employing the real-orbital approximation.

	$0.00B_0$	$0.10B_0$	$0.20B_0$	$0.30B_0$	$0.40B_0$	$0.50B_0$	$0.60B_0$
$\sigma^{5,4}\pi_+^{1,1}\pi_-^{1,1}$	−241.880752	−241.908376	−241.895443	−241.849340	−241.776239	−241.680895	−241.567061
$\sigma^{4,4}\pi_+^{2,1}\pi_-^{1,1}$	−241.877747	−241.848511	−241.768288	−241.648058	−241.495784	−241.317652	−241.118484
$\sigma^{4,4}\pi_+^{1,1}\pi_-^{2,1}$	−241.877747	−241.948511	−241.968288	−241.948058	−241.895784	−241.817652	−241.718484
$\sigma^{5,3}\pi_+^{1,1}\pi_-^{2,1}$	−241.791521	−241.965565	−242.093275	−242.183789	−242.244370	−242.280744	−242.297278
$\sigma^{4,3}\pi_+^{2,1}\pi_-^{2,1}$	−241.793038	−241.910874	−241.971797	−241.987626	−241.967459	−241.918486	−241.846179
$\sigma^{4,3}\pi_+^{1,1}\pi_-^{2,1}\delta_-^{1,0}$	−241.603070	−241.843630	−242.016689	−242.149295	−242.248076	−242.321737	−242.375093
$\sigma^{4,3}\pi_+^{1,1}\pi_-^{3,1}$	−241.613965	−241.806863	−241.948501	−242.055381	−242.131477	−242.181943	−242.211700

Table S381: Total energies in E_h for the Al atom in the HGBSP1-5 basis set in fully uncontracted form, employing the real-orbital approximation.

	$0.00B_0$	$0.10B_0$	$0.20B_0$	$0.30B_0$	$0.40B_0$	$0.50B_0$	$0.60B_0$
$\sigma^{5,4}\pi_+^{1,1}\pi_-^{1,1}$	−241.875767	−241.903214	−241.889109	−241.840102	−241.762086	−241.659862	−241.537250
$\sigma^{4,4}\pi_+^{2,1}\pi_-^{1,1}$	−241.872799	−241.843544	−241.762874	−241.641395	−241.487336	−241.306762	−241.103763
$\sigma^{4,4}\pi_+^{1,1}\pi_-^{2,1}$	−241.872799	−241.943544	−241.962874	−241.941395	−241.887336	−241.806762	−241.703763
$\sigma^{5,3}\pi_+^{1,1}\pi_-^{2,1}$	−241.786590	−241.960511	−242.087001	−242.174270	−242.229401	−242.257800	−242.263251
$\sigma^{4,3}\pi_+^{2,1}\pi_-^{2,1}$	−241.788092	−241.905876	−241.965982	−241.979711	−241.956451	−241.903017	−241.823765
$\sigma^{4,3}\pi_+^{1,1}\pi_-^{2,1}\delta_-^{1,0}$	−241.602279	−241.839344	−242.011398	−242.141320	−242.237674	−242.307747	−242.355996
$\sigma^{4,3}\pi_+^{1,1}\pi_-^{3,1}$	−241.602443	−241.800963	−241.939776	−242.038494	−242.105246	−242.145737	−242.164601

Table S382: Total energies in E_h for the Al atom in the HGBSP1-7 basis set in fully uncontracted form, employing the real-orbital approximation.

	$0.00B_0$	$0.10B_0$	$0.20B_0$	$0.30B_0$	$0.40B_0$	$0.50B_0$	$0.60B_0$
$\sigma^{5,4}\pi_+^{1,1}\pi_-^{1,1}$	−241.880707	−241.908160	−241.894066	−241.845167	−241.767537	−241.665872	−241.543673
$\sigma^{4,4}\pi_+^{2,1}\pi_-^{1,1}$	−241.877739	−241.848492	−241.767848	−241.646632	−241.492956	−241.312612	−241.109839
$\sigma^{4,4}\pi_+^{1,1}\pi_-^{2,1}$	−241.877739	−241.948492	−241.967848	−241.946632	−241.892956	−241.812612	−241.709839
$\sigma^{5,3}\pi_+^{1,1}\pi_-^{2,1}$	−241.791544	−241.965473	−242.092029	−242.179568	−242.235043	−242.263623	−242.269167
$\sigma^{4,3}\pi_+^{2,1}\pi_-^{2,1}$	−241.793046	−241.910839	−241.971046	−241.985172	−241.962262	−241.908793	−241.829539
$\sigma^{4,3}\pi_+^{1,1}\pi_-^{2,1}\delta_-^{1,0}$	−241.607294	−241.844326	−242.016543	−242.146579	−242.243629	−242.313850	−242.361838
$\sigma^{4,3}\pi_+^{1,1}\pi_-^{3,1}$	−241.607458	−241.805977	−241.944943	−242.043723	−242.110806	−242.151788	−242.170821

Table S383: Total energies in E_h for the Al atom in the HGBSP1-9 basis set in fully uncontracted form, employing the real-orbital approximation.

	$0.00B_0$	$0.10B_0$	$0.20B_0$	$0.30B_0$	$0.40B_0$	$0.50B_0$	$0.60B_0$
$\sigma^{5,4}\pi_+^{1,1}\pi_-^{1,1}$	−241.880770	−241.908223	−241.894132	−241.845253	−241.767645	−241.665982	−241.543794
$\sigma^{4,4}\pi_+^{2,1}\pi_-^{1,1}$	−241.877802	−241.848556	−241.767918	−241.646715	−241.493085	−241.312799	−241.110025
$\sigma^{4,4}\pi_+^{1,1}\pi_-^{2,1}$	−241.877802	−241.948556	−241.967918	−241.946715	−241.893085	−241.812799	−241.710025
$\sigma^{5,3}\pi_+^{1,1}\pi_-^{2,1}$	−241.791607	−241.965536	−242.092098	−242.179646	−242.235147	−242.263770	−242.269337
$\sigma^{4,3}\pi_+^{2,1}\pi_-^{2,1}$	−241.793109	−241.910903	−241.971118	−241.985253	−241.962391	−241.909000	−241.829751
$\sigma^{4,3}\pi_+^{1,1}\pi_-^{2,1}\delta_-^{1,0}$	−241.607359	−241.844396	−242.016624	−242.146662	−242.243759	−242.314086	−242.362043
$\sigma^{4,3}\pi_+^{1,1}\pi_-^{3,1}$	−241.607523	−241.806042	−241.945009	−242.043824	−242.110904	−242.151952	−242.171047

Table S384: Total energies in E_h for the Al atom in the HGBSP2-5 basis set in fully uncontracted form, employing the real-orbital approximation.

	$0.00B_0$	$0.10B_0$	$0.20B_0$	$0.30B_0$	$0.40B_0$	$0.50B_0$	$0.60B_0$
$\sigma^{5,4}\pi_+^{1,1}\pi_-^{1,1}$	−241.875832	−241.903467	−241.890525	−241.844210	−241.770561	−241.674264	−241.558868
$\sigma^{4,4}\pi_+^{2,1}\pi_-^{1,1}$	−241.872813	−241.843597	−241.763349	−241.642911	−241.490520	−241.312152	−241.111913
$\sigma^{4,4}\pi_+^{1,1}\pi_-^{2,1}$	−241.872813	−241.943597	−241.963349	−241.942911	−241.890520	−241.812152	−241.711913
$\sigma^{5,3}\pi_+^{1,1}\pi_-^{2,1}$	−241.786603	−241.960652	−242.088306	−242.178747	−242.239443	−242.275764	−242.291342
$\sigma^{4,3}\pi_+^{2,1}\pi_-^{2,1}$	−241.788136	−241.905981	−241.966829	−241.982633	−241.962995	−241.914689	−241.842124
$\sigma^{4,3}\pi_+^{1,1}\pi_-^{2,1}\delta_-^{1,0}$	−241.602302	−241.839378	−242.011672	−242.142446	−242.240425	−242.312911	−242.364383
$\sigma^{4,3}\pi_+^{1,1}\pi_-^{3,1}$	−241.608971	−241.801886	−241.942397	−242.043731	−242.113975	−242.158814	−242.182875

Table S385: Total energies in E_h for the Al atom in the HGBSP2-7 basis set in fully uncontracted form, employing the real-orbital approximation.

	$0.00B_0$	$0.10B_0$	$0.20B_0$	$0.30B_0$	$0.40B_0$	$0.50B_0$	$0.60B_0$
$\sigma^{5,4}\pi_+^{1,1}\pi_-^{1,1}$	−241.880772	−241.908412	−241.895484	−241.849279	−241.775943	−241.680075	−241.565051
$\sigma^{4,4}\pi_+^{2,1}\pi_-^{1,1}$	−241.877753	−241.848544	−241.768323	−241.648122	−241.496086	−241.318001	−241.118058
$\sigma^{4,4}\pi_+^{1,1}\pi_-^{2,1}$	−241.877753	−241.948544	−241.968323	−241.948122	−241.896086	−241.818001	−241.718058
$\sigma^{5,3}\pi_+^{1,1}\pi_-^{2,1}$	−241.791556	−241.965613	−242.093336	−242.184013	−242.244962	−242.281414	−242.297144
$\sigma^{4,3}\pi_+^{2,1}\pi_-^{2,1}$	−241.793090	−241.910944	−241.971892	−241.988049	−241.968718	−241.920458	−241.847995
$\sigma^{4,3}\pi_+^{1,1}\pi_-^{2,1}\delta_-^{1,0}$	−241.607317	−241.844359	−242.016815	−242.147688	−242.246347	−242.319011	−242.370263
$\sigma^{4,3}\pi_+^{1,1}\pi_-^{3,1}$	−241.614027	−241.806896	−241.947555	−242.048930	−242.119512	−242.164880	−242.189132

Table S386: Total energies in E_h for the Al atom in the HGBSP2-9 basis set in fully uncontracted form, employing the real-orbital approximation.

	$0.00B_0$	$0.10B_0$	$0.20B_0$	$0.30B_0$	$0.40B_0$	$0.50B_0$	$0.60B_0$
$\sigma^{5,4}\pi_+^{1,1}\pi_-^{1,1}$	−241.880835	−241.908476	−241.895549	−241.849360	−241.776047	−241.680178	−241.565140
$\sigma^{4,4}\pi_+^{2,1}\pi_-^{1,1}$	−241.877816	−241.848608	−241.768392	−241.648206	−241.496210	−241.318161	−241.118213
$\sigma^{4,4}\pi_+^{1,1}\pi_-^{2,1}$	−241.877816	−241.948608	−241.968392	−241.948206	−241.896210	−241.818161	−241.718213
$\sigma^{5,3}\pi_+^{1,1}\pi_-^{2,1}$	−241.791619	−241.965677	−242.093404	−242.184089	−242.245059	−242.281533	−242.297263
$\sigma^{4,3}\pi_+^{2,1}\pi_-^{2,1}$	−241.793153	−241.911007	−241.971962	−241.988130	−241.968836	−241.920622	−241.848160
$\sigma^{4,3}\pi_+^{1,1}\pi_-^{2,1}\delta_-^{1,0}$	−241.607382	−241.844429	−242.016896	−242.147771	−242.246472	−242.319227	−242.370446
$\sigma^{4,3}\pi_+^{1,1}\pi_-^{3,1}$	−241.614305	−241.806961	−241.947621	−242.049029	−242.119603	−242.165025	−242.189337

Table S387: Total energies in E_h for the Al atom in the HGBSP3-5 basis set in fully uncontracted form, employing the real-orbital approximation.

	$0.00B_0$	$0.10B_0$	$0.20B_0$	$0.30B_0$	$0.40B_0$	$0.50B_0$	$0.60B_0$
$\sigma^{5,4}\pi_+^{1,1}\pi_-^{1,1}$	−241.875834	−241.903469	−241.890547	−241.844328	−241.770952	−241.675272	−241.561029
$\sigma^{4,4}\pi_+^{2,1}\pi_-^{1,1}$	−241.872813	−241.843598	−241.763357	−241.642971	−241.490779	−241.312953	−241.113846
$\sigma^{4,4}\pi_+^{1,1}\pi_-^{2,1}$	−241.872813	−241.943598	−241.963357	−241.942971	−241.890779	−241.812953	−241.713846
$\sigma^{5,3}\pi_+^{1,1}\pi_-^{2,1}$	−241.786603	−241.960652	−242.088311	−242.178783	−242.239592	−242.276200	−242.292356
$\sigma^{4,3}\pi_+^{2,1}\pi_-^{2,1}$	−241.788136	−241.905981	−241.966832	−241.982638	−241.963006	−241.914753	−241.842360
$\sigma^{4,3}\pi_+^{1,1}\pi_-^{2,1}\delta_-^{1,0}$	−241.602313	−241.839910	−242.013329	−242.145561	−242.245346	−242.319781	−242.373447
$\sigma^{4,3}\pi_+^{1,1}\pi_-^{3,1}$	−241.608971	−241.803476	−241.947369	−242.052888	−242.128174	−242.178674	−242.208641

Table S388: Total energies in E_h for the Al atom in the HGBSP3-7 basis set in fully uncontracted form, employing the real-orbital approximation.

	$0.00B_0$	$0.10B_0$	$0.20B_0$	$0.30B_0$	$0.40B_0$	$0.50B_0$	$0.60B_0$
$\sigma^{5,4}\pi_+^{1,1}\pi_-^{1,1}$	−241.880774	−241.908414	−241.895506	−241.849398	−241.776341	−241.681077	−241.567141
$\sigma^{4,4}\pi_+^{2,1}\pi_-^{1,1}$	−241.877753	−241.848545	−241.768332	−241.648184	−241.496352	−241.318794	−241.119905
$\sigma^{4,4}\pi_+^{1,1}\pi_-^{2,1}$	−241.877753	−241.948545	−241.968332	−241.948184	−241.896352	−241.818794	−241.719905
$\sigma^{5,3}\pi_+^{1,1}\pi_-^{2,1}$	−241.791556	−241.965613	−242.093341	−242.184050	−242.245111	−242.281840	−242.298118
$\sigma^{4,3}\pi_+^{2,1}\pi_-^{2,1}$	−241.793090	−241.910945	−241.971895	−241.988055	−241.968731	−241.920517	−241.848201
$\sigma^{4,3}\pi_+^{1,1}\pi_-^{2,1}\delta_-^{1,0}$	−241.607328	−241.844894	−242.018466	−242.150792	−242.251176	−242.325827	−242.379318
$\sigma^{4,3}\pi_+^{1,1}\pi_-^{3,1}$	−241.614027	−241.808486	−241.952475	−242.058124	−242.133613	−242.184458	−242.214683

Table S389: Total energies in E_h for the Al atom in the HGBSP3-9 basis set in fully uncontracted form, employing the real-orbital approximation.

	$0.00B_0$	$0.10B_0$	$0.20B_0$	$0.30B_0$	$0.40B_0$	$0.50B_0$	$0.60B_0$
$\sigma^{5,4}\pi_+^{1,1}\pi_-^{1,1}$	−241.880837	−241.908478	−241.895571	−241.849480	−241.776446	−241.681177	−241.567218
$\sigma^{4,4}\pi_+^{2,1}\pi_-^{1,1}$	−241.877816	−241.848609	−241.768401	−241.648268	−241.496477	−241.318949	−241.120044
$\sigma^{4,4}\pi_+^{1,1}\pi_-^{2,1}$	−241.877816	−241.948609	−241.968401	−241.948268	−241.896477	−241.818949	−241.720044
$\sigma^{5,3}\pi_+^{1,1}\pi_-^{2,1}$	−241.791619	−241.965677	−242.093409	−242.184127	−242.245209	−242.281958	−242.298232
$\sigma^{4,3}\pi_+^{2,1}\pi_-^{2,1}$	−241.793153	−241.911008	−241.971966	−241.988135	−241.968849	−241.920679	−241.848364
$\sigma^{4,3}\pi_+^{1,1}\pi_-^{2,1}\delta_-^{1,0}$	−241.607393	−241.844963	−242.018544	−242.150875	−242.251296	−242.326018	−242.379487
$\sigma^{4,3}\pi_+^{1,1}\pi_-^{3,1}$	−241.614305	−241.808552	−241.952543	−242.058209	−242.133709	−242.184581	−242.214818

Table S390: Total energies in E_h for the Al atom in the AHGBSP1-5 basis set in fully uncontracted form, employing the real-orbital approximation.

	$0.00B_0$	$0.10B_0$	$0.20B_0$	$0.30B_0$	$0.40B_0$	$0.50B_0$	$0.60B_0$
$\sigma^{5,4}\pi_+^{1,1}\pi_-^{1,1}$	−241.875772	−241.903221	−241.889113	−241.840118	−241.762163	−241.660018	−241.537461
$\sigma^{4,4}\pi_+^{2,1}\pi_-^{1,1}$	−241.872804	−241.843553	−241.762879	−241.641424	−241.487429	−241.306917	−241.103963
$\sigma^{4,4}\pi_+^{1,1}\pi_-^{2,1}$	−241.872804	−241.943553	−241.962879	−241.941424	−241.887429	−241.806917	−241.703963
$\sigma^{5,3}\pi_+^{1,1}\pi_-^{2,1}$	−241.786593	−241.960515	−242.087010	−242.174312	−242.229495	−242.257935	−242.263405
$\sigma^{4,3}\pi_+^{2,1}\pi_-^{2,1}$	−241.788095	−241.905879	−241.965994	−241.979772	−241.956571	−241.903169	−241.823922
$\sigma^{4,3}\pi_+^{1,1}\pi_-^{2,1}\delta_-^{1,0}$	−241.602297	−241.839354	−242.011421	−242.141364	−242.237773	−242.307881	−242.356136
$\sigma^{4,3}\pi_+^{1,1}\pi_-^{3,1}$	−241.602461	−241.800980	−241.939801	−242.038540	−242.105332	−242.145865	−242.164746

Table S391: Total energies in E_h for the Al atom in the AHGBSP1-7 basis set in fully uncontracted form, employing the real-orbital approximation.

	$0.00B_0$	$0.10B_0$	$0.20B_0$	$0.30B_0$	$0.40B_0$	$0.50B_0$	$0.60B_0$
$\sigma^{5,4}\pi_+^{1,1}\pi_-^{1,1}$	−241.880707	−241.908160	−241.894067	−241.845174	−241.767552	−241.665888	−241.543687
$\sigma^{4,4}\pi_+^{2,1}\pi_-^{1,1}$	−241.877739	−241.848493	−241.767850	−241.646639	−241.492973	−241.312636	−241.109859
$\sigma^{4,4}\pi_+^{1,1}\pi_-^{2,1}$	−241.877739	−241.948493	−241.967850	−241.946639	−241.892973	−241.812636	−241.709859
$\sigma^{5,3}\pi_+^{1,1}\pi_-^{2,1}$	−241.791544	−241.965473	−242.092031	−242.179574	−242.235053	−242.263636	−242.269179
$\sigma^{4,3}\pi_+^{2,1}\pi_-^{2,1}$	−241.793046	−241.910839	−241.971049	−241.985179	−241.962275	−241.908811	−241.829555
$\sigma^{4,3}\pi_+^{1,1}\pi_-^{2,1}\delta_-^{1,0}$	−241.607295	−241.844327	−242.016545	−242.146584	−242.243639	−242.313864	−242.361849
$\sigma^{4,3}\pi_+^{1,1}\pi_-^{3,1}$	−241.614183	−241.805977	−241.944944	−242.043728	−242.110815	−242.151799	−242.170831

Table S392: Total energies in E_h for the Al atom in the AHGBSP1-9 basis set in fully uncontracted form, employing the real-orbital approximation.

	$0.00B_0$	$0.10B_0$	$0.20B_0$	$0.30B_0$	$0.40B_0$	$0.50B_0$	$0.60B_0$
$\sigma^{5,4}\pi_+^{1,1}\pi_-^{1,1}$	−241.880770	−241.908223	−241.894132	−241.845253	−241.767646	−241.665982	−241.543795
$\sigma^{4,4}\pi_+^{2,1}\pi_-^{1,1}$	−241.877802	−241.848556	−241.767918	−241.646717	−241.493086	−241.312799	−241.110026
$\sigma^{4,4}\pi_+^{1,1}\pi_-^{2,1}$	−241.877802	−241.948556	−241.967918	−241.946717	−241.893086	−241.812799	−241.710026
$\sigma^{5,3}\pi_+^{1,1}\pi_-^{2,1}$	−241.791607	−241.965536	−242.092099	−242.179647	−242.235147	−242.263770	−242.269338
$\sigma^{4,3}\pi_+^{2,1}\pi_-^{2,1}$	−241.793109	−241.910903	−241.971118	−241.985253	−241.962391	−241.909000	−241.829753
$\sigma^{4,3}\pi_+^{1,1}\pi_-^{2,1}\delta_-^{1,0}$	−241.607359	−241.844396	−242.016624	−242.146662	−242.243759	−242.314086	−242.362045
$\sigma^{4,3}\pi_+^{1,1}\pi_-^{3,1}$	−241.607523	−241.806042	−241.945009	−242.043825	−242.110904	−242.151952	−242.171048

Table S393: Total energies in E_h for the Al atom in the AHGBSP2-5 basis set in fully uncontracted form, employing the real-orbital approximation.

	$0.00B_0$	$0.10B_0$	$0.20B_0$	$0.30B_0$	$0.40B_0$	$0.50B_0$	$0.60B_0$
$\sigma^{5,4}\pi_+^{1,1}\pi_-^{1,1}$	−241.875837	−241.903474	−241.890528	−241.844225	−241.770628	−241.674398	−241.559052
$\sigma^{4,4}\pi_+^{2,1}\pi_-^{1,1}$	−241.872818	−241.843606	−241.763353	−241.642936	−241.490606	−241.312305	−241.112112
$\sigma^{4,4}\pi_+^{1,1}\pi_-^{2,1}$	−241.872818	−241.943606	−241.963353	−241.942936	−241.890606	−241.812305	−241.712112
$\sigma^{5,3}\pi_+^{1,1}\pi_-^{2,1}$	−241.786606	−241.960655	−242.088315	−242.178783	−242.239519	−242.275872	−242.291468
$\sigma^{4,3}\pi_+^{2,1}\pi_-^{2,1}$	−241.788139	−241.905984	−241.966840	−241.982687	−241.963103	−241.914832	−241.842276
$\sigma^{4,3}\pi_+^{1,1}\pi_-^{2,1}\delta_-^{1,0}$	−241.602321	−241.839387	−242.011694	−242.142486	−242.240519	−242.313041	−242.364520
$\sigma^{4,3}\pi_+^{1,1}\pi_-^{3,1}$	−241.609207	−241.801902	−241.942421	−242.043772	−242.114056	−242.158939	−242.183017

Table S394: Total energies in E_h for the Al atom in the AHGBSP2-7 basis set in fully uncontracted form, employing the real-orbital approximation.

	$0.00B_0$	$0.10B_0$	$0.20B_0$	$0.30B_0$	$0.40B_0$	$0.50B_0$	$0.60B_0$
$\sigma^{5,4}\pi_+^{1,1}\pi_-^{1,1}$	−241.880773	−241.908413	−241.895484	−241.849284	−241.775956	−241.680088	−241.565060
$\sigma^{4,4}\pi_+^{2,1}\pi_-^{1,1}$	−241.877753	−241.848545	−241.768325	−241.648129	−241.496103	−241.318022	−241.118075
$\sigma^{4,4}\pi_+^{1,1}\pi_-^{2,1}$	−241.877753	−241.948545	−241.968325	−241.948129	−241.896103	−241.818022	−241.718075
$\sigma^{5,3}\pi_+^{1,1}\pi_-^{2,1}$	−241.791556	−241.965613	−242.093338	−242.184018	−242.244970	−242.281423	−242.297151
$\sigma^{4,3}\pi_+^{2,1}\pi_-^{2,1}$	−241.793090	−241.910944	−241.971894	−241.988056	−241.968730	−241.920472	−241.848006
$\sigma^{4,3}\pi_+^{1,1}\pi_-^{2,1}\delta_-^{1,0}$	−241.607318	−241.844360	−242.016817	−242.147693	−242.246356	−242.319023	−242.370271
$\sigma^{4,3}\pi_+^{1,1}\pi_-^{3,1}$	−241.614240	−241.806896	−241.947556	−242.048934	−242.119520	−242.164889	−242.189140

Table S395: Total energies in E_h for the Al atom in the AHGBSP2-9 basis set in fully uncontracted form, employing the real-orbital approximation.

	$0.00B_0$	$0.10B_0$	$0.20B_0$	$0.30B_0$	$0.40B_0$	$0.50B_0$	$0.60B_0$
$\sigma^{5,4}\pi_+^{1,1}\pi_-^{1,1}$	−241.880835	−241.908476	−241.895549	−241.849361	−241.776047	−241.680178	−241.565140
$\sigma^{4,4}\pi_+^{2,1}\pi_-^{1,1}$	−241.877816	−241.848608	−241.768392	−241.648207	−241.496211	−241.318161	−241.118213
$\sigma^{4,4}\pi_+^{1,1}\pi_-^{2,1}$	−241.877816	−241.948608	−241.968392	−241.948207	−241.896211	−241.818161	−241.718213
$\sigma^{5,3}\pi_+^{1,1}\pi_-^{2,1}$	−241.791619	−241.965677	−242.093404	−242.184090	−242.245059	−242.281533	−242.297263
$\sigma^{4,3}\pi_+^{2,1}\pi_-^{2,1}$	−241.793153	−241.911007	−241.971962	−241.988130	−241.968836	−241.920622	−241.848162
$\sigma^{4,3}\pi_+^{1,1}\pi_-^{2,1}\delta_-^{1,0}$	−241.607382	−241.844429	−242.016896	−242.147771	−242.246472	−242.319227	−242.370447
$\sigma^{4,3}\pi_+^{1,1}\pi_-^{3,1}$	−241.614305	−241.806961	−241.947622	−242.049029	−242.119603	−242.165025	−242.189338

Table S396: Total energies in E_h for the Al atom in the AHGBSP3-5 basis set in fully uncontracted form, employing the real-orbital approximation.

	$0.00B_0$	$0.10B_0$	$0.20B_0$	$0.30B_0$	$0.40B_0$	$0.50B_0$	$0.60B_0$
$\sigma^{5,4}\pi_+^{1,1}\pi_-^{1,1}$	−241.875839	−241.903476	−241.890550	−241.844344	−241.771022	−241.675402	−241.561189
$\sigma^{4,4}\pi_+^{2,1}\pi_-^{1,1}$	−241.872818	−241.843606	−241.763362	−241.642997	−241.490867	−241.313100	−241.114014
$\sigma^{4,4}\pi_+^{1,1}\pi_-^{2,1}$	−241.872818	−241.943606	−241.963362	−241.942997	−241.890867	−241.813100	−241.714014
$\sigma^{5,3}\pi_+^{1,1}\pi_-^{2,1}$	−241.786606	−241.960655	−242.088320	−242.178820	−242.239669	−242.276305	−242.292470
$\sigma^{4,3}\pi_+^{2,1}\pi_-^{2,1}$	−241.788139	−241.905984	−241.966844	−241.982692	−241.963116	−241.914895	−241.842503
$\sigma^{4,3}\pi_+^{1,1}\pi_-^{2,1}\delta_-^{1,0}$	−241.602332	−241.839920	−242.013350	−242.145602	−242.245437	−242.319905	−242.373573
$\sigma^{4,3}\pi_+^{1,1}\pi_-^{3,1}$	−241.602717	−241.803491	−241.947388	−242.052931	−242.128251	−242.178782	−242.208759

Table S397: Total energies in E_h for the Al atom in the AHGBSP3-7 basis set in fully uncontracted form, employing the real-orbital approximation.

	$0.00B_0$	$0.10B_0$	$0.20B_0$	$0.30B_0$	$0.40B_0$	$0.50B_0$	$0.60B_0$
$\sigma^{5,4}\pi_+^{1,1}\pi_-^{1,1}$	−241.880774	−241.908415	−241.895506	−241.849405	−241.776355	−241.681088	−241.567146
$\sigma^{4,4}\pi_+^{2,1}\pi_-^{1,1}$	−241.877753	−241.848546	−241.768333	−241.648192	−241.496369	−241.318811	−241.119916
$\sigma^{4,4}\pi_+^{1,1}\pi_-^{2,1}$	−241.877753	−241.948546	−241.968333	−241.948192	−241.896369	−241.818811	−241.719916
$\sigma^{5,3}\pi_+^{1,1}\pi_-^{2,1}$	−241.791556	−241.965613	−242.093343	−242.184055	−242.245119	−242.281849	−242.298123
$\sigma^{4,3}\pi_+^{2,1}\pi_-^{2,1}$	−241.793090	−241.910945	−241.971898	−241.988061	−241.968743	−241.920530	−241.848212
$\sigma^{4,3}\pi_+^{1,1}\pi_-^{2,1}\delta_-^{1,0}$	−241.607330	−241.844895	−242.018467	−242.150797	−242.251185	−242.325837	−242.379325
$\sigma^{4,3}\pi_+^{1,1}\pi_-^{3,1}$	−241.614240	−241.808487	−241.952476	−242.058128	−242.133620	−242.184466	−242.214688

Table S398: Total energies in E_h for the Al atom in the AHGBSP3-9 basis set in fully uncontracted form, employing the real-orbital approximation.

	$0.00B_0$	$0.10B_0$	$0.20B_0$	$0.30B_0$	$0.40B_0$	$0.50B_0$	$0.60B_0$
$\sigma^{5,4}\pi_+^{1,1}\pi_-^{1,1}$	−241.880837	−241.908478	−241.895571	−241.849481	−241.776447	−241.681177	−241.567220
$\sigma^{4,4}\pi_+^{2,1}\pi_-^{1,1}$	−241.877816	−241.848609	−241.768401	−241.648270	−241.496478	−241.318949	−241.120046
$\sigma^{4,4}\pi_+^{1,1}\pi_-^{2,1}$	−241.877816	−241.948609	−241.968401	−241.948270	−241.896478	−241.818949	−241.720046
$\sigma^{5,3}\pi_+^{1,1}\pi_-^{2,1}$	−241.791619	−241.965677	−242.093409	−242.184127	−242.245209	−242.281958	−242.298233
$\sigma^{4,3}\pi_+^{2,1}\pi_-^{2,1}$	−241.793153	−241.911008	−241.971966	−241.988135	−241.968849	−241.920680	−241.848366
$\sigma^{4,3}\pi_+^{1,1}\pi_-^{2,1}\delta_-^{1,0}$	−241.607393	−241.844963	−242.018544	−242.150876	−242.251296	−242.326018	−242.379488
$\sigma^{4,3}\pi_+^{1,1}\pi_-^{3,1}$	−241.607777	−241.808552	−241.952543	−242.058209	−242.133710	−242.184581	−242.214819

Table S399: Total energies in E_h for the Al atom in the 6-311++G(3df,3pd) basis set in fully uncontracted form, employing the real-orbital approximation.

	$0.00B_0$	$0.10B_0$	$0.20B_0$	$0.30B_0$	$0.40B_0$	$0.50B_0$	$0.60B_0$
$\sigma^{5,4}\pi_+^{1,1}\pi_-^{1,1}$	−241.875254	−241.902716	−241.888717	−241.840310	−241.763925	−241.664410	−241.545180
$\sigma^{4,4}\pi_+^{2,1}\pi_-^{1,1}$	−241.872353	−241.843086	−241.762369	−241.641187	−241.487594	−241.307160	−241.104116
$\sigma^{4,4}\pi_+^{1,1}\pi_-^{2,1}$	−241.872353	−241.943086	−241.962369	−241.941187	−241.887594	−241.807160	−241.704116
$\sigma^{5,3}\pi_+^{1,1}\pi_-^{2,1}$	−241.786080	−241.960006	−242.086718	−242.175008	−242.232310	−242.264317	−242.275545
$\sigma^{4,3}\pi_+^{2,1}\pi_-^{2,1}$	−241.787543	−241.905295	−241.965536	−241.980135	−241.958352	−241.906916	−241.831228
$\sigma^{4,3}\pi_+^{1,1}\pi_-^{2,1}\delta_-^{1,0}$	−241.563674	−241.823634	−242.008706	−242.131834	−242.212121	−242.271056	−242.320321
$\sigma^{4,3}\pi_+^{1,1}\pi_-^{3,1}$	−241.596668	−241.768835	−241.935874	−242.037256	−242.096224	−242.125721	−242.138389

Table S400: Total energies in E_h for the Al atom in the def2-TZVP basis set in fully uncontracted form, employing the real-orbital approximation.

	$0.00B_0$	$0.10B_0$	$0.20B_0$	$0.30B_0$	$0.40B_0$	$0.50B_0$	$0.60B_0$
$\sigma^{5,4}\pi_+^{1,1}\pi_-^{1,1}$	−241.875029	−241.902488	−241.887667	−241.837545	−241.760040	−241.661186	−241.544363
$\sigma^{4,4}\pi_+^{2,1}\pi_-^{1,1}$	−241.872059	−241.842705	−241.760022	−241.636542	−241.483712	−241.307165	−241.108573
$\sigma^{4,4}\pi_+^{1,1}\pi_-^{2,1}$	−241.872059	−241.942705	−241.960022	−241.936542	−241.883712	−241.807165	−241.708573
$\sigma^{5,3}\pi_+^{1,1}\pi_-^{2,1}$	−241.785141	−241.958506	−242.083050	−242.169109	−242.226627	−242.261534	−242.276447
$\sigma^{4,3}\pi_+^{2,1}\pi_-^{2,1}$	−241.786617	−241.903608	−241.960918	−241.973051	−241.952936	−241.906620	−241.835640
$\sigma^{4,3}\pi_+^{1,1}\pi_-^{2,1}\delta_-^{1,0}$	−241.533161	−241.797438	−241.994710	−242.135910	−242.233943	−242.301195	−242.347704
$\sigma^{4,3}\pi_+^{1,1}\pi_-^{3,1}$	−241.534170	−241.753731	−241.916284	−242.030850	−242.106691	−242.151276	−242.171052

Table S401: Total energies in E_h for the Si atom in the cc-pVDZ basis set in fully uncontracted form, employing the real-orbital approximation.

	$0.00B_0$	$0.10B_0$	$0.20B_0$	$0.30B_0$	$0.40B_0$	$0.50B_0$	$0.60B_0$
$\sigma^{4,4}\pi_+^{2,1}\pi_-^{2,1}$	−288.850220	−288.921077	−288.936011	−288.901499	−288.826319	−288.719072	−288.586195
$\sigma^{5,4}\pi_+^{2,1}\pi_-^{1,1}$	−288.847485	−288.872591	−288.849609	−288.783228	−288.679952	−288.546502	−288.388491
$\sigma^{5,4}\pi_+^{1,1}\pi_-^{2,1}$	−288.847485	−288.972591	−289.049609	−289.083228	−289.079952	−289.046502	−288.988491
$\sigma^{5,3}\pi_+^{2,1}\pi_-^{2,1}$	−288.754371	−288.927345	−289.048335	−289.122957	−289.158715	−289.162888	−289.140927
$\sigma^{5,3}\pi_+^{1,1}\pi_-^{2,1}\delta_-^{1,0}$	−288.260747	−288.585145	−288.859664	−289.087885	−289.274590	−289.424452	−289.541092
$\sigma^{5,3}\pi_+^{1,1}\pi_-^{3,1}$	−288.281923	−288.558294	−288.789854	−288.979762	−289.132726	−289.253331	−289.345116
$\sigma^{4,3}\pi_+^{1,1}\pi_-^{3,1}\delta_-^{1,0}$	−287.786712	−288.150412	−288.462820	−288.737272	−288.967314	−289.156459	−289.307216
$\sigma^{4,3}\pi_+^{1,1}\pi_-^{2,1}\delta_-^{1,0}\phi_-^{1,0}$							

Table S402: Total energies in E_h for the Si atom in the cc-pVTZ basis set in fully uncontracted form, employing the real-orbital approximation.

	$0.00B_0$	$0.10B_0$	$0.20B_0$	$0.30B_0$	$0.40B_0$	$0.50B_0$	$0.60B_0$
$\sigma^{4,4}\pi_+^{2,1}\pi_-^{2,1}$	−288.856476	−288.927189	−288.942437	−288.910194	−288.839908	−288.739145	−288.612390
$\sigma^{5,4}\pi_+^{2,1}\pi_-^{1,1}$	−288.853342	−288.878380	−288.855766	−288.791410	−288.692582	−288.565663	−288.415136
$\sigma^{5,4}\pi_+^{1,1}\pi_-^{2,1}$	−288.853342	−288.978380	−289.055766	−289.091410	−289.092582	−289.065663	−289.015136
$\sigma^{5,3}\pi_+^{2,1}\pi_-^{2,1}$	−288.760495	−288.933590	−289.055456	−289.132712	−289.173294	−289.183889	−289.168942
$\sigma^{5,3}\pi_+^{1,1}\pi_-^{2,1}\delta_-^{1,0}$	−288.395477	−288.716768	−288.982491	−289.197577	−289.368604	−289.502540	−289.606168
$\sigma^{5,3}\pi_+^{1,1}\pi_-^{3,1}$	−288.415416	−288.690240	−288.916377	−289.098165	−289.241103	−289.350515	−289.431034
$\sigma^{4,3}\pi_+^{1,1}\pi_-^{3,1}\delta_-^{1,0}$	−288.025540	−288.394166	−288.701710	−288.952660	−289.153180	−289.310163	−289.430819
$\sigma^{4,3}\pi_+^{1,1}\pi_-^{2,1}\delta_-^{1,0}\phi_-^{1,0}$	−287.233464	−287.701201	−288.106089	−288.452517	−288.746176	−288.992932	−289.198452

Table S403: Total energies in E_h for the Si atom in the cc-pVQZ basis set in fully uncontracted form, employing the real-orbital approximation.

	$0.00B_0$	$0.10B_0$	$0.20B_0$	$0.30B_0$	$0.40B_0$	$0.50B_0$	$0.60B_0$
$\sigma^{4,4}\pi_+^{2,1}\pi_-^{2,1}$	−288.858481	−288.929134	−288.945328	−288.915864	−288.848478	−288.748646	−288.620650
$\sigma^{5,4}\pi_+^{2,1}\pi_-^{1,1}$	−288.855300	−288.880288	−288.858385	−288.796405	−288.700913	−288.576914	−288.428233
$\sigma^{5,4}\pi_+^{1,1}\pi_-^{2,1}$	−288.855300	−288.980288	−289.058385	−289.096405	−289.100913	−289.076914	−289.028233
$\sigma^{5,3}\pi_+^{2,1}\pi_-^{2,1}$	−288.762875	−288.936177	−289.059338	−289.139422	−289.183209	−289.195990	−289.182031
$\sigma^{5,3}\pi_+^{1,1}\pi_-^{2,1}\delta_-^{1,0}$	−288.438715	−288.757166	−289.015390	−289.220874	−289.383685	−289.514422	−289.621633
$\sigma^{5,3}\pi_+^{1,1}\pi_-^{3,1}$	−288.454941	−288.728123	−288.949899	−289.125756	−289.262461	−289.367034	−289.446100
$\sigma^{4,3}\pi_+^{1,1}\pi_-^{3,1}\delta_-^{1,0}$	−288.100928	−288.465252	−288.761069	−288.996122	−289.181409	−289.329097	−289.449871
$\sigma^{4,3}\pi_+^{1,1}\pi_-^{2,1}\delta_-^{1,0}\phi_-^{1,0}$	−287.613047	−288.074089	−288.459691	−288.776447	−289.033459	−289.240491	−289.405898

Table S404: Total energies in E_h for the Si atom in the cc-pV5Z basis set in fully uncontracted form, employing the real-orbital approximation.

	$0.00B_0$	$0.10B_0$	$0.20B_0$	$0.30B_0$	$0.40B_0$	$0.50B_0$	$0.60B_0$
$\sigma^{4,4}\pi_+^{2,1}\pi_-^{2,1}$	−288.858819	−288.929484	−288.945871	−288.916727	−288.849544	−288.749874	−288.622259
$\sigma^{5,4}\pi_+^{2,1}\pi_-^{1,1}$	−288.855612	−288.880614	−288.858925	−288.797441	−288.702557	−288.579165	−288.431101
$\sigma^{5,4}\pi_+^{1,1}\pi_-^{2,1}$	−288.855612	−288.980614	−289.058925	−289.097441	−289.102557	−289.079165	−289.031101
$\sigma^{5,3}\pi_+^{2,1}\pi_-^{2,1}$	−288.763193	−288.936523	−289.059892	−289.140394	−289.184688	−289.198050	−289.184787
$\sigma^{5,3}\pi_+^{1,1}\pi_-^{2,1}\delta_-^{1,0}$	−288.435265	−288.754435	−289.014792	−289.223577	−289.390297	−289.524909	−289.635620
$\sigma^{5,3}\pi_+^{1,1}\pi_-^{3,1}$	−288.454600	−288.726271	−288.949453	−289.127688	−289.267622	−289.376049	−289.459312
$\sigma^{4,3}\pi_+^{1,1}\pi_-^{3,1}\delta_-^{1,0}$	−288.098295	−288.462181	−288.761472	−289.001899	−289.193795	−289.348588	−289.476446
$\sigma^{4,3}\pi_+^{1,1}\pi_-^{2,1}\delta_-^{1,0}\phi_-^{1,0}$	−287.746671	−288.204400	−288.580173	−288.881048	−289.117253	−289.300803	−289.443931

Table S405: Total energies in E_h for the Si atom in the aug-cc-pVDZ basis set in fully uncontracted form, employing the real-orbital approximation.

	$0.00B_0$	$0.10B_0$	$0.20B_0$	$0.30B_0$	$0.40B_0$	$0.50B_0$	$0.60B_0$
$\sigma^{4,4}\pi_+^{2,1}\pi_-^{2,1}$	−288.851086	−288.921574	−288.936837	−288.904248	−288.831838	−288.727144	−288.595852
$\sigma^{5,4}\pi_+^{2,1}\pi_-^{1,1}$	−288.848143	−288.872966	−288.850234	−288.785495	−288.684802	−288.553982	−288.397868
$\sigma^{5,4}\pi_+^{1,1}\pi_-^{2,1}$	−288.848143	−288.972966	−289.050234	−289.085495	−289.084802	−289.053982	−288.997868
$\sigma^{5,3}\pi_+^{2,1}\pi_-^{2,1}$	−288.754505	−288.927515	−289.049189	−289.125342	−289.162803	−289.168138	−289.146479
$\sigma^{5,3}\pi_+^{1,1}\pi_-^{2,1}\delta_-^{1,0}$	−288.452283	−288.765710	−289.010064	−289.197236	−289.344243	−289.464607	−289.562621
$\sigma^{5,3}\pi_+^{1,1}\pi_-^{3,1}$	−288.496185	−288.731629	−288.944244	−289.106256	−289.227549	−289.318603	−289.386852
$\sigma^{4,3}\pi_+^{1,1}\pi_-^{3,1}\delta_-^{1,0}$	−288.139896	−288.475158	−288.751234	−288.960474	−289.122492	−289.254102	−289.363239
$\sigma^{4,3}\pi_+^{1,1}\pi_-^{2,1}\delta_-^{1,0}\phi_-^{1,0}$							

Table S406: Total energies in E_h for the Si atom in the aug-cc-pVTZ basis set in fully uncontracted form, employing the real-orbital approximation.

	$0.00B_0$	$0.10B_0$	$0.20B_0$	$0.30B_0$	$0.40B_0$	$0.50B_0$	$0.60B_0$
$\sigma^{4,4}\pi_+^{2,1}\pi_-^{2,1}$	−288.856669	−288.927226	−288.943114	−288.912703	−288.844464	−288.745075	−288.618800
$\sigma^{5,4}\pi_+^{2,1}\pi_-^{1,1}$	−288.853537	−288.878478	−288.856562	−288.794414	−288.698834	−288.575347	−288.427747
$\sigma^{5,4}\pi_+^{1,1}\pi_-^{2,1}$	−288.853537	−288.978478	−289.056562	−289.094414	−289.098834	−289.075347	−289.027747
$\sigma^{5,3}\pi_+^{2,1}\pi_-^{2,1}$	−288.760500	−288.933687	−289.056504	−289.136044	−289.179804	−289.193697	−289.181536
$\sigma^{5,3}\pi_+^{1,1}\pi_-^{2,1}\delta_-^{1,0}$	−288.480524	−288.787434	−289.022228	−289.213999	−289.375963	−289.509229	−289.615725
$\sigma^{5,3}\pi_+^{1,1}\pi_-^{3,1}$	−288.503910	−288.754248	−288.960612	−289.123052	−289.254449	−289.359483	−289.439844
$\sigma^{4,3}\pi_+^{1,1}\pi_-^{3,1}\delta_-^{1,0}$	−288.159855	−288.507151	−288.773860	−288.987706	−289.166984	−289.315573	−289.434879
$\sigma^{4,3}\pi_+^{1,1}\pi_-^{2,1}\delta_-^{1,0}\phi_-^{1,0}$	−287.855518	−288.301059	−288.649554	−288.922745	−289.133167	−289.289918	−289.406879

Table S407: Total energies in E_h for the Si atom in the aug-cc-pVQZ basis set in fully uncontracted form, employing the real-orbital approximation.

	$0.00B_0$	$0.10B_0$	$0.20B_0$	$0.30B_0$	$0.40B_0$	$0.50B_0$	$0.60B_0$
$\sigma^{4,4}\pi_+^{2,1}\pi_-^{2,1}$	−288.858496	−288.929169	−288.945716	−288.916709	−288.849568	−288.749834	−288.622010
$\sigma^{5,4}\pi_+^{2,1}\pi_-^{1,1}$	−288.855313	−288.880340	−288.858915	−288.797827	−288.703209	−288.579802	−288.431451
$\sigma^{5,4}\pi_+^{1,1}\pi_-^{2,1}$	−288.855313	−288.980340	−289.058915	−289.097827	−289.103209	−289.079802	−289.031451
$\sigma^{5,3}\pi_+^{2,1}\pi_-^{2,1}$	−288.762878	−288.936234	−289.059785	−289.140567	−289.185060	−289.198332	−289.184597
$\sigma^{5,3}\pi_+^{1,1}\pi_-^{2,1}\delta_-^{1,0}$	−288.490281	−288.792797	−289.027038	−289.223413	−289.386172	−289.519480	−289.628925
$\sigma^{5,3}\pi_+^{1,1}\pi_-^{3,1}$	−288.509335	−288.759938	−288.965173	−289.131700	−289.267141	−289.374939	−289.458955
$\sigma^{4,3}\pi_+^{1,1}\pi_-^{3,1}\delta_-^{1,0}$	−288.167061	−288.514750	−288.782897	−289.004207	−289.188904	−289.342216	−289.470161
$\sigma^{4,3}\pi_+^{1,1}\pi_-^{2,1}\delta_-^{1,0}\phi_-^{1,0}$	−287.961517	−288.397010	−288.721365	−288.960368	−289.140004	−289.291278	−289.427595

Table S408: Total energies in E_h for the Si atom in the aug-cc-pV5Z basis set in fully uncontracted form, employing the real-orbital approximation.

	$0.00B_0$	$0.10B_0$	$0.20B_0$	$0.30B_0$	$0.40B_0$	$0.50B_0$	$0.60B_0$
$\sigma^{4,4}\pi_+^{2,1}\pi_-^{2,1}$	−288.858829	−288.929516	−288.946123	−288.917148	−288.849962	−288.750324	−288.623004
$\sigma^{5,4}\pi_+^{2,1}\pi_-^{1,1}$	−288.855618	−288.880656	−288.859279	−288.798227	−288.703599	−288.580298	−288.432392
$\sigma^{5,4}\pi_+^{1,1}\pi_-^{2,1}$	−288.855618	−288.980656	−289.059279	−289.098227	−289.103599	−289.080298	−289.032392
$\sigma^{5,3}\pi_+^{2,1}\pi_-^{2,1}$	−288.763194	−288.936560	−289.060144	−289.140924	−289.185392	−289.198845	−289.185735
$\sigma^{5,3}\pi_+^{1,1}\pi_-^{2,1}\delta_-^{1,0}$	−288.489684	−288.793839	−289.029551	−289.227215	−289.392486	−289.528596	−289.640523
$\sigma^{5,3}\pi_+^{1,1}\pi_-^{3,1}$	−288.510126	−288.761139	−288.967878	−289.135749	−289.273298	−289.383656	−289.469992
$\sigma^{4,3}\pi_+^{1,1}\pi_-^{3,1}\delta_-^{1,0}$	−288.169586	−288.518849	−288.789289	−289.013405	−289.202586	−289.360911	−289.493057
$\sigma^{4,3}\pi_+^{1,1}\pi_-^{2,1}\delta_-^{1,0}\phi_-^{1,0}$	−287.992973	−288.424057	−288.736114	−288.965288	−289.151088	−289.313839	−289.453349

Table S409: Total energies in E_h for the Si atom in the HGBSP1-5 basis set in fully uncontracted form, employing the real-orbital approximation.

	$0.00B_0$	$0.10B_0$	$0.20B_0$	$0.30B_0$	$0.40B_0$	$0.50B_0$	$0.60B_0$
$\sigma^{4,4}\pi_+^{2,1}\pi_-^{2,1}$	−288.852652	−288.923356	−288.939677	−288.909534	−288.840303	−288.738052	−288.607345
$\sigma^{5,4}\pi_+^{2,1}\pi_-^{1,1}$	−288.849480	−288.874442	−288.852395	−288.789334	−288.691087	−288.562658	−288.408070
$\sigma^{5,4}\pi_+^{1,1}\pi_-^{2,1}$	−288.849480	−288.974442	−289.052395	−289.089334	−289.091087	−289.062658	−289.008070
$\sigma^{5,3}\pi_+^{2,1}\pi_-^{2,1}$	−288.757083	−288.930393	−289.053292	−289.131888	−289.172353	−289.179971	−289.158856
$\sigma^{5,3}\pi_+^{1,1}\pi_-^{2,1}\delta_-^{1,0}$	−288.494712	−288.788433	−289.024130	−289.219434	−289.379957	−289.510782	−289.616194
$\sigma^{5,3}\pi_+^{1,1}\pi_-^{3,1}$	−288.496913	−288.755070	−288.959980	−289.124859	−289.255713	−289.357032	−289.432823
$\sigma^{4,3}\pi_+^{1,1}\pi_-^{3,1}\delta_-^{1,0}$	−288.167870	−288.512919	−288.782156	−289.001787	−289.182758	−289.332030	−289.455076
$\sigma^{4,3}\pi_+^{1,1}\pi_-^{2,1}\delta_-^{1,0}\phi_-^{1,0}$							

Table S410: Total energies in E_h for the Si atom in the HGBSP1-7 basis set in fully uncontracted form, employing the real-orbital approximation.

	$0.00B_0$	$0.10B_0$	$0.20B_0$	$0.30B_0$	$0.40B_0$	$0.50B_0$	$0.60B_0$
$\sigma^{4,4}\pi_+^{2,1}\pi_-^{2,1}$	−288.858778	−288.929488	−288.945852	−288.915913	−288.846982	−288.744878	−288.614206
$\sigma^{5,4}\pi_+^{2,1}\pi_-^{1,1}$	−288.855606	−288.880572	−288.858556	−288.795637	−288.697659	−288.569492	−288.415090
$\sigma^{5,4}\pi_+^{1,1}\pi_-^{2,1}$	−288.855606	−288.980572	−289.058556	−289.095637	−289.097659	−289.069492	−289.015090
$\sigma^{5,3}\pi_+^{2,1}\pi_-^{2,1}$	−288.763219	−288.936539	−289.059497	−289.138275	−289.178970	−289.186691	−289.165591
$\sigma^{5,3}\pi_+^{1,1}\pi_-^{2,1}\delta_-^{1,0}$	−288.500901	−288.794605	−289.030422	−289.225697	−289.386605	−289.517784	−289.623141
$\sigma^{5,3}\pi_+^{1,1}\pi_-^{3,1}$	−288.503090	−288.761244	−288.966211	−289.131164	−289.262145	−289.363803	−289.439839
$\sigma^{4,3}\pi_+^{1,1}\pi_-^{3,1}\delta_-^{1,0}$	−288.174023	−288.519065	−288.788412	−289.008035	−289.189299	−289.339058	−289.462255
$\sigma^{4,3}\pi_+^{1,1}\pi_-^{2,1}\delta_-^{1,0}\phi_-^{1,0}$							

Table S411: Total energies in E_h for the Si atom in the HGBSP1-9 basis set in fully uncontracted form, employing the real-orbital approximation.

	$0.00B_0$	$0.10B_0$	$0.20B_0$	$0.30B_0$	$0.40B_0$	$0.50B_0$	$0.60B_0$
$\sigma^{4,4}\pi_+^{2,1}\pi_-^{2,1}$	−288.858855	−288.929566	−288.945935	−288.916003	−288.847096	−288.745049	−288.614419
$\sigma^{5,4}\pi_+^{2,1}\pi_-^{1,1}$	−288.855684	−288.880650	−288.858637	−288.795727	−288.697767	−288.569631	−288.415253
$\sigma^{5,4}\pi_+^{1,1}\pi_-^{2,1}$	−288.855684	−288.980650	−289.058637	−289.095727	−289.097767	−289.069631	−289.015253
$\sigma^{5,3}\pi_+^{2,1}\pi_-^{2,1}$	−288.763296	−288.936616	−289.059578	−289.138359	−289.179070	−289.186830	−289.165766
$\sigma^{5,3}\pi_+^{1,1}\pi_-^{2,1}\delta_-^{1,0}$	−288.500980	−288.794686	−289.030504	−289.225796	−289.386697	−289.517943	−289.623346
$\sigma^{5,3}\pi_+^{1,1}\pi_-^{3,1}$	−288.503169	−288.761322	−288.966290	−289.131259	−289.262241	−289.363916	−289.440010
$\sigma^{4,3}\pi_+^{1,1}\pi_-^{3,1}\delta_-^{1,0}$	−288.174102	−288.519145	−288.788494	−289.008138	−289.189398	−289.339210	−289.462478
$\sigma^{4,3}\pi_+^{1,1}\pi_-^{2,1}\delta_-^{1,0}\phi_-^{1,0}$							

Table S412: Total energies in E_h for the Si atom in the HGBSP2-5 basis set in fully uncontracted form, employing the real-orbital approximation.

	$0.00B_0$	$0.10B_0$	$0.20B_0$	$0.30B_0$	$0.40B_0$	$0.50B_0$	$0.60B_0$
$\sigma^{4,4}\pi_+^{2,1}\pi_-^{2,1}$	−288.852732	−288.923430	−288.940027	−288.910827	−288.843377	−288.743754	−288.616518
$\sigma^{5,4}\pi_+^{2,1}\pi_-^{1,1}$	−288.849503	−288.874549	−288.853174	−288.791971	−288.697091	−288.573616	−288.425506
$\sigma^{5,4}\pi_+^{1,1}\pi_-^{2,1}$	−288.849503	−288.974549	−289.053174	−289.091971	−289.097091	−289.073616	−289.025506
$\sigma^{5,3}\pi_+^{2,1}\pi_-^{2,1}$	−288.757083	−288.930449	−289.053992	−289.134618	−289.179044	−289.192788	−289.180028
$\sigma^{5,3}\pi_+^{1,1}\pi_-^{2,1}\delta_-^{1,0}$	−288.494754	−288.788561	−289.024747	−289.221570	−289.385145	−289.520810	−289.632910
$\sigma^{5,3}\pi_+^{1,1}\pi_-^{3,1}$	−288.497349	−288.756178	−288.962125	−289.128783	−289.262669	−289.368591	−289.450687
$\sigma^{4,3}\pi_+^{1,1}\pi_-^{3,1}\delta_-^{1,0}$	−288.168378	−288.513685	−288.783725	−289.004748	−289.187742	−289.339700	−289.466106
$\sigma^{4,3}\pi_+^{1,1}\pi_-^{2,1}\delta_-^{1,0}\phi_-^{1,0}$	−288.112616	−288.467770	−288.737132	−288.958342	−289.146686	−289.305971	−289.438262

Table S413: Total energies in E_h for the Si atom in the HGBSP2-7 basis set in fully uncontracted form, employing the real-orbital approximation.

	$0.00B_0$	$0.10B_0$	$0.20B_0$	$0.30B_0$	$0.40B_0$	$0.50B_0$	$0.60B_0$
$\sigma^{4,4}\pi_+^{2,1}\pi_-^{2,1}$	−288.858858	−288.929561	−288.946203	−288.917192	−288.850009	−288.750546	−288.623417
$\sigma^{5,4}\pi_+^{2,1}\pi_-^{1,1}$	−288.855629	−288.880679	−288.859335	−288.798266	−288.703614	−288.580351	−288.432421
$\sigma^{5,4}\pi_+^{1,1}\pi_-^{2,1}$	−288.855629	−288.980679	−289.059335	−289.098266	−289.103614	−289.080351	−289.032421
$\sigma^{5,3}\pi_+^{2,1}\pi_-^{2,1}$	−288.763219	−288.936594	−289.060196	−289.140989	−289.185601	−289.199421	−289.186714
$\sigma^{5,3}\pi_+^{1,1}\pi_-^{2,1}\delta_-^{1,0}$	−288.500943	−288.794732	−289.031038	−289.227826	−289.391759	−289.527743	−289.639785
$\sigma^{5,3}\pi_+^{1,1}\pi_-^{3,1}$	−288.503525	−288.762348	−288.968354	−289.135075	−289.269076	−289.375316	−289.457654
$\sigma^{4,3}\pi_+^{1,1}\pi_-^{3,1}\delta_-^{1,0}$	−288.174531	−288.519831	−288.789980	−289.010987	−289.194275	−289.346729	−289.473296
$\sigma^{4,3}\pi_+^{1,1}\pi_-^{2,1}\delta_-^{1,0}\phi_-^{1,0}$	−288.118795	−288.474116	−288.743277	−288.966187	−289.153826	−289.312778	−289.446845

Table S414: Total energies in E_h for the Si atom in the HGBSP2-9 basis set in fully uncontracted form, employing the real-orbital approximation.

	$0.00B_0$	$0.10B_0$	$0.20B_0$	$0.30B_0$	$0.40B_0$	$0.50B_0$	$0.60B_0$
$\sigma^{4,4}\pi_+^{2,1}\pi_-^{2,1}$	−288.858936	−288.929639	−288.946285	−288.917282	−288.850122	−288.750701	−288.623595
$\sigma^{5,4}\pi_+^{2,1}\pi_-^{1,1}$	−288.855707	−288.880757	−288.859416	−288.798355	−288.703721	−288.580480	−288.432557
$\sigma^{5,4}\pi_+^{1,1}\pi_-^{2,1}$	−288.855707	−288.980757	−289.059416	−289.098355	−289.103721	−289.080480	−289.032557
$\sigma^{5,3}\pi_+^{2,1}\pi_-^{2,1}$	−288.763296	−288.936672	−289.060277	−289.141073	−289.185698	−289.199545	−289.186854
$\sigma^{5,3}\pi_+^{1,1}\pi_-^{2,1}\delta_-^{1,0}$	−288.501021	−288.794813	−289.031120	−289.227924	−289.391851	−289.527895	−289.639967
$\sigma^{5,3}\pi_+^{1,1}\pi_-^{3,1}$	−288.503603	−288.762426	−288.968432	−289.135169	−289.269169	−289.375421	−289.457805
$\sigma^{4,3}\pi_+^{1,1}\pi_-^{3,1}\delta_-^{1,0}$	−288.174610	−288.519910	−288.790062	−289.011090	−289.194372	−289.346875	−289.473508
$\sigma^{4,3}\pi_+^{1,1}\pi_-^{2,1}\delta_-^{1,0}\phi_-^{1,0}$	−288.118874	−288.474227	−288.743434	−288.966373	−289.154078	−289.312907	−289.447240

Table S415: Total energies in E_h for the Si atom in the HGBSP3-5 basis set in fully uncontracted form, employing the real-orbital approximation.

	$0.00B_0$	$0.10B_0$	$0.20B_0$	$0.30B_0$	$0.40B_0$	$0.50B_0$	$0.60B_0$
$\sigma^{4,4}\pi_+^{2,1}\pi_-^{2,1}$	−288.852733	−288.923432	−288.940037	−288.910865	−288.843500	−288.744092	−288.617311
$\sigma^{5,4}\pi_+^{2,1}\pi_-^{1,1}$	−288.849503	−288.874548	−288.853175	−288.791985	−288.697156	−288.573823	−288.426037
$\sigma^{5,4}\pi_+^{1,1}\pi_-^{2,1}$	−288.849503	−288.974548	−289.053175	−289.091985	−289.097156	−289.073823	−289.026037
$\sigma^{5,3}\pi_+^{2,1}\pi_-^{2,1}$	−288.757083	−288.930449	−289.053992	−289.134620	−289.179061	−289.192851	−289.180207
$\sigma^{5,3}\pi_+^{1,1}\pi_-^{2,1}\delta_-^{1,0}$	−288.494765	−288.788800	−289.025551	−289.223133	−289.387732	−289.524607	−289.638081
$\sigma^{5,3}\pi_+^{1,1}\pi_-^{3,1}$	−288.497369	−288.756759	−288.964288	−289.133252	−289.270137	−289.379734	−289.465978
$\sigma^{4,3}\pi_+^{1,1}\pi_-^{3,1}\delta_-^{1,0}$	−288.168651	−288.514945	−288.787987	−289.013270	−289.201539	−289.359616	−289.492641
$\sigma^{4,3}\pi_+^{1,1}\pi_-^{2,1}\delta_-^{1,0}\phi_-^{1,0}$	−288.112638	−288.467920	−288.737779	−288.959838	−289.149334	−289.309967	−289.443782

Table S416: Total energies in E_h for the Si atom in the HGBSP3-7 basis set in fully uncontracted form, employing the real-orbital approximation.

	$0.00B_0$	$0.10B_0$	$0.20B_0$	$0.30B_0$	$0.40B_0$	$0.50B_0$	$0.60B_0$
$\sigma^{4,4}\pi_+^{2,1}\pi_-^{2,1}$	−288.858860	−288.929564	−288.946213	−288.917230	−288.850134	−288.750884	−288.624194
$\sigma^{5,4}\pi_+^{2,1}\pi_-^{1,1}$	−288.855629	−288.880679	−288.859337	−288.798281	−288.703681	−288.580561	−288.432940
$\sigma^{5,4}\pi_+^{1,1}\pi_-^{2,1}$	−288.855629	−288.980679	−289.059337	−289.098281	−289.103681	−289.080561	−289.032940
$\sigma^{5,3}\pi_+^{2,1}\pi_-^{2,1}$	−288.763219	−288.936594	−289.060197	−289.140992	−289.185618	−289.199484	−289.186886
$\sigma^{5,3}\pi_+^{1,1}\pi_-^{2,1}\delta_-^{1,0}$	−288.500953	−288.794972	−289.031830	−289.229394	−289.394304	−289.531482	−289.644934
$\sigma^{5,3}\pi_+^{1,1}\pi_-^{3,1}$	−288.503545	−288.762928	−288.970495	−289.139546	−289.276529	−289.386327	−289.472769
$\sigma^{4,3}\pi_+^{1,1}\pi_-^{3,1}\delta_-^{1,0}$	−288.174804	−288.521091	−288.794216	−289.019516	−289.208025	−289.366446	−289.499610
$\sigma^{4,3}\pi_+^{1,1}\pi_-^{2,1}\delta_-^{1,0}\phi_-^{1,0}$	−288.118818	−288.474267	−288.743924	−288.967683	−289.156442	−289.316742	−289.452362

Table S417: Total energies in E_h for the Si atom in the HGBSP3-9 basis set in fully uncontracted form, employing the real-orbital approximation.

	$0.00B_0$	$0.10B_0$	$0.20B_0$	$0.30B_0$	$0.40B_0$	$0.50B_0$	$0.60B_0$
$\sigma^{4,4}\pi_+^{2,1}\pi_-^{2,1}$	−288.858937	−288.929642	−288.946295	−288.917320	−288.850247	−288.751038	−288.624367
$\sigma^{5,4}\pi_+^{2,1}\pi_-^{1,1}$	−288.855707	−288.880757	−288.859418	−288.798370	−288.703788	−288.580690	−288.433073
$\sigma^{5,4}\pi_+^{1,1}\pi_-^{2,1}$	−288.855707	−288.980757	−289.059418	−289.098370	−289.103788	−289.080690	−289.033073
$\sigma^{5,3}\pi_+^{2,1}\pi_-^{2,1}$	−288.763296	−288.936672	−289.060277	−289.141076	−289.185716	−289.199607	−289.187026
$\sigma^{5,3}\pi_+^{1,1}\pi_-^{2,1}\delta_-^{1,0}$	−288.501032	−288.795053	−289.031911	−289.229490	−289.394397	−289.531624	−289.645101
$\sigma^{5,3}\pi_+^{1,1}\pi_-^{3,1}$	−288.503624	−288.763006	−288.970574	−289.139634	−289.276621	−289.386432	−289.472894
$\sigma^{4,3}\pi_+^{1,1}\pi_-^{3,1}\delta_-^{1,0}$	−288.174882	−288.521171	−288.794298	−289.019611	−289.208121	−289.366584	−289.499779
$\sigma^{4,3}\pi_+^{1,1}\pi_-^{2,1}\delta_-^{1,0}\phi_-^{1,0}$	−288.118896	−288.474377	−288.744079	−288.967869	−289.156693	−289.316864	−289.452748

Table S418: Total energies in E_h for the Si atom in the AHGBSP1-5 basis set in fully uncontracted form, employing the real-orbital approximation.

	$0.00B_0$	$0.10B_0$	$0.20B_0$	$0.30B_0$	$0.40B_0$	$0.50B_0$	$0.60B_0$
$\sigma^{4,4}\pi_+^{2,1}\pi_-^{2,1}$	−288.852655	−288.923360	−288.939685	−288.909571	−288.840393	−288.738189	−288.607510
$\sigma^{5,4}\pi_+^{2,1}\pi_-^{1,1}$	−288.849483	−288.874445	−288.852401	−288.789361	−288.691163	−288.562788	−288.408240
$\sigma^{5,4}\pi_+^{1,1}\pi_-^{2,1}$	−288.849483	−288.974445	−289.052401	−289.089361	−289.091163	−289.062788	−289.008240
$\sigma^{5,3}\pi_+^{2,1}\pi_-^{2,1}$	−288.757090	−288.930401	−289.053310	−289.131935	−289.172441	−289.180087	−289.158982
$\sigma^{5,3}\pi_+^{1,1}\pi_-^{2,1}\delta_-^{1,0}$	−288.494736	−288.788449	−289.024149	−289.219468	−289.380023	−289.510876	−289.616304
$\sigma^{5,3}\pi_+^{1,1}\pi_-^{3,1}$	−288.505922	−288.755086	−288.959997	−289.124893	−289.255774	−289.357121	−289.432928
$\sigma^{4,3}\pi_+^{1,1}\pi_-^{3,1}\delta_-^{1,0}$	−288.167886	−288.512931	−288.782172	−289.001816	−289.182811	−289.332109	−289.455170
$\sigma^{4,3}\pi_+^{1,1}\pi_-^{2,1}\delta_-^{1,0}\phi_-^{1,0}$							

Table S419: Total energies in E_h for the Si atom in the AHGBSP1-7 basis set in fully uncontracted form, employing the real-orbital approximation.

	$0.00B_0$	$0.10B_0$	$0.20B_0$	$0.30B_0$	$0.40B_0$	$0.50B_0$	$0.60B_0$
$\sigma^{4,4}\pi_+^{2,1}\pi_-^{2,1}$	−288.858778	−288.929489	−288.945854	−288.915919	−288.846996	−288.744900	−288.614230
$\sigma^{5,4}\pi_+^{2,1}\pi_-^{1,1}$	−288.855607	−288.880573	−288.858557	−288.795643	−288.697672	−288.569510	−288.415108
$\sigma^{5,4}\pi_+^{1,1}\pi_-^{2,1}$	−288.855607	−288.980573	−289.058557	−289.095643	−289.097672	−289.069510	−289.015108
$\sigma^{5,3}\pi_+^{2,1}\pi_-^{2,1}$	−288.763219	−288.936539	−289.059499	−289.138279	−289.178977	−289.186701	−289.165602
$\sigma^{5,3}\pi_+^{1,1}\pi_-^{2,1}\delta_-^{1,0}$	−288.500902	−288.794606	−289.030423	−289.225700	−289.386610	−289.517792	−289.623150
$\sigma^{5,3}\pi_+^{1,1}\pi_-^{3,1}$	−288.503091	−288.761244	−288.966212	−289.131167	−289.262150	−289.363810	−289.439847
$\sigma^{4,3}\pi_+^{1,1}\pi_-^{3,1}\delta_-^{1,0}$	−288.174024	−288.519066	−288.788413	−289.008038	−289.189303	−289.339065	−289.462263
$\sigma^{4,3}\pi_+^{1,1}\pi_-^{2,1}\delta_-^{1,0}\phi_-^{1,0}$							

Table S420: Total energies in E_h for the Si atom in the AHGBSP1-9 basis set in fully uncontracted form, employing the real-orbital approximation.

	$0.00B_0$	$0.10B_0$	$0.20B_0$	$0.30B_0$	$0.40B_0$	$0.50B_0$	$0.60B_0$
$\sigma^{4,4}\pi_+^{2,1}\pi_-^{2,1}$	−288.858856	−288.929566	−288.945935	−288.916004	−288.847097	−288.745049	−288.614420
$\sigma^{5,4}\pi_+^{2,1}\pi_-^{1,1}$	−288.855684	−288.880650	−288.858637	−288.795727	−288.697768	−288.569631	−288.415254
$\sigma^{5,4}\pi_+^{1,1}\pi_-^{2,1}$	−288.855684	−288.980650	−289.058637	−289.095727	−289.097768	−289.069631	−289.015254
$\sigma^{5,3}\pi_+^{2,1}\pi_-^{2,1}$	−288.763296	−288.936616	−289.059578	−289.138359	−289.179070	−289.186830	−289.165767
$\sigma^{5,3}\pi_+^{1,1}\pi_-^{2,1}\delta_-^{1,0}$	−288.500980	−288.794686	−289.030504	−289.225796	−289.386697	−289.517943	−289.623346
$\sigma^{5,3}\pi_+^{1,1}\pi_-^{3,1}$	−288.503169	−288.761322	−288.966290	−289.131259	−289.262241	−289.363916	−289.440010
$\sigma^{4,3}\pi_+^{1,1}\pi_-^{3,1}\delta_-^{1,0}$	−288.174102	−288.519145	−288.788494	−289.008138	−289.189398	−289.339211	−289.462479
$\sigma^{4,3}\pi_+^{1,1}\pi_-^{2,1}\delta_-^{1,0}\phi_-^{1,0}$							

Table S421: Total energies in E_h for the Si atom in the AHGBSP2-5 basis set in fully uncontracted form, employing the real-orbital approximation.

	$0.00B_0$	$0.10B_0$	$0.20B_0$	$0.30B_0$	$0.40B_0$	$0.50B_0$	$0.60B_0$
$\sigma^{4,4}\pi_+^{2,1}\pi_-^{2,1}$	−288.852735	−288.923433	−288.940035	−288.910861	−288.843459	−288.743884	−288.616680
$\sigma^{5,4}\pi_+^{2,1}\pi_-^{1,1}$	−288.849506	−288.874552	−288.853180	−288.791995	−288.697157	−288.573728	−288.425656
$\sigma^{5,4}\pi_+^{1,1}\pi_-^{2,1}$	−288.849506	−288.974552	−289.053180	−289.091995	−289.097157	−289.073728	−289.025656
$\sigma^{5,3}\pi_+^{2,1}\pi_-^{2,1}$	−288.757091	−288.930457	−289.054009	−289.134661	−289.179119	−289.192885	−289.180134
$\sigma^{5,3}\pi_+^{1,1}\pi_-^{2,1}\delta_-^{1,0}$	−288.494778	−288.788576	−289.024765	−289.221603	−289.385203	−289.520892	−289.633005
$\sigma^{5,3}\pi_+^{1,1}\pi_-^{3,1}$	−288.497369	−288.756193	−288.962142	−289.128814	−289.262723	−289.368668	−289.450780
$\sigma^{4,3}\pi_+^{1,1}\pi_-^{3,1}\delta_-^{1,0}$	−288.168395	−288.513698	−288.783741	−289.004776	−289.187793	−289.339777	−289.466199
$\sigma^{4,3}\pi_+^{1,1}\pi_-^{2,1}\delta_-^{1,0}\phi_-^{1,0}$	−288.112637	−288.467795	−288.737155	−288.958378	−289.146735	−289.306034	−289.438337

Table S422: Total energies in E_h for the Si atom in the AHGBSP2-7 basis set in fully uncontracted form, employing the real-orbital approximation.

	$0.00B_0$	$0.10B_0$	$0.20B_0$	$0.30B_0$	$0.40B_0$	$0.50B_0$	$0.60B_0$
$\sigma^{4,4}\pi_+^{2,1}\pi_-^{2,1}$	−288.858858	−288.929562	−288.946205	−288.917198	−288.850022	−288.750565	−288.623437
$\sigma^{5,4}\pi_+^{2,1}\pi_-^{1,1}$	−288.855629	−288.880680	−288.859337	−288.798272	−288.703626	−288.580367	−288.432435
$\sigma^{5,4}\pi_+^{1,1}\pi_-^{2,1}$	−288.855629	−288.980680	−289.059337	−289.098272	−289.103626	−289.080367	−289.032435
$\sigma^{5,3}\pi_+^{2,1}\pi_-^{2,1}$	−288.763219	−288.936595	−289.060198	−289.140993	−289.185607	−289.199429	−289.186721
$\sigma^{5,3}\pi_+^{1,1}\pi_-^{2,1}\delta_-^{1,0}$	−288.500943	−288.794733	−289.031039	−289.227829	−289.391764	−289.527750	−289.639791
$\sigma^{5,3}\pi_+^{1,1}\pi_-^{3,1}$	−288.503525	−288.762349	−288.968354	−289.135077	−289.269080	−289.375321	−289.457660
$\sigma^{4,3}\pi_+^{1,1}\pi_-^{3,1}\delta_-^{1,0}$	−288.174532	−288.519831	−288.789981	−289.010990	−289.194280	−289.346735	−289.473302
$\sigma^{4,3}\pi_+^{1,1}\pi_-^{2,1}\delta_-^{1,0}\phi_-^{1,0}$	−288.118795	−288.474119	−288.743280	−288.966190	−289.153831	−289.312782	−289.446850

Table S423: Total energies in E_h for the Si atom in the AHGBSP2-9 basis set in fully uncontracted form, employing the real-orbital approximation.

	$0.00B_0$	$0.10B_0$	$0.20B_0$	$0.30B_0$	$0.40B_0$	$0.50B_0$	$0.60B_0$
$\sigma^{4,4}\pi_+^{2,1}\pi_-^{2,1}$	−288.858936	−288.929639	−288.946285	−288.917282	−288.850123	−288.750701	−288.623595
$\sigma^{5,4}\pi_+^{2,1}\pi_-^{1,1}$	−288.855707	−288.880757	−288.859416	−288.798355	−288.703721	−288.580480	−288.432557
$\sigma^{5,4}\pi_+^{1,1}\pi_-^{2,1}$	−288.855707	−288.980757	−289.059416	−289.098355	−289.103721	−289.080480	−289.032557
$\sigma^{5,3}\pi_+^{2,1}\pi_-^{2,1}$	−288.763296	−288.936672	−289.060277	−289.141073	−289.185698	−289.199545	−289.186854
$\sigma^{5,3}\pi_+^{1,1}\pi_-^{2,1}\delta_-^{1,0}$	−288.501021	−288.794813	−289.031120	−289.227924	−289.391851	−289.527895	−289.639968
$\sigma^{5,3}\pi_+^{1,1}\pi_-^{3,1}$	−288.503603	−288.762426	−288.968432	−289.135169	−289.269170	−289.375421	−289.457805
$\sigma^{4,3}\pi_+^{1,1}\pi_-^{3,1}\delta_-^{1,0}$	−288.174610	−288.519910	−288.790062	−289.011090	−289.194372	−289.346875	−289.473508
$\sigma^{4,3}\pi_+^{1,1}\pi_-^{2,1}\delta_-^{1,0}\phi_-^{1,0}$	−288.118874	−288.474227	−288.743434	−288.966373	−289.154078	−289.312907	−289.447241

Table S424: Total energies in E_h for the Si atom in the AHGBSP3-5 basis set in fully uncontracted form, employing the real-orbital approximation.

	$0.00B_0$	$0.10B_0$	$0.20B_0$	$0.30B_0$	$0.40B_0$	$0.50B_0$	$0.60B_0$
$\sigma^{4,4}\pi_+^{2,1}\pi_-^{2,1}$	−288.852737	−288.923436	−288.940045	−288.910899	−288.843583	−288.744221	−288.617467
$\sigma^{5,4}\pi_+^{2,1}\pi_-^{1,1}$	−288.849506	−288.874552	−288.853182	−288.792010	−288.697222	−288.573937	−288.426182
$\sigma^{5,4}\pi_+^{1,1}\pi_-^{2,1}$	−288.849506	−288.974552	−289.053182	−289.092010	−289.097222	−289.073937	−289.026182
$\sigma^{5,3}\pi_+^{2,1}\pi_-^{2,1}$	−288.757090	−288.930457	−289.054009	−289.134663	−289.179136	−289.192949	−289.180312
$\sigma^{5,3}\pi_+^{1,1}\pi_-^{2,1}\delta_-^{1,0}$	−288.494788	−288.788815	−289.025569	−289.223166	−289.387790	−289.524688	−289.638172
$\sigma^{5,3}\pi_+^{1,1}\pi_-^{3,1}$	−288.497389	−288.756774	−288.964304	−289.133284	−289.270190	−289.379808	−289.466063
$\sigma^{4,3}\pi_+^{1,1}\pi_-^{3,1}\delta_-^{1,0}$	−288.168667	−288.514958	−288.788003	−289.013297	−289.201588	−289.359688	−289.492725
$\sigma^{4,3}\pi_+^{1,1}\pi_-^{2,1}\delta_-^{1,0}\phi_-^{1,0}$	−288.112660	−288.467944	−288.737802	−288.959874	−289.149382	−289.310029	−289.443854

Table S425: Total energies in E_h for the Si atom in the AHGBSP3-7 basis set in fully uncontracted form, employing the real-orbital approximation.

	$0.00B_0$	$0.10B_0$	$0.20B_0$	$0.30B_0$	$0.40B_0$	$0.50B_0$	$0.60B_0$
$\sigma^{4,4}\pi_+^{2,1}\pi_-^{2,1}$	−288.858860	−288.929565	−288.946215	−288.917237	−288.850147	−288.750903	−288.624211
$\sigma^{5,4}\pi_+^{2,1}\pi_-^{1,1}$	−288.855629	−288.880680	−288.859339	−288.798287	−288.703694	−288.580576	−288.432952
$\sigma^{5,4}\pi_+^{1,1}\pi_-^{2,1}$	−288.855629	−288.980680	−289.059339	−289.098287	−289.103694	−289.080576	−289.032952
$\sigma^{5,3}\pi_+^{2,1}\pi_-^{2,1}$	−288.763219	−288.936595	−289.060198	−289.140996	−289.185624	−289.199491	−289.186893
$\sigma^{5,3}\pi_+^{1,1}\pi_-^{2,1}\delta_-^{1,0}$	−288.500954	−288.794973	−289.031830	−289.229397	−289.394309	−289.531488	−289.644939
$\sigma^{5,3}\pi_+^{1,1}\pi_-^{3,1}$	−288.503546	−288.762928	−288.970496	−289.139548	−289.276533	−289.386332	−289.472773
$\sigma^{4,3}\pi_+^{1,1}\pi_-^{3,1}\delta_-^{1,0}$	−288.174804	−288.521092	−288.794217	−289.019518	−289.208029	−289.366451	−289.499615
$\sigma^{4,3}\pi_+^{1,1}\pi_-^{2,1}\delta_-^{1,0}\phi_-^{1,0}$	−288.118818	−288.474270	−288.743927	−288.967686	−289.156446	−289.316745	−289.452367

Table S426: Total energies in E_h for the Si atom in the AHGBSP3-9 basis set in fully uncontracted form, employing the real-orbital approximation.

	$0.00B_0$	$0.10B_0$	$0.20B_0$	$0.30B_0$	$0.40B_0$	$0.50B_0$	$0.60B_0$
$\sigma^{4,4}\pi_+^{2,1}\pi_-^{2,1}$	−288.858937	−288.929642	−288.946295	−288.917321	−288.850248	−288.751038	−288.624368
$\sigma^{5,4}\pi_+^{2,1}\pi_-^{1,1}$	−288.855707	−288.880757	−288.859418	−288.798370	−288.703789	−288.580690	−288.433074
$\sigma^{5,4}\pi_+^{1,1}\pi_-^{2,1}$	−288.855707	−288.980757	−289.059418	−289.098370	−289.103789	−289.080690	−289.033074
$\sigma^{5,3}\pi_+^{2,1}\pi_-^{2,1}$	−288.763296	−288.936672	−289.060277	−289.141076	−289.185716	−289.199608	−289.187026
$\sigma^{5,3}\pi_+^{1,1}\pi_-^{2,1}\delta_-^{1,0}$	−288.501032	−288.795053	−289.031911	−289.229490	−289.394397	−289.531624	−289.645101
$\sigma^{5,3}\pi_+^{1,1}\pi_-^{3,1}$	−288.503624	−288.763006	−288.970574	−289.139634	−289.276621	−289.386432	−289.472894
$\sigma^{4,3}\pi_+^{1,1}\pi_-^{3,1}\delta_-^{1,0}$	−288.174882	−288.521171	−288.794298	−289.019611	−289.208121	−289.366584	−289.499780
$\sigma^{4,3}\pi_+^{1,1}\pi_-^{2,1}\delta_-^{1,0}\phi_-^{1,0}$	−288.118896	−288.474377	−288.744080	−288.967869	−289.156693	−289.316864	−289.452749

Table S427: Total energies in E_h for the Si atom in the 6-311++G(3df,3pd) basis set in fully uncontracted form, employing the real-orbital approximation.

	$0.00B_0$	$0.10B_0$	$0.20B_0$	$0.30B_0$	$0.40B_0$	$0.50B_0$	$0.60B_0$
$\sigma^{4,4}\pi_+^{2,1}\pi_-^{2,1}$	−288.853066	−288.923713	−288.939844	−288.909688	−288.841045	−288.739901	−288.610549
$\sigma^{5,4}\pi_+^{2,1}\pi_-^{1,1}$	−288.849966	−288.874922	−288.852871	−288.790093	−288.692889	−288.566519	−288.415057
$\sigma^{5,4}\pi_+^{1,1}\pi_-^{2,1}$	−288.849966	−288.974922	−289.052871	−289.090093	−289.092889	−289.066519	−289.015057
$\sigma^{5,3}\pi_+^{2,1}\pi_-^{2,1}$	−288.757453	−288.930740	−289.053672	−289.132804	−289.174882	−289.185490	−289.168938
$\sigma^{5,3}\pi_+^{1,1}\pi_-^{2,1}\delta_-^{1,0}$	−288.442089	−288.760026	−289.016202	−289.216805	−289.370822	−289.489586	−289.585116
$\sigma^{5,3}\pi_+^{1,1}\pi_-^{3,1}$	−288.492931	−288.727315	−288.950388	−289.122818	−289.253486	−289.349127	−289.416944
$\sigma^{4,3}\pi_+^{1,1}\pi_-^{3,1}\delta_-^{1,0}$	−288.139585	−288.466244	−288.767344	−288.994617	−289.167018	−289.297332	−289.399385
$\sigma^{4,3}\pi_+^{1,1}\pi_-^{2,1}\delta_-^{1,0}\phi_-^{1,0}$	−287.318520	−287.782657	−288.177266	−288.508072	−288.783354	−289.013393	−289.208673

Table S428: Total energies in E_h for the Si atom in the def2-TZVP basis set in fully uncontracted form, employing the real-orbital approximation.

	$0.00B_0$	$0.10B_0$	$0.20B_0$	$0.30B_0$	$0.40B_0$	$0.50B_0$	$0.60B_0$
$\sigma^{4,4}\pi_+^{2,1}\pi_-^{2,1}$	−288.851766	−288.922440	−288.937997	−288.906847	−288.837814	−288.737352	−288.609566
$\sigma^{5,4}\pi_+^{2,1}\pi_-^{1,1}$	−288.848587	−288.873579	−288.851102	−288.787396	−288.689507	−288.563264	−288.412828
$\sigma^{5,4}\pi_+^{1,1}\pi_-^{2,1}$	−288.848587	−288.973579	−289.051102	−289.087396	−289.089507	−289.063264	−289.012828
$\sigma^{5,3}\pi_+^{2,1}\pi_-^{2,1}$	−288.756206	−288.929366	−289.051710	−289.130121	−289.172043	−289.183264	−289.167783
$\sigma^{5,3}\pi_+^{1,1}\pi_-^{2,1}\delta_-^{1,0}$	−288.397565	−288.719086	−288.985633	−289.202291	−289.375538	−289.512136	−289.618836
$\sigma^{5,3}\pi_+^{1,1}\pi_-^{3,1}$	−288.385824	−288.692712	−288.919473	−289.102446	−289.246943	−289.357970	−289.440035
$\sigma^{4,3}\pi_+^{1,1}\pi_-^{3,1}\delta_-^{1,0}$	−288.011951	−288.404507	−288.713191	−288.966147	−289.169479	−289.330032	−289.455141
$\sigma^{4,3}\pi_+^{1,1}\pi_-^{2,1}\delta_-^{1,0}\phi_-^{1,0}$	−287.235680	−287.703645	−288.109323	−288.457202	−288.752843	−289.001989	−289.210415

Table S429: Total energies in E_h for the P atom in the cc-pVDZ basis set in fully uncontracted form, employing the real-orbital approximation.

	$0.00B_0$	$0.10B_0$	$0.20B_0$	$0.30B_0$	$0.40B_0$	$0.50B_0$	$0.60B_0$
$\sigma^{5,4}\pi_+^{2,1}\pi_-^{2,1}$	−340.709290	−340.834481	−340.911072	−340.941969	−340.931538	−340.884938	−340.807375
$\sigma^{4,5}\pi_+^{2,1}\pi_-^{2,1}$	−340.676754	−340.701896	−340.678350	−340.609037	−340.498343	−340.351435	−340.173503
$\sigma^{5,4}\pi_+^{1,2}\pi_-^{2,1}$	−340.665647	−340.690605	−340.666497	−340.596238	−340.484232	−340.335731	−340.156075
$\sigma^{5,4}\pi_+^{1,1}\pi_-^{2,2}$	−340.619207	−340.743889	−340.818949	−340.847305	−340.833404	−340.782611	−340.700453
$\sigma^{5,4}\pi_+^{1,1}\pi_-^{2,1}\delta_-^{1,0}$	−340.058768	−340.335292	−340.565559	−340.751558	−340.896285	−341.003310	−341.076293
$\sigma^{5,3}\pi_+^{2,1}\pi_-^{2,1}\delta_-^{1,0}$	−339.992566	−340.317499	−340.593138	−340.821859	−341.007173	−341.153127	−341.263664
$\sigma^{5,3}\pi_+^{1,1}\pi_-^{3,1}\delta_-^{1,0}$	−339.339058	−339.766643	−340.149934	−340.490444	−340.790407	−341.052400	−341.278958
$\sigma^{5,3}\pi_+^{1,1}\pi_-^{2,1}\delta_-^{1,0}\phi_-^{1,0}$							
$\sigma^{6,3}\pi_+^{1,1}\pi_-^{2,1}\delta_-^{1,0}$	−339.407906	−339.780942	−340.100967	−340.372822	−340.613000	−340.827060	−341.009631

Table S430: Total energies in E_h for the P atom in the cc-pVTZ basis set in fully uncontracted form, employing the real-orbital approximation.

	$0.00B_0$	$0.10B_0$	$0.20B_0$	$0.30B_0$	$0.40B_0$	$0.50B_0$	$0.60B_0$
$\sigma^{5,4}\pi_+^{2,1}\pi_-^{2,1}$	−340.716455	−340.841485	−340.918093	−340.950352	−340.943685	−340.903518	−340.834388
$\sigma^{4,5}\pi_+^{2,1}\pi_-^{2,1}$	−340.688814	−340.713766	−340.690169	−340.622159	−340.515211	−340.374762	−340.205332
$\sigma^{5,4}\pi_+^{1,2}\pi_-^{2,1}$	−340.674212	−340.698909	−340.674571	−340.605430	−340.497140	−340.355357	−340.184773
$\sigma^{5,4}\pi_+^{1,1}\pi_-^{2,2}$	−340.628403	−340.752698	−340.827209	−340.856347	−340.846059	−340.802327	−340.730079
$\sigma^{5,4}\pi_+^{1,1}\pi_-^{2,1}\delta_-^{1,0}$	−340.241563	−340.515747	−340.739374	−340.915401	−341.047982	−341.141730	−341.201227
$\sigma^{5,3}\pi_+^{2,1}\pi_-^{2,1}\delta_-^{1,0}$	−340.161207	−340.484094	−340.753971	−340.974138	−341.149138	−341.283860	−341.382984
$\sigma^{5,3}\pi_+^{1,1}\pi_-^{3,1}\delta_-^{1,0}$	−339.668899	−340.092989	−340.466088	−340.790506	−341.069581	−341.307217	−341.507579
$\sigma^{5,3}\pi_+^{1,1}\pi_-^{2,1}\delta_-^{1,0}\phi_-^{1,0}$	−338.592722	−339.116064	−339.586941	−340.007677	−340.381521	−340.712092	−341.003032
$\sigma^{6,3}\pi_+^{1,1}\pi_-^{2,1}\delta_-^{1,0}$	−339.681062	−340.049505	−340.352230	−340.594772	−340.896945	−341.097844	−341.263465

Table S431: Total energies in E_h for the P atom in the cc-pVQZ basis set in fully uncontracted form, employing the real-orbital approximation.

	$0.00B_0$	$0.10B_0$	$0.20B_0$	$0.30B_0$	$0.40B_0$	$0.50B_0$	$0.60B_0$
$\sigma^{5,4}\pi_+^{2,1}\pi_-^{2,1}$	−340.718820	−340.843806	−340.920695	−340.954236	−340.949917	−340.912564	−340.846048
$\sigma^{4,5}\pi_+^{2,1}\pi_-^{2,1}$	−340.691947	−340.716810	−340.693399	−340.626600	−340.522001	−340.384463	−340.217860
$\sigma^{5,4}\pi_+^{1,2}\pi_-^{2,1}$	−340.676853	−340.701431	−340.677250	−340.609406	−340.503702	−340.365107	−340.197498
$\sigma^{5,4}\pi_+^{1,1}\pi_-^{2,2}$	−340.631326	−340.755387	−340.829846	−340.860253	−340.852842	−340.812784	−340.743950
$\sigma^{5,4}\pi_+^{1,1}\pi_-^{2,1}\delta_-^{1,0}$	−340.303780	−340.575778	−340.793309	−340.960503	−341.083091	−341.167606	−341.220772
$\sigma^{5,3}\pi_+^{2,1}\pi_-^{2,1}\delta_-^{1,0}$	−340.212086	−340.533158	−340.798036	−341.011129	−341.178400	−341.306386	−341.401544
$\sigma^{5,3}\pi_+^{1,1}\pi_-^{3,1}\delta_-^{1,0}$	−339.764955	−340.186082	−340.550785	−340.862755	−341.127458	−341.351490	−341.541893
$\sigma^{5,3}\pi_+^{1,1}\pi_-^{2,1}\delta_-^{1,0}\phi_-^{1,0}$	−339.103632	−339.622032	−340.078406	−340.475992	−340.819504	−341.114527	−341.366923
$\sigma^{6,3}\pi_+^{1,1}\pi_-^{2,1}\delta_-^{1,0}$	−339.774680	−340.138441	−340.430772	−340.735414	−340.965302	−341.157092	−341.316532

Table S432: Total energies in E_h for the P atom in the cc-pV5Z basis set in fully uncontracted form, employing the real-orbital approximation.

	$0.00B_0$	$0.10B_0$	$0.20B_0$	$0.30B_0$	$0.40B_0$	$0.50B_0$	$0.60B_0$
$\sigma^{5,4}\pi_+^{2,1}\pi_-^{2,1}$	−340.719169	−340.844157	−340.921189	−340.955161	−340.951471	−340.914782	−340.848920
$\sigma^{4,5}\pi_+^{2,1}\pi_-^{2,1}$	−340.692472	−340.717330	−340.694058	−340.627723	−340.523836	−340.387099	−340.221341
$\sigma^{5,4}\pi_+^{1,2}\pi_-^{2,1}$	−340.677255	−340.701825	−340.677797	−340.610460	−340.505494	−340.367644	−340.200721
$\sigma^{5,4}\pi_+^{1,1}\pi_-^{2,2}$	−340.631776	−340.755806	−340.830410	−340.861406	−340.854858	−340.815614	−340.747439
$\sigma^{5,4}\pi_+^{1,1}\pi_-^{2,1}\delta_-^{1,0}$	−340.304879	−340.577088	−340.795352	−340.963984	−341.088852	−341.176544	−341.233712
$\sigma^{5,3}\pi_+^{2,1}\pi_-^{2,1}\delta_-^{1,0}$	−340.214266	−340.535565	−340.801209	−341.015750	−341.185229	−341.316195	−341.415023
$\sigma^{5,3}\pi_+^{1,1}\pi_-^{3,1}\delta_-^{1,0}$	−339.770360	−340.191884	−340.557856	−340.872158	−341.140458	−341.369483	−341.566230
$\sigma^{5,3}\pi_+^{1,1}\pi_-^{2,1}\delta_-^{1,0}\phi_-^{1,0}$	−339.290748	−339.806247	−340.254051	−340.637804	−340.962939	−341.236117	−341.464658
$\sigma^{6,3}\pi_+^{1,1}\pi_-^{2,1}\delta_-^{1,0}$	−339.804067	−340.167719	−340.473870	−340.746193	−340.977616	−341.172511	−341.336603

Table S433: Total energies in E_h for the P atom in the aug-cc-pVDZ basis set in fully uncontracted form, employing the real-orbital approximation.

	$0.00B_0$	$0.10B_0$	$0.20B_0$	$0.30B_0$	$0.40B_0$	$0.50B_0$	$0.60B_0$
$\sigma^{5,4}\pi_{+}^{2,1}\pi_{-}^{2,1}$	−340.710205	−340.835053	−340.911409	−340.943225	−340.935016	−340.891492	−340.817235
$\sigma^{4,5}\pi_{+}^{2,1}\pi_{-}^{2,1}$	−340.679229	−340.703936	−340.679983	−340.611456	−340.502891	−340.358961	−340.184194
$\sigma^{5,4}\pi_{+}^{1,2}\pi_{-}^{2,1}$	−340.667379	−340.691781	−340.667062	−340.597470	−340.487575	−340.342097	−340.165684
$\sigma^{5,4}\pi_{+}^{1,1}\pi_{-}^{2,2}$	−340.622049	−340.745813	−340.819656	−340.848323	−340.836451	−340.788759	−340.709983
$\sigma^{5,4}\pi_{+}^{1,1}\pi_{-}^{2,1}\delta_{-}^{1,0}$	−340.328064	−340.596247	−340.802728	−340.952965	−341.055525	−341.121043	−341.159232
$\sigma^{5,3}\pi_{+}^{2,1}\pi_{-}^{2,1}\delta_{-}^{1,0}$	−340.220460	−340.538070	−340.792934	−340.990754	−341.140101	−341.250872	−341.331573
$\sigma^{5,3}\pi_{+}^{1,1}\pi_{-}^{3,1}\delta_{-}^{1,0}$	−339.802865	−340.192998	−340.542578	−340.831956	−341.070540	−341.269379	−341.438331
$\sigma^{5,3}\pi_{+}^{1,1}\pi_{-}^{2,1}\delta_{-}^{1,0}\phi_{-}^{1,0}$							
$\sigma^{6,3}\pi_{+}^{1,1}\pi_{-}^{2,1}\delta_{-}^{1,0}$	−339.853690	−340.206620	−340.479720	−340.720660	−340.926033	−341.096045	−341.236697

Table S434: Total energies in E_h for the P atom in the aug-cc-pVTZ basis set in fully uncontracted form, employing the real-orbital approximation.

	$0.00B_0$	$0.10B_0$	$0.20B_0$	$0.30B_0$	$0.40B_0$	$0.50B_0$	$0.60B_0$
$\sigma^{5,4}\pi_{+}^{2,1}\pi_{-}^{2,1}$	−340.716617	−340.841563	−340.918507	−340.952210	−340.948101	−340.911216	−340.845623
$\sigma^{4,5}\pi_{+}^{2,1}\pi_{-}^{2,1}$	−340.689674	−340.714478	−340.691101	−340.624463	−340.520090	−340.383057	−340.217412
$\sigma^{5,4}\pi_{+}^{1,2}\pi_{-}^{2,1}$	−340.674700	−340.699225	−340.675149	−340.607548	−340.502047	−340.363835	−340.197080
$\sigma^{5,4}\pi_{+}^{1,1}\pi_{-}^{2,2}$	−340.629308	−340.753268	−340.827900	−340.858712	−340.851515	−340.811669	−340.743481
$\sigma^{5,4}\pi_{+}^{1,1}\pi_{-}^{2,1}\delta_{-}^{1,0}$	−340.371354	−340.634041	−340.827887	−340.969186	−341.076024	−341.156466	−341.211493
$\sigma^{5,3}\pi_{+}^{2,1}\pi_{-}^{2,1}\delta_{-}^{1,0}$	−340.256195	−340.569864	−340.816791	−341.011830	−341.169113	−341.295151	−341.392098
$\sigma^{5,3}\pi_{+}^{1,1}\pi_{-}^{3,1}\delta_{-}^{1,0}$	−339.835189	−340.245443	−340.584638	−340.868820	−341.114601	−341.330890	−341.520497
$\sigma^{5,3}\pi_{+}^{1,1}\pi_{-}^{2,1}\delta_{-}^{1,0}\phi_{-}^{1,0}$	−339.424259	−339.930700	−340.355346	−340.710954	−341.009331	−341.257220	−341.459823
$\sigma^{6,3}\pi_{+}^{1,1}\pi_{-}^{2,1}\delta_{-}^{1,0}$	−339.884445	−340.234613	−340.515604	−340.763603	−340.976478	−341.157886	−341.310613

Table S435: Total energies in E_h for the P atom in the aug-cc-pVQZ basis set in fully uncontracted form, employing the real-orbital approximation.

	$0.00B_0$	$0.10B_0$	$0.20B_0$	$0.30B_0$	$0.40B_0$	$0.50B_0$	$0.60B_0$
$\sigma^{5,4}\pi_{+}^{2,1}\pi_{-}^{2,1}$	−340.718842	−340.843832	−340.921020	−340.955329	−340.952011	−340.915591	−340.849773
$\sigma^{4,5}\pi_{+}^{2,1}\pi_{-}^{2,1}$	−340.692109	−340.716957	−340.693843	−340.627883	−340.524444	−340.388071	−340.222436
$\sigma^{5,4}\pi_{+}^{1,2}\pi_{-}^{2,1}$	−340.676933	−340.701494	−340.677645	−340.610689	−340.506123	−340.368571	−340.201733
$\sigma^{5,4}\pi_{+}^{1,1}\pi_{-}^{2,2}$	−340.631506	−340.755494	−340.830312	−340.861751	−340.855600	−340.816617	−340.748511
$\sigma^{5,4}\pi_{+}^{1,1}\pi_{-}^{2,1}\delta_{-}^{1,0}$	−340.388632	−340.646381	−340.832786	−340.975885	−341.088546	−341.171525	−341.226716
$\sigma^{5,3}\pi_{+}^{2,1}\pi_{-}^{2,1}\delta_{-}^{1,0}$	−340.268792	−340.579181	−340.822654	−341.020604	−341.182114	−341.309885	−341.407160
$\sigma^{5,3}\pi_{+}^{1,1}\pi_{-}^{3,1}\delta_{-}^{1,0}$	−339.851972	−340.259699	−340.595862	−340.884152	−341.137434	−341.359727	−341.553986
$\sigma^{5,3}\pi_{+}^{1,1}\pi_{-}^{2,1}\delta_{-}^{1,0}\phi_{-}^{1,0}$	−339.575697	−340.073123	−340.475197	−340.799416	−341.059839	−341.273178	−341.459766
$\sigma^{6,3}\pi_{+}^{1,1}\pi_{-}^{2,1}\delta_{-}^{1,0}$	−339.896004	−340.244001	−340.527338	−340.778261	−340.994882	−341.181059	−341.340838

Table S436: Total energies in E_h for the P atom in the aug-cc-pV5Z basis set in fully uncontracted form, employing the real-orbital approximation.

	$0.00B_0$	$0.10B_0$	$0.20B_0$	$0.30B_0$	$0.40B_0$	$0.50B_0$	$0.60B_0$
$\sigma^{5,4}\pi_{+}^{2,1}\pi_{-}^{2,1}$	−340.719176	−340.844174	−340.921405	−340.955785	−340.952495	−340.916057	−340.850316
$\sigma^{4,5}\pi_{+}^{2,1}\pi_{-}^{2,1}$	−340.692548	−340.717407	−340.694347	−340.628480	−340.525097	−340.388736	−340.223225
$\sigma^{5,4}\pi_{+}^{1,2}\pi_{-}^{2,1}$	−340.677290	−340.701859	−340.678059	−340.611197	−340.506684	−340.369110	−340.202317
$\sigma^{5,4}\pi_{+}^{1,1}\pi_{-}^{2,2}$	−340.631861	−340.755857	−340.830724	−340.862280	−340.856217	−340.817221	−340.749125
$\sigma^{5,4}\pi_{+}^{1,1}\pi_{-}^{2,1}\delta_{-}^{1,0}$	−340.387942	−340.646812	−340.835512	−340.980293	−341.094610	−341.179887	−341.237910
$\sigma^{5,3}\pi_{+}^{2,1}\pi_{-}^{2,1}\delta_{-}^{1,0}$	−340.269769	−340.581133	−340.826427	−341.025831	−341.188943	−341.318815	−341.418627
$\sigma^{5,3}\pi_{+}^{1,1}\pi_{-}^{3,1}\delta_{-}^{1,0}$	−339.857089	−340.265969	−340.604598	−340.895540	−341.151955	−341.378160	−341.576858
$\sigma^{5,3}\pi_{+}^{1,1}\pi_{-}^{2,1}\delta_{-}^{1,0}\phi_{-}^{1,0}$	−339.617915	−340.111931	−340.503948	−340.814674	−341.067469	−341.287306	−341.487576
$\sigma^{6,3}\pi_{+}^{1,1}\pi_{-}^{2,1}\delta_{-}^{1,0}$	−339.900239	−340.248248	−340.533582	−340.787811	−341.008573	−341.199589	−341.364523

Table S437: Total energies in E_h for the P atom in the HGBSP1-5 basis set in fully uncontracted form, employing the real-orbital approximation.

	$0.00B_0$	$0.10B_0$	$0.20B_0$	$0.30B_0$	$0.40B_0$	$0.50B_0$	$0.60B_0$
$\sigma^{5,4}\pi_+^{2,1}\pi_-^{2,1}$	−340.711669	−340.836641	−340.913521	−340.946664	−340.940863	−340.900577	−340.829645
$\sigma^{4,5}\pi_+^{2,1}\pi_-^{2,1}$	−340.681665	−340.706501	−340.683075	−340.615884	−340.509783	−340.369238	−340.198068
$\sigma^{5,4}\pi_+^{1,2}\pi_-^{2,1}$	−340.668895	−340.693390	−340.669082	−340.600673	−340.493142	−340.351057	−340.178325
$\sigma^{5,4}\pi_+^{1,1}\pi_-^{2,2}$	−340.623490	−340.747325	−340.821443	−340.851070	−340.841434	−340.797246	−340.722514
$\sigma^{5,4}\pi_+^{1,1}\pi_-^{2,1}\delta_-^{1,0}$	−340.406542	−340.644972	−340.828229	−340.973592	−341.084863	−341.165198	−341.218283
$\sigma^{5,3}\pi_+^{2,1}\pi_-^{2,1}\delta_-^{1,0}$	−340.275371	−340.576245	−340.819665	−341.018800	−341.178686	−341.303698	−341.397938
$\sigma^{5,3}\pi_+^{1,1}\pi_-^{3,1}\delta_-^{1,0}$	−339.857730	−340.260924	−340.597877	−340.888158	−341.140170	−341.359119	−341.549369
$\sigma^{5,3}\pi_+^{1,1}\pi_-^{2,1}\delta_-^{1,0}\phi_-^{1,0}$							
$\sigma^{6,3}\pi_+^{1,1}\pi_-^{2,1}\delta_-^{1,0}$	−339.887489	−340.238100	−340.527892	−340.779599	−340.993892	−341.175179	−341.327788

Table S438: Total energies in E_h for the P atom in the HGBSP1-7 basis set in fully uncontracted form, employing the real-orbital approximation.

	$0.00B_0$	$0.10B_0$	$0.20B_0$	$0.30B_0$	$0.40B_0$	$0.50B_0$	$0.60B_0$
$\sigma^{5,4}\pi_+^{2,1}\pi_-^{2,1}$	−340.719179	−340.844156	−340.921063	−340.954302	−340.948670	−340.908537	−340.837691
$\sigma^{4,5}\pi_+^{2,1}\pi_-^{2,1}$	−340.689161	−340.714002	−340.690600	−340.623508	−340.517590	−340.377223	−340.206164
$\sigma^{5,4}\pi_+^{1,2}\pi_-^{2,1}$	−340.676392	−340.700890	−340.676604	−340.608298	−340.500971	−340.359080	−340.186442
$\sigma^{5,4}\pi_+^{1,1}\pi_-^{2,2}$	−340.630988	−340.754826	−340.828959	−340.858689	−340.849297	−340.805352	−340.730710
$\sigma^{5,4}\pi_+^{1,1}\pi_-^{2,1}\delta_-^{1,0}$	−340.414111	−340.652572	−340.835888	−340.981241	−341.092608	−341.173377	−341.226688
$\sigma^{5,3}\pi_+^{2,1}\pi_-^{2,1}\delta_-^{1,0}$	−340.282910	−340.583774	−340.827253	−341.026387	−341.186429	−341.311721	−341.406034
$\sigma^{5,3}\pi_+^{1,1}\pi_-^{3,1}\delta_-^{1,0}$	−339.865251	−340.268437	−340.605451	−340.895720	−341.147817	−341.367108	−341.557607
$\sigma^{5,3}\pi_+^{1,1}\pi_-^{2,1}\delta_-^{1,0}\phi_-^{1,0}$							
$\sigma^{6,3}\pi_+^{1,1}\pi_-^{2,1}\delta_-^{1,0}$	−339.896597	−340.246639	−340.535567	−340.787187	−341.002221	−341.184714	−341.338328

Table S439: Total energies in E_h for the P atom in the HGBSP1-9 basis set in fully uncontracted form, employing the real-orbital approximation.

	$0.00B_0$	$0.10B_0$	$0.20B_0$	$0.30B_0$	$0.40B_0$	$0.50B_0$	$0.60B_0$
$\sigma^{5,4}\pi_+^{2,1}\pi_-^{2,1}$	−340.719274	−340.844251	−340.921160	−340.954404	−340.948783	−340.908674	−340.837859
$\sigma^{4,5}\pi_+^{2,1}\pi_-^{2,1}$	−340.689256	−340.714097	−340.690697	−340.623610	−340.517703	−340.377358	−340.206329
$\sigma^{5,4}\pi_+^{1,2}\pi_-^{2,1}$	−340.676487	−340.700985	−340.676702	−340.608400	−340.501083	−340.359217	−340.186614
$\sigma^{5,4}\pi_+^{1,1}\pi_-^{2,2}$	−340.631083	−340.754921	−340.829056	−340.858792	−340.849408	−340.805485	−340.730886
$\sigma^{5,4}\pi_+^{1,1}\pi_-^{2,1}\delta_-^{1,0}$	−340.414207	−340.652667	−340.835984	−340.981365	−341.092719	−341.173510	−341.226885
$\sigma^{5,3}\pi_+^{2,1}\pi_-^{2,1}\delta_-^{1,0}$	−340.283004	−340.583869	−340.827347	−341.026496	−341.186530	−341.311852	−341.406220
$\sigma^{5,3}\pi_+^{1,1}\pi_-^{3,1}\delta_-^{1,0}$	−339.865346	−340.268532	−340.605545	−340.895831	−341.147923	−341.367228	−341.557780
$\sigma^{5,3}\pi_+^{1,1}\pi_-^{2,1}\delta_-^{1,0}\phi_-^{1,0}$							
$\sigma^{6,3}\pi_+^{1,1}\pi_-^{2,1}\delta_-^{1,0}$	−339.904515	−340.250911	−340.536391	−340.787306	−341.002531	−341.185294	−341.338924

Table S440: Total energies in E_h for the P atom in the HGBSP2-5 basis set in fully uncontracted form, employing the real-orbital approximation.

	$0.00B_0$	$0.10B_0$	$0.20B_0$	$0.30B_0$	$0.40B_0$	$0.50B_0$	$0.60B_0$
$\sigma^{5,4}\pi_+^{2,1}\pi_-^{2,1}$	−340.711670	−340.836671	−340.913904	−340.948208	−340.944750	−340.908196	−340.842461
$\sigma^{4,5}\pi_+^{2,1}\pi_-^{2,1}$	−340.685082	−340.709945	−340.686886	−340.620914	−340.517278	−340.380658	−340.214942
$\sigma^{5,4}\pi_+^{1,2}\pi_-^{2,1}$	−340.669805	−340.694380	−340.670596	−340.603655	−340.498910	−340.361125	−340.194265
$\sigma^{5,4}\pi_+^{1,1}\pi_-^{2,2}$	−340.624377	−340.748379	−340.823282	−340.854780	−340.848446	−340.809156	−340.740943
$\sigma^{5,4}\pi_+^{1,1}\pi_-^{2,1}\delta_-^{1,0}$	−340.406590	−340.645084	−340.828617	−340.974835	−341.087885	−341.171141	−341.228381
$\sigma^{5,3}\pi_+^{2,1}\pi_-^{2,1}\delta_-^{1,0}$	−340.275397	−340.576325	−340.820015	−341.020098	−341.182075	−341.310656	−341.410138
$\sigma^{5,3}\pi_+^{1,1}\pi_-^{3,1}\delta_-^{1,0}$	−339.858800	−340.261996	−340.599349	−340.890529	−341.144218	−341.365900	−341.560133
$\sigma^{5,3}\pi_+^{1,1}\pi_-^{2,1}\delta_-^{1,0}\phi_-^{1,0}$	−339.789324	−340.198288	−340.526168	−340.810450	−341.060784	−341.284953	−341.482464
$\sigma^{6,3}\pi_+^{1,1}\pi_-^{2,1}\delta_-^{1,0}$	−339.887584	−340.238192	−340.528805	−340.782089	−340.998766	−341.183559	−341.340924

Table S441: Total energies in E_h for the P atom in the HGBSP2-7 basis set in fully uncontracted form, employing the real-orbital approximation.

	$0.00B_0$	$0.10B_0$	$0.20B_0$	$0.30B_0$	$0.40B_0$	$0.50B_0$	$0.60B_0$
$\sigma^{5,4}\pi_+^{2,1}\pi_-^{2,1}$	−340.719179	−340.844186	−340.921447	−340.955841	−340.952531	−340.916102	−340.850451
$\sigma^{4,5}\pi_+^{2,1}\pi_-^{2,1}$	−340.692578	−340.717445	−340.694413	−340.628536	−340.525059	−340.388574	−340.222948
$\sigma^{5,4}\pi_+^{1,2}\pi_-^{2,1}$	−340.677302	−340.701880	−340.678119	−340.611275	−340.506708	−340.369077	−340.202299
$\sigma^{5,4}\pi_+^{1,1}\pi_-^{2,2}$	−340.631875	−340.755880	−340.830798	−340.862395	−340.856272	−340.817173	−340.749035
$\sigma^{5,4}\pi_+^{1,1}\pi_-^{2,1}\delta_-^{1,0}$	−340.414159	−340.652683	−340.836275	−340.982481	−341.095618	−341.179286	−341.236732
$\sigma^{5,3}\pi_+^{2,1}\pi_-^{2,1}\delta_-^{1,0}$	−340.282935	−340.583854	−340.827602	−341.027681	−341.189802	−341.318648	−341.418202
$\sigma^{5,3}\pi_+^{1,1}\pi_-^{3,1}\delta_-^{1,0}$	−339.866320	−340.269508	−340.606922	−340.898086	−341.151858	−341.373875	−341.568349
$\sigma^{5,3}\pi_+^{1,1}\pi_-^{2,1}\delta_-^{1,0}\phi_-^{1,0}$	−339.796872	−340.205805	−340.533941	−340.819118	−341.070156	−341.293002	−341.490798
$\sigma^{6,3}\pi_+^{1,1}\pi_-^{2,1}\delta_-^{1,0}$	−339.896689	−340.246730	−340.536472	−340.789679	−341.007076	−341.193021	−341.351321

Table S442: Total energies in E_h for the P atom in the HGBSP2-9 basis set in fully uncontracted form, employing the real-orbital approximation.

	$0.00B_0$	$0.10B_0$	$0.20B_0$	$0.30B_0$	$0.40B_0$	$0.50B_0$	$0.60B_0$
$\sigma^{5,4}\pi_+^{2,1}\pi_-^{2,1}$	−340.719274	−340.844281	−340.921543	−340.955943	−340.952643	−340.916235	−340.850604
$\sigma^{4,5}\pi_+^{2,1}\pi_-^{2,1}$	−340.692673	−340.717541	−340.694510	−340.628638	−340.525171	−340.388705	−340.223098
$\sigma^{5,4}\pi_+^{1,2}\pi_-^{2,1}$	−340.677397	−340.701975	−340.678216	−340.611377	−340.506820	−340.369210	−340.202453
$\sigma^{5,4}\pi_+^{1,1}\pi_-^{2,2}$	−340.631970	−340.755974	−340.830895	−340.862498	−340.856382	−340.817302	−340.749191
$\sigma^{5,4}\pi_+^{1,1}\pi_-^{2,1}\delta_-^{1,0}$	−340.414255	−340.652778	−340.836371	−340.982605	−341.095729	−341.179417	−341.236921
$\sigma^{5,3}\pi_+^{2,1}\pi_-^{2,1}\delta_-^{1,0}$	−340.283029	−340.583949	−340.827696	−341.027789	−341.189904	−341.318776	−341.418376
$\sigma^{5,3}\pi_+^{1,1}\pi_-^{3,1}\delta_-^{1,0}$	−339.866415	−340.269604	−340.607017	−340.898197	−341.151963	−341.373993	−341.568516
$\sigma^{5,3}\pi_+^{1,1}\pi_-^{2,1}\delta_-^{1,0}\phi_-^{1,0}$	−339.796968	−340.205949	−340.534148	−340.819227	−341.070487	−341.293205	−341.490964
$\sigma^{6,3}\pi_+^{1,1}\pi_-^{2,1}\delta_-^{1,0}$	−339.904603	−340.251003	−340.537261	−340.789795	−341.007380	−341.193574	−341.351875

Table S443: Total energies in E_h for the P atom in the HGBSP3-5 basis set in fully uncontracted form, employing the real-orbital approximation.

	$0.00B_0$	$0.10B_0$	$0.20B_0$	$0.30B_0$	$0.40B_0$	$0.50B_0$	$0.60B_0$
$\sigma^{5,4}\pi_+^{2,1}\pi_-^{2,1}$	−340.711669	−340.836671	−340.913904	−340.948210	−340.944764	−340.908249	−340.842617
$\sigma^{4,5}\pi_+^{2,1}\pi_-^{2,1}$	−340.685099	−340.709964	−340.686914	−340.620975	−340.517418	−340.380957	−340.215533
$\sigma^{5,4}\pi_+^{1,2}\pi_-^{2,1}$	−340.669806	−340.694381	−340.670599	−340.603665	−340.498944	−340.361220	−340.194492
$\sigma^{5,4}\pi_+^{1,1}\pi_-^{2,2}$	−340.624378	−340.748380	−340.823283	−340.854789	−340.848476	−340.809243	−340.741156
$\sigma^{5,4}\pi_+^{1,1}\pi_-^{2,1}\delta_-^{1,0}$	−340.406596	−340.645414	−340.829513	−340.976363	−341.090184	−341.174365	−341.232626
$\sigma^{5,3}\pi_+^{2,1}\pi_-^{2,1}\delta_-^{1,0}$	−340.275397	−340.576505	−340.820610	−341.021269	−341.184014	−341.313534	−341.414072
$\sigma^{5,3}\pi_+^{1,1}\pi_-^{3,1}\delta_-^{1,0}$	−339.858849	−340.262373	−340.601147	−340.894664	−341.151468	−341.377011	−341.575694
$\sigma^{5,3}\pi_+^{1,1}\pi_-^{2,1}\delta_-^{1,0}\phi_-^{1,0}$	−339.789342	−340.198316	−340.526391	−340.811097	−341.062068	−341.287052	−341.485522
$\sigma^{6,3}\pi_+^{1,1}\pi_-^{2,1}\delta_-^{1,0}$	−339.887603	−340.238287	−340.530939	−340.788935	−341.012466	−341.205547	−341.372036

Table S444: Total energies in E_h for the P atom in the HGBSP3-7 basis set in fully uncontracted form, employing the real-orbital approximation.

	$0.00B_0$	$0.10B_0$	$0.20B_0$	$0.30B_0$	$0.40B_0$	$0.50B_0$	$0.60B_0$
$\sigma^{5,4}\pi_+^{2,1}\pi_-^{2,1}$	−340.719179	−340.844186	−340.921447	−340.955844	−340.952545	−340.916157	−340.850607
$\sigma^{4,5}\pi_+^{2,1}\pi_-^{2,1}$	−340.692596	−340.717465	−340.694441	−340.628597	−340.525199	−340.388874	−340.223536
$\sigma^{5,4}\pi_+^{1,2}\pi_-^{2,1}$	−340.677304	−340.701881	−340.678122	−340.611286	−340.506743	−340.369173	−340.202524
$\sigma^{5,4}\pi_+^{1,1}\pi_-^{2,2}$	−340.631876	−340.755881	−340.830800	−340.862404	−340.856303	−340.817261	−340.749246
$\sigma^{5,4}\pi_+^{1,1}\pi_-^{2,1}\delta_-^{1,0}$	−340.414164	−340.653010	−340.837155	−340.984014	−341.097905	−341.182451	−341.240926
$\sigma^{5,3}\pi_+^{2,1}\pi_-^{2,1}\delta_-^{1,0}$	−340.282935	−340.584034	−340.828190	−341.028856	−341.191728	−341.321485	−341.422106
$\sigma^{5,3}\pi_+^{1,1}\pi_-^{3,1}\delta_-^{1,0}$	−339.866369	−340.269885	−340.608709	−340.902218	−341.159103	−341.384898	−341.583758
$\sigma^{5,3}\pi_+^{1,1}\pi_-^{2,1}\delta_-^{1,0}\phi_-^{1,0}$	−339.785712	−340.205833	−340.534164	−340.819768	−341.071428	−341.295075	−341.493839
$\sigma^{6,3}\pi_+^{1,1}\pi_-^{2,1}\delta_-^{1,0}$	−339.896708	−340.246825	−340.538645	−340.796464	−341.020293	−341.213971	−341.381059

Table S445: Total energies in E_h for the P atom in the HGBSP3-9 basis set in fully uncontracted form, employing the real-orbital approximation.

	$0.00B_0$	$0.10B_0$	$0.20B_0$	$0.30B_0$	$0.40B_0$	$0.50B_0$	$0.60B_0$
$\sigma^{5,4}\pi_+^{2,1}\pi_-^{2,1}$	−340.719274	−340.844281	−340.921544	−340.955945	−340.952657	−340.916290	−340.850760
$\sigma^{4,5}\pi_+^{2,1}\pi_-^{2,1}$	−340.692691	−340.717560	−340.694539	−340.628699	−340.525311	−340.389005	−340.223685
$\sigma^{5,4}\pi_+^{1,2}\pi_-^{2,1}$	−340.677398	−340.701976	−340.678220	−340.611389	−340.506855	−340.369306	−340.202678
$\sigma^{5,4}\pi_+^{1,1}\pi_-^{2,2}$	−340.631970	−340.755975	−340.830898	−340.862507	−340.856413	−340.817391	−340.749402
$\sigma^{5,4}\pi_+^{1,1}\pi_-^{2,1}\delta_-^{1,0}$	−340.414261	−340.653105	−340.837251	−340.984133	−341.098017	−341.182581	−341.241102
$\sigma^{5,3}\pi_+^{2,1}\pi_-^{2,1}\delta_-^{1,0}$	−340.283030	−340.584128	−340.828284	−341.028962	−341.191830	−341.321611	−341.422271
$\sigma^{5,3}\pi_+^{1,1}\pi_-^{3,1}\delta_-^{1,0}$	−339.866464	−340.269981	−340.608804	−340.902324	−341.159205	−341.385016	−341.583912
$\sigma^{5,3}\pi_+^{1,1}\pi_-^{2,1}\delta_-^{1,0}\phi_-^{1,0}$	−339.796987	−340.205977	−340.534370	−340.819876	−341.071759	−341.295276	−341.494000
$\sigma^{6,3}\pi_+^{1,1}\pi_-^{2,1}\delta_-^{1,0}$	−339.904623	−340.251129	−340.539748	−340.796767	−341.020424	−341.214164	−341.381353

Table S446: Total energies in E_h for the P atom in the AHGBSP1-5 basis set in fully uncontracted form, employing the real-orbital approximation.

	$0.00B_0$	$0.10B_0$	$0.20B_0$	$0.30B_0$	$0.40B_0$	$0.50B_0$	$0.60B_0$
$\sigma^{5,4}\pi_+^{2,1}\pi_-^{2,1}$	−340.711675	−340.836648	−340.913531	−340.946691	−340.940922	−340.900673	−340.829770
$\sigma^{4,5}\pi_+^{2,1}\pi_-^{2,1}$	−340.681668	−340.706505	−340.683082	−340.615909	−340.509843	−340.369337	−340.198198
$\sigma^{5,4}\pi_+^{1,2}\pi_-^{2,1}$	−340.668898	−340.693393	−340.669088	−340.600697	−340.493202	−340.351159	−340.178460
$\sigma^{5,4}\pi_+^{1,1}\pi_-^{2,2}$	−340.623493	−340.747329	−340.821448	−340.851092	−340.841496	−340.797353	−340.722655
$\sigma^{5,4}\pi_+^{1,1}\pi_-^{2,1}\delta_-^{1,0}$	−340.406563	−340.644989	−340.828243	−340.973616	−341.084909	−341.165278	−341.218393
$\sigma^{5,3}\pi_+^{2,1}\pi_-^{2,1}\delta_-^{1,0}$	−340.275389	−340.576258	−340.819681	−341.018828	−341.178734	−341.303767	−341.398018
$\sigma^{5,3}\pi_+^{1,1}\pi_-^{3,1}\delta_-^{1,0}$	−339.857742	−340.260933	−340.597888	−340.888177	−341.140203	−341.359170	−341.549434
$\sigma^{5,3}\pi_+^{1,1}\pi_-^{2,1}\delta_-^{1,0}\phi_-^{1,0}$							
$\sigma^{6,3}\pi_+^{1,1}\pi_-^{2,1}\delta_-^{1,0}$	−339.897020	−340.243005	−340.528660	−340.779617	−340.994281	−341.176082	−341.328941

Table S447: Total energies in E_h for the P atom in the AHGBSP1-7 basis set in fully uncontracted form, employing the real-orbital approximation.

	$0.00B_0$	$0.10B_0$	$0.20B_0$	$0.30B_0$	$0.40B_0$	$0.50B_0$	$0.60B_0$
$\sigma^{5,4}\pi_+^{2,1}\pi_-^{2,1}$	−340.719180	−340.844157	−340.921064	−340.954306	−340.948678	−340.908548	−340.837703
$\sigma^{4,5}\pi_+^{2,1}\pi_-^{2,1}$	−340.689162	−340.714002	−340.690602	−340.623512	−340.517598	−340.377234	−340.206176
$\sigma^{5,4}\pi_+^{1,2}\pi_-^{2,1}$	−340.676392	−340.700890	−340.676606	−340.608302	−340.500979	−340.359092	−340.186455
$\sigma^{5,4}\pi_+^{1,1}\pi_-^{2,2}$	−340.630989	−340.754826	−340.828960	−340.858693	−340.849305	−340.805364	−340.730722
$\sigma^{5,4}\pi_+^{1,1}\pi_-^{2,1}\delta_-^{1,0}$	−340.414112	−340.652573	−340.835889	−340.981245	−341.092614	−341.173386	−341.226698
$\sigma^{5,3}\pi_+^{2,1}\pi_-^{2,1}\delta_-^{1,0}$	−340.282910	−340.583775	−340.827254	−341.026389	−341.186431	−341.311725	−341.406038
$\sigma^{5,3}\pi_+^{1,1}\pi_-^{3,1}\delta_-^{1,0}$	−339.865251	−340.268437	−340.605451	−340.895722	−341.147819	−341.367111	−341.557610
$\sigma^{5,3}\pi_+^{1,1}\pi_-^{2,1}\delta_-^{1,0}\phi_-^{1,0}$							
$\sigma^{6,3}\pi_+^{1,1}\pi_-^{2,1}\delta_-^{1,0}$	−339.904606	−340.250800	−340.536290	−340.787193	−341.002379	−341.185043	−341.338613

Table S448: Total energies in E_h for the P atom in the AHGBSP1-9 basis set in fully uncontracted form, employing the real-orbital approximation.

	$0.00B_0$	$0.10B_0$	$0.20B_0$	$0.30B_0$	$0.40B_0$	$0.50B_0$	$0.60B_0$
$\sigma^{5,4}\pi_+^{2,1}\pi_-^{2,1}$	−340.719274	−340.844251	−340.921160	−340.954404	−340.948783	−340.908675	−340.837859
$\sigma^{4,5}\pi_+^{2,1}\pi_-^{2,1}$	−340.689256	−340.714097	−340.690697	−340.623610	−340.517703	−340.377358	−340.206330
$\sigma^{5,4}\pi_+^{1,2}\pi_-^{2,1}$	−340.676487	−340.700985	−340.676702	−340.608400	−340.501084	−340.359217	−340.186614
$\sigma^{5,4}\pi_+^{1,1}\pi_-^{2,2}$	−340.631083	−340.754921	−340.829056	−340.858792	−340.849408	−340.805485	−340.730887
$\sigma^{5,4}\pi_+^{1,1}\pi_-^{2,1}\delta_-^{1,0}$	−340.414207	−340.652667	−340.835984	−340.981365	−341.092720	−341.173510	−341.226885
$\sigma^{5,3}\pi_+^{2,1}\pi_-^{2,1}\delta_-^{1,0}$	−340.283004	−340.583869	−340.827347	−341.026496	−341.186530	−341.311852	−341.406220
$\sigma^{5,3}\pi_+^{1,1}\pi_-^{3,1}\delta_-^{1,0}$	−339.865346	−340.268532	−340.605545	−340.895831	−341.147923	−341.367228	−341.557780
$\sigma^{5,3}\pi_+^{1,1}\pi_-^{2,1}\delta_-^{1,0}\phi_-^{1,0}$							
$\sigma^{6,3}\pi_+^{1,1}\pi_-^{2,1}\delta_-^{1,0}$	−339.904703	−340.250912	−340.536396	−340.787306	−341.002536	−341.185306	−341.338930

Table S449: Total energies in E_h for the P atom in the AHGBSP2-5 basis set in fully uncontracted form, employing the real-orbital approximation.

	$0.00B_0$	$0.10B_0$	$0.20B_0$	$0.30B_0$	$0.40B_0$	$0.50B_0$	$0.60B_0$
$\sigma^{5,4}\pi_+^{2,1}\pi_-^{2,1}$	−340.711675	−340.836677	−340.913915	−340.948233	−340.944803	−340.908280	−340.842572
$\sigma^{4,5}\pi_+^{2,1}\pi_-^{2,1}$	−340.685085	−340.709948	−340.686893	−340.620938	−340.517332	−340.380743	−340.215054
$\sigma^{5,4}\pi_+^{1,2}\pi_-^{2,1}$	−340.669808	−340.694383	−340.670602	−340.603676	−340.498963	−340.361214	−340.194383
$\sigma^{5,4}\pi_+^{1,1}\pi_-^{2,2}$	−340.624381	−340.748383	−340.823287	−340.854800	−340.848499	−340.809248	−340.741065
$\sigma^{5,4}\pi_+^{1,1}\pi_-^{2,1}\delta_-^{1,0}$	−340.406611	−340.645100	−340.828631	−340.974859	−341.087929	−341.171214	−341.228481
$\sigma^{5,3}\pi_+^{2,1}\pi_-^{2,1}\delta_-^{1,0}$	−340.275415	−340.576339	−340.820031	−341.020124	−341.182118	−341.310718	−341.410209
$\sigma^{5,3}\pi_+^{1,1}\pi_-^{3,1}\delta_-^{1,0}$	−339.858812	−340.262006	−340.599361	−340.890548	−341.144249	−341.365946	−341.560192
$\sigma^{5,3}\pi_+^{1,1}\pi_-^{2,1}\delta_-^{1,0}\phi_-^{1,0}$	−339.789339	−340.198304	−340.526188	−340.810474	−341.060816	−341.284988	−341.482506
$\sigma^{6,3}\pi_+^{1,1}\pi_-^{2,1}\delta_-^{1,0}$	−339.897108	−340.243097	−340.529536	−340.782107	−340.999149	−341.184425	−341.342013

Table S450: Total energies in E_h for the P atom in the AHGBSP2-7 basis set in fully uncontracted form, employing the real-orbital approximation.

	$0.00B_0$	$0.10B_0$	$0.20B_0$	$0.30B_0$	$0.40B_0$	$0.50B_0$	$0.60B_0$
$\sigma^{5,4}\pi_+^{2,1}\pi_-^{2,1}$	−340.719180	−340.844186	−340.921448	−340.955845	−340.952538	−340.916112	−340.850462
$\sigma^{4,5}\pi_+^{2,1}\pi_-^{2,1}$	−340.692579	−340.717446	−340.694414	−340.628540	−340.525066	−340.388584	−340.222958
$\sigma^{5,4}\pi_+^{1,2}\pi_-^{2,1}$	−340.677303	−340.701880	−340.678120	−340.611279	−340.506716	−340.369088	−340.202310
$\sigma^{5,4}\pi_+^{1,1}\pi_-^{2,2}$	−340.631875	−340.755880	−340.830799	−340.862399	−340.856280	−340.817183	−340.749045
$\sigma^{5,4}\pi_+^{1,1}\pi_-^{2,1}\delta_-^{1,0}$	−340.414160	−340.652684	−340.836276	−340.982485	−341.095623	−341.179294	−341.236741
$\sigma^{5,3}\pi_+^{2,1}\pi_-^{2,1}\delta_-^{1,0}$	−340.282935	−340.583854	−340.827602	−341.027682	−341.189805	−341.318651	−341.418205
$\sigma^{5,3}\pi_+^{1,1}\pi_-^{3,1}\delta_-^{1,0}$	−339.866320	−340.269509	−340.606923	−340.898088	−341.151860	−341.373877	−341.568352
$\sigma^{5,3}\pi_+^{1,1}\pi_-^{2,1}\delta_-^{1,0}\phi_-^{1,0}$	−339.796872	−340.205807	−340.533943	−340.819119	−341.070157	−341.293004	−341.490799
$\sigma^{6,3}\pi_+^{1,1}\pi_-^{2,1}\delta_-^{1,0}$	−339.904694	−340.250891	−340.537160	−340.789683	−341.007231	−341.193330	−341.351581

Table S451: Total energies in E_h for the P atom in the AHGBSP2-9 basis set in fully uncontracted form, employing the real-orbital approximation.

	$0.00B_0$	$0.10B_0$	$0.20B_0$	$0.30B_0$	$0.40B_0$	$0.50B_0$	$0.60B_0$
$\sigma^{5,4}\pi_+^{2,1}\pi_-^{2,1}$	−340.719274	−340.844281	−340.921543	−340.955943	−340.952643	−340.916235	−340.850604
$\sigma^{4,5}\pi_+^{2,1}\pi_-^{2,1}$	−340.692673	−340.717540	−340.694510	−340.628638	−340.525171	−340.388705	−340.223098
$\sigma^{5,4}\pi_+^{1,2}\pi_-^{2,1}$	−340.677397	−340.701975	−340.678216	−340.611378	−340.506820	−340.369210	−340.202453
$\sigma^{5,4}\pi_+^{1,1}\pi_-^{2,2}$	−340.631970	−340.755974	−340.830895	−340.862498	−340.856382	−340.817303	−340.749191
$\sigma^{5,4}\pi_+^{1,1}\pi_-^{2,1}\delta_-^{1,0}$	−340.414255	−340.652778	−340.836371	−340.982605	−341.095729	−341.179417	−341.236921
$\sigma^{5,3}\pi_+^{2,1}\pi_-^{2,1}\delta_-^{1,0}$	−340.283029	−340.583949	−340.827696	−341.027789	−341.189904	−341.318776	−341.418376
$\sigma^{5,3}\pi_+^{1,1}\pi_-^{3,1}\delta_-^{1,0}$	−339.866415	−340.269604	−340.607017	−340.898197	−341.151963	−341.373993	−341.568516
$\sigma^{5,3}\pi_+^{1,1}\pi_-^{2,1}\delta_-^{1,0}\phi_-^{1,0}$	−339.796968	−340.205949	−340.534148	−340.819227	−341.070487	−341.293205	−341.490964
$\sigma^{6,3}\pi_+^{1,1}\pi_-^{2,1}\delta_-^{1,0}$	−339.904791	−340.251004	−340.537266	−340.789795	−341.007386	−341.193585	−341.351880

Table S452: Total energies in E_h for the P atom in the AHGBSP3-5 basis set in fully uncontracted form, employing the real-orbital approximation.

	$0.00B_0$	$0.10B_0$	$0.20B_0$	$0.30B_0$	$0.40B_0$	$0.50B_0$	$0.60B_0$
$\sigma^{5,4}\pi_+^{2,1}\pi_-^{2,1}$	−340.711675	−340.836677	−340.913914	−340.948235	−340.944817	−340.908335	−340.842729
$\sigma^{4,5}\pi_+^{2,1}\pi_-^{2,1}$	−340.685102	−340.709967	−340.686922	−340.620999	−340.517471	−340.381044	−340.215644
$\sigma^{5,4}\pi_+^{1,2}\pi_-^{2,1}$	−340.669809	−340.694384	−340.670605	−340.603687	−340.498998	−340.361310	−340.194609
$\sigma^{5,4}\pi_+^{1,1}\pi_-^{2,2}$	−340.624381	−340.748383	−340.823289	−340.854809	−340.848530	−340.809336	−340.741278
$\sigma^{5,4}\pi_+^{1,1}\pi_-^{2,1}\delta_-^{1,0}$	−340.406617	−340.645431	−340.829527	−340.976386	−341.090228	−341.174437	−341.232724
$\sigma^{5,3}\pi_+^{2,1}\pi_-^{2,1}\delta_-^{1,0}$	−340.275415	−340.576518	−340.820626	−341.021296	−341.184058	−341.313594	−341.414142
$\sigma^{5,3}\pi_+^{1,1}\pi_-^{3,1}\delta_-^{1,0}$	−339.858861	−340.262383	−340.601159	−340.894682	−341.151498	−341.377055	−341.575749
$\sigma^{5,3}\pi_+^{1,1}\pi_-^{2,1}\delta_-^{1,0}\phi_-^{1,0}$	−339.789358	−340.198331	−340.526411	−340.811121	−341.062101	−341.287087	−341.485563
$\sigma^{6,3}\pi_+^{1,1}\pi_-^{2,1}\delta_-^{1,0}$	−339.897128	−340.243239	−340.532040	−340.789098	−341.012498	−341.205768	−341.372531

Table S453: Total energies in E_h for the P atom in the AHGBSP3-7 basis set in fully uncontracted form, employing the real-orbital approximation.

	$0.00B_0$	$0.10B_0$	$0.20B_0$	$0.30B_0$	$0.40B_0$	$0.50B_0$	$0.60B_0$
$\sigma^{5,4}\pi_+^{2,1}\pi_-^{2,1}$	−340.719180	−340.844186	−340.921448	−340.955847	−340.952552	−340.916167	−340.850617
$\sigma^{4,5}\pi_+^{2,1}\pi_-^{2,1}$	−340.692597	−340.717465	−340.694443	−340.628601	−340.525206	−340.388884	−340.223545
$\sigma^{5,4}\pi_+^{1,2}\pi_-^{2,1}$	−340.677304	−340.701882	−340.678124	−340.611290	−340.506751	−340.369185	−340.202534
$\sigma^{5,4}\pi_+^{1,1}\pi_-^{2,2}$	−340.631876	−340.755881	−340.830802	−340.862408	−340.856311	−340.817272	−340.749256
$\sigma^{5,4}\pi_+^{1,1}\pi_-^{2,1}\delta_-^{1,0}$	−340.414166	−340.653011	−340.837156	−340.984017	−341.097911	−341.182459	−341.240934
$\sigma^{5,3}\pi_+^{2,1}\pi_-^{2,1}\delta_-^{1,0}$	−340.282936	−340.584034	−340.828190	−341.028857	−341.191730	−341.321488	−341.422109
$\sigma^{5,3}\pi_+^{1,1}\pi_-^{3,1}\delta_-^{1,0}$	−339.866369	−340.269886	−340.608709	−340.902219	−341.159105	−341.384900	−341.583760
$\sigma^{5,3}\pi_+^{1,1}\pi_-^{2,1}\delta_-^{1,0}\phi_-^{1,0}$	−339.796891	−340.205835	−340.534165	−340.819768	−341.071430	−341.295076	−341.493841
$\sigma^{6,3}\pi_+^{1,1}\pi_-^{2,1}\delta_-^{1,0}$	−339.904714	−340.251019	−340.539648	−340.796659	−341.020301	−341.214007	−341.381172

Table S454: Total energies in E_h for the P atom in the AHGBSP3-9 basis set in fully uncontracted form, employing the real-orbital approximation.

	$0.00B_0$	$0.10B_0$	$0.20B_0$	$0.30B_0$	$0.40B_0$	$0.50B_0$	$0.60B_0$
$\sigma^{5,4}\pi_+^{2,1}\pi_-^{2,1}$	−340.719274	−340.844281	−340.921544	−340.955945	−340.952658	−340.916290	−340.850760
$\sigma^{4,5}\pi_+^{2,1}\pi_-^{2,1}$	−340.692691	−340.717560	−340.694539	−340.628699	−340.525311	−340.389005	−340.223685
$\sigma^{5,4}\pi_+^{1,2}\pi_-^{2,1}$	−340.677398	−340.701976	−340.678220	−340.611389	−340.506855	−340.369306	−340.202678
$\sigma^{5,4}\pi_+^{1,1}\pi_-^{2,2}$	−340.631970	−340.755975	−340.830898	−340.862507	−340.856413	−340.817391	−340.749402
$\sigma^{5,4}\pi_+^{1,1}\pi_-^{2,1}\delta_-^{1,0}$	−340.414261	−340.653105	−340.837251	−340.984133	−341.098017	−341.182581	−341.241103
$\sigma^{5,3}\pi_+^{2,1}\pi_-^{2,1}\delta_-^{1,0}$	−340.283030	−340.584128	−340.828284	−341.028962	−341.191830	−341.321611	−341.422271
$\sigma^{5,3}\pi_+^{1,1}\pi_-^{3,1}\delta_-^{1,0}$	−339.866464	−340.269981	−340.608804	−340.902324	−341.159205	−341.385016	−341.583912
$\sigma^{5,3}\pi_+^{1,1}\pi_-^{2,1}\delta_-^{1,0}\phi_-^{1,0}$	−339.796987	−340.205977	−340.534370	−340.819876	−341.071760	−341.295276	−341.494000
$\sigma^{6,3}\pi_+^{1,1}\pi_-^{2,1}\delta_-^{1,0}$	−339.904811	−340.251132	−340.539748	−340.796768	−341.020424	−341.214164	−341.381355

Table S455: Total energies in E_h for the P atom in the 6-311++G(3df,3pd) basis set in fully uncontracted form, employing the real-orbital approximation.

	$0.00B_0$	$0.10B_0$	$0.20B_0$	$0.30B_0$	$0.40B_0$	$0.50B_0$	$0.60B_0$
$\sigma^{5,4}\pi_+^{2,1}\pi_-^{2,1}$	−340.710150	−340.834910	−340.911141	−340.943505	−340.937435	−340.897842	−340.828658
$\sigma^{4,5}\pi_+^{2,1}\pi_-^{2,1}$	−340.682559	−340.707184	−340.683084	−340.615035	−340.508534	−340.368517	−340.198918
$\sigma^{5,4}\pi_+^{1,2}\pi_-^{2,1}$	−340.668124	−340.692426	−340.667476	−340.598274	−340.490535	−340.349373	−340.178808
$\sigma^{5,4}\pi_+^{1,1}\pi_-^{2,2}$	−340.622782	−340.746468	−340.819989	−340.848831	−340.839106	−340.796209	−340.724267
$\sigma^{5,4}\pi_+^{1,1}\pi_-^{2,1}\delta_-^{1,0}$	−340.322393	−340.592908	−340.805948	−340.965572	−341.077701	−341.149725	−341.190155
$\sigma^{5,3}\pi_+^{2,1}\pi_-^{2,1}\delta_-^{1,0}$	−340.225779	−340.545385	−340.805844	−341.011611	−341.169056	−341.285757	−341.369847
$\sigma^{5,3}\pi_+^{1,1}\pi_-^{3,1}\delta_-^{1,0}$	−339.821463	−340.206005	−340.574871	−340.875864	−341.125860	−341.332716	−341.505466
$\sigma^{5,3}\pi_+^{1,1}\pi_-^{2,1}\delta_-^{1,0}\phi_-^{1,0}$	−338.662002	−339.182016	−339.643318	−340.049412	−340.405544	−340.718292	−340.994953
$\sigma^{6,3}\pi_+^{1,1}\pi_-^{2,1}\delta_-^{1,0}$	−339.873148	−340.225065	−340.496375	−340.750496	−340.965442	−341.142341	−341.288487

Table S456: Total energies in E_h for the P atom in the def2-TZVP basis set in fully uncontracted form, employing the real-orbital approximation.

	$0.00B_0$	$0.10B_0$	$0.20B_0$	$0.30B_0$	$0.40B_0$	$0.50B_0$	$0.60B_0$
$\sigma^{5,4}\pi_+^{2,1}\pi_-^{2,1}$	−340.711265	−340.836282	−340.912937	−340.945436	−340.939153	−340.899263	−340.830058
$\sigma^{4,5}\pi_+^{2,1}\pi_-^{2,1}$	−340.683966	−340.708891	−340.685307	−340.617503	−340.510922	−340.370771	−340.201336
$\sigma^{5,4}\pi_+^{1,2}\pi_-^{2,1}$	−340.669195	−340.693844	−340.669477	−340.600518	−340.492619	−340.351188	−340.180631
$\sigma^{5,4}\pi_+^{1,1}\pi_-^{2,2}$	−340.623506	−340.747700	−340.822063	−340.851291	−340.841412	−340.798162	−340.726113
$\sigma^{5,4}\pi_+^{1,1}\pi_-^{2,1}\delta_-^{1,0}$	−340.247212	−340.521569	−340.745757	−340.922803	−341.056876	−341.152563	−341.214460
$\sigma^{5,3}\pi_+^{2,1}\pi_-^{2,1}\delta_-^{1,0}$	−340.168201	−340.491265	−340.761727	−340.982974	−341.159552	−341.296261	−341.397701
$\sigma^{5,3}\pi_+^{1,1}\pi_-^{3,1}\delta_-^{1,0}$	−339.689755	−340.114147	−340.488187	−340.814247	−341.095716	−341.336524	−341.540879
$\sigma^{5,3}\pi_+^{1,1}\pi_-^{2,1}\delta_-^{1,0}\phi_-^{1,0}$	−338.600179	−339.123710	−339.595195	−340.017027	−340.392494	−340.725213	−341.018843
$\sigma^{6,3}\pi_+^{1,1}\pi_-^{2,1}\delta_-^{1,0}$	−339.690898	−340.066041	−340.393237	−340.675959	−340.917589	−341.121477	−341.291112

Table S457: Total energies in E_h for the S atom in the cc-pVDZ basis set in fully uncontracted form, employing the real-orbital approximation.

	$0.00B_0$	$0.10B_0$	$0.20B_0$	$0.30B_0$	$0.40B_0$	$0.50B_0$	$0.60B_0$
$\sigma^{5,5}\pi_+^{2,1}\pi_-^{2,1}$	−397.497141	−397.575054	−397.609368	−397.601737	−397.554718	−397.471493	−397.355546
$\sigma^{5,4}\pi_+^{2,1}\pi_-^{2,2}$	−397.494329	−397.619527	−397.695828	−397.725288	−397.711096	−397.657249	−397.568123
$\sigma^{5,4}\pi_+^{2,2}\pi_-^{2,1}$	−397.494329	−397.519527	−397.495828	−397.425288	−397.311096	−397.157249	−396.968123
$\sigma^{5,4}\pi_+^{2,1}\pi_-^{2,1}\delta_-^{1,0}$	−396.833767	−397.110574	−397.341527	−397.528162	−397.672838	−397.778472	−397.848213
$\sigma^{5,4}\pi_+^{1,1}\pi_-^{2,2}\delta_-^{1,0}$	−396.647139	−396.923214	−397.152014	−397.335201	−397.475358	−397.575711	−397.639789
$\sigma^{5,3}\pi_+^{2,1}\pi_-^{3,1}\delta_-^{1,0}$	−395.978810	−396.399904	−396.763475	−397.070363	−397.410513	−397.672383	−397.898326
$\sigma^{5,4}\pi_+^{1,1}\pi_-^{3,1}\delta_-^{1,0}$	−396.061475	−396.433892	−396.751336	−397.023661	−397.281257	−397.501805	−397.687496
$\sigma^{5,3}\pi_+^{2,1}\pi_-^{2,1}\delta_-^{1,0}\phi_-^{1,0}$							
$\sigma^{5,3}\pi_+^{1,1}\pi_-^{3,1}\delta_-^{1,0}\phi_-^{1,0}$							

Table S458: Total energies in E_h for the S atom in the cc-pVTZ basis set in fully uncontracted form, employing the real-orbital approximation.

	$0.00B_0$	$0.10B_0$	$0.20B_0$	$0.30B_0$	$0.40B_0$	$0.50B_0$	$0.60B_0$
$\sigma^{5,5}\pi_+^{2,1}\pi_-^{2,1}$	−397.509395	−397.587118	−397.621154	−397.613895	−397.568714	−397.489333	−397.379318
$\sigma^{5,4}\pi_+^{2,1}\pi_-^{2,2}$	−397.504704	−397.629685	−397.705770	−397.736100	−397.725065	−397.677377	−397.597303
$\sigma^{5,4}\pi_+^{2,2}\pi_-^{2,1}$	−397.504704	−397.529685	−397.505770	−397.436100	−397.325065	−397.177377	−396.997303
$\sigma^{5,4}\pi_+^{2,1}\pi_-^{2,1}\delta_-^{1,0}$	−397.058835	−397.333923	−397.559981	−397.739219	−397.874793	−397.970278	−398.029259
$\sigma^{5,4}\pi_+^{1,1}\pi_-^{2,2}\delta_-^{1,0}$	−396.881831	−397.156182	−397.380147	−397.556249	−397.688057	−397.779523	−397.834473
$\sigma^{5,3}\pi_+^{2,1}\pi_-^{3,1}\delta_-^{1,0}$	−396.333657	−396.749275	−397.174987	−397.504159	−397.788687	−398.031506	−398.235690
$\sigma^{5,4}\pi_+^{1,1}\pi_-^{3,1}\delta_-^{1,0}$	−396.453866	−396.829984	−397.158891	−397.442140	−397.682037	−397.881346	−398.043053
$\sigma^{5,3}\pi_+^{2,1}\pi_-^{2,1}\delta_-^{1,0}\phi_-^{1,0}$	−395.042455	−395.566922	−396.040972	−396.466395	−396.845739	−397.181881	−397.477710
$\sigma^{5,3}\pi_+^{1,1}\pi_-^{3,1}\delta_-^{1,0}\phi_-^{1,0}$	−394.386451	−395.001886	−395.628066	−396.156829	−396.639767	−397.079098	−397.477257

Table S459: Total energies in E_h for the S atom in the cc-pVQZ basis set in fully uncontracted form, employing the real-orbital approximation.

	$0.00B_0$	$0.10B_0$	$0.20B_0$	$0.30B_0$	$0.40B_0$	$0.50B_0$	$0.60B_0$
$\sigma^{5,5}\pi_+^{2,1}\pi_-^{2,1}$	−397.512655	−397.590306	−397.624329	−397.617549	−397.573665	−397.496455	−397.389340
$\sigma^{5,4}\pi_+^{2,1}\pi_-^{2,2}$	−397.507685	−397.632615	−397.708851	−397.740135	−397.731147	−397.686316	−397.609325
$\sigma^{5,4}\pi_+^{2,2}\pi_-^{2,1}$	−397.507685	−397.532615	−397.508851	−397.440135	−397.331147	−397.186316	−397.009325
$\sigma^{5,4}\pi_+^{2,1}\pi_-^{2,1}\delta_-^{1,0}$	−397.128530	−397.401995	−397.623445	−397.795763	−397.923016	−398.009852	−398.061110
$\sigma^{5,4}\pi_+^{1,1}\pi_-^{2,2}\delta_-^{1,0}$	−396.963149	−397.235808	−397.454973	−397.623836	−397.746776	−397.828653	−397.874449
$\sigma^{5,3}\pi_+^{2,1}\pi_-^{3,1}\delta_-^{1,0}$	−396.488650	−396.911638	−397.281481	−397.600653	−397.872817	−398.102403	−398.294260
$\sigma^{5,4}\pi_+^{1,1}\pi_-^{3,1}\delta_-^{1,0}$	−396.587959	−396.961449	−397.282748	−397.554199	−397.779352	−397.962609	−398.108921
$\sigma^{5,3}\pi_+^{2,1}\pi_-^{2,1}\delta_-^{1,0}\phi_-^{1,0}$	−395.676856	−396.197411	−396.659877	−397.066482	−397.420481	−397.725744	−397.986432
$\sigma^{5,3}\pi_+^{1,1}\pi_-^{3,1}\delta_-^{1,0}\phi_-^{1,0}$	−395.103030	−395.723503	−396.285605	−396.791307	−397.243653	−397.646500	−398.004237

Table S460: Total energies in E_h for the S atom in the cc-pV5Z basis set in fully uncontracted form, employing the real-orbital approximation.

	$0.00B_0$	$0.10B_0$	$0.20B_0$	$0.30B_0$	$0.40B_0$	$0.50B_0$	$0.60B_0$
$\sigma^{5,5}\pi_+^{2,1}\pi_-^{2,1}$	−397.513183	−397.590814	−397.624888	−397.618431	−397.575235	−397.499039	−397.393176
$\sigma^{5,4}\pi_+^{2,1}\pi_-^{2,2}$	−397.508153	−397.633077	−397.709446	−397.741202	−397.732957	−397.688895	−397.612554
$\sigma^{5,4}\pi_+^{2,2}\pi_-^{2,1}$	−397.508153	−397.533077	−397.509446	−397.441202	−397.332957	−397.188895	−397.012554
$\sigma^{5,4}\pi_+^{2,1}\pi_-^{2,1}\delta_-^{1,0}$	−397.131599	−397.405311	−397.627564	−397.801351	−397.930810	−398.020608	−398.075572
$\sigma^{5,4}\pi_+^{1,1}\pi_-^{2,2}\delta_-^{1,0}$	−396.965081	−397.237970	−397.457939	−397.628370	−397.753715	−397.838807	−397.888594
$\sigma^{5,3}\pi_+^{2,1}\pi_-^{3,1}\delta_-^{1,0}$	−396.492636	−396.900806	−397.227058	−397.477015	−397.890509	−398.124707	−398.322409
$\sigma^{5,4}\pi_+^{1,1}\pi_-^{3,1}\delta_-^{1,0}$	−396.612599	−396.971290	−397.292015	−397.565821	−397.794508	−397.982602	−398.135107
$\sigma^{5,3}\pi_+^{2,1}\pi_-^{2,1}\delta_-^{1,0}\phi_-^{1,0}$	−395.919848	−396.437824	−396.892619	−397.286666	−397.623595	−397.907874	−398.144532
$\sigma^{5,3}\pi_+^{1,1}\pi_-^{3,1}\delta_-^{1,0}\phi_-^{1,0}$	−395.290670	−395.893499	−396.403820	−396.827603	−397.452078	−397.836422	−398.173306

Table S461: Total energies in E_h for the S atom in the aug-cc-pVDZ basis set in fully uncontracted form, employing the real-orbital approximation.

	$0.00B_0$	$0.10B_0$	$0.20B_0$	$0.30B_0$	$0.40B_0$	$0.50B_0$	$0.60B_0$
$\sigma^{5,5}\pi_+^{2,1}\pi_-^{2,1}$	−397.499053	−397.576709	−397.610675	−397.603353	−397.557696	−397.476850	−397.364018
$\sigma^{5,4}\pi_+^{2,1}\pi_-^{2,2}$	−397.495986	−397.620675	−397.696227	−397.725864	−397.713178	−397.661940	−397.576082
$\sigma^{5,4}\pi_+^{2,2}\pi_-^{2,1}$	−397.495986	−397.520675	−397.496227	−397.425864	−397.313178	−397.161940	−396.976082
$\sigma^{5,4}\pi_+^{2,1}\pi_-^{2,1}\delta_-^{1,0}$	−397.130201	−397.401508	−397.616544	−397.778409	−397.891780	−397.962571	−397.997346
$\sigma^{5,4}\pi_+^{1,1}\pi_-^{2,2}\delta_-^{1,0}$	−396.975450	−397.245808	−397.458109	−397.615568	−397.722834	−397.785849	−397.811512
$\sigma^{5,3}\pi_+^{2,1}\pi_-^{3,1}\delta_-^{1,0}$	−396.521454	−396.913902	−397.249717	−397.554456	−397.807894	−398.016111	−398.185793
$\sigma^{5,4}\pi_+^{1,1}\pi_-^{3,1}\delta_-^{1,0}$	−396.661952	−397.004465	−397.271996	−397.527711	−397.732469	−397.892518	−398.014964
$\sigma^{5,3}\pi_+^{2,1}\pi_-^{2,1}\delta_-^{1,0}\phi_-^{1,0}$							
$\sigma^{5,3}\pi_+^{1,1}\pi_-^{3,1}\delta_-^{1,0}\phi_-^{1,0}$							

Table S462: Total energies in E_h for the S atom in the aug-cc-pVTZ basis set in fully uncontracted form, employing the real-orbital approximation.

	$0.00B_0$	$0.10B_0$	$0.20B_0$	$0.30B_0$	$0.40B_0$	$0.50B_0$	$0.60B_0$
$\sigma^{5,5}\pi_+^{2,1}\pi_-^{2,1}$	−397.509991	−397.587580	−397.621559	−397.614927	−397.571456	−397.494993	−397.389028
$\sigma^{5,4}\pi_+^{2,1}\pi_-^{2,2}$	−397.505087	−397.629961	−397.706243	−397.737722	−397.728884	−397.684217	−397.607711
$\sigma^{5,4}\pi_+^{2,2}\pi_-^{2,1}$	−397.505087	−397.529961	−397.506243	−397.437722	−397.328884	−397.184217	−397.007711
$\sigma^{5,4}\pi_+^{2,1}\pi_-^{2,1}\delta_-^{1,0}$	−397.187381	−397.454808	−397.660150	−397.811992	−397.921661	−397.998279	−398.046371
$\sigma^{5,4}\pi_+^{1,1}\pi_-^{2,2}\delta_-^{1,0}$	−397.046530	−397.312110	−397.511837	−397.654491	−397.753021	−397.819222	−397.858985
$\sigma^{5,3}\pi_+^{2,1}\pi_-^{3,1}\delta_-^{1,0}$	−396.555630	−396.971627	−397.322901	−397.618195	−397.868434	−398.082800	−398.266916
$\sigma^{5,4}\pi_+^{1,1}\pi_-^{3,1}\delta_-^{1,0}$	−396.712638	−397.050168	−397.348009	−397.588567	−397.784663	−397.947331	−398.082938
$\sigma^{5,3}\pi_+^{2,1}\pi_-^{2,1}\delta_-^{1,0}\phi_-^{1,0}$	−395.999773	−396.512542	−396.953469	−397.329369	−397.648327	−397.916815	−398.139268
$\sigma^{5,3}\pi_+^{1,1}\pi_-^{3,1}\delta_-^{1,0}\phi_-^{1,0}$	−395.428157	−396.039298	−396.575633	−397.044775	−397.455995	−397.817094	−398.133458

Table S463: Total energies in E_h for the S atom in the aug-cc-pVQZ basis set in fully uncontracted form, employing the real-orbital approximation.

	$0.00B_0$	$0.10B_0$	$0.20B_0$	$0.30B_0$	$0.40B_0$	$0.50B_0$	$0.60B_0$
$\sigma^{5,5}\pi_+^{2,1}\pi_-^{2,1}$	−397.512766	−397.590380	−397.624529	−397.618385	−397.575737	−397.500191	−397.394877
$\sigma^{5,4}\pi_+^{2,1}\pi_-^{2,2}$	−397.507765	−397.632675	−397.709144	−397.741143	−397.733186	−397.689433	−397.613375
$\sigma^{5,4}\pi_+^{2,2}\pi_-^{2,1}$	−397.507765	−397.532675	−397.509144	−397.441143	−397.333186	−397.189433	−397.013375
$\sigma^{5,4}\pi_+^{2,1}\pi_-^{2,1}\delta_-^{1,0}$	−397.208522	−397.472117	−397.670232	−397.819849	−397.933484	−398.014854	−398.065507
$\sigma^{5,4}\pi_+^{1,1}\pi_-^{2,2}\delta_-^{1,0}$	−397.075429	−397.335946	−397.524664	−397.661179	−397.763496	−397.836478	−397.880338
$\sigma^{5,3}\pi_+^{2,1}\pi_-^{3,1}\delta_-^{1,0}$	−396.584570	−396.969625	−397.341287	−397.636507	−397.891980	−398.113560	−398.304129
$\sigma^{5,4}\pi_+^{1,1}\pi_-^{3,1}\delta_-^{1,0}$	−396.728795	−397.075815	−397.365883	−397.603950	−397.805412	−397.976708	−398.119951
$\sigma^{5,3}\pi_+^{2,1}\pi_-^{2,1}\delta_-^{1,0}\phi_-^{1,0}$	−396.219211	−396.724477	−397.145340	−397.492283	−397.774781	−398.001869	−398.185062
$\sigma^{5,3}\pi_+^{1,1}\pi_-^{3,1}\delta_-^{1,0}\phi_-^{1,0}$	−395.583731	−396.161693	−396.767298	−397.210144	−397.588992	−397.914042	−398.197226

Table S464: Total energies in E_h for the S atom in the aug-cc-pV5Z basis set in fully uncontracted form, employing the real-orbital approximation.

	$0.00B_0$	$0.10B_0$	$0.20B_0$	$0.30B_0$	$0.40B_0$	$0.50B_0$	$0.60B_0$
$\sigma^{5,5}\pi_+^{2,1}\pi_-^{2,1}$	−397.513230	−397.590853	−397.625048	−397.619004	−397.576452	−397.500931	−397.395596
$\sigma^{5,4}\pi_+^{2,1}\pi_-^{2,2}$	−397.508182	−397.633103	−397.709625	−397.741743	−397.733898	−397.690132	−397.613950
$\sigma^{5,4}\pi_+^{2,2}\pi_-^{2,1}$	−397.508182	−397.533103	−397.509625	−397.441743	−397.333898	−397.190132	−397.013950
$\sigma^{5,4}\pi_+^{2,1}\pi_-^{2,1}\delta_-^{1,0}$	−397.209086	−397.473796	−397.674485	−397.826518	−397.942087	−398.025572	−398.078807
$\sigma^{5,4}\pi_+^{1,1}\pi_-^{2,2}\delta_-^{1,0}$	−397.073697	−397.335442	−397.527226	−397.666827	−397.771357	−397.846550	−397.893122
$\sigma^{5,3}\pi_+^{2,1}\pi_-^{3,1}\delta_-^{1,0}$	−396.593636	−396.977725	−397.354910	−397.653318	−397.912072	−398.137317	−398.332109
$\sigma^{5,4}\pi_+^{1,1}\pi_-^{3,1}\delta_-^{1,0}$	−396.734841	−397.083226	−397.376668	−397.618518	−397.823648	−397.998941	−398.146802
$\sigma^{5,3}\pi_+^{2,1}\pi_-^{2,1}\delta_-^{1,0}\phi_-^{1,0}$	−396.289134	−396.790995	−397.201609	−397.532697	−397.798504	−398.016072	−398.202514
$\sigma^{5,3}\pi_+^{1,1}\pi_-^{3,1}\delta_-^{1,0}\phi_-^{1,0}$	−395.645289	−396.220083	−396.832145	−397.260848	−397.625317	−397.943432	−398.232008

Table S465: Total energies in E_h for the S atom in the HGBSP1-5 basis set in fully uncontracted form, employing the real-orbital approximation.

	$0.00B_0$	$0.10B_0$	$0.20B_0$	$0.30B_0$	$0.40B_0$	$0.50B_0$	$0.60B_0$
$\sigma^{5,5}\pi_+^{2,1}\pi_-^{2,1}$	−397.501837	−397.579614	−397.614025	−397.607696	−397.563824	−397.485625	−397.376098
$\sigma^{5,4}\pi_+^{2,1}\pi_-^{2,2}$	−397.498471	−397.623268	−397.699241	−397.729967	−397.719488	−397.671720	−397.590217
$\sigma^{5,4}\pi_+^{2,2}\pi_-^{2,1}$	−397.498471	−397.523268	−397.499241	−397.429967	−397.319488	−397.171720	−396.990217
$\sigma^{5,4}\pi_+^{2,1}\pi_-^{2,1}\delta_-^{1,0}$	−397.229202	−397.474096	−397.666547	−397.818679	−397.933514	−398.013809	−398.062743
$\sigma^{5,4}\pi_+^{1,1}\pi_-^{2,2}\delta_-^{1,0}$	−397.112607	−397.343335	−397.518284	−397.656280	−397.759750	−397.830172	−397.870199
$\sigma^{5,3}\pi_+^{2,1}\pi_-^{3,1}\delta_-^{1,0}$	−396.592746	−397.002253	−397.348303	−397.646164	−397.903114	−398.123702	−398.311687
$\sigma^{5,4}\pi_+^{1,1}\pi_-^{3,1}\delta_-^{1,0}$	−396.733640	−397.083114	−397.369469	−397.610661	−397.814494	−397.985156	−398.126120
$\sigma^{5,3}\pi_+^{2,1}\pi_-^{2,1}\delta_-^{1,0}\phi_-^{1,0}$							
$\sigma^{5,3}\pi_+^{1,1}\pi_-^{3,1}\delta_-^{1,0}\phi_-^{1,0}$							

Table S466: Total energies in E_h for the S atom in the HGBSP1-7 basis set in fully uncontracted form, employing the real-orbital approximation.

	$0.00B_0$	$0.10B_0$	$0.20B_0$	$0.30B_0$	$0.40B_0$	$0.50B_0$	$0.60B_0$
$\sigma^{5,5}\pi_+^{2,1}\pi_-^{2,1}$	−397.510916	−397.588696	−397.623121	−397.616837	−397.573058	−397.494988	−397.385589
$\sigma^{5,4}\pi_+^{2,1}\pi_-^{2,2}$	−397.507551	−397.632350	−397.708338	−397.739128	−397.728799	−397.681200	−397.599778
$\sigma^{5,4}\pi_+^{2,2}\pi_-^{2,1}$	−397.507551	−397.532350	−397.508338	−397.439128	−397.328799	−397.181200	−396.999778
$\sigma^{5,4}\pi_+^{2,1}\pi_-^{2,1}\delta_-^{1,0}$	−397.238340	−397.483253	−397.675704	−397.827891	−397.942711	−398.023238	−398.072397
$\sigma^{5,4}\pi_+^{1,1}\pi_-^{2,2}\delta_-^{1,0}$	−397.121747	−397.352544	−397.527439	−397.665620	−397.768968	−397.839669	−397.880062
$\sigma^{5,3}\pi_+^{2,1}\pi_-^{3,1}\delta_-^{1,0}$	−396.601844	−397.011343	−397.357408	−397.655292	−397.912232	−398.132977	−398.321192
$\sigma^{5,4}\pi_+^{1,1}\pi_-^{3,1}\delta_-^{1,0}$	−396.742953	−397.092272	−397.378649	−397.619903	−397.823673	−397.994527	−398.135831
$\sigma^{5,3}\pi_+^{2,1}\pi_-^{2,1}\delta_-^{1,0}\phi_-^{1,0}$							
$\sigma^{5,3}\pi_+^{1,1}\pi_-^{3,1}\delta_-^{1,0}\phi_-^{1,0}$							

Table S467: Total energies in E_h for the S atom in the HGBSP1-9 basis set in fully uncontracted form, employing the real-orbital approximation.

	$0.00B_0$	$0.10B_0$	$0.20B_0$	$0.30B_0$	$0.40B_0$	$0.50B_0$	$0.60B_0$
$\sigma^{5,5}\pi_+^{2,1}\pi_-^{2,1}$	−397.511031	−397.588812	−397.623238	−397.616957	−397.573184	−397.495124	−397.385741
$\sigma^{5,4}\pi_+^{2,1}\pi_-^{2,2}$	−397.507666	−397.632466	−397.708455	−397.739249	−397.728924	−397.681339	−397.599950
$\sigma^{5,4}\pi_+^{2,2}\pi_-^{2,1}$	−397.507666	−397.532466	−397.508455	−397.439249	−397.328924	−397.181339	−396.999950
$\sigma^{5,4}\pi_+^{2,1}\pi_-^{2,1}\delta_-^{1,0}$	−397.238457	−397.483370	−397.675823	−397.828020	−397.942844	−398.023372	−398.072574
$\sigma^{5,4}\pi_+^{1,1}\pi_-^{2,2}\delta_-^{1,0}$	−397.121864	−397.352663	−397.527568	−397.665753	−397.769116	−397.839799	−397.880238
$\sigma^{5,3}\pi_+^{2,1}\pi_-^{3,1}\delta_-^{1,0}$	−396.601959	−397.011457	−397.357523	−397.655413	−397.912359	−398.133104	−398.321347
$\sigma^{5,4}\pi_+^{1,1}\pi_-^{3,1}\delta_-^{1,0}$	−396.743070	−397.092388	−397.378767	−397.620030	−397.823813	−397.994659	−398.135990
$\sigma^{5,3}\pi_+^{2,1}\pi_-^{2,1}\delta_-^{1,0}\phi_-^{1,0}$							
$\sigma^{5,3}\pi_+^{1,1}\pi_-^{3,1}\delta_-^{1,0}\phi_-^{1,0}$							

Table S468: Total energies in E_h for the S atom in the HGBSP2-5 basis set in fully uncontracted form, employing the real-orbital approximation.

	$0.00B_0$	$0.10B_0$	$0.20B_0$	$0.30B_0$	$0.40B_0$	$0.50B_0$	$0.60B_0$
$\sigma^{5,5}\pi_+^{2,1}\pi_-^{2,1}$	−397.504194	−397.581819	−397.616029	−397.609971	−397.567333	−397.491700	−397.386300
$\sigma^{5,4}\pi_+^{2,1}\pi_-^{2,2}$	−397.499120	−397.624045	−397.700585	−397.732675	−397.724681	−397.680743	−397.604535
$\sigma^{5,4}\pi_+^{2,2}\pi_-^{2,1}$	−397.499120	−397.524045	−397.500585	−397.432675	−397.324681	−397.180743	−397.004535
$\sigma^{5,4}\pi_+^{2,1}\pi_-^{2,1}\delta_-^{1,0}$	−397.229211	−397.474141	−397.666727	−397.819361	−397.935371	−398.017757	−398.069857
$\sigma^{5,4}\pi_+^{1,1}\pi_-^{2,2}\delta_-^{1,0}$	−397.113594	−397.344457	−397.519866	−397.658989	−397.764554	−397.838256	−397.882877
$\sigma^{5,3}\pi_+^{2,1}\pi_-^{3,1}\delta_-^{1,0}$	−396.593569	−397.003167	−397.349520	−397.647993	−397.906119	−398.128711	−398.319753
$\sigma^{5,4}\pi_+^{1,1}\pi_-^{3,1}\delta_-^{1,0}$	−396.733755	−397.084044	−397.370702	−397.612523	−397.817457	−397.989872	−398.133394
$\sigma^{5,3}\pi_+^{2,1}\pi_-^{2,1}\delta_-^{1,0}\phi_-^{1,0}$	−396.529229	−396.936635	−397.262376	−397.546232	−397.791461	−398.008486	−398.198541
$\sigma^{5,3}\pi_+^{1,1}\pi_-^{3,1}\delta_-^{1,0}\phi_-^{1,0}$	−395.954694	−396.463611	−396.890913	−397.275075	−397.620222	−397.936736	−398.226168

Table S469: Total energies in E_h for the S atom in the HGBSP2-7 basis set in fully uncontracted form, employing the real-orbital approximation.

	$0.00B_0$	$0.10B_0$	$0.20B_0$	$0.30B_0$	$0.40B_0$	$0.50B_0$	$0.60B_0$
$\sigma^{5,5}\pi_+^{2,1}\pi_-^{2,1}$	−397.513272	−397.590901	−397.625125	−397.619112	−397.576560	−397.501023	−397.395704
$\sigma^{5,4}\pi_+^{2,1}\pi_-^{2,2}$	−397.508199	−397.633126	−397.709681	−397.741833	−397.733972	−397.690173	−397.614029
$\sigma^{5,4}\pi_+^{2,2}\pi_-^{2,1}$	−397.508199	−397.533126	−397.509681	−397.441833	−397.333972	−397.190173	−397.014029
$\sigma^{5,4}\pi_+^{2,1}\pi_-^{2,1}\delta_-^{1,0}$	−397.238349	−397.483298	−397.675884	−397.828570	−397.944561	−398.027169	−398.079486
$\sigma^{5,4}\pi_+^{1,1}\pi_-^{2,2}\delta_-^{1,0}$	−397.122734	−397.353666	−397.529021	−397.668326	−397.773761	−397.847725	−397.892690
$\sigma^{5,3}\pi_+^{2,1}\pi_-^{3,1}\delta_-^{1,0}$	−396.602666	−397.012255	−397.358625	−397.657118	−397.915232	−398.137980	−398.329252
$\sigma^{5,4}\pi_+^{1,1}\pi_-^{3,1}\delta_-^{1,0}$	−396.743067	−397.093202	−397.379882	−397.621762	−397.826630	−397.999236	−398.143093
$\sigma^{5,3}\pi_+^{2,1}\pi_-^{2,1}\delta_-^{1,0}\phi_-^{1,0}$	−396.538357	−396.945834	−397.272492	−397.555585	−397.802974	−398.019239	−398.207982
$\sigma^{5,3}\pi_+^{1,1}\pi_-^{3,1}\delta_-^{1,0}\phi_-^{1,0}$	−395.963795	−396.472761	−396.900858	−397.284445	−397.631518	−397.947245	−398.235599

Table S470: Total energies in E_h for the S atom in the HGBSP2-9 basis set in fully uncontracted form, employing the real-orbital approximation.

	$0.00B_0$	$0.10B_0$	$0.20B_0$	$0.30B_0$	$0.40B_0$	$0.50B_0$	$0.60B_0$
$\sigma^{5,5}\pi_+^{2,1}\pi_-^{2,1}$	−397.513388	−397.591017	−397.625242	−397.619232	−397.576684	−397.501158	−397.395851
$\sigma^{5,4}\pi_+^{2,1}\pi_-^{2,2}$	−397.508315	−397.633242	−397.709799	−397.741954	−397.734098	−397.690311	−397.614191
$\sigma^{5,4}\pi_+^{2,2}\pi_-^{2,1}$	−397.508315	−397.533242	−397.509799	−397.441954	−397.334098	−397.190311	−397.014191
$\sigma^{5,4}\pi_+^{2,1}\pi_-^{2,1}\delta_-^{1,0}$	−397.238466	−397.483414	−397.676003	−397.828699	−397.944694	−398.027303	−398.079658
$\sigma^{5,4}\pi_+^{1,1}\pi_-^{2,2}\delta_-^{1,0}$	−397.122850	−397.353785	−397.529150	−397.668458	−397.773909	−397.847854	−397.892860
$\sigma^{5,3}\pi_+^{2,1}\pi_-^{3,1}\delta_-^{1,0}$	−396.602781	−397.012370	−397.358739	−397.657240	−397.915358	−398.138107	−398.329403
$\sigma^{5,4}\pi_+^{1,1}\pi_-^{3,1}\delta_-^{1,0}$	−396.743184	−397.093318	−397.380000	−397.621889	−397.826770	−397.999368	−398.143249
$\sigma^{5,3}\pi_+^{2,1}\pi_-^{2,1}\delta_-^{1,0}\phi_-^{1,0}$	−396.538472	−396.945979	−397.272674	−397.555742	−397.803241	−398.019643	−398.208175
$\sigma^{5,3}\pi_+^{1,1}\pi_-^{3,1}\delta_-^{1,0}\phi_-^{1,0}$	−395.963910	−396.472905	−396.901038	−397.284592	−397.631782	−397.947615	−398.235777

Table S471: Total energies in E_h for the S atom in the HGBSP3-5 basis set in fully uncontracted form, employing the real-orbital approximation.

	$0.00B_0$	$0.10B_0$	$0.20B_0$	$0.30B_0$	$0.40B_0$	$0.50B_0$	$0.60B_0$
$\sigma^{5,5}\pi_+^{2,1}\pi_-^{2,1}$	−397.504194	−397.581820	−397.616030	−397.609976	−397.567351	−397.491750	−397.386419
$\sigma^{5,4}\pi_+^{2,1}\pi_-^{2,2}$	−397.499120	−397.624045	−397.700585	−397.732675	−397.724684	−397.680757	−397.604580
$\sigma^{5,4}\pi_+^{2,2}\pi_-^{2,1}$	−397.499120	−397.524045	−397.500585	−397.432675	−397.324684	−397.180757	−397.004580
$\sigma^{5,4}\pi_+^{2,1}\pi_-^{2,1}\delta_-^{1,0}$	−397.229211	−397.474397	−397.667382	−397.820499	−397.937094	−398.020202	−398.073124
$\sigma^{5,4}\pi_+^{1,1}\pi_-^{2,2}\delta_-^{1,0}$	−397.113596	−397.345009	−397.521150	−397.660973	−397.767255	−397.841812	−397.887364
$\sigma^{5,3}\pi_+^{2,1}\pi_-^{3,1}\delta_-^{1,0}$	−396.593643	−397.003552	−397.351067	−397.651414	−397.911994	−398.137628	−398.332244
$\sigma^{5,4}\pi_+^{1,1}\pi_-^{3,1}\delta_-^{1,0}$	−396.733763	−397.084463	−397.372546	−397.616540	−397.824153	−397.999771	−398.146964
$\sigma^{5,3}\pi_+^{2,1}\pi_-^{2,1}\delta_-^{1,0}\phi_-^{1,0}$	−396.529233	−396.936662	−397.262544	−397.546703	−397.792401	−398.010047	−398.200847
$\sigma^{5,3}\pi_+^{1,1}\pi_-^{3,1}\delta_-^{1,0}\phi_-^{1,0}$	−395.954765	−396.463796	−396.891645	−397.277014	−397.624112	−397.943270	−398.235916

Table S472: Total energies in E_h for the S atom in the HGBSP3-7 basis set in fully uncontracted form, employing the real-orbital approximation.

	$0.00B_0$	$0.10B_0$	$0.20B_0$	$0.30B_0$	$0.40B_0$	$0.50B_0$	$0.60B_0$
$\sigma^{5,5}\pi_+^{2,1}\pi_-^{2,1}$	−397.513273	−397.590901	−397.625127	−397.619118	−397.576578	−397.501075	−397.395823
$\sigma^{5,4}\pi_+^{2,1}\pi_-^{2,2}$	−397.508199	−397.633127	−397.709681	−397.741834	−397.733976	−397.690188	−397.614073
$\sigma^{5,4}\pi_+^{2,2}\pi_-^{2,1}$	−397.508199	−397.533127	−397.509681	−397.441834	−397.333976	−397.190188	−397.014073
$\sigma^{5,4}\pi_+^{2,1}\pi_-^{2,1}\delta_-^{1,0}$	−397.238350	−397.483551	−397.676533	−397.829705	−397.946286	−398.029586	−398.082710
$\sigma^{5,4}\pi_+^{1,1}\pi_-^{2,2}\delta_-^{1,0}$	−397.122736	−397.354208	−397.530296	−397.670295	−397.776474	−397.851243	−397.897106
$\sigma^{5,3}\pi_+^{2,1}\pi_-^{3,1}\delta_-^{1,0}$	−396.602740	−397.012641	−397.360168	−397.660527	−397.921113	−398.146872	−398.341652
$\sigma^{5,4}\pi_+^{1,1}\pi_-^{3,1}\delta_-^{1,0}$	−396.743076	−397.093620	−397.381720	−397.625755	−397.833336	−398.009109	−398.156546
$\sigma^{5,3}\pi_+^{2,1}\pi_-^{2,1}\delta_-^{1,0}\phi_-^{1,0}$	−396.538361	−396.945862	−397.272659	−397.556059	−397.803912	−398.020786	−398.210272
$\sigma^{5,3}\pi_+^{1,1}\pi_-^{3,1}\delta_-^{1,0}\phi_-^{1,0}$	−395.963867	−396.472946	−396.901585	−397.286391	−397.635399	−397.953732	−398.245291

Table S473: Total energies in E_h for the S atom in the HGBSP3-9 basis set in fully uncontracted form, employing the real-orbital approximation.

	$0.00B_0$	$0.10B_0$	$0.20B_0$	$0.30B_0$	$0.40B_0$	$0.50B_0$	$0.60B_0$
$\sigma^{5,5}\pi_+^{2,1}\pi_-^{2,1}$	−397.513389	−397.591017	−397.625244	−397.619237	−397.576703	−397.501209	−397.395971
$\sigma^{5,4}\pi_+^{2,1}\pi_-^{2,2}$	−397.508315	−397.633243	−397.709799	−397.741955	−397.734102	−397.690327	−397.614235
$\sigma^{5,4}\pi_+^{2,2}\pi_-^{2,1}$	−397.508315	−397.533243	−397.509799	−397.441955	−397.334102	−397.190327	−397.014235
$\sigma^{5,4}\pi_+^{2,1}\pi_-^{2,1}\delta_-^{1,0}$	−397.238467	−397.483668	−397.676652	−397.829833	−397.946418	−398.029720	−398.082878
$\sigma^{5,4}\pi_+^{1,1}\pi_-^{2,2}\delta_-^{1,0}$	−397.122852	−397.354327	−397.530423	−397.670425	−397.776618	−397.851374	−397.897273
$\sigma^{5,3}\pi_+^{2,1}\pi_-^{3,1}\delta_-^{1,0}$	−396.602854	−397.012755	−397.360282	−397.660647	−397.921235	−398.146997	−398.341801
$\sigma^{5,4}\pi_+^{1,1}\pi_-^{3,1}\delta_-^{1,0}$	−396.743192	−397.093736	−397.381837	−397.625881	−397.833468	−398.009238	−398.156700
$\sigma^{5,3}\pi_+^{2,1}\pi_-^{2,1}\delta_-^{1,0}\phi_-^{1,0}$	−396.538477	−396.946007	−397.272841	−397.556215	−397.804179	−398.021190	−398.210463
$\sigma^{5,3}\pi_+^{1,1}\pi_-^{3,1}\delta_-^{1,0}\phi_-^{1,0}$	−395.963982	−396.473090	−396.901765	−397.286536	−397.635663	−397.954099	−398.245464

Table S474: Total energies in E_h for the S atom in the AHGBSP1-5 basis set in fully uncontracted form, employing the real-orbital approximation.

	$0.00B_0$	$0.10B_0$	$0.20B_0$	$0.30B_0$	$0.40B_0$	$0.50B_0$	$0.60B_0$
$\sigma^{5,5}\pi_+^{2,1}\pi_-^{2,1}$	−397.501841	−397.579619	−397.614033	−397.607713	−397.563859	−397.485686	−397.376187
$\sigma^{5,4}\pi_+^{2,1}\pi_-^{2,2}$	−397.498476	−397.623273	−397.699248	−397.729986	−397.719533	−397.671797	−397.590321
$\sigma^{5,4}\pi_+^{2,2}\pi_-^{2,1}$	−397.498476	−397.523273	−397.499248	−397.429986	−397.319533	−397.171797	−396.990321
$\sigma^{5,4}\pi_+^{2,1}\pi_-^{2,1}\delta_-^{1,0}$	−397.229217	−397.474109	−397.666558	−397.818699	−397.933547	−398.013863	−398.062819
$\sigma^{5,4}\pi_+^{1,1}\pi_-^{2,2}\delta_-^{1,0}$	−397.112624	−397.343354	−397.518295	−397.656301	−397.759784	−397.830233	−397.870285
$\sigma^{5,3}\pi_+^{2,1}\pi_-^{3,1}\delta_-^{1,0}$	−396.592753	−397.002260	−397.348311	−397.646177	−397.903135	−398.123734	−398.311729
$\sigma^{5,4}\pi_+^{1,1}\pi_-^{3,1}\delta_-^{1,0}$	−396.733783	−397.083123	−397.369478	−397.610677	−397.814518	−397.985198	−398.126183
$\sigma^{5,3}\pi_+^{2,1}\pi_-^{2,1}\delta_-^{1,0}\phi_-^{1,0}$							
$\sigma^{5,3}\pi_+^{1,1}\pi_-^{3,1}\delta_-^{1,0}\phi_-^{1,0}$							

Table S475: Total energies in E_h for the S atom in the AHGBSP1-7 basis set in fully uncontracted form, employing the real-orbital approximation.

	$0.00B_0$	$0.10B_0$	$0.20B_0$	$0.30B_0$	$0.40B_0$	$0.50B_0$	$0.60B_0$
$\sigma^{5,5}\pi_+^{2,1}\pi_-^{2,1}$	−397.510916	−397.588696	−397.623122	−397.616839	−397.573063	−397.494994	−397.385596
$\sigma^{5,4}\pi_+^{2,1}\pi_-^{2,2}$	−397.507551	−397.632351	−397.708339	−397.739131	−397.728804	−397.681208	−397.599788
$\sigma^{5,4}\pi_+^{2,2}\pi_-^{2,1}$	−397.507551	−397.532351	−397.508339	−397.439131	−397.328804	−397.181208	−396.999788
$\sigma^{5,4}\pi_+^{2,1}\pi_-^{2,1}\delta_-^{1,0}$	−397.238341	−397.483254	−397.675705	−397.827893	−397.942715	−398.023244	−398.072404
$\sigma^{5,4}\pi_+^{1,1}\pi_-^{2,2}\delta_-^{1,0}$	−397.121748	−397.352545	−397.527441	−397.665623	−397.768972	−397.839675	−397.880069
$\sigma^{5,3}\pi_+^{2,1}\pi_-^{3,1}\delta_-^{1,0}$	−396.601844	−397.011343	−397.357408	−397.655293	−397.912233	−398.132978	−398.321194
$\sigma^{5,4}\pi_+^{1,1}\pi_-^{3,1}\delta_-^{1,0}$	−396.742953	−397.092272	−397.378650	−397.619904	−397.823676	−397.994531	−398.135836
$\sigma^{5,3}\pi_+^{2,1}\pi_-^{2,1}\delta_-^{1,0}\phi_-^{1,0}$							
$\sigma^{5,3}\pi_+^{1,1}\pi_-^{3,1}\delta_-^{1,0}\phi_-^{1,0}$							

Table S476: Total energies in E_h for the S atom in the AHGBSP1-9 basis set in fully uncontracted form, employing the real-orbital approximation.

	$0.00B_0$	$0.10B_0$	$0.20B_0$	$0.30B_0$	$0.40B_0$	$0.50B_0$	$0.60B_0$
$\sigma^{5,5}\pi_+^{2,1}\pi_-^{2,1}$	−397.511031	−397.588812	−397.623238	−397.616957	−397.573184	−397.495124	−397.385741
$\sigma^{5,4}\pi_+^{2,1}\pi_-^{2,2}$	−397.507666	−397.632466	−397.708455	−397.739249	−397.728925	−397.681339	−397.599950
$\sigma^{5,4}\pi_+^{2,2}\pi_-^{2,1}$	−397.507666	−397.532466	−397.508455	−397.439249	−397.328925	−397.181339	−396.999950
$\sigma^{5,4}\pi_+^{2,1}\pi_-^{2,1}\delta_-^{1,0}$	−397.238457	−397.483370	−397.675823	−397.828020	−397.942844	−398.023372	−398.072574
$\sigma^{5,4}\pi_+^{1,1}\pi_-^{2,2}\delta_-^{1,0}$	−397.121864	−397.352663	−397.527568	−397.665753	−397.769117	−397.839799	−397.880238
$\sigma^{5,3}\pi_+^{2,1}\pi_-^{3,1}\delta_-^{1,0}$	−396.601959	−397.011457	−397.357523	−397.655413	−397.912359	−398.133104	−398.321347
$\sigma^{5,4}\pi_+^{1,1}\pi_-^{3,1}\delta_-^{1,0}$	−396.743070	−397.092388	−397.378767	−397.620030	−397.823813	−397.994659	−398.135990
$\sigma^{5,3}\pi_+^{2,1}\pi_-^{2,1}\delta_-^{1,0}\phi_-^{1,0}$							
$\sigma^{5,3}\pi_+^{1,1}\pi_-^{3,1}\delta_-^{1,0}\phi_-^{1,0}$							

Table S477: Total energies in E_h for the S atom in the AHGBSP2-5 basis set in fully uncontracted form, employing the real-orbital approximation.

	$0.00B_0$	$0.10B_0$	$0.20B_0$	$0.30B_0$	$0.40B_0$	$0.50B_0$	$0.60B_0$
$\sigma^{5,5}\pi_+^{2,1}\pi_-^{2,1}$	−397.504199	−397.581824	−397.616037	−397.609988	−397.567365	−397.491752	−397.386370
$\sigma^{5,4}\pi_+^{2,1}\pi_-^{2,2}$	−397.499125	−397.624050	−397.700592	−397.732692	−397.724721	−397.680810	−397.604626
$\sigma^{5,4}\pi_+^{2,2}\pi_-^{2,1}$	−397.499125	−397.524050	−397.500592	−397.432692	−397.324721	−397.180810	−397.004626
$\sigma^{5,4}\pi_+^{2,1}\pi_-^{2,1}\delta_-^{1,0}$	−397.229227	−397.474154	−397.666738	−397.819379	−397.935402	−398.017808	−398.069927
$\sigma^{5,4}\pi_+^{1,1}\pi_-^{2,2}\delta_-^{1,0}$	−397.113611	−397.344476	−397.519878	−397.659010	−397.764586	−397.838310	−397.882954
$\sigma^{5,3}\pi_+^{2,1}\pi_-^{3,1}\delta_-^{1,0}$	−396.593576	−397.003174	−397.349528	−397.648007	−397.906138	−398.128740	−398.319792
$\sigma^{5,4}\pi_+^{1,1}\pi_-^{3,1}\delta_-^{1,0}$	−396.733898	−397.084054	−397.370712	−397.612538	−397.817480	−397.989912	−398.133453
$\sigma^{5,3}\pi_+^{2,1}\pi_-^{2,1}\delta_-^{1,0}\phi_-^{1,0}$	−396.529238	−396.936648	−397.262399	−397.546244	−397.791485	−398.008511	−398.198565
$\sigma^{5,3}\pi_+^{1,1}\pi_-^{3,1}\delta_-^{1,0}\phi_-^{1,0}$	−395.954700	−396.463620	−396.890931	−397.275083	−397.620241	−397.936756	−398.226188

Table S478: Total energies in E_h for the S atom in the AHGBSP2-7 basis set in fully uncontracted form, employing the real-orbital approximation.

	$0.00B_0$	$0.10B_0$	$0.20B_0$	$0.30B_0$	$0.40B_0$	$0.50B_0$	$0.60B_0$
$\sigma^{5,5}\pi_+^{2,1}\pi_-^{2,1}$	−397.513273	−397.590901	−397.625126	−397.619115	−397.576564	−397.501029	−397.395710
$\sigma^{5,4}\pi_+^{2,1}\pi_-^{2,2}$	−397.508200	−397.633127	−397.709682	−397.741836	−397.733977	−397.690180	−397.614037
$\sigma^{5,4}\pi_+^{2,2}\pi_-^{2,1}$	−397.508200	−397.533127	−397.509682	−397.441836	−397.333977	−397.190180	−397.014037
$\sigma^{5,4}\pi_+^{2,1}\pi_-^{2,1}\delta_-^{1,0}$	−397.238350	−397.483298	−397.675885	−397.828572	−397.944565	−398.027175	−398.079493
$\sigma^{5,4}\pi_+^{1,1}\pi_-^{2,2}\delta_-^{1,0}$	−397.122735	−397.353667	−397.529023	−397.668328	−397.773766	−397.847730	−397.892696
$\sigma^{5,3}\pi_+^{2,1}\pi_-^{3,1}\delta_-^{1,0}$	−396.602666	−397.012256	−397.358625	−397.657119	−397.915233	−398.137982	−398.329254
$\sigma^{5,4}\pi_+^{1,1}\pi_-^{3,1}\delta_-^{1,0}$	−396.743068	−397.093202	−397.379883	−397.621763	−397.826633	−397.999240	−398.143098
$\sigma^{5,3}\pi_+^{2,1}\pi_-^{2,1}\delta_-^{1,0}\phi_-^{1,0}$	−396.538357	−396.945835	−397.272493	−397.555586	−397.802975	−398.019240	−398.207983
$\sigma^{5,3}\pi_+^{1,1}\pi_-^{3,1}\delta_-^{1,0}\phi_-^{1,0}$	−395.963795	−396.472762	−396.900859	−397.284446	−397.631519	−397.947246	−398.235600

Table S479: Total energies in E_h for the S atom in the AHGBSP2-9 basis set in fully uncontracted form, employing the real-orbital approximation.

	$0.00B_0$	$0.10B_0$	$0.20B_0$	$0.30B_0$	$0.40B_0$	$0.50B_0$	$0.60B_0$
$\sigma^{5,5}\pi_+^{2,1}\pi_-^{2,1}$	−397.513388	−397.591017	−397.625242	−397.619232	−397.576684	−397.501158	−397.395851
$\sigma^{5,4}\pi_+^{2,1}\pi_-^{2,2}$	−397.508315	−397.633242	−397.709799	−397.741954	−397.734098	−397.690311	−397.614191
$\sigma^{5,4}\pi_+^{2,2}\pi_-^{2,1}$	−397.508315	−397.533242	−397.509799	−397.441954	−397.334098	−397.190311	−397.014191
$\sigma^{5,4}\pi_+^{2,1}\pi_-^{2,1}\delta_-^{1,0}$	−397.238466	−397.483414	−397.676003	−397.828699	−397.944694	−398.027303	−398.079658
$\sigma^{5,4}\pi_+^{1,1}\pi_-^{2,2}\delta_-^{1,0}$	−397.122850	−397.353785	−397.529150	−397.668458	−397.773910	−397.847854	−397.892860
$\sigma^{5,3}\pi_+^{2,1}\pi_-^{3,1}\delta_-^{1,0}$	−396.602781	−397.012370	−397.358739	−397.657240	−397.915359	−398.138107	−398.329403
$\sigma^{5,4}\pi_+^{1,1}\pi_-^{3,1}\delta_-^{1,0}$	−396.743184	−397.093318	−397.380000	−397.621889	−397.826770	−397.999368	−398.143249
$\sigma^{5,3}\pi_+^{2,1}\pi_-^{2,1}\delta_-^{1,0}\phi_-^{1,0}$	−396.538472	−396.945979	−397.272674	−397.555742	−397.803241	−398.019643	−398.208175
$\sigma^{5,3}\pi_+^{1,1}\pi_-^{3,1}\delta_-^{1,0}\phi_-^{1,0}$	−395.963910	−396.472905	−396.901038	−397.284592	−397.631782	−397.947615	−398.235777

Table S480: Total energies in E_h for the S atom in the AHGBSP3-5 basis set in fully uncontracted form, employing the real-orbital approximation.

	$0.00B_0$	$0.10B_0$	$0.20B_0$	$0.30B_0$	$0.40B_0$	$0.50B_0$	$0.60B_0$
$\sigma^{5,5}\pi_+^{2,1}\pi_-^{2,1}$	−397.504199	−397.581825	−397.616038	−397.609992	−397.567384	−397.491802	−397.386490
$\sigma^{5,4}\pi_+^{2,1}\pi_-^{2,2}$	−397.499124	−397.624050	−397.700592	−397.732693	−397.724724	−397.680825	−397.604671
$\sigma^{5,4}\pi_+^{2,2}\pi_-^{2,1}$	−397.499124	−397.524050	−397.500592	−397.432693	−397.324724	−397.180825	−397.004671
$\sigma^{5,4}\pi_+^{2,1}\pi_-^{2,1}\delta_-^{1,0}$	−397.229226	−397.474410	−397.667393	−397.820518	−397.937125	−398.020252	−398.073194
$\sigma^{5,4}\pi_+^{1,1}\pi_-^{2,2}\delta_-^{1,0}$	−397.113612	−397.345028	−397.521161	−397.660993	−397.767288	−397.841866	−397.887440
$\sigma^{5,3}\pi_+^{2,1}\pi_-^{3,1}\delta_-^{1,0}$	−396.593649	−397.003559	−397.351075	−397.651426	−397.912013	−398.137656	−398.332280
$\sigma^{5,4}\pi_+^{1,1}\pi_-^{3,1}\delta_-^{1,0}$	−396.733906	−397.084473	−397.372555	−397.616554	−397.824176	−397.999809	−398.147020
$\sigma^{5,3}\pi_+^{2,1}\pi_-^{2,1}\delta_-^{1,0}\phi_-^{1,0}$	−396.529242	−396.936676	−397.262567	−397.546715	−397.792425	−398.010072	−398.200870
$\sigma^{5,3}\pi_+^{1,1}\pi_-^{3,1}\delta_-^{1,0}\phi_-^{1,0}$	−395.954772	−396.463806	−396.891662	−397.277022	−397.624131	−397.943289	−398.235935

Table S481: Total energies in E_h for the S atom in the AHGBSP3-7 basis set in fully uncontracted form, employing the real-orbital approximation.

	$0.00B_0$	$0.10B_0$	$0.20B_0$	$0.30B_0$	$0.40B_0$	$0.50B_0$	$0.60B_0$
$\sigma^{5,5}\pi_+^{2,1}\pi_-^{2,1}$	−397.513274	−397.590902	−397.625128	−397.619120	−397.576583	−397.501081	−397.395830
$\sigma^{5,4}\pi_+^{2,1}\pi_-^{2,2}$	−397.508200	−397.633127	−397.709683	−397.741837	−397.733982	−397.690195	−397.614082
$\sigma^{5,4}\pi_+^{2,2}\pi_-^{2,1}$	−397.508200	−397.533127	−397.509683	−397.441837	−397.333982	−397.190195	−397.014082
$\sigma^{5,4}\pi_+^{2,1}\pi_-^{2,1}\delta_-^{1,0}$	−397.238351	−397.483552	−397.676534	−397.829707	−397.946290	−398.029591	−398.082717
$\sigma^{5,4}\pi_+^{1,1}\pi_-^{2,2}\delta_-^{1,0}$	−397.122737	−397.354209	−397.530297	−397.670297	−397.776478	−397.851249	−397.897113
$\sigma^{5,3}\pi_+^{2,1}\pi_-^{3,1}\delta_-^{1,0}$	−396.602740	−397.012641	−397.360168	−397.660528	−397.921114	−398.146873	−398.341653
$\sigma^{5,4}\pi_+^{1,1}\pi_-^{3,1}\delta_-^{1,0}$	−396.743076	−397.093621	−397.381721	−397.625756	−397.833339	−398.009112	−398.156550
$\sigma^{5,3}\pi_+^{2,1}\pi_-^{2,1}\delta_-^{1,0}\phi_-^{1,0}$	−396.538362	−396.945863	−397.272660	−397.556059	−397.803913	−398.020787	−398.210273
$\sigma^{5,3}\pi_+^{1,1}\pi_-^{3,1}\delta_-^{1,0}\phi_-^{1,0}$	−395.963867	−396.472947	−396.901586	−397.286392	−397.635400	−397.953733	−398.245291

Table S482: Total energies in E_h for the S atom in the AHGBSP3-9 basis set in fully uncontracted form, employing the real-orbital approximation.

	$0.00B_0$	$0.10B_0$	$0.20B_0$	$0.30B_0$	$0.40B_0$	$0.50B_0$	$0.60B_0$
$\sigma^{5,5}\pi_+^{2,1}\pi_-^{2,1}$	−397.513389	−397.591017	−397.625244	−397.619237	−397.576703	−397.501209	−397.395971
$\sigma^{5,4}\pi_+^{2,1}\pi_-^{2,2}$	−397.508315	−397.633243	−397.709799	−397.741955	−397.734102	−397.690327	−397.614236
$\sigma^{5,4}\pi_+^{2,2}\pi_-^{2,1}$	−397.508315	−397.533243	−397.509799	−397.441955	−397.334102	−397.190327	−397.014236
$\sigma^{5,4}\pi_+^{2,1}\pi_-^{2,1}\delta_-^{1,0}$	−397.238467	−397.483668	−397.676652	−397.829833	−397.946418	−398.029720	−398.082878
$\sigma^{5,4}\pi_+^{1,1}\pi_-^{2,2}\delta_-^{1,0}$	−397.122852	−397.354327	−397.530423	−397.670425	−397.776618	−397.851375	−397.897273
$\sigma^{5,3}\pi_+^{2,1}\pi_-^{3,1}\delta_-^{1,0}$	−396.602854	−397.012755	−397.360282	−397.660647	−397.921235	−398.146997	−398.341802
$\sigma^{5,4}\pi_+^{1,1}\pi_-^{3,1}\delta_-^{1,0}$	−396.743193	−397.093736	−397.381837	−397.625881	−397.833468	−398.009238	−398.156700
$\sigma^{5,3}\pi_+^{2,1}\pi_-^{2,1}\delta_-^{1,0}\phi_-^{1,0}$	−396.538477	−396.946007	−397.272841	−397.556216	−397.804179	−398.021190	−398.210463
$\sigma^{5,3}\pi_+^{1,1}\pi_-^{3,1}\delta_-^{1,0}\phi_-^{1,0}$	−395.963982	−396.473091	−396.901766	−397.286537	−397.635663	−397.954099	−398.245464

Table S483: Total energies in E_h for the S atom in the 6-311++G(3df,3pd) basis set in fully uncontracted form, employing the real-orbital approximation.

	$0.00B_0$	$0.10B_0$	$0.20B_0$	$0.30B_0$	$0.40B_0$	$0.50B_0$	$0.60B_0$
$\sigma^{5,5}\pi_+^{2,1}\pi_-^{2,1}$	−397.504191	−397.581790	−397.615660	−397.608517	−397.563802	−397.485003	−397.375295
$\sigma^{5,4}\pi_+^{2,1}\pi_-^{2,2}$	−397.499473	−397.624222	−397.700018	−397.730580	−397.720373	−397.673672	−397.594088
$\sigma^{5,4}\pi_+^{2,2}\pi_-^{2,1}$	−397.499473	−397.524222	−397.500018	−397.430580	−397.320373	−397.173672	−396.994088
$\sigma^{5,4}\pi_+^{2,1}\pi_-^{2,1}\delta_-^{1,0}$	−397.153911	−397.426009	−397.643381	−397.808995	−397.927175	−398.003180	−398.042897
$\sigma^{5,4}\pi_+^{1,1}\pi_-^{2,2}\delta_-^{1,0}$	−396.994377	−397.265627	−397.480592	−397.642472	−397.755749	−397.825781	−397.858613
$\sigma^{5,3}\pi_+^{2,1}\pi_-^{3,1}\delta_-^{1,0}$	−396.534513	−396.955493	−397.319376	−397.628855	−397.888089	−398.102388	−398.277861
$\sigma^{5,4}\pi_+^{1,1}\pi_-^{3,1}\delta_-^{1,0}$	−396.696549	−397.037334	−397.277482	−397.590448	−397.801669	−397.968336	−398.096784
$\sigma^{5,3}\pi_+^{2,1}\pi_-^{2,1}\delta_-^{1,0}\phi_-^{1,0}$	−395.137332	−395.659090	−396.125280	−396.538461	−396.902419	−397.221790	−397.501694
$\sigma^{5,3}\pi_+^{1,1}\pi_-^{3,1}\delta_-^{1,0}\phi_-^{1,0}$	−394.605517	−395.226481	−395.790174	−396.298922	−396.756413	−397.167482	−397.537785

Table S484: Total energies in E_h for the S atom in the def2-TZVP basis set in fully uncontracted form, employing the real-orbital approximation.

	$0.00B_0$	$0.10B_0$	$0.20B_0$	$0.30B_0$	$0.40B_0$	$0.50B_0$	$0.60B_0$
$\sigma^{5,5}\pi_+^{2,1}\pi_-^{2,1}$	−397.503854	−397.581586	−397.615649	−397.608424	−397.563253	−397.483809	−397.373617
$\sigma^{5,4}\pi_+^{2,1}\pi_-^{2,2}$	−397.499082	−397.624067	−397.700174	−397.730552	−397.719566	−397.671853	−397.591598
$\sigma^{5,4}\pi_+^{2,2}\pi_-^{2,1}$	−397.499082	−397.524067	−397.500174	−397.430552	−397.319566	−397.171853	−396.991598
$\sigma^{5,4}\pi_+^{2,1}\pi_-^{2,1}\delta_-^{1,0}$	−397.070784	−397.346060	−397.572687	−397.752878	−397.889788	−397.986986	−398.048070
$\sigma^{5,4}\pi_+^{1,1}\pi_-^{2,2}\delta_-^{1,0}$	−396.893872	−397.168376	−397.392821	−397.569772	−397.702834	−397.795978	−397.853055
$\sigma^{5,3}\pi_+^{2,1}\pi_-^{3,1}\delta_-^{1,0}$	−396.408219	−396.833733	−397.210913	−397.541532	−397.828161	−398.073783	−398.281524
$\sigma^{5,4}\pi_+^{1,1}\pi_-^{3,1}\delta_-^{1,0}$	−396.462749	−396.829232	−397.129128	−397.476868	−397.718994	−397.921221	−398.086599
$\sigma^{5,3}\pi_+^{2,1}\pi_-^{2,1}\delta_-^{1,0}\phi_-^{1,0}$	−395.056819	−395.581471	−396.056085	−396.482473	−396.863211	−397.201201	−397.499365
$\sigma^{5,3}\pi_+^{1,1}\pi_-^{3,1}\delta_-^{1,0}\phi_-^{1,0}$	−394.463554	−395.089216	−395.666654	−396.197135	−396.682541	−397.125147	−397.527448

Table S485: Total energies in E_h for the Cl atom in the cc-pVDZ basis set in fully uncontracted form, employing the real-orbital approximation.

	$0.00B_0$	$0.10B_0$	$0.20B_0$	$0.30B_0$	$0.40B_0$	$0.50B_0$	$0.60B_0$
$\sigma^{5,4}\pi_+^{2,2}\pi_-^{2,2}$	−459.471358	−459.497233	−459.475375	−459.407289	−459.295340	−459.142555	−458.952355
$\sigma^{5,5}\pi_+^{2,2}\pi_-^{2,1}$	−459.468465	−459.446445	−459.380824	−459.272873	−459.124593	−458.938534	−458.717583
$\sigma^{5,5}\pi_+^{2,1}\pi_-^{2,2}$	−459.468465	−459.546445	−459.580824	−459.572873	−459.524593	−459.438534	−459.317583
$\sigma^{5,4}\pi_+^{2,1}\pi_-^{2,2}\delta_-^{1,0}$	−458.578391	−458.855291	−459.086418	−459.273023	−459.417059	−459.521013	−459.587679
$\sigma^{5,5}\pi_+^{2,1}\pi_-^{2,1}\delta_-^{1,0}$	−458.633471	−458.862700	−459.050712	−459.198452	−459.307398	−459.379431	−459.416675
$\sigma^{5,4}\pi_+^{2,1}\pi_-^{3,1}\delta_-^{1,0}$	−457.924088	−458.297294	−458.617122	−458.884187	−459.099458	−459.264186	−459.379827
$\sigma^{5,4}\pi_+^{2,1}\pi_-^{2,1}\delta_-^{1,0}\phi_-^{1,0}$							
$\sigma^{5,3}\pi_+^{2,1}\pi_-^{3,1}\delta_-^{1,0}\phi_-^{1,0}$							
$\sigma^{6,3}\pi_+^{2,1}\pi_-^{3,1}\delta_-^{1,0}$	−456.920672	−457.392381	−457.807647	−458.166873	−458.470695	−458.719932	−458.915545

Table S486: Total energies in E_h for the Cl atom in the cc-pVTZ basis set in fully uncontracted form, employing the real-orbital approximation.

	$0.00B_0$	$0.10B_0$	$0.20B_0$	$0.30B_0$	$0.40B_0$	$0.50B_0$	$0.60B_0$
$\sigma^{5,4}\pi_+^{2,2}\pi_-^{2,2}$	−459.485508	−459.511258	−459.489281	−459.421756	−459.311885	−459.163379	−458.979939
$\sigma^{5,5}\pi_+^{2,2}\pi_-^{2,1}$	−459.480732	−459.458539	−459.392605	−459.284742	−459.137629	−458.954387	−458.738189
$\sigma^{5,5}\pi_+^{2,1}\pi_-^{2,2}$	−459.480732	−459.558539	−459.592605	−459.584742	−459.537629	−459.454387	−459.338189
$\sigma^{5,4}\pi_+^{2,1}\pi_-^{2,2}\delta_-^{1,0}$	−458.849061	−459.124816	−459.352695	−459.534442	−459.672625	−459.770253	−459.830406
$\sigma^{5,5}\pi_+^{2,1}\pi_-^{2,1}\delta_-^{1,0}$	−458.900529	−459.128505	−459.312894	−459.455003	−459.556795	−459.620641	−459.649085
$\sigma^{5,4}\pi_+^{2,1}\pi_-^{3,1}\delta_-^{1,0}$	−458.326936	−458.703022	−459.035535	−459.324361	−459.571255	−459.778337	−459.947898
$\sigma^{5,4}\pi_+^{2,1}\pi_-^{2,1}\delta_-^{1,0}\phi_-^{1,0}$	−456.649854	−457.126649	−457.557464	−457.943518	−458.286616	−458.588907	−458.852658
$\sigma^{5,3}\pi_+^{2,1}\pi_-^{3,1}\delta_-^{1,0}\phi_-^{1,0}$	−455.790772	−456.409134	−456.964513	−457.457770	−457.890305	−458.504902	−458.915852
$\sigma^{6,3}\pi_+^{2,1}\pi_-^{3,1}\delta_-^{1,0}$	−457.397295	−457.863718	−458.263269	−458.596796	−458.865711	−459.358339	−459.598020

Table S487: Total energies in E_h for the Cl atom in the cc-pVQZ basis set in fully uncontracted form, employing the real-orbital approximation.

	$0.00B_0$	$0.10B_0$	$0.20B_0$	$0.30B_0$	$0.40B_0$	$0.50B_0$	$0.60B_0$
$\sigma^{5,4}\pi_+^{2,2}\pi_-^{2,2}$	−459.489134	−459.514856	−459.493001	−459.426207	−459.318033	−459.172181	−458.991995
$\sigma^{5,5}\pi_+^{2,2}\pi_-^{2,1}$	−459.484065	−459.461812	−459.395865	−459.288424	−459.142505	−458.961330	−458.747932
$\sigma^{5,5}\pi_+^{2,1}\pi_-^{2,2}$	−459.484065	−459.561812	−459.595865	−459.588424	−459.542505	−459.461330	−459.347932
$\sigma^{5,4}\pi_+^{2,1}\pi_-^{2,2}\delta_-^{1,0}$	−458.944798	−459.219286	−459.443533	−459.619695	−459.750840	−459.840462	−459.892165
$\sigma^{5,5}\pi_+^{2,1}\pi_-^{2,1}\delta_-^{1,0}$	−458.990036	−459.216674	−459.397204	−459.533360	−459.627703	−459.683302	−459.703486
$\sigma^{5,4}\pi_+^{2,1}\pi_-^{3,1}\delta_-^{1,0}$	−458.493570	−458.868924	−459.195545	−459.475012	−459.709710	−459.902583	−460.056937
$\sigma^{5,4}\pi_+^{2,1}\pi_-^{2,1}\delta_-^{1,0}\phi_-^{1,0}$	−457.472742	−457.946327	−458.367597	−458.738007	−459.059726	−459.335398	−459.567938
$\sigma^{5,3}\pi_+^{2,1}\pi_-^{3,1}\delta_-^{1,0}\phi_-^{1,0}$	−456.614761	−457.226917	−457.764022	−458.384421	−458.852281	−459.272642	−459.648383
$\sigma^{6,3}\pi_+^{2,1}\pi_-^{3,1}\delta_-^{1,0}$	−457.584418	−458.045559	−458.429704	−458.923026	−459.336403	−459.637371	−459.900590

Table S488: Total energies in E_h for the Cl atom in the cc-pV5Z basis set in fully uncontracted form, employing the real-orbital approximation.

	$0.00B_0$	$0.10B_0$	$0.20B_0$	$0.30B_0$	$0.40B_0$	$0.50B_0$	$0.60B_0$
$\sigma^{5,4}\pi_+^{2,2}\pi_-^{2,2}$	−459.489733	−459.515460	−459.493718	−459.427316	−459.319841	−459.174851	−458.995522
$\sigma^{5,5}\pi_+^{2,2}\pi_-^{2,1}$	−459.484592	−459.462326	−459.396431	−459.289275	−459.143962	−458.963676	−458.751365
$\sigma^{5,5}\pi_+^{2,1}\pi_-^{2,2}$	−459.484592	−459.562326	−459.596431	−459.589275	−459.543962	−459.463676	−459.351365
$\sigma^{5,4}\pi_+^{2,1}\pi_-^{2,2}\delta_-^{1,0}$	−458.954411	−459.228988	−459.453579	−459.630503	−459.762956	−459.854504	−459.908818
$\sigma^{5,5}\pi_+^{2,1}\pi_-^{2,1}\delta_-^{1,0}$	−459.000128	−459.226855	−459.407714	−459.544591	−459.640223	−459.697842	−459.720906
$\sigma^{5,4}\pi_+^{2,1}\pi_-^{3,1}\delta_-^{1,0}$	−458.539276	−458.901660	−459.217239	−459.497863	−459.734448	−459.930130	−460.088404
$\sigma^{5,4}\pi_+^{2,1}\pi_-^{2,1}\delta_-^{1,0}\phi_-^{1,0}$	−457.798913	−458.270285	−458.684961	−459.044528	−459.351389	−459.608534	−459.819362
$\sigma^{5,3}\pi_+^{2,1}\pi_-^{3,1}\delta_-^{1,0}\phi_-^{1,0}$	−456.940051	−457.547929	−458.072702	−458.692046	−459.146508	−459.550342	−459.906944
$\sigma^{6,3}\pi_+^{2,1}\pi_-^{3,1}\delta_-^{1,0}$	−457.722381	−458.179724	−458.551737	−459.037023	−459.380021	−459.683444	−459.950337

Table S489: Total energies in E_h for the Cl atom in the aug-cc-pVDZ basis set in fully uncontracted form, employing the real-orbital approximation.

	$0.00B_0$	$0.10B_0$	$0.20B_0$	$0.30B_0$	$0.40B_0$	$0.50B_0$	$0.60B_0$
$\sigma^{5,4}\pi_+^{2,2}\pi_-^{2,2}$	−459.472997	−459.498487	−459.475952	−459.407753	−459.296719	−459.145854	−458.958319
$\sigma^{5,5}\pi_+^{2,2}\pi_-^{2,1}$	−459.469863	−459.447565	−459.381469	−459.273519	−459.126122	−458.941878	−458.723505
$\sigma^{5,5}\pi_+^{2,1}\pi_-^{2,2}$	−459.469863	−459.547565	−459.581469	−459.573519	−459.526122	−459.441878	−459.323505
$\sigma^{5,4}\pi_+^{2,1}\pi_-^{2,2}\delta_-^{1,0}$	−458.937153	−459.210074	−459.429615	−459.597854	−459.717769	−459.793048	−459.827976
$\sigma^{5,5}\pi_+^{2,1}\pi_-^{2,1}\delta_-^{1,0}$	−458.974637	−459.199823	−459.376009	−459.504958	−459.589332	−459.632512	−459.638410
$\sigma^{5,4}\pi_+^{2,1}\pi_-^{3,1}\delta_-^{1,0}$	−458.571313	−458.920186	−459.170698	−459.426684	−459.646360	−459.820950	−459.954729
$\sigma^{5,4}\pi_+^{2,1}\pi_-^{2,1}\delta_-^{1,0}\phi_-^{1,0}$							
$\sigma^{5,3}\pi_+^{2,1}\pi_-^{3,1}\delta_-^{1,0}\phi_-^{1,0}$							
$\sigma^{6,3}\pi_+^{2,1}\pi_-^{3,1}\delta_-^{1,0}$	−457.740308	−458.203118	−458.593941	−458.925436	−459.230202	−459.506039	−459.745238

Table S490: Total energies in E_h for the Cl atom in the aug-cc-pVTZ basis set in fully uncontracted form, employing the real-orbital approximation.

	$0.00B_0$	$0.10B_0$	$0.20B_0$	$0.30B_0$	$0.40B_0$	$0.50B_0$	$0.60B_0$
$\sigma^{5,4}\pi_+^{2,2}\pi_-^{2,2}$	−459.486044	−459.511725	−459.489868	−459.423166	−459.315018	−459.169064	−458.988857
$\sigma^{5,5}\pi_+^{2,2}\pi_-^{2,1}$	−459.481049	−459.458753	−459.392775	−459.285407	−459.139614	−458.958613	−458.745582
$\sigma^{5,5}\pi_+^{2,1}\pi_-^{2,2}$	−459.481049	−459.558753	−459.592775	−459.585407	−459.539614	−459.458613	−459.345582
$\sigma^{5,4}\pi_+^{2,1}\pi_-^{2,2}\delta_-^{1,0}$	−459.018110	−459.288207	−459.500059	−459.658134	−459.769179	−459.840878	−459.879910
$\sigma^{5,5}\pi_+^{2,1}\pi_-^{2,1}\delta_-^{1,0}$	−459.050577	−459.273083	−459.442088	−459.561855	−459.638700	−459.679514	−459.689982
$\sigma^{5,4}\pi_+^{2,1}\pi_-^{3,1}\delta_-^{1,0}$	−458.637156	−458.982085	−459.269960	−459.526334	−459.735176	−459.904016	−460.039501
$\sigma^{5,4}\pi_+^{2,1}\pi_-^{2,1}\delta_-^{1,0}\phi_-^{1,0}$	−457.807886	−458.275980	−458.681595	−459.028493	−459.322063	−459.568006	−459.771090
$\sigma^{5,3}\pi_+^{2,1}\pi_-^{3,1}\delta_-^{1,0}\phi_-^{1,0}$	−456.992753	−457.609887	−458.162659	−458.654870	−459.091883	−459.479430	−459.822576
$\sigma^{6,3}\pi_+^{2,1}\pi_-^{3,1}\delta_-^{1,0}$	−457.854282	−458.315190	−458.705382	−459.054092	−459.369650	−459.648046	−459.892640

Table S491: Total energies in E_h for the Cl atom in the aug-cc-pVQZ basis set in fully uncontracted form, employing the real-orbital approximation.

	$0.00B_0$	$0.10B_0$	$0.20B_0$	$0.30B_0$	$0.40B_0$	$0.50B_0$	$0.60B_0$
$\sigma^{5,4}\pi_+^{2,2}\pi_-^{2,2}$	−459.489219	−459.514938	−459.493253	−459.427013	−459.319761	−459.175068	−458.996155
$\sigma^{5,5}\pi_+^{2,2}\pi_-^{2,1}$	−459.484114	−459.461838	−459.395985	−459.289005	−459.144011	−458.964195	−458.752494
$\sigma^{5,5}\pi_+^{2,1}\pi_-^{2,2}$	−459.484114	−459.561838	−459.595985	−459.589005	−459.544011	−459.464195	−459.352494
$\sigma^{5,4}\pi_+^{2,1}\pi_-^{2,2}\delta_-^{1,0}$	−459.054119	−459.320231	−459.522500	−459.671741	−459.780482	−459.856117	−459.901077
$\sigma^{5,5}\pi_+^{2,1}\pi_-^{2,1}\delta_-^{1,0}$	−459.081192	−459.300259	−459.461360	−459.574605	−459.650619	−459.695254	−459.710671
$\sigma^{5,4}\pi_+^{2,1}\pi_-^{3,1}\delta_-^{1,0}$	−458.632654	−458.998840	−459.301529	−459.551036	−459.758591	−459.931961	−460.075100
$\sigma^{5,4}\pi_+^{2,1}\pi_-^{2,1}\delta_-^{1,0}\phi_-^{1,0}$	−458.102898	−458.565142	−458.955064	−459.280279	−459.548520	−459.765351	−459.935244
$\sigma^{5,3}\pi_+^{2,1}\pi_-^{3,1}\delta_-^{1,0}\phi_-^{1,0}$	−457.282492	−457.894811	−458.434473	−458.908004	−459.322623	−459.684446	−459.998781
$\sigma^{6,3}\pi_+^{2,1}\pi_-^{3,1}\delta_-^{1,0}$	−457.891410	−458.350582	−458.741336	−459.094619	−459.412283	−459.694786	−459.945493

Table S492: Total energies in E_h for the Cl atom in the aug-cc-pV5Z basis set in fully uncontracted form, employing the real-orbital approximation.

	$0.00B_0$	$0.10B_0$	$0.20B_0$	$0.30B_0$	$0.40B_0$	$0.50B_0$	$0.60B_0$
$\sigma^{5,4}\pi_+^{2,2}\pi_-^{2,2}$	−459.489765	−459.515497	−459.493876	−459.427793	−459.320761	−459.176226	−458.997286
$\sigma^{5,5}\pi_+^{2,2}\pi_-^{2,1}$	−459.484607	−459.462337	−459.396535	−459.289698	−459.144925	−458.965311	−458.753683
$\sigma^{5,5}\pi_+^{2,1}\pi_-^{2,2}$	−459.484607	−459.562337	−459.596535	−459.589698	−459.544925	−459.465311	−459.353683
$\sigma^{5,4}\pi_+^{2,1}\pi_-^{2,2}\delta_-^{1,0}$	−459.055047	−459.322029	−459.526701	−459.679115	−459.790837	−459.869026	−459.916429
$\sigma^{5,5}\pi_+^{2,1}\pi_-^{2,1}\delta_-^{1,0}$	−459.083757	−459.303660	−459.467003	−459.583109	−459.661835	−459.708968	−459.726952
$\sigma^{5,4}\pi_+^{2,1}\pi_-^{3,1}\delta_-^{1,0}$	−458.644111	−459.011362	−459.316922	−459.570306	−459.782010	−459.959552	−460.107002
$\sigma^{5,4}\pi_+^{2,1}\pi_-^{2,1}\delta_-^{1,0}\phi_-^{1,0}$	−458.253575	−458.712213	−459.091264	−459.398474	−459.642867	−459.833350	−459.979587
$\sigma^{5,3}\pi_+^{2,1}\pi_-^{3,1}\delta_-^{1,0}\phi_-^{1,0}$	−457.444244	−458.053145	−458.582509	−459.039113	−459.431578	−459.769311	−460.062660
$\sigma^{6,3}\pi_+^{2,1}\pi_-^{3,1}\delta_-^{1,0}$	−457.919733	−458.378603	−458.770082	−459.128131	−459.450734	−459.738771	−459.995712

Table S493: Total energies in E_h for the Cl atom in the HGBSP1-5 basis set in fully uncontracted form, employing the real-orbital approximation.

	$0.00B_0$	$0.10B_0$	$0.20B_0$	$0.30B_0$	$0.40B_0$	$0.50B_0$	$0.60B_0$
$\sigma^{5,4}\pi_+^{2,2}\pi_-^{2,2}$	−459.476550	−459.502122	−459.479907	−459.412498	−459.303063	−459.154837	−458.970899
$\sigma^{5,5}\pi_+^{2,2}\pi_-^{2,1}$	−459.473082	−459.450865	−459.385071	−459.277822	−459.131777	−458.949703	−458.734274
$\sigma^{5,5}\pi_+^{2,1}\pi_-^{2,2}$	−459.473082	−459.550865	−459.585071	−459.577822	−459.531777	−459.449703	−459.334274
$\sigma^{5,4}\pi_+^{2,1}\pi_-^{2,2}\delta_-^{1,0}$	−459.100156	−459.335307	−459.519914	−459.667596	−459.778814	−459.854773	−459.897558
$\sigma^{5,5}\pi_+^{2,1}\pi_-^{2,1}\delta_-^{1,0}$	−459.109401	−459.307048	−459.457718	−459.571949	−459.651246	−459.697247	−459.712066
$\sigma^{5,4}\pi_+^{2,1}\pi_-^{3,1}\delta_-^{1,0}$	−458.657549	−459.016132	−459.312523	−459.562928	−459.774095	−459.949852	−460.093156
$\sigma^{5,4}\pi_+^{2,1}\pi_-^{2,1}\delta_-^{1,0}\phi_-^{1,0}$							
$\sigma^{5,3}\pi_+^{2,1}\pi_-^{3,1}\delta_-^{1,0}\phi_-^{1,0}$							
$\sigma^{6,3}\pi_+^{2,1}\pi_-^{3,1}\delta_-^{1,0}$	−457.875964	−458.340718	−458.769935	−459.129181	−459.449608	−459.733278	−459.983411

Table S494: Total energies in E_h for the Cl atom in the HGBSP1-7 basis set in fully uncontracted form, employing the real-orbital approximation.

	$0.00B_0$	$0.10B_0$	$0.20B_0$	$0.30B_0$	$0.40B_0$	$0.50B_0$	$0.60B_0$
$\sigma^{5,4}\pi_+^{2,2}\pi_-^{2,2}$	−459.487426	−459.513000	−459.490797	−459.423434	−459.314107	−459.166025	−458.982181
$\sigma^{5,5}\pi_+^{2,2}\pi_-^{2,1}$	−459.483958	−459.461743	−459.395960	−459.288747	−459.142785	−458.960828	−458.745498
$\sigma^{5,5}\pi_+^{2,1}\pi_-^{2,2}$	−459.483958	−459.561743	−459.595960	−459.588747	−459.542785	−459.460828	−459.345498
$\sigma^{5,4}\pi_+^{2,1}\pi_-^{2,2}\delta_-^{1,0}$	−459.111095	−459.346275	−459.530826	−459.678664	−459.789804	−459.865850	−459.908893
$\sigma^{5,5}\pi_+^{2,1}\pi_-^{2,1}\delta_-^{1,0}$	−459.120334	−459.317995	−459.468633	−459.582949	−459.662189	−459.708290	−459.723331
$\sigma^{5,4}\pi_+^{2,1}\pi_-^{3,1}\delta_-^{1,0}$	−458.668506	−459.027083	−459.323463	−459.573929	−459.785055	−459.960840	−460.104337
$\sigma^{5,4}\pi_+^{2,1}\pi_-^{2,1}\delta_-^{1,0}\phi_-^{1,0}$							
$\sigma^{5,3}\pi_+^{2,1}\pi_-^{3,1}\delta_-^{1,0}\phi_-^{1,0}$							
$\sigma^{6,3}\pi_+^{2,1}\pi_-^{3,1}\delta_-^{1,0}$	−457.886838	−458.351606	−458.781176	−459.140057	−459.460624	−459.744805	−459.995664

Table S495: Total energies in E_h for the Cl atom in the HGBSP1-9 basis set in fully uncontracted form, employing the real-orbital approximation.

	$0.00B_0$	$0.10B_0$	$0.20B_0$	$0.30B_0$	$0.40B_0$	$0.50B_0$	$0.60B_0$
$\sigma^{5,4}\pi_+^{2,2}\pi_-^{2,2}$	−459.487567	−459.513142	−459.490939	−459.423578	−459.314255	−459.166179	−458.982356
$\sigma^{5,5}\pi_+^{2,2}\pi_-^{2,1}$	−459.484099	−459.461885	−459.396102	−459.288891	−459.142932	−458.960981	−458.745665
$\sigma^{5,5}\pi_+^{2,1}\pi_-^{2,2}$	−459.484099	−459.561885	−459.596102	−459.588891	−459.542932	−459.460981	−459.345665
$\sigma^{5,4}\pi_+^{2,1}\pi_-^{2,2}\delta_-^{1,0}$	−459.111236	−459.346421	−459.530978	−459.678808	−459.789969	−459.866005	−459.909062
$\sigma^{5,5}\pi_+^{2,1}\pi_-^{2,1}\delta_-^{1,0}$	−459.120476	−459.318137	−459.468779	−459.583094	−459.662347	−459.708441	−459.723495
$\sigma^{5,4}\pi_+^{2,1}\pi_-^{3,1}\delta_-^{1,0}$	−458.668647	−459.027224	−459.323606	−459.574073	−459.785213	−459.960999	−460.104502
$\sigma^{5,4}\pi_+^{2,1}\pi_-^{2,1}\delta_-^{1,0}\phi_-^{1,0}$							
$\sigma^{5,3}\pi_+^{2,1}\pi_-^{3,1}\delta_-^{1,0}\phi_-^{1,0}$							
$\sigma^{6,3}\pi_+^{2,1}\pi_-^{3,1}\delta_-^{1,0}$	−457.886978	−458.351805	−458.782947	−459.140382	−459.460787	−459.745118	−459.996137

Table S496: Total energies in E_h for the Cl atom in the HGBSP2-5 basis set in fully uncontracted form, employing the real-orbital approximation.

	$0.00B_0$	$0.10B_0$	$0.20B_0$	$0.30B_0$	$0.40B_0$	$0.50B_0$	$0.60B_0$
$\sigma^{5,4}\pi_+^{2,2}\pi_-^{2,2}$	−459.478934	−459.504671	−459.483071	−459.416995	−459.309882	−459.165185	−458.986130
$\sigma^{5,5}\pi_+^{2,2}\pi_-^{2,1}$	−459.473745	−459.451479	−459.385697	−459.278880	−459.134068	−458.954349	−458.742632
$\sigma^{5,5}\pi_+^{2,1}\pi_-^{2,2}$	−459.473745	−459.551479	−459.585697	−459.578880	−459.534068	−459.454349	−459.342632
$\sigma^{5,4}\pi_+^{2,1}\pi_-^{2,2}\delta_-^{1,0}$	−459.100921	−459.336240	−459.521260	−459.669823	−459.782654	−459.861174	−459.907628
$\sigma^{5,5}\pi_+^{2,1}\pi_-^{2,1}\delta_-^{1,0}$	−459.111831	−459.309437	−459.460124	−459.574576	−459.654632	−459.702249	−459.719799
$\sigma^{5,4}\pi_+^{2,1}\pi_-^{3,1}\delta_-^{1,0}$	−458.658197	−459.016848	−459.313499	−459.564339	−459.776269	−459.953292	−460.098525
$\sigma^{5,4}\pi_+^{2,1}\pi_-^{2,1}\delta_-^{1,0}\phi_-^{1,0}$	−458.604846	−458.962157	−459.237808	−459.471970	−459.667441	−459.831949	−459.971360
$\sigma^{5,3}\pi_+^{2,1}\pi_-^{3,1}\delta_-^{1,0}\phi_-^{1,0}$	−457.795394	−458.303286	−458.729592	−459.113868	−459.458333	−459.770724	−460.056502
$\sigma^{6,3}\pi_+^{2,1}\pi_-^{3,1}\delta_-^{1,0}$	−457.877044	−458.341742	−458.770894	−459.130270	−459.451350	−459.736182	−459.988135

Table S497: Total energies in E_h for the Cl atom in the HGBSP2-7 basis set in fully uncontracted form, employing the real-orbital approximation.

	$0.00B_0$	$0.10B_0$	$0.20B_0$	$0.30B_0$	$0.40B_0$	$0.50B_0$	$0.60B_0$
$\sigma^{5,4}\pi_+^{2,2}\pi_-^{2,2}$	−459.489810	−459.515549	−459.493961	−459.427930	−459.320916	−459.176338	−458.997353
$\sigma^{5,5}\pi_+^{2,2}\pi_-^{2,1}$	−459.484621	−459.462357	−459.396585	−459.289805	−459.145069	−458.965446	−458.753797
$\sigma^{5,5}\pi_+^{2,1}\pi_-^{2,2}$	−459.484621	−459.562357	−459.596585	−459.589805	−459.545069	−459.465446	−459.353797
$\sigma^{5,4}\pi_+^{2,1}\pi_-^{2,2}\delta_-^{1,0}$	−459.111859	−459.347207	−459.532171	−459.680889	−459.793637	−459.872235	−459.918934
$\sigma^{5,5}\pi_+^{2,1}\pi_-^{2,1}\delta_-^{1,0}$	−459.122765	−459.320383	−459.471038	−459.585574	−459.665571	−459.713279	−459.731029
$\sigma^{5,4}\pi_+^{2,1}\pi_-^{3,1}\delta_-^{1,0}$	−458.669152	−459.027798	−459.324438	−459.575338	−459.787225	−459.964275	−460.109701
$\sigma^{5,4}\pi_+^{2,1}\pi_-^{2,1}\delta_-^{1,0}\phi_-^{1,0}$	−458.615815	−458.973493	−459.250135	−459.482818	−459.680186	−459.845872	−459.983737
$\sigma^{5,3}\pi_+^{2,1}\pi_-^{3,1}\delta_-^{1,0}\phi_-^{1,0}$	−457.806306	−458.314561	−458.741835	−459.124657	−459.470980	−459.784340	−460.068623
$\sigma^{6,3}\pi_+^{2,1}\pi_-^{3,1}\delta_-^{1,0}$	−457.887917	−458.352633	−458.782144	−459.141144	−459.462365	−459.747704	−460.000371

Table S498: Total energies in E_h for the Cl atom in the HGBSP2-9 basis set in fully uncontracted form, employing the real-orbital approximation.

	$0.00B_0$	$0.10B_0$	$0.20B_0$	$0.30B_0$	$0.40B_0$	$0.50B_0$	$0.60B_0$
$\sigma^{5,4}\pi_+^{2,2}\pi_-^{2,2}$	−459.489951	−459.515690	−459.494104	−459.428074	−459.321063	−459.176492	−458.997525
$\sigma^{5,5}\pi_+^{2,2}\pi_-^{2,1}$	−459.484762	−459.462498	−459.396727	−459.289949	−459.145216	−458.965598	−458.753962
$\sigma^{5,5}\pi_+^{2,1}\pi_-^{2,2}$	−459.484762	−459.562498	−459.596727	−459.589949	−459.545216	−459.465598	−459.353962
$\sigma^{5,4}\pi_+^{2,1}\pi_-^{2,2}\delta_-^{1,0}$	−459.112001	−459.347353	−459.532323	−459.681033	−459.793803	−459.872390	−459.919101
$\sigma^{5,5}\pi_+^{2,1}\pi_-^{2,1}\delta_-^{1,0}$	−459.122906	−459.320526	−459.471184	−459.585719	−459.665728	−459.713430	−459.731192
$\sigma^{5,4}\pi_+^{2,1}\pi_-^{3,1}\delta_-^{1,0}$	−458.669293	−459.027939	−459.324582	−459.575483	−459.787383	−459.964433	−460.109864
$\sigma^{5,4}\pi_+^{2,1}\pi_-^{2,1}\delta_-^{1,0}\phi_-^{1,0}$	−458.615957	−458.973635	−459.250277	−459.483126	−459.680350	−459.846336	−459.984178
$\sigma^{5,3}\pi_+^{2,1}\pi_-^{3,1}\delta_-^{1,0}\phi_-^{1,0}$	−457.806446	−458.314701	−458.741975	−459.124951	−459.471143	−459.784782	−460.069025
$\sigma^{6,3}\pi_+^{2,1}\pi_-^{3,1}\delta_-^{1,0}$	−457.888057	−458.352850	−458.783950	−459.141468	−459.462529	−459.748014	−460.000837

Table S499: Total energies in E_h for the Cl atom in the HGBSP3-5 basis set in fully uncontracted form, employing the real-orbital approximation.

	$0.00B_0$	$0.10B_0$	$0.20B_0$	$0.30B_0$	$0.40B_0$	$0.50B_0$	$0.60B_0$
$\sigma^{5,4}\pi_+^{2,2}\pi_-^{2,2}$	−459.478934	−459.504671	−459.483072	−459.416997	−459.309887	−459.165197	−458.986158
$\sigma^{5,5}\pi_+^{2,2}\pi_-^{2,1}$	−459.473745	−459.451478	−459.385696	−459.278880	−459.134070	−458.954358	−458.742658
$\sigma^{5,5}\pi_+^{2,1}\pi_-^{2,2}$	−459.473745	−459.551478	−459.585696	−459.578880	−459.534070	−459.454358	−459.342658
$\sigma^{5,4}\pi_+^{2,1}\pi_-^{2,2}\delta_-^{1,0}$	−459.100927	−459.336733	−459.522273	−459.671366	−459.784757	−459.863946	−459.911164
$\sigma^{5,5}\pi_+^{2,1}\pi_-^{2,1}\delta_-^{1,0}$	−459.111878	−459.309703	−459.460614	−459.575298	−459.655616	−459.703583	−459.721570
$\sigma^{5,4}\pi_+^{2,1}\pi_-^{3,1}\delta_-^{1,0}$	−458.658263	−459.017267	−459.315109	−459.567713	−459.781803	−459.961375	−460.109565
$\sigma^{5,4}\pi_+^{2,1}\pi_-^{2,1}\delta_-^{1,0}\phi_-^{1,0}$	−458.604851	−458.962181	−459.237950	−459.472363	−459.668202	−459.833194	−459.973187
$\sigma^{5,3}\pi_+^{2,1}\pi_-^{3,1}\delta_-^{1,0}\phi_-^{1,0}$	−457.784299	−458.303515	−458.730258	−459.115460	−459.461399	−459.775840	−460.064163
$\sigma^{6,3}\pi_+^{2,1}\pi_-^{3,1}\delta_-^{1,0}$	−457.877174	−458.341997	−458.771780	−459.133923	−459.459582	−459.750601	−460.010058

Table S500: Total energies in E_h for the Cl atom in the HGBSP3-7 basis set in fully uncontracted form, employing the real-orbital approximation.

	$0.00B_0$	$0.10B_0$	$0.20B_0$	$0.30B_0$	$0.40B_0$	$0.50B_0$	$0.60B_0$
$\sigma^{5,4}\pi_+^{2,2}\pi_-^{2,2}$	−459.489810	−459.515549	−459.493963	−459.427933	−459.320921	−459.176351	−458.997382
$\sigma^{5,5}\pi_+^{2,2}\pi_-^{2,1}$	−459.484621	−459.462357	−459.396585	−459.289805	−459.145072	−458.965456	−458.753823
$\sigma^{5,5}\pi_+^{2,1}\pi_-^{2,2}$	−459.484621	−459.562357	−459.596585	−459.589805	−459.545072	−459.465456	−459.353823
$\sigma^{5,4}\pi_+^{2,1}\pi_-^{2,2}\delta_-^{1,0}$	−459.111865	−459.347694	−459.533186	−459.682415	−459.795747	−459.874998	−459.922428
$\sigma^{5,5}\pi_+^{2,1}\pi_-^{2,1}\delta_-^{1,0}$	−459.122812	−459.320647	−459.471528	−459.586287	−459.666559	−459.714605	−459.732771
$\sigma^{5,4}\pi_+^{2,1}\pi_-^{3,1}\delta_-^{1,0}$	−458.669219	−459.028217	−459.326048	−459.578690	−459.792755	−459.972364	−460.120695
$\sigma^{5,4}\pi_+^{2,1}\pi_-^{2,1}\delta_-^{1,0}\phi_-^{1,0}$	−458.615820	−458.973517	−459.250276	−459.483212	−459.680950	−459.847110	−459.985545
$\sigma^{5,3}\pi_+^{2,1}\pi_-^{3,1}\delta_-^{1,0}\phi_-^{1,0}$	−457.806419	−458.314789	−458.742499	−459.126251	−459.474053	−459.789433	−460.076236
$\sigma^{6,3}\pi_+^{2,1}\pi_-^{3,1}\delta_-^{1,0}$	−457.888048	−458.352888	−458.783042	−459.144808	−459.470474	−459.761729	−460.021570

Table S501: Total energies in E_h for the Cl atom in the HGBSP3-9 basis set in fully uncontracted form, employing the real-orbital approximation.

	$0.00B_0$	$0.10B_0$	$0.20B_0$	$0.30B_0$	$0.40B_0$	$0.50B_0$	$0.60B_0$
$\sigma^{5,4}\pi_+^{2,2}\pi_-^{2,2}$	−459.489951	−459.515691	−459.494105	−459.428077	−459.321068	−459.176505	−458.997553
$\sigma^{5,5}\pi_+^{2,2}\pi_-^{2,1}$	−459.484762	−459.462498	−459.396727	−459.289949	−459.145219	−458.965608	−458.753988
$\sigma^{5,5}\pi_+^{2,1}\pi_-^{2,2}$	−459.484762	−459.562498	−459.596727	−459.589949	−459.545219	−459.465608	−459.353988
$\sigma^{5,4}\pi_+^{2,1}\pi_-^{2,2}\delta_-^{1,0}$	−459.112007	−459.347840	−459.533336	−459.682559	−459.795909	−459.875153	−459.922595
$\sigma^{5,5}\pi_+^{2,1}\pi_-^{2,1}\delta_-^{1,0}$	−459.122954	−459.320789	−459.471673	−459.586432	−459.666714	−459.714755	−459.732934
$\sigma^{5,4}\pi_+^{2,1}\pi_-^{3,1}\delta_-^{1,0}$	−458.669360	−459.028358	−459.326191	−459.578835	−459.792908	−459.972518	−460.120859
$\sigma^{5,4}\pi_+^{2,1}\pi_-^{2,1}\delta_-^{1,0}\phi_-^{1,0}$	−458.615962	−458.973659	−459.250418	−459.483520	−459.681114	−459.847574	−459.985986
$\sigma^{5,3}\pi_+^{2,1}\pi_-^{3,1}\delta_-^{1,0}\phi_-^{1,0}$	−457.795349	−458.314929	−458.742639	−459.126543	−459.474215	−459.789873	−460.076635
$\sigma^{6,3}\pi_+^{2,1}\pi_-^{3,1}\delta_-^{1,0}$	−457.888188	−458.353104	−458.785041	−459.145376	−459.470712	−459.761900	−460.021773

Table S502: Total energies in E_h for the Cl atom in the AHGBSP1-5 basis set in fully uncontracted form, employing the real-orbital approximation.

	$0.00B_0$	$0.10B_0$	$0.20B_0$	$0.30B_0$	$0.40B_0$	$0.50B_0$	$0.60B_0$
$\sigma^{5,4}\pi_+^{2,2}\pi_-^{2,2}$	−459.476554	−459.502127	−459.479914	−459.412513	−459.303097	−459.154896	−458.970981
$\sigma^{5,5}\pi_+^{2,2}\pi_-^{2,1}$	−459.473086	−459.450870	−459.385077	−459.277835	−459.131805	−458.949753	−458.734346
$\sigma^{5,5}\pi_+^{2,1}\pi_-^{2,2}$	−459.473086	−459.550870	−459.585077	−459.577835	−459.531805	−459.449753	−459.334346
$\sigma^{5,4}\pi_+^{2,1}\pi_-^{2,2}\delta_-^{1,0}$	−459.100169	−459.335321	−459.519923	−459.667612	−459.778839	−459.854815	−459.897619
$\sigma^{5,5}\pi_+^{2,1}\pi_-^{2,1}\delta_-^{1,0}$	−459.109412	−459.307059	−459.457726	−459.571962	−459.651267	−459.697282	−459.712119
$\sigma^{5,4}\pi_+^{2,1}\pi_-^{3,1}\delta_-^{1,0}$	−458.657556	−459.016138	−459.312529	−459.562939	−459.774112	−459.949878	−460.093196
$\sigma^{5,4}\pi_+^{2,1}\pi_-^{2,1}\delta_-^{1,0}\phi_-^{1,0}$							
$\sigma^{5,3}\pi_+^{2,1}\pi_-^{3,1}\delta_-^{1,0}\phi_-^{1,0}$							
$\sigma^{6,3}\pi_+^{2,1}\pi_-^{3,1}\delta_-^{1,0}$	−457.875969	−458.340790	−458.771720	−459.129325	−459.449652	−459.733614	−459.984068

Table S503: Total energies in E_h for the Cl atom in the AHGBSP1-7 basis set in fully uncontracted form, employing the real-orbital approximation.

	$0.00B_0$	$0.10B_0$	$0.20B_0$	$0.30B_0$	$0.40B_0$	$0.50B_0$	$0.60B_0$
$\sigma^{5,4}\pi_+^{2,2}\pi_-^{2,2}$	−459.487426	−459.513001	−459.490798	−459.423436	−459.314111	−459.166030	−458.982187
$\sigma^{5,5}\pi_+^{2,2}\pi_-^{2,1}$	−459.483959	−459.461744	−459.395961	−459.288749	−459.142788	−458.960832	−458.745504
$\sigma^{5,5}\pi_+^{2,1}\pi_-^{2,2}$	−459.483959	−459.561744	−459.595961	−459.588749	−459.542788	−459.460832	−459.345504
$\sigma^{5,4}\pi_+^{2,1}\pi_-^{2,2}\delta_-^{1,0}$	−459.111095	−459.346276	−459.530827	−459.678665	−459.789806	−459.865854	−459.908898
$\sigma^{5,5}\pi_+^{2,1}\pi_-^{2,1}\delta_-^{1,0}$	−459.120335	−459.317996	−459.468634	−459.582950	−459.662191	−459.708293	−459.723336
$\sigma^{5,4}\pi_+^{2,1}\pi_-^{3,1}\delta_-^{1,0}$	−458.668506	−459.027083	−459.323463	−459.573929	−459.785057	−459.960843	−460.104340
$\sigma^{5,4}\pi_+^{2,1}\pi_-^{2,1}\delta_-^{1,0}\phi_-^{1,0}$							
$\sigma^{5,3}\pi_+^{2,1}\pi_-^{3,1}\delta_-^{1,0}\phi_-^{1,0}$							
$\sigma^{6,3}\pi_+^{2,1}\pi_-^{3,1}\delta_-^{1,0}$	−457.886840	−458.351668	−458.782799	−459.140237	−459.460629	−459.744930	−459.995898

Table S504: Total energies in E_h for the Cl atom in the AHGBSP1-9 basis set in fully uncontracted form, employing the real-orbital approximation.

	$0.00B_0$	$0.10B_0$	$0.20B_0$	$0.30B_0$	$0.40B_0$	$0.50B_0$	$0.60B_0$
$\sigma^{5,4}\pi_+^{2,2}\pi_-^{2,2}$	−459.487567	−459.513142	−459.490939	−459.423578	−459.314255	−459.166179	−458.982356
$\sigma^{5,5}\pi_+^{2,2}\pi_-^{2,1}$	−459.484099	−459.461885	−459.396102	−459.288891	−459.142932	−458.960981	−458.745665
$\sigma^{5,5}\pi_+^{2,1}\pi_-^{2,2}$	−459.484099	−459.561885	−459.596102	−459.588891	−459.542932	−459.460981	−459.345665
$\sigma^{5,4}\pi_+^{2,1}\pi_-^{2,2}\delta_-^{1,0}$	−459.111236	−459.346421	−459.530978	−459.678808	−459.789969	−459.866005	−459.909062
$\sigma^{5,5}\pi_+^{2,1}\pi_-^{2,1}\delta_-^{1,0}$	−459.120476	−459.318137	−459.468779	−459.583094	−459.662347	−459.708441	−459.723495
$\sigma^{5,4}\pi_+^{2,1}\pi_-^{3,1}\delta_-^{1,0}$	−458.668647	−459.027224	−459.323606	−459.574073	−459.785213	−459.960999	−460.104502
$\sigma^{5,4}\pi_+^{2,1}\pi_-^{2,1}\delta_-^{1,0}\phi_-^{1,0}$							
$\sigma^{5,3}\pi_+^{2,1}\pi_-^{3,1}\delta_-^{1,0}\phi_-^{1,0}$							
$\sigma^{6,3}\pi_+^{2,1}\pi_-^{3,1}\delta_-^{1,0}$	−457.886978	−458.351807	−458.782949	−459.140384	−459.460788	−459.745122	−459.996144

Table S505: Total energies in E_h for the Cl atom in the AHGBSP2-5 basis set in fully uncontracted form, employing the real-orbital approximation.

	$0.00B_0$	$0.10B_0$	$0.20B_0$	$0.30B_0$	$0.40B_0$	$0.50B_0$	$0.60B_0$
$\sigma^{5,4}\pi_+^{2,2}\pi_-^{2,2}$	−459.478939	−459.504676	−459.483078	−459.417010	−459.309913	−459.165237	−458.986202
$\sigma^{5,5}\pi_+^{2,2}\pi_-^{2,1}$	−459.473750	−459.451484	−459.385703	−459.278893	−459.134094	−458.954392	−458.742692
$\sigma^{5,5}\pi_+^{2,1}\pi_-^{2,2}$	−459.473750	−459.551484	−459.585703	−459.578893	−459.534094	−459.454392	−459.342692
$\sigma^{5,4}\pi_+^{2,1}\pi_-^{2,2}\delta_-^{1,0}$	−459.100934	−459.336253	−459.521268	−459.669839	−459.782678	−459.861212	−459.907683
$\sigma^{5,5}\pi_+^{2,1}\pi_-^{2,1}\delta_-^{1,0}$	−459.111843	−459.309448	−459.460131	−459.574589	−459.654652	−459.702281	−459.719843
$\sigma^{5,4}\pi_+^{2,1}\pi_-^{3,1}\delta_-^{1,0}$	−458.658203	−459.016855	−459.313506	−459.564350	−459.776286	−459.953317	−460.098563
$\sigma^{5,4}\pi_+^{2,1}\pi_-^{2,1}\delta_-^{1,0}\phi_-^{1,0}$	−458.604855	−458.962183	−459.237837	−459.471982	−459.667464	−459.831981	−459.971391
$\sigma^{5,3}\pi_+^{2,1}\pi_-^{3,1}\delta_-^{1,0}\phi_-^{1,0}$	−457.795399	−458.303306	−458.729615	−459.113873	−459.458346	−459.770742	−460.056516
$\sigma^{6,3}\pi_+^{2,1}\pi_-^{3,1}\delta_-^{1,0}$	−457.877049	−458.341835	−458.772719	−459.130413	−459.451394	−459.736515	−459.988783

Table S506: Total energies in E_h for the Cl atom in the AHGBSP2-7 basis set in fully uncontracted form, employing the real-orbital approximation.

	$0.00B_0$	$0.10B_0$	$0.20B_0$	$0.30B_0$	$0.40B_0$	$0.50B_0$	$0.60B_0$
$\sigma^{5,4}\pi_+^{2,2}\pi_-^{2,2}$	−459.489810	−459.515549	−459.493962	−459.427932	−459.320919	−459.176343	−458.997359
$\sigma^{5,5}\pi_+^{2,2}\pi_-^{2,1}$	−459.484622	−459.462357	−459.396586	−459.289807	−459.145072	−458.965450	−458.753802
$\sigma^{5,5}\pi_+^{2,1}\pi_-^{2,2}$	−459.484622	−459.562357	−459.596586	−459.589807	−459.545072	−459.465450	−459.353802
$\sigma^{5,4}\pi_+^{2,1}\pi_-^{2,2}\delta_-^{1,0}$	−459.111860	−459.347208	−459.532172	−459.680890	−459.793640	−459.872238	−459.918939
$\sigma^{5,5}\pi_+^{2,1}\pi_-^{2,1}\delta_-^{1,0}$	−459.122765	−459.320384	−459.471039	−459.585575	−459.665573	−459.713283	−459.731033
$\sigma^{5,4}\pi_+^{2,1}\pi_-^{3,1}\delta_-^{1,0}$	−458.669153	−459.027798	−459.324439	−459.575339	−459.787227	−459.964278	−460.109704
$\sigma^{5,4}\pi_+^{2,1}\pi_-^{2,1}\delta_-^{1,0}\phi_-^{1,0}$	−458.615815	−458.973494	−459.250135	−459.482821	−459.680187	−459.845875	−459.983739
$\sigma^{5,3}\pi_+^{2,1}\pi_-^{3,1}\delta_-^{1,0}\phi_-^{1,0}$	−457.806307	−458.314561	−458.741835	−459.124660	−459.470980	−459.784342	−460.068624
$\sigma^{6,3}\pi_+^{2,1}\pi_-^{3,1}\delta_-^{1,0}$	−457.887919	−458.352713	−458.783802	−459.141323	−459.462370	−459.747827	−460.000600

Table S507: Total energies in E_h for the Cl atom in the AHGBSP2-9 basis set in fully uncontracted form, employing the real-orbital approximation.

	$0.00B_0$	$0.10B_0$	$0.20B_0$	$0.30B_0$	$0.40B_0$	$0.50B_0$	$0.60B_0$
$\sigma^{5,4}\pi_+^{2,2}\pi_-^{2,2}$	−459.489951	−459.515690	−459.494104	−459.428074	−459.321063	−459.176492	−458.997525
$\sigma^{5,5}\pi_+^{2,2}\pi_-^{2,1}$	−459.484762	−459.462498	−459.396727	−459.289949	−459.145216	−458.965598	−458.753962
$\sigma^{5,5}\pi_+^{2,1}\pi_-^{2,2}$	−459.484762	−459.562498	−459.596727	−459.589949	−459.545216	−459.465598	−459.353962
$\sigma^{5,4}\pi_+^{2,1}\pi_-^{2,2}\delta_-^{1,0}$	−459.112001	−459.347353	−459.532323	−459.681033	−459.793803	−459.872390	−459.919101
$\sigma^{5,5}\pi_+^{2,1}\pi_-^{2,1}\delta_-^{1,0}$	−459.122906	−459.320526	−459.471184	−459.585719	−459.665728	−459.713430	−459.731192
$\sigma^{5,4}\pi_+^{2,1}\pi_-^{3,1}\delta_-^{1,0}$	−458.669293	−459.027939	−459.324582	−459.575483	−459.787383	−459.964433	−460.109864
$\sigma^{5,4}\pi_+^{2,1}\pi_-^{2,1}\delta_-^{1,0}\phi_-^{1,0}$	−458.615957	−458.973636	−459.250277	−459.483127	−459.680350	−459.846336	−459.984178
$\sigma^{5,3}\pi_+^{2,1}\pi_-^{3,1}\delta_-^{1,0}\phi_-^{1,0}$	−457.806446	−458.314701	−458.741975	−459.124951	−459.471143	−459.784782	−460.069026
$\sigma^{6,3}\pi_+^{2,1}\pi_-^{3,1}\delta_-^{1,0}$	−457.888057	−458.352852	−458.783953	−459.141470	−459.462529	−459.748018	−460.000844

Table S508: Total energies in E_h for the Cl atom in the AHGBSP3-5 basis set in fully uncontracted form, employing the real-orbital approximation.

	$0.00B_0$	$0.10B_0$	$0.20B_0$	$0.30B_0$	$0.40B_0$	$0.50B_0$	$0.60B_0$
$\sigma^{5,4}\pi_+^{2,2}\pi_-^{2,2}$	−459.478939	−459.504676	−459.483079	−459.417012	−459.309918	−459.165250	−458.986230
$\sigma^{5,5}\pi_+^{2,2}\pi_-^{2,1}$	−459.473749	−459.451483	−459.385703	−459.278893	−459.134096	−458.954402	−458.742718
$\sigma^{5,5}\pi_+^{2,1}\pi_-^{2,2}$	−459.473749	−459.551483	−459.585703	−459.578893	−459.534096	−459.454402	−459.342718
$\sigma^{5,4}\pi_+^{2,1}\pi_-^{2,2}\delta_-^{1,0}$	−459.100940	−459.336746	−459.522282	−459.671381	−459.784780	−459.863984	−459.911218
$\sigma^{5,5}\pi_+^{2,1}\pi_-^{2,1}\delta_-^{1,0}$	−459.111890	−459.309713	−459.460621	−459.575311	−459.655636	−459.703614	−459.721615
$\sigma^{5,4}\pi_+^{2,1}\pi_-^{3,1}\delta_-^{1,0}$	−458.658269	−459.017274	−459.315115	−459.567723	−459.781819	−459.961400	−460.109601
$\sigma^{5,4}\pi_+^{2,1}\pi_-^{2,1}\delta_-^{1,0}\phi_-^{1,0}$	−458.604860	−458.962206	−459.237979	−459.472376	−459.668225	−459.833226	−459.973218
$\sigma^{5,3}\pi_+^{2,1}\pi_-^{3,1}\delta_-^{1,0}\phi_-^{1,0}$	−457.795510	−458.303535	−458.730280	−459.115465	−459.461412	−459.775858	−460.064176
$\sigma^{6,3}\pi_+^{2,1}\pi_-^{3,1}\delta_-^{1,0}$	−457.877179	−458.342088	−458.773846	−459.134316	−459.459621	−459.750628	−460.010210

Table S509: Total energies in E_h for the Cl atom in the AHGBSP3-7 basis set in fully uncontracted form, employing the real-orbital approximation.

	$0.00B_0$	$0.10B_0$	$0.20B_0$	$0.30B_0$	$0.40B_0$	$0.50B_0$	$0.60B_0$
$\sigma^{5,4}\pi_+^{2,2}\pi_-^{2,2}$	−459.489811	−459.515550	−459.493964	−459.427935	−459.320925	−459.176356	−458.997388
$\sigma^{5,5}\pi_+^{2,2}\pi_-^{2,1}$	−459.484622	−459.462357	−459.396586	−459.289807	−459.145075	−458.965460	−458.753828
$\sigma^{5,5}\pi_+^{2,1}\pi_-^{2,2}$	−459.484622	−459.562357	−459.596586	−459.589807	−459.545075	−459.465460	−459.353828
$\sigma^{5,4}\pi_+^{2,1}\pi_-^{2,2}\delta_-^{1,0}$	−459.111866	−459.347695	−459.533187	−459.682416	−459.795750	−459.875002	−459.922433
$\sigma^{5,5}\pi_+^{2,1}\pi_-^{2,1}\delta_-^{1,0}$	−459.122813	−459.320647	−459.471528	−459.586288	−459.666561	−459.714608	−459.732775
$\sigma^{5,4}\pi_+^{2,1}\pi_-^{3,1}\delta_-^{1,0}$	−458.669219	−459.028217	−459.326049	−459.578691	−459.792756	−459.972367	−460.120699
$\sigma^{5,4}\pi_+^{2,1}\pi_-^{2,1}\delta_-^{1,0}\phi_-^{1,0}$	−458.615821	−458.973518	−459.250277	−459.483215	−459.680951	−459.847113	−459.985548
$\sigma^{5,3}\pi_+^{2,1}\pi_-^{3,1}\delta_-^{1,0}\phi_-^{1,0}$	−457.806419	−458.314789	−458.742499	−459.126253	−459.474053	−459.789434	−460.076238
$\sigma^{6,3}\pi_+^{2,1}\pi_-^{3,1}\delta_-^{1,0}$	−457.888049	−458.352967	−458.784894	−459.145232	−459.470558	−459.761733	−460.021589

Table S510: Total energies in E_h for the Cl atom in the AHGBSP3-9 basis set in fully uncontracted form, employing the real-orbital approximation.

	$0.00B_0$	$0.10B_0$	$0.20B_0$	$0.30B_0$	$0.40B_0$	$0.50B_0$	$0.60B_0$
$\sigma^{5,4}\pi_+^{2,2}\pi_-^{2,2}$	−459.489951	−459.515691	−459.494105	−459.428077	−459.321068	−459.176505	−458.997553
$\sigma^{5,5}\pi_+^{2,2}\pi_-^{2,1}$	−459.484762	−459.462498	−459.396727	−459.289949	−459.145219	−458.965608	−458.753988
$\sigma^{5,5}\pi_+^{2,1}\pi_-^{2,2}$	−459.484762	−459.562498	−459.596727	−459.589949	−459.545219	−459.465608	−459.353988
$\sigma^{5,4}\pi_+^{2,1}\pi_-^{2,2}\delta_-^{1,0}$	−459.112007	−459.347840	−459.533336	−459.682559	−459.795909	−459.875153	−459.922595
$\sigma^{5,5}\pi_+^{2,1}\pi_-^{2,1}\delta_-^{1,0}$	−459.122954	−459.320789	−459.471673	−459.586432	−459.666714	−459.714755	−459.732934
$\sigma^{5,4}\pi_+^{2,1}\pi_-^{3,1}\delta_-^{1,0}$	−458.669360	−459.028358	−459.326191	−459.578835	−459.792909	−459.972518	−460.120859
$\sigma^{5,4}\pi_+^{2,1}\pi_-^{2,1}\delta_-^{1,0}\phi_-^{1,0}$	−458.615962	−458.973660	−459.250418	−459.483520	−459.681114	−459.847574	−459.985986
$\sigma^{5,3}\pi_+^{2,1}\pi_-^{3,1}\delta_-^{1,0}\phi_-^{1,0}$	−457.806558	−458.314929	−458.742639	−459.126543	−459.474215	−459.789873	−460.076635
$\sigma^{6,3}\pi_+^{2,1}\pi_-^{3,1}\delta_-^{1,0}$	−457.934421	−458.390768	−458.785041	−459.145376	−459.470712	−459.761900	−460.021773

Table S511: Total energies in E_h for the Cl atom in the 6-311++G(3df,3pd) basis set in fully uncontracted form, employing the real-orbital approximation.

	$0.00B_0$	$0.10B_0$	$0.20B_0$	$0.30B_0$	$0.40B_0$	$0.50B_0$	$0.60B_0$
$\sigma^{5,4}\pi_+^{2,2}\pi_-^{2,2}$	−459.478704	−459.504207	−459.481715	−459.413781	−459.303770	−459.155337	−458.972024
$\sigma^{5,5}\pi_+^{2,2}\pi_-^{2,1}$	−459.473875	−459.451487	−459.385134	−459.276918	−459.129653	−458.946430	−458.730301
$\sigma^{5,5}\pi_+^{2,1}\pi_-^{2,2}$	−459.473875	−459.551487	−459.585134	−459.576918	−459.529653	−459.446430	−459.330301
$\sigma^{5,4}\pi_+^{2,1}\pi_-^{2,2}\delta_-^{1,0}$	−458.988428	−459.261326	−459.480837	−459.649217	−459.769796	−459.846697	−459.884607
$\sigma^{5,5}\pi_+^{2,1}\pi_-^{2,1}\delta_-^{1,0}$	−459.028277	−459.253390	−459.429398	−459.558204	−459.642734	−459.686715	−459.694465
$\sigma^{5,4}\pi_+^{2,1}\pi_-^{3,1}\delta_-^{1,0}$	−458.631871	−458.945580	−459.265381	−459.533815	−459.753856	−459.929395	−460.065035
$\sigma^{5,4}\pi_+^{2,1}\pi_-^{2,1}\delta_-^{1,0}\phi_-^{1,0}$	−456.783626	−457.257664	−457.680415	−458.053699	−458.380292	−458.663714	−458.907990
$\sigma^{5,3}\pi_+^{2,1}\pi_-^{3,1}\delta_-^{1,0}\phi_-^{1,0}$	−456.048771	−456.671714	−457.241131	−457.758737	−458.227248	−458.650201	−459.031734
$\sigma^{6,3}\pi_+^{2,1}\pi_-^{3,1}\delta_-^{1,0}$	−457.885502	−458.346140	−458.732157	−459.080451	−459.402632	−459.683410	−459.925754

Table S512: Total energies in E_h for the Cl atom in the def2-TZVP basis set in fully uncontracted form, employing the real-orbital approximation.

	$0.00B_0$	$0.10B_0$	$0.20B_0$	$0.30B_0$	$0.40B_0$	$0.50B_0$	$0.60B_0$
$\sigma^{5,4}\pi_+^{2,2}\pi_-^{2,2}$	−459.479367	−459.505134	−459.483193	−459.415687	−459.305774	−459.157122	−458.973414
$\sigma^{5,5}\pi_+^{2,2}\pi_-^{2,1}$	−459.474496	−459.452319	−459.386418	−459.278577	−459.131431	−458.948068	−458.731640
$\sigma^{5,5}\pi_+^{2,1}\pi_-^{2,2}$	−459.474496	−459.552319	−459.586418	−459.578577	−459.531431	−459.448068	−459.331640
$\sigma^{5,4}\pi_+^{2,1}\pi_-^{2,2}\delta_-^{1,0}$	−458.878375	−459.154177	−459.382199	−459.564178	−459.702689	−459.800763	−459.861538
$\sigma^{5,5}\pi_+^{2,1}\pi_-^{2,1}\delta_-^{1,0}$	−458.929678	−459.157718	−459.342298	−459.484726	−459.586975	−459.651441	−459.680719
$\sigma^{5,4}\pi_+^{2,1}\pi_-^{3,1}\delta_-^{1,0}$	−458.355856	−458.725146	−459.033358	−459.281500	−459.471188	−459.846049	−460.016901
$\sigma^{5,4}\pi_+^{2,1}\pi_-^{2,1}\delta_-^{1,0}\phi_-^{1,0}$	−456.678823	−457.155694	−457.586738	−457.973174	−458.316822	−458.619858	−458.884599
$\sigma^{5,3}\pi_+^{2,1}\pi_-^{3,1}\delta_-^{1,0}\phi_-^{1,0}$	−455.821703	−456.440238	−456.996138	−457.490273	−458.124609	−458.576632	−458.989252
$\sigma^{6,3}\pi_+^{2,1}\pi_-^{3,1}\delta_-^{1,0}$	−457.429889	−457.896537	−458.296764	−458.631419	−459.148408	−459.433992	−459.847589

Table S513: Total energies in E_h for the Ar atom in the cc-pVDZ basis set in fully uncontracted form, employing the real-orbital approximation.

	$0.00B_0$	$0.10B_0$	$0.20B_0$	$0.30B_0$	$0.40B_0$	$0.50B_0$	$0.60B_0$
$\sigma^{5,5}\pi_+^{2,2}\pi_-^{2,2}$	−526.799865	−526.778392	−526.714302	−526.608551	−526.462661	−526.278602	−526.058660
$\sigma^{6,4}\pi_+^{2,2}\pi_-^{2,2}$	−525.936802	−526.013943	−526.045655	−526.032781	−525.976653	−525.878976	−525.741701
$\sigma^{6,5}\pi_+^{2,2}\pi_-^{2,1}$	−525.893481	−525.922263	−525.908850	−525.853949	−525.758670	−525.624440	−525.452892
$\sigma^{6,5}\pi_+^{2,1}\pi_-^{2,2}$	−525.893481	−526.022263	−526.108850	−526.153949	−526.158670	−526.124440	−526.052892
$\sigma^{5,5}\pi_+^{2,1}\pi_-^{2,2}\delta_-^{1,0}$	−525.703709	−525.933048	−526.121338	−526.269376	−526.378426	−526.450121	−526.486349
$\sigma^{5,4}\pi_+^{2,1}\pi_-^{3,2}\delta_-^{1,0}$	−524.826478	−525.200382	−525.522300	−525.792841	−526.012964	−526.183909	−526.307118
$\sigma^{5,4}\pi_+^{2,1}\pi_-^{2,2}\delta_-^{1,0}\phi_-^{1,0}$							
$\sigma^{5,4}\pi_+^{2,1}\pi_-^{3,1}\delta_-^{1,0}\phi_-^{1,0}$							
$\sigma^{6,4}\pi_+^{2,1}\pi_-^{3,1}\delta_-^{1,0}$	−524.097646	−524.521300	−524.892371	−525.211182	−525.478254	−525.694280	−525.860098

Table S514: Total energies in E_h for the Ar atom in the cc-pVTZ basis set in fully uncontracted form, employing the real-orbital approximation.

	$0.00B_0$	$0.10B_0$	$0.20B_0$	$0.30B_0$	$0.40B_0$	$0.50B_0$	$0.60B_0$
$\sigma^{5,5}\pi_+^{2,2}\pi_-^{2,2}$	−526.813134	−526.791546	−526.727234	−526.621486	−526.476274	−526.294015	−526.077319
$\sigma^{6,4}\pi_+^{2,2}\pi_-^{2,2}$	−526.093566	−526.169883	−526.199195	−526.182536	−526.121476	−526.017932	−525.873969
$\sigma^{6,5}\pi_+^{2,2}\pi_-^{2,1}$	−526.057057	−526.084919	−526.068804	−526.009577	−525.908550	−525.767340	−525.587705
$\sigma^{6,5}\pi_+^{2,1}\pi_-^{2,2}$	−526.057057	−526.184919	−526.268804	−526.309577	−526.308550	−526.267340	−526.187705
$\sigma^{5,5}\pi_+^{2,1}\pi_-^{2,2}\delta_-^{1,0}$	−526.033823	−526.262191	−526.447660	−526.591277	−526.694650	−526.759768	−526.788819
$\sigma^{5,4}\pi_+^{2,1}\pi_-^{3,2}\delta_-^{1,0}$	−525.283772	−525.654844	−525.986005	−526.276573	−526.525560	−526.734843	−526.906469
$\sigma^{5,4}\pi_+^{2,1}\pi_-^{2,2}\delta_-^{1,0}\phi_-^{1,0}$	−523.152903	−523.630164	−524.062324	−524.450459	−524.796205	−525.101559	−525.368668
$\sigma^{5,4}\pi_+^{2,1}\pi_-^{3,1}\delta_-^{1,0}\phi_-^{1,0}$	−522.561932	−523.133496	−523.648390	−524.107214	−524.510940	−524.860887	−525.413470
$\sigma^{6,4}\pi_+^{2,1}\pi_-^{3,1}\delta_-^{1,0}$	−524.623807	−525.043581	−525.403094	−525.702916	−525.943992	−526.127632	−526.565285

Table S515: Total energies in E_h for the Ar atom in the cc-pVQZ basis set in fully uncontracted form, employing the real-orbital approximation.

	$0.00B_0$	$0.10B_0$	$0.20B_0$	$0.30B_0$	$0.40B_0$	$0.50B_0$	$0.60B_0$
$\sigma^{5,5}\pi_+^{2,2}\pi_-^{2,2}$	−526.816780	−526.795150	−526.730830	−526.625404	−526.481149	−526.300646	−526.086467
$\sigma^{6,4}\pi_+^{2,2}\pi_-^{2,2}$	−526.208894	−526.283880	−526.309260	−526.286231	−526.230551	−526.150475	−526.035881
$\sigma^{6,5}\pi_+^{2,2}\pi_-^{2,1}$	−526.180786	−526.207219	−526.186877	−526.120775	−526.013956	−525.894274	−525.743111
$\sigma^{6,5}\pi_+^{2,1}\pi_-^{2,2}$	−526.180786	−526.307219	−526.386877	−526.420775	−526.413956	−526.394274	−526.343111
$\sigma^{5,5}\pi_+^{2,1}\pi_-^{2,2}\delta_-^{1,0}$	−526.139258	−526.366672	−526.549381	−526.688706	−526.786610	−526.845450	−526.867772
$\sigma^{5,4}\pi_+^{2,1}\pi_-^{3,2}\delta_-^{1,0}$	−525.474110	−525.850497	−526.180090	−526.464113	−526.704396	−526.903174	−527.062907
$\sigma^{5,4}\pi_+^{2,1}\pi_-^{2,2}\delta_-^{1,0}\phi_-^{1,0}$	−524.155684	−524.630827	−525.056668	−525.434373	−525.765668	−526.052630	−526.297511
$\sigma^{5,4}\pi_+^{2,1}\pi_-^{3,1}\delta_-^{1,0}\phi_-^{1,0}$	−523.596777	−524.163036	−524.662222	−525.095586	−525.640158	−526.030219	−526.378032
$\sigma^{6,4}\pi_+^{2,1}\pi_-^{3,1}\delta_-^{1,0}$	−524.871531	−525.286468	−525.631738	−525.908743	−526.332013	−526.659474	−526.884902

Table S516: Total energies in E_h for the Ar atom in the cc-pV5Z basis set in fully uncontracted form, employing the real-orbital approximation.

	$0.00B_0$	$0.10B_0$	$0.20B_0$	$0.30B_0$	$0.40B_0$	$0.50B_0$	$0.60B_0$
$\sigma^{5,5}\pi_+^{2,2}\pi_-^{2,2}$	−526.817342	−526.795707	−526.731429	−526.626218	−526.482444	−526.302697	−526.089501
$\sigma^{6,4}\pi_+^{2,2}\pi_-^{2,2}$	−526.247603	−526.320905	−526.342051	−526.316486	−526.258243	−526.172707	−526.056037
$\sigma^{6,5}\pi_+^{2,2}\pi_-^{2,1}$	−526.241591	−526.266147	−526.240665	−526.168245	−526.057127	−525.921741	−525.764360
$\sigma^{6,5}\pi_+^{2,1}\pi_-^{2,2}$	−526.241591	−526.366147	−526.440665	−526.468245	−526.457127	−526.421741	−526.364360
$\sigma^{5,5}\pi_+^{2,1}\pi_-^{2,2}\delta_-^{1,0}$	−526.151950	−526.379497	−526.562656	−526.702856	−526.802178	−526.863065	−526.888119
$\sigma^{5,4}\pi_+^{2,1}\pi_-^{3,2}\delta_-^{1,0}$	−525.553577	−525.918936	−526.215708	−526.494033	−526.736428	−526.938158	−527.101766
$\sigma^{5,4}\pi_+^{2,1}\pi_-^{2,2}\delta_-^{1,0}\phi_-^{1,0}$	−524.588327	−525.061889	−525.482993	−525.852817	−526.173118	−526.446036	−526.673956
$\sigma^{5,4}\pi_+^{2,1}\pi_-^{3,1}\delta_-^{1,0}\phi_-^{1,0}$	−524.030443	−524.593215	−525.082214	−525.499562	−526.049918	−526.426945	−526.759298
$\sigma^{6,4}\pi_+^{2,1}\pi_-^{3,1}\delta_-^{1,0}$	−525.020643	−525.432228	−525.766390	−526.026949	−526.453441	−526.717470	−526.945741

Table S517: Total energies in E_h for the Ar atom in the aug-cc-pVDZ basis set in fully uncontracted form, employing the real-orbital approximation.

	$0.00B_0$	$0.10B_0$	$0.20B_0$	$0.30B_0$	$0.40B_0$	$0.50B_0$	$0.60B_0$
$\sigma^{5,5}\pi_+^{2,2}\pi_-^{2,2}$	−526.801012	−526.779308	−526.714765	−526.608837	−526.463418	−526.280598	−526.062593
$\sigma^{6,4}\pi_+^{2,2}\pi_-^{2,2}$	−526.364883	−526.433136	−526.439000	−526.386394	−526.285513	−526.158833	−526.017826
$\sigma^{6,5}\pi_+^{2,2}\pi_-^{2,1}$	−526.361218	−526.380806	−526.340466	−526.243284	−526.096769	−525.920216	−525.735556
$\sigma^{6,5}\pi_+^{2,1}\pi_-^{2,2}$	−526.361218	−526.480806	−526.540466	−526.543284	−526.496769	−526.420216	−526.335556
$\sigma^{5,5}\pi_+^{2,1}\pi_-^{2,2}\delta_-^{1,0}$	−526.121409	−526.347616	−526.526700	−526.659930	−526.749178	−526.796776	−526.805440
$\sigma^{5,4}\pi_+^{2,1}\pi_-^{3,2}\delta_-^{1,0}$	−525.597923	−525.950786	−526.211849	−526.408195	−526.635558	−526.817885	−526.958029
$\sigma^{5,4}\pi_+^{2,1}\pi_-^{2,2}\delta_-^{1,0}\phi_-^{1,0}$							
$\sigma^{5,4}\pi_+^{2,1}\pi_-^{3,1}\delta_-^{1,0}\phi_-^{1,0}$							
$\sigma^{6,4}\pi_+^{2,1}\pi_-^{3,1}\delta_-^{1,0}$	−525.020433	−525.436772	−525.786947	−526.075504	−526.317169	−526.538626	−526.735472

Table S518: Total energies in E_h for the Ar atom in the aug-cc-pVTZ basis set in fully uncontracted form, employing the real-orbital approximation.

	$0.00B_0$	$0.10B_0$	$0.20B_0$	$0.30B_0$	$0.40B_0$	$0.50B_0$	$0.60B_0$
$\sigma^{5,5}\pi_+^{2,2}\pi_-^{2,2}$	−526.813355	−526.791689	−526.727326	−526.621923	−526.477715	−526.297229	−526.083095
$\sigma^{6,4}\pi_+^{2,2}\pi_-^{2,2}$	−526.389256	−526.456829	−526.462232	−526.415687	−526.336883	−526.235123	−526.105489
$\sigma^{6,5}\pi_+^{2,2}\pi_-^{2,1}$	−526.383546	−526.402072	−526.359857	−526.265223	−526.136807	−525.990378	−525.821887
$\sigma^{6,5}\pi_+^{2,1}\pi_-^{2,2}$	−526.383546	−526.502072	−526.559857	−526.565223	−526.536807	−526.490378	−526.421887
$\sigma^{5,5}\pi_+^{2,1}\pi_-^{2,2}\delta_-^{1,0}$	−526.217128	−526.441151	−526.614120	−526.738629	−526.818696	−526.859274	−526.865483
$\sigma^{5,4}\pi_+^{2,1}\pi_-^{3,2}\delta_-^{1,0}$	−525.683817	−526.033338	−526.286911	−526.549074	−526.763566	−526.934814	−527.068401
$\sigma^{5,4}\pi_+^{2,1}\pi_-^{2,2}\delta_-^{1,0}\phi_-^{1,0}$	−524.578881	−525.049680	−525.462889	−525.820840	−526.127090	−526.385911	−526.601619
$\sigma^{5,4}\pi_+^{2,1}\pi_-^{3,1}\delta_-^{1,0}\phi_-^{1,0}$	−524.039121	−524.609692	−525.122231	−525.579112	−525.983922	−526.340938	−526.654505
$\sigma^{6,4}\pi_+^{2,1}\pi_-^{3,1}\delta_-^{1,0}$	−525.163438	−525.577686	−525.923743	−526.215770	−526.477866	−526.710004	−526.908914

Table S519: Total energies in E_h for the Ar atom in the aug-cc-pVQZ basis set in fully uncontracted form, employing the real-orbital approximation.

	$0.00B_0$	$0.10B_0$	$0.20B_0$	$0.30B_0$	$0.40B_0$	$0.50B_0$	$0.60B_0$
$\sigma^{5,5}\pi_+^{2,2}\pi_-^{2,2}$	−526.816805	−526.795162	−526.730911	−526.625825	−526.482286	−526.302900	−526.090228
$\sigma^{6,4}\pi_+^{2,2}\pi_-^{2,2}$	−526.403049	−526.469574	−526.474626	−526.435187	−526.366214	−526.267396	−526.137161
$\sigma^{6,5}\pi_+^{2,2}\pi_-^{2,1}$	−526.397174	−526.414303	−526.370366	−526.281064	−526.164823	−526.023599	−525.854309
$\sigma^{6,5}\pi_+^{2,1}\pi_-^{2,2}$	−526.397174	−526.514303	−526.570366	−526.581064	−526.564823	−526.523599	−526.454309
$\sigma^{5,5}\pi_+^{2,1}\pi_-^{2,2}\delta_-^{1,0}$	−526.263498	−526.484143	−526.648375	−526.762735	−526.836088	−526.875881	−526.886024
$\sigma^{5,4}\pi_+^{2,1}\pi_-^{3,2}\delta_-^{1,0}$	−525.718671	−526.064117	−526.341348	−526.593369	−526.800080	−526.969661	−527.107564
$\sigma^{5,4}\pi_+^{2,1}\pi_-^{2,2}\delta_-^{1,0}\phi_-^{1,0}$	−524.958580	−525.424410	−525.823952	−526.162634	−526.447087	−526.682760	−526.873289
$\sigma^{5,4}\pi_+^{2,1}\pi_-^{3,1}\delta_-^{1,0}\phi_-^{1,0}$	−524.411352	−524.977648	−525.478413	−525.918517	−526.303980	−526.640241	−526.931525
$\sigma^{6,4}\pi_+^{2,1}\pi_-^{3,1}\delta_-^{1,0}$	−525.216788	−525.628914	−525.972180	−526.269205	−526.535795	−526.769930	−526.972593

Table S520: Total energies in E_h for the Ar atom in the aug-cc-pV5Z basis set in fully uncontracted form, employing the real-orbital approximation.

	$0.00B_0$	$0.10B_0$	$0.20B_0$	$0.30B_0$	$0.40B_0$	$0.50B_0$	$0.60B_0$
$\sigma^{5,5}\pi_+^{2,2}\pi_-^{2,2}$	−526.817347	−526.795711	−526.731505	−526.626550	−526.483255	−526.304182	−526.091788
$\sigma^{6,4}\pi_+^{2,2}\pi_-^{2,2}$	−526.411462	−526.476271	−526.477107	−526.434781	−526.367306	−526.271916	−526.145753
$\sigma^{6,5}\pi_+^{2,2}\pi_-^{2,1}$	−526.405555	−526.421061	−526.373063	−526.280257	−526.164639	−526.026630	−525.861481
$\sigma^{6,5}\pi_+^{2,1}\pi_-^{2,2}$	−526.405555	−526.521061	−526.573063	−526.580257	−526.564639	−526.526630	−526.461481
$\sigma^{5,5}\pi_+^{2,1}\pi_-^{2,2}\delta_-^{1,0}$	−526.266967	−526.488337	−526.654654	−526.772103	−526.848920	−526.892050	−526.905339
$\sigma^{5,4}\pi_+^{2,1}\pi_-^{3,2}\delta_-^{1,0}$	−525.682411	−526.051388	−526.360740	−526.617007	−526.828674	−527.003396	−527.146416
$\sigma^{5,4}\pi_+^{2,1}\pi_-^{2,2}\delta_-^{1,0}\phi_-^{1,0}$	−525.192116	−525.654812	−526.044925	−526.367808	−526.630234	−526.838605	−526.998696
$\sigma^{5,4}\pi_+^{2,1}\pi_-^{3,1}\delta_-^{1,0}\phi_-^{1,0}$	−524.653311	−525.216776	−525.708965	−526.134619	−526.499944	−526.811352	−527.075145
$\sigma^{6,4}\pi_+^{2,1}\pi_-^{3,1}\delta_-^{1,0}$	−525.254236	−525.665834	−526.008332	−526.308321	−526.580924	−526.821356	−527.030861

Table S521: Total energies in E_h for the Ar atom in the HGBSP1-5 basis set in fully uncontracted form, employing the real-orbital approximation.

	$0.00B_0$	$0.10B_0$	$0.20B_0$	$0.30B_0$	$0.40B_0$	$0.50B_0$	$0.60B_0$
$\sigma^{5,5}\pi_+^{2,2}\pi_-^{2,2}$	−526.804437	−526.782794	−526.718474	−526.613051	−526.468608	−526.287405	−526.071703
$\sigma^{6,4}\pi_+^{2,2}\pi_-^{2,2}$	−526.381501	−526.449900	−526.462121	−526.430430	−526.361677	−526.258245	−526.122008
$\sigma^{6,5}\pi_+^{2,2}\pi_-^{2,1}$	−526.377171	−526.395612	−526.358195	−526.278548	−526.165210	−526.020711	−525.846540
$\sigma^{6,5}\pi_+^{2,1}\pi_-^{2,2}$	−526.377171	−526.495612	−526.558195	−526.578548	−526.565210	−526.520711	−526.446540
$\sigma^{5,5}\pi_+^{2,1}\pi_-^{2,2}\delta_-^{1,0}$	−526.323974	−526.510932	−526.651904	−526.760907	−526.836736	−526.879633	−526.890635
$\sigma^{5,4}\pi_+^{2,1}\pi_-^{3,2}\delta_-^{1,0}$	−525.712524	−526.067942	−526.360332	−526.607832	−526.816598	−526.989852	−527.129835
$\sigma^{5,4}\pi_+^{2,1}\pi_-^{2,2}\delta_-^{1,0}\phi_-^{1,0}$							
$\sigma^{5,4}\pi_+^{2,1}\pi_-^{3,1}\delta_-^{1,0}\phi_-^{1,0}$							
$\sigma^{6,4}\pi_+^{2,1}\pi_-^{3,1}\delta_-^{1,0}$	−525.191941	−525.607506	−526.005937	−526.312047	−526.583317	−526.820233	−527.024494

Table S522: Total energies in E_h for the Ar atom in the HGBSP1-7 basis set in fully uncontracted form, employing the real-orbital approximation.

	$0.00B_0$	$0.10B_0$	$0.20B_0$	$0.30B_0$	$0.40B_0$	$0.50B_0$	$0.60B_0$
$\sigma^{5,5}\pi_+^{2,2}\pi_-^{2,2}$	−526.817340	−526.795699	−526.731388	−526.625993	−526.481609	−526.300492	−526.084871
$\sigma^{6,4}\pi_+^{2,2}\pi_-^{2,2}$	−526.421860	−526.482453	−526.484046	−526.446230	−526.374974	−526.271517	−526.136408
$\sigma^{6,5}\pi_+^{2,2}\pi_-^{2,1}$	−526.417740	−526.428380	−526.380435	−526.294724	−526.178626	−526.033918	−525.860925
$\sigma^{6,5}\pi_+^{2,1}\pi_-^{2,2}$	−526.417740	−526.528380	−526.580435	−526.594724	−526.578626	−526.533918	−526.460925
$\sigma^{5,5}\pi_+^{2,1}\pi_-^{2,2}\delta_-^{1,0}$	−526.336937	−526.523898	−526.664840	−526.773959	−526.849761	−526.892619	−526.903778
$\sigma^{5,4}\pi_+^{2,1}\pi_-^{3,2}\delta_-^{1,0}$	−525.725523	−526.080936	−526.373299	−526.620863	−526.829643	−527.002838	−527.142896
$\sigma^{5,4}\pi_+^{2,1}\pi_-^{2,2}\delta_-^{1,0}\phi_-^{1,0}$							
$\sigma^{5,4}\pi_+^{2,1}\pi_-^{3,1}\delta_-^{1,0}\phi_-^{1,0}$							
$\sigma^{6,4}\pi_+^{2,1}\pi_-^{3,1}\delta_-^{1,0}$	−525.204914	−525.679829	−526.022850	−526.325840	−526.596330	−526.833589	−527.038659

Table S523: Total energies in E_h for the Ar atom in the HGBSP1-9 basis set in fully uncontracted form, employing the real-orbital approximation.

	$0.00B_0$	$0.10B_0$	$0.20B_0$	$0.30B_0$	$0.40B_0$	$0.50B_0$	$0.60B_0$
$\sigma^{5,5}\pi_+^{2,2}\pi_-^{2,2}$	−526.817511	−526.795870	−526.731559	−526.626165	−526.481782	−526.300667	−526.085056
$\sigma^{6,4}\pi_+^{2,2}\pi_-^{2,2}$	−526.421551	−526.482458	−526.484292	−526.446478	−526.375191	−526.271736	−526.136728
$\sigma^{6,5}\pi_+^{2,2}\pi_-^{2,1}$	−526.417427	−526.428388	−526.380685	−526.294978	−526.178858	−526.034141	−525.861235
$\sigma^{6,5}\pi_+^{2,1}\pi_-^{2,2}$	−526.417427	−526.528388	−526.580685	−526.594978	−526.578858	−526.534141	−526.461235
$\sigma^{5,5}\pi_+^{2,1}\pi_-^{2,2}\delta_-^{1,0}$	−526.337107	−526.524074	−526.665020	−526.774128	−526.849948	−526.892806	−526.903957
$\sigma^{5,4}\pi_+^{2,1}\pi_-^{3,2}\delta_-^{1,0}$	−525.725693	−526.081106	−526.373473	−526.621035	−526.829825	−527.003030	−527.143086
$\sigma^{5,4}\pi_+^{2,1}\pi_-^{2,2}\delta_-^{1,0}\phi_-^{1,0}$							
$\sigma^{5,4}\pi_+^{2,1}\pi_-^{3,1}\delta_-^{1,0}\phi_-^{1,0}$							
$\sigma^{6,4}\pi_+^{2,1}\pi_-^{3,1}\delta_-^{1,0}$	−525.205083	−525.679966	−526.023068	−526.326035	−526.596521	−526.833805	−527.038941

Table S524: Total energies in E_h for the Ar atom in the HGBSP2-5 basis set in fully uncontracted form, employing the real-orbital approximation.

	$0.00B_0$	$0.10B_0$	$0.20B_0$	$0.30B_0$	$0.40B_0$	$0.50B_0$	$0.60B_0$
$\sigma^{5,5}\pi_+^{2,2}\pi_-^{2,2}$	−526.804438	−526.782804	−526.718617	−526.613698	−526.470416	−526.291298	−526.078828
$\sigma^{6,4}\pi_+^{2,2}\pi_-^{2,2}$	−526.383943	−526.452355	−526.464699	−526.433489	−526.365862	−526.264400	−526.131110
$\sigma^{6,5}\pi_+^{2,2}\pi_-^{2,1}$	−526.377855	−526.396293	−526.358895	−526.279365	−526.166415	−526.022851	−525.850422
$\sigma^{6,5}\pi_+^{2,1}\pi_-^{2,2}$	−526.377855	−526.496293	−526.558895	−526.579365	−526.566415	−526.522851	−526.450422
$\sigma^{5,5}\pi_+^{2,1}\pi_-^{2,2}\delta_-^{1,0}$	−526.324648	−526.511639	−526.652863	−526.762388	−526.839238	−526.883912	−526.897677
$\sigma^{5,4}\pi_+^{2,1}\pi_-^{3,2}\delta_-^{1,0}$	−525.714290	−526.069554	−526.362408	−526.610629	−526.820496	−526.995384	−527.137682
$\sigma^{5,4}\pi_+^{2,1}\pi_-^{2,2}\delta_-^{1,0}\phi_-^{1,0}$	−525.671427	−526.027481	−526.300838	−526.529772	−526.721428	−526.877585	−527.006723
$\sigma^{5,4}\pi_+^{2,1}\pi_-^{3,1}\delta_-^{1,0}\phi_-^{1,0}$	−525.123924	−525.581881	−525.959032	−526.292845	−526.589385	−526.850848	−527.085367
$\sigma^{6,4}\pi_+^{2,1}\pi_-^{3,1}\delta_-^{1,0}$	−525.192921	−525.608489	−526.007061	−526.313053	−526.584635	−526.822304	−527.027787

Table S525: Total energies in E_h for the Ar atom in the HGBSP2-7 basis set in fully uncontracted form, employing the real-orbital approximation.

	$0.00B_0$	$0.10B_0$	$0.20B_0$	$0.30B_0$	$0.40B_0$	$0.50B_0$	$0.60B_0$
$\sigma^{5,5}\pi_+^{2,2}\pi_-^{2,2}$	−526.817340	−526.795709	−526.731531	−526.626639	−526.483412	−526.304370	−526.091962
$\sigma^{6,4}\pi_+^{2,2}\pi_-^{2,2}$	−526.424346	−526.484944	−526.486642	−526.449288	−526.379155	−526.277667	−526.145481
$\sigma^{6,5}\pi_+^{2,2}\pi_-^{2,1}$	−526.418433	−526.429068	−526.381131	−526.295523	−526.179821	−526.036062	−525.864798
$\sigma^{6,5}\pi_+^{2,1}\pi_-^{2,2}$	−526.418433	−526.529068	−526.581131	−526.595523	−526.579821	−526.536062	−526.464798
$\sigma^{5,5}\pi_+^{2,1}\pi_-^{2,2}\delta_-^{1,0}$	−526.337610	−526.524605	−526.665797	−526.775437	−526.852258	−526.896890	−526.910801
$\sigma^{5,4}\pi_+^{2,1}\pi_-^{3,2}\delta_-^{1,0}$	−525.727288	−526.082547	−526.375374	−526.623659	−526.833535	−527.008361	−527.150736
$\sigma^{5,4}\pi_+^{2,1}\pi_-^{2,2}\delta_-^{1,0}\phi_-^{1,0}$	−525.684430	−526.041072	−526.314915	−526.543251	−526.734979	−526.893848	−527.022977
$\sigma^{5,4}\pi_+^{2,1}\pi_-^{3,1}\delta_-^{1,0}\phi_-^{1,0}$	−525.136916	−525.595454	−525.973092	−526.306226	−526.602972	−526.866892	−527.101199
$\sigma^{6,4}\pi_+^{2,1}\pi_-^{3,1}\delta_-^{1,0}$	−525.205893	−525.621461	−526.024008	−526.326855	−526.597647	−526.835658	−527.041942

Table S526: Total energies in E_h for the Ar atom in the HGBSP2-9 basis set in fully uncontracted form, employing the real-orbital approximation.

	$0.00B_0$	$0.10B_0$	$0.20B_0$	$0.30B_0$	$0.40B_0$	$0.50B_0$	$0.60B_0$
$\sigma^{5,5}\pi_+^{2,2}\pi_-^{2,2}$	−526.817511	−526.795880	−526.731702	−526.626810	−526.483585	−526.304546	−526.092146
$\sigma^{6,4}\pi_+^{2,2}\pi_-^{2,2}$	−526.424036	−526.484948	−526.486888	−526.449537	−526.379372	−526.277886	−526.145797
$\sigma^{6,5}\pi_+^{2,2}\pi_-^{2,1}$	−526.418120	−526.429076	−526.381381	−526.295777	−526.180052	−526.036285	−525.865107
$\sigma^{6,5}\pi_+^{2,1}\pi_-^{2,2}$	−526.418120	−526.529076	−526.581381	−526.595777	−526.580052	−526.536285	−526.465107
$\sigma^{5,5}\pi_+^{2,1}\pi_-^{2,2}\delta_-^{1,0}$	−526.337781	−526.524781	−526.665977	−526.775607	−526.852445	−526.897077	−526.910980
$\sigma^{5,4}\pi_+^{2,1}\pi_-^{3,2}\delta_-^{1,0}$	−525.727458	−526.082717	−526.375548	−526.623831	−526.833718	−527.008553	−527.150924
$\sigma^{5,4}\pi_+^{2,1}\pi_-^{2,2}\delta_-^{1,0}\phi_-^{1,0}$	−525.684600	−526.041248	−526.315101	−526.543653	−526.735186	−526.894183	−527.023605
$\sigma^{5,4}\pi_+^{2,1}\pi_-^{3,1}\delta_-^{1,0}\phi_-^{1,0}$	−525.137086	−525.595629	−525.973275	−526.306614	−526.603169	−526.867225	−527.101782
$\sigma^{6,4}\pi_+^{2,1}\pi_-^{3,1}\delta_-^{1,0}$	−525.206062	−525.621633	−526.024226	−526.327049	−526.597838	−526.835874	−527.042222

Table S527: Total energies in E_h for the Ar atom in the HGBSP3-5 basis set in fully uncontracted form, employing the real-orbital approximation.

	$0.00B_0$	$0.10B_0$	$0.20B_0$	$0.30B_0$	$0.40B_0$	$0.50B_0$	$0.60B_0$
$\sigma^{5,5}\pi_+^{2,2}\pi_-^{2,2}$	−526.804437	−526.782804	−526.718616	−526.613697	−526.470416	−526.291300	−526.078833
$\sigma^{6,4}\pi_+^{2,2}\pi_-^{2,2}$	−526.383943	−526.452378	−526.465417	−526.437456	−526.375980	−526.282386	−526.157573
$\sigma^{6,5}\pi_+^{2,2}\pi_-^{2,1}$	−526.377855	−526.396297	−526.359317	−526.282548	−526.175634	−526.040122	−525.876308
$\sigma^{6,5}\pi_+^{2,1}\pi_-^{2,2}$	−526.377855	−526.496297	−526.559317	−526.582548	−526.575634	−526.540122	−526.476308
$\sigma^{5,5}\pi_+^{2,1}\pi_-^{2,2}\delta_-^{1,0}$	−526.324653	−526.512015	−526.653547	−526.763337	−526.840457	−526.885462	−526.899641
$\sigma^{5,4}\pi_+^{2,1}\pi_-^{3,2}\delta_-^{1,0}$	−525.714684	−526.070506	−526.364989	−526.615314	−526.827571	−527.005090	−527.150326
$\sigma^{5,4}\pi_+^{2,1}\pi_-^{2,2}\delta_-^{1,0}\phi_-^{1,0}$	−525.671474	−526.027576	−526.301100	−526.530335	−526.722385	−526.879029	−527.008739
$\sigma^{5,4}\pi_+^{2,1}\pi_-^{3,1}\delta_-^{1,0}\phi_-^{1,0}$	−525.112827	−525.582089	−525.959609	−526.294213	−526.591975	−526.855122	−527.091744
$\sigma^{6,4}\pi_+^{2,1}\pi_-^{3,1}\delta_-^{1,0}$	−525.193051	−525.608761	−526.007718	−526.316161	−526.592254	−526.835899	−527.048491

Table S528: Total energies in E_h for the Ar atom in the HGBSP3-7 basis set in fully uncontracted form, employing the real-orbital approximation.

	$0.00B_0$	$0.10B_0$	$0.20B_0$	$0.30B_0$	$0.40B_0$	$0.50B_0$	$0.60B_0$
$\sigma^{5,5}\pi_+^{2,2}\pi_-^{2,2}$	−526.817340	−526.795709	−526.731531	−526.626639	−526.483413	−526.304372	−526.091968
$\sigma^{6,4}\pi_+^{2,2}\pi_-^{2,2}$	−526.424347	−526.484987	−526.487925	−526.454736	−526.390671	−526.295905	−526.170834
$\sigma^{6,5}\pi_+^{2,2}\pi_-^{2,1}$	−526.418433	−526.429090	−526.382126	−526.300409	−526.190882	−526.053936	−525.889630
$\sigma^{6,5}\pi_+^{2,1}\pi_-^{2,2}$	−526.418433	−526.529090	−526.582126	−526.600409	−526.590882	−526.553936	−526.489630
$\sigma^{5,5}\pi_+^{2,1}\pi_-^{2,2}\delta_-^{1,0}$	−526.337615	−526.524979	−526.666485	−526.776370	−526.853476	−526.898445	−526.912746
$\sigma^{5,4}\pi_+^{2,1}\pi_-^{3,2}\delta_-^{1,0}$	−525.727682	−526.083498	−526.377957	−526.628323	−526.840583	−527.018077	−527.163379
$\sigma^{5,4}\pi_+^{2,1}\pi_-^{2,2}\delta_-^{1,0}\phi_-^{1,0}$	−525.684477	−526.041168	−526.315178	−526.543812	−526.735940	−526.895293	−527.024979
$\sigma^{5,4}\pi_+^{2,1}\pi_-^{3,1}\delta_-^{1,0}\phi_-^{1,0}$	−525.125815	−525.595661	−525.973670	−526.307589	−526.605574	−526.871163	−527.107539
$\sigma^{6,4}\pi_+^{2,1}\pi_-^{3,1}\delta_-^{1,0}$	−525.206023	−525.621732	−526.024880	−526.330481	−526.605571	−526.849005	−527.061801

Table S529: Total energies in E_h for the Ar atom in the HGBSP3-9 basis set in fully uncontracted form, employing the real-orbital approximation.

	$0.00B_0$	$0.10B_0$	$0.20B_0$	$0.30B_0$	$0.40B_0$	$0.50B_0$	$0.60B_0$
$\sigma^{5,5}\pi_+^{2,2}\pi_-^{2,2}$	−526.817511	−526.795880	−526.731702	−526.626810	−526.483586	−526.304548	−526.092153
$\sigma^{6,4}\pi_+^{2,2}\pi_-^{2,2}$	−526.424037	−526.484988	−526.488109	−526.454921	−526.390871	−526.296116	−526.171059
$\sigma^{6,5}\pi_+^{2,2}\pi_-^{2,1}$	−526.418120	−526.429095	−526.382314	−526.300587	−526.191087	−526.054157	−525.889859
$\sigma^{6,5}\pi_+^{2,1}\pi_-^{2,2}$	−526.418120	−526.529095	−526.582314	−526.600587	−526.591087	−526.554157	−526.489859
$\sigma^{5,5}\pi_+^{2,1}\pi_-^{2,2}\delta_-^{1,0}$	−526.337786	−526.525155	−526.666664	−526.776540	−526.853661	−526.898629	−526.912926
$\sigma^{5,4}\pi_+^{2,1}\pi_-^{3,2}\delta_-^{1,0}$	−525.727852	−526.083669	−526.378130	−526.628494	−526.840764	−527.018262	−527.163563
$\sigma^{5,4}\pi_+^{2,1}\pi_-^{2,2}\delta_-^{1,0}\phi_-^{1,0}$	−525.684647	−526.041344	−526.315365	−526.544214	−526.736147	−526.895629	−527.025608
$\sigma^{5,4}\pi_+^{2,1}\pi_-^{3,1}\delta_-^{1,0}\phi_-^{1,0}$	−525.125986	−525.595836	−525.973853	−526.307976	−526.605769	−526.871496	−527.108119
$\sigma^{6,4}\pi_+^{2,1}\pi_-^{3,1}\delta_-^{1,0}$	−525.206193	−525.621905	−526.025079	−526.330660	−526.605758	−526.849201	−527.062011

Table S530: Total energies in E_h for the Ar atom in the AHGBSP1-5 basis set in fully uncontracted form, employing the real-orbital approximation.

	$0.00B_0$	$0.10B_0$	$0.20B_0$	$0.30B_0$	$0.40B_0$	$0.50B_0$	$0.60B_0$
$\sigma^{5,5}\pi_+^{2,2}\pi_-^{2,2}$	−526.804443	−526.782800	−526.718481	−526.613063	−526.468627	−526.287435	−526.071748
$\sigma^{6,4}\pi_+^{2,2}\pi_-^{2,2}$	−526.409714	−526.469377	−526.470799	−526.433125	−526.362006	−526.258341	−526.122746
$\sigma^{6,5}\pi_+^{2,2}\pi_-^{2,1}$	−526.405606	−526.415293	−526.367171	−526.281592	−526.165673	−526.020772	−525.847237
$\sigma^{6,5}\pi_+^{2,1}\pi_-^{2,2}$	−526.405606	−526.515293	−526.567171	−526.581592	−526.565673	−526.520772	−526.447237
$\sigma^{5,5}\pi_+^{2,1}\pi_-^{2,2}\delta_-^{1,0}$	−526.323986	−526.510942	−526.651913	−526.760920	−526.836753	−526.879656	−526.890671
$\sigma^{5,4}\pi_+^{2,1}\pi_-^{3,2}\delta_-^{1,0}$	−525.712530	−526.067949	−526.360339	−526.607842	−526.816612	−526.989871	−527.129863
$\sigma^{5,4}\pi_+^{2,1}\pi_-^{2,2}\delta_-^{1,0}\phi_-^{1,0}$							
$\sigma^{5,4}\pi_+^{2,1}\pi_-^{3,1}\delta_-^{1,0}\phi_-^{1,0}$							
$\sigma^{6,4}\pi_+^{2,1}\pi_-^{3,1}\delta_-^{1,0}$	−525.191947	−525.666678	−526.009722	−526.312821	−526.583339	−526.820429	−527.025136

Table S531: Total energies in E_h for the Ar atom in the AHGBSP1-7 basis set in fully uncontracted form, employing the real-orbital approximation.

	$0.00B_0$	$0.10B_0$	$0.20B_0$	$0.30B_0$	$0.40B_0$	$0.50B_0$	$0.60B_0$
$\sigma^{5,5}\pi_+^{2,2}\pi_-^{2,2}$	−526.817340	−526.795700	−526.731389	−526.625993	−526.481609	−526.300492	−526.084873
$\sigma^{6,4}\pi_+^{2,2}\pi_-^{2,2}$	−526.422691	−526.482488	−526.484086	−526.446288	−526.374990	−526.271521	−526.136452
$\sigma^{6,5}\pi_+^{2,2}\pi_-^{2,1}$	−526.418581	−526.428411	−526.380476	−526.294787	−526.178649	−526.033920	−525.860966
$\sigma^{6,5}\pi_+^{2,1}\pi_-^{2,2}$	−526.418581	−526.528411	−526.580476	−526.594787	−526.578649	−526.533920	−526.460966
$\sigma^{5,5}\pi_+^{2,1}\pi_-^{2,2}\delta_-^{1,0}$	−526.336937	−526.523898	−526.664840	−526.773959	−526.849762	−526.892620	−526.903778
$\sigma^{5,4}\pi_+^{2,1}\pi_-^{3,2}\delta_-^{1,0}$	−525.725523	−526.080936	−526.373300	−526.620863	−526.829643	−527.002838	−527.142897
$\sigma^{5,4}\pi_+^{2,1}\pi_-^{2,2}\delta_-^{1,0}\phi_-^{1,0}$							
$\sigma^{5,4}\pi_+^{2,1}\pi_-^{3,1}\delta_-^{1,0}\phi_-^{1,0}$							
$\sigma^{6,4}\pi_+^{2,1}\pi_-^{3,1}\delta_-^{1,0}$	−525.204914	−525.679829	−526.022881	−526.325857	−526.596331	−526.833597	−527.038693

Table S532: Total energies in E_h for the Ar atom in the AHGBSP1-9 basis set in fully uncontracted form, employing the real-orbital approximation.

	$0.00B_0$	$0.10B_0$	$0.20B_0$	$0.30B_0$	$0.40B_0$	$0.50B_0$	$0.60B_0$
$\sigma^{5,5}\pi_+^{2,2}\pi_-^{2,2}$	−526.817511	−526.795870	−526.731559	−526.626165	−526.481782	−526.300667	−526.085056
$\sigma^{6,4}\pi_+^{2,2}\pi_-^{2,2}$	−526.422862	−526.482660	−526.484293	−526.446491	−526.375197	−526.271737	−526.136739
$\sigma^{6,5}\pi_+^{2,2}\pi_-^{2,1}$	−526.418753	−526.428584	−526.380686	−526.294991	−526.178868	−526.034141	−525.861246
$\sigma^{6,5}\pi_+^{2,1}\pi_-^{2,2}$	−526.418753	−526.528584	−526.580686	−526.594991	−526.578868	−526.534141	−526.461246
$\sigma^{5,5}\pi_+^{2,1}\pi_-^{2,2}\delta_-^{1,0}$	−526.337108	−526.524074	−526.665020	−526.774128	−526.849948	−526.892806	−526.903957
$\sigma^{5,4}\pi_+^{2,1}\pi_-^{3,2}\delta_-^{1,0}$	−525.725693	−526.081106	−526.373473	−526.621035	−526.829825	−527.003030	−527.143086
$\sigma^{5,4}\pi_+^{2,1}\pi_-^{2,2}\delta_-^{1,0}\phi_-^{1,0}$							
$\sigma^{5,4}\pi_+^{2,1}\pi_-^{3,1}\delta_-^{1,0}\phi_-^{1,0}$							
$\sigma^{6,4}\pi_+^{2,1}\pi_-^{3,1}\delta_-^{1,0}$	−525.205083	−525.680005	−526.023070	−526.326039	−526.596522	−526.833807	−527.038948

Table S533: Total energies in E_h for the Ar atom in the AHGBSP2-5 basis set in fully uncontracted form, employing the real-orbital approximation.

	$0.00B_0$	$0.10B_0$	$0.20B_0$	$0.30B_0$	$0.40B_0$	$0.50B_0$	$0.60B_0$
$\sigma^{5,5}\pi_+^{2,2}\pi_-^{2,2}$	−526.804444	−526.782811	−526.718625	−526.613709	−526.470434	−526.291327	−526.078870
$\sigma^{6,4}\pi_+^{2,2}\pi_-^{2,2}$	−526.412203	−526.471868	−526.473394	−526.436184	−526.366188	−526.264497	−526.131839
$\sigma^{6,5}\pi_+^{2,2}\pi_-^{2,1}$	−526.406300	−526.415982	−526.367869	−526.282393	−526.166867	−526.022915	−525.851119
$\sigma^{6,5}\pi_+^{2,1}\pi_-^{2,2}$	−526.406300	−526.515982	−526.567869	−526.582393	−526.566867	−526.522915	−526.451119
$\sigma^{5,5}\pi_+^{2,1}\pi_-^{2,2}\delta_-^{1,0}$	−526.324660	−526.511650	−526.652872	−526.762401	−526.839255	−526.883934	−526.897711
$\sigma^{5,4}\pi_+^{2,1}\pi_-^{3,2}\delta_-^{1,0}$	−525.714297	−526.069561	−526.362415	−526.610639	−526.820510	−526.995402	−527.137710
$\sigma^{5,4}\pi_+^{2,1}\pi_-^{2,2}\delta_-^{1,0}\phi_-^{1,0}$	−525.671438	−526.027516	−526.300863	−526.529791	−526.721443	−526.877615	−527.006756
$\sigma^{5,4}\pi_+^{2,1}\pi_-^{3,1}\delta_-^{1,0}\phi_-^{1,0}$	−525.123932	−525.581914	−525.959055	−526.292859	−526.589396	−526.850872	−527.085393
$\sigma^{6,4}\pi_+^{2,1}\pi_-^{3,1}\delta_-^{1,0}$	−525.192927	−525.667917	−526.010879	−526.313836	−526.584657	−526.822500	−527.028425

Table S534: Total energies in E_h for the Ar atom in the AHGBSP2-7 basis set in fully uncontracted form, employing the real-orbital approximation.

	$0.00B_0$	$0.10B_0$	$0.20B_0$	$0.30B_0$	$0.40B_0$	$0.50B_0$	$0.60B_0$
$\sigma^{5,5}\pi_+^{2,2}\pi_-^{2,2}$	−526.817340	−526.795709	−526.731531	−526.626639	−526.483413	−526.304371	−526.091963
$\sigma^{6,4}\pi_+^{2,2}\pi_-^{2,2}$	−526.425179	−526.484978	−526.486682	−526.449347	−526.379171	−526.277671	−526.145524
$\sigma^{6,5}\pi_+^{2,2}\pi_-^{2,1}$	−526.419275	−526.429099	−526.381172	−526.295586	−526.179843	−526.036064	−525.864839
$\sigma^{6,5}\pi_+^{2,1}\pi_-^{2,2}$	−526.419275	−526.529099	−526.581172	−526.595586	−526.579843	−526.536064	−526.464839
$\sigma^{5,5}\pi_+^{2,1}\pi_-^{2,2}\delta_-^{1,0}$	−526.337610	−526.524605	−526.665797	−526.775438	−526.852259	−526.896891	−526.910802
$\sigma^{5,4}\pi_+^{2,1}\pi_-^{3,2}\delta_-^{1,0}$	−525.727288	−526.082547	−526.375374	−526.623659	−526.833536	−527.008362	−527.150736
$\sigma^{5,4}\pi_+^{2,1}\pi_-^{2,2}\delta_-^{1,0}\phi_-^{1,0}$	−525.684430	−526.041073	−526.314915	−526.543254	−526.734979	−526.893849	−527.022979
$\sigma^{5,4}\pi_+^{2,1}\pi_-^{3,1}\delta_-^{1,0}\phi_-^{1,0}$	−525.136916	−525.595455	−525.973093	−526.306229	−526.602973	−526.866893	−527.101201
$\sigma^{6,4}\pi_+^{2,1}\pi_-^{3,1}\delta_-^{1,0}$	−525.205893	−525.681067	−526.024038	−526.326872	−526.597648	−526.835667	−527.041976

Table S535: Total energies in E_h for the Ar atom in the AHGBSP2-9 basis set in fully uncontracted form, employing the real-orbital approximation.

	$0.00B_0$	$0.10B_0$	$0.20B_0$	$0.30B_0$	$0.40B_0$	$0.50B_0$	$0.60B_0$
$\sigma^{5,5}\pi_+^{2,2}\pi_-^{2,2}$	−526.817511	−526.795880	−526.731702	−526.626810	−526.483585	−526.304546	−526.092146
$\sigma^{6,4}\pi_+^{2,2}\pi_-^{2,2}$	−526.425350	−526.485151	−526.486889	−526.449550	−526.379378	−526.277887	−526.145809
$\sigma^{6,5}\pi_+^{2,2}\pi_-^{2,1}$	−526.419447	−526.429272	−526.381382	−526.295790	−526.180061	−526.036285	−525.865118
$\sigma^{6,5}\pi_+^{2,1}\pi_-^{2,2}$	−526.419447	−526.529272	−526.581382	−526.595790	−526.580061	−526.536285	−526.465118
$\sigma^{5,5}\pi_+^{2,1}\pi_-^{2,2}\delta_-^{1,0}$	−526.337781	−526.524781	−526.665977	−526.775607	−526.852445	−526.897077	−526.910980
$\sigma^{5,4}\pi_+^{2,1}\pi_-^{3,2}\delta_-^{1,0}$	−525.727458	−526.082717	−526.375548	−526.623831	−526.833718	−527.008554	−527.150924
$\sigma^{5,4}\pi_+^{2,1}\pi_-^{2,2}\delta_-^{1,0}\phi_-^{1,0}$	−525.684600	−526.041248	−526.315101	−526.543653	−526.735186	−526.894184	−527.023606
$\sigma^{5,4}\pi_+^{2,1}\pi_-^{3,1}\delta_-^{1,0}\phi_-^{1,0}$	−525.137086	−525.595629	−525.973276	−526.306614	−526.603169	−526.867225	−527.101782
$\sigma^{6,4}\pi_+^{2,1}\pi_-^{3,1}\delta_-^{1,0}$	−525.206062	−525.681243	−526.024228	−526.327054	−526.597838	−526.835876	−527.042230

Table S536: Total energies in E_h for the Ar atom in the AHGBSP3-5 basis set in fully uncontracted form, employing the real-orbital approximation.

	$0.00B_0$	$0.10B_0$	$0.20B_0$	$0.30B_0$	$0.40B_0$	$0.50B_0$	$0.60B_0$
$\sigma^{5,5}\pi_+^{2,2}\pi_-^{2,2}$	−526.804443	−526.782810	−526.718624	−526.613708	−526.470434	−526.291329	−526.078876
$\sigma^{6,4}\pi_+^{2,2}\pi_-^{2,2}$	−526.412203	−526.471915	−526.474708	−526.441642	−526.377647	−526.282848	−526.157628
$\sigma^{6,5}\pi_+^{2,2}\pi_-^{2,1}$	−526.406300	−526.416007	−526.368894	−526.287301	−526.177843	−526.040886	−525.876437
$\sigma^{6,5}\pi_+^{2,1}\pi_-^{2,2}$	−526.406300	−526.516007	−526.568894	−526.587301	−526.577843	−526.540886	−526.476437
$\sigma^{5,5}\pi_+^{2,1}\pi_-^{2,2}\delta_-^{1,0}$	−526.324665	−526.512026	−526.653556	−526.763349	−526.840473	−526.885485	−526.899675
$\sigma^{5,4}\pi_+^{2,1}\pi_-^{3,2}\delta_-^{1,0}$	−525.714691	−526.070513	−526.364996	−526.615323	−526.827584	−527.005108	−527.150353
$\sigma^{5,4}\pi_+^{2,1}\pi_-^{2,2}\delta_-^{1,0}\phi_-^{1,0}$	−525.671485	−526.027612	−526.301126	−526.530353	−526.722400	−526.879059	−527.008772
$\sigma^{5,4}\pi_+^{2,1}\pi_-^{3,1}\delta_-^{1,0}\phi_-^{1,0}$	−525.124042	−525.582122	−525.959632	−526.294227	−526.591987	−526.855146	−527.091769
$\sigma^{6,4}\pi_+^{2,1}\pi_-^{3,1}\delta_-^{1,0}$	−525.193057	−525.668070	−526.011755	−526.317456	−526.592578	−526.835938	−527.048545

Table S537: Total energies in E_h for the Ar atom in the AHGBSP3-7 basis set in fully uncontracted form, employing the real-orbital approximation.

	$0.00B_0$	$0.10B_0$	$0.20B_0$	$0.30B_0$	$0.40B_0$	$0.50B_0$	$0.60B_0$
$\sigma^{5,5}\pi_+^{2,2}\pi_-^{2,2}$	−526.817340	−526.795709	−526.731531	−526.626639	−526.483413	−526.304373	−526.091969
$\sigma^{6,4}\pi_+^{2,2}\pi_-^{2,2}$	−526.425180	−526.485026	−526.487931	−526.454745	−526.390685	−526.295916	−526.170837
$\sigma^{6,5}\pi_+^{2,2}\pi_-^{2,1}$	−526.419276	−526.429125	−526.382133	−526.300415	−526.190898	−526.053955	−525.889639
$\sigma^{6,5}\pi_+^{2,1}\pi_-^{2,2}$	−526.419276	−526.529125	−526.582133	−526.600415	−526.590898	−526.553955	−526.489639
$\sigma^{5,5}\pi_+^{2,1}\pi_-^{2,2}\delta_-^{1,0}$	−526.337615	−526.524980	−526.666486	−526.776370	−526.853477	−526.898445	−526.912746
$\sigma^{5,4}\pi_+^{2,1}\pi_-^{3,2}\delta_-^{1,0}$	−525.727682	−526.083499	−526.377958	−526.628323	−526.840583	−527.018077	−527.163379
$\sigma^{5,4}\pi_+^{2,1}\pi_-^{2,2}\delta_-^{1,0}\phi_-^{1,0}$	−525.684477	−526.041169	−526.315179	−526.543815	−526.735941	−526.895294	−527.024982
$\sigma^{5,4}\pi_+^{2,1}\pi_-^{3,1}\delta_-^{1,0}\phi_-^{1,0}$	−525.137026	−525.595662	−525.973671	−526.307592	−526.605575	−526.871164	−527.107541
$\sigma^{6,4}\pi_+^{2,1}\pi_-^{3,1}\delta_-^{1,0}$	−525.206023	−525.681220	−526.024894	−526.330485	−526.605573	−526.849006	−527.061802

Table S538: Total energies in E_h for the Ar atom in the AHGBSP3-9 basis set in fully uncontracted form, employing the real-orbital approximation.

	$0.00B_0$	$0.10B_0$	$0.20B_0$	$0.30B_0$	$0.40B_0$	$0.50B_0$	$0.60B_0$
$\sigma^{5,5}\pi_+^{2,2}\pi_-^{2,2}$	−526.817511	−526.795880	−526.731702	−526.626810	−526.483586	−526.304548	−526.092153
$\sigma^{6,4}\pi_+^{2,2}\pi_-^{2,2}$	−526.425351	−526.485199	−526.488128	−526.454925	−526.390872	−526.296121	−526.171062
$\sigma^{6,5}\pi_+^{2,2}\pi_-^{2,1}$	−526.419447	−526.429298	−526.382333	−526.300594	−526.191087	−526.054164	−525.889866
$\sigma^{6,5}\pi_+^{2,1}\pi_-^{2,2}$	−526.419447	−526.529298	−526.582333	−526.600594	−526.591087	−526.554164	−526.489866
$\sigma^{5,5}\pi_+^{2,1}\pi_-^{2,2}\delta_-^{1,0}$	−526.337786	−526.525155	−526.666664	−526.776540	−526.853661	−526.898629	−526.912926
$\sigma^{5,4}\pi_+^{2,1}\pi_-^{3,2}\delta_-^{1,0}$	−525.727852	−526.083669	−526.378130	−526.628494	−526.840764	−527.018262	−527.163563
$\sigma^{5,4}\pi_+^{2,1}\pi_-^{2,2}\delta_-^{1,0}\phi_-^{1,0}$	−525.684647	−526.041344	−526.315365	−526.544214	−526.736147	−526.895629	−527.025608
$\sigma^{5,4}\pi_+^{2,1}\pi_-^{3,1}\delta_-^{1,0}\phi_-^{1,0}$	−525.137196	−525.595836	−525.973853	−526.307976	−526.605769	−526.871496	−527.108119
$\sigma^{6,4}\pi_+^{2,1}\pi_-^{3,1}\delta_-^{1,0}$	−525.273237	−525.681396	−526.025080	−526.330660	−526.605758	−526.849202	−527.062011

Table S539: Total energies in E_h for the Ar atom in the 6-311++G(3df,3pd) basis set in fully uncontracted form, employing the real-orbital approximation.

	$0.00B_0$	$0.10B_0$	$0.20B_0$	$0.30B_0$	$0.40B_0$	$0.50B_0$	$0.60B_0$
$\sigma^{5,5}\pi_+^{2,2}\pi_-^{2,2}$	−526.808584	−526.786926	−526.722553	−526.617041	−526.472495	−526.291247	−526.075687
$\sigma^{6,4}\pi_+^{2,2}\pi_-^{2,2}$	−526.388850	−526.455840	−526.458331	−526.402896	−526.312405	−526.209482	−526.081094
$\sigma^{6,5}\pi_+^{2,2}\pi_-^{2,1}$	−526.383257	−526.401488	−526.357403	−526.255844	−526.113190	−525.961274	−525.794297
$\sigma^{6,5}\pi_+^{2,1}\pi_-^{2,2}$	−526.383257	−526.501488	−526.557403	−526.555844	−526.513190	−526.461274	−526.394297
$\sigma^{5,5}\pi_+^{2,1}\pi_-^{2,2}\delta_-^{1,0}$	−526.202815	−526.428694	−526.606860	−526.738795	−526.826727	−526.873471	−526.882318
$\sigma^{5,4}\pi_+^{2,1}\pi_-^{3,2}\delta_-^{1,0}$	−525.679215	−526.032871	−526.292505	−526.565303	−526.790429	−526.970792	−527.109867
$\sigma^{5,4}\pi_+^{2,1}\pi_-^{2,2}\delta_-^{1,0}\phi_-^{1,0}$	−523.409970	−523.884811	−524.309897	−524.686759	−525.017635	−525.305284	−525.552881
$\sigma^{5,4}\pi_+^{2,1}\pi_-^{3,1}\delta_-^{1,0}\phi_-^{1,0}$	−522.951308	−523.526223	−524.051398	−524.528080	−524.958252	−525.344527	−525.690025
$\sigma^{6,4}\pi_+^{2,1}\pi_-^{3,1}\delta_-^{1,0}$	−525.205907	−525.620736	−525.967110	−526.256399	−526.524193	−526.762675	−526.964023

Table S540: Total energies in E_h for the Ar atom in the def2-TZVP basis set in fully uncontracted form, employing the real-orbital approximation.

	$0.00B_0$	$0.10B_0$	$0.20B_0$	$0.30B_0$	$0.40B_0$	$0.50B_0$	$0.60B_0$
$\sigma^{5,5}\pi_+^{2,2}\pi_-^{2,2}$	−526.806169	−526.784604	−526.720343	−526.614633	−526.469391	−526.286986	−526.070004
$\sigma^{6,4}\pi_+^{2,2}\pi_-^{2,2}$	−526.073859	−526.150279	−526.179891	−526.163707	−526.153850	−526.070933	−525.953569
$\sigma^{6,5}\pi_+^{2,2}\pi_-^{2,1}$	−526.036328	−526.064296	−526.048492	−525.989763	−525.927171	−525.808054	−525.656667
$\sigma^{6,5}\pi_+^{2,1}\pi_-^{2,2}$	−526.036328	−526.164296	−526.248492	−526.289763	−526.327171	−526.308054	−526.256667
$\sigma^{5,5}\pi_+^{2,1}\pi_-^{2,2}\delta_-^{1,0}$	−526.071337	−526.299816	−526.485611	−526.629755	−526.733837	−526.799846	−526.829987
$\sigma^{5,4}\pi_+^{2,1}\pi_-^{3,2}\delta_-^{1,0}$	−525.311822	−525.683259	−525.997846	−526.256385	−526.460142	−526.610763	−526.995726
$\sigma^{5,4}\pi_+^{2,1}\pi_-^{2,2}\delta_-^{1,0}\phi_-^{1,0}$	−523.191175	−523.668556	−524.101069	−524.489770	−524.836277	−525.142583	−525.410856
$\sigma^{5,4}\pi_+^{2,1}\pi_-^{3,1}\delta_-^{1,0}\phi_-^{1,0}$	−522.591635	−523.163599	−523.679688	−524.140482	−524.546920	−525.126727	−525.505508
$\sigma^{6,4}\pi_+^{2,1}\pi_-^{3,1}\delta_-^{1,0}$	−524.649226	−525.069509	−525.430542	−525.732873	−525.977401	−526.446292	−526.657047
